# Supplementary material for: Photocatalytic three-component asymmetric sulfonylation via direct C(sp3)-H functionalization
Source: Nat Commun. 2021 Apr 22;12:2377. doi: 10.1038/s41467-021-22690-3 (PMC8062459; doi:10.1038/s41467-021-22690-3)
Supplement: Supplementary file 1 — Supplementary Information [file 41467_2021_22690_MOESM1_ESM.pdf]

Supplementary Information

Photocatalytic three-component asymmetric sulfonylation via direct $C(sp^3)$-H functionalization

Cao et al.

Table of Contents

Supplementary Methods S3

1. General Information S3

2. Synthesis of the Ligands, Substrates and Racemic Products S4

  2.1 Synthesis of the Chiral Ligands S4

  2.2 Preparation of α,β-Unsaturated *N*-Acyl Pyrazoles S8

  2.3 Synthesis of the Racemic Products as References S9

3. Photocatalytic Asymmetric Three-Component Reactions S11

  3.1 Reaction Optimization S11

  3.2 Substrate Scope of C(sp^3)-H Precursors S14

  3.3 Substrate Scope of α,β-Unsaturated *N*-Acylpyrazoles Containing a β-Alkyl Substituent S56

  3.4 Substrate Scope of α,β-Unsaturated *N*-Acylpyrazoles Containing a β-Aryl Substituent S63

  3.5 A Scale-up Catalytic Reaction S68

  3.6 Absolute Configuration Assignment of the Chiral Products S68

  3.7 Set-up of the Photoreactions and Emission Spectra of the Light Source S69

4. Synthetic Transformations S70

  4.1 Transformation of Product **3v** to an Alcohol Derivative S70

  4.2 Transformation of Product **3v** to an Ester Derivative S71

  4.3 Transformation of Product **3v** to an Amide Derivative S72

  4.4 Late-Stage Modification of Bioactive Molecules S73

5. Mechanistic Investigations S77

  5.1 Radical Trapping Experiments S77

  5.2 Radical Clock Experiments S79

  5.3 Control Experiments S81

  5.4 Evidence for Lewis Acid Activation S81

  5.5 Trend of *Z*/*E* Isomerization of **2l** S84

  5.6 Luminescence Quenching Experiments S86

5.7 Density Functional Theory (DFT) Calculations S88
5.8 An alternative Reaction Pathway S97

6. Chiral Chromatography S99
6.1 Determination of Enantioselectivities of the Photocatalytic Asymmetric Reactions S99
6.2 Determination of Enantiopurities of the Transformation Products S152

7. X-Ray Diffraction S159
7.1 Crystal Structure of Chiral Nickel Catalyst **[L6-Ni]** S159
7.2 Crystal Structure of Chiral Product **3b** S160

8. ^{1}H and ^{13}C NMR Spectrum S162

Supplementary References S241

Supplementary Methods

1. General Information

Synthesis of the substrates and chiral ligands were carried out under an atmosphere of argon with magnetic stirring unless stated otherwise. Visible-light photocatalytic reactions were performed in 10, 25 or 50 mL Schlenk tubes at the indicated temperature under an atmosphere of argon and under irradiation with a 24 W blue LEDs lamp (λ_{max} = 455 nm; commercial supplier: Hong Chang Lighting Co. Ltd., website: http://hongchang-led.taobao.com) or a 50 W blue LEDs lamp (λ_{max} = 420 nm; commercial supplier: Taiwan Guang Hong, website: https://kiwilight.taobao.com). α,β-unsaturated *N*-acyl pyrazoles (**2a**, **2f**, **2g**, **2i**, **2k**, **2l−2o**,[1] **2j**[2]) and a chiral ligand (**L4**)[3] were synthesized according to the published procedures. **L7−L9** were prepared by a four-step synthesis according to a modified method.[4–6] All other reagents were purchased from commercial suppliers (TCI, Aldrich, Alfa, Strem, Energy Chemical, Adamas-beta® and J&K) and used without further purification. Flash column chromatography was performed with silica gel (300-400 mesh, pH = 6.7–7.0). ^{1}H NMR and ^{13}C NMR spectra were recorded on a Bruker AM (500 MHz) or Bruker AM (600 MHz) spectrometer at ambient temperature. NMR standards were used as follows: $CDCl_3$ = 7.26 ppm (^{1}H NMR), 77.0 ppm (^{13}C NMR). IR spectra were recorded on a Nicolet Avatar 330 FT-IR spectrophotometer. Chiral HPLC chromatograms were obtained from an Agilent 1260 Series HPLC system. Enantiomeric excess of the products were determined by HPLC analysis on chiral stationary phases. High-resolution mass spectra were recorded on a Bruker En Apex Ultra 7.0 T FT-MS and Agilent 1290-G6545XT QTOF instrument using ESI technique. Power X-ray diffraction data were recorded on a XtaLAB Synergy four-circle diffractometer with monochromatic Cu Kα radiation (λ = 1.54184 Å) at 100 K. Emission spectra were recorded on a Hitachi F-7000. Optical rotations were measured on Anton Paar MCP 500 polarimeter at concentrations of 1.0 g/100 mL.

2. Synthesis of the Ligands, Substrates and Racemic Products

2.1 Synthesis of the Chiral Ligands

Chiral ligands **L1−L3**, **L5**, **L6** and **L10−L12** were purchased from Daicel or J&K, and used directly without further purification. **L4** was synthesized by a published procedure.[3] **L7−L9** were prepared by a four-step synthesis according to a modified method.[4–6]

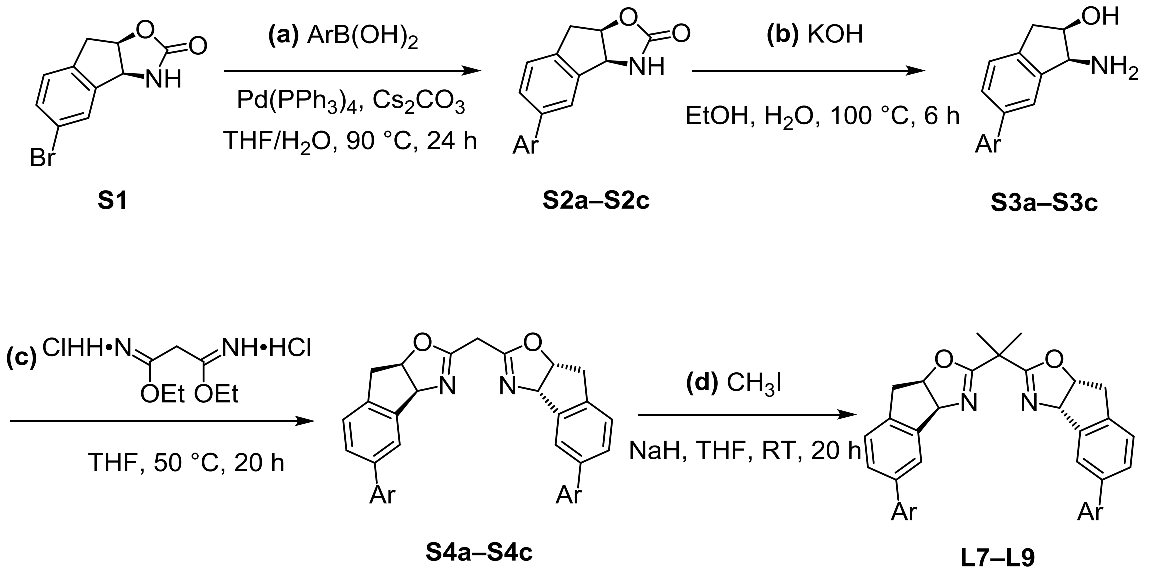

S2a, S3a, S4a, L7: Ar = Ph; **S2b, S3b, S4b, L8**: Ar = Ph(4-Me); **S2c, S3c, S4c, L9**: Ar = Ph(4-*t*Bu)

General Procedure

Step (a):

S2a−2c were synthesized by a published procedure with some modifications.[4] Accordingly, **S1** (1.00 g, 4.00 mmol), $Pd(PPh_3)_4$ (0.46 g, 0.40 mmol), Cs_2CO_3 (2.61 g, 8.00 mmol), aryl boronic acid (4.40 mmol), THF (20 mL) and H_2O (5 mL) were added to a nitrogen-filled round-bottom flask. The solution was heated at 90 ℃ for 24 h. The reaction mixture was cooled down to room temperature, quenched with water (50 mL) and extracted with EtOAc (25 mL × 3). The combined organic layers were washed with brine, dried over Na_2SO_4 and filtered. The solvent was removed under reduced pressure, and the residue was

purified by flash silica gel column chromatography (eluted with PE:EtOAc = 1:1) to afford product **S2a**, **S2b** or **S2c** as a yellow solid, which was used directly for the next step without any further purification.

Step (b):

S3a−3c were synthesized by a published procedure with some modifications.[5] The resulting product of step (**a**) (**S2a**, **S2b** or **S2c**) and KOH (0.81 g, 14.4 mmol) were dissolved in a mixture of EtOH (10 mL) and H_2O (10 mL), and then heated at 100 ℃ for 6 h. The solution was cooled down to room temperature and concentrated in vacuum to remove EtOH. The residue was extracted with EtOAc (10 mL × 3), dried *in vacuo*, and purified by flash silica gel chromatography (eluted with EtOAc:MeOH = 1:2). Crude product **S3a**, **S3b** or **S3c** was obtained as a brown solid, which was used directly for the next step without any further purification.

Step (c):

S4a−4c were synthesized by a published procedure with some modifications.[6] To a round-bottom flask under argon, the resulting product of step (**b**) (**S3a**, **S3b** or **S3c**) and diethyl malonimidate dihydrochloride (0.44 g, 1.90 mmol) and dry THF (20 mL) were added. The reaction mixture was stirred at 50 ℃ for 20 h, then cooled down to 5 ℃. Aqueous sodium bicarbonate (0.5 M, 17 mL) was slowly added. The resulting precipitate was collected after filteration, washing with water (10 mL × 2), and dried *in vacuo* to afford the crude product (**S4a**, **S4b** or **S4c**) as a brown solid, which was directly used for the next step without any further purification.

Step (d):

To a solution of the resulting product of step (**c**) (**S4a**, **S4b** or **S4c**) in dry THF (50 mL) was added NaH (0.88 g, 60% in mineral oil, 22.0 mmol) in portions at 0 ℃ under argon. The mixture was stirred at 0 ℃ for 30 minutes. Methyl iodide (598 μL, 9.60 mmol) was added dropwise over 10 minute at this temperature. The suspension was stirred at room temperature for 20 h, then quenched with saturated NH_4Cl (25 mL) and extracted with EtOAc (20 mL ×

3). The combined organic layers were washed with brine, dried over Na_2SO_4 and concentrated under reduced pressure. The crude product was purified by silica gel column chromatography (eluted with PE:EtOAc = 1:1, with 1 mol% Et_3N) to afford the pure chiral ligand (**L7**, **L8** or **L9**).

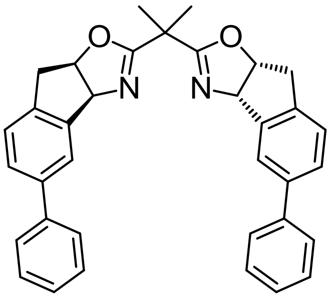

L7

According to the general procedure, chiral ligand **L7** was obtained as a white solid (367 mg, 0.72 mmol, overall yield of 4 steps: 18 %).

^{1}H NMR (600 MHz, $CDCl_3$) δ 7.76 (s, 2H), 7.62 (d, *J* = 9.4 Hz, 4H), 7.52 (d, *J* = 7.9 Hz, 2H), 7.43 (t, *J* = 7.8 Hz, 4H), 7.37 – 7.28 (m, 4H), 5.58 (d, *J* = 7.9 Hz, 2H), 5.36 – 5.29 (m, 2H), 3.36 (dd, *J* = 17.9, 7.1 Hz, 2H), 3.04 (d, *J* = 20.0 Hz, 2H), 1.45 (s, 6H).

^{13}C NMR (151 MHz, $CDCl_3$) δ 169.26, 142.55, 140.95, 140.57, 138.93, 128.77, 127.50, 127.22, 127.11, 125.40, 124.31, 83.54, 76.46, 39.43, 38.58, 23.95.

IR (film): ν (cm^{-1}) 3031, 2966, 2933, 1651, 1479, 1344, 1117, 994, 843, 759, 734, 696, 598, 507.

HRMS (ESI, m/z) calcd for $C_{35}H_{30}N_2NaO_2$ $(M+Na)^+$: 533.2199, found: 533.2204.

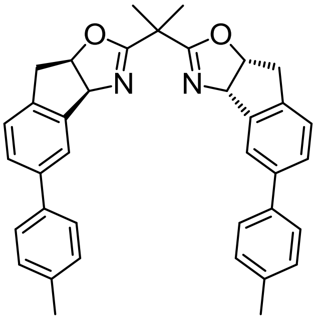

L8

According to the general procedure, chiral ligand **L8** was obtained as a white solid (397 mg, 0.74 mmol, overall yield of 4 steps: 19 %).

^{1}H NMR (600 MHz, $CDCl_3$) δ 7.73 (s, 2H), 7.57 – 7.46 (m, 6H), 7.27 (d, *J* = 7.9 Hz, 2H), 7.25 – 7.21 (m, 4H), 5.57 (d, *J* = 7.9 Hz, 2H), 5.37 – 5.27 (m, 2H), 3.40 – 3.26 (m, 2H), 3.02 (d, *J* = 17.7 Hz, 2H), 2.39 (s, 6H), 1.45 (s, 6H).

^{13}C NMR (151 MHz, $CDCl_3$) δ 169.18, 142.45, 140.45, 138.56, 138.02, 136.92, 129.45, 127.26, 126.89, 125.30, 124.03, 83.50, 76.43, 39.38, 38.53, 23.92, 21.06.

IR (film): ν (cm^{-1}) 2929, 1651, 1467, 1458, 1231, 1151, 1117, 995, 806, 738, 648, 582, 512.

HRMS (ESI, m/z) calcd for $C_{37}H_{34}N_2NaO_2$ $(M+Na)^+$: 561.2512, found: 561.2515.

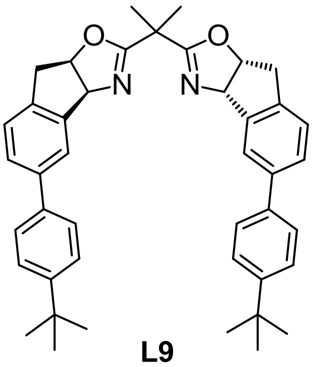

L9

According to the general procedure, chiral ligand **L9** was obtained as a yellow solid (479 mg, 0.77 mmol, overall yield of 4 steps: 19 %).

^{1}H NMR (600 MHz, $CDCl_3$) δ 7.75 (s, 2H), 7.58 – 7.55 (m, 4H), 7.52 (dd, *J* = 7.9, 1.8 Hz, 2H), 7.48 – 7.44 (m, 4H), 7.28 (d, *J* = 7.9 Hz, 2H), 5.57 (d, *J* = 7.9 Hz, 2H), 5.33 – 5.28 (m, 2H), 3.34 (dd, *J* = 17.8, 7.7 Hz, 2H), 3.01 (d, *J* = 17.9, 2H), 1.45 (s, 6H), 1.36 (s, 18H).

^{13}C NMR (151 MHz, $CDCl_3$) δ 169.23, 150.22, 142.49, 140.34, 138.62, 138.00, 127.30, 126.71, 125.73, 125.33, 124.13, 83.57, 76.46, 39.39, 38.57, 34.54, 31.38, 23.93.

IR (film): ν (cm^{-1}) 2961, 1651, 1466, 1393, 1362, 1267, 1142, 999, 815, 737, 612, 531, 509.

HRMS (ESI, m/z) calcd for $C_{43}H_{46}N_2NaO_2$ $(M+Na)^+$: 645.3451, found: 645.3467.

2.2. Preparation of α,β-Unsaturated *N*-Acyl Pyrazoles

α,β-unsaturated *N*-acyl pyrazoles **2a**, **2f−2i**, **2l−2o**,[1] and **2j**[2] were prepared by published procedures.

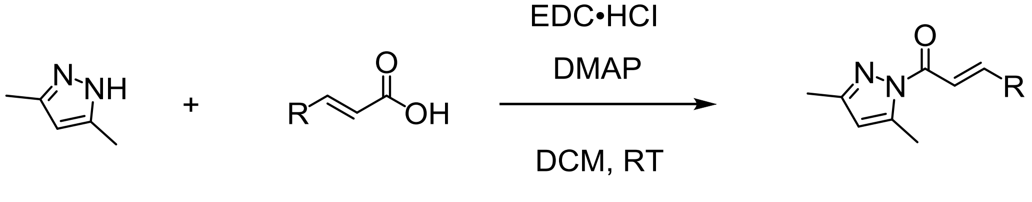

S5 **S6a**,**S6f–S6o** **2a, 2f–2o**

2a: R = Me
2f: R = Et
2g: R = *n*-Pr
2h: R = *n*-Pent
2i: R = *i*-Pr
2j: R = *i*-Bu

2k: R = Cy
2l: R = Ph
2m: R = Ph(4-Me)
2n: R = Ph(4-F)
2o: R = Ph(4-Cl)

General procedure. To a solution of α,β-unsaturated carboxylic acid (8.7 mmol) and 3,5-dimethyl-1H-pyrazole (**S5**, 923 mg, 9.6 mmol) in dichloromethane (20 mL) at 0 ℃, *N*-(3-dimethylaminopropyl)-N'-ethylcarbodiimide hydrochloride (EDC·HCl, 1.84 g, 9.6 mmol) and 4-dimethylaminopyridine (DMAP, 10.5 mg, 0.087 mmol) were added. The resulting mixture was stirred at 0 ℃ for 30 min, then slowly warmed up to room temperature and stirred for additional 12 h. The resulting mixture was concentrated to dryness. The residue was purified by column chromatography on silica gel (PE:EtOAc = 10:1) to afford the pure product.

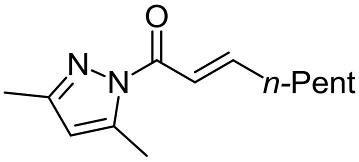

2h

According to the general procedure, **S6h** (1.24 g, 8.70 mmol) was converted to

α,β-unsaturated *N*-acyl pyrazole **2h** as a colorless oil (1.75 g, 7.95 mmol, yield: 91%).

^{1}H NMR (600 MHz, $CDCl_3$) δ 7.31 – 7.26 (m, 1H), 7.25 – 7.18 (m, 1H), 5.98 (s, 1H), 2.58 (s, 3H), 2.36 – 2.30 (m, 2H), 2.26 (s, 3H), 1.56 – 1.50 (m, 2H), 1.35 – 1.31 (m, 4H), 0.92 – 0.87 (m, 3H).

^{13}C NMR (151 MHz, $CDCl_3$) δ 165.27, 151.82, 151.71, 144.36, 120.98, 111.20, 32.72, 31.39, 27.82, 22.41, 14.61, 13.93, 13.77.

IR (film): ν (cm^{-1}) 3401, 3082, 2928, 2858, 1709, 1639, 1581, 1414, 1346, 1293, 1239, 1173, 984, 962, 863, 754, 623, 586.

HRMS (ESI, m/z) calcd for $C_{13}H_{20}N_2NaO$ $(M+Na)^+$: 243.1468, found: 243.1470.

2.3 Synthesis of the Racemic Products as References

2.3.1 Preparation of a Solution of the Racemic Catalyst [*rac*-L6-Ni] in DCE.

A solution of $Ni(ClO_4)_2·6H_2O$ (7.30 mg, 0.020 mmol) and *rac*-**L6** (8.60 mg, 0.024 mmol) in dichloroethane (DCE, 4.0 mL) was stirred at 75 °C for 5 h, then used freshly for the catalytic reactions.

2.3.2 Synthesis of the Racemic Reference Compounds *rac*-3a–3zo.

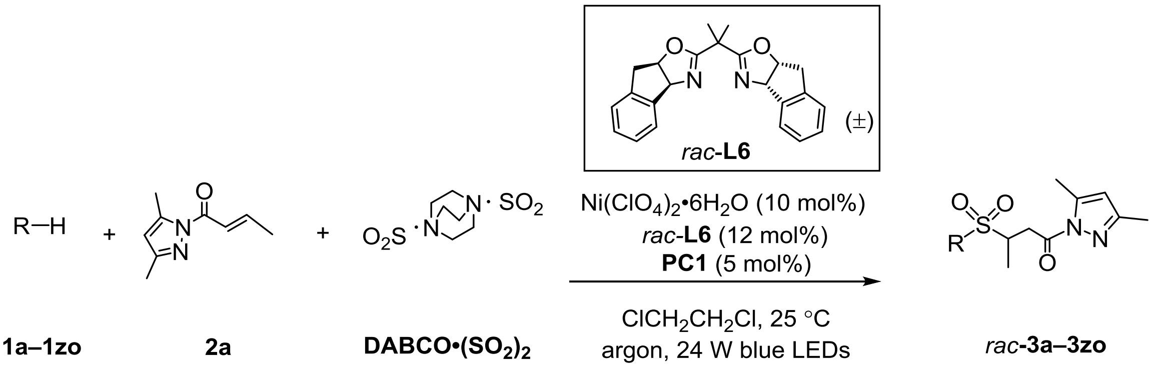

General procedure A. A dried 10 mL Schlenk tube was charged with **1a**–**1zo** (2.0 mmol), **2a** (0.20 mmol), DABCO·$(SO_2)_2$ (36.0 mg, 0.15 mmol), **PC1** (3.4 mg, 0.010 mmol), racemic catalyst **[*rac*-L6-Ni]** (4.0 mL, taken from the above-mentioned freshly prepared solution in DCE). The mixture was degassed *via* three freeze-pump-thaw cycles. The Schlenk tube was positioned approximately 5 cm away from a 24 W blue LEDs lamp (λ_{max} = 455 nm). After being stirred at 25 ℃ for the 10–24 h (monitored by TLC analysis), the reaction mixture was concentrated and purified by flash chromatography on silica gel (eluted with CH_2Cl_2 or PE:EtOAc = 4:1) to afford racemic product *rac*-**3a–3zo** as HPLC reference for the determination of enantiomeric excess.

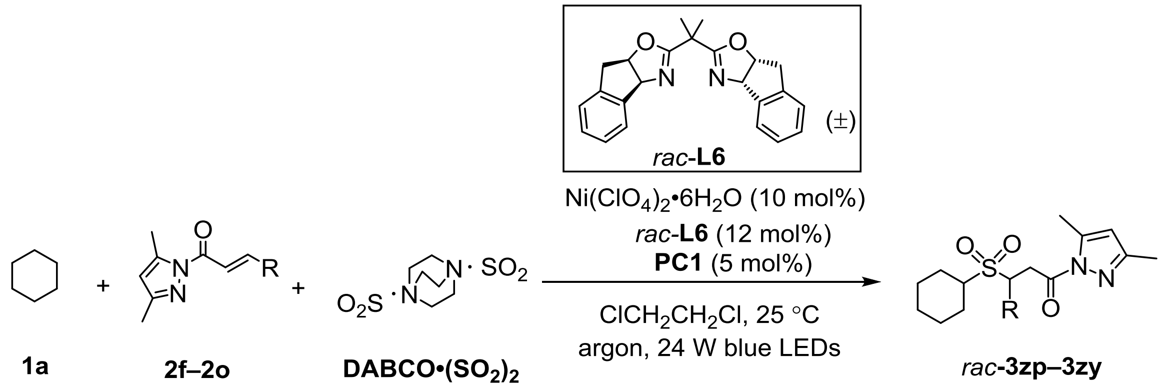

General procedure B. A dried 10 mL Schlenk tube was charged with **1a** (2.0 mmol), **2f**–**2o** (0.20 mmol), **PC1** (3.4 mg, 0.010 mmol), DABCO·$(SO_2)_2$ (36.0 mg, 0.15 mmol), racemic catalyst **[*rac*-L6-Ni]** (4.0 mL, taken from the above-mentioned freshly prepared solution in DCE). The mixture was degassed *via* three freeze-pump-thaw cycles. The Schlenk tube was positioned approximately 5 cm away from a 24 W blue LEDs lamp (λ_{max} = 455 nm). After being stirred at 25 ℃ for the 10–24 h (monitored by TLC analysis), the reaction mixture was concentrated and purified by flash chromatography on silica gel (eluted with CH_2Cl_2 or PE:EtOAc = 4:1) to afford racemic product *rac*-**3zp–3zy** as HPLC reference for the determination of enantiomeric excess.

3. Photocatalytic Asymmetric Three-Component Reactions

3.1 Reaction Optimization

3.1.1 Preparation of a Solution of the Non Racemic Metal Catalyst [L*-M] in DCE

According to the published procedure,[7,8] a solution of nickel salt (0.010 mmol) and a chiral ligand (**L1–L11**, 0.012 mmol) in 1,2-dimethoxyethane (DME, 4.0 mL) was stirred at 75 °C for 5 h, then the resulting solution was concentrated under reduced pressure to remove the solvent. The residue was redissolved in dichloroethane (DCE, 4.0 mL), which was used freshly as the metal catalyst for the photochemical reactions.

3.1.2 General Procedure for Reaction Optimization

A dried 10 mL Schlenk tube was charged with cyclohexane (**1a**, 1.0 mmol), **2a** (0.10 mmol), photocatalyst (**PC1−PC4**, 0.0050 mmol), SO_2 surrogate (0.075 mmol), metal catalyst **[L*-M]** (4.0 mL, taken from the above-mentioned freshly prepared solution in DCE). The mixture was degassed *via* three freeze-pump-thaw cycles. The Schlenk tube was positioned approximately 5 cm away from a 24 W blue LEDs lamp (λ_{max} = 455 nm). After being stirred at the indicated temperature for the indicated time, the reaction mixture was concentrated to dryness. The conversion was determined by ^{1}H NMR analysis of the crude product, and ee value was determined by chiral HPLC chromatography using a Daicel Chiralpak AD-H column.

Table 1. Optimization of reaction conditions[a].

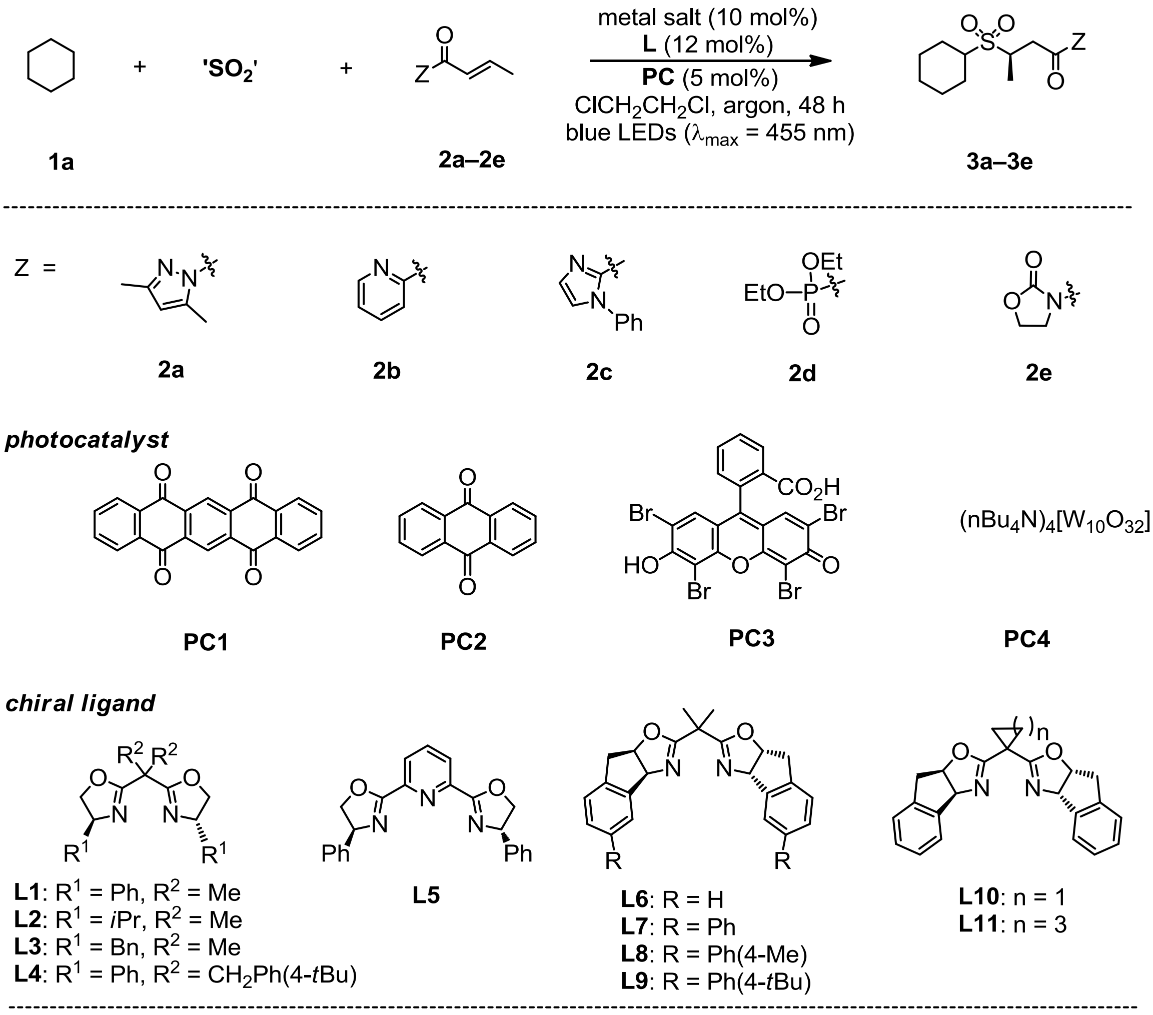

entry	metal salt	ligand	solvent	HAT PC	SO_2 source ('SO_2')	subs.	T (℃)	t (h)	prod.	conv. (%)[b]	ee (%)[c]
1	$Ni(ClO_4)_2·6H_2O$	**L1**	DCE	**PC1**	none	**2a**	20	48	**-**	0	n.a.
2	$Ni(ClO_4)_2·6H_2O$	**L1**	DCE	**PC1**	$Na_2S_2O_5$	**2a**	20	48	**3a**	0	n.a.
3	$Ni(ClO_4)_2·6H_2O$	**L1**	DCE	**PC1**	DABSO	**2a**	20	48	**3a**	58	3
4	$Ni(ClO_4)_2·6H_2O$	**L1**	DCE	**PC1**	DABSO	**2b**	20	48	**3b**	0	n.a.
5	$Ni(ClO_4)_2·6H_2O$	**L1**	DCE	**PC1**	DABSO	**2c**	20	48	**3c**	0	n.a.
6	$Ni(ClO_4)_2·6H_2O$	**L1**	DCE	**PC1**	DABSO	**2d**	20	48	**3d**	0	n.a.
7	$Ni(ClO_4)_2·6H_2O$	**L1**	DCE	**PC1**	DABSO	**2e**	20	48	**3e**	0	n.a.
8	$Ni(ClO_4)_2·6H_2O$	**L1**	DCE	**PC2**	DABSO	**2a**	20	48	**3a**	0	n.a.
9	$Ni(ClO_4)_2·6H_2O$	**L1**	DCE	**PC3**	DABSO	**2a**	20	48	**3a**	0	n.a.
10	$Ni(ClO_4)_2·6H_2O$	**L1**	DCE	**PC4**	DABSO	**2a**	20	48	**3a**	0	n.a.
11	$Cu(ClO_4)_2·6H_2O$	**L1**	DCE	**PC1**	DABSO	**2a**	20	48	**3a**	31	0

12	$Mg(ClO_4)_2·6H_2O$	**L1**	DCE	**PC1**	DABSO	**2a**	20	48	**3a**	trace	n.a.
13	$Zn(ClO_4)_2·6H_2O$	**L1**	DCE	**PC1**	DABSO	**2a**	20	48	**3a**	25	0
14	$Fe(ClO_4)_2$	**L1**	DCE	**PC1**	DABSO	**2a**	20	48	**3a**	17	n.d.
15	$Ni(ClO_4)_2·6H_2O$	**L1**	MeOH	**PC1**	DABSO	**2a**	20	48	**3a**	0	n.a.
16	$Ni(ClO_4)_2·6H_2O$	**L1**	MeCN	**PC1**	DABSO	**2a**	20	48	**3a**	trace	n.a.
17	$Ni(ClO_4)_2·6H_2O$	**L1**	CH_2Cl_2	**PC1**	DABSO	**2a**	20	48	**3a**	18	n.d.
18	$Ni(ClO_4)_2·6H_2O$	**L1**	$CHCl_3$	**PC1**	DABSO	**2a**	20	48	**3a**	23	2
19	$Ni(ClO_4)_2·6H_2O$	**L2**	DCE	**PC1**	DABSO	**2a**	20	48	**3a**	47	13
20	$Ni(ClO_4)_2·6H_2O$	**L3**	DCE	**PC1**	DABSO	**2e**	20	48	**3a**	53	11
21	$Ni(ClO_4)_2·6H_2O$	**L4**	DCE	**PC1**	DABSO	**2a**	20	48	**3a**	49	21
22	$Ni(ClO_4)_2·6H_2O$	**L5**	DCE	**PC1**	DABSO	**2a**	20	48	**3a**	37	11
23	$Ni(ClO_4)_2·6H_2O$	**L6**	DCE	**PC1**	DABSO	**2a**	20	48	**3a**	quant.	82
24	$Ni(ClO_4)_2·6H_2O$	**L7**	DCE	**PC1**	DABSO	**2a**	20	48	**3a**	quant.	86
25	$Ni(ClO_4)_2·6H_2O$	**L8**	DCE	**PC1**	DABSO	**2a**	20	48	**3a**	90	84
26	$Ni(ClO_4)_2·6H_2O$	**L9**	DCE	**PC1**	DABSO	**2a**	20	48	**3a**	85	86
27	$Ni(ClO_4)_2·6H_2O$	**L10**	DCE	**PC1**	DABSO	**2a**	20	48	**3a**	quant.	82
28	$Ni(ClO_4)_2·6H_2O$	**L11**	DCE	**PC1**	DABSO	**2a**	20	48	**3a**	quant.	79
29	$Ni(acac)_2$	**L7**	DCE	**PC1**	DABSO	**2a**	20	48	**3a**	43	17
30	$Ni(OAc)_2$	**L7**	DCE	**PC1**	DABSO	**2a**	20	48	**3a**	16	n.d.
31	$Ni(OTf)_2$	**L7**	DCE	**PC1**	DABSO	**2a**	20	48	**3a**	55	23
32	$Ni(COD)_2$	**L7**	DCE	**PC1**	DABSO	**2a**	20	48	**3a**	trace	n.a.
33	$Ni(ClO_4)_2·6H_2O$	**L7**	DCE	**PC1**	DABSO	**2a**	0	48	**3a**	77	95
34	$Ni(ClO_4)_2·6H_2O$	**L7**	DCE	**PC1**	DABSO	**2a**	-20	48	**3a**	47	95
35 [d]	$Ni(ClO_4)_2·6H_2O$	**L7**	DCE	**PC1**	DABSO	**2a**	0	48	**3a**	quant.	95
36	$Ni(ClO_4)_2·6H_2O$	**L7**	DCE	none	DABSO	**2a**	20	48	**3a**	0	n.a.
37	none	none	DCE	**PC1**	DABSO	**2a**	20	48	**3a**	0	n.a.
38 [e]	$Ni(ClO_4)_2·6H_2O$	**L7**	DCE	**PC1**	DABSO	**2a**	20	48	**3a**	0	n.a.
39 [f]	$Ni(ClO_4)_2·6H_2O$	**L7**	DCE	**PC1**	DABSO	**2a**	20	48	**3a**	0	n.a.
40 [g]	$Ni(ClO_4)_2·6H_2O$	**L7**	DCE	**PC1**	DABSO	**2a**	0	96	**3a**	52	93

[a]Reaction conditions: **1a** (1.0 mmol, 10 equiv.), **2a–2e** (0.10 mmol, 1.0 equiv.), SO_2 surrogate (0.075 mmol, 1.5 equiv.), metal salt **[L*-M]** (0.010 mmol, 10 mol%), ligand (0.012 mmol, 12 mol%), **PC1−PC4** (0.0050 mmol, 5 mol%), $ClCH_2CH_2Cl$ (4.0 mL), indicated temperature, indicated light source, under argon. [b]Conversion determined by ^{1}H-NMR. [c]Ee value determined by chiral HPLC. [d]Nickel salt (15 mol%), ligand (18 mol%). [e]In the dark. [f]In air. [g] Reaction performed in the presence of cyclohexane (1.0 equiv). DABSO = DABCO·$(SO_2)_2$, n.a. = not applicable. n.d. = not determined.

3.2 Substrate Scope of C(sp^3)-H precursors

3.2.1 Preparation of a Solution of the Non-Racemic Nickel Catalyst [L7-Ni] in DCE

According to the published procedure,[7,8] a solution of $Ni(ClO_4)_2 \cdot 6H_2O$ (11.0 mg, 0.030 mmol) and non-racemic ligand **L7** (18.4 mg, 0.036 mmol) in 1,2-dimethoxyethane (DME, 4.0 mL) was stirred at 75 °C for 5 h, then the resulting solution was concentrated under reduced pressure to remove the solvent. The residue was redissolved in dichloroethane (DCE, 4.0 mL), which was used freshly as the metal catalyst for the photochemical reactions.

3.2.2 General Procedure

A dried 25 mL Schlenk tube was charged with **1a**–**1zo** (2.0 or 4.0 mmol), **2a** (0.20 mmol), **PC1** (3.4 mg, 0.010 mmol), DABCO·$(SO_2)_2$ (36.0 mg, 0.15 mmol), chiral nickel catalyst **[L7-Ni]** (4.0 mL, taken from the above-mentioned freshly prepared solution in DCE), and DCE (4.0 mL). The mixture was degassed *via* three freeze-pump-thaw cycles. The Schlenk tube was positioned approximately 5 cm away from a 24 W blue LEDs lamp (λ_{max} = 455 nm). After being stirred at 0 ℃ or 20 ℃ for 24–75 h (monitored by TLC analysis), the reaction mixture was concentrated, then purified by flash chromatography on silica gel (eluted with CH_2Cl_2 or PE:EtOAc = 4:1) to afford non-racemic product **3a–3zo**.

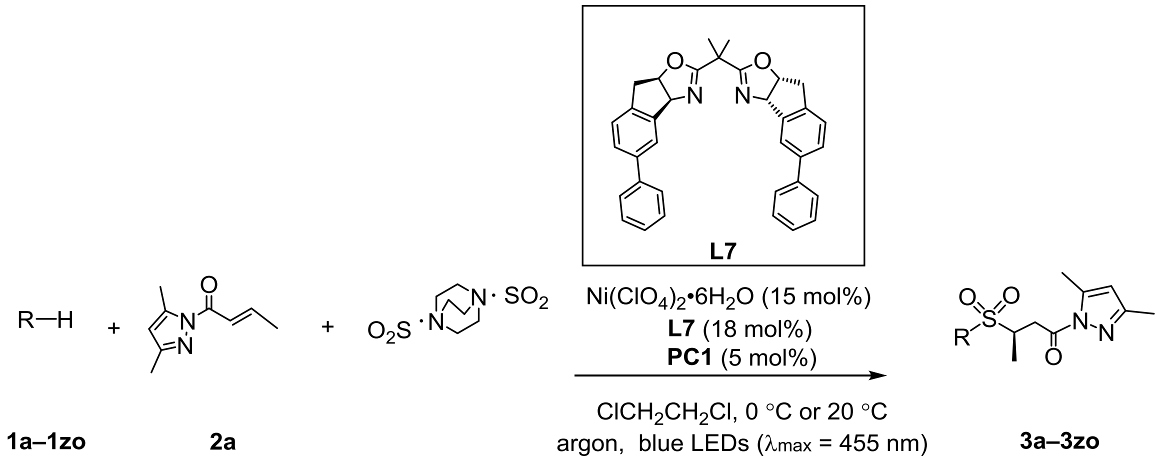

3.2.3 Experimental Details and Characterization Data

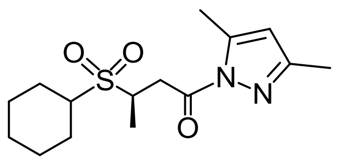

3a

A dried 25 mL Schlenk tube was charged with **1a** (168.3 mg, 2.0 mmol), **2a** (32.8 mg, 0.20 mmol), **PC1** (3.4 mg, 0.010 mmol), DABCO·$(SO_2)_2$ (36.0 mg, 0.15 mmol), chiral nickel catalyst **[L7-Ni]** (4.0 mL, taken from the above-mentioned freshly prepared solution in DCE), and DCE (4.0 mL). The mixture was degassed *via* three freeze-pump-thaw cycles. The Schlenk tube was positioned approximately 5 cm away from a 24 W blue LEDs lamp (λ_{max} = 455 nm). After being stirred at 0 ℃ for 48 h, the reaction mixture was concentrated, then purified by flash chromatography on silica gel (eluted with PE:EtOAc = 4:1) to afford product **3a** as a yellow solid (45.6 mg, 0.146 mmol, 73% yield). Enantiomeric excess was established by HPLC analysis using a Chiralpak AD-H column, ee = 95% (HPLC: AD-H, 254 nm, *n*-hexane:isopropanol = 90:10, flow rate: 1.0 mL/min, 30 ℃, t_r(minor) = 8.9 min, t_r(major) = 9.6 min). $[\alpha]_D^{23}$ = +4.7 °(*c* = 1.0, CH_2Cl_2). The configuration of **3a** was assigned *R* by analogy to (*R*)-**3b**.

^{1}H NMR (600 MHz, $CDCl_3$) δ 5.97 (s, 1H), 3.88 – 3.76 (m, 2H), 3.29 (dd, *J* = 16.9, 8.9 Hz, 1H), 3.09 – 3.01 (m, 1H), 2.53 (s, 3H), 2.23 – 2.20 (m, 4H), 2.18 – 2.13 (m, 1H), 1.97 – 1.92 (m, 2H), 1.75 – 1.70 (m, 1H), 1.67 – 1.55 (m, 2H), 1.45 (d, *J* = 6.8 Hz, 3H), 1.38 – 1.23 (m, 3H).

^{13}C NMR (151 MHz, $CDCl_3$) δ 170.35, 152.64, 144.14, 111.54, 58.23, 49.80, 35.64, 25.09, 24.85, 24.60, 14.35, 13.75, 13.59.

IR (film): ν (cm^{-1}) 2921, 2850, 1959, 1727, 1654, 1633, 1470, 1383, 1329, 1036, 963, 894, 804, 749, 696, 659, 587, 512.

HRMS (ESI, m/z) calcd for $C_{15}H_{24}N_2NaO_3S$ $(M+Na)^+$: 335.1400, found: 335.1405.

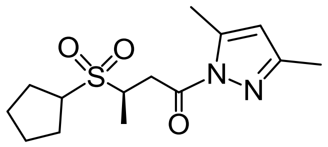

3b

A dried 25 mL Schlenk tube was charged with **1b** (140.3 mg, 2.0 mmol), **2a** (32.8 mg, 0.20 mmol), **PC1** (3.4 mg, 0.010 mmol), DABCO·$(SO_2)_2$ (36.0 mg, 0.15 mmol), chiral nickel catalyst **[L7-Ni]** (4.0 mL, taken from the above-mentioned freshly prepared solution in DCE), and DCE (4.0 mL). The mixture was degassed *via* three freeze-pump-thaw cycles. The Schlenk tube was positioned approximately 5 cm away from a 24 W blue LEDs lamp (λ_{max} = 455 nm). After being stirred at 0 ℃ for 48 h, the reaction mixture was concentrated, then purified by flash chromatography on silica gel (eluted with PE:EtOAc = 4:1) to afford product **3b** as a white solid (37.0 mg, 0.124 mmol, 62% yield). Enantiomeric excess was established by HPLC analysis using a Chiralpak AD-H column, ee = 94% (HPLC: AD-H, 254 nm, *n*-hexane:isopropanol = 90:10, flow rate: 1.0 mL/min, 30 ℃, t_r(minor) = 10.0 min, t_r(major) = 11.3 min). $[\alpha]_D^{23}$ = +7.5 °(*c* = 1.0, CH_2Cl_2). The configuration of **3b** was assigned as *R* by its crystal structure (CCDC no. 2028396).

^{1}H NMR (500 MHz, $CDCl_3$) δ 5.97 (s, 1H), 3.84 (dd, *J* = 17.3, 4.1 Hz, 1H), 3.80 – 3.71 (m, 1H), 3.60 – 3.49 (m, 1H), 3.31 (dd, *J* = 17.3, 8.9 Hz, 1H), 2.53 (s, 3H), 2.22 (s, 3H), 2.16 – 2.09 (m, 2H), 2.07 – 2.00 (m, 2H), 1.87 – 1.80 (m, 2H), 1.69 – 1.65 (m, 2H), 1.47 (d, *J* = 6.9 Hz, 3H).

^{13}C NMR (126 MHz, $CDCl_3$) δ 170.38, 152.65, 144.14, 111.55, 58.53, 52.11, 35.66, 26.96, 26.67, 26.01, 25.98, 14.34, 13.92, 13.75.

IR (film): ν (cm^{-1}) 2928, 2871, 1725, 1586, 1452, 1411, 1386, 1325, 1302, 1132, 962, 905, 869, 801, 758, 701, 656, 586, 507.

HRMS (ESI, m/z) calcd for $C_{14}H_{22}N_2NaO_3S$ $(M+Na)^+$: 321.1243, found: 321.1246.

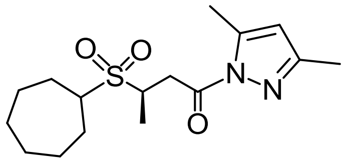

3c

A dried 25 mL Schlenk tube was charged with **1c** (196.4 mg, 2.0 mmol), **2a** (32.8 mg, 0.20 mmol), **PC1** (3.4 mg, 0.010 mmol), DABCO·$(SO_2)_2$ (36.0 mg, 0.15 mmol), chiral nickel catalyst **[L7-Ni]** (4.0 mL, taken from the above-mentioned freshly prepared solution in DCE), and DCE (4.0 mL). The mixture was degassed *via* three freeze-pump-thaw cycles. The Schlenk tube was positioned approximately 5 cm away from a 24 W blue LEDs lamp (λ_{max} = 455 nm). After being stirred at 0 ℃ for 48 h, the reaction mixture was concentrated, then purified by flash chromatography on silica gel (eluted with PE:EtOAc = 4:1) to afford product **3c** as a white solid (48.3 mg, 0.148 mmol, 74% yield). Enantiomeric excess was established by HPLC analysis using a Chiralpak AD-H column, ee = 94% (HPLC: AD-H, 254 nm, *n*-hexane:isopropanol = 90:10, flow rate: 1.0 mL/min, 30 ℃, t_r(minor) = 9.0 min, t_r(major) = 9.8 min). $[\alpha]_D^{23}$ = +21.3 °(*c* = 1.0, CH_2Cl_2). The configuration of **3c** was assigned *R* by analogy to (*R*)-**3b**.

^{1}H NMR (500 MHz, $CDCl_3$) δ 5.97 (s, 1H), 3.95 – 3.85 (m, 1H), 3.79 (dd, *J* = 17.2, 4.1 Hz, 1H), 3.28 (dd, *J* = 17.2, 9.0 Hz, 1H), 3.19 – 3.10 (m, 1H), 2.52 (s, 3H), 2.31 – 2.17 (m, 5H), 1.93 – 1.80 (m, 4H), 1.66 – 1.48 (m, 6H), 1.44 (d, *J* = 6.9 Hz, 3H).

^{13}C NMR (126 MHz, $CDCl_3$) δ 170.40, 152.62, 144.13, 111.54, 60.04, 50.07, 35.70, 28.17, 26.60, 26.21, 25.95, 25.92, 14.32, 13.81, 13.74.

IR (film): ν (cm^{-1}) 2961, 2927, 2854, 1725, 1586, 1459, 1410, 1384, 1324, 1304, 1261, 1108, 1019, 963, 870, 798, 756, 701, 658, 593, 514.

HRMS (ESI, m/z) calcd for $C_{16}H_{26}N_2NaO_3S$ $(M+Na)^+$: 349.1556, found: 349.1563.

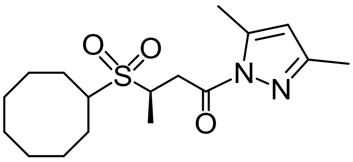

3d

A dried 25 mL Schlenk tube was charged with **1d** (224.4 mg, 2.0 mmol), **2a** (32.8 mg, 0.20 mmol), **PC1** (3.4 mg, 0.010 mmol), DABCO·$(SO_2)_2$ (36.0 mg, 0.15 mmol), chiral nickel catalyst **[L7-Ni]** (4.0 mL, taken from the above-mentioned freshly prepared solution in DCE), and DCE (4.0 mL). The mixture was degassed *via* three freeze-pump-thaw cycles. The Schlenk tube was positioned approximately 5 cm away from a 24 W blue LEDs lamp (λ_{max} = 455 nm). After being stirred at 0 ℃ for 48 h, the reaction mixture was concentrated, then purified by flash chromatography on silica gel (eluted with PE:EtOAc = 4:1) to afford product **3d** as a yellow oil (49.7 mg, 0.146 mmol, 73% yield). Enantiomeric excess was established by HPLC analysis using a Chiralpak AD-H column, ee = 95% (HPLC: AD-H, 254 nm, *n*-hexane:isopropanol = 90:10, flow rate: 1.0 mL/min, 30 ℃, t_r(minor) = 8.9 min, t_r(major) = 9.8 min). $[\alpha]_D^{23}$ = +5.2 ° (*c* = 1.0, CH_2Cl_2). The configuration of **3d** was assigned *R* by analogy to (*R*)-**3b**.

^{1}H NMR (500 MHz, $CDCl_3$) δ 5.97 (s, 1H), 3.97 – 3.85 (m, 1H), 3.81 (dd, *J* = 17.2, 4.3 Hz, 1H), 3.29 (dd, *J* = 17.2, 8.9 Hz, 1H), 3.25 – 3.14 (m, 1H), 2.53 (s, 3H), 2.27 – 2.14 (m, 5H), 1.92 – 1.71 (m, 5H), 1.69 – 1.52 (m, 7H), 1.45 (d, *J* = 6.9 Hz, 3H).

^{13}C NMR (126 MHz, $CDCl_3$) δ 170.44, 152.62, 144.13, 111.55, 59.09, 49.94, 35.68, 26.32, 26.14, 26.08, 25.53, 25.26, 25.13, 14.32, 13.98, 13.73.

IR (film): ν (cm^{-1}) 2961, 2926, 2854, 1727, 1586, 1449, 1412, 1386, 1325, 1292, 1261, 1096, 1025, 963, 872, 799, 706, 593, 511.

HRMS (ESI, m/z) calcd for $C_{17}H_{28}N_2NaO_3S$ $(M+Na)^+$: 363.1713, found: 363.1714.

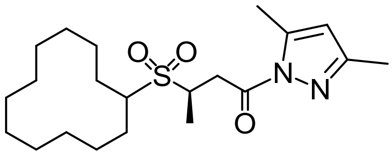

3e

A dried 25 mL Schlenk tube was charged with **1e** (336.6 mg, 2.0 mmol), **2a** (32.8 mg, 0.20 mmol), **PC1** (3.4 mg, 0.010 mmol), DABCO·$(SO_2)_2$ (36.0 mg, 0.15 mmol), chiral nickel catalyst **[L7-Ni]** (4.0 mL, taken from the above-mentioned freshly prepared solution in DCE), and DCE (4.0 mL). The mixture was degassed *via* three freeze-pump-thaw cycles. The Schlenk tube was positioned approximately 5 cm away from a 24 W blue LEDs lamp (λ_{max} = 455 nm). After being stirred at 0 ℃ for 48 h, the reaction mixture was concentrated, then purified by flash chromatography on silica gel (eluted with PE:EtOAc = 4:1) to afford product **3e** as a yellow oil (51.2 mg, 0.130 mmol, 65% yield). Enantiomeric excess was established by HPLC analysis using a Chiralpak AD-H column, ee = 91% (HPLC: AD-H, 254 nm, *n*-hexane:isopropanol = 90:10, flow rate: 1.0 mL/min, 30 ℃, t_r(minor) = 7.7 min, t_r(major) = 8.2 min). $[\alpha]_D^{23}$ = +9.7 °(*c* = 1.0, CH_2Cl_2). The configuration of **3e** was assigned *R* by analogy to (*R*)-**3b**.

^{1}H NMR (500 MHz, $CDCl_3$) δ 5.97 (s, 1H), 3.93 – 3.80 (m, 2H), 3.29 (dd, *J* = 17.0, 8.4 Hz, 1H), 3.22 – 3.14 (m, 1H), 2.53 (s, 3H), 2.22 (s, 3H), 2.03 – 1.86 (m, 2H), 1.86 – 1.77 (m, 2H), 1.69 – 1.49 (m, 4H), 1.45 (d, *J* = 6.9 Hz, 3H), 1.41 – 1.28 (m, 14H).

^{13}C NMR (126 MHz, $CDCl_3$) δ 170.51, 152.63, 144.12, 111.57, 56.32, 50.89, 35.61, 24.43, 24.40, 24.16, 23.71, 23.65, 23.46, 23.42, 23.41, 22.40, 22.32, 14.33, 14.06, 13.75.

IR (film): ν (cm^{-1}) 2927, 2855, 1727, 1586, 1471, 1446, 1410, 1385, 1325, 1302, 1261, 1127, 1020, 964, 866, 804, 717, 588, 510.

HRMS (ESI, m/z) calcd for $C_{21}H_{36}N_2NaO_3S$ $(M+Na)^+$: 419.2339, found: 419.2342.

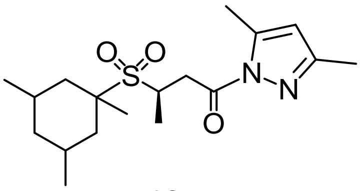

3f

A dried 25 mL Schlenk tube was charged with **1f** (252.4 mg, 2.0 mmol), **2a** (32.8 mg, 0.20 mmol), **PC1** (3.4 mg, 0.010 mmol), DABCO·$(SO_2)_2$ (36.0 mg, 0.15 mmol), chiral nickel catalyst **[L7-Ni]** (4.0 mL, taken from the above-mentioned freshly prepared solution in DCE), and DCE (4.0 mL). The mixture was degassed *via* three freeze-pump-thaw cycles. The Schlenk tube was positioned approximately 5 cm away from a 24 W blue LEDs lamp (λ_{max} = 455 nm). After being stirred at 0 ℃ for 60 h, the reaction mixture was concentrated, then purified by flash chromatography on silica gel (eluted with PE:EtOAc = 4:1) to afford product **3f** as a colorless oil (37.5 mg, 0.106 mmol, 53% yield). Enantiomeric excess was established by HPLC analysis using a Chiralpak AY-H column, ee = 78% (HPLC: AY-H, 240 nm, *n*-hexane:isopropanol = 90:10, flow rate: 1.0 mL/min, 30 ℃, t_r(minor) = 24.3 min, t_r(major) = 31.7 min). $[\alpha]_D^{23}$ = +7.4 ° (*c* = 1.0, CH_2Cl_2). The configuration of **3f** was assigned *R* by analogy to (*R*)-**3b**.

^{1}H NMR (500 MHz, $CDCl_3$) δ 5.97 (s, 1H), 4.14 – 4.06 (m, 1H), 3.96 (dd, *J* = 17.3, 5.1 Hz, 1H), 3.19 (dd, *J* = 17.3, 8.3 Hz, 1H), 2.53 (s, 3H), 2.22 (s, 3H), 1.84 – 1.75 (m, 2H), 1.68 – 1.59 (m, 5H), 1.50 (s, 3H), 1.47 (d, *J* = 7.0 Hz, 3H), 1.25 (s, 1H), 0.95 (dd, *J* = 6.4, 2.2 Hz, 6H).

^{13}C NMR (151 MHz, $CDCl_3$) δ 170.69, 152.47, 144.11, 111.45, 66.2, 48.70, 42.82, 37.70, 37.49, 37.39, 27.93, 27.90, 22.40, 18.62, 16.39, 14.38, 13.77.

IR (film): ν (cm^{-1}) 2951, 2928, 1726, 1585, 1457, 1410, 1384, 1323, 1285, 1142, 1107, 1024, 962, 875, 803, 747, 717, 592, 505.

HRMS (ESI, m/z) calcd for $C_{18}H_{30}N_2NaO_3S$ $(M+Na)^+$: 377.1869, found: 377.1871.

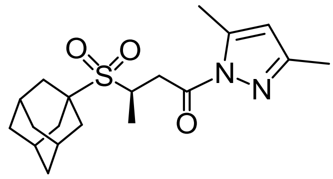

3g

A dried 25 mL Schlenk tube was charged with **1g** (272.5 mg, 2.0 mmol), **2a** (32.8 mg, 0.20 mmol), **PC1** (3.4 mg, 0.010 mmol), DABCO·$(SO_2)_2$ (36.0 mg, 0.15 mmol), chiral nickel catalyst **[L7-Ni]** (4.0 mL, taken from the above-mentioned freshly prepared solution in DCE), and DCE (4.0 mL). The mixture was degassed *via* three freeze-pump-thaw cycles. The Schlenk tube was positioned approximately 5 cm away from a 24 W blue LEDs lamp (λ_{max} = 455 nm). After being stirred at 0 ℃ for 40 h, the reaction mixture was concentrated, then purified by flash chromatography on silica gel (eluted with PE:EtOAc = 4:1) to afford product **3g** as a white solid (56.1 mg, 0.154 mmol, 77% yield). Enantiomeric excess was established by HPLC analysis using a Chiralpak AD-H column, ee = 90% (HPLC: AD-H, 220 nm, *n*-hexane:isopropanol = 95:5, flow rate: 1.0 mL/min, 30 ℃, t_r(minor) = 20.2 min, t_r(major) = 17.8 min). $[\alpha]_D^{23}$ = +11.7 ° (*c* = 1.0, CH_2Cl_2). The configuration of **3g** was assigned *R* by analogy to (*R*)-**3b**.

^{1}H NMR (500 MHz, $CDCl_3$) δ 5.96 (s, 1H), 4.13 – 4.03 (m, 1H), 3.95 (dd, *J* = 17.5, 5.4 Hz, 1H), 3.15 (dd, *J* = 17.4, 7.8 Hz, 1H), 2.53 (s, 3H), 2.22 (s, 3H), 2.20 – 2.16 (m, 3H), 2.17 – 2.06 (m, 6H), 1.77 – 1.72 (m, 6H), 1.45 (d, *J* = 7.0 Hz, 3H).

^{13}C NMR (151 MHz, $CDCl_3$) δ 170.69, 152.43, 144.08, 111.42, 62.67, 47.44, 37.27, 35.80, 35.12, 28.26, 16.26, 14.39, 13.76.

^{13}C NMR-dept 135 (151 MHz, $CDCl_3$) δ 111.46, 47.48, 37.31, 35.84, 35.16, 28.30, 16.30, 14.43, 13.80.

IR (film): ν (cm^{-1}) 2912, 2853, 1726, 1585, 1455, 1410, 1384, 1324, 1287, 1138, 1106, 1023, 963, 892, 801, 755, 706, 600, 504.

HRMS (ESI, m/z) calcd for $C_{19}H_{28}N_2NaO_3S$ $(M+Na)^+$: 387.1713, found: 387.1721.

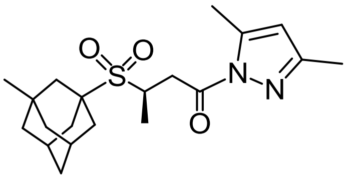

3h

A dried 25 mL Schlenk tube was charged with **1h** (300.5 mg, 2.0 mmol), **2a** (32.8 mg, 0.20 mmol), **PC1** (3.4 mg, 0.010 mmol), DABCO·$(SO_2)_2$ (36.0 mg, 0.15 mmol), chiral nickel catalyst **[L7-Ni]** (4.0 mL, taken from the above-mentioned freshly prepared solution in DCE), and DCE (4.0 mL). The mixture was degassed *via* three freeze-pump-thaw cycles. The Schlenk tube was positioned approximately 5 cm away from a 24 W blue LEDs lamp (λ_{max} = 455 nm). After being stirred at 0 ℃ for 40 h, the reaction mixture was concentrated, then purified by flash chromatography on silica gel (eluted with PE:EtOAc = 4:1) to afford product **3h** as a yellow solid (50.7 mg, 0.134 mmol, 67% yield). Enantiomeric excess was established by HPLC analysis using a Chiralpak AD-H column, ee = 90% (HPLC: AD-H, 220 nm, *n*-hexane:isopropanol = 98:2, flow rate: 1.0 mL/min, 30 ℃, t_r(minor) = 35.4 min, t_r(major) = 31.1 min). $[\alpha]_D^{23}$ = +12.4 ° (*c* = 1.0, CH_2Cl_2). The configuration of **3h** was assigned *R* by analogy to (*R*)-**3b**.

^{1}H NMR (600 MHz, $CDCl_3$) δ 5.97 (s, 1H), 4.10 – 4.04 (m, 1H), 3.94 (dd, *J* = 17.4, 5.3 Hz, 1H), 3.17 (dd, *J* = 17.4, 8.1 Hz, 1H), 2.53 (s, 3H), 2.26 – 2.19 (m, 5H), 2.10 – 2.04 (m, 1H), 2.03 (d, *J* = 3.0 Hz, 2H), 2.02 – 1.95 (m, 1H), 1.84 – 1.75 (m, 2H), 1.69 – 1.59 (m, 4H), 1.48 – 1.46 (m, 2H), 1.45 (d, *J* = 7.0 Hz, 3H), 0.90 (s, 3H).

^{13}C NMR (151 MHz, $CDCl_3$) δ 170.69, 152.46, 144.09, 111.44, 63.57, 47.69, 42.81, 41.42, 37.29, 35.03, 34.66, 34.51, 31.10, 30.53, 28.82, 28.80, 16.24, 14.39, 13.78.

^{13}C NMR-dept 135 (151 MHz, $CDCl_3$) δ 111.47, 47.73, 42.86, 41.46, 37.33, 35.07, 34.70, 34.54, 30.57, 28.86, 28.84, 16.27, 14.43, 13.82.

IR (film): ν (cm^{-1}) 2913, 1726, 1585, 1456, 1385, 1324, 1299, 1128, 962, 805, 757, 701, 600, 513.

HRMS (ESI, m/z) calcd for $C_{20}H_{30}N_2NaO_3S$ $(M+Na)^+$: 401.1869, found: 401.1874.

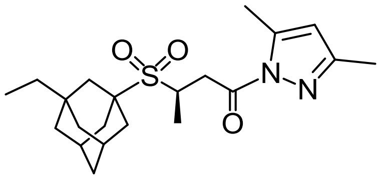

3i

A dried 25 mL Schlenk tube was charged with **1i** (328.6 mg, 2.0 mmol), **2a** (32.8 mg, 0.20 mmol), **PC1** (3.4 mg, 0.010 mmol), DABCO·$(SO_2)_2$ (36.0 mg, 0.15 mmol), chiral nickel catalyst **[L7-Ni]** (4.0 mL, taken from the above-mentioned freshly prepared solution in DCE), and DCE (4.0 mL). The mixture was degassed *via* three freeze-pump-thaw cycles. The Schlenk tube was positioned approximately 5 cm away from a 24 W blue LEDs lamp (λ_{max} = 455 nm). After being stirred at 0 ℃ for 30 h, the reaction mixture was concentrated, then purified by flash chromatography on silica gel (eluted with CH_2Cl_2) to afford product **3i** as a yellow oil (54.9 mg, 0.140 mmol, 70% yield). Enantiomeric excess was established by HPLC analysis using a Chiralpak OX-H column, ee = 87% (HPLC: OX-H, 240 nm, *n*-hexane:isopropanol = 95:5, flow rate: 1.0 mL/min, 30 ℃, t_r(minor) = 30.4 min, t_r(major) = 33.2 min). $[\alpha]_D^{23}$ = +8.8 ° (*c* = 1.0, CH_2Cl_2). The configuration of **3i** was assigned *R* by analogy to (*R*)-**3b**.

^{1}H NMR (600 MHz, $CDCl_3$) δ 5.96 (s, 1H), 4.07 (dd, *J* = 7.0, 1.9 Hz, 1H), 3.95 (dd, *J* = 17.4, 5.4 Hz, 1H), 3.15 (dd, *J* = 17.4, 7.9 Hz, 1H), 2.52 (s, 3H), 2.26 – 2.23 (m, 2H), 2.21 (s, 3H), 2.10 – 1.97 (m, 4H), 1.82 – 1.73 (m, 2H), 1.69 – 1.60 (m, 2H), 1.50 – 1.42 (m, 7H), 1.24 – 1.19 (m, 2H), 0.81 (t, *J* = 7.6 Hz, 3H).

^{13}C NMR (151 MHz, $CDCl_3$) δ 170.67, 152.43, 144.07, 111.42, 63.60, 47.66, 40.25, 40.21, 39.11, 37.27, 35.95, 35.42, 35.04, 34.88, 33.65, 28.70, 28.68, 16.25, 14.37, 13.76, 6.88.

^{13}C NMR-dept 135 (151 MHz, $CDCl_3$) δ 111.47, 47.70, 40.30, 40.26, 39.15, 37.32, 35.99, 35.47, 35.08, 34.93, 28.75, 28.72, 16.29, 14.42, 13.81, 6.93.

IR (film): ν (cm^{-1}) 2912, 2850, 1727, 1586, 1456, 1411, 1385, 1324, 1290, 1149, 1128, 1102, 1024, 963, 889, 803, 755, 699, 599, 516.

HRMS (ESI, m/z) calcd for $C_{21}H_{32}N_2NaO_3S$ $(M+Na)^+$: 415.2026, found: 415.2037.

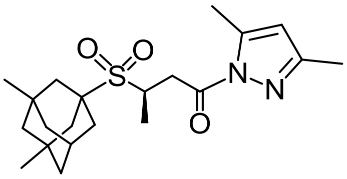

3j

A dried 25 mL Schlenk tube was charged with **1j** (328.6 mg, 2.0 mmol), **2a** (32.8 mg, 0.20 mmol), **PC1** (3.4 mg, 0.010 mmol), DABCO·$(SO_2)_2$ (36.0 mg, 0.15 mmol), chiral nickel catalyst **[L7-Ni]** (4.0 mL, taken from the above-mentioned freshly prepared solution in DCE), and DCE (4.0 mL). The mixture was degassed *via* three freeze-pump-thaw cycles. The Schlenk tube was positioned approximately 5 cm away from a 24 W blue LEDs lamp (λ_{max} = 455 nm). After being stirred at 0 ℃ for 35 h, the reaction mixture was concentrated, then purified by flash chromatography on silica gel (eluted with PE:EtOAc = 4:1) to afford product **3j** as a yellow oil (54.9 mg, 0.140 mmol, 70% yield). Enantiomeric excess was established by HPLC analysis using a Chiralpak AY-H column, ee = 84% (HPLC: AY-H, 240 nm, *n*-hexane:isopropanol = 70:30, flow rate: 1.0 mL/min, 30 ℃, t_r(minor) = 15.2 min, t_r(major) = 23.4 min). $[\alpha]_D^{23}$ = +8.8 ° (*c* = 1.0, CH_2Cl_2). The configuration of **3j** was assigned *R* by analogy to (*R*)-**3b**.

^{1}H NMR (600 MHz, $CDCl_3$) δ 5.96 (s, 1H), 4.10 – 3.99 (m, 1H), 3.92 (dd, *J* = 17.3, 5.1 Hz, 1H), 3.17 (dd, *J* = 17.3, 8.3 Hz, 1H), 2.53 (s, 3H), 2.29 – 2.24 (m, 1H), 2.22 (s, 3H), 2.00 – 1.90 (m, 2H), 1.79 – 1.66 (m, 5H), 1.44 (d, *J* = 7.0 Hz, 3H), 1.44 – 1.33 (m, 4H), 1.20 (s, 1H), 0.91 (s, 6H).

^{13}C NMR (151 MHz, $CDCl_3$) δ 170.67, 152.46, 144.09, 111.44, 64.43, 50.03, 47.91, 42.12, 42.11, 41.02, 40.85, 37.30, 34.04, 31.84, 31.81, 30.14, 29.36, 16.20, 14.37, 13.78.

^{13}C NMR-dept 135 (151 MHz, $CDCl_3$) δ 111.49, 50.07, 47.96, 42.16, 42.15, 41.06, 40.89, 37.34, 34.08, 30.19, 29.40, 16.24, 14.42, 13.83.

IR (film): ν (cm^{-1}) 2922, 2862, 1724, 1584, 1454, 1410, 1377, 1323, 1287, 1126, 1022, 961, 870, 803, 754, 703, 596, 505.

HRMS (ESI, m/z) calcd for $C_{21}H_{32}N_2NaO_3S$ $(M+Na)^+$: 415.2026, found: 415.2032.

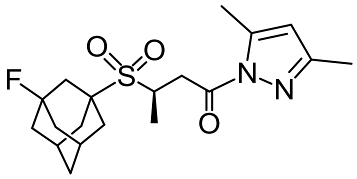

3k

A dried 25 mL Schlenk tube was charged with **1k** (308.4 mg, 2.0 mmol), **2a** (32.8 mg, 0.20 mmol), **PC1** (3.4 mg, 0.010 mmol), DABCO·$(SO_2)_2$ (36.0 mg, 0.15 mmol), chiral nickel catalyst **[L7-Ni]** (4.0 mL, taken from the above-mentioned freshly prepared solution in DCE), and DCE (4.0 mL). The mixture was degassed *via* three freeze-pump-thaw cycles. The Schlenk tube was positioned approximately 5 cm away from a 24 W blue LEDs lamp (λ_{max} = 455 nm). After being stirred at 0 ℃ for 35 h, the reaction mixture was concentrated, then purified by flash chromatography on silica gel (eluted with CH_2Cl_2) to afford product **3k** as a yellow oil (46.7 mg, 0.122 mmol, 61% yield). Enantiomeric excess was established by HPLC analysis using a Chiralpak AD-H column, ee = 87% (HPLC: AD-H, 240 nm, *n*-hexane:isopropanol = 95:5, flow rate: 1.0 mL/min, 30 ℃, t_r(minor) = 30.8 min, t_r(major) = 27.6 min). $[\alpha]_D^{23}$ = +9.2 ° (*c* = 1.0, CH_2Cl_2). The configuration of **3k** was assigned *R* by analogy to (*R*)-**3b**.

^{1}H NMR (600 MHz, $CDCl_3$) δ 5.97 (s, 1H), 4.14 – 4.04 (m, 1H), 3.96 (dd, *J* = 17.3, 5.5 Hz, 1H), 3.14 (dd, *J* = 17.3, 7.7 Hz, 1H), 2.54 – 2.48 (m, 5H), 2.29 – 2.19 (m, 5H), 2.10 – 1.99 (m, 4H), 1.93 – 1.89 (m, 4H), 1.64 – 1.62 (m, 2H), 1.46 (d, *J* = 7.0 Hz, 3H).

^{13}C NMR (151 MHz, $CDCl_3$) δ 170.40, 152.61, 144.17, 111.53, 92.49, 91.24, 65.15 (d, *J* = 9.8 Hz), 48.29, 41.35 (dd, *J* = 17.8, 2.4 Hz), 40.45 (d, *J* = 22.4 Hz), 37.29, 34.20, 34.04, 33.90, 30.74 (d, *J* = 9.4 Hz), 16.18, 14.38, 13.77.

^{13}C NMR-dept 135 (151 MHz, $CDCl_3$) δ 111.56, 48.32, 41.39 (dd, *J* = 17.9, 2.4 Hz), 40.48 (d, *J* = 22.4 Hz), 37.33, 34.24 (d, *J* = 1.9 Hz), 34.08 (d, *J* = 1.8 Hz), 33.94 (d, *J* = 1.8 Hz), 30.77 (dd, *J* = 9.9, 1.8 Hz), 30.74 (d, *J* = 1.8 Hz), 16.21, 14.42, 13.81.

IR (film): ν (cm^{-1}) 2922, 1727, 1587, 1457, 1384, 1300, 1229, 1138, 1080, 964, 753, 701, 501.

HRMS (ESI, m/z) calcd for $C_{19}H_{27}FN_2NaO_3S$ $(M+Na)^+$: 405.1619, found: 405.1626.

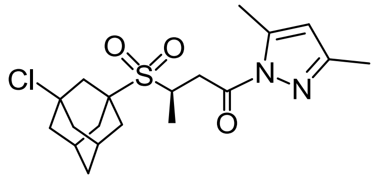

3l

A dried 25 mL Schlenk tube was charged with **1l** (341.4 mg, 2.0 mmol), **2a** (32.8 mg, 0.20 mmol), **PC1** (3.4 mg, 0.010 mmol), DABCO·$(SO_2)_2$ (36.0 mg, 0.15 mmol), chiral nickel catalyst **[L7-Ni]** (4.0 mL, taken from the above-mentioned freshly prepared solution in DCE), and DCE (4.0 mL). The mixture was degassed *via* three freeze-pump-thaw cycles. The Schlenk tube was positioned approximately 5 cm away from a 24 W blue LEDs lamp (λ_{max} = 455 nm). After being stirred at 0 ℃ for 40 h, the reaction mixture was concentrated, then purified by flash chromatography on silica gel (eluted with CH_2Cl_2) to afford product **3l** as a yellow solid (50.3 mg, 0.126 mmol, 63% yield). Enantiomeric excess was established by HPLC analysis using a Chiralpak OD-H column, ee = 88% (HPLC: OD-H, 220 nm, *n*-hexane:isopropanol = 97:3, flow rate: 1.0 mL/min, 30 ℃, t_r(minor) = 24.4 min, t_r(major) = 29.5 min). $[\alpha]_D^{23}$ = +13.4 ° (*c* = 1.0, CH_2Cl_2). The configuration of **3l** was assigned *R* by analogy to (*R*)-**3b**.

^{1}H NMR (600 MHz, $CDCl_3$) δ 5.97 (s, 1H), 4.14 – 4.02 (m, 1H), 3.95 (dd, *J* = 17.2, 5.4 Hz, 1H), 3.14 (dd, *J* = 17.2, 7.8 Hz, 1H), 2.53 (s, 3H), 2.51 – 2.44 (m, 2H), 2.43 – 2.39 (m, 2H), 2.23 (s, 3H), 2.14 – 2.09 (m, 5H), 2.09 – 2.02 (m, 3H), 1.71 – 1.66 (m, 2H), 1.47 (d, *J* = 7.0 Hz, 3H).

^{13}C NMR (151 MHz, $CDCl_3$) δ 170.38, 152.63, 144.17, 111.54, 65.92, 64.74, 48.32, 46.04, 46.02, 44.60, 37.32, 33.91, 33.76, 33.55, 30.99, 30.97, 16.15, 14.38, 13.80.

^{13}C NMR-dept 135 (151 MHz, $CDCl_3$) δ 111.57, 48.36, 46.08, 46.06, 44.64, 37.36, 33.95, 33.80, 33.58, 31.03, 31.01, 16.19, 14.42, 13.84.

IR (film): ν (cm^{-1}) 2926, 2861, 1725, 1588, 1457, 1383, 1323, 1298, 1136, 1027, 962, 802, 600, 507.

HRMS (ESI, m/z) calcd for $C_{19}H_{27}ClN_2NaO_3S$ $(M+Na)^+$: 421.1323, found: 421.1328.

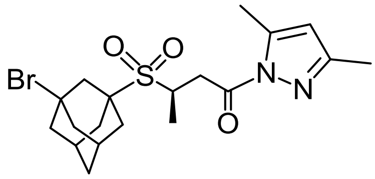

3m

A dried 25 mL Schlenk tube was charged with **1m** (430.2 mg, 2.0 mmol), **2a** (32.8 mg, 0.20 mmol), **PC1** (3.4 mg, 0.010 mmol), DABCO·$(SO_2)_2$ (36.0 mg, 0.15 mmol), chiral nickel catalyst **[L7-Ni]** (4.0 mL, taken from the above-mentioned freshly prepared solution in DCE), and DCE (4.0 mL). The mixture was degassed *via* three freeze-pump-thaw cycles. The Schlenk tube was positioned approximately 5 cm away from a 24 W blue LEDs lamp (λ_{max} = 455 nm). After being stirred at 0 ℃ for 40 h, the reaction mixture was concentrated, then purified by flash chromatography on silica gel (eluted with PE:EtOAc = 4:1) to afford product **3m** as a yellow solid (52.3 mg, 0.118 mmol, 59% yield). Enantiomeric excess was established by HPLC analysis using a Chiralpak AD-H column, ee = 89% (HPLC: AD-H, 220 nm, *n*-hexane:isopropanol = 90:10, flow rate: 0.8 mL/min, 30 ℃, t_r(minor) = 17.5 min, t_r(major) = 18.2 min). $[\alpha]_D^{23}$ = +8.6 °(*c* = 1.0, CH_2Cl_2). The configuration of **3m** was assigned *R* by analogy to (*R*)-**3b**.

^{1}H NMR (600 MHz, $CDCl_3$) δ 5.97 (s, 1H), 4.12 – 4.03 (m, 1H), 3.95 (dd, *J* = 17.2, 5.5 Hz, 1H), 3.14 (dd, *J* = 17.2, 7.9 Hz, 1H), 2.73 – 2.60 (m, 2H), 2.53 (s, 3H), 2.39 – 2.29 (m, 6H), 2.23 (s, 3H), 2.18 – 2.07 (m, 4H), 1.76 – 1.69 (m, 2H), 1.46 (d, *J* = 7.0 Hz, 3H).

^{13}C NMR (151 MHz, $CDCl_3$) δ 170.37, 152.63, 144.17, 111.54, 64.96, 61.24, 48.32, 47.45, 47.42, 45.82, 37.33, 33.89, 33.72, 33.48, 31.70, 31.68, 16.16, 14.38, 13.82.

^{13}C NMR-dept 135 (151 MHz, $CDCl_3$) δ 111.58, 48.35, 47.48, 47.45, 45.86, 37.36, 33.93, 33.75, 33.51, 31.74, 31.71, 16.19, 14.42, 13.85.

IR (film): ν (cm^{-1}) 2909, 1725, 1586, 1456, 1384, 1324, 1298, 1129, 1021, 963, 804, 754, 599, 510.

HRMS (ESI, m/z) calcd for $C_{19}H_{27}BrN_2NaO_3S$ $(M+Na)^+$: 465.0818, found: 465.0830.

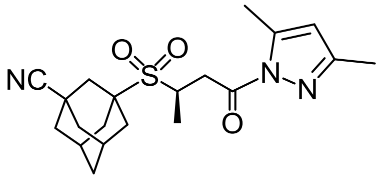

3n

A dried 25 mL Schlenk tube was charged with **1n** (322.5 mg, 2.0 mmol), **2a** (32.8 mg, 0.20 mmol), **PC1** (3.4 mg, 0.010 mmol), DABCO·$(SO_2)_2$ (36.0 mg, 0.15 mmol), chiral nickel catalyst **[L7-Ni]** (4.0 mL, taken from the above-mentioned freshly prepared solution in DCE), and DCE (4.0 mL). The mixture was degassed *via* three freeze-pump-thaw cycles. The Schlenk tube was positioned approximately 5 cm away from a 24 W blue LEDs lamp (λ_{max} = 455 nm). After being stirred at 0 ℃ for 35 h, the reaction mixture was concentrated, then purified by flash chromatography on silica gel (eluted with PE:EtOAc = 4:1) to afford product **3n** as a white solid (52.9 mg, 0.136 mmol, 68% yield). Enantiomeric excess was established by HPLC analysis using a Chiralpak AD-H column, ee = 82% (HPLC: AD-H, 220 nm, *n*-hexane:isopropanol = 80:20, flow rate: 1.0 mL/min, 30 ℃, t_r(minor) = 15.3 min, t_r(major) = 14.1 min). $[\alpha]_D^{23}$ = +8.4 ° (*c* = 1.0, CH_2Cl_2). The configuration of **3n** was assigned *R* by analogy to (*R*)-**3b**.

^{1}H NMR (600 MHz, $CDCl_3$) δ 5.98 (s, 1H), 4.13 – 4.03 (m, 1H), 3.96 (dd, *J* = 17.3, 5.7 Hz, 1H), 3.13 (dd, *J* = 17.3, 7.5 Hz, 1H), 2.53 (s, 3H), 2.45 – 2.32 (m, 4H), 2.23 (s, 3H), 2.16 – 2.01 (m, 8H), 1.76 – 1.73 (m, 2H), 1.47 (d, *J* = 7.0 Hz, 3H).

^{13}C NMR (151 MHz, $CDCl_3$) δ 170.25, 152.69, 144.22, 123.27, 111.59, 61.31, 48.22, 38.56, 38.53, 37.37, 37.27, 34.06, 33.80, 33.75, 30.99, 27.30, 16.20, 14.38, 13.80.

^{13}C NMR-dept 135 (151 MHz, $CDCl_3$) δ 111.63, 48.26, 38.61, 38.58, 37.42, 37.31, 34.11, 33.84, 33.79, 27.35, 16.24, 14.42, 13.84.

IR (film): ν (cm^{-1}) 2919, 1726, 1585, 1458, 1385, 1324, 1142, 962, 830, 738, 591, 517.

HRMS (ESI, m/z) calcd for $C_{20}H_{27}N_3NaO_3S$ $(M+Na)^+$: 412.1665, found: 412.1667.

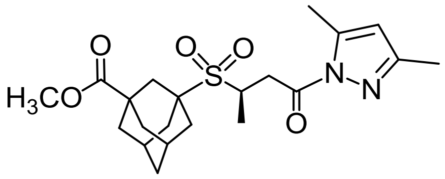

3o

A dried 25 mL Schlenk tube was charged with **1o** (388.6 mg, 2.0 mmol), **2a** (32.8 mg, 0.20 mmol), **PC1** (3.4 mg, 0.010 mmol), DABCO·$(SO_2)_2$ (36.0 mg, 0.15 mmol), chiral nickel catalyst **[L7-Ni]** (4.0 mL, taken from the above-mentioned freshly prepared solution in DCE), and DCE (4.0 mL). The mixture was degassed *via* three freeze-pump-thaw cycles. The Schlenk tube was positioned approximately 5 cm away from a 24 W blue LEDs lamp (λ_{max} = 455 nm). After being stirred at 0 ℃ for 35 h, the reaction mixture was concentrated, then purified by flash chromatography on silica gel (eluted with PE:EtOAc = 4:1) to afford product **3o** as a white solid (51.5 mg, 0.122 mmol, 61% yield). Enantiomeric excess was established by HPLC analysis using a Chiralpak OJ-H column, ee = 87% (HPLC: OJ-H, 240 nm, *n*-hexane:isopropanol = 90:10, flow rate: 1.0 mL/min, 30 ℃, t_r(minor) = 16.5 min, t_r(major) = 25.0 min). $[\alpha]_D^{23}$ = +11.3 ° (*c* = 1.0, CH_2Cl_2). The configuration of **3o** was assigned *R* by analogy to (*R*)-**3b**.

^1H NMR (600 MHz, $CDCl_3$) δ 5.96 (s, 1H), 4.12 – 4.02 (m, 1H), 3.94 (dd, *J* = 17.3, 5.3 Hz, 1H), 3.67 (s, 3H), 3.16 (dd, *J* = 17.4, 8.0 Hz, 1H), 2.52 (s, 3H), 2.34 – 2.30 (m, 2H), 2.28 – 2.19 (m, 5H), 2.13 – 2.03 (m, 4H), 1.93 – 1.84 (m, 4H), 1.72 – 1.69 (m, 2H), 1.45 (d, *J* = 7.0 Hz, 3H).

^{13}C NMR (151 MHz, $CDCl_3$) δ 176.24, 170.49, 152.51, 144.11, 111.46, 62.66, 51.95, 47.94, 41.50, 37.48, 37.42, 37.27, 36.29, 34.68, 34.47, 34.14, 28.07, 28.03, 16.19, 14.35, 13.74.

^{13}C NMR-dept 135 (151 MHz, $CDCl_3$) δ 111.51, 52.00, 47.99, 37.54, 37.47, 37.33, 36.35, 34.76, 34.52, 34.19, 28.13, 28.08, 16.25, 14.41, 13.81.

IR (film): ν (cm^{-1}) 2918, 1728, 1586, 1455, 1385, 1324, 1272, 1136, 1081, 964, 801, 751, 600, 510.

HRMS (ESI, m/z) calcd for $C_{21}H_{30}N_2NaO_5S$ $(M+Na)^+$: 445.1768, found: 445.1770.

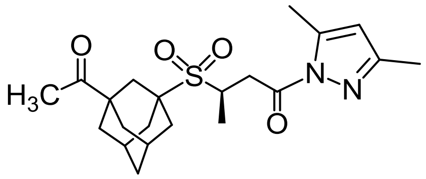

3p

A dried 25 mL Schlenk tube was charged with **1p** (356.5 mg, 2.0 mmol), **2a** (32.8 mg, 0.20 mmol), **PC1** (3.4 mg, 0.010 mmol), DABCO·$(SO_2)_2$ (36.0 mg, 0.15 mmol), chiral nickel catalyst **[L7-Ni]** (4.0 mL, taken from the above-mentioned freshly prepared solution in DCE), and DCE (4.0 mL). The mixture was degassed *via* three freeze-pump-thaw cycles. The Schlenk tube was positioned approximately 5 cm away from a 24 W blue LEDs lamp (λ_{max} = 455 nm). After being stirred at 0 ℃ for 35 h, the reaction mixture was concentrated, then purified by flash chromatography on silica gel (eluted with PE:EtOAc = 4:1) to afford product **3p** as a yellow oil (49.6 mg, 0.122 mmol, 61% yield). Enantiomeric excess was established by HPLC analysis using a Chiralpak OJ-H column, ee = 87% (HPLC: OJ-H, 240 nm, *n*-hexane:isopropanol = 90:10, flow rate: 1.0 mL/min, 30 ℃, t_r(minor) = 21.3 min, t_r(major) = 26.3 min). $[\alpha]_D^{23}$ = +10.7 ° (*c* = 1.0, CH_2Cl_2). The configuration of **3p** was assigned *R* by analogy to (*R*)-**3b**.

^1H NMR (600 MHz, $CDCl_3$) δ 5.96 (s, 1H), 4.13 – 4.03 (m, 1H), 3.95 (dd, *J* = 17.4, 5.6 Hz, 1H), 3.14 (dd, *J* = 17.4, 7.7 Hz, 1H), 2.51 (s, 3H), 2.39 – 2.32 (m, 2H), 2.21 (s, 3H), 2.19 – 2.14 (m, 1H), 2.15 – 2.10 (m, 5H), 2.11 – 2.07 (m, 2H), 2.06 – 2.02 (m, 1H), 1.85 – 1.79 (m, 3H), 1.76 – 1.71 (m, 3H), 1.45 (d, *J* = 7.0 Hz, 3H).

^{13}C NMR (151 MHz, $CDCl_3$) δ 211.42, 170.45, 152.53, 144.11, 111.48, 62.77, 47.91, 47.05, 37.25, 36.88, 35.59, 34.79, 34.45, 34.26, 28.08, 28.06, 24.48, 16.23, 14.35, 13.74.

^{13}C NMR-dept 135 (151 MHz, $CDCl_3$) δ 111.54, 47.96, 37.31, 36.95, 35.65, 34.85, 34.51, 34.32, 28.14, 28.12, 24.54, 16.29, 14.41, 13.81.

IR (film): ν (cm^{-1}) 2917, 2858, 1725, 1589, 1458, 1412, 1383, 1324, 1135, 1027, 963, 754, 663, 508.

HRMS (ESI, m/z) calcd for $C_{21}H_{30}N_2NaO_4S$ $(M+Na)^+$: 429.1818, found: 429.1821.

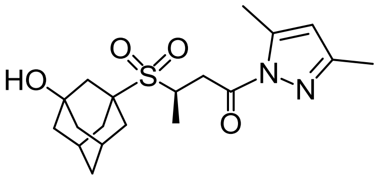

3q

A dried 25 mL Schlenk tube was charged with **1q** (304.5 mg, 2.0 mmol), **2a** (32.8 mg, 0.20 mmol), **PC1** (3.4 mg, 0.010 mmol), DABCO·$(SO_2)_2$ (36.0 mg, 0.15 mmol), chiral nickel catalyst **[L7-Ni]** (4.0 mL, taken from the above-mentioned freshly prepared solution in DCE), and DCE (4.0 mL). The mixture was degassed *via* three freeze-pump-thaw cycles. The Schlenk tube was positioned approximately 5 cm away from a 24 W blue LEDs lamp (λ_{max} = 455 nm). After being stirred at 0 ℃ for 30 h, the reaction mixture was concentrated, then purified by flash chromatography on silica gel (eluted with PE:EtOAc = 4:1) to afford product **3q** as a white solid (54.0 mg, 0.142 mmol, 71% yield). Enantiomeric excess was established by HPLC analysis using a Chiralpak AS-H column, ee = 91% (HPLC: AS-H, 240 nm, *n*-hexane:isopropanol = 80:20, flow rate: 1.0 mL/min, 30 ℃, t_r(minor) = 19.4 min, t_r(major) = 17.2 min). $[\alpha]_D^{23}$ = +5.4 °(*c* = 1.0, CH_2Cl_2). The configuration of **3q** was assigned *R* by analogy to (*R*)-**3b**.

^{1}H NMR (600 MHz, $CDCl_3$) δ 5.97 (s, 1H), 4.11 – 4.04 (m, 1H), 3.95 (dd, *J* = 17.4, 5.5 Hz, 1H), 3.15 (dd, *J* = 17.5, 7.8 Hz, 1H), 2.53 (s, 3H), 2.44 – 2.39 (m, 2H), 2.22 (s, 3H), 2.09 – 1.98 (m, 6H), 1.76 – 1.68 (m, 7H), 1.46 (d, *J* = 7.0 Hz, 3H).

^{13}C NMR (151 MHz, $CDCl_3$) δ 170.50, 152.55, 144.14, 111.50, 68.73, 64.81, 48.12, 43.76, 43.73, 42.77, 37.27, 34.38, 34.14, 34.02, 30.22, 16.23, 14.39, 13.78.

^{13}C NMR-dept 135 (151 MHz, $CDCl_3$) δ 111.53, 48.15, 43.80, 43.76, 42.80, 37.30, 34.41, 34.17, 34.05, 30.25, 16.26, 14.42, 13.82.

IR (film): ν (cm^{-1}) 2927, 2861, 1724, 1384, 1323, 1137, 987, 962, 800, 702, 652, 574, 510.

HRMS (ESI, m/z) calcd for $C_{19}H_{28}N_2NaO_4S$ $(M+Na)^+$: 403.1662, found: 403.1664.

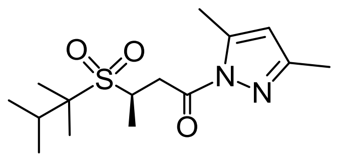

3r

A dried 25 mL Schlenk tube was charged with **1r** (344.7 mg, 4.0 mmol), **2a** (32.8 mg, 0.20 mmol), **PC1** (3.4 mg, 0.010 mmol), DABCO·$(SO_2)_2$ (36.0 mg, 0.15 mmol), chiral nickel catalyst **[L7-Ni]** (4.0 mL, taken from the above-mentioned freshly prepared solution in DCE), and DCE (4.0 mL). The mixture was degassed *via* three freeze-pump-thaw cycles. The Schlenk tube was positioned approximately 5 cm away from a 24 W blue LEDs lamp (λ_{max} = 455 nm). After being stirred at 0 ℃ for 48 h, the reaction mixture was concentrated, then purified by flash chromatography on silica gel (eluted with CH_2Cl_2) to afford product **3r** as a colorless solid (36.5 mg, 0.116 mmol, 58% yield). Enantiomeric excess was established by HPLC analysis using a Chiralpak AY-H column, ee = 75% (HPLC: AY-H, 240 nm, *n*-hexane:isopropanol = 90:10, flow rate: 1.5 mL/min, 30 ℃, t_r(minor) = 9.9 min, t_r(major) = 13.2 min). $[\alpha]_D^{23}$ = +6.8 ° (*c* = 1.0, CH_2Cl_2). The configuration of **3r** was assigned *R* by analogy to (*R*)-**3b**.

^{1}H NMR (600 MHz, $CDCl_3$) δ 5.96 (s, 1H), 4.21 – 4.07 (m, 1H), 3.98 (dd, *J* = 17.3, 5.3 Hz, 1H), 3.16 (dd, *J* = 17.3, 8.0 Hz, 1H), 2.53 (s, 3H), 2.42 – 2.30 (m, 1H), 2.22 (s, 3H), 1.47 (d, *J* = 7.0 Hz, 3H), 1.40 (d, *J* = 2.1 Hz, 6H), 1.08 (d, *J* = 6.8 Hz, 6H).

^{13}C NMR (151 MHz, $CDCl_3$) δ 170.69, 152.47, 144.10, 111.44, 67.98, 50.32, 37.38, 32.15, 19.38, 19.36, 18.75, 16.28, 14.37, 13.76.

IR (film): ν (cm^{-1}) 2925, 1723, 1585, 1465, 1383, 1323, 1288, 1104, 962, 870, 799, 751, 609, 505.

HRMS (ESI, m/z) calcd for $C_{15}H_{26}N_2NaO_3S$ $(M+Na)^+$: 337.1556, found: 337.1559.

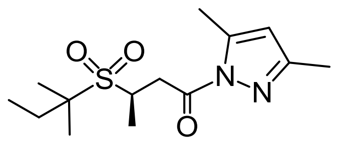

3s

A dried 25 mL Schlenk tube was charged with **1s** (288.6 mg, 4.0 mmol), **2a** (32.8 mg, 0.20 mmol), **PC1** (3.4 mg, 0.010 mmol), DABCO·$(SO_2)_2$ (36.0 mg, 0.15 mmol), chiral nickel catalyst **[L7-Ni]** (4.0 mL, taken from the above-mentioned freshly prepared solution in DCE), and DCE (4.0 mL). The mixture was degassed *via* three freeze-pump-thaw cycles. The Schlenk tube was positioned approximately 5 cm away from a 24 W blue LEDs lamp (λ_{max} = 455 nm). After being stirred at 0 ℃ for 72 h, the reaction mixture was concentrated, then purified by flash chromatography on silica gel (eluted with PE:Acetone = 4:1) to afford product **3s** as a white solid (30.6 mg, 0.102 mmol, 51% yield). Enantiomeric excess was established by HPLC analysis using a Chiralpak OX-H column, ee = 80% (HPLC: OX-H, 240 nm, *n*-hexane:isopropanol = 95:5, flow rate: 1.0 mL/min, 30 ℃, t_r(minor) = 21.7 min, t_r(major) = 23.5 min). $[\alpha]_D^{23}$ = +7.8 ° (*c* = 1.0, CH_2Cl_2). The configuration of **3s** was assigned *R* by analogy to (*R*)-**3b**.

^{1}H NMR (600 MHz, $CDCl_3$) δ 5.98 (s, 1H), 4.17 – 4.05 (m, 1H), 3.98 (dd, *J* = 17.4, 5.3 Hz, 1H), 3.17 (dd, *J* = 17.4, 7.9 Hz, 1H), 2.53 (s, 3H), 2.22 (s, 3H), 1.93 – 1.86 (m, 2H), 1.48 (d, *J* = 7.0 Hz, 3H), 1.41 (s, 6H), 1.01 (t, *J* = 7.5 Hz, 3H).

^{13}C NMR (151 MHz, $CDCl_3$) δ 170.65, 152.48, 144.11, 111.44, 64.73, 49.08, 37.44, 28.19, 20.34, 20.31, 16.38, 14.38, 13.76, 8.19.

IR (film): ν (cm^{-1}) 2933, 2850, 1724, 1586, 1416, 1384, 1323, 1288, 1105, 967, 804, 611, 585, 512.

HRMS (ESI, m/z) calcd for $C_{14}H_{24}N_2NaO_3S$ $(M+Na)^+$: 323.1400, found: 323.1400.

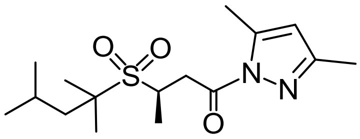

3t

A dried 25 mL Schlenk tube was charged with **1t** (400.8 mg, 4.0 mmol), **2a** (32.8 mg, 0.20 mmol), **PC1** (3.4 mg, 0.010 mmol), DABCO·$(SO_2)_2$ (36.0 mg, 0.15 mmol), chiral nickel catalyst **[L7-Ni]** (4.0 mL, taken from the above-mentioned freshly prepared solution in DCE), and DCE (4.0 mL). The mixture was degassed *via* three freeze-pump-thaw cycles. The Schlenk tube was positioned approximately 5 cm away from a 24 W blue LEDs lamp (λ_{max} = 455 nm). After being stirred at 0 ℃ for 75 h, the reaction mixture was concentrated, then purified by flash chromatography on silica gel (eluted with PE:Acetone = 4:1) to afford product **3t** as a colorless oil (37.4 mg, 0.114 mmol, 57% yield). Enantiomeric excess was established by HPLC analysis using a Chiralpak OX-H column, ee = 82% (HPLC: OX-H, 240 nm, *n*-hexane:isopropanol = 95:5, flow rate: 1.0 mL/min, 30 ℃, t_r(minor) = 18.1 min, t_r(major) = 20.1 min). $[\alpha]_D^{23}$ = +8.5 ° (*c* = 1.0, CH_2Cl_2). The configuration of **3t** was assigned *R* by analogy to (*R*)-**3b**.

^{1}H NMR (600 MHz, $CDCl_3$) δ 5.97 (s, 1H), 4.19 – 4.06 (m, 1H), 3.97 (dd, *J* = 17.3, 5.3 Hz, 1H), 3.17 (dd, *J* = 17.4, 8.0 Hz, 1H), 2.53 (s, 3H), 2.22 (s, 3H), 1.84 – 1.72 (m, 3H), 1.49 – 1.45 (m, 9H), 1.00 (d, *J* = 6.5 Hz, 6H).

^{13}C NMR (151 MHz, $CDCl_3$) δ 170.65, 152.50, 144.11, 111.46, 65.39, 48.96, 43.12, 37.52, 25.10, 24.34, 21.23, 21.15, 16.47, 14.38, 13.77.

IR (film): ν (cm^{-1}) 2932, 1725, 1585, 1470, 1386, 1324, 1290, 1121, 1026, 963, 798, 757, 700, 579.

HRMS (ESI, m/z) calcd for $C_{16}H_{28}N_2NaO_3S$ $(M+Na)^+$: 351.1713, found: 351.1712.

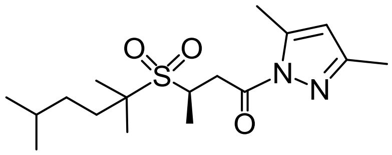

3u

A dried 25 mL Schlenk tube was charged with **1u** (456.8 mg, 4.0 mmol), **2a** (32.8 mg, 0.20 mmol), **PC1** (3.4 mg, 0.010 mmol), DABCO·$(SO_2)_2$ (36.0 mg, 0.15 mmol), chiral nickel catalyst **[L7-Ni]** (4.0 mL, taken from the above-mentioned freshly prepared solution in DCE), and DCE (4.0 mL). The mixture was degassed *via* three freeze-pump-thaw cycles. The Schlenk tube was positioned approximately 5 cm away from a 24 W blue LEDs lamp (λ_{max} = 455 nm). After being stirred at 0 ℃ for 75 h, the reaction mixture was concentrated, then purified by flash chromatography on silica gel (eluted with CH_2Cl_2) to afford product **3u** as a colorless oil (38.3 mg, 0.112 mmol, 56% yield). Enantiomeric excess was established by HPLC analysis using a Chiralpak OX-H column, ee = 77% (HPLC: OX-H, 240 nm, *n*-hexane:isopropanol = 95:5, flow rate: 1.0 mL/min, 30 ℃, t_r(minor) = 16.4 min, t_r(major) = 18.3 min). $[\alpha]_D^{23}$ = +5.1 ° (*c* = 1.0, CH_2Cl_2). The configuration of **3u** was assigned *R* by analogy to (*R*)-**3b**.

^{1}H NMR (600 MHz, $CDCl_3$) δ 5.96 (s, 1H), 4.16 – 4.08 (m, 1H), 3.99 (dd, *J* = 17.4, 5.4 Hz, 1H), 3.16 (dd, *J* = 17.4, 7.8 Hz, 1H), 2.52 (s, 3H), 2.21 (s, 3H), 1.85 – 1.75 (m, 2H), 1.57 – 1.50 (m, 1H), 1.48 (d, *J* = 7.0 Hz, 3H), 1.41 (s, 5H), 1.28 – 1.22 (m, 3H), 0.91 (d, *J* = 6.6 Hz, 6H).

^{13}C NMR (151 MHz, $CDCl_3$) δ 170.64, 152.47, 144.10, 111.44, 64.62, 49.04, 37.45, 33.06, 32.53, 28.52, 22.51, 22.49, 20.79, 16.43, 14.37, 13.75.

IR (film): ν (cm^{-1}) 2923, 2853, 1727, 1587, 1464, 1385, 1323, 1288, 1102, 963, 804, 752, 705, 602, 504.

HRMS (ESI, m/z) calcd for $C_{17}H_{30}N_2NaO_3S$ $(M+Na)^+$: 365.1869, found: 365.1870.

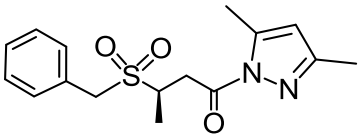

3v

A dried 25 mL Schlenk tube was charged with **1v** (184.3 mg, 2.0 mmol), **2a** (32.8 mg, 0.20 mmol), **PC1** (3.4 mg, 0.010 mmol), DABCO·$(SO_2)_2$ (36.0 mg, 0.15 mmol), chiral nickel catalyst **[L7-Ni]** (4.0 mL, taken from the above-mentioned freshly prepared solution in DCE), and DCE (4.0 mL). The mixture was degassed *via* three freeze-pump-thaw cycles. The Schlenk tube was positioned approximately 5 cm away from a 24 W blue LEDs lamp (λ_{max} = 455 nm). After being stirred at 0 ℃ for 20 h, the reaction mixture was concentrated, then purified by flash chromatography on silica gel (eluted with PE:EtOAc = 4:1) to afford product **3v** as a yellow oil (48.1 mg, 0.150 mmol, 75% yield). Enantiomeric excess was established by HPLC analysis using a Chiralpak AD-H column, ee = 92% (HPLC: AD-H, 220 nm, *n*-hexane:isopropanol = 90:10, flow rate: 1.0 mL/min, 30 ℃, t_r(minor) = 18.5 min, t_r(major) = 21.6 min). $[\alpha]_D^{23}$ = +48.2 ° (*c* = 1.0, CH_2Cl_2). The configuration of **3v** was assigned *R* by analogy to (*R*)-**3b**.

^1H NMR (600 MHz, $CDCl_3$) δ 7.50 – 7.35 (m, 5H), 5.97 (s, 1H), 4.31 (d, *J* = 2.6 Hz, 2H), 3.80 (dd, *J* = 17.4, 4.1 Hz, 1H), 3.73 – 3.64 (m, 1H), 3.35 (dd, *J* = 17.4, 9.1 Hz, 1H), 2.51 (s, 3H), 2.22 (s, 3H), 1.45 (d, *J* = 6.9 Hz, 3H).

^{13}C NMR (151 MHz, $CDCl_3$) δ 170.07, 152.69, 144.15, 130.77, 129.00, 127.43, 111.59, 57.03, 52.38, 35.59, 14.33, 14.12, 13.75.

IR (film): ν (cm^{-1}) 2930, 1725, 1586, 1456, 1410, 1387, 1326, 1310, 1264, 1121, 1030, 963, 872, 787, 756, 700, 596, 509.

HRMS (ESI, m/z) calcd for $C_{16}H_{20}N_2NaO_3S$ $(M+Na)^+$: 343.1087, found: 343.1091.

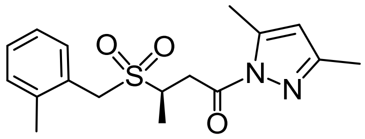

3w

A dried 25 mL Schlenk tube was charged with **1w** (212.3 mg, 2.0 mmol), **2a** (32.8 mg, 0.20 mmol), **PC1** (3.4 mg, 0.010 mmol), DABCO·$(SO_2)_2$ (36.0 mg, 0.15 mmol), chiral nickel catalyst **[L7-Ni]** (4.0 mL, taken from the above-mentioned freshly prepared solution in DCE), and DCE (4.0 mL). The mixture was degassed *via* three freeze-pump-thaw cycles. The Schlenk tube was positioned approximately 5 cm away from a 24 W blue LEDs lamp (λ_{max} = 455 nm). After being stirred at 0 ℃ for 30 h, the reaction mixture was concentrated, then purified by flash chromatography on silica gel (eluted with PE:EtOAc = 4:1) to afford product **3w** as a white solid (41.4 mg, 0.124 mmol, 62% yield). Enantiomeric excess was established by HPLC analysis using a Chiralpak AD-H column, ee = 90% (HPLC: AD-H, 220 nm, *n*-hexane:isopropanol = 90:10, flow rate: 1.0 mL/min, 30 ℃, t_r(minor) = 16.6 min, t_r(major) = 20.3 min). $[\alpha]_D^{23}$ = +33.2 ° (*c* = 1.0, CH_2Cl_2). The configuration of **3w** was assigned *R* by analogy to (*R*)-**3b**.

^{1}H NMR (500 MHz, $CDCl_3$) δ 7.42 – 7.34 (m, 1H), 7.32 – 7.13 (m, 3H), 5.98 (s, 1H), 4.37 (s, 2H), 3.93 – 3.79 (m, 2H), 3.46 – 3.31 (m, 1H), 2.53 (s, 3H), 2.46 (s, 3H), 2.22 (s, 3H), 1.51 (d, *J* = 6.9 Hz, 3H).

^{13}C NMR (126 MHz, $CDCl_3$) δ 170.13, 152.68, 144.16, 138.68, 131.82, 131.03, 129.08, 126.30, 125.57, 111.60, 54.03, 53.49, 35.89, 19.89, 14.31, 14.21, 13.73.

IR (film): ν (cm^{-1}) 2928, 1724, 1586, 1458, 1411, 1387, 1325, 1313, 1298, 1131, 1030, 963, 874, 806, 757, 711, 609, 511.

HRMS (ESI, m/z) calcd for $C_{17}H_{22}N_2NaO_3S$ $(M+Na)^+$: 357.1243, found: 357.1246.

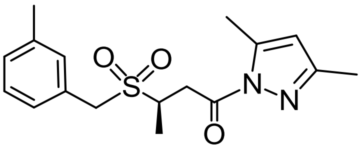

3x

A dried 25 mL Schlenk tube was charged with **1x** (212.3 mg, 2.0 mmol), **2a** (32.8 mg, 0.20 mmol), **PC1** (3.4 mg, 0.010 mmol), DABCO·$(SO_2)_2$ (36.0 mg, 0.15 mmol), chiral nickel catalyst **[L7-Ni]** (4.0 mL, taken from the above-mentioned freshly prepared solution in DCE), and DCE (4.0 mL). The mixture was degassed *via* three freeze-pump-thaw cycles. The Schlenk tube was positioned approximately 5 cm away from a 24 W blue LEDs lamp (λ_{max} = 455 nm). After being stirred at 0 ℃ for 35 h, the reaction mixture was concentrated, then purified by flash chromatography on silica gel (eluted with PE:EtOAc = 4:1) to afford product **3x** as a yellow oil (43.5 mg, 0.130 mmol, 65% yield). Enantiomeric excess was established by HPLC analysis using a Chiralpak OD-H column, ee = 92% (HPLC: OD-H, 220 nm, *n*-hexane:isopropanol = 90:10, flow rate: 1.0 mL/min, 30 ℃, t_r(minor) = 16.4 min, t_r(major) = 19.8 min). $[\alpha]_D^{23}$ = +24.3 ° (*c* = 1.0, CH_2Cl_2). The configuration of **3x** was assigned *R* by analogy to (*R*)-**3b**.

^{1}H NMR (500 MHz, $CDCl_3$) δ 7.34 – 7.17 (m, 4H), 5.97 (s, 1H), 4.27 (d, *J* = 1.9 Hz, 2H), 3.80 (dd, *J* = 17.5, 4.0 Hz, 1H), 3.76 – 3.63 (m, 1H), 3.35 (dd, *J* = 17.5, 9.1 Hz, 1H), 2.52 (s, 3H), 2.36 (s, 3H), 2.22 (s, 3H), 1.45 (d, *J* = 6.9 Hz, 3H).

^{13}C NMR (126 MHz, $CDCl_3$) δ 170.11, 152.64, 144.13, 138.77, 131.43, 129.78, 128.84, 127.76, 127.26, 111.57, 57.04, 52.41, 35.60, 21.31, 14.32, 14.13, 13.74.

IR (film): ν (cm^{-1}) 2963, 2928, 1724, 1586, 1455, 1385, 1309, 1262, 1112, 1028, 987, 963, 870, 798, 759, 699, 611, 515.

HRMS (ESI, m/z) calcd for $C_{17}H_{22}N_2NaO_3S$ $(M+Na)^+$: 357.1243, found: 357.1245.

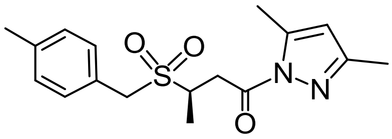

3y

A dried 25 mL Schlenk tube was charged with **1y** (212.3 mg, 2.0 mmol), **2a** (32.8 mg, 0.20 mmol), **PC1** (3.4 mg, 0.010 mmol), DABCO·$(SO_2)_2$ (36.0 mg, 0.15 mmol), chiral nickel catalyst **[L7-Ni]** (4.0 mL, taken from the above-mentioned freshly prepared solution in DCE), and DCE (4.0 mL). The mixture was degassed *via* three freeze-pump-thaw cycles. The Schlenk tube was positioned approximately 5 cm away from a 24 W blue LEDs lamp (λ_{max} = 455 nm). After being stirred at 0 ℃ for 35 h, the reaction mixture was concentrated, then purified by flash chromatography on silica gel (eluted with PE:EtOAc = 4:1) to afford product **3y** as a white oil (38.8 mg, 0.116 mmol, 58% yield). Enantiomeric excess was established by HPLC analysis using a Chiralpak OD-H column, ee = 94% (HPLC: OD-H, 220 nm, *n*-hexane:isopropanol = 90:10, flow rate: 1.0 mL/min, 30 ℃, t_r(minor) = 17.0 min, t_r(major) = 19.7 min). $[\alpha]_D^{23}$ = +17.6 ° (*c* = 1.0, CH_2Cl_2). The configuration of **3y** was assigned *R* by analogy to (*R*)-**3b**.

^{1}H NMR (600 MHz, $CDCl_3$) δ 7.38 – 7.29 (m, 2H), 7.24 – 7.16 (m, 2H), 5.97 (s, 1H), 4.27 (d, *J* = 1.9 Hz, 2H), 3.78 (dd, *J* = 17.5, 4.0 Hz, 1H), 3.72 – 3.62 (m, 1H), 3.34 (dd, *J* = 17.4, 9.2 Hz, 1H), 2.51 (s, 3H), 2.36 (s, 3H), 2.22 (s, 3H), 1.44 (d, *J* = 6.9 Hz, 3H).

^{13}C NMR (151 MHz, $CDCl_3$) δ 170.10, 152.63, 144.11, 138.95, 130.57, 129.71, 124.29, 111.55, 56.73, 52.12, 35.54, 21.19, 14.32, 14.07, 13.73.

IR (film): ν (cm^{-1}) 2927, 1725, 1586, 1456, 1410, 1386, 1326, 1311, 1123, 1026, 963, 877, 822, 767, 705, 587, 514.

HRMS (ESI, m/z) calcd for $C_{17}H_{22}N_2NaO_3S$ $(M+Na)^+$: 357.1243, found: 357.1245.

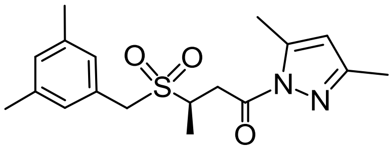

3z

A dried 25 mL Schlenk tube was charged with **1z** (240.4 mg, 2.0 mmol), **2a** (32.8 mg, 0.20 mmol), **PC1** (3.4 mg, 0.010 mmol), DABCO·$(SO_2)_2$ (36.0 mg, 0.15 mmol), chiral nickel catalyst **[L7-Ni]** (4.0 mL, taken from the above-mentioned freshly prepared solution in DCE), and DCE (4.0 mL). The mixture was degassed *via* three freeze-pump-thaw cycles. The Schlenk tube was positioned approximately 5 cm away from a 24 W blue LEDs lamp (λ_{max} = 455 nm). After being stirred at 0 ℃ for 30 h, the reaction mixture was concentrated, then purified by flash chromatography on silica gel (eluted with PE:EtOAc = 4:1) to afford product **3z** as a white solid (50.1 mg, 0.144 mmol, 72% yield). Enantiomeric excess was established by HPLC analysis using a Chiralpak OD-H column, ee = 90% (HPLC: OD-H, 220 nm, *n*-hexane:isopropanol = 90:10, flow rate: 1.0 mL/min, 30 ℃, t_r(minor) = 12.8 min, t_r(major) = 16.3 min). $[\alpha]_D^{23}$ = +39.7 ° (*c* = 1.0, CH_2Cl_2). The configuration of **3z** was assigned *R* by analogy to (*R*)-**3b**.

^{1}H NMR (600 MHz, $CDCl_3$) δ 7.02 (d, *J* = 19.6 Hz, 3H), 5.97 (s, 1H), 4.23 (d, *J* = 2.4 Hz, 2H), 3.79 (dd, *J* = 17.5, 4.0 Hz, 1H), 3.74 – 3.64 (m, 1H), 3.35 (dd, *J* = 17.5, 9.1 Hz, 1H), 2.52 (s, 3H), 2.31 (s, 6H), 2.22 (s, 3H), 1.46 (d, *J* = 6.9 Hz, 3H).

^{13}C NMR (126 MHz, $CDCl_3$) δ 170.14, 152.61, 144.11, 138.57, 130.69, 128.47, 127.09, 111.55, 57.02, 52.42, 35.60, 21.18, 14.32, 14.15, 13.74.

IR (film): ν (cm^{-1}) 2926, 1725, 1586, 1459, 1411, 1386, 1325, 1313, 1120, 1029, 963, 873, 801, 758, 702, 572, 516.

HRMS (ESI, m/z) calcd for $C_{18}H_{24}N_2NaO_3S$ $(M+Na)^+$: 371.1400, found: 371.1402.

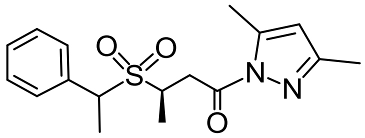

3za

A dried 25 mL Schlenk tube was charged with **1za** (212.3 mg, 2.0 mmol), **2a** (32.8 mg, 0.20 mmol), **PC1** (3.4 mg, 0.010 mmol), DABCO·$(SO_2)_2$ (36.0 mg, 0.15 mmol), chiral nickel catalyst **[L7-Ni]** (4.0 mL, taken from the above-mentioned freshly prepared solution in DCE), and DCE (4.0 mL). The mixture was degassed *via* three freeze-pump-thaw cycles. The Schlenk tube was positioned approximately 5 cm away from a 24 W blue LEDs lamp (λ_{max} = 455 nm). After being stirred at 0 ℃ for 30 h, the reaction mixture was concentrated, the diastereomeric ratio was determined as 1:1 dr by ^{1}H NMR analysis of the crude product. The residue was then purified by flash chromatography on silica gel (eluted with CH_2Cl_2) to afford product **3za** as a diastereomeric mixture (46.8 mg, 0.140 mmol, 70% yield). Enantiomeric excess was established by HPLC analysis using a Chiralpak AD-H column, ee = 90%/87% (HPLC: AD-H, 220 nm, *n*-hexane:isopropanol = 90:10, flow rate: 1.0 mL/min, 30 ℃, isomer I, t_r(minor) = 13.7 min, t_r(major) = 16.0 min, isomer II, t_r(minor) = 10.7 min, t_r(major) = 11.3 min). $[\alpha]_D^{23}$ = +8.1 ° (*c* = 1.0, CH_2Cl_2). The configuration of **3za** was assigned *R* by analogy to (*R*)-**3b**.

^{1}H NMR (600 MHz, $CDCl_3$) δ 7.51 – 7.45 (m, 2H), 7.41 – 7.34 (m, 3H), 5.97 (s, 0.5H), 5.93 (s, 0.5H), 4.44 – 4.36 (m, 1H), 3.79 – 3.74 (m, 0.5H), 3.72 – 3.63 (m, 1H), 3.56 – 3.50 (m, 0.5H), 3.29 (dd, *J* = 17.5, 9.6 Hz, 0.5H), 3.21 (dd, *J* = 17.6, 8.6 Hz, 0.5H), 2.51 (s, 1.5H), 2.47 (s, 1.5H), 2.22 (s, 1.5H), 2.19 (s, 1.5H), 1.80 (d, *J* = 7.1 Hz, 3H), 1.38 (d, *J* = 6.8 Hz, 1.5H), 1.26 (d, *J* = 7.0 Hz, 1.5H).

^{13}C NMR (151 MHz, $CDCl_3$) δ 170.21, 170.18, 152.56, 152.49, 144.10, 143.98, 134.36, 134.21, 129.18, 129.06, 128.97, 128.86, 111.52, 111.42, 61.62, 60.75, 51.14, 50.75, 36.31, 34.78, 14.52, 14.35, 14.28, 14.21, 13.75, 13.72, 13.70.

IR (film): ν (cm^{-1}) 2984, 2931, 1725, 1585, 1455, 1410, 1385, 1325, 1308, 1134, 1086, 963,

874, 757, 701, 610, 526.

HRMS (ESI, m/z) calcd for $C_{17}H_{22}N_2NaO_3S$ $(M+Na)^+$: 357.1243, found: 357.1244.

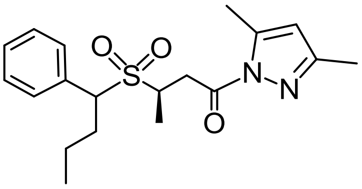

3zb

A dried 25 mL Schlenk tube was charged with **1zb** (268.4 mg, 2.0 mmol), **2a** (32.8 mg, 0.20 mmol), **PC1** (3.4 mg, 0.010 mmol), DABCO·$(SO_2)_2$ (36.0 mg, 0.15 mmol), chiral nickel catalyst **[L7-Ni]** (4.0 mL, taken from the above-mentioned freshly prepared solution in DCE), and DCE (4.0 mL). The mixture was degassed *via* three freeze-pump-thaw cycles. The Schlenk tube was positioned approximately 5 cm away from a 24 W blue LEDs lamp (λ_{max} = 455 nm). After being stirred at 0 ℃ for 30 h, the reaction mixture was concentrated, the diastereomeric ratio was determined as 1:1 dr by ^{1}H NMR analysis of the crude product. The residue was then purified by flash chromatography on silica gel (eluted with CH_2Cl_2) to afford product **3zb** as a diastereomeric mixture (51.5 mg, 0.142 mmol, 71% yield). Enantiomeric excess was established by HPLC analysis using a Chiralpak OJ-H column, ee = 91%/91% (HPLC: OJ-H, 220 nm, *n*-hexane:isopropanol = 95:5, flow rate: 0.8 mL/min, 30 ℃, isomer I, t_r(minor) = 15.0 min, t_r(major) = 40.0 min, isomer II, t_r(minor) = 33.2 min, t_r(major) = 28.9 min). $[\alpha]_D^{23}$ = +47.7 ° (*c* = 1.0, CH_2Cl_2). The configuration of **3zb** was assigned *R* by analogy to (*R*)-**3b**.

^{1}H NMR (500 MHz, $CDCl_3$) δ 7.48 – 7.42 (m, 2H), 7.41 – 7.33 (m, 3H), 5.96 (s, 0.5H), 5.92 (s, 0.5H), 4.26 – 4.16 (m, 1H), 3.75 – 3.65 (m, 1H), 3.60 – 3.53 (m, 0.5H), 3.47 – 3.40 (m, 0.5H), 3.27 (dd, *J* = 17.5, 9.6 Hz, 0.5H), 3.19 (dd, *J* = 17.5, 8.5 Hz, 0.5H), 2.48 (dd, *J* = 23.4, 1.1 Hz, 3H), 2.40 – 2.31 (m, 1H), 2.20 (d, *J* = 18.5 Hz, 3H), 2.15 – 2.07 (m, 1H), 1.35 (d, *J* = 6.7 Hz, 2H), 1.23 (d, *J* = 7.0 Hz, 3H), 0.90 (t, *J* = 7.4 Hz, 3H).

^{13}C NMR (126 MHz, $CDCl_3$) δ 170.24, 170.13, 152.49, 152.39, 144.07, 143.92, 132.94, 132.82, 129.58, 129.26, 129.08, 129.01, 129.00, 111.49, 111.36, 66.82, 65.82, 51.55, 51.11,

36.43, 34.41, 29.45, 29.39, 19.92, 19.88, 14.64, 14.31, 14.24, 13.73, 13.69, 13.56, 13.41.

IR (film): ν (cm^{-1}) 2961, 2933, 1727, 1586, 1455, 1411, 1386, 1364, 1325, 1311, 1290, 1134, 1030, 963, 871, 807, 756, 702, 611, 529.

HRMS (ESI, m/z) calcd for $C_{19}H_{26}N_2NaO_3S$ $(M+Na)^+$: 385.1556, found: 385.1558.

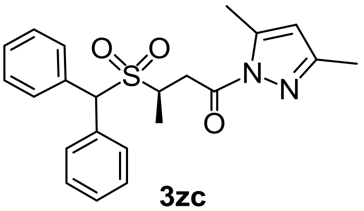

3zc

A dried 25 mL Schlenk tube was charged with **1zb** (336.5 mg, 2.0 mmol), **2a** (32.8 mg, 0.20 mmol), **PC1** (3.4 mg, 0.010 mmol), DABCO·$(SO_2)_2$ (36.0 mg, 0.15 mmol), chiral nickel catalyst **[L7-Ni]** (4.0 mL, taken from the above-mentioned freshly prepared solution in DCE), and DCE (4.0 mL). The mixture was degassed *via* three freeze-pump-thaw cycles. The Schlenk tube was positioned approximately 5 cm away from a 24 W blue LEDs lamp (λ_{max} = 455 nm). After being stirred at 0 ℃ for 24 h, the reaction mixture was concentrated, then purified by flash chromatography on silica gel (eluted with PE:EtOAc = 4:1) to afford product **3zb** as a white solid (57.0 mg, 0.144 mmol, 72% yield). Enantiomeric excess was established by HPLC analysis using a Chiralpak AD-H column, ee = 62% (HPLC: AD-H, 240 nm, *n*-hexane:isopropanol = 90:10, flow rate: 1.0 mL/min, 30 ℃, t_r(minor) = 24.1 min, t_r(major) = 22.5 min). $[\alpha]_D^{23}$ = +7.2 ° (*c* = 1.0, CH_2Cl_2). The configuration of **3zc** was assigned *R* by analogy to (*R*)-**3b**.

^{1}H NMR (600 MHz, $CDCl_3$) δ 7.70 – 7.64 (m, 4H), 7.42 – 7.35 (m, 6H), 5.95 (s, 1H), 5.54 (s, 1H), 3.81 (dd, *J* = 17.4, 4.6 Hz, 1H), 3.75 – 3.68 (m, 1H), 3.30 (dd, *J* = 17.4, 8.7 Hz, 1H), 2.49 (s, 3H), 2.21 (s, 3H), 1.39 (d, *J* = 6.9 Hz, 3H).

^{13}C NMR (126 MHz, $CDCl_3$) δ 170.17, 152.53, 144.08, 132.63, 132.12, 130.11, 129.71, 129.05, 128.90, 128.87, 128.48, 127.56, 126.52, 111.49, 70.98, 52.52, 35.47, 14.44, 14.35, 13.76.

IR (film): ν (cm^{-1}) 2926, 2853, 1719, 1584, 1451, 1378, 1323, 1309, 1131, 1029, 961, 866, 800, 752, 698, 596, 511.

HRMS (ESI, m/z) calcd for $C_{22}H_{24}N_2NaO_3S$ $(M+Na)^+$: 419.1400, found: 419.1400.

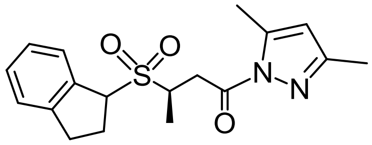

3zd

A dried 25 mL Schlenk tube was charged with **1zd** (236.4 mg, 2.0 mmol), **2a** (32.8 mg, 0.20 mmol), **PC1** (3.4 mg, 0.010 mmol), DABCO·$(SO_2)_2$ (36.0 mg, 0.15 mmol), chiral nickel catalyst **[L7-Ni]** (4.0 mL, taken from the above-mentioned freshly prepared solution in DCE), and DCE (4.0 mL). The mixture was degassed *via* three freeze-pump-thaw cycles. The Schlenk tube was positioned approximately 5 cm away from a 24 W blue LEDs lamp (λ_{max} = 455 nm). After being stirred at 0 ℃ for 35 h, the reaction mixture was concentrated, the diastereomeric ratio was determined as 1:1 dr by ^{1}H NMR analysis of the crude product. The residue was then purified by flash chromatography on silica gel (eluted with PE:Acetone = 4:1) to afford product **3zd** as a colorless oil (48.5 mg, 0.140 mmol, 70% yield). Enantiomeric excess was established by HPLC analysis using a Chiralpak OJ-H column, ee = 92%/91% (HPLC: OJ-H, 240 nm, *n*-hexane:isopropanol = 80:20, flow rate: 1.0 mL/min, 30 ℃, isomer I, t_r(minor) = 17.7 min, t_r(major) = 22.1 min), isomer II, t_r(minor) = 40.8 min, t_r(major) = 24.9 min). $[\alpha]_D^{23}$ = +8.5 ° (*c* = 1.0, CH_2Cl_2). The configuration of **3zd** was assigned *R* by analogy to (*R*)-**3b**.

^{1}H NMR (600 MHz, $CDCl_3$) δ 7.61 (dd, *J* = 36.2, 7.6 Hz, 1H), 7.34 – 7.28 (m, 2H), 7.26 – 7.22 (m, 1H), 5.98 (s, 1H), 4.79 – 4.72 (m, 1H), 4.02 – 3.90 (m, 1H), 3.90 – 3.79 (m, 1H), 3.37 – 3.27 (m, 2H), 3.02 – 2.95 (m, 1H), 2.80 – 2.72 (m, 1H), 2.60 – 2.50 (m, 4H), 2.22 (d, *J* = 5.9 Hz, 3H), 1.45 (dd, *J* = 33.2, 6.9 Hz, 3H).

^{13}C NMR (126 MHz, $CDCl_3$) δ 170.33, 170.29, 152.69, 152.66, 146.04, 146.02, 144.14, 134.07, 133.93, 129.41, 129.36, 126.91, 126.86, 126.82, 126.75, 126.71, 125.22, 125.16,

111.58, 66.44, 65.92, 51.02, 36.07, 35.72, 31.30, 31.26, 27.09, 26.98, 14.37, 14.19, 14.11, 13.78, 13.76.

IR (film): ν (cm^{-1}) 2923, 1721, 1654, 1586, 1450, 1407, 1377, 1323, 1302, 1042, 957, 873, 785, 752, 714, 615, 501.

HRMS (ESI, m/z) calcd for $C_{18}H_{22}N_2NaO_3S$ $(M+Na)^+$: 369.1243, found: 369.1248.

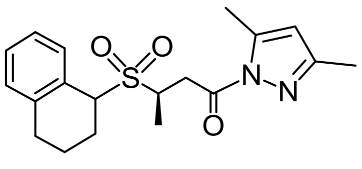

3ze

A dried 25 mL Schlenk tube was charged with **1ze** (264.4 mg, 2.0 mmol), **2a** (32.8 mg, 0.20 mmol), **PC1** (3.4 mg, 0.010 mmol), DABCO·$(SO_2)_2$ (36.0 mg, 0.15 mmol), chiral nickel catalyst **[L7-Ni]** (4.0 mL, taken from the above-mentioned freshly prepared solution in DCE), and DCE (4.0 mL). The mixture was degassed *via* three freeze-pump-thaw cycles. The Schlenk tube was positioned approximately 5 cm away from a 24 W blue LEDs lamp (λ_{max} = 455 nm). After being stirred at 0 ℃ for 45 h, the reaction mixture was concentrated, the diastereomeric ratio was determined as 1:1 dr by ^{1}H NMR analysis of the crude product. The residue was then purified by flash chromatography on silica gel (eluted with CH_2Cl_2) to afford product **3ze** as a colorless oil (49.7 mg, 0.138 mmol, 69% yield). Enantiomeric excess was established by HPLC analysis using a Chiralpak OJ-H column, ee = 88%/81% (HPLC: OJ-H, 240 nm, *n*-hexane:isopropanol = 80:20, flow rate: 1.0 mL/min, 30 ℃, isomer I, t_r(minor) = 44.1 min, t_r(major) = 14.3 min, isomer II, t_r(minor) = 12.0 min, t_r(major) = 19.8 min). $[\alpha]_D^{23}$ = +11.9 °(*c* = 1.0, CH_2Cl_2). The configuration of **3ze** was assigned *R* by analogy to (*R*)-**3b**.

^{1}H NMR (600 MHz, $CDCl_3$) δ 7.57 – 7.47 (m, 1H), 7.26 – 7.15 (m, 3H), 5.97 (d, *J* = 6.3 Hz, 1H), 4.56 – 4.51 (m, 1H), 4.09 – 3.97 (m, 1H), 3.84 – 3.78 (m, 1H), 3.36 – 3.27 (m, 1H), 3.00 – 2.92 (m, 1H), 2.84 – 2.74 (m, 1H), 2.63 – 2.55 (m, 1H), 2.53 (dd, *J* = 8.3, 1.0 Hz, 3H), 2.34 – 2.26 (m, 1H), 2.24 – 2.14 (m, 4H), 1.79 – 1.73 (m, 1H), 1.42 (dd, *J* = 23.0, 6.9 Hz, 3H).

^{13}C NMR (151 MHz, $CDCl_3$) δ 170.35, 170.32, 152.64, 152.62, 144.13, 139.94, 131.31, 131.24, 129.68, 128.60, 128.58, 126.63, 126.57, 125.88, 125.84, 111.56, 111.54, 60.23, 59.93, 50.99, 50.89, 36.11, 35.85, 28.61, 28.52, 23.69, 23.50, 19.33, 19.23, 14.37, 14.35, 14.32, 14.18, 14.05, 13.75.

IR (film): ν (cm^{-1}) 2929, 1720, 1584, 1450, 1407, 1379, 1323, 1297, 1125, 1027, 962, 862, 796, 741, 702, 591.

HRMS (ESI, m/z) calcd for $C_{19}H_{24}N_2NaO_3S$ $(M+Na)^+$: 383.1400, found: 383.1401.

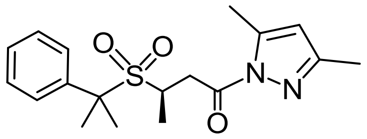

3zf

A dried 25 mL Schlenk tube was charged with **1zf** (240.4 mg, 2.0 mmol), **2a** (32.8 mg, 0.20 mmol), **PC1** (3.4 mg, 0.010 mmol), DABCO·$(SO_2)_2$ (36.0 mg, 0.15 mmol), chiral nickel catalyst **[L7-Ni]** (4.0 mL, taken from the above-mentioned freshly prepared solution in DCE), and DCE (4.0 mL). The mixture was degassed *via* three freeze-pump-thaw cycles. The Schlenk tube was positioned approximately 5 cm away from a 24 W blue LEDs lamp (λ_{max} = 455 nm). After being stirred at 0 ℃ for 30 h, the reaction mixture was concentrated, then purified by flash chromatography on silica gel (eluted with CH_2Cl_2) to afford product **3zf** as a white solid (46.7 mg, 0.134 mmol, 67% yield). Enantiomeric excess was established by HPLC analysis using a Chiralpak AD-H column, ee = 89% (HPLC: AD-H, 220 nm, *n*-hexane:isopropanol = 90:10, flow rate: 1.0 mL/min, 30 ℃, t_r(minor) = 9.2 min, t_r(major) = 9.8 min). $[\alpha]_D^{23}$ = +9.4 ° (*c* = 1.0, CH_2Cl_2). The configuration of **3zf** was assigned *R* by analogy to (*R*)-**3b**.

^1H NMR (500 MHz, $CDCl_3$) δ 7.71 – 7.66 (m, 2H), 7.39 – 7.34 (m, 2H), 7.34 – 7.29 (m, 1H), 5.93 (s, 1H), 3.95 – 3.86 (m, 1H), 3.56 (dd, *J* = 17.8, 5.4 Hz, 1H), 2.94 (dd, *J* = 17.8, 7.7 Hz, 1H), 2.49 (s, 3H), 2.20 (s, 3H), 1.89 (d, *J* = 13.1 Hz, 6H), 0.92 (d, *J* = 7.0 Hz, 3H).

^{13}C NMR (126 MHz, $CDCl_3$) δ 170.46, 152.26, 143.94, 137.48, 128.60, 128.54, 128.15, 111.31, 66.06, 49.54, 37.02, 22.98, 22.84, 15.83, 14.32, 13.72.

IR (film): ν (cm^{-1}) 2925, 2852, 1725, 1585, 1457, 1411, 1384, 1323, 1292, 1120, 1095, 962, 861, 804, 755, 621, 527.

HRMS (ESI, m/z) calcd for $C_{18}H_{24}N_2NaO_3S$ $(M+Na)^+$: 371.1400, found: 371.1404.

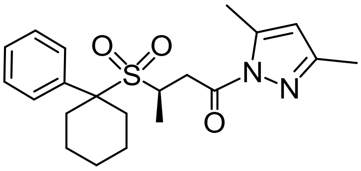

3zg

A dried 25 mL Schlenk tube was charged with **1zg** (320.5 mg, 2.0 mmol), **2a** (32.8 mg, 0.20 mmol), **PC1** (3.4 mg, 0.010 mmol), DABCO·$(SO_2)_2$ (36.0 mg, 0.15 mmol), chiral nickel catalyst **[L7-Ni]** (4.0 mL, taken from the above-mentioned freshly prepared solution in DCE), and DCE (4.0 mL). The mixture was degassed *via* three freeze-pump-thaw cycles. The Schlenk tube was positioned approximately 5 cm away from a 24 W blue LEDs lamp (λ_{max} = 455 nm). After being stirred at 0 ℃ for 72 h, the reaction mixture was concentrated, then purified by flash chromatography on silica gel (eluted with CH_2Cl_2) to afford product **3zg** as a colorless oil (50.5 mg, 0.130 mmol, 65% yield). Enantiomeric excess was established by HPLC analysis using a Chiralpak AD-H column, ee = 73% (HPLC: AD-H, 220 nm, *n*-hexane:isopropanol = 90:10, flow rate: 1.0 mL/min, 30 ℃, t_r(minor) = 10.4 min, t_r(major) = 11.8 min). $[\alpha]_D^{23}$ = +16.5 ° (*c* = 1.0, CH_2Cl_2). The configuration of **3zg** was assigned *R* by analogy to (*R*)-**3b**.

^{1}H NMR (500 MHz, $CDCl_3$) δ 7.70 – 7.63 (m, 2H), 7.44 – 7.37 (m, 2H), 7.33 – 7.28 (m, 1H), 5.93 (s, 1H), 3.87 – 3.79 (m, 1H), 3.47 (dd, *J* = 17.9, 5.5 Hz, 1H), 2.90 (dd, *J* = 17.9, 7.6 Hz, 1H), 2.83 – 2.70 (m, 2H), 2.49 (s, 3H), 2.26 – 2.16 (m, 5H), 1.77 – 1.64 (m, 3H), 1.37 – 1.25 (m, 3H), 0.85 (d, *J* = 7.0 Hz, 3H).

^{13}C NMR (151 MHz, $CDCl_3$) δ 170.55, 152.17, 143.89, 133.26, 129.67, 128.87, 128.44, 111.25, 70.89, 49.09, 37.02, 28.77, 28.68, 25.44, 21.61, 21.51, 15.71, 14.35, 13.72.

IR (film): ν (cm^{-1}) 2930, 2859, 1724, 1585, 1456, 1410, 1384, 1364, 1323, 1301, 1288, 1134, 1110, 1030, 963, 872, 801, 748, 698, 602, 517.

HRMS (ESI, m/z) calcd for $C_{21}H_{28}N_2NaO_3S$ $(M+Na)^+$: 411.1713, found: 411.1718.

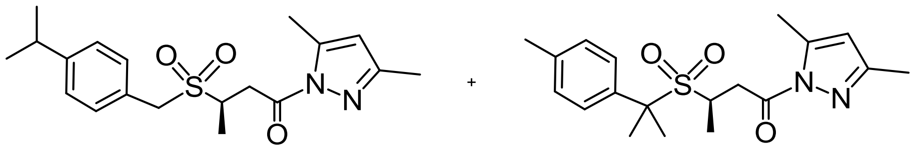

3zh + **3zh'**

A dried 25 mL Schlenk tube was charged with **1zh** (268.4 mg, 2.0 mmol), **2a** (32.8 mg, 0.20 mmol), **PC1** (3.4 mg, 0.010 mmol), DABCO·$(SO_2)_2$ (36.0 mg, 0.15 mmol), chiral nickel catalyst **[L7-Ni]** (4.0 mL, taken from the above-mentioned freshly prepared solution in DCE), and DCE (4.0 mL). The mixture was degassed *via* three freeze-pump-thaw cycles. The Schlenk tube was positioned approximately 5 cm away from a 24 W blue LEDs lamp (λ_{max} = 455 nm). After being stirred at 0 ℃ for 40 h, the reaction mixture was concentrated, the regioselectivity was determined as 1.5:1 rr by ^{1}H NMR analysis of the crude product. The residue was then purified by flash chromatography on silica gel (eluted with CH_2Cl_2) to afford product **3zh** as a colorless oil (28.9 mg, 0.080 mmol, 40% yield). Enantiomeric excess was established by HPLC analysis using a Chiralpak AD-H column, ee = 94% (HPLC: AD-H, 240 nm, *n*-hexane:isopropanol = 90:10, flow rate: 1.0 mL/min, 30 ℃, t_r(minor) = 14.3 min, t_r(major) = 16.9 min). $[\alpha]_D^{23}$ = +20.6 ° (*c* = 1.0, CH_2Cl_2). The configuration of **3zh** was assigned *R* by analogy to (*R*)-**3b**.

^{1}H NMR (600 MHz, $CDCl_3$) δ 7.39 – 7.34 (m, 2H), 7.26 – 7.23 (m, 2H), 5.97 (s, 1H), 4.28 (s, 2H), 3.79 (dd, *J* = 17.5, 4.0 Hz, 1H), 3.75 – 3.64 (m, 1H), 3.36 (dd, *J* = 17.5, 9.2 Hz, 1H), 2.95 – 2.87 (m, 1H), 2.52 (s, 3H), 2.22 (s, 3H), 1.46 (d, *J* = 7.0 Hz, 3H), 1.24 (d, *J* = 6.9 Hz, 6H).

^{13}C NMR (151 MHz, $CDCl_3$) δ 170.13, 152.66, 149.80, 144.12, 130.70, 127.13, 124.57,

111.58, 56.67, 52.23, 35.56, 33.85, 23.81, 14.33, 14.08, 13.75.

IR (film): ν (cm^{-1}) 2929, 2872, 1724, 1585, 1512, 1385, 1325, 1122, 962, 827, 756, 588, 507.

HRMS (ESI, m/z) calcd for $C_{19}H_{26}N_2NaO_3S$ $(M+Na)^+$: 385.1556, found: 385.1559.

The minor product **3zh'** was isolated as a colorless oil (18.8 mg, 0.052 mmol, 26% yield). Enantiomeric excess was established by HPLC analysis using a Chiralpak OJ-H column, ee = 76% (HPLC: OJ-H, 240 nm, *n*-hexane:isopropanol = 90:10, flow rate: 1.0 mL/min, 30 ℃, t_r(minor) = 11.6 min, t_r(major) = 21.4 min). $[\alpha]_D^{23}$ = +8.3 ° (*c* = 1.0, CH_2Cl_2). The configuration of **3zh'** was assigned *R* by analogy to (*R*)-**3b**.

^{1}H NMR (600 MHz, $CDCl_3$) δ 7.61 – 7.50 (m, 2H), 7.15 (d, *J* = 8.0 Hz, 2H), 5.93 (s, 1H), 3.93 – 3.84 (m, 1H), 3.50 (dd, *J* = 18.0, 5.2 Hz, 1H), 2.92 (dd, *J* = 17.9, 7.8 Hz, 1H), 2.49 (s, 3H), 2.28 (s, 3H), 2.20 (s, 3H), 1.86 (d, *J* = 13.5 Hz, 6H), 0.96 (d, *J* = 7.0 Hz, 3H).

^{13}C NMR (151 MHz, $CDCl_3$) δ 170.55, 152.21, 143.89, 138.60, 134.39, 129.23, 128.01, 111.26, 65.84, 49.31, 37.14, 22.94, 22.84, 20.92, 15.82, 14.34, 13.73.

IR (film): ν (cm^{-1}) 2927, 2851, 1723, 1659, 1584, 1385, 1129, 1112, 961, 755, 685, 566, 504.

HRMS (ESI, m/z) calcd for $C_{19}H_{26}N_2NaO_3S$ $(M+Na)^+$: 385.1556, found: 385.1558.

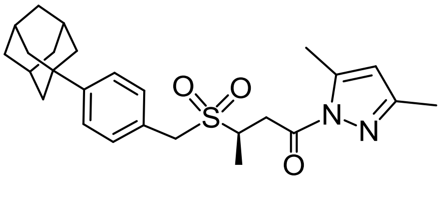

3zi

A dried 25 mL Schlenk tube was charged with **1zi** (452.7 mg, 2.0 mmol), **2a** (32.8 mg, 0.20 mmol), **PC1** (3.4 mg, 0.010 mmol), DABCO·$(SO_2)_2$ (36.0 mg, 0.15 mmol), chiral nickel catalyst **[L7-Ni]** (4.0 mL, taken from the above-mentioned freshly prepared solution in DCE), and DCE (4.0 mL). The mixture was degassed *via* three freeze-pump-thaw cycles. The Schlenk tube was positioned approximately 5 cm away from a 24 W blue LEDs lamp (λ_{max} = 455 nm). After being stirred at 0 ℃ for 18 h, the reaction mixture was concentrated, then

purified by flash chromatography on silica gel (eluted with CH_2Cl_2) to afford product **3zi** as a colorless oil (57.2 mg, 0.126 mmol, 63% yield). Enantiomeric excess was established by HPLC analysis using a Chiralpak AD-H column, ee = 90% (HPLC: AD-H, 240 nm, *n*-hexane:isopropanol = 90:10, flow rate: 1.0 mL/min, 30 ℃, t_r(minor) = 17.9 min, t_r(major) = 22.0 min). $[\alpha]_D^{23}$ = +27.5 ° (*c* = 1.0, CH_2Cl_2). The configuration of **3zi** was assigned *R* by analogy to (*R*)-**3b**.

^{1}H NMR (500 MHz, $CDCl_3$) δ 7.38 (s, 4H), 5.97 (s, 1H), 4.28 (s, 2H), 3.76 (dd, *J* = 17.4, 3.9 Hz, 1H), 3.72 – 3.66 (m, 1H), 3.35 (dd, *J* = 17.4, 9.2 Hz, 1H), 2.52 (s, 3H), 2.22 (s, 3H), 2.11 – 2.08 (m, 3H), 1.89 (d, *J* = 2.9 Hz, 5H), 1.80 – 1.73 (m, 7H), 1.46 (d, *J* = 6.9 Hz, 3H).

^{13}C NMR (151 MHz, $CDCl_3$) δ 170.14, 152.64, 152.25, 144.12, 130.47, 125.61, 124.26, 111.57, 56.66, 52.26, 43.00, 36.69, 36.16, 35.58, 28.84, 14.34, 14.08, 13.77.

IR (film): ν (cm^{-1}) 2906, 2848, 1726, 1586, 1451, 1387, 1326, 1301, 1288, 1125, 1018, 963, 806, 734, 610, 512.

HRMS (ESI, m/z) calcd for $C_{26}H_{34}N_2NaO_3S$ $(M+Na)^+$: 477.2182, found: 477.2189.

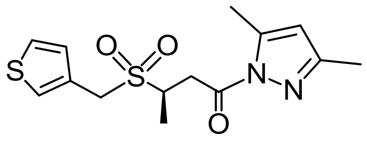

3zj

A dried 25 mL Schlenk tube was charged with **1zj** (196.3 mg, 2.0 mmol), **2a** (32.8 mg, 0.20 mmol), **PC1** (3.4 mg, 0.010 mmol), DABCO·$(SO_2)_2$ (36.0 mg, 0.15 mmol), chiral nickel catalyst **[L7-Ni]** (4.0 mL, taken from the above-mentioned freshly prepared solution in DCE), and DCE (4.0 mL). The mixture was degassed *via* three freeze-pump-thaw cycles. The Schlenk tube was positioned approximately 5 cm away from a 24 W blue LEDs lamp (λ_{max} = 455 nm). After being stirred at 20 ℃ for 40 h, the reaction mixture was concentrated, then purified by flash chromatography on silica gel (eluted with PE:EtOAc = 4:1) to afford product **3zj** as a yellow oil (35.3 mg, 0.108 mmol, 54% yield). Enantiomeric excess was established by HPLC analysis using a Chiralpak AD-H column, ee = 82% (HPLC: AD-H,

240 nm, *n*-hexane:isopropanol = 90:10, flow rate: 1.0 mL/min, 30 ℃, t_r(minor) = 21.1 min, t_r(major) = 24.1 min). $[\alpha]_D^{23}$ = +11.3 ° (*c* = 1.0, CH_2Cl_2). The configuration of **3zj** was assigned *R* by analogy to (*R*)-**3b**.

^{1}H NMR (600 MHz, $CDCl_3$) δ 7.48 – 7.41 (m, 1H), 7.41 – 7.35 (m, 1H), 7.21 (dd, *J* = 5.0, 1.3 Hz, 1H), 5.97 (s, 1H), 4.38 (s, 2H), 3.79 (dd, *J* = 17.4, 4.2 Hz, 1H), 3.74 – 3.64 (m, 1H), 3.33 (dd, *J* = 17.4, 9.0 Hz, 1H), 2.52 (s, 3H), 2.22 (s, 3H), 1.44 (d, *J* = 7.0 Hz, 3H).

^{13}C NMR (151 MHz, $CDCl_3$) δ 170.06, 152.71, 144.16, 128.87, 127.25, 126.76, 126.69, 111.62, 52.15, 51.98, 35.64, 14.35, 14.02, 13.77.

IR (film): ν (cm^{-1}) 2925, 2851, 1722, 1585, 1408, 1384, 1324, 1307, 1137, 1110, 1027, 962, 868, 797, 628, 571, 501.

HRMS (ESI, m/z) calcd for $C_{14}H_{18}N_2NaO_3S_2$ $(M+Na)^+$: 349.0651, found: 349.0658.

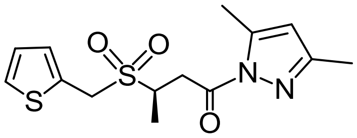

3zk

A dried 25 mL Schlenk tube was charged with **1zk** (196.3 mg, 2.0 mmol), **2a** (32.8 mg, 0.20 mmol), **PC1** (3.4 mg, 0.010 mmol), DABCO·$(SO_2)_2$ (36.0 mg, 0.15 mmol), chiral nickel catalyst **[L7-Ni]** (4.0 mL, taken from the above-mentioned freshly prepared solution in DCE), and DCE (4.0 mL). The mixture was degassed *via* three freeze-pump-thaw cycles. The Schlenk tube was positioned approximately 5 cm away from a 24 W blue LEDs lamp (λ_{max} = 455 nm). After being stirred at 0 ℃ for 24 h, the reaction mixture was concentrated, then purified by flash chromatography on silica gel (eluted with PE:EtOAc = 4:1) to afford product **3zk** as a colorless oil (39.2 mg, 0.120 mmol, 60% yield). Enantiomeric excess was established by HPLC analysis using a Chiralpak AD-H column, ee = 88% (HPLC: AD-H, 240 nm, *n*-hexane:isopropanol = 90:10, flow rate: 1.0 mL/min, 30 ℃, t_r(minor) = 19.6 min, t_r(major) = 21.7 min). $[\alpha]_D^{23}$ = +7.4 ° (*c* = 1.0, CH_2Cl_2). The configuration of **3zk** was assigned *R* by analogy to (*R*)-**3b**.

^{1}H NMR (600 MHz, $CDCl_3$) δ 7.37 (dd, *J* = 5.1, 1.2 Hz, 1H), 7.21 (dd, *J* = 3.6, 1.1 Hz, 1H), 7.05 (dd, *J* = 5.2, 3.5 Hz, 1H), 5.98 (s, 1H), 4.54 (d, *J* = 2.7 Hz, 2H), 3.83 – 3.73 (m, 2H), 3.37 (dd, *J* = 17.1, 8.5 Hz, 1H), 2.52 (s, 3H), 2.22 (s, 3H), 1.46 (d, *J* = 6.9 Hz, 3H).

^{13}C NMR (151 MHz, $CDCl_3$) δ 170.02, 152.74, 144.17, 130.25, 127.95, 127.77, 127.54, 111.62, 51.91, 51.90, 35.55, 14.34, 14.12, 13.77.

IR (film): ν (cm^{-1}) 2919, 2849, 1717, 1652, 1385, 1309, 1050, 952, 813, 791, 662, 578, 529, 505.

HRMS (ESI, m/z) calcd for $C_{14}H_{18}N_2NaO_3S_2$ $(M+Na)^+$: 349.0651, found: 349.0661.

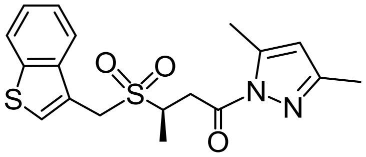

3zl

A dried 25 mL Schlenk tube was charged with **1zl** (296.4 mg, 2.0 mmol), **2a** (32.8 mg, 0.20 mmol), **PC1** (3.4 mg, 0.010 mmol), DABCO·$(SO_2)_2$ (36.0 mg, 0.15 mmol), chiral nickel catalyst **[L7-Ni]** (4.0 mL, taken from the above-mentioned freshly prepared solution in DCE), and DCE (4.0 mL). The mixture was degassed *via* three freeze-pump-thaw cycles. The Schlenk tube was positioned approximately 5 cm away from a 24 W blue LEDs lamp (λ_{max} = 455 nm). After being stirred at 20 ℃ for 30 h, the reaction mixture was concentrated, then purified by flash chromatography on silica gel (eluted with PE:EtOAc = 4:1) to afford product **3zl** as a colorless oil (38.4 mg, 0.102 mmol, 51% yield). Enantiomeric excess was established by HPLC analysis using a Chiralpak AD-H column, ee = 76% (HPLC: AD-H, 240 nm, *n*-hexane:isopropanol = 90:10, flow rate: 1.0 mL/min, 30 ℃, t_r(minor) = 26.7 min, t_r(major) = 28.8 min). $[\alpha]_D^{23}$ = +9.8 °(*c* = 1.0, CH_2Cl_2). The configuration of **3zl** was assigned *R* by analogy to (*R*)-**3b**.

^{1}H NMR (600 MHz, $CDCl_3$) δ 7.95 – 7.83 (m, 2H), 7.70 (s, 1H), 7.48 – 7.37 (m, 2H), 5.97 (s, 1H), 4.62 (dd, *J* = 4.5, 0.9 Hz, 2H), 3.86 (dd, *J* = 17.3, 4.4 Hz, 1H), 3.83 – 3.77 (m, 1H), 3.33 (dd, *J* = 17.3, 8.5 Hz, 1H), 2.50 (s, 3H), 2.22 (s, 3H), 1.46 (d, *J* = 6.9 Hz, 3H).

^{13}C NMR (151 MHz, $CDCl_3$) δ 170.02, 152.74, 144.19, 140.08, 138.06, 128.89, 124.87, 124.69, 122.89, 121.94, 121.92, 111.62, 52.64, 50.39, 35.93, 14.33, 14.17, 13.77.

IR (film): ν (cm^{-1}) 2922, 2850, 1717, 1654, 1583, 1408, 1384, 1363, 1324, 1306, 1113, 1076, 1041, 961, 887, 800, 757, 613, 597, 500

HRMS (ESI, m/z) calcd for $C_{18}H_{20}N_2NaO_3S_2$ $(M+Na)^+$: 399.0808, found: 399.0811.

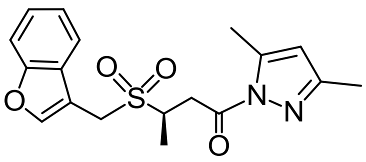

3zm

A dried 25 mL Schlenk tube was charged with **1zm** (264.3 mg, 2.0 mmol), **2a** (32.8 mg, 0.20 mmol), **PC1** (3.4 mg, 0.010 mmol), DABCO·$(SO_2)_2$ (36.0 mg, 0.15 mmol), chiral nickel catalyst **[L7-Ni]** (4.0 mL, taken from the above-mentioned freshly prepared solution in DCE), and DCE (4.0 mL). The mixture was degassed *via* three freeze-pump-thaw cycles. The Schlenk tube was positioned approximately 5 cm away from a 24 W blue LEDs lamp (λ_{max} = 455 nm). After being stirred at 20 ℃ for 30 h, the reaction mixture was concentrated, then purified by flash chromatography on silica gel (eluted with PE:EtOAc = 4:1) to afford product **3zm** as a colorless oil (25.9 mg, 0.072 mmol, 36% yield). Enantiomeric excess was established by HPLC analysis using a Chiralpak AD-H column, ee = 85% (HPLC: AD-H, 240 nm, *n*-hexane:isopropanol = 90:10, flow rate: 1.0 mL/min, 30 ℃, t_r(minor) = 23.1 min, t_r(major) = 27.6 min). $[\alpha]_D^{23}$ = +6.7 ° (*c* = 1.0, CH_2Cl_2). The configuration of **3zm** was assigned *R* by analogy to (*R*)-**3b**.

^{1}H NMR (600 MHz, $CDCl_3$) δ 7.84 (s, 1H), 7.75 – 7.70 (m, 1H), 7.53 – 7.49 (m, 1H), 7.37 – 7.32 (m, 1H), 7.32 – 7.28 (m, 1H), 5.97 (s, 1H), 4.46 (dd, *J* = 12.3, 0.9 Hz, 2H), 3.90 – 3.80 (m, 2H), 3.35 (dd, *J* = 17.1, 8.3 Hz, 1H), 2.50 (s, 3H), 2.21 (s, 3H), 1.48 (d, *J* = 6.9 Hz, 3H).

^{13}C NMR (151 MHz, $CDCl_3$) δ 170.01, 155.31, 152.79, 145.46, 144.21, 126.71, 125.12, 123.34, 120.08, 111.75, 111.65, 108.00, 52.25, 47.01, 35.91, 14.33, 14.12, 13.74.

IR (film): ν (cm^{-1}) 2917, 2855, 1715, 1646, 1582, 1452, 1407, 1376, 1324, 1308, 1104, 1035, 960, 883, 811, 745, 616, 599, 512.

HRMS (ESI, m/z) calcd for $C_{18}H_{20}N_2NaO_4S$ $(M+Na)^+$: 383.1036, found: 383.1042.

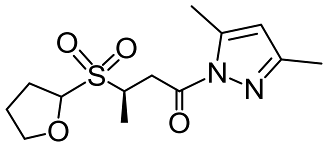

3zn

A dried 25 mL Schlenk tube was charged with **1zn** (144.2 mg, 2.0 mmol), **2a** (32.8 mg, 0.20 mmol), **PC1** (3.4 mg, 0.010 mmol), DABCO·$(SO_2)_2$ (36.0 mg, 0.15 mmol), chiral nickel catalyst **[L7-Ni]** (4.0 mL, taken from the above-mentioned freshly prepared solution in DCE), and DCE (4.0 mL). The mixture was degassed *via* three freeze-pump-thaw cycles. The Schlenk tube was positioned approximately 5 cm away from a 24 W blue LEDs lamp (λ_{max} = 455 nm). After being stirred at 20 ℃ for 35 h, the reaction mixture was concentrated, the diastereomeric ratio was determined as 1.5:1 dr by ^{1}H NMR analysis of the crude product. The residue was then purified by flash chromatography on silica gel (eluted with PE/EtOAc = 4:1) to afford product **3zn** as a diastereomeric mixture (33.0 mg, 0.110 mmol, 55% yield). Enantiomeric excess was established by HPLC analysis using a Chiralpak AD-H column, ee = 80%/82% (HPLC: AD-H, 240 nm, *n*-hexane:isopropanol = 90:10, flow rate: 1.0 mL/min, 30 ℃, isomer I, t_r(minor) = 13.9 min, t_r(major) = 11.9 min, isomer II, t_r(minor) = 14.8 min, t_r(major) = 15.4 min). $[\alpha]_D^{23}$ = +9.2 ° (*c* = 1.0, CH_2Cl_2). The configuration of **3zn** was assigned *R* by analogy to (*R*)-**3b**.

^{1}H NMR (600 MHz, $CDCl_3$) δ 5.97 (s, 1H), 5.11 – 5.06 (m, 1H), 4.18 – 4.11 (m, 1H), 4.08 – 3.95 (m, 2H), 3.91 (dd, *J* = 17.9, 4.1 Hz, 0.6H), 3.80 (dd, *J* = 17.7, 4.5 Hz, 0.4H), 3.44 – 3.37 (m, 1H), 2.67 – 2.60 (m, 1H), 2.53 (s, 3H), 2.32 – 2.16 (m, 5H), 1.99 – 1.93 (m, 1H), 1.51 (dd, *J* = 29.0, 7.0 Hz, 3H).

^{13}C NMR (151 MHz, $CDCl_3$) δ 170.42, 170.32, 152.55, 152.47, 144.17, 144.10, 111.46,

111.40, 90.65, 90.05, 71.09, 51.62, 50.86, 36.64, 34.57, 25.27, 25.11, 24.92, 24.90, 14.37, 14.36, 13.77, 13.18.

IR (film): ν (cm^{-1}) 2924, 2849, 1725, 1652, 1542, 1385, 1161, 1048, 873, 755, 656, 605, 588, 502.

HRMS (ESI, m/z) calcd for $C_{13}H_{20}N_2NaO_4S$ $(M+Na)^+$: 323.1036, found: 323.1024.

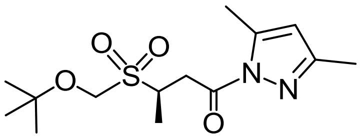

3zo

A dried 25 mL Schlenk tube was charged with **1zo** (176.3 mg, 2.0 mmol), **2a** (32.8 mg, 0.20 mmol), **PC1** (3.4 mg, 0.010 mmol), DABCO·$(SO_2)_2$ (36.0 mg, 0.15 mmol), chiral nickel catalyst **[L7-Ni]** (4.0 mL, taken from the above-mentioned freshly prepared solution in DCE), and DCE (4.0 mL). The mixture was degassed *via* three freeze-pump-thaw cycles. The Schlenk tube was positioned approximately 5 cm away from a 24 W blue LEDs lamp (λ_{max} = 455 nm). After being stirred at 0 ℃ for 45 h, the reaction mixture was concentrated, then purified by flash chromatography on silica gel (eluted with PE:EtOAc = 4:1) to afford product **1zo** as a colorless oil (46.2 mg, 0.146 mmol, 73% yield). Enantiomeric excess was established by HPLC analysis using a Chiralpak AS-H column, ee = 92% (HPLC: AS-H, 240 nm, *n*-hexane:isopropanol = 90:10, flow rate: 1.0 mL/min, 30 ℃, t_r(minor) = 15.2 min, t_r(major) = 23.4 min). $[\alpha]_D^{23}$ = +12.5 ° (*c* = 1.0, CH_2Cl_2). The configuration of **3zo** was assigned *R* by analogy to (*R*)-**3b**.

^{1}H NMR (600 MHz, $CDCl_3$) δ 5.97 (s, 1H), 4.56 – 4.42 (m, 2H), 3.96 – 3.90 (m, 1H), 3.86 (dd, *J* = 17.8, 4.3 Hz, 1H), 3.39 (dd, *J* = 17.8, 8.8 Hz, 1H), 2.53 (s, 3H), 2.21 (s, 3H), 1.48 (d, *J* = 7.0 Hz, 3H), 1.25 (s, 9H).

^{13}C NMR (151 MHz, $CDCl_3$) δ 170.33, 152.48, 144.06, 111.43, 77.67, 76.19, 51.74, 35.45, 27.35, 14.35, 13.73, 13.63.

IR (film): ν (cm^{-1}) 2976, 2927, 1724, 1585, 1458, 1411, 1386, 1326, 1308, 1262, 1137, 1097,

1029, 962, 867, 801, 744, 600, 515.

HRMS (ESI, m/z) calcd for $C_{14}H_{24}N_2NaO_4S$ $(M+Na)^+$: 339.1349, found: 339.1349.

3.3 Substrate Scope of α,β-Unsaturated *N*-Acylpyrazoles Containing a β-Alkyl Substituent

3.3.1 Preparation of a Solution of the Non-Racemic Nickel Catalyst [L7-Ni] in DCE

According to the published procedure,[7,8] a solution of $Ni(ClO_4)_2{\cdot}6H_2O$ (11.0 mg, 0.030 mmol) and non-racemic ligand **L7** (18.4 mg, 0.036 mmol) in 1,2-dimethoxyethane (DME, 4.0 mL) was stirred at 75 °C for 5 h, then the resulting solution was concentrated under reduced pressure to remove the solvent. The residue was redissolved in dichloroethane (DCE, 4.0 mL), which was used freshly as the metal catalyst for the photochemical reactions.

3.3.2 General Procedure

A dried 25 mL Schlenk tube was charged with **1a** (2.0 mmol), **2f−2k** (0.20 mmol), **PC1** (3.4 mg, 0.010 mmol), $DABCO{\cdot}(SO_2)_2$ (36.0 mg, 0.15 mmol), chiral nickel catalyst **[L7-Ni]** (4.0 mL, taken from the above-mentioned freshly prepared solution in DCE), and DCE (4.0 mL). The mixture was degassed *via* three freeze-pump-thaw cycles. The Schlenk tube was positioned approximately 5 cm away from a 24 W blue LEDs lamp (λ_{max} = 455 nm). After being stirred at 0 ℃ for 44–72 h (monitored by TLC analysis), the reaction mixture was concentrated, then purified by flash chromatography on silica gel (eluted with CH_2Cl_2 or PE:EtOAc = 4:1) to afford non-racemic product **3zp–3zu**.

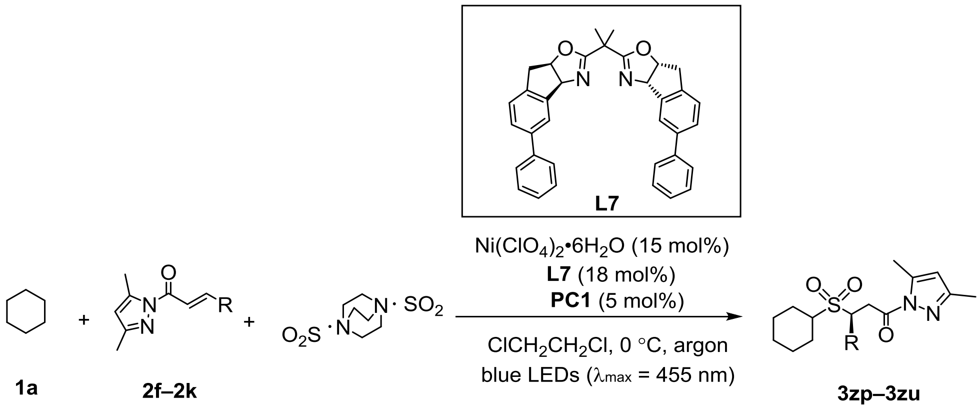

3.3.3 Experimental Details and Characterization Data

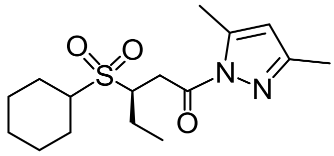

3zp

A dried 25 mL Schlenk tube was charged with **1a** (168.3 mg, 2.0 mmol), **2f** (35.6 mg, 0.20 mmol), **PC1** (3.4 mg, 0.010 mmol), DABCO·$(SO_2)_2$ (36.0 mg, 0.15 mmol), chiral nickel catalyst **[L7-Ni]** (4.0 mL, taken from the above-mentioned freshly prepared solution in DCE), and DCE (4.0 mL). The mixture was degassed *via* three freeze-pump-thaw cycles. The Schlenk tube was positioned approximately 5 cm away from a 24 W blue LEDs lamp (λ_{max} = 455 nm). After being stirred at 0 ℃ for 48 h, the reaction mixture was concentrated, then purified by flash chromatography on silica gel (eluted with CH_2Cl_2) to afford product **3zp** as a colorless oil (45.7 mg, 0.140 mmol, 70% yield). Enantiomeric excess was established by HPLC analysis using a Chiralpak AS-H column, ee = 93% (HPLC: AS-H, 240 nm, *n*-hexane:isopropanol = 90:10, flow rate: 1.0 mL/min, 30 ℃, t_r(minor) = 17.8 min, t_r(major) = 11.3 min). $[\alpha]_D^{23}$ = +5.4 ° (*c* = 1.0, CH_2Cl_2). The configuration of **3zp** was assigned *R* by analogy to (*R*)-**3b**.

^{1}H NMR (600 MHz, $CDCl_3$) δ 5.98 (s, 1H), 3.86 – 3.73 (m, 2H), 3.39 – 3.31 (m, 1H), 3.04 – 2.97 (m, 1H), 2.53 (s, 3H), 2.23 (s, 3H), 2.22 – 2.14 (m, 2H), 2.09 – 2.03 (m, 1H), 1.96 – 1.91

(m, 2H), 1.84 – 1.78 (m, 1H), 1.74 – 1.70 (m, 1H), 1.62 – 1.56 (m, 2H), 1.29 – 1.23 (m, 3H), 1.08 (t, *J* = 7.5 Hz, 3H).

^{13}C NMR (151 MHz, $CDCl_3$) δ 170.76, 152.60, 144.19, 111.54, 59.29, 55.18, 33.70, 25.14, 25.11, 25.00, 24.63, 21.62, 14.39, 13.78, 11.19.

IR (film): ν (cm^{-1}) 2857, 1723, 1584, 1450, 1382, 1324, 1297, 1090, 1044, 958, 893, 801, 756, 692, 608, 504.

HRMS (ESI, m/z) calcd for $C_{16}H_{26}N_2NaO_3S$ $(M+Na)^+$: 349.1556, found: 349.1556.

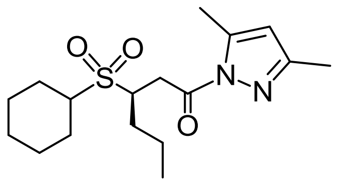

3zq

A dried 25 mL Schlenk tube was charged with **1a** (168.3 mg, 2.0 mmol), **2g** (38.5 mg, 0.20 mmol), **PC1** (3.4 mg, 0.010 mmol), DABCO·$(SO_2)_2$ (36.0 mg, 0.15 mmol), chiral nickel catalyst **[L7-Ni]** (4.0 mL, taken from the above-mentioned freshly prepared solution in DCE), and DCE (4.0 mL). The mixture was degassed *via* three freeze-pump-thaw cycles. The Schlenk tube was positioned approximately 5 cm away from a 24 W blue LEDs lamp (λ_{max} = 455 nm). After being stirred at 0 ℃ for 48 h, the reaction mixture was concentrated, then purified by flash chromatography on silica gel (eluted with CH_2Cl_2) to afford product **3zq** as a colorless oil (48.3 mg, 0.142 mmol, 71% yield). Enantiomeric excess was established by HPLC analysis using a Chiralpak AS-H column, ee = 93% (HPLC: AS-H, 240 nm, *n*-hexane:isopropanol = 90:10, flow rate: 1.0 mL/min, 30 ℃, t_r(minor) = 17.3 min, t_r(major) = 9.8 min). $[\alpha]_D^{23}$ = +4.5 ° (*c* = 1.0, CH_2Cl_2). The configuration of **3zq** was assigned *R* by analogy to (*R*)-**3b**.

^{1}H NMR (600 MHz, $CDCl_3$) δ 5.98 (s, 1H), 3.90 – 3.78 (m, 2H), 3.39 – 3.30 (m, 1H), 3.04 – 2.95 (m, 1H), 2.53 (s, 3H), 2.22 (s, 3H), 2.21 – 2.14 (m, 2H), 1.98 – 1.90 (m, 3H), 1.75 – 1.68 (m, 2H), 1.60 – 1.56 (m, 2H), 1.54 – 1.48 (m, 1H), 1.47 – 1.40 (m, 1H), 1.30 – 1.25 (m, 3H), 0.95 (t, *J* = 7.3 Hz, 3H).

^{13}C NMR (151 MHz, $CDCl_3$) δ 170.72, 152.60, 144.20, 111.52, 59.17, 53.79, 34.24, 30.40, 25.14, 25.13, 25.11, 25.00, 24.66, 19.98, 14.39, 13.90, 13.77.

IR (film): ν (cm^{-1}) 2928, 2853, 1723, 1582, 1451, 1378, 1323, 1297, 1089, 1034, 958, 887, 797, 750, 687, 609, 511.

HRMS (ESI, m/z) calcd for $C_{17}H_{28}N_2NaO_3S$ $(M+Na)^+$: 363.1713, found: 363.1713.

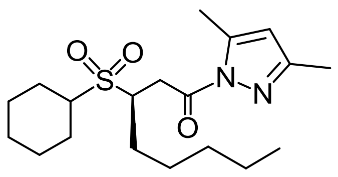

3zr

A dried 25 mL Schlenk tube was charged with **1a** (168.3 mg, 2.0 mmol), **2h** (44.1 mg, 0.20 mmol), **PC1** (3.4 mg, 0.010 mmol), DABCO·$(SO_2)_2$ (36.0 mg, 0.15 mmol), chiral nickel catalyst **[L7-Ni]** (4.0 mL, taken from the above-mentioned freshly prepared solution in DCE), and DCE (4.0 mL). The mixture was degassed *via* three freeze-pump-thaw cycles. The Schlenk tube was positioned approximately 5 cm away from a 24 W blue LEDs lamp (λ_{max} = 455 nm). After being stirred at 0 ℃ for 44 h, the reaction mixture was concentrated, then purified by flash chromatography on silica gel (eluted with CH_2Cl_2) to afford product **3zr** as a colorless oil (47.1 mg, 0.128 mmol, 64% yield). Enantiomeric excess was established by HPLC analysis using a Chiralpak AS-H column, ee = 90% (HPLC: AS-H, 240 nm, *n*-hexane:isopropanol = 90:10, flow rate: 1.0 mL/min, 30 ℃, t_r(minor) = 17.9 min, t_r(major) = 9.7 min). $[\alpha]_D^{23}$ = -15.6 ° (*c* = 1.0, CH_2Cl_2). The configuration of **3zr** was assigned *R* by analogy to (*R*)-**3b**.

^1H NMR (600 MHz, $CDCl_3$) δ 5.98 (s, 1H), 3.89 – 3.77 (m, 2H), 3.35 – 3.26 (m, 1H), 3.02 – 2.95 (m, 1H), 2.53 (s, 3H), 2.22 (s, 3H), 2.21 – 2.14 (m, 2H), 2.01 – 1.90 (m, 3H), 1.76 – 1.69 (m, 2H), 1.61 – 1.55 (m, 2H), 1.50 – 1.38 (m, 2H), 1.33 – 1.25 (m, 7H), 0.89 – 0.84 (m, 3H).

^{13}C NMR (151 MHz, $CDCl_3$) δ 170.73, 152.58, 144.18, 111.51, 59.18, 53.99, 34.25, 31.53,

28.25, 26.30, 25.14, 25.13, 25.11, 25.01, 24.64, 22.32, 14.38, 13.91, 13.77.

IR (film): ν (cm^{-1}) 2933, 2855, 1724, 1586, 1454, 1383, 1298, 1124, 963, 796, 755, 656, 604, 525.

HRMS (ESI, m/z) calcd for $C_{19}H_{32}N_2NaO_3S$ $(M+Na)^+$: 391.2026, found: 391.2030.

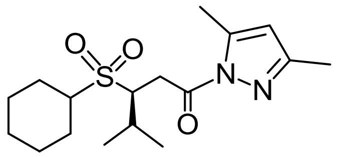

3zs

A dried 25 mL Schlenk tube was charged with **1a** (168.3 mg, 2.0 mmol), **2i** (38.5 mg, 0.20 mmol), **PC1** (3.4 mg, 0.010 mmol), DABCO·$(SO_2)_2$ (36.0 mg, 0.15 mmol), chiral nickel catalyst **[L7-Ni]** (4.0 mL, taken from the above-mentioned freshly prepared solution in DCE), and DCE (4.0 mL). The mixture was degassed *via* three freeze-pump-thaw cycles. The Schlenk tube was positioned approximately 5 cm away from a 24 W blue LEDs lamp (λ_{max} = 455 nm). After being stirred at 0 ℃ for 72 h, the reaction mixture was concentrated, then purified by flash chromatography on silica gel (eluted with CH_2Cl_2) to afford product **3zs** as a colorless oil (44.2 mg, 0.130 mmol, 65% yield). Enantiomeric excess was established by HPLC analysis using a Chiralpak AS-H column, ee = 91% (HPLC: AS-H, 240 nm, *n*-hexane:isopropanol = 90:10, flow rate: 1.0 mL/min, 30 ℃, t_r(minor) = 7.4 min, t_r(major) = 7.8 min). $[\alpha]_D^{23}$ = +6.6 ° (*c* = 1.0, CH_2Cl_2). The configuration of **3zs** was assigned *S* by analogy to (*R*)-**3b**.

^{1}H NMR (600 MHz, $CDCl_3$) δ 5.98 (s, 1H), 3.90 – 3.76 (m, 2H), 3.38 – 3.24 (m, 1H), 3.01 – 2.87 (m, 1H), 2.63 – 2.56 (m, 1H), 2.52 (s, 3H), 2.23 (s, 3H), 2.22 – 2.14 (m, 2H), 1.96 – 1.89 (m, 2H), 1.73 – 1.68 (m, 1H), 1.60 – 1.53 (m, 2H), 1.28 – 1.22 (m, 3H), 1.15 (d, *J* = 6.8 Hz, 3H), 1.04 (d, *J* = 7.0 Hz, 3H).

^{13}C NMR (151 MHz, $CDCl_3$) δ 171.39, 152.62, 144.17, 111.55, 60.50, 58.02, 30.23, 27.06, 25.52, 25.19, 25.16, 25.12, 24.12, 21.35, 17.74, 14.42, 13.79.

IR (film): ν (cm^{-1}) 2930, 2857, 1720, 1585, 1453, 1383, 1310, 1298, 1112, 1029, 956, 896, 790, 744, 624, 504.

HRMS (ESI, m/z) calcd for $C_{17}H_{28}N_2NaO_3S$ $(M+Na)^+$: 363.1713, found: 363.1716.

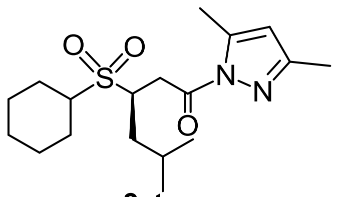

3zt

A dried 25 mL Schlenk tube was charged with **1a** (168.3 mg, 2.0 mmol), **2j** (41.3 mg, 0.20 mmol), **PC1** (3.4 mg, 0.010 mmol), DABCO·$(SO_2)_2$ (36.0 mg, 0.15 mmol), chiral nickel catalyst **[L7-Ni]** (4.0 mL, taken from the above-mentioned freshly prepared solution in DCE), and DCE (4.0 mL). The mixture was degassed *via* three freeze-pump-thaw cycles. The Schlenk tube was positioned approximately 5 cm away from a 24 W blue LEDs lamp (λ_{max} = 455 nm). After being stirred at 0 ℃ for 44 h, the reaction mixture was concentrated, then purified by flash chromatography on silica gel (eluted with CH_2Cl_2) to afford product **3zt** as a colorless oil (37.5 mg, 0.106 mmol, 53% yield). Enantiomeric excess was established by HPLC analysis using a Chiralpak AY-H column, ee = 64% (HPLC: AY-H, 230 nm, *n*-hexane:isopropanol = 90:10, flow rate: 1.5 mL/min, 30 ℃, t_r(minor) = 23.8 min, t_r(major) = 37.2 min). $[\alpha]_D^{23}$ = -6.7 ° (*c* = 1.0, CH_2Cl_2). The configuration of **3zt** was assigned *R* by analogy to (*R*)-**3b**.

^{1}H NMR (600 MHz, $CDCl_3$) δ 5.97 (s, 1H), 3.98 – 3.92 (m, 1H), 3.87 (dd, *J* = 17.8, 6.7 Hz, 1H), 3.21 (dd, *J* = 17.8, 4.9 Hz, 1H), 3.03 – 2.96 (m, 1H), 2.53 (s, 3H), 2.24 – 2.13 (m, 5H), 1.95 – 1.90 (m, 2H), 1.83 – 1.78 (m, 1H), 1.72 – 1.59 (m, 5H), 1.32 – 1.24 (m, 3H), 0.95 (dd, *J* = 10.6, 6.5 Hz, 6H).

^{13}C NMR (151 MHz, $CDCl_3$) δ 170.64, 152.57, 144.21, 111.48, 59.00, 52.33, 37.18, 34.64, 25.65, 25.14, 25.12, 24.92, 24.75, 23.22, 21.43, 14.38, 13.77.

IR (film): ν (cm^{-1}) 2932, 2862, 1726, 1585, 1455, 1384, 1323, 1125, 961, 821, 721, 606, 534.

HRMS (ESI, m/z) calcd for $C_{18}H_{30}N_2NaO_3S$ $(M+Na)^+$: 377.1869, found: 377.1871.

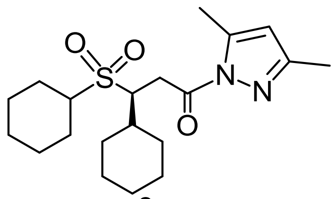

3zu

A dried 25 mL Schlenk tube was charged with **1a** (168.3 mg, 2.0 mmol), **2k** (46.4 mg, 0.20 mmol), **PC1** (3.4 mg, 0.010 mmol), DABCO·$(SO_2)_2$ (36.0 mg, 0.15 mmol), chiral nickel catalyst **[L7-Ni]** (4.0 mL, taken from the above-mentioned freshly prepared solution in DCE), and DCE (4.0 mL). The mixture was degassed *via* three freeze-pump-thaw cycles. The Schlenk tube was positioned approximately 5 cm away from a 24 W blue LEDs lamp (λ_{max} = 455 nm). After being stirred at 0 ℃ for 72 h, the reaction mixture was concentrated, then purified by flash chromatography on silica gel (eluted with CH_2Cl_2) to afford product **3zu** as a colorless oil (43.3 mg, 0.114 mmol, 57% yield). Enantiomeric excess was established by HPLC analysis using a Chiralpak AS-H column, ee = 86% (HPLC: AS-H, 240 nm, *n*-hexane:isopropanol = 90:10, flow rate: 1.0 mL/min, 30 ℃, t_r(minor) = 7.6 min, t_r(major) = 8.0 min). $[\alpha]_D^{23}$ = -11.6 ° (c = 1.0, CH_2Cl_2). The configuration of **3zu** was assigned *S* by analogy to (*R*)-**3b**.

^{1}H NMR (600 MHz, $CDCl_3$) δ 5.98 (s, 1H), 3.90 – 3.77 (m, 2H), 3.36 (dd, *J* = 17.7, 3.6 Hz, 1H), 2.99 – 2.85 (m, 1H), 2.52 (s, 3H), 2.25 – 2.15 (m, 6H), 2.11 – 2.06 (m, 1H), 1.95 – 1.89 (m, 2H), 1.81 – 1.74 (m, 2H), 1.72 – 1.65 (m, 2H), 1.59 – 1.52 (m, 2H), 1.37 – 1.13 (m, 9H).

^{13}C NMR (151 MHz, $CDCl_3$) δ 171.41, 152.59, 144.16, 111.53, 60.58, 58.08, 37.16, 31.46, 31.20, 28.25, 26.65, 25.99, 25.93, 25.61, 25.21, 25.17, 25.13, 24.11, 14.44, 13.81.

IR (film): ν (cm^{-1}) 2923, 2853, 1720, 1583, 1448, 1382, 1306, 1269, 1126, 1078, 1023, 955, 887, 807, 796, 752, 688, 602, 508.

HRMS (ESI, m/z) calcd for $C_{20}H_{32}N_2NaO_3S$ $(M+Na)^+$: 403.2026, found: 403.2030.

3.4 Substrate Scope of α,β-Unsaturated *N*-Acylpyrazoles Containing a β-Aryl Substituent

3.4.1 Preparation of a Solution of the Non-Racemic Nickel Catalyst [L7-Ni] in DCE

According to the published procedure,[7,8] a solution of $Ni(BF_4)_2{\cdot}6H_2O$ (17.0 mg, 0.050 mmol) and non-racemic ligand **L7** (30.7 mg, 0.060 mmol) in dichloroethane (DCE, 4.0 mL) was stirred at 75 °C for 5 h, then used freshly for the catalytic reactions.

3.4.2 General Procedure

A dried 50 mL Schlenk tube was charged with **1a** (2.0 mmol), **2l–2o** (0.60 mmol), **PC1** (3.4 mg, 0.010 mmol), DABCO·$(SO_2)_2$ (24.0 mg, 0.1 mmol), chiral nickel catalyst **[L7-Ni]** (4.0 mL, taken from the above-mentioned freshly prepared solution in DCE), and DCE (12.0 mL). The mixture was degassed *via* three freeze-pump-thaw cycles. The Schlenk tube was positioned approximately 5 cm away from a 24 W blue LEDs lamp (λ_{max} = 455 nm). After being stirred at 0 °C for 72 h (monitored by TLC analysis), the reaction mixture was concentrated, then purified by flash chromatography on silica gel (eluted with CH_2Cl_2 or PE:EtOAc = 4:1) to afford non-racemic product **3zv–3zy**.

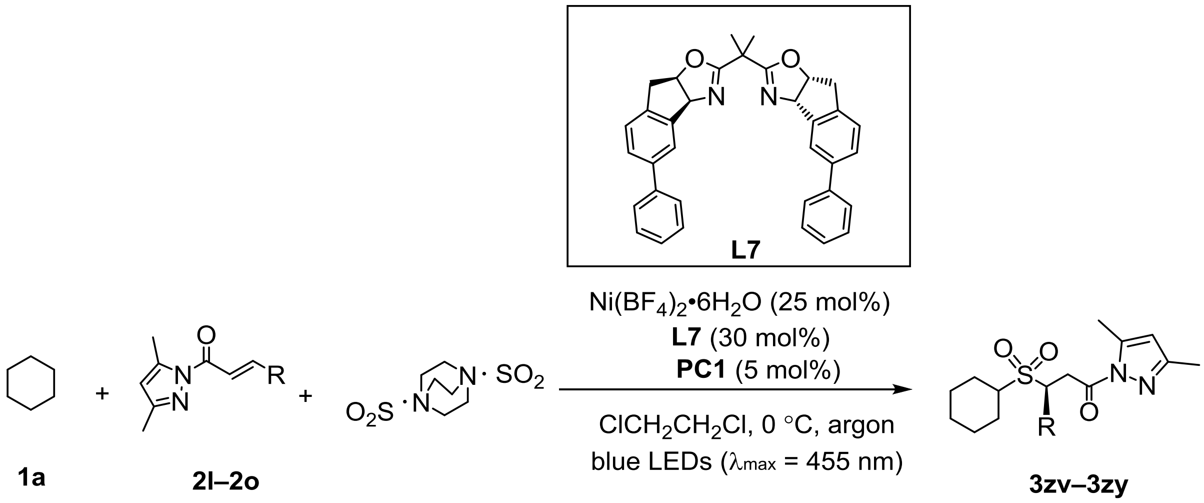

1a **2l–2o** **3zv–3zy**

Supplementary Fig. 1 Nickel-catalyzed photocatalytic asymmetric sulfonylation to produce

the non-racemic products (**3zv–3zy**).

3.4.3 Experimental Details and Characterization Data

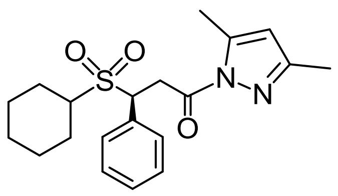

3zv

A dried 50 mL Schlenk tube was charged with **1a** (168.3 mg, 2.0 mmol), **2l** (135.7 mg, 0.60 mmol), **PC1** (3.4 mg, 0.010 mmol), DABCO·$(SO_2)_2$ (24.0 mg, 0.10 mmol), chiral nickel catalyst **[L7-Ni]** (4.0 mL, taken from the above-mentioned freshly prepared solution in DCE), and DCE (12.0 mL). The mixture was degassed *via* three freeze-pump-thaw cycles. The Schlenk tube was positioned approximately 5 cm away from a 24 W blue LEDs lamp (λ_{max} = 455 nm). After being stirred at 0 ℃ for 72 h, the reaction mixture was concentrated, then purified by flash chromatography on silica gel (eluted with PE:EtOAc = 4:1) to afford product **3zv** as a white solid (44.9 mg, 0.120 mmol, 60% yield). Enantiomeric excess was established by HPLC analysis using a Chiralpak IA column, ee = 83% (HPLC: IA, 240 nm, *n*-hexane:isopropanol = 90:10, flow rate: 1.0 mL/min, 30 ℃, t_r(minor) = 10.4 min, t_r(major) = 11.1 min). $[\alpha]_D^{23}$ = +8.2 ° (*c* = 1.0, CH_2Cl_2). The configuration of **3zv** was assigned *S* by analogy to (*R*)-**3b**.

^{1}H NMR (600 MHz, $CDCl_3$) δ 7.59 – 7.48 (m, 2H), 7.44 – 7.32 (m, 3H), 5.91 (s, 1H), 4.96 (dd, *J* = 9.5, 4.0 Hz, 1H), 4.14 (dd, *J* = 18.0, 4.0 Hz, 1H), 4.00 (dd, *J* = 18.0, 9.5 Hz, 1H), 2.68 – 2.57 (m, 1H), 2.42 (s, 3H), 2.22 (s, 3H), 2.18 – 2.12 (m, 1H), 2.05 – 2.01 (m, 1H), 1.88 – 1.82 (m, 2H), 1.66 – 1.49 (m, 3H), 1.26 – 1.12 (m, 3H).

^{13}C NMR (151 MHz, $CDCl_3$) δ 169.83, 152.53, 144.04, 133.05, 129.62, 129.01, 128.95, 111.38, 60.24, 58.05, 34.72, 26.18, 24.99, 24.79, 23.39, 14.21, 13.75.

IR (film): ν (cm^{-1}) 2925, 2855, 1725, 1584, 1452, 1410, 1381, 1307, 1125, 960, 803, 774, 697,

612, 523.

HRMS (ESI, m/z) calcd for $C_{20}H_{26}N_2NaO_3S$ $(M+Na)^+$: 397.1556, found: 397.1561.

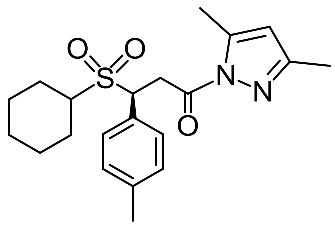

3zw

A dried 50 mL Schlenk tube was charged with **1a** (168.3 mg, 2.0 mmol), **2m** (144.2 mg, 0.60 mmol), **PC1** (3.4 mg, 0.010 mmol), DABCO·$(SO_2)_2$ (24.0 mg, 0.10 mmol), chiral nickel catalyst **[L7-Ni]** (4.0 mL, taken from the above-mentioned freshly prepared solution in DCE), and DCE (12.0 mL). The mixture was degassed *via* three freeze-pump-thaw cycles. The Schlenk tube was positioned approximately 5 cm away from a 24 W blue LEDs lamp (λ_{max} = 455 nm). After being stirred at 0 ℃ for 72 h, the reaction mixture was concentrated, then purified by flash chromatography on silica gel (eluted with PE:EtOAc = 4:1) to afford product **3zw** as a white solid (44.3 mg, 0.114 mmol, 57% yield). Enantiomeric excess was established by HPLC analysis using a Chiralpak IA column, ee = 78% (HPLC: IA, 240 nm, *n*-hexane:isopropanol = 90:10, flow rate: 1.0 mL/min, 30 ℃, t_r(minor) = 10.4 min, t_r(major) = 12.5 min). $[\alpha]_D^{23}$ = -5.7 ° (*c* = 1.0, CH_2Cl_2). The configuration of **3zw** was assigned *S* by analogy to (*R*)-**3b**.

^{1}H NMR (600 MHz, $CDCl_3$) δ 7.43 – 7.39 (m, 2H), 7.20 – 7.16 (m, 2H), 5.91 (s, 1H), 4.93 (dd, *J* = 9.6, 3.9 Hz, 1H), 4.15 – 4.08 (m, 1H), 3.98 (dd, *J* = 18.0, 9.6 Hz, 1H), 2.69 – 2.60 (m, 1H), 2.42 (s, 3H), 2.34 (s, 3H), 2.22 (s, 3H), 2.18 – 2.13 (m, 1H), 2.06 – 2.02 (m, 1H), 1.87 – 1.82 (m, 2H), 1.64 – 1.47 (m, 3H), 1.24 – 1.08 (m, 3H).

^{13}C NMR (151 MHz, $CDCl_3$) δ 169.94, 152.47, 144.02, 138.95, 129.92, 129.68, 129.45, 111.35, 59.93, 57.80, 34.66, 26.22, 25.03, 24.99, 24.79, 21.21, 14.24, 13.77.

IR (film): ν (cm^{-1}) 2931, 2859, 1726, 1586, 1453, 1410, 1383, 1308, 1131, 982, 964, 874, 760, 632, 585.

HRMS (ESI-TOF, m/z) calcd for $C_{21}H_{28}N_2NaO_3S$ $(M+Na)^+$: 411.1713, found: 411.1695.

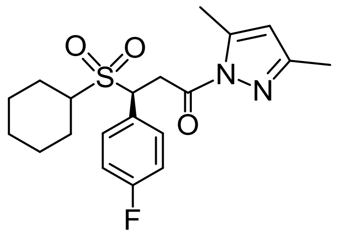

3zx

A dried 50 mL Schlenk tube was charged with **1a** (168.3 mg, 2.0 mmol), **2n** (146.6 mg, 0.60 mmol), **PC1** (3.4 mg, 0.010 mmol), DABCO·$(SO_2)_2$ (24.0 mg, 0.10 mmol), chiral nickel catalyst **[L7-Ni]** (4.0 mL, taken from the above-mentioned freshly prepared solution in DCE), and DCE (12.0 mL). The mixture was degassed *via* three freeze-pump-thaw cycles. The Schlenk tube was positioned approximately 5 cm away from a 24 W blue LEDs lamp (λ_{max} = 455 nm). After being stirred at 0 ℃ for 72 h, the reaction mixture was concentrated, then purified by flash chromatography on silica gel (eluted with PE:EtOAc = 4:1) to afford product **3zx** as a yellow solid (39.9 mg, 0.102 mmol, 51% yield). Enantiomeric excess was established by HPLC analysis using a Chiralpak AD-H column, ee = 83% (HPLC: AD-H, 240 nm, *n*-hexane:isopropanol = 90:10, flow rate: 1.0 mL/min, 30 ℃, t_r(minor) = 19.2 min, t_r(major) = 16.5 min). $[\alpha]_D^{23}$ = +8.2 ° (*c* = 1.0, CH_2Cl_2). The configuration of **3zx** was assigned *S* by analogy to (*R*)-**3b**.

^{1}H NMR (600 MHz, $CDCl_3$) δ 7.55 – 7.49 (m, 2H), 7.10 – 7.04 (m, 2H), 5.92 (s, 1H), 4.94 (dd, *J* = 9.8, 3.9 Hz, 1H), 4.09 (dd, *J* = 17.9, 3.9 Hz, 1H), 3.97 (dd, *J* = 17.9, 9.8 Hz, 1H), 2.68 – 2.60 (m, 1H), 2.42 (s, 3H), 2.22 (s, 3H), 2.15 – 2.10 (m, 1H), 2.07 – 2.04 (m, 1H), 1.89 – 1.84 (m, 2H), 1.68 – 1.49 (m, 3H), 1.29 – 1.08 (m, 3H).

^{13}C NMR (151 MHz, $CDCl_3$) δ 169.90, 164.04, 162.39, 152.87, 144.28, 131.65 (d, *J* = 8.3 Hz), 128.98 (d, *J* = 3.3 Hz), 116.34, 116.19, 111.70, 59.64, 58.37, 35.11, 26.28, 25.20, 25.03, 23.73, 14.42, 13.97.

IR (film): ν (cm^{-1}) 2933, 2855, 1728, 1606, 1510, 1454, 1384, 1309, 1229, 1130, 982, 963, 843, 816, 586.

HRMS (ESI-TOF, m/z) calcd for $C_{20}H_{25}FN_2NaO_3S$ $(M+Na)^+$: 415.1462, found: 415.1500.

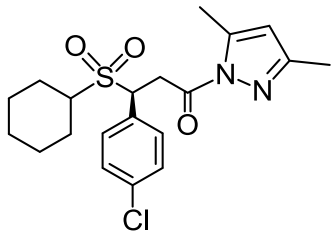

3zy

A dried 50 mL Schlenk tube was charged with **1a** (168.3 mg, 2.0 mmol), **2o** (156.4 mg, 0.60 mmol), **PC1** (3.4 mg, 0.010 mmol), DABCO·$(SO_2)_2$ (24.0 mg, 0.10 mmol), chiral nickel catalyst **[L7-Ni]** (4.0 mL, taken from the above-mentioned freshly prepared solution in DCE), and DCE (12.0 mL). The mixture was degassed *via* three freeze-pump-thaw cycles. The Schlenk tube was positioned approximately 5 cm away from a 24 W blue LEDs lamp (λ_{max} = 455 nm). After being stirred at 0 ℃ for 72 h, the reaction mixture was concentrated, then purified by flash chromatography on silica gel (eluted with PE:EtOAc = 4:1) to afford product **3zy** as a white solid (42.5 mg, 0.104 mmol, 52% yield). Enantiomeric excess was established by HPLC analysis using a Chiralpak IE column, ee = 80% (HPLC: IE, 240 nm, *n*-hexane:isopropanol = 90:10, flow rate: 1.0 mL/min, 30 ℃, t_r(minor) = 40.5 min, t_r(major) = 44.6 min). $[\alpha]_D^{23}$ = +6.4 ° (*c* = 1.0, CH_2Cl_2). The configuration of **3zy** was assigned *S* by analogy to (*R*)-**3b**.

^{1}H NMR (600 MHz, $CDCl_3$) δ 7.51 – 7.45 (m, 2H), 7.39 – 7.33 (m, 2H), 5.92 (s, 1H), 4.92 (dd, *J* = 9.8, 3.9 Hz, 1H), 4.09 (dd, *J* = 17.9, 3.9 Hz, 1H), 3.97 (dd, *J* = 17.9, 9.8 Hz, 1H), 2.69 – 2.60 (m, 1H), 2.42 (s, 3H), 2.22 (s, 3H), 2.15 – 2.09 (m, 1H), 2.08 – 2.03 (m, 1H), 1.89 – 1.84 (m, 2H), 1.63 – 1.51 (m, 3H), 1.24 – 1.12 (m, 3H).

^{13}C NMR (151 MHz, $CDCl_3$) δ 169.83, 152.91, 144.28, 135.34, 131.74, 131.19, 129.43, 111.73, 59.71, 58.42, 34.99, 26.31, 25.19, 25.16, 25.00, 23.69, 14.42, 13.97.

IR (film): ν (cm^{-1}) 2935, 2857, 1726, 1587, 1491, 1453, 1384, 1308, 1131, 1094, 1015, 962, 889, 703, 648, 561.

HRMS (ESI-TOF, m/z) calcd for $C_{20}H_{25}ClN_2NaO_3S$ $(M+Na)^+$: 431.1167, found: 431.1185.

3.5 A Scale-up Catalytic Reaction

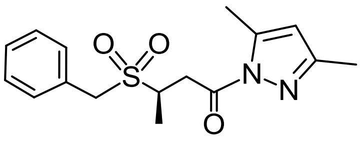

3v

A dried 50 mL Schlenk tube was charged with **4a** (460.7 mg, 5.0 mmol), **2a** (164.0 mg, 1.00 mmol), **PC1** (17.0 mg, 0.050 mmol), DABCO·$(SO_2)_2$ (180.0 mg, 0.75 mmol), chiral nickel catalyst **[L7-Ni]** (20.0 mL, taken from the above-mentioned freshly prepared solution in DCE), and DCE (10.0 mL). The mixture was degassed *via* three freeze-pump-thaw cycles. The Schlenk tube was positioned approximately 5 cm away from a 24 W blue LEDs lamp (λ_{max} = 455 nm). After being stirred at 0 ℃ for 45 h, the reaction mixture was concentrated, then purified by flash chromatography on silica gel (eluted with PE:EtOAc = 4:1) to afford product **3v** as a yellow oil (227.0 mg, 0.710 mmol, 71% yield). Enantiomeric excess was established by HPLC analysis using a Chiralpak AD-H column, ee = 91% (HPLC: AD-H, 220 nm, *n*-hexane:isopropanol = 90:10, flow rate: 1.0 mL/min, 30 ℃, t_r(minor) = 18.5 min, t_r(major) = 21.6 min).

3.6 Absolute Configuration Assignment of the Chiral Products

Absolute configuration of the major enantiomer of product **3b** was assigned as *R* by its crystal structure (CCDC no. 2028396). The configuration of **3zs**, **3zu–3zy** was assigned *S* by analogy to (*R*)-**3b** and **3a–3zo, 3zp–3zr, 3zt, 5, 7–12** in the photocatalytic reaction were accordingly assigned as *R* by analogy.

3.7 Set-up of the Photoreactions and Emission Spectra of the Light Source

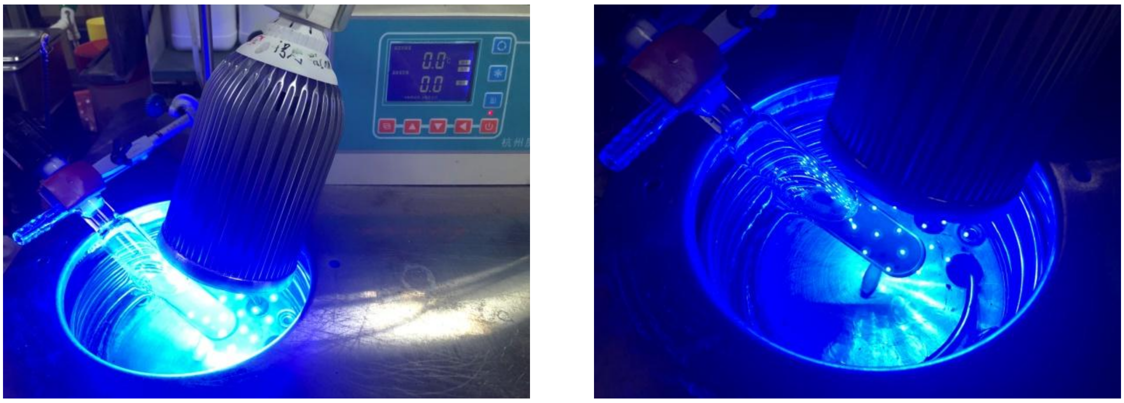

Supplementary Fig. 2 Reaction set-up of the photochemical reaction in a constant low temperature box.

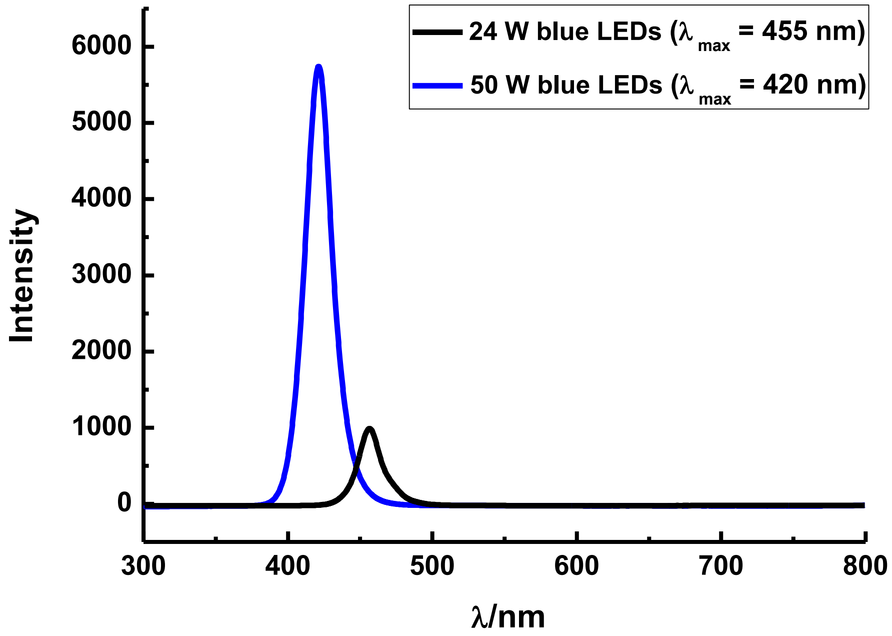

Supplementary Fig. 3 Emission spectra of of 24 W blue LEDs lamp (maxium emission at λ = 455 nm) and 50 W blue LEDs lamp (maxium emission at λ = 420 nm).

4. Synthetic Transformations

4.1 Transformation of Product 3v to an Alcohol Derivative

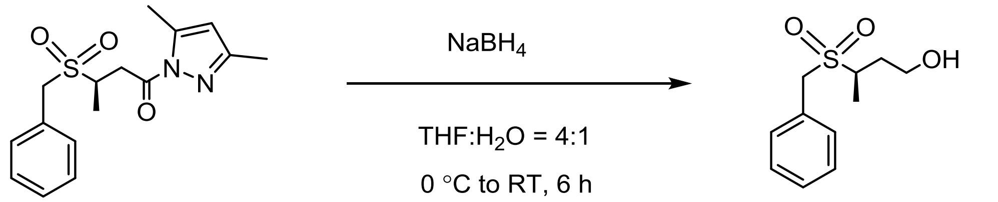

3v 91% ee

7 93% yield, 91% ee

To a solution of **3v** (32.0 mg, 0.10 mmol) in THF:H_2O (v:v = 4:1, 1.0 mL) at 0 ℃ was added $NaBH_4$ (37.3 mg, 1.0 mmol). The mixture was stirred at room temperature for 6 h, then quenched with aqueous HCl (2.0 M) and extracted with dichloromethane. The combined organic layers were dried over anhydrous Na_2SO_4, filtered, and concentrated to dryness. The residue was then purified by flash chromatography on silica gel (eluted with PE:EtOAc = 1:1) to afford product **7** as a white solid (21.2 mg, 0.093 mmol, 93% yield). Enantiomeric excess was established by HPLC analysis using a Chiralpak AY-H column, ee = 91% (HPLC: AY-H, 220 nm, *n*-hexane:isopropanol = 80:20, flow rate: 1 mL/min, 30 ℃, t_r(minor) = 18.3 min, t_r(major) = 21.2 min). $[\alpha]_D^{23}$ = -8.3 °(*c* = 1.0, CH_2Cl_2). The configuration of **7** was assigned *R* by analogy to (*R*)-**3b.**

^{1}H NMR (600 MHz, $CDCl_3$) δ 7.45 – 7.38 (m, 5H), 4.25 (d, *J* = 1.1 Hz, 2H), 3.90 – 3.80 (m, 1H), 3.75 – 3.61 (m, 1H), 3.23 – 3.14 (m, 1H), 2.32 – 2.20 (m, 1H), 1.87 – 1.73 (m, 2H), 1.41 (d, *J* = 6.9 Hz, 3H).

^{13}C NMR (151 MHz, $CDCl_3$) δ 130.74, 129.03, 129.02, 127.58, 59.14, 56.56, 53.15, 31.98, 13.47.

IR (film): ν (cm^{-1}) 2917, 2850, 1708, 1639, 1376, 1116, 1054, 915, 844, 789, 737, 698, 590, 537.

HRMS (ESI, m/z) calcd for $C_{11}H_{16}NaO_3S$ $(M+Na)^+$: 251.0712, found: 251.0716.

4.2 Transformation of Product 3v to an Ester Derivative

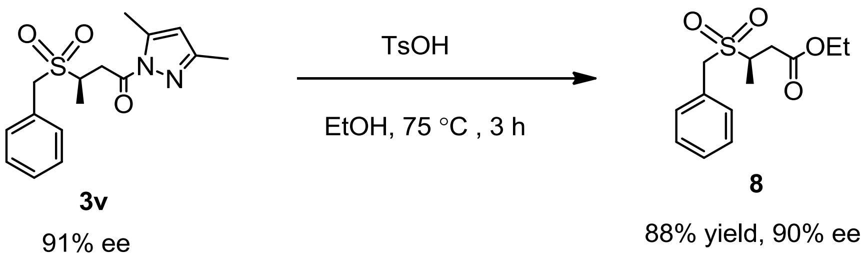

3v
91% ee

8
88% yield, 90% ee

To a solution of **3v** (32.0 mg, 0.10 mmol) in ethanol (0.50 mL) was added *p*-toluenesulfonic acid (206.6 mg, 1.2 mmol). The reaction mixture was heated to 75 ℃ for 3 h, then cooled down to room temperature and concentrated to dryness. The residue was purified by flash chromatography on silica gel (eluted with PE:EtOAc = 5:1) to afford product **8** as a white solid (23.8 mg, 0.088 mmol, 88% yield). Enantiomeric excess was established by HPLC analysis using a Chiralpak AD-H column, ee = 90% (HPLC: AD-H, 220 nm, *n*-hexane:isopropanol = 90:10, flow rate: 1.0 mL/min, 30 ℃, t_r(minor) = 15.5 min, t_r(major) = 19.7 min). $[\alpha]_D^{23}$ = -6.3 ° (*c* = 1.0, CH_2Cl_2). The configuration of **8** was assigned *R* by analogy to (*R*)-**3b.**

^1H NMR (600 MHz, $CDCl_3$) δ 7.45 – 7.37 (m, 5H), 4.25 (s, 2H), 4.19 – 4.12 (m, 2H), 3.51 – 3.39 (m, 1H), 3.00 (dd, *J* = 16.6, 4.2 Hz, 1H), 2.45 (dd, *J* = 16.6, 9.5 Hz, 1H), 1.41 (d, *J* = 6.9 Hz, 3H), 1.25 (t, *J* = 14.3 Hz, 3H).

^{13}C NMR (151 MHz, $CDCl_3$) δ 170.18, 130.71, 129.14, 129.10, 127.35, 61.34, 57.10, 52.61, 34.04, 14.16, 14.13.

IR (film): ν (cm^{-1}) 2923, 1733, 1498, 1456, 1373, 1307, 1183, 1117, 1030, 872, 777, 696, 586, 507.

HRMS (ESI, m/z) calcd for $C_{13}H_{18}NaO_4S$ $(M+Na)^+$: 293.0818, found: 293.0822.

4.3 Transformation of Product 3v to an Amide Derivative

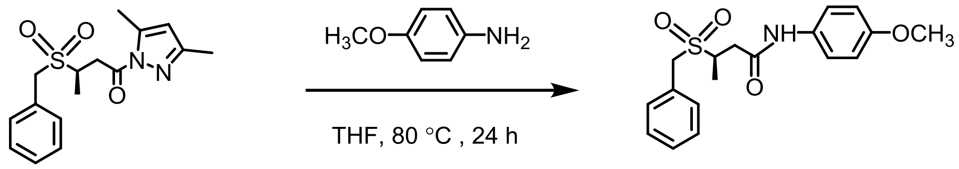

To a solution of **3v** (32.0 mg, 0.10 mmol) in THF (0.50 mL) was added *p*-anisidine (107.2 mg, 1.00 mmol). The reaction mixture was stirred at 80 ℃ for 24 h, then cooled down to room temperature and concentrated to dryness. The residue was purified by flash silica gel column chromatography (eluted with PE:EtOAc = 2:1) to afford **9** as a white solid (31.6 mg, 0.091 mmol, 91% yield). Enantiomeric excess was established by HPLC analysis using a Chiralpak AD-H column, ee = 90% (HPLC: AD-H, 240 nm, *n*-hexane:isopropanol = 70:30, flow rate: 1.0 mL/min, 30 ℃, t_r(minor) = 14.8 min, t_r(major) = 18.2 min). $[\alpha]_D^{23}$ = -8.6 ° (*c* = 1.0, CH_2Cl_2). The configuration of **9** was assigned *R* by analogy to (*R*)-**3b.**

^{1}H NMR (600 MHz, $CDCl_3$) δ 7.79 (s, 1H), 7.44 – 7.40 (m, 2H), 7.40 – 7.36 (m, 3H), 7.36 – 7.31 (m, 2H), 6.84 – 6.78 (m, 2H), 4.32 – 4.25 (m, 2H), 3.78 (s, 3H), 3.71 – 3.65 (m, 1H), 3.04 (dd, *J* = 15.4, 4.9 Hz, 1H), 2.54 – 2.47 (m, 1H), 1.47 (d, *J* = 6.9 Hz, 3H).

^{13}C NMR (151 MHz, $CDCl_3$) δ 166.90, 156.57, 130.85, 130.67, 129.20, 129.11, 127.03, 121.78, 114.13, 57.46, 55.49, 53.13, 36.35, 14.34.

IR (film): ν (cm^{-1}) 2974, 2835, 1657, 1545, 1512, 1455, 1299, 1247, 1126, 1032, 826, 768, 697, 599, 520.

HRMS (ESI, m/z) calcd for $C_{18}H_{21}NNaO_4S$ $(M+Na)^+$: 370.1083, found: 370.1092.

4.4 Late-Stage Modification of Bioactive Molecules

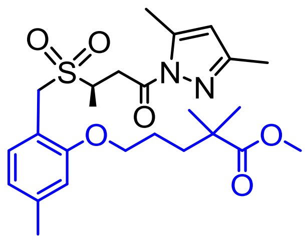

10

A dried 25 mL Schlenk tube was charged with the lopid derivative (158.6 mg, 0.6 mmol), **2a** (32.8 mg, 0.20 mmol), **PC1** (3.4 mg, 0.010 mmol), DABCO·$(SO_2)_2$ (36.0 mg, 0.15 mmol), chiral nickel catalyst **[L7-Ni]** (4.0 mL, taken from the above-mentioned freshly prepared solution in DCE), and DCE (4.0 mL). The mixture was degassed *via* three freeze-pump-thaw cycles. The Schlenk tube was positioned approximately 5 cm away from a 24 W blue LEDs lamp (λ_{max} = 455 nm). After being stirred at 0 ℃ for 80 h, the reaction mixture was concentrated, then purified by flash chromatography on silica gel (eluted with PE:EtOAc = 4:1) to afford product **10** as a yellow oil (63.1 mg, 0.128 mmol, 64% yield). Enantiomeric excess was established by HPLC analysis using a Chiralpak AD-H column, ee = 86% (HPLC: AD-H, 220 nm, *n*-hexane:isopropanol = 90:10, flow rate: 1.0 mL/min, 30 ℃, t_r(minor) = 25.6 min, t_r(major) = 18.6 min). $[\alpha]_D^{23}$ = +5.2 ° (*c* = 1.0, CH_2Cl_2). The configuration of **10** was assigned *R* by analogy to (*R*)-**3b.**

^{1}H NMR (600 MHz, $CDCl_3$) δ 7.35 (d, *J* = 7.7 Hz, 1H), 6.79 (d, *J* = 5.3 Hz, 1H), 6.68 (s, 1H), 5.95 (s, 1H), 4.45 – 4.33 (m, 2H), 3.98 – 3.88 (m, 2H), 3.72 (dd, *J* = 17.7, 3.1 Hz, 1H), 3.65 (s, 3H), 3.64 – 3.58 (m, 1H), 3.39 (dd, *J* = 17.7, 10.1 Hz, 1H), 2.49 (s, 3H), 2.33 (s, 3H), 2.19 (s, 3H), 1.69 – 1.65 (m, 4H), 1.42 (d, *J* = 6.8 Hz, 3H), 1.17 (s, 6H).

^{13}C NMR (151 MHz, $CDCl_3$) δ 178.12, 170.35, 156.59, 152.48, 143.93, 140.76, 132.31, 121.82, 113.34, 112.51, 111.45, 68.48, 52.06, 51.81, 50.79, 42.03, 36.89, 35.47, 25.17, 25.07, 24.88, 21.74, 14.34, 13.89, 13.75.

IR (film): ν (cm^{-1}) 2930, 2877, 1728, 1615, 1584, 1509, 1386, 1326, 1268, 1126, 1043, 987,

963, 870, 758, 645, 594,515.

HRMS (ESI, m/z) calcd for $C_{25}H_{36}N_2NaO_6S$ $(M+Na)^+$: 515.2186, found: 515.2200.

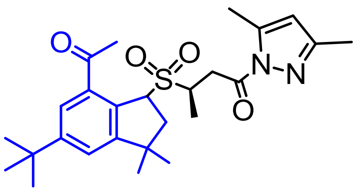

11

A dried 25 mL Schlenk tube was charged with celestolide (146.6 mg, 0.6 mmol), **2a** (32.8 mg, 0.20 mmol), **PC1** (3.4 mg, 0.010 mmol), DABCO·$(SO_2)_2$ (36.0 mg, 0.15 mmol), chiral nickel catalyst **[L7-Ni]** (4.0 mL, taken from the above-mentioned freshly prepared solution in DCE), and DCE (4.0 mL). The mixture was degassed *via* three freeze-pump-thaw cycles. The Schlenk tube was positioned approximately 5 cm away from a 24 W blue LEDs lamp (λ_{max} = 455 nm). After being stirred at 0 ℃ for 72 h, the reaction mixture was concentrated, the diastereomeric ratio was determined as 1:1 dr by ^{1}H NMR analysis of the crude product. The residue was purified by flash chromatography on silica gel (eluted with CH_2Cl_2:EtOAc = 40:1). The less polar diastereomer of **11** was collected as a white solid (26.5 mg, 0.056 mmol, 28% yield), and the more polar diastereomer of **11** was collected as a colorless oil (28.4 mg, 0.060 mmol, 30% yield).

Analytic data of the less polar diastereomer: Enantiomeric excess was established by HPLC analysis using a Chiralpak AD-H column, ee = 81% (HPLC: AD-H, 220 nm, *n*-hexane:isopropanol = 90:10, flow rate: 1.0 mL/min, 30 ℃, t_r(minor) = 10.6 min, t_r(major) = 13.3 min). $[\alpha]_D^{23}$ = +4.8 ° (*c* = 1.0, CH_2Cl_2). The configuration of this diastereomer was assigned *R* by analogy to (*R*)-**3b.**

^{1}H NMR (600 MHz, $CDCl_3$) δ 7.58 (d, *J* = 1.8 Hz, 1H), 7.31 (d, *J* = 1.8 Hz, 1H), 5.97 (s, 1H), 5.69 (dd, *J* = 9.8, 1.7 Hz, 1H), 3.93 – 3.82 (m, 2H), 3.34 – 3.26 (m, 1H), 2.73 – 2.66 (m, 1H), 2.63 (s, 3H), 2.54 (s, 3H), 2.41 (dd, *J* = 14.3, 9.8 Hz, 1H), 2.23 (s, 3H), 1.51 – 1.48 (m, 3H), 1.45 (s, 3H), 1.36 – 1.34 (m, 12H).

^{13}C NMR (151 MHz, $CDCl_3$) δ 202.69, 170.46, 155.99, 153.17, 152.52, 144.16, 137.95, 127.91, 124.34, 122.84, 111.48, 62.69, 54.61, 43.92, 42.71, 36.15, 34.98, 31.47, 31.39, 30.46, 28.24, 14.41, 13.86, 13.56.

IR (film): ν (cm^{-1}) 2925, 2847, 1729, 1690, 1647, 1386, 1330, 1302, 1236, 1140, 961, 879, 804, 677, 604, 512.

HRMS (ESI, m/z) calcd for $C_{26}H_{36}N_2NaO_4S$ $(M+Na)^+$: 495.2288, found: 495.2299.

Analytic data of the more polar diastereomer: Enantiomeric excess was established by HPLC analysis using a Chiralpak AZ-H column, ee = 88% (HPLC: AZ-H, 220 nm, *n*-hexane:isopropanol = 90:10, flow rate: 1.0 mL/min, 30 ℃, t_r(minor) = 15.7 min, t_r(major) = 12.4 min). $[\alpha]_D^{23}$ = -5.9 ° (*c* = 1.0, CH_2Cl_2). The configuration of this diastereomer was assigned *R* by analogy to (*R*)-**3b.**

^1H NMR (600 MHz, $CDCl_3$) δ 7.58 (d, *J* = 1.8 Hz, 1H), 7.31 (d, *J* = 1.8 Hz, 1H), 5.97 (d, *J* = 1.3 Hz, 1H), 5.69 (dd, *J* = 9.8, 1.7 Hz, 1H), 3.93 – 3.83 (m, 2H), 3.34 – 3.26 (m, 1H), 2.67 (dd, *J* = 14.4, 1.8 Hz, 1H), 2.63 (s, 3H), 2.54 (s, 3H), 2.38 (dd, *J* = 14.3, 9.8 Hz, 1H), 2.21 (s, 3H), 1.51 (d, *J* = 6.9 Hz, 3H), 1.44 (s, 3H), 1.36 – 1.34 (m, 12H).

^{13}C NMR (151 MHz, $CDCl_3$) δ 202.64, 170.54, 156.07, 153.19, 152.56, 144.11, 138.06, 127.61, 124.29, 122.84, 111.49, 62.53, 53.90, 44.00, 42.40, 35.44, 34.98, 31.65, 31.39, 30.38, 28.28, 14.40, 14.12, 13.80.

IR (film): ν (cm^{-1}) 2963, 2867, 1726, 1688, 1585, 1465, 1385, 1232, 1138, 1026, 965, 807, 655, 590, 518.

HRMS (ESI, m/z) calcd for $C_{26}H_{36}N_2NaO_4S$ $(M+Na)^+$: 495.2288, found: 495.2303.

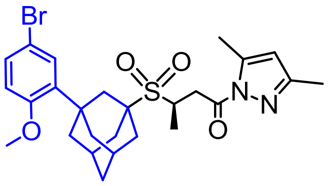

12

A dried 25 mL Schlenk tube was charged with **S14** (321.3 mg, 1.0 mmol), **2a** (32.8 mg, 0.20 mmol), **PC1** (3.4 mg, 0.010 mmol), DABCO·$(SO_2)_2$ (36.0 mg, 0.15 mmol), chiral nickel catalyst **[L7-Ni]** (4.0 mL, taken from the above-mentioned freshly prepared solution in DCE), and DCE (4.0 mL). The mixture was degassed *via* three freeze-pump-thaw cycles. The Schlenk tube was positioned approximately 5 cm away from a 50 W blue LEDs lamp (λ_{max} = 420 nm). After being stirred at -10 ℃ for 80 h, the reaction mixture was concentrated, then purified by flash chromatography on silica gel (eluted with CH_2Cl_2:EtOAc = 20:1) to afford product **12** as a white solid (57.0 mg, 0.104 mmol, 52% yield). Enantiomeric excess was established by HPLC analysis using a Chiralpak AD-H column, ee = 85% (HPLC: AD-H, 220 nm, *n*-hexane:isopropanol = 90:10, flow rate: 1.0 mL/min, 30 ℃, t_r(minor) = 23.3 min, t_r(major) = 32.3 min). $[\alpha]_D^{23}$ = +5.7 °(*c* = 1.0, CH_2Cl_2). The configuration of **12** was assigned *R* by analogy to (*R*)-**3b.**

^{1}H NMR (600 MHz, $CDCl_3$) δ 7.32 – 7.24 (m, 2H), 6.75 (d, *J* = 8.5 Hz, 1H), 5.96 (s, 1H), 4.16 – 4.07 (m, 1H), 3.97 (dd, *J* = 17.5, 5.4 Hz, 1H), 3.81 (s, 3H), 3.17 (dd, *J* = 17.4, 7.7 Hz, 1H), 2.53 (s, 3H), 2.47 – 2.39 (m, 2H), 2.38 – 2.35 (m, 2H), 2.19 (s, 3H), 2.16 – 2.09 (m, 6H), 2.02 – 1.97 (m, 2H), 1.75 (s, 2H), 1.46 (d, *J* = 6.9 Hz, 3H).

^{13}C NMR (151 MHz, $CDCl_3$) δ 170.63, 157.57, 152.46, 144.09, 138.00, 130.11, 129.58, 113.37, 113.29, 111.42, 63.54, 55.28, 47.79, 38.93, 38.90, 38.04, 37.28, 37.24, 35.24, 34.58, 28.92, 16.26, 14.43, 13.74.

^{13}C NMR-dept 135 (151 MHz, $CDCl_3$) δ 130.14, 129.62, 113.41, 111.46, 55.31, 47.83, 38.97, 38.94, 37.31, 37.27, 35.27, 34.62, 28.95, 16.30, 14.46, 13.78.

IR (film): ν (cm^{-1}) 2917, 2855, 1725, 1484, 1387, 1281, 1138, 1036, 1021, 812, 758, 655, 620, 590, 513.

HRMS (ESI, m/z) calcd for $C_{26}H_{33}BrN_2NaO_4S$ $(M+Na)^+$: 571.1237, found: 571.1256.

5. Mechanistic Investigations

5.1 Radical Trapping Experiments

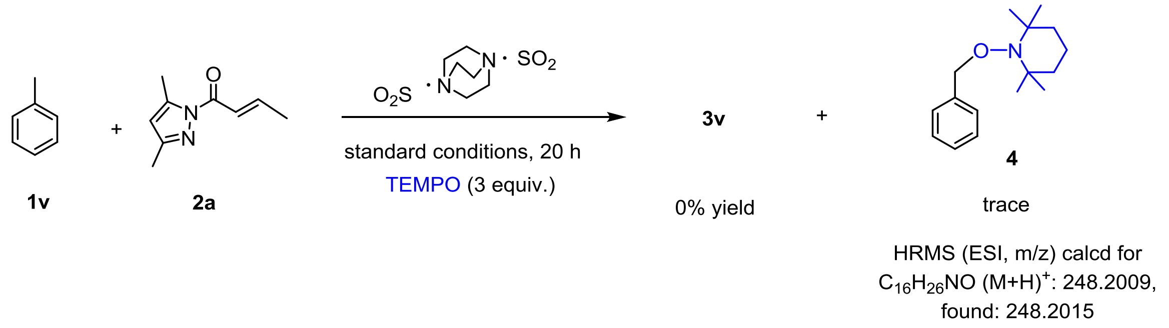

A dried 25 mL Schlenk tube was charged with **1v** (184.3 mg, 2.0 mmol), **2a** (32.8 mg, 0.20 mmol), **PC1** (3.4 mg, 0.010 mmol), DABCO·$(SO_2)_2$ (36.0 mg, 0.15 mmol), TEMPO (2,2,6,6-tetramethylpiperidine-1-oxyl, 93.7 mg, 0.60 mmol), chiral nickel catalyst **[L7-Ni]** (4.0 mL, taken from the above-mentioned freshly prepared solution in DCE), and DCE (4.0 mL). The mixture was degassed *via* three freeze-pump-thaw cycles. The Schlenk tube was positioned approximately 5 cm away from a 24 W blue LEDs lamp (λ_{max} = 455 nm). After being stirred at 0 ℃ for 20 h, the resulting solution was concentrated to dryness. The residue was analysis by ^{1}H-NMR and HRMS. As a result, compound **3v** was not detected, while product **4** was observed by HRMS. HRMS (ESI, m/z) calcd for $C_{16}H_{26}NO$ $(M+H)^+$: 248.2009, found: 248.2015.

Display Report

Analysis Info

Analysis Name	D:\Data\origin data\gonglei\yuying\20200727\yy-2-142-5_000013.d	Acquisition Date	8/1/2020 9:37:22 AM
Method	APEX_Pos_NaFA_400_20100407	Operator	
Sample Name	yy-2-142-5	Instrument	apex-Ultra
Comment			

Acquisition Parameter

Polarity	Positive	Source	ESI	No. of Laser Shots	20
Averaged Scans	4	No. of Cell Fills	1	Laser Power	51.0 %
Broadband Low Mass	100.4 m/z	End Plate	3900.0 V	MALDI Plate	300.0 V
Broadband High Mass	3000.0 m/z	Capillary Entrance	4400.0 V	Imaging Spot Diameter	2000.0 µm
Acquisition Mode	Single MS	Skimmer 1	36.0 V		
Pulse Program	basic	Drying Gas Temperature	200.0 °C	Calibration Date	Thu Feb 21 03:16:44 2019
Source Accumulation	0.0 sec	Drying Gas Flow Rate	4.0 L/min	Data Acquisition Size	1048576
Ion Accumulation Time	0.8 sec	Nebulizer Gas Flow Rate	1.0 L/min	Apodization	Sine-Bell Multiplication
Flight Time to Acq. Cell	0.0 sec				

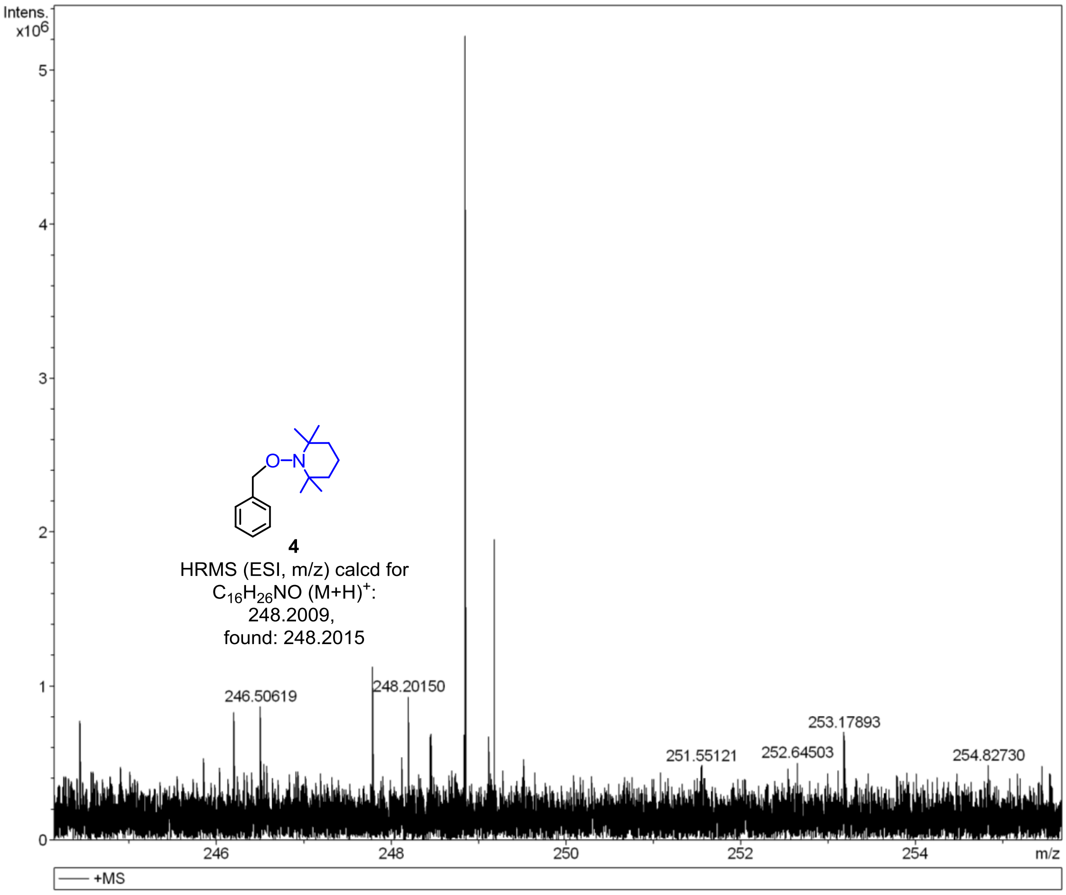

Bruker Compass DataAnalysis 4.0 printed: 8/1/2020 9:40:57 AM Page 1 of 2

Supplementary Fig. 4 HRMS analysis of the control experiment.

5.2 Radical Clock Experiments

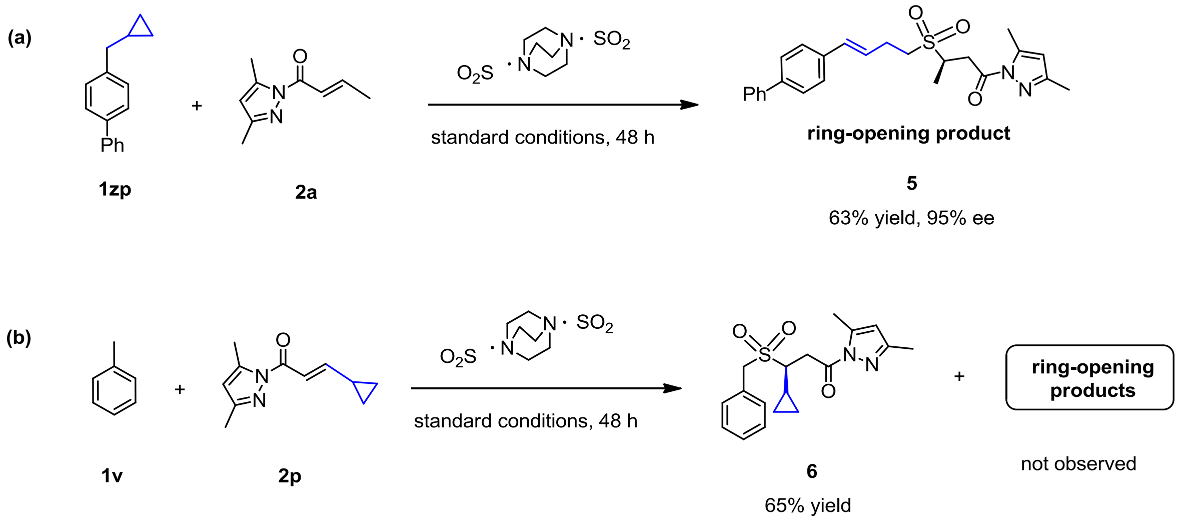

Procedure for radical clock experiment (a): A dried 25 mL Schlenk tube was charged with **1zp** (416.6 mg, 2.0 mmol), **2a** (32.8 mg, 0.20 mmol), **PC1** (3.4 mg, 0.010 mmol), DABCO·$(SO_2)_2$ (36.0 mg, 0.15 mmol), chiral nickel catalyst **[L7-Ni]** (4.0 mL, taken from the above-mentioned freshly prepared solution in DCE), and DCE (4.0 mL). The mixture was degassed *via* three freeze-pump-thaw cycles. The Schlenk tube was positioned approximately 5 cm away from a 24 W blue LEDs lamp (λ_{max} = 455 nm). After being stirred at 0 ℃ for 48 h, the reaction mixture was concentrated, then purified by flash chromatography on silica gel (eluted with CH_2Cl_2) to afford product **5** as a white solid (55.0 mg, 0.126 mmol, 63% yield). Enantiomeric excess was established by HPLC analysis using a Chiralpak IA column, ee = 95% (HPLC: IA, 240 nm, *n*-hexane:isopropanol = 90:10, flow rate: 1 mL/min, 30 ℃, t_r(minor) = 25.1 min, t_r(major) = 32.8 min). $[\alpha]_D^{23}$ = +23.1 °(*c* = 1.0, CH_2Cl_2).

^{1}H NMR (600 MHz, $CDCl_3$) δ 7.57 (dd, *J* = 24.5, 7.6 Hz, 4H), 7.42 (dd, *J* = 9.7, 8.0 Hz, 4H), 7.37 – 7.32 (m, 1H), 6.59 – 6.54 (m, 1H), 6.29 – 6.21 (m, 1H), 5.99 (s, 1H), 3.87 (dd, *J* = 17.4, 4.3 Hz, 1H), 3.81 – 3.73 (m, 1H), 3.35 (dd, *J* = 17.4, 8.7 Hz, 1H), 3.25 – 3.13 (m, 2H), 2.90 – 2.78 (m, 2H), 2.53 (s, 3H), 2.23 (s, 3H), 1.53 (d, *J* = 6.9 Hz, 3H).

^{13}C NMR (151 MHz, $CDCl_3$) δ 169.81, 152.55, 144.00, 140.37, 140.14, 132.04, 128.55, 127.10, 127.04, 126.67, 126.37, 125.25, 111.41, 53.53, 49.49, 35.62, 24.85, 14.12, 13.66,

13.56.

IR (film): ν (cm^{-1}) 2917, 2845, 1725, 1586, 1487, 1410, 1386, 1326, 1312, 1127, 963, 870, 760, 655, 561.

HRMS (ESI-TOF, m/z) calcd for $C_{25}H_{29}N_2O_3S$ $(M+H)^+$: 437.1893, found: 437.1904.

Procedure for radical clock experiment (b):A dried 25 mL Schlenk tube was charged with **1v** (184.3 mg, 2.0 mmol), **2p** (38.0 mg, 0.20 mmol), **PC1** (3.4 mg, 0.010 mmol), DABCO·$(SO_2)_2$ (36.0 mg, 0.15 mmol), chiral nickel catalyst **[L7-Ni]** (4.0 mL, taken from the above-mentioned freshly prepared solution in DCE), and DCE (4.0 mL). The mixture was degassed *via* three freeze-pump-thaw cycles. The Schlenk tube was positioned approximately 5 cm away from a 24 W blue LEDs lamp (λ_{max} = 455 nm). After being stirred at 0 ℃ for 48 h, the reaction mixture was concentrated, then purified by flash chromatography on silica gel (eluted with CH_2Cl_2) to afford product **6** as a colorless oil (22.5 mg, 0.130 mmol, 65% yield).

^1H NMR (600 MHz, $CDCl_3$) δ 7.49 – 7.44 (m, 2H), 7.41 – 7.35 (m, 3H), 5.97 (s, 1H), 4.46 (d, *J* = 13.5 Hz, 1H), 4.28 (d, *J* = 13.5 Hz, 1H), 3.95 (dd, *J* = 17.3, 6.1 Hz, 1H), 3.47 (dd, *J* = 17.3, 6.7 Hz, 1H), 3.19 – 3.11 (m, 1H), 2.52 (s, 3H), 2.22 (s, 3H), 1.25 – 1.19 (m, 1H), 0.83 – 0.78 (m, 1H), 0.67 – 0.61 (m, 1H), 0.50 – 0.44 (m, 1H), 0.32 – 0.26 (m, 1H).

^{13}C NMR (151 MHz, $CDCl_3$) δ 170.34, 152.59, 144.21, 131.12, 128.92, 128.84, 127.06, 111.55, 63.09, 57.95, 35.36, 14.41, 13.82, 11.23, 6.37, 4.10.

IR (film): ν (cm^{-1}) 2975, 1723, 1587, 1456, 1410, 1383, 1302, 1119, 1026, 962, 868, 700, 656, 599, 507.

HRMS (ESI, m/z) calcd for $C_{18}H_{22}N_2NaO_3S$ $(M+Na)^+$: 369.1243, found: 369.1246.

5.3 Control Experiments

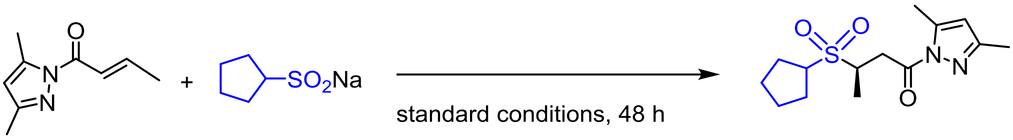

2a

3b

0% yield

A dried 25 mL Schlenk tube was charged with **2a** (32.8 mg, 0.20 mmol), sodium cyclopentanesulfinate (46.8 mg, 0.30 mmol), **PC1** (3.40 mg, 0.010 mmol), chiral nickel catalyst **[L7-Ni]** (4.00 mL, taken from the above-mentioned freshly prepared solution in DCE), and DCE (4.00 mL). The mixture was degassed *via* three freeze-pump-thaw cycles. The Schlenk tube was positioned approximately 5 cm away from a 24 W blue LEDs lamp (λ_{max} = 455 nm). After being stirred at 0 ℃ for 48 h, the resulting solution was concentrated to dryness. The residue was analysis by ^{1}H-NMR and HRMS. As a result, compound **3b** was not found.

5.4 Evidence for Lewis Acid Activation

5.4.1 Preparation of a Solution of the Non-Racemic Metal Catalyst [L*-M] in DCE

According to the published procedure,[7,8] a solution of metal salt (11.0 mg, 0.030 mmol) and non-racemic ligand **L7** (18.4 mg, 0.036 mmol) in 1,2-dimethoxyethane (DME, 4.0 mL) was stirred at 75 °C for 5 h, then the resulting solution was concentrated under reduced pressure to remove the solvent. The residue was redissolved in dichloroethane (DCE, 4.0 mL), which was used freshly as the metal catalyst for the photochemical reactions.

5.4.2 General Procedure

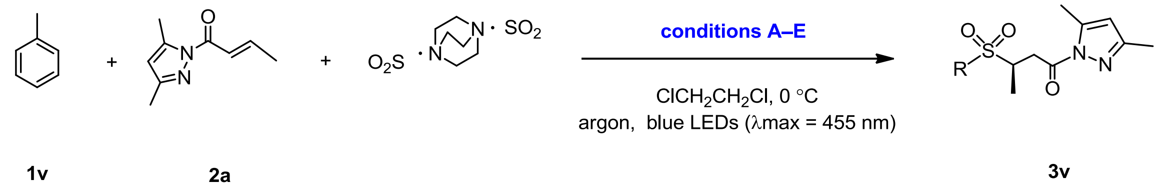

Condition A: A dried 25 mL Schlenk tube was charged with **1v** (184.3 mg, 2.0 mmol), **2a** (32.8 mg, 0.20 mmol), **PC1** (3.4 mg, 0.010 mmol), DABCO·$(SO_2)_2$ (36.0 mg, 0.15 mmol), chiral copper catalyst **[L7-Cu]** (4.0 mL, taken from the above-mentioned freshly prepared solution in DCE), and DCE (4.0 mL). The mixture was degassed *via* three freeze-pump-thaw cycles. The Schlenk tube was positioned approximately 5 cm away from a 24 W blue LEDs lamp (λ_{max} = 455 nm). After being stirred at 0 °C for 20 h, the reaction mixture was concentrated, then purified by flash chromatography on silica gel (eluted with PE:EtOAc = 4:1) to afford product **3v** as a yellow oil (23.6 mg, 0.074 mmol, 37% yield). Enantiomeric excess was established by HPLC analysis using a Chiralpak AD-H column, ee = 0% (HPLC: AD-H, 220 nm, *n*-hexane:isopropanol = 90:10, flow rate: 1.0 mL/min, 30 °C, t_r(minor) = 19.4 min, t_r(major) = 23.5 min).

Condition B: A dried 25 mL Schlenk tube was charged with **1v** (184.3 mg, 2.0 mmol), **2a** (32.8 mg, 0.20 mmol), **PC1** (3.4 mg, 0.010 mmol), DABCO·$(SO_2)_2$ (36.0 mg, 0.15 mmol), chiral iron catalyst **[L7-Fe]** (4.0 mL, taken from the above-mentioned freshly prepared solution in DCE), and DCE (4.0 mL). The mixture was degassed *via* three freeze-pump-thaw cycles. The Schlenk tube was positioned approximately 5 cm away from a 24 W blue LEDs lamp (λ_{max} = 455 nm). After being stirred at 0 °C for 20 h, the reaction mixture was concentrated, then purified by flash chromatography on silica gel (eluted with PE:EtOAc = 4:1) to afford product **3v** as a yellow oil (10.8 mg, 0.034 mmol, 17% yield). Enantiomeric excess was established by HPLC analysis using a Chiralpak AD-H column, ee = 13% (HPLC: AD-H, 220 nm, *n*-hexane:isopropanol = 90:10, flow rate: 1.0 mL/min, 30 °C, t_r(minor) = 19.5 min, t_r(major) = 23.5 min).

Condition C: A dried 25 mL Schlenk tube was charged with **1v** (184.3 mg, 2.0 mmol), **2a** (32.8 mg, 0.20 mmol), **PC1** (3.4 mg, 0.010 mmol), DABCO·$(SO_2)_2$ (36.0 mg, 0.15 mmol), The mixture was degassed *via* three freeze-pump-thaw cycles. The Schlenk tube was positioned approximately 5 cm away from a 24 W blue LEDs lamp (λ_{max} = 455 nm). After being stirred at 0 ℃ for 20 h, the resulting solution was concentrated to dryness. The residue was analysis by ^{1}H-NMR and HRMS. As a result, compound **3v** was not found.

Condition D: A dried 25 mL Schlenk tube was charged with **1v** (184.3 mg, 2.0 mmol), **2a** (32.8 mg, 0.20 mmol), **PC1** (3.4 mg, 0.010 mmol), DABCO·$(SO_2)_2$ (36.0 mg, 0.15 mmol), chiral zinc catalyst **[L7-Zn]** (4.0 mL, taken from the above-mentioned freshly prepared solution in DCE), and DCE (4.0 mL). The mixture was degassed *via* three freeze-pump-thaw cycles. The Schlenk tube was positioned approximately 5 cm away from a 24 W blue LEDs lamp (λ_{max} = 455 nm). After being stirred at 0 ℃ for 20 h, the reaction mixture was concentrated, then purified by flash chromatography on silica gel (eluted with PE:EtOAc = 4:1) to afford product **3v** as a yellow oil (14.1 mg, 0.044 mmol, 22% yield). Enantiomeric excess was established by HPLC analysis using a Chiralpak AD-H column, ee = 0% (HPLC: AD-H, 220 nm, *n*-hexane:isopropanol = 90:10, flow rate: 1.0 mL/min, 30 ℃, t_r(minor) = 19.4 min, t_r(major) = 23.4 min).

Condition E: A dried 25 mL Schlenk tube was charged with **1v** (184.3 mg, 2.0 mmol), **2a** (32.8 mg, 0.20 mmol), **PC1** (3.4 mg, 0.010 mmol), DABCO·$(SO_2)_2$ (36.0 mg, 0.15 mmol), chiral cobalt catalyst **[L7-Co]** (4.0 mL, taken from the above-mentioned freshly prepared solution in DCE), and DCE (4.0 mL). The mixture was degassed *via* three freeze-pump-thaw cycles. The Schlenk tube was positioned approximately 5 cm away from a 24 W blue LEDs lamp (λ_{max} = 455 nm). After being stirred at 0 ℃ for 20 h, the reaction mixture was concentrated, then purified by flash chromatography on silica gel (eluted with PE:EtOAc = 4:1) to afford product **3v** as a yellow oil (19.8 mg, 0.062 mmol, 31% yield). Enantiomeric excess was established by HPLC analysis using a Chiralpak AD-H column, ee = 80% (HPLC: AD-H, 220 nm, *n*-hexane:isopropanol = 90:10, flow rate: 1.0 mL/min, 30 ℃, t_r(minor) = 19.9

min, t_r(major) = 23.6 min).

5.5 Trend of *Z*/*E* isomerization of 2l

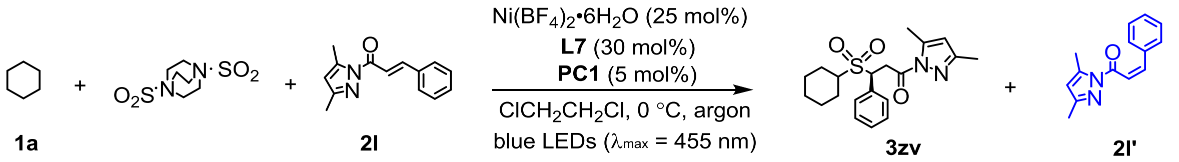

Supplementary Fig. 5 Formation of a *Z*/*E* isomerization product.

A dried 50 mL Schlenk tube was charged with **1a** (168.3 mg, 2.0 mmol), **2l** (135.7 mg, 0.60 mmol), **PC1** (3.4 mg, 0.010 mmol), DABCO·$(SO_2)_2$ (24.0 mg, 0.10 mmol), chiral nickel catalyst **[L7-Ni]** (4.0 mL, taken from the above-mentioned freshly prepared solution in DCE), and DCE (12.0 mL). The mixture was degassed *via* three freeze-pump-thaw cycles. The Schlenk tube was positioned approximately 5 cm away from a 24 W blue LEDs lamp (λ_{max} = 455 nm). After being stirred at 0 ℃ for 72 h, the reaction mixture was concentrated, then purified by flash chromatography on silica gel (eluted with CH_2Cl_2). The *Z*/*E*-isomerization product **2l’** was isolated as a colorless olil (38.1 mg, 0.168 mmol, 28% yield).

^{1}H NMR (600 MHz, $CDCl_3$) δ 7.63 – 7.59 (m, 2H), 7.38 – 7.30 (m, 3H), 7.12 (s, 2H), 5.99 (s, 1H), 2.57 (s, 3H), 2.26 (s, 3H).

^{13}C NMR (151 MHz, $CDCl_3$) δ 165.31, 152.17, 144.58, 144.41, 135.14, 129.96, 129.39, 128.34, 120.62, 111.45, 14.79, 14.04.

Note: *Such Z/E-isomerization was not observed in the case of α,β-unsaturated N-acylpyrazoles containing a β-alkyl substituent*

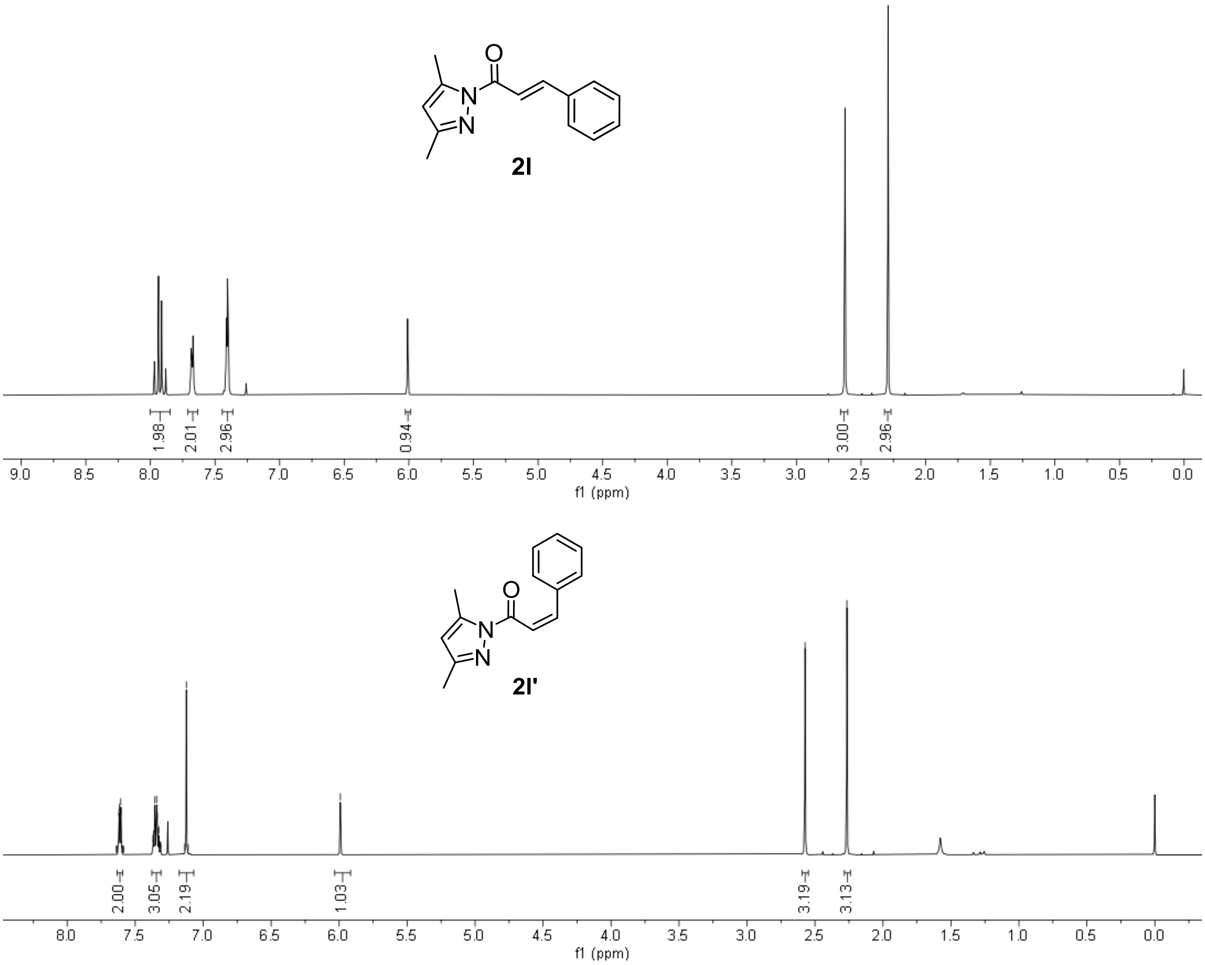

Supplementary Fig. 6 ^{1}H NMR of **2l** and **2l'**.

Control experiment: a phtochemical reaction of *Z*-isomer of the α,β-unsaturated *N*-acylpyrazole

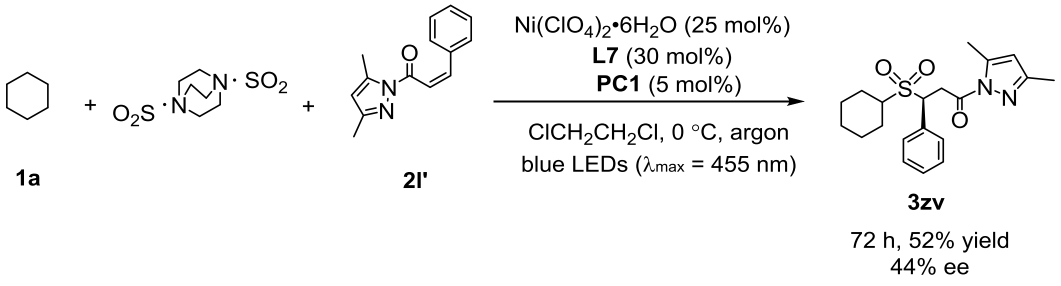

A dried 50 mL Schlenk tube was charged with **1a** (168.3 mg, 2.0 mmol), **2l'** (135.7 mg, 0.6 mmol), **PC1** (3.4 mg, 0.010 mmol), DABCO·$(SO_2)_2$ (24.0 mg, 0.10 mmol), chiral nickel

catalyst **[L7-Ni]** (4.0 mL, taken from the above-mentioned freshly prepared solution in DCE), and DCE (12.0 mL). The mixture was degassed *via* three freeze-pump-thaw cycles. The Schlenk tube was positioned approximately 5 cm away from a 24 W blue LEDs lamp (λ_{max} = 455 nm). After being stirred at 0 ℃ for 72 h, the reaction mixture was concentrated, then purified by flash chromatography on silica gel (eluted with PE:EtOAc = 4:1) to afford product **3zv** as a white solid (38.9 mg, 0.104 mmol, 52% yield). Enantiomeric excess was established by HPLC analysis using a Chiralpak IA column, ee = 44% (HPLC: IA, 240 nm, *n*-hexane:isopropanol = 90:10, flow rate: 1.0 mL/min, 30 ℃, t_r(minor) = 10.1 min, t_r(major) = 11.0 min)

5.6 Luminescence Quenching Experiments

Emission intensities were recorded on a Spectra Max M5 microplate reader in a 10.0 mm quartz cuvette. A solution of **PC1** was excited at 430 nm and the emission intensity at 525 nm was observed. DCE was selected as solvent, which was degassed with a stream of argon for 30 min. In a typical experiment, the sample was degassed with a stream of argon for 15 minutes, then the emission spectrum of the sample was collected. First, the emission spectrum of a 5×10^{-4} M solution of **PC1** in $C_2H_4Cl_2$ was collected. Then, appropriate amount of quencher was added to the measured solution and the emission spectrum of the sample was collected.

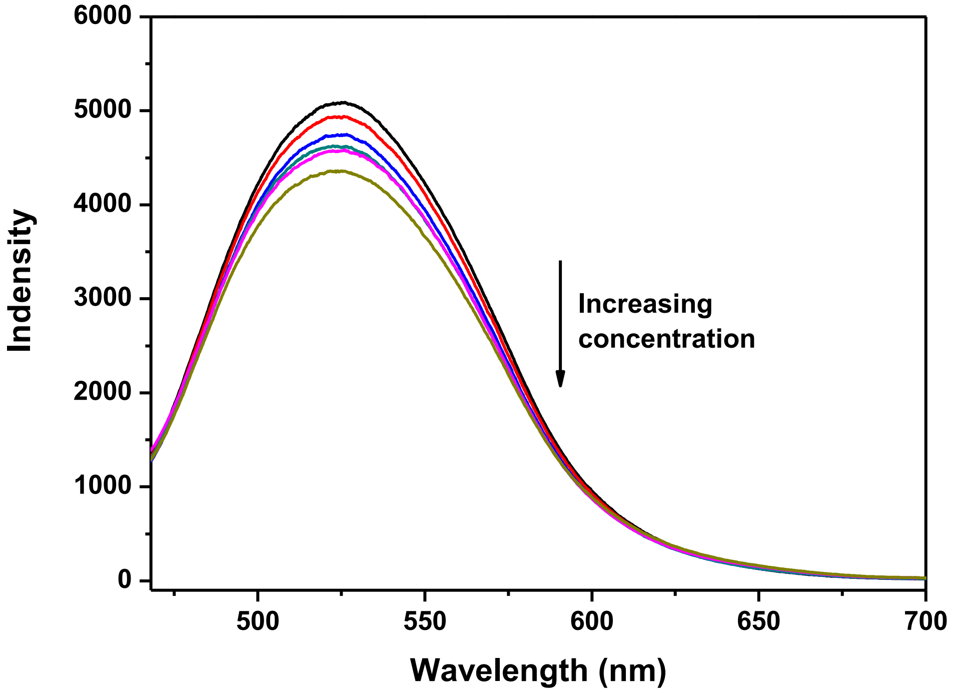

Supplementary Fig. 7 Emission quenching of **PC1** by toluene (**1v**).

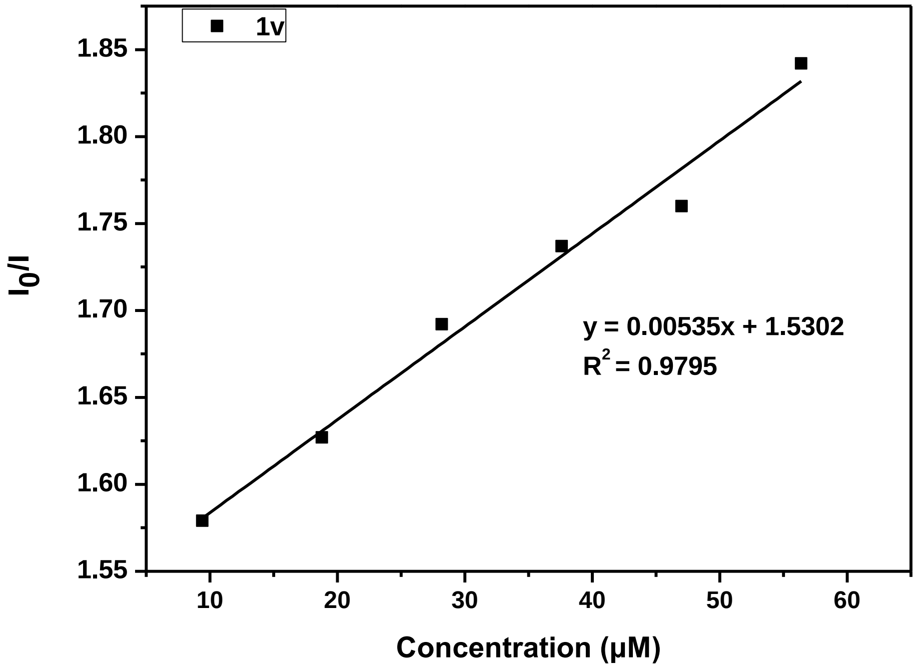

Supplementary Fig. 8 Stern-Volmer plots. I_0 and I are respective luminescence intensities in

the absence and presence of the indicated concentrations of the corresponding quencher.

5.7 Density Functional Theory (DFT) calculations

All density functional theory (DFT) calculations were performed using Gaussian 09 program[9]. Geometry optimizations were conducted with B3LYP functional, with def2SVP basis set[10]. Frequency analysis was performed to ensure all geometries were minimal.

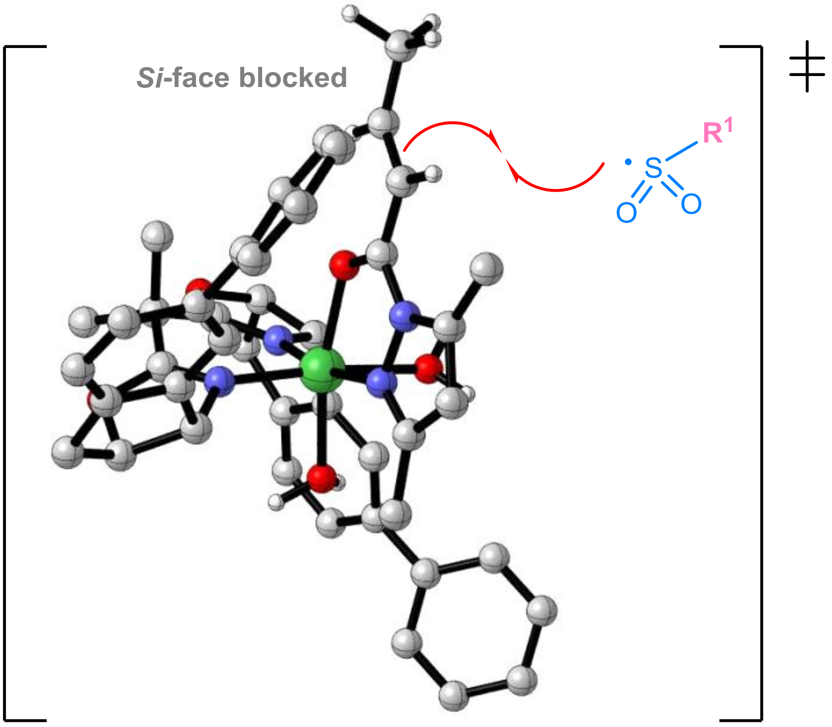

Supplementary Fig. 9 The coordination model of chiral nickel complex interacts with α,β-unsaturated carbonyl substrates which the nitrogen atom of the pyrazole coordinates to the planar site.

Atom	X	Y	Z
C	1.07457000	-0.97800300	-2.23201800
C	0.16086100	-2.50771500	-0.89265700
C	0.72297800	-2.23663500	-3.06558600
H	0.69585500	-0.04083600	-2.66736400
H	-0.17912000	-2.15526400	-3.69112800
C	-1.39559300	-2.63184500	1.07272200
C	-2.88966000	-1.22319600	1.98971400
C	-3.15695900	-2.67696500	2.49374400

H	-2.66571100	-0.52074100	2.80728300
H	-2.97785900	-2.82687300	3.56647600
C	-0.27585200	-3.32522700	0.31088800
C	0.95046800	-3.44564000	1.26614200
H	1.77943700	-3.94066600	0.73964000
H	1.28583400	-2.45766600	1.60968200
H	0.67804000	-4.06134900	2.13539100
C	-0.73447400	-4.73396900	-0.12810700
H	0.08063200	-5.23997800	-0.65959400
H	-1.00461700	-5.32834900	0.75233400
H	-1.60384900	-4.69008500	-0.79968400
N	0.40401500	-1.23845600	-0.92759700
N	-1.67451500	-1.37275600	1.13170400
O	0.39792600	-3.19186100	-2.00304600
O	-2.14378500	-3.45199400	1.79514400
C	2.59905100	-1.01950500	-2.18626900
C	3.08952900	-1.96224200	-3.10273500
C	3.47574600	-0.26441100	-1.40545000
C	4.46631100	-2.13349700	-3.25869100
C	4.86614700	-0.43417100	-1.54313800
H	3.09384100	0.47175900	-0.69868600
C	5.34121900	-1.36837000	-2.48648900
H	4.86104100	-2.86423900	-3.96894600
H	6.41814100	-1.51605500	-2.59293500
C	1.97027200	-2.66480000	-3.83382800
H	2.06908300	-3.76126100	-3.85570300
H	1.89401700	-2.33267000	-4.88347700
C	5.82209900	0.34353400	-0.70791700
C	6.93347500	0.98281900	-1.28753700
C	5.65368600	0.43078600	0.68663600
C	7.84135500	1.69502800	-0.49997700
H	7.08110000	0.93408900	-2.36915000
C	6.56808500	1.13554300	1.47583300
H	4.82522100	-0.10257100	1.16076000
C	7.66296700	1.77468500	0.88454400
H	8.69565200	2.18756200	-0.97038100
H	6.44503200	1.16209800	2.56222200
H	8.38263900	2.31856200	1.50081900
C	-4.15680600	-0.85412800	1.23336400
C	-5.04907600	-1.93303700	1.20348800
C	-4.49215900	0.36475600	0.62973100
C	-6.25798800	-1.81160900	0.50967300

C	-5.70764900	0.50363800	-0.07187800
H	-3.83594700	1.23010000	0.74249400
C	-6.56888700	-0.61357800	-0.13393600
H	-6.96965600	-2.64078400	0.48494500
H	-7.52484800	-0.52073400	-0.65305100
C	-4.56855300	-3.09083800	2.04622400
H	-4.53720200	-4.05115200	1.50749300
H	-5.22420300	-3.24302700	2.91931600
C	-6.07759600	1.79544800	-0.70902400
C	-6.71469300	1.82438500	-1.96422300
C	-5.80829000	3.01983400	-0.06721600
C	-7.06418600	3.03780700	-2.56028900
H	-6.92635200	0.88928600	-2.48862600
C	-6.16390500	4.23250500	-0.66123000
H	-5.36016700	3.02167200	0.93012500
C	-6.79071700	4.24570600	-1.91127500
H	-7.55539300	3.03973100	-3.53619500
H	-5.97016100	5.17185700	-0.13745300
H	-7.07516300	5.19385700	-2.37335000
C	0.92198800	2.78302700	-1.00077900
C	1.97897600	3.64692000	-0.59709900
H	2.33126700	4.53262700	-1.12126900
N	0.77144000	1.80911800	-0.11222700
C	1.72207500	1.07989000	1.92504600
N	1.71903400	2.01711100	0.87348500
C	2.47624500	3.16026800	0.58643300
C	0.09139800	2.90563300	-2.23496000
H	0.67695800	2.59227700	-3.11596900
H	-0.81853300	2.29664700	-2.17398500
H	-0.18857100	3.95642500	-2.40304800
C	3.59298500	3.73566700	1.39307700
H	4.45226400	3.04895500	1.45250400
H	3.93782200	4.65241900	0.89786400
H	3.27867600	4.00572600	2.41265700
Ni	-0.49668300	0.15311400	0.31906700
O	0.86998500	0.17878800	1.88075900
C	2.68818700	1.19155100	3.00901300
C	2.64680900	0.33581700	4.05745500
C	3.57770800	0.35153800	5.21325000
H	3.01153600	0.47005400	6.15435000
H	4.08779300	-0.62459300	5.29990200
H	4.33343500	1.14641200	5.14819500

O	-1.99023000	0.52191000	-1.15645300
O	-1.62094300	1.64741400	1.48390900
H	3.44979500	1.96390800	2.96777800
H	1.86389800	-0.43158400	4.05812900
H	-1.66492700	1.65556600	2.45184800
H	-1.58038200	2.57523000	1.20690000
H	-2.02186600	-0.10287400	-1.89644400
H	-2.89368700	0.50575200	-0.77463200

Geometry optimization of other possible isomers of the nickel intermediate via DFT calculations.

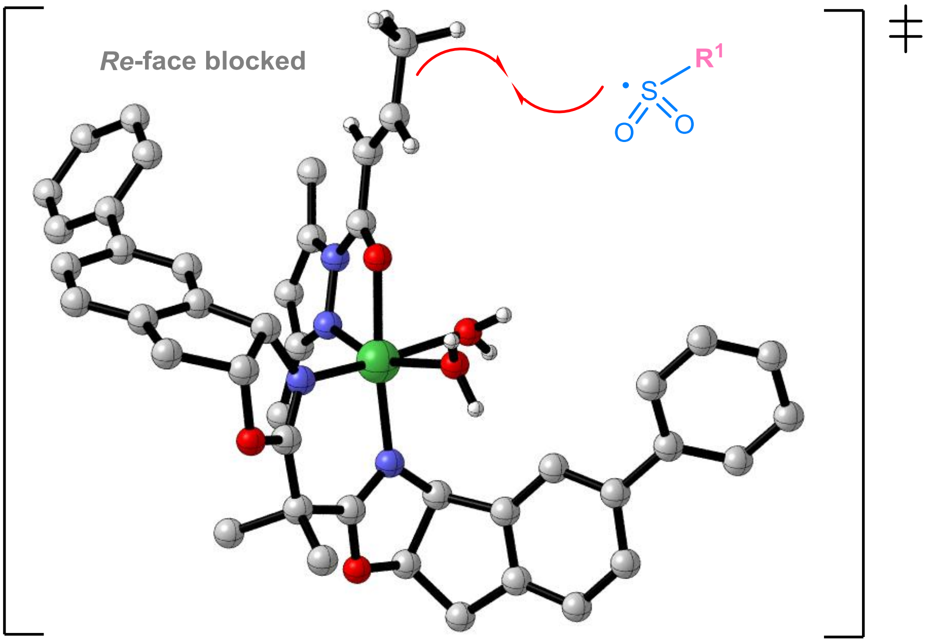

Supplementary Fig. 10 The coordination model of chiral nickel complex interacts with α,β-unsaturated carbonyl substrates which the nitrogen atom of the pyrazole coordinates to the axial site.

Atom	X	Y	Z
C	-1.48350200	-0.94015600	2.29302800
C	-0.11466500	-2.44242500	1.35282500
C	-1.23433300	-2.10539600	3.29281600
H	-1.26557800	0.05658400	2.70416800
H	-0.57504400	-1.86429700	4.13938600

C	1.42434100	-2.58236100	-0.66605100
C	2.58070800	-1.14283300	-1.93372500
C	2.71362500	-2.57220100	-2.53312000
H	2.22088400	-0.40047400	-2.66301800
H	2.15819500	-2.74101900	-3.46642700
C	0.68594600	-3.33123300	0.42309700
C	-0.32070600	-4.32551300	-0.22707800
H	-0.82203500	-4.90725200	0.55772700
H	-1.08727300	-3.79369100	-0.81028900
H	0.21630600	-5.01670200	-0.88964200
C	1.75080700	-4.11899800	1.23801200
H	1.25687100	-4.70163100	2.02511100
H	2.29364800	-4.80476900	0.57573100
H	2.47735300	-3.44039200	1.71084800
N	-0.52624000	-1.23143300	1.18246300
N	1.56268200	-1.31014900	-0.85424700
O	-0.50357900	-3.05845400	2.46173600
O	2.05939800	-3.38295200	-1.51090900
C	-2.95167100	-1.10515200	1.92042100
C	-3.54600700	-2.11852200	2.68743400
C	-3.70327800	-0.39018900	0.98589100
C	-4.88879500	-2.44738600	2.48955200
C	-5.06263300	-0.69563900	0.78921500
H	-3.24564300	0.39712400	0.38787100
C	-5.63239200	-1.74371700	1.54151700
H	-5.36341200	-3.23393000	3.08149600
H	-6.68932500	-1.98278700	1.40513700
C	-2.58771600	-2.68171700	3.70901000
H	-2.55553200	-3.78184800	3.74148500
H	-2.84503800	-2.34340000	4.72723700
C	-5.89534200	0.08290200	-0.16797900
C	-5.87414700	1.49058700	-0.15594500
C	-6.75441800	-0.56448400	-1.07469700
C	-6.68925200	2.22623600	-1.02118100
H	-5.25252600	2.01041100	0.57867700
C	-7.55948400	0.17120200	-1.94798100
H	-6.78609800	-1.65653200	-1.10310300
C	-7.53110400	1.56889600	-1.92425300
H	-6.69376900	3.31863800	-0.97021200
H	-8.21899800	-0.35053500	-2.64563400
H	-8.17567500	2.14300900	-2.59397500
C	3.98268400	-0.84446800	-1.41671000

C	4.87217400	-1.86626900	-1.77690200
C	4.42938900	0.26517400	-0.69487800
C	6.21117200	-1.78618400	-1.38435900
C	5.77509100	0.36471000	-0.29121800
H	3.74893500	1.08397600	-0.47137000
C	6.64823700	-0.68754300	-0.64316700
H	6.91691800	-2.57724900	-1.64991100
H	7.69022400	-0.64784400	-0.32015300
C	4.20202700	-2.91711200	-2.62966300
H	4.38878300	-3.95110700	-2.30027600
H	4.53733100	-2.85556300	-3.67895100
C	6.25681500	1.54849700	0.46943700
C	5.42929500	2.19828100	1.40662400
C	7.55478500	2.05565000	0.26967900
C	5.88195000	3.31025900	2.11910500
H	4.42557500	1.81167100	1.60349500
C	8.00705700	3.16962200	0.97960100
H	8.21272900	1.59104200	-0.46789500
C	7.17398700	3.80100500	1.90762800
H	5.23017100	3.78995900	2.85371500
H	9.01581200	3.54964800	0.80242500
H	7.53113900	4.66928500	2.46593900
Ni	0.39049600	0.17898400	-0.01715500
O	1.93006800	0.54148700	1.47727500
O	1.46952000	1.86811000	-0.97658400
H	1.94641400	1.93499600	-1.81679100
H	1.98814400	2.37607900	-0.33349300
H	1.75575900	0.68779700	2.41897200
H	2.73399600	-0.00103000	1.42273200
C	-1.54687700	0.22349600	-2.61288400
C	-2.41294400	1.20648200	-3.16959900
C	-2.48281700	2.24103400	-2.27095700
N	-1.09522000	0.63241500	-1.43769500
H	-2.93010000	1.15439000	-4.12506400
O	-0.61401800	1.80051700	0.80292700
N	-1.66943400	1.86539100	-1.19460900
C	-1.38625400	2.43139300	0.06148900
C	-1.99860700	3.69128800	0.45654100
C	-3.26088900	3.50591500	-2.42222900
H	-4.08141000	3.57146000	-1.69184600
H	-2.62766100	4.40149200	-2.33528600
H	-3.71548600	3.51248800	-3.42109200

C	-1.18435000	-1.09076300	-3.21671600
H	-0.51663000	-1.66089500	-2.56150600
H	-2.09436000	-1.68555100	-3.39487100
H	-0.69972400	-0.94912400	-4.19599500
C	-1.71389600	4.25034500	1.65636300
H	-2.69520800	4.18103200	-0.21681900
H	-1.00698200	3.72415000	2.30840000
C	-2.28301600	5.52266800	2.16549500
H	-2.80413300	5.34571600	3.12345200
H	-1.47072100	6.23325300	2.40115600
H	-2.98030300	5.99634200	1.46092800

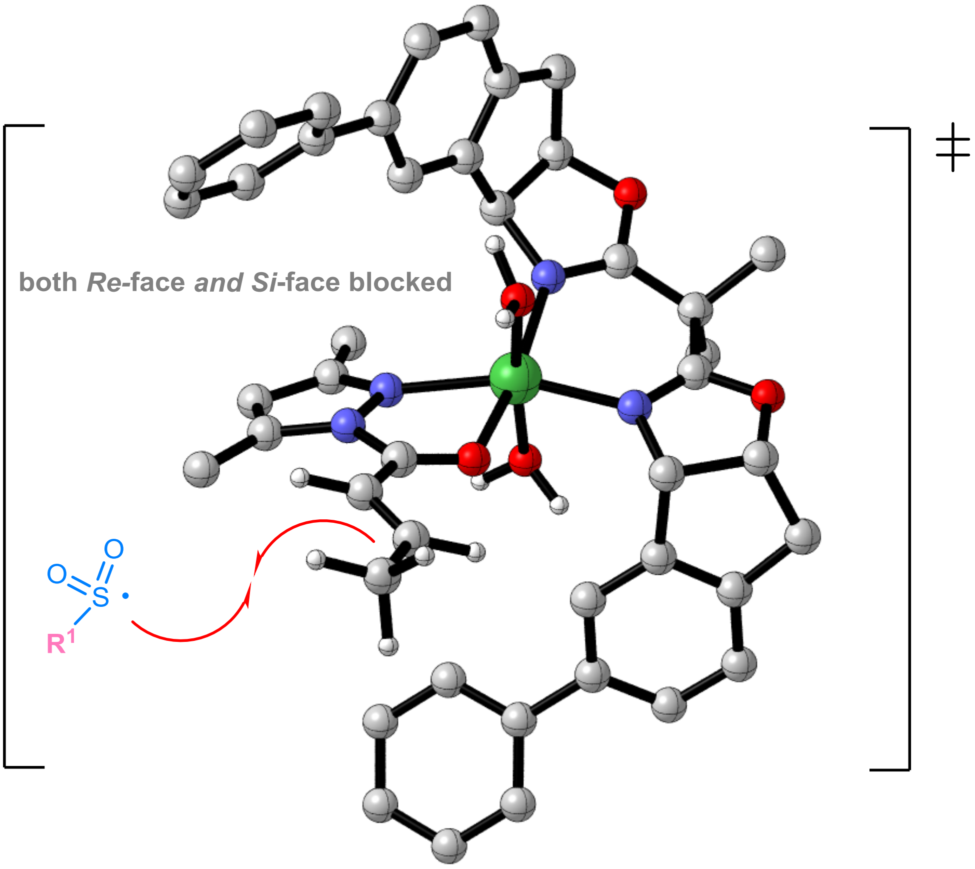

Supplementary Fig. 11 The coordination model of chiral nickel complex interacts with α,β-unsaturated carbonyl substrates which the both oxygen and nitrogen atoms coordinate to the planar sites.

Atom	X	Y	Z
C	2.46690200	-1.97303900	-1.70241700
C	0.92369300	-3.33769100	-0.79747100
C	2.86094300	-3.45750900	-1.96732800
H	2.30232600	-1.40552100	-2.62665100
H	2.83926900	-3.74775900	-3.02594100
C	-0.99803500	-3.15856900	0.86313500
C	-2.09670200	-1.53336900	1.95620000
C	-2.39439800	-2.90586300	2.63107500
H	-1.67555400	-0.78111900	2.63876500
H	-1.94149400	-3.04022700	3.62278400
C	-0.26061400	-4.02749900	-0.13925200
C	0.21906300	-5.32598900	0.55712500
H	0.69678100	-5.98821800	-0.17365000
H	0.94192400	-5.11054300	1.35814700
H	-0.63853300	-5.84640400	0.99867200
C	-1.27297400	-4.38154200	-1.27391300
H	-0.79086300	-5.05168700	-1.99955100
H	-2.13993600	-4.90340500	-0.84486400
H	-1.61585800	-3.47551800	-1.79313500
N	1.16461800	-2.08026300	-0.97331800
N	-1.08175200	-1.87394300	0.91918000
O	1.79409200	-4.19420000	-1.31767800
O	-1.71842400	-3.84286700	1.74221700
C	3.63046600	-1.41648500	-0.90086000
C	4.57834800	-2.41362000	-0.63589400
C	3.83721100	-0.09990000	-0.47278600
C	5.72030700	-2.09941500	0.10784900
C	4.98176000	0.23158400	0.28165700
H	3.11246200	0.67932300	-0.71520000
C	5.90711000	-0.79546500	0.56925500
H	6.47589800	-2.86119100	0.31557500
H	6.81334900	-0.55497800	1.12868100
C	4.22105900	-3.72458100	-1.29616800
H	4.14282200	-4.56473800	-0.58751500
H	4.97745600	-4.01522000	-2.04300400
C	5.20893600	1.62604300	0.74790600
C	5.75550800	1.88308400	2.01978500
C	4.89311000	2.72359100	-0.07672500
C	5.97508400	3.19229500	2.45401100

H	6.00212100	1.05108500	2.68390100
C	5.11945900	4.03253500	0.35570300
H	4.51401800	2.54976200	-1.08709300
C	5.65873500	4.27201800	1.62393100
H	6.40131000	3.36947100	3.44428800
H	4.90252900	4.87049800	-0.31205700
H	5.84707300	5.29501800	1.95801800
C	-3.44092700	-1.11589900	1.37660000
C	-4.43561400	-2.04901000	1.70023400
C	-3.74705400	0.02760200	0.63113800
C	-5.73934200	-1.85626100	1.23430000
C	-5.05721700	0.23531400	0.15182500
H	-2.98565700	0.79073900	0.46953600
C	-6.03612700	-0.73486300	0.45794200
H	-6.52681000	-2.57663100	1.46976500
H	-7.05108400	-0.61034900	0.07582200
C	-3.91122900	-3.13416600	2.61092700
H	-4.14908600	-4.15556000	2.27484600
H	-4.32680900	-3.03386100	3.62770600
C	-5.39385800	1.44736300	-0.64230400
C	-6.65948600	2.05510900	-0.53834500
C	-4.45610200	2.02435100	-1.52112100
C	-6.97440000	3.19176200	-1.28576100
H	-7.40181500	1.65011700	0.15268200
C	-4.77033600	3.15820100	-2.27211700
H	-3.47289600	1.55991700	-1.63801500
C	-6.03305000	3.74755600	-2.15691100
H	-7.96162000	3.64830500	-1.18371300
H	-4.03373700	3.57813500	-2.96200000
H	-6.28381000	4.63257400	-2.74604500
Ni	-0.00514000	-0.53391200	-0.20658300
O	-1.63558400	-0.74951700	-1.60461900
H	-2.49749000	-0.84972200	-1.15733100
H	-1.79448100	-0.15429700	-2.35235300
C	0.89593400	1.60437800	-2.57258400
C	1.05235000	3.01725700	-2.63621200
C	0.73924200	3.51453000	-1.39482600
N	0.41070700	2.40250400	-0.61432800
H	1.34279700	3.60211500	-3.50640600
N	0.52431100	1.24089800	-1.35086600
C	-0.21155900	2.26790300	0.64329400
C	-1.15432600	4.45071900	3.66695800

H	-0.49679300	5.30543300	3.45703600
H	-2.19912600	4.80507700	3.72601400
H	-0.92309800	4.05530400	4.67218000
C	-0.25358700	3.39862200	1.55950500
H	0.37009800	4.26640400	1.35944400
C	-1.04489500	3.36835800	2.65817300
H	-1.66772400	2.47914200	2.81318000
O	-0.68691300	1.15644000	0.91540500
O	1.48103900	-0.41961100	1.33301700
H	2.42173000	-0.49404100	1.08267000
H	1.43186400	0.21791000	2.05938100
C	0.72516900	4.94622400	-0.97129900
H	-0.25417100	5.25944500	-0.57990700
H	1.49079500	5.15517200	-0.20729200
H	0.95334200	5.56942200	-1.84564400
C	1.07424100	0.64581800	-3.70337200
H	2.14256600	0.50808500	-3.93777800
H	0.62966400	-0.33300300	-3.48308200
H	0.60453700	1.04985200	-4.61311900

5.8 An alternative Reaction Pathway

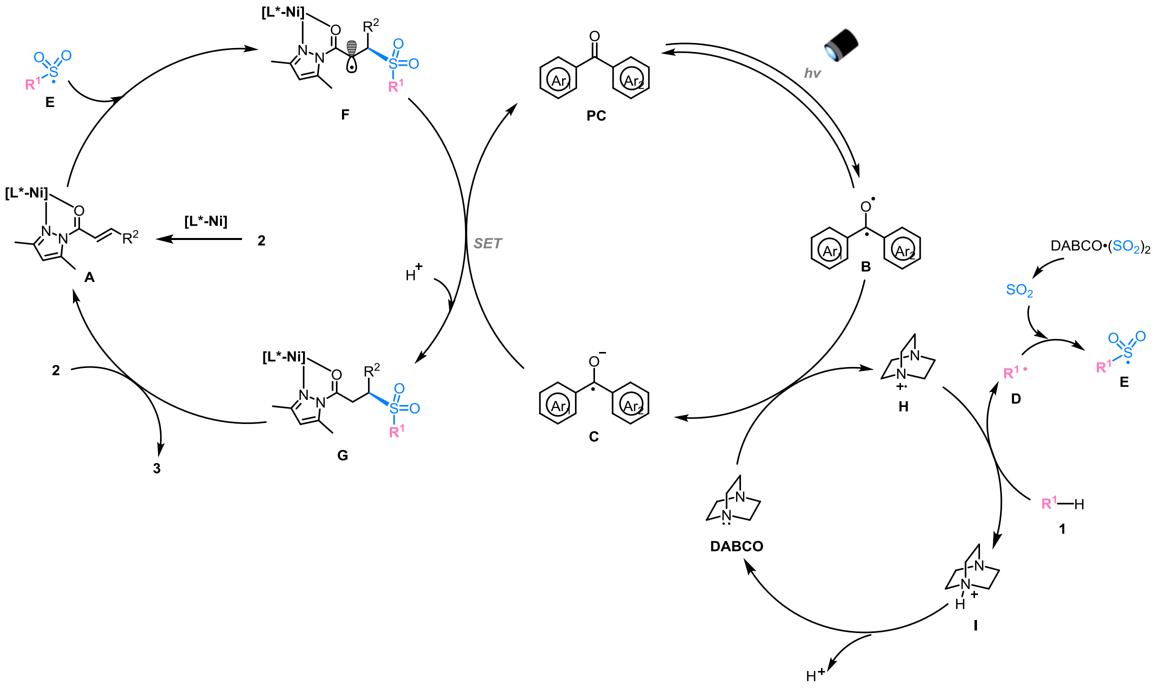

Supplementary Fig. 12 A pathway involving DABCO-participated reductive quench.

Control experiment: a photochemical reaction in the presence of an iridium photocatalyst (as reference 58)

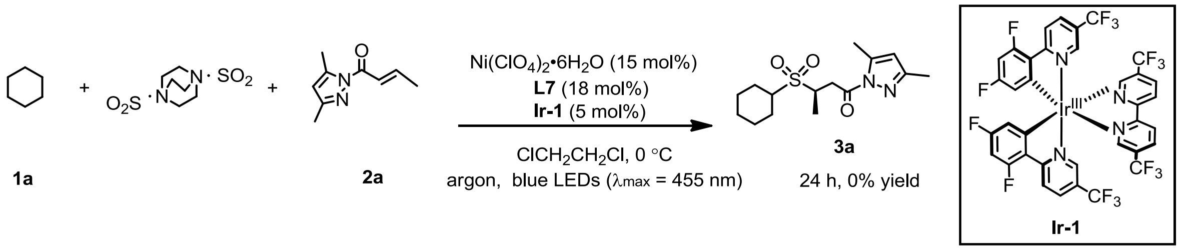

A dried 25 mL Schlenk tube was charged with **1a** (168.3 mg, 2.0 mmol), **2a** (32.8 mg, 0.20 mmol), **Ir-1** (11.4 mg, 0.010 mmol), DABCO·$(SO_2)_2$ (36.0 mg, 0.15 mmol), chiral nickel catalyst **[L7-Ni]** (4.0 mL, taken from the above-mentioned freshly prepared solution in DCE), and DCE (4.0 mL). The mixture was degassed *via* three freeze-pump-thaw cycles. The Schlenk tube was positioned approximately 5 cm away from a 24 W blue LEDs lamp (λ_{max} = 455 nm). After being stirred at 0 ℃ for 24 h, the resulting solution was concentrated to dryness. The residue was analysis by ^{1}H-NMR and HRMS. As a result, compound **3a** was not found.

Note: On the basis of this result, we assume that a pathway involving DABCO-participated reductive quench might exist, but would not be the dominant mechanism.

6. Chiral Chromatography

6.1 Determination of Enantioselectivities of the Asymmetric Photoredox Reactions

Optical purities of the compounds **3a**–**3zy** and **5** were determined with a Daicel Chiralpak AD-H, AY-H, AS-H, OD-H, OX-H, OJ-H, IA, IE HPLC column on an Agilent 1260 Series HPLC System. The column temperature was 30 ℃ and UV-absorption was measured at 220 nm, 240 nm or 254 nm.

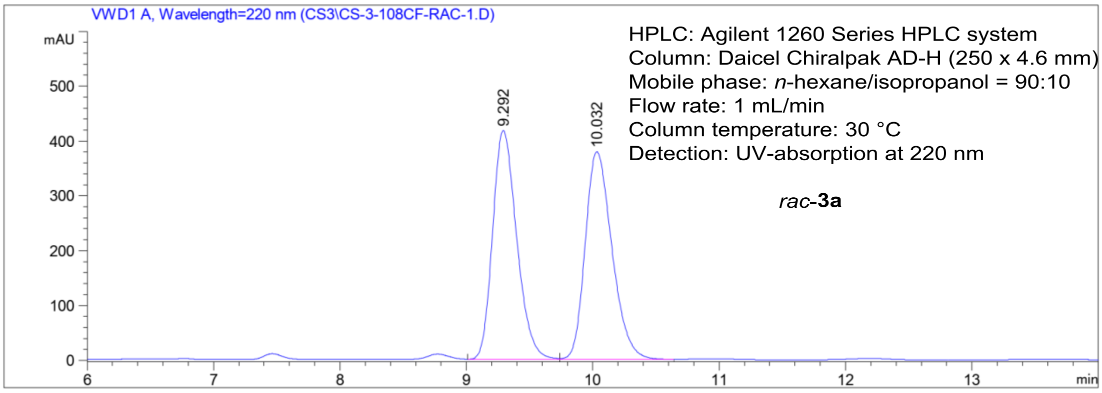

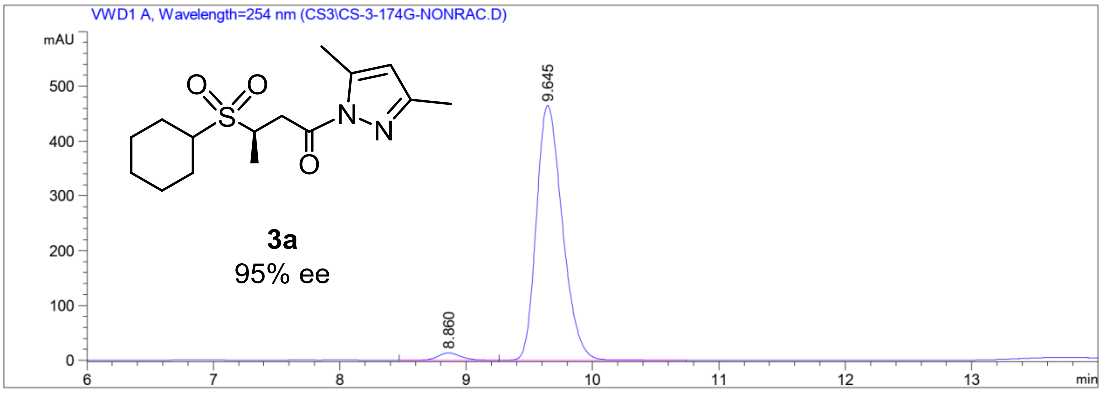

#	[min]		[min]	[mAU*s]	[mAU]	%
1	8.860	BB	0.1862	160.72073	13.28323	2.3682
2	9.645	BB	0.2214	6626.02441	464.55457	97.6318

Supplementary Fig. 13 HPLC trace for the racemic reference *rac*-**3a**, and non-racemic product **3a** generated from the photocatalytic asymmetric reaction.

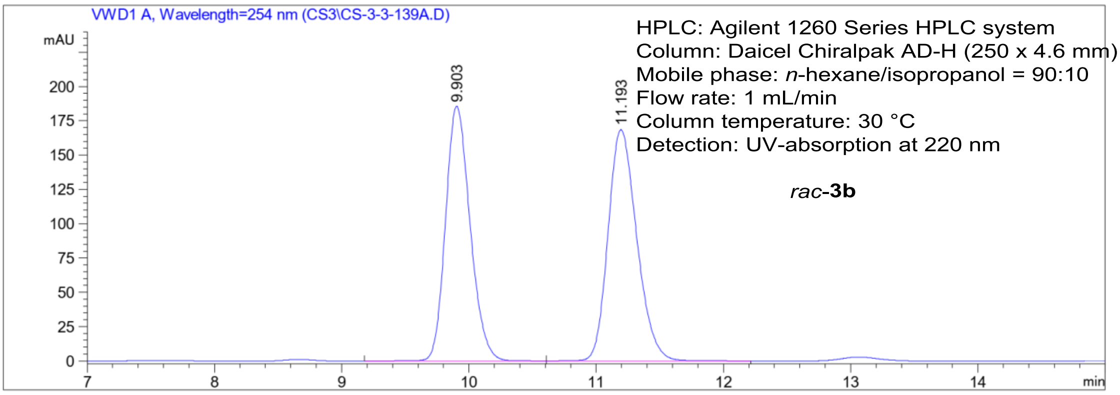

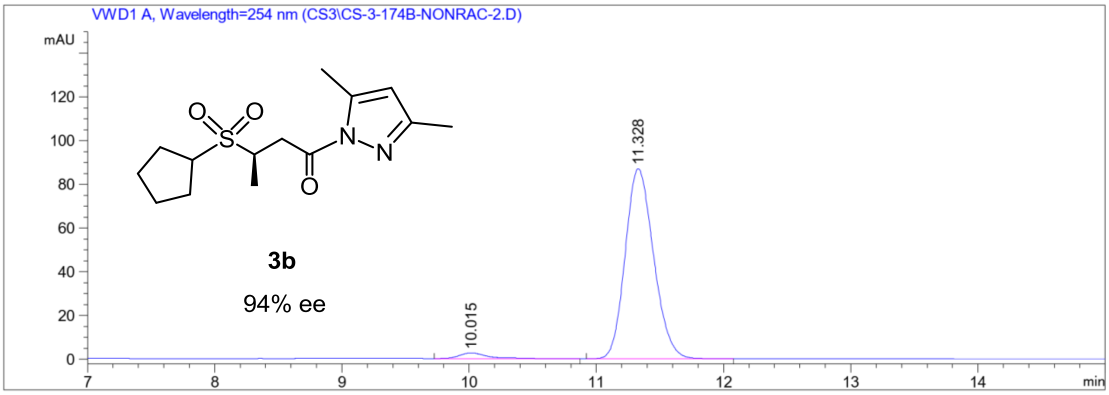

#	[min]		[min]	[mAU*s]	[mAU]	%
1	10.015	BB	0.2263	39.31015	2.58668	2.8341
2	11.328	BB	0.2399	1347.71045	86.93405	97.1659

Supplementary Fig. 14 HPLC trace for the racemic reference *rac*-**3b**, and non-racemic product **3b** generated from the photocatalytic asymmetric reaction.

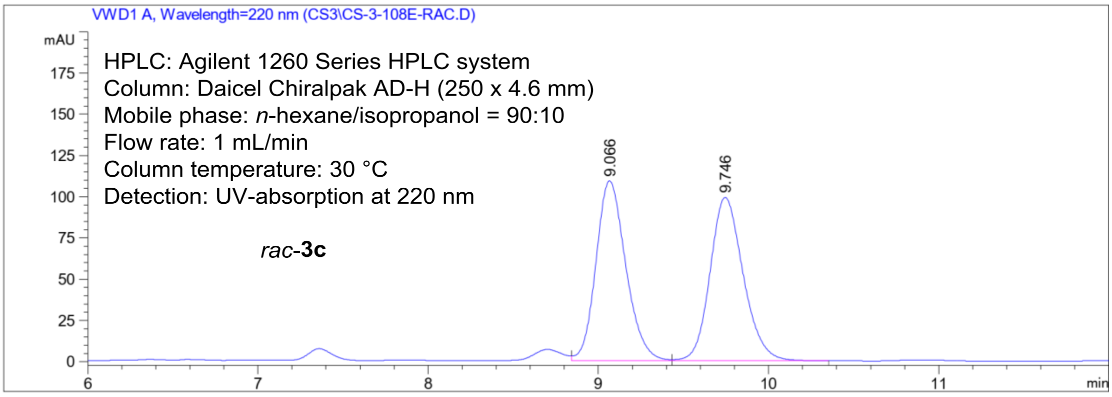

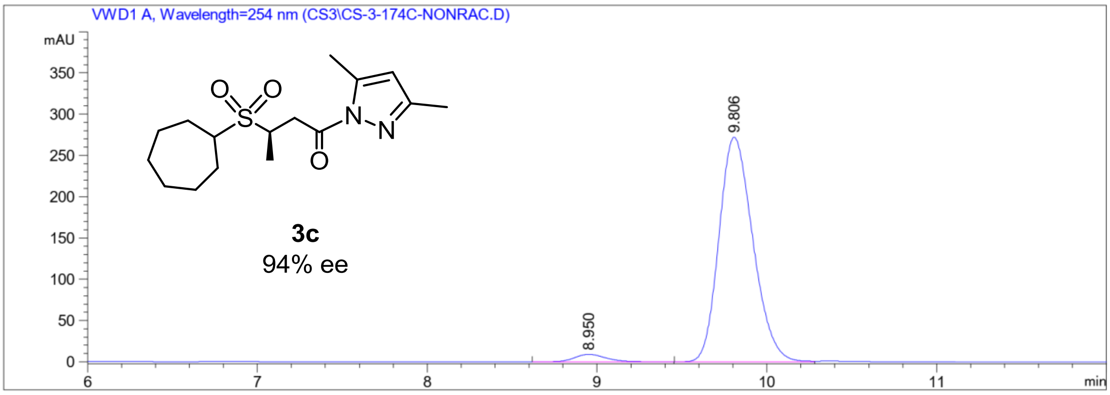

#	[min]		[min]	[mAU*s]	[mAU]	%
1	8.950	BB	0.1938	112.88151	8.96966	2.9032
2	9.806	BV	0.2160	3775.27197	271.87210	97.0968

Supplementary Fig. 15 HPLC trace for the racemic reference *rac*-**3c**, and non-racemic product **3c** generated from the photocatalytic asymmetric reaction.

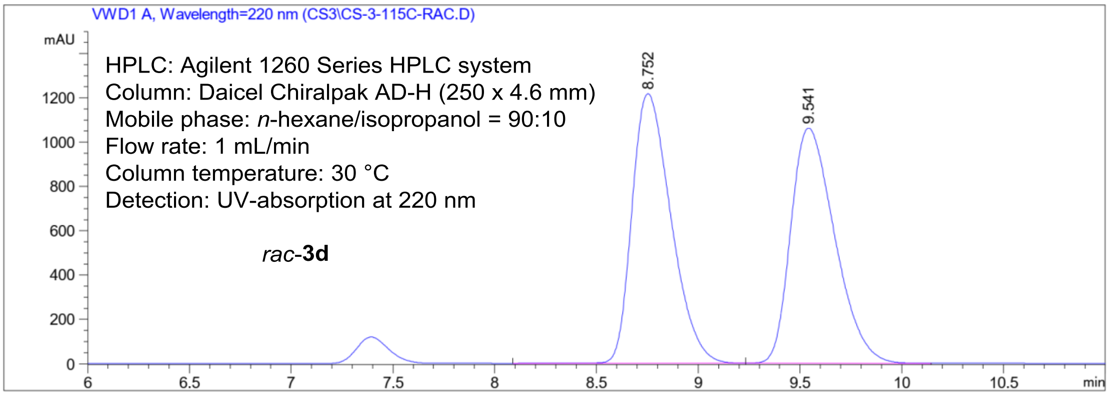

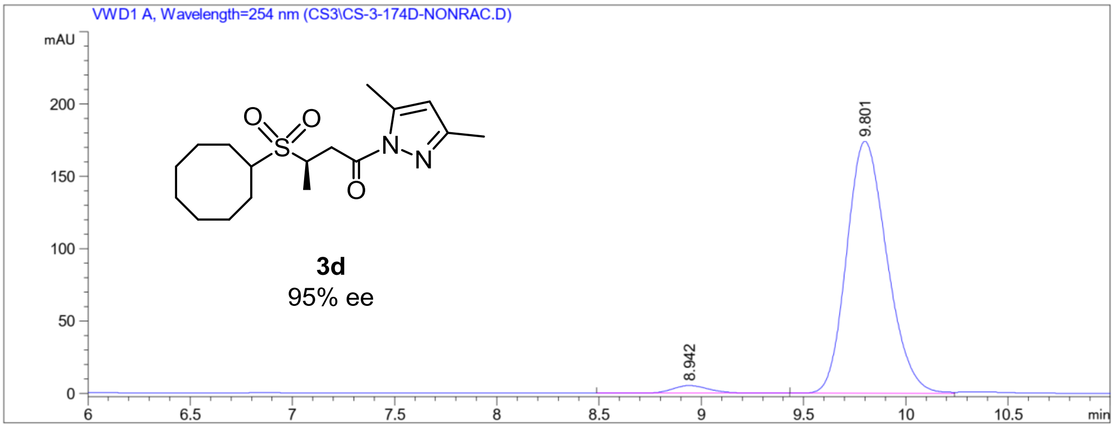

#	[min]		[min]	[mAU*s]	[mAU]	%
1	8.942	BB	0.1918	65.93766	5.27770	2.6904
2	9.801	BV	0.2129	2384.94214	174.00635	97.3096

Supplementary Fig. 16 HPLC trace for the racemic reference *rac*-**3d**, and non-racemic product **3d** generated from the photocatalytic asymmetric reaction.

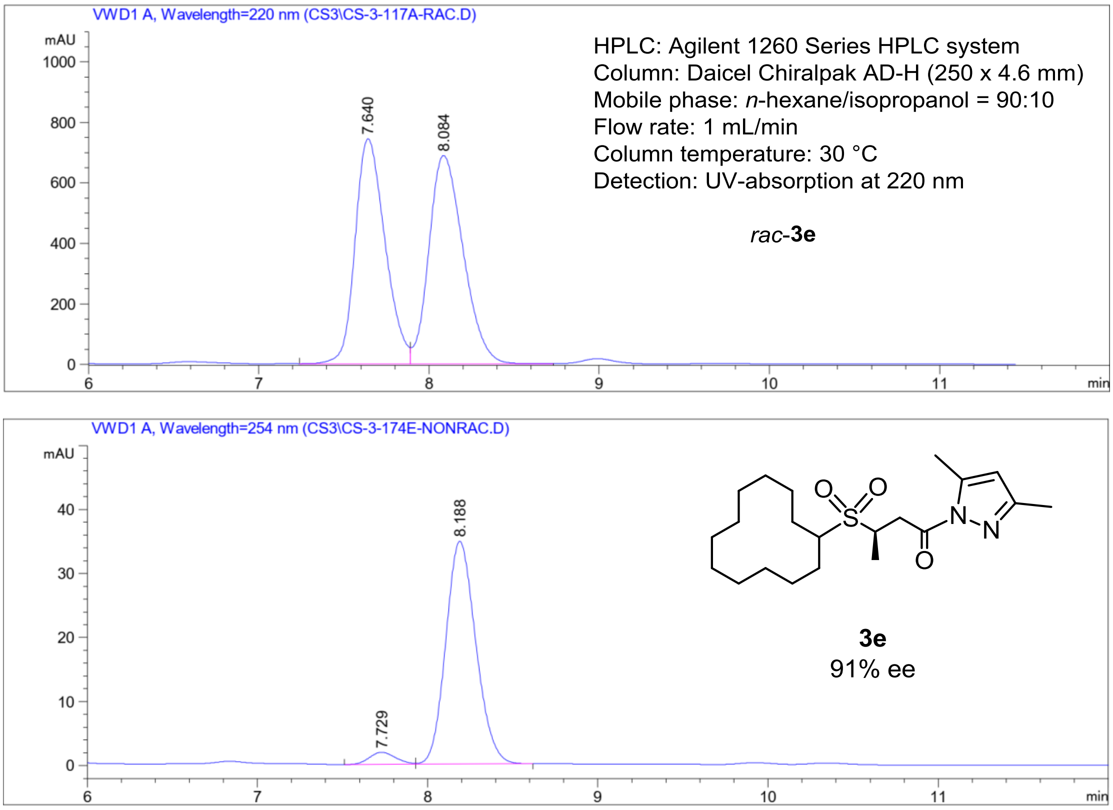

#	[min]		[min]	[mAU*s]	[mAU]	%
1	7.729	BV	0.1670	20.18278	1.88442	4.5117
2	8.188	VB	0.1903	427.15631	34.78365	95.4883

Supplementary Fig. 17 HPLC trace for the racemic reference *rac*-**3e**, and non-racemic product **3e** generated from the photocatalytic asymmetric reaction.

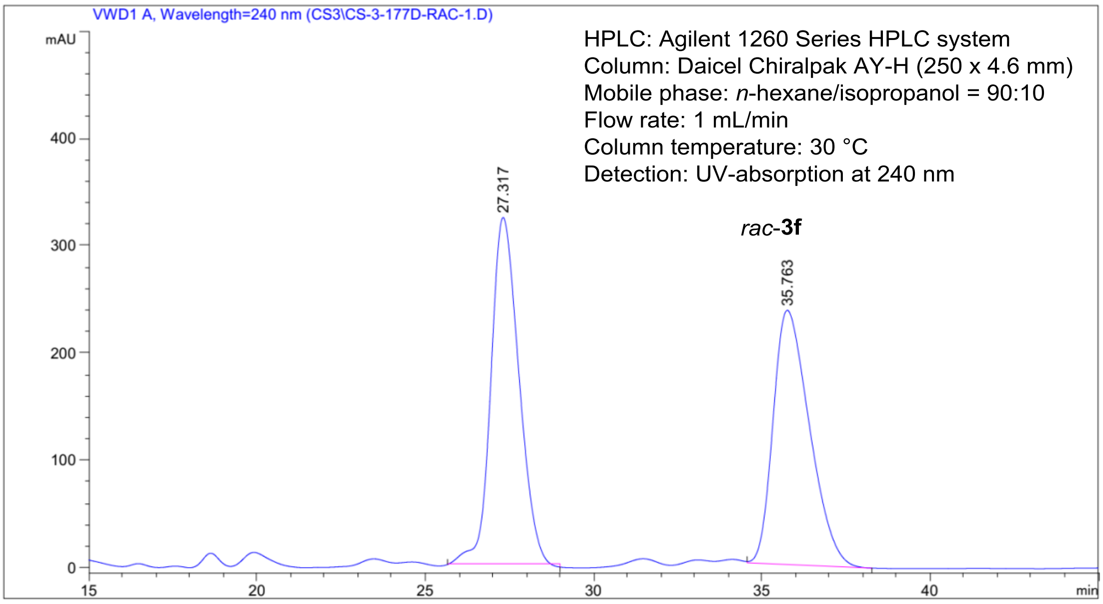

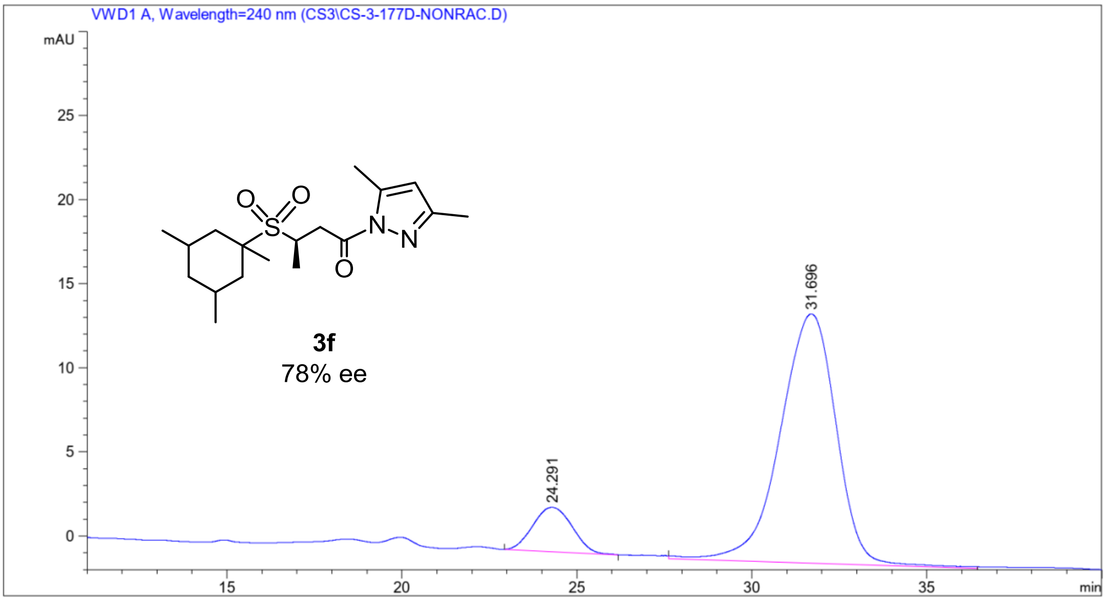

#	[min]		[min]	[mAU*s]	[mAU]	%
1	24.291	BB	0.9173	204.27173	2.63253	11.0042
2	31.696	MM R	1.8577	1652.02808	14.82172	88.9958

Supplementary Fig. 18 HPLC trace for the racemic reference *rac*-**3f**, and non-racemic product **3f** generated from the photocatalytic asymmetric reaction.

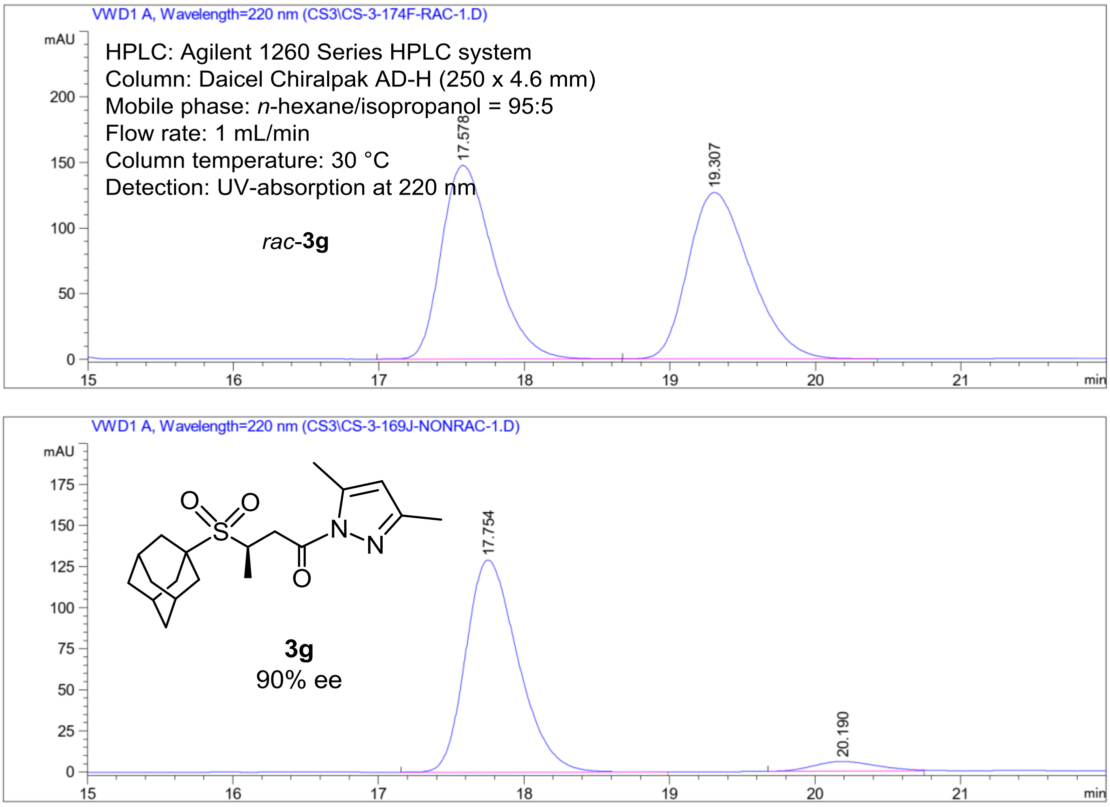

#	[min]		[min]	[mAU*s]	[mAU]	%
1	17.754	BB	0.3847	3200.31323	128.88065	94.9504
2	20.190	MM R	0.4916	170.19846	5.77043	5.0496

Supplementary Fig. 19 HPLC trace for the racemic reference *rac*-**3g**, and non-racemic product **3g** generated from the photocatalytic asymmetric reaction.

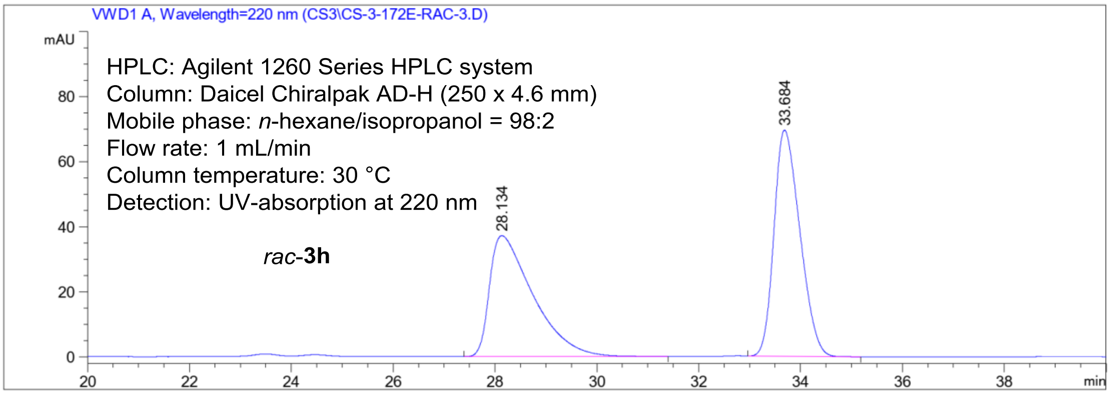

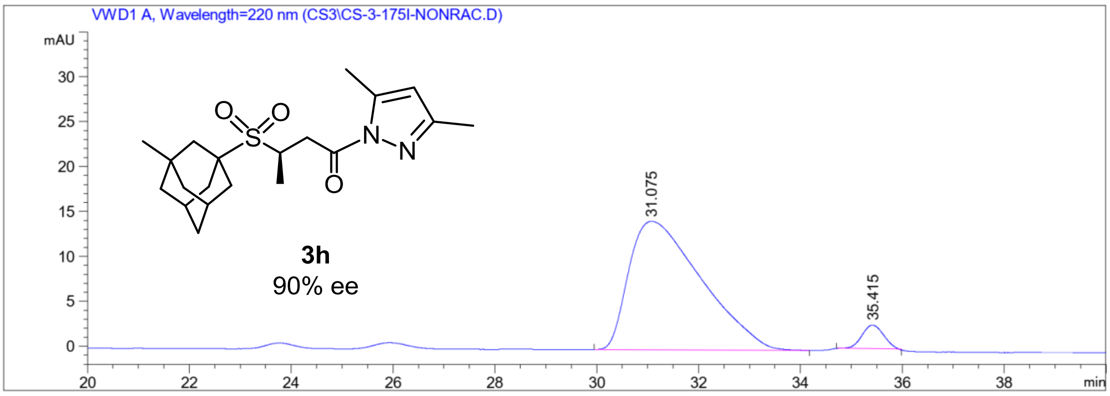

#	[min]		[min]	[mAU*s]	[mAU]	%
1	31.075	BB	1.3334	1413.20337	14.29408	94.8184
2	35.415	MM R	0.4898	77.22892	2.62801	5.1816

Supplementary Fig. 20 HPLC trace for the racemic reference *rac*-**3h**, and non-racemic product **3h** generated from the photocatalytic asymmetric reaction.

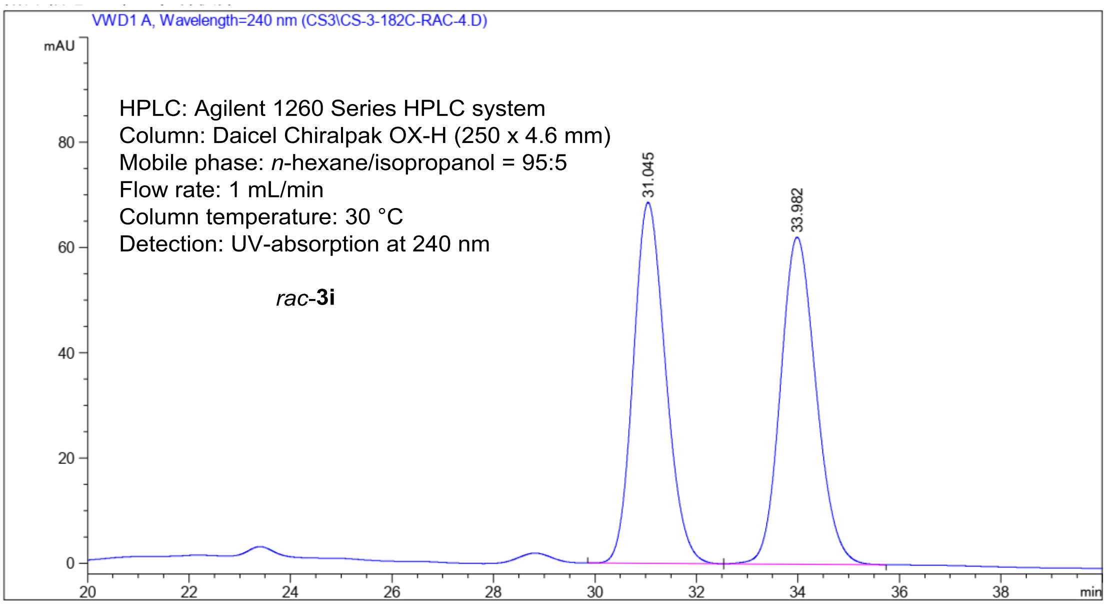

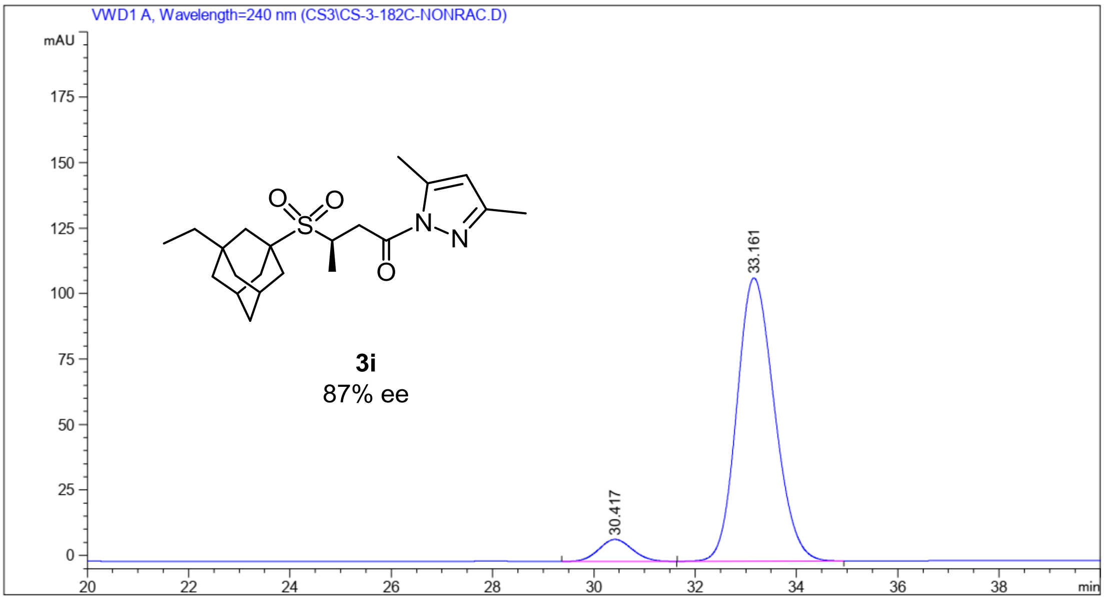

#	[min]		[min]	[mAU*s]	[mAU]	%
1	30.417	BB	0.7021	385.03928	8.39539	6.4858
2	33.161	BB	0.7991	5551.60156	108.11236	93.5142

Supplementary Fig. 21 HPLC trace for the racemic reference *rac*-**3i**, and non-racemic product **3i** generated from the photocatalytic asymmetric reaction.

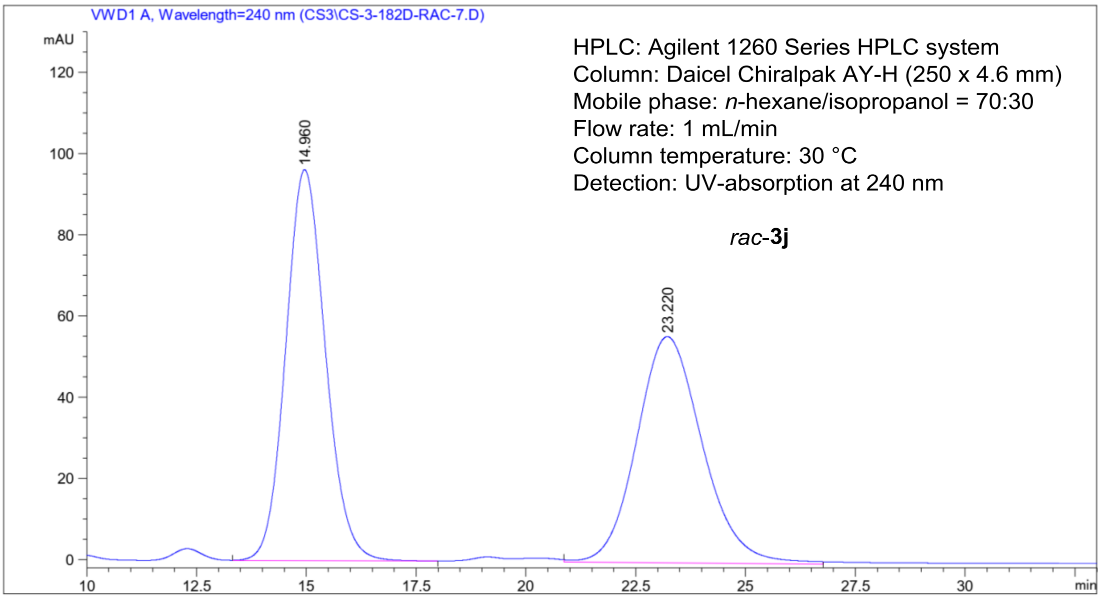

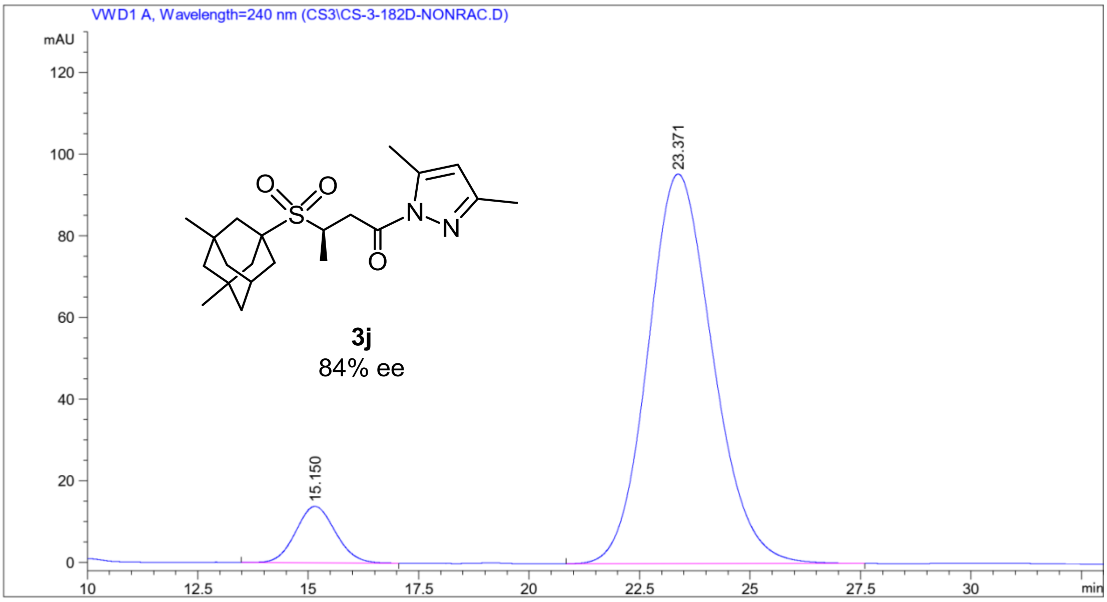

#	[min]		[min]	[mAU*s]	[mAU]	%
1	15.150	BB	0.9479	868.27820	13.84143	8.1849
2	23.371	BB	1.5351	9739.96582	95.40483	91.8151

Supplementary Fig. 22 HPLC trace for the racemic reference *rac*-**3j**, and non-racemic product **3j** generated from the photocatalytic asymmetric reaction.

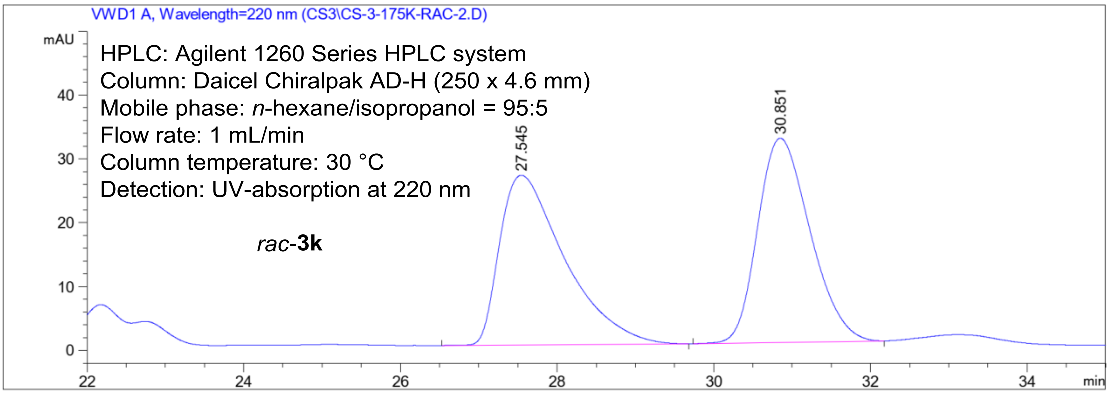

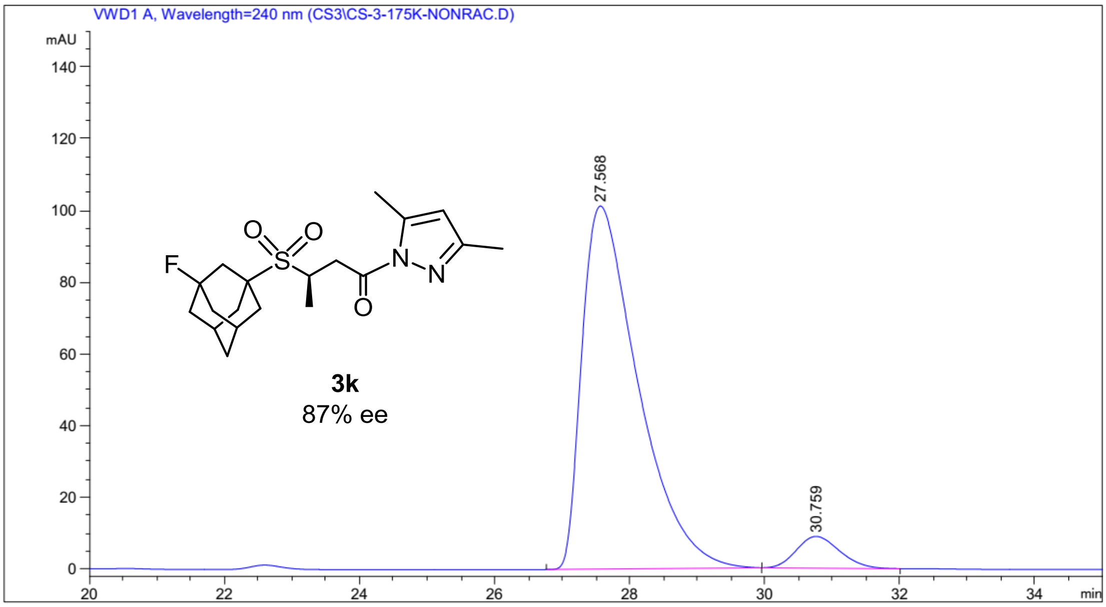

#	[min]		[min]	[mAU*s]	[mAU]	%
1	27.568	BB	0.8688	5836.25439	101.20635	93.7123
2	30.759	BB	0.6864	391.58847	8.83040	6.2877

Supplementary Fig. 23 HPLC trace for the racemic reference *rac*-**3k**, and non-racemic product **3k** generated from the photocatalytic asymmetric reaction.

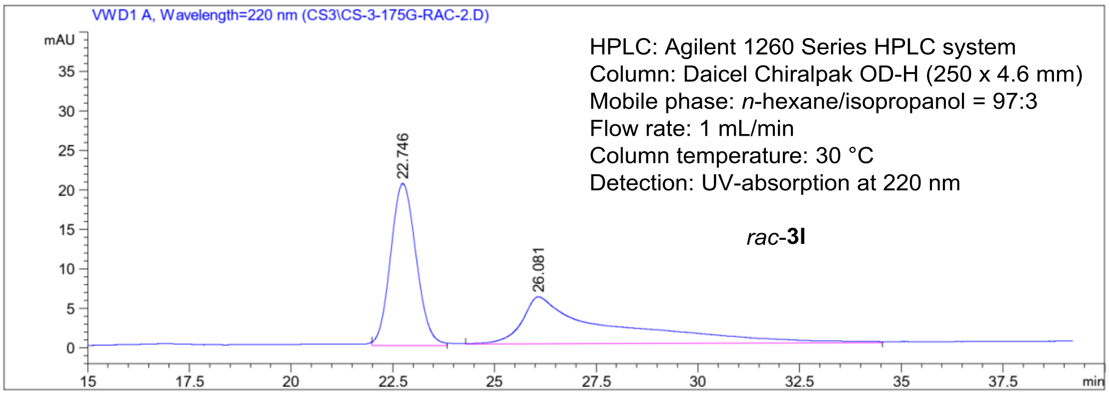

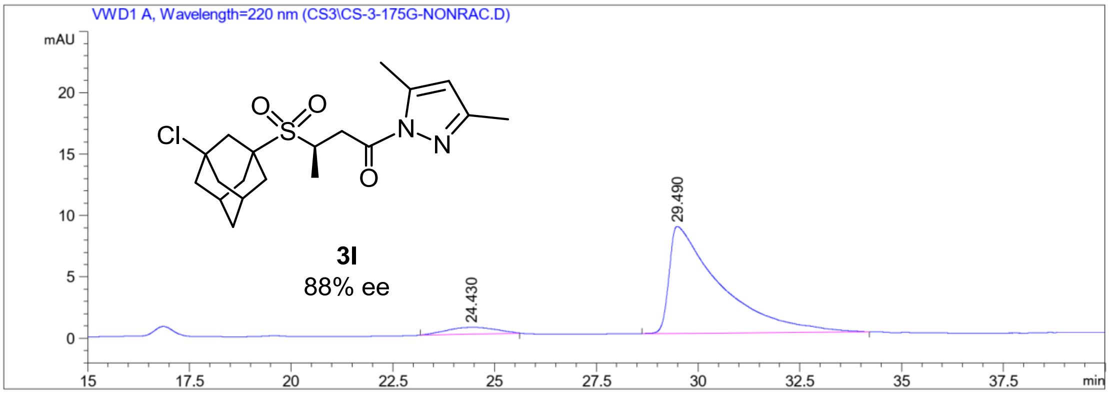

#	[min]		[min]	[mAU*s]	[mAU]	%
1	24.430	MM R	1.3861	46.99101	5.65008e-1	5.8343
2	29.490	BB	1.1178	758.44202	8.71081	94.1657

Supplementary Fig. 24 HPLC trace for the racemic reference *rac*-**3l**, and non-racemic product **3l** generated from the photocatalytic asymmetric reaction.

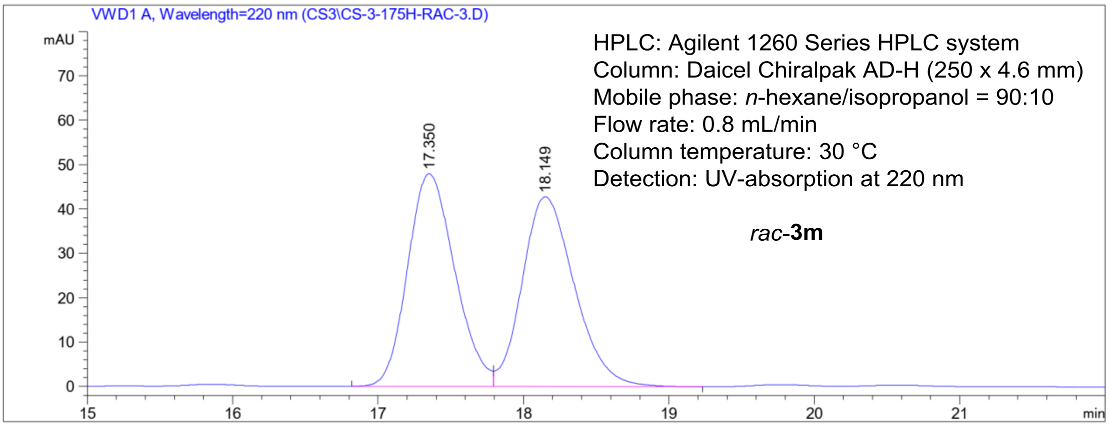

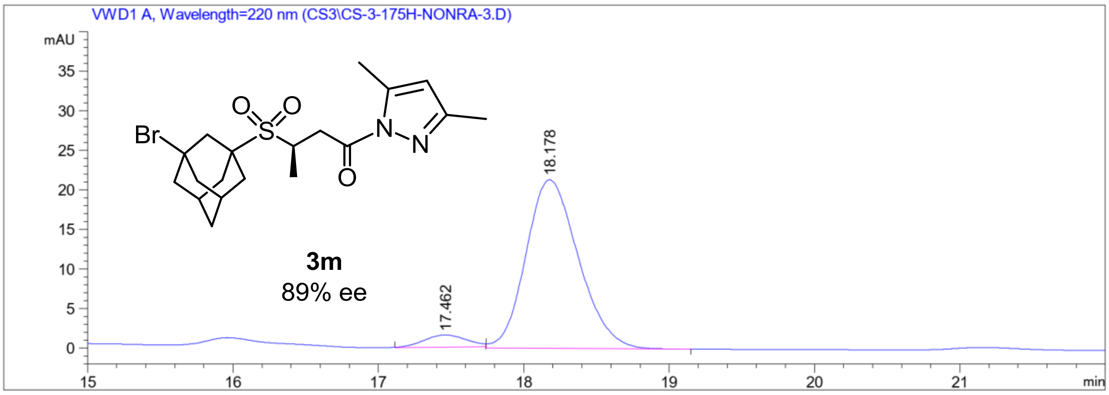

#	[min]		[min]	[mAU*s]	[mAU]	%
1	17.462	MM R	0.3450	32.22387	1.55682	5.6864
2	18.178	VB	0.3875	534.45874	21.31617	94.3136

Supplementary Fig. 25 HPLC trace for the racemic reference *rac*-**3m**, and non-racemic product **3m** generated from the photocatalytic asymmetric reaction.

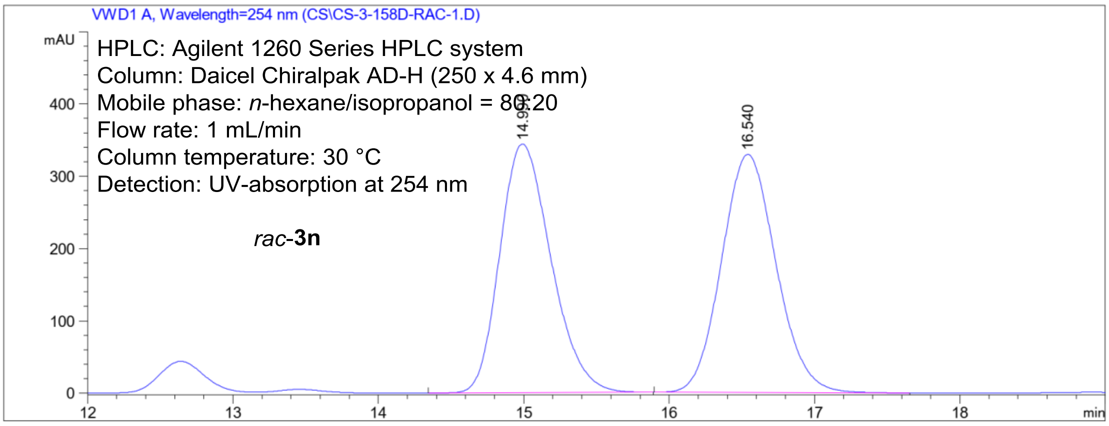

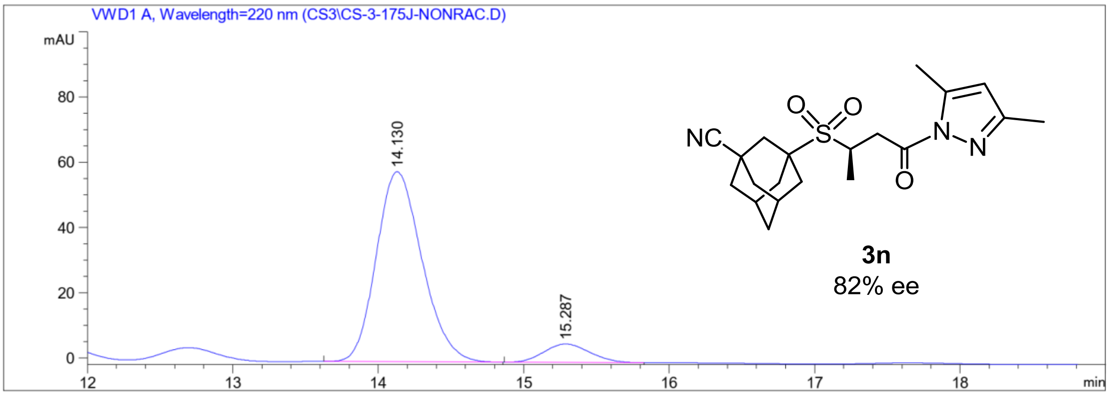

#	[min]		[min]	[mAU*s]	[mAU]	%
1	14.130	BB	0.3385	1269.98743	58.22820	91.0407
2	15.287	BB	0.3434	124.97872	5.66427	8.9593

Supplementary Fig. 26 HPLC trace for the racemic reference *rac*-**3n**, and non-racemic product **3n** generated from the photocatalytic asymmetric reaction.

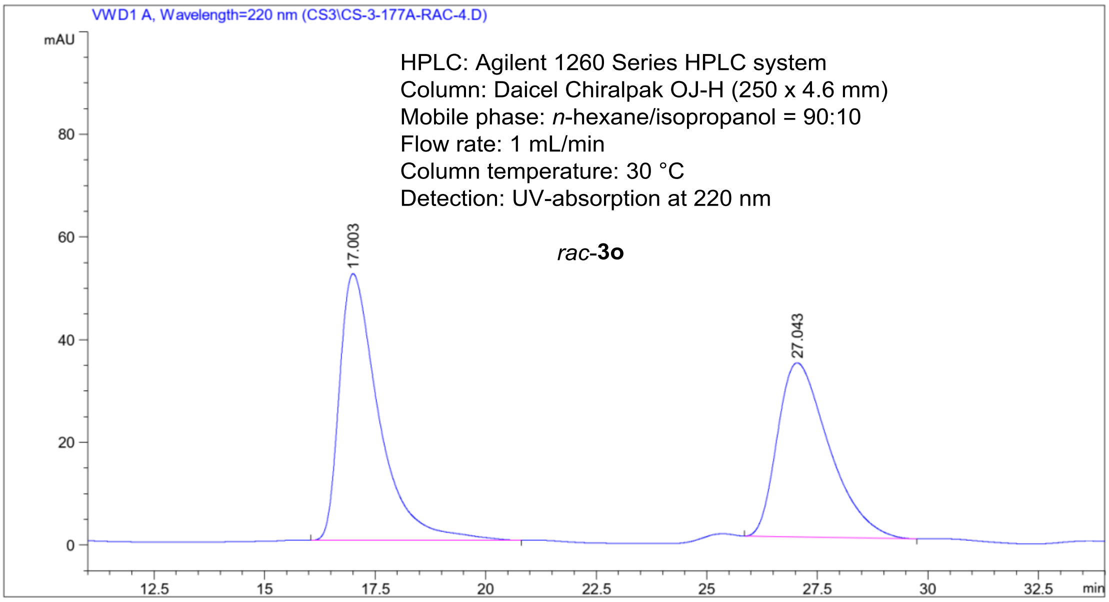

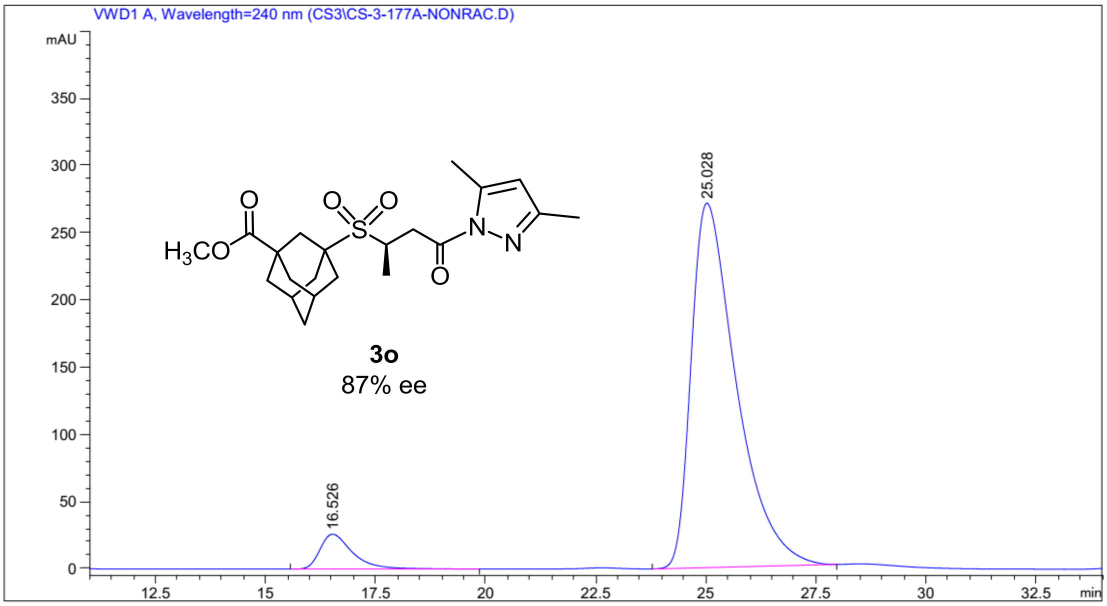

#	[min]		[min]	[mAU*s]	[mAU]	%
1	16.526	BB	0.7712	1338.88245	25.93811	6.4864
2	25.028	BB	1.0658	1.93024e4	271.05243	93.5136

Supplementary Fig. 27 HPLC trace for the racemic reference *rac*-**3o**, and non-racemic product **3o** generated from the photocatalytic asymmetric reaction.

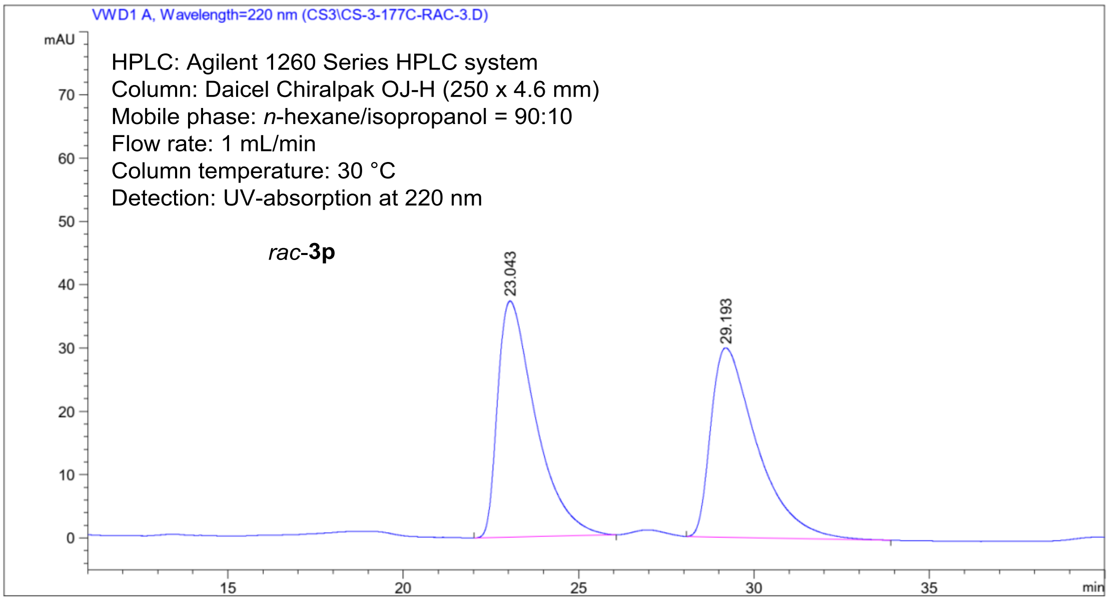

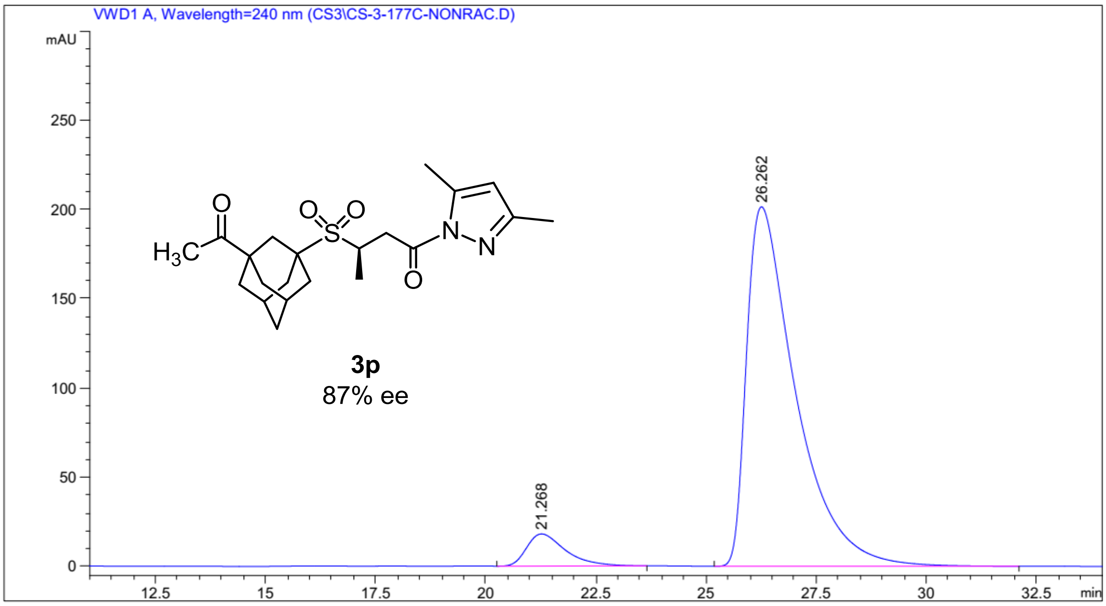

#	[min]		[min]	[mAU*s]	[mAU]	%
1	21.268	BB	0.9044	1099.07764	17.99841	6.5645
2	26.262	BB	1.1573	1.56437e4	201.38492	93.4355

Supplementary Fig. 28 HPLC trace for the racemic reference *rac*-**3p**, and non-racemic product **3p** generated from the photocatalytic asymmetric reaction.

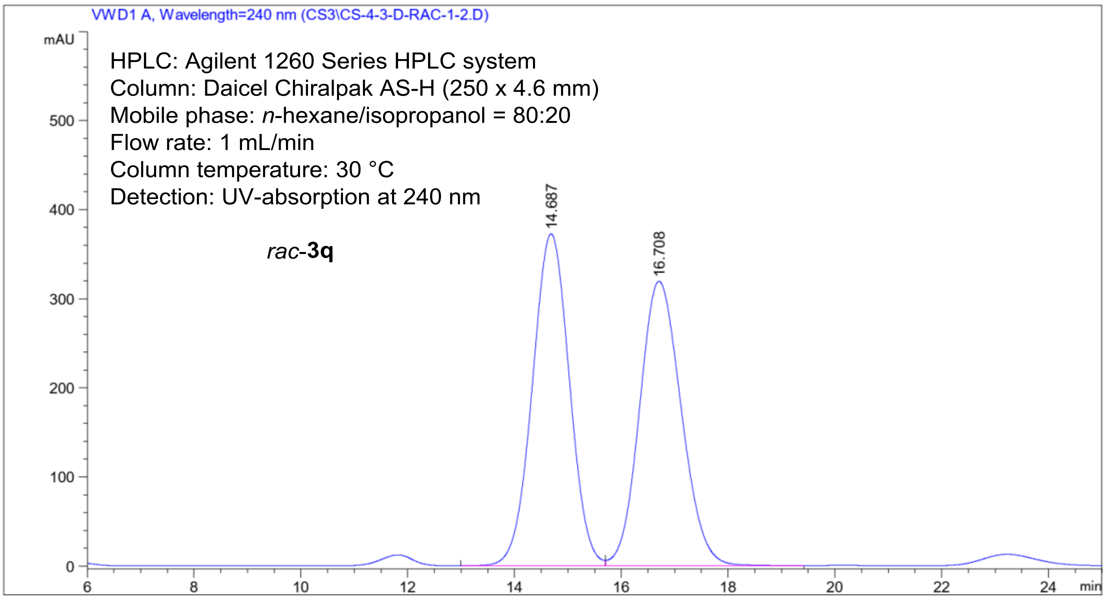

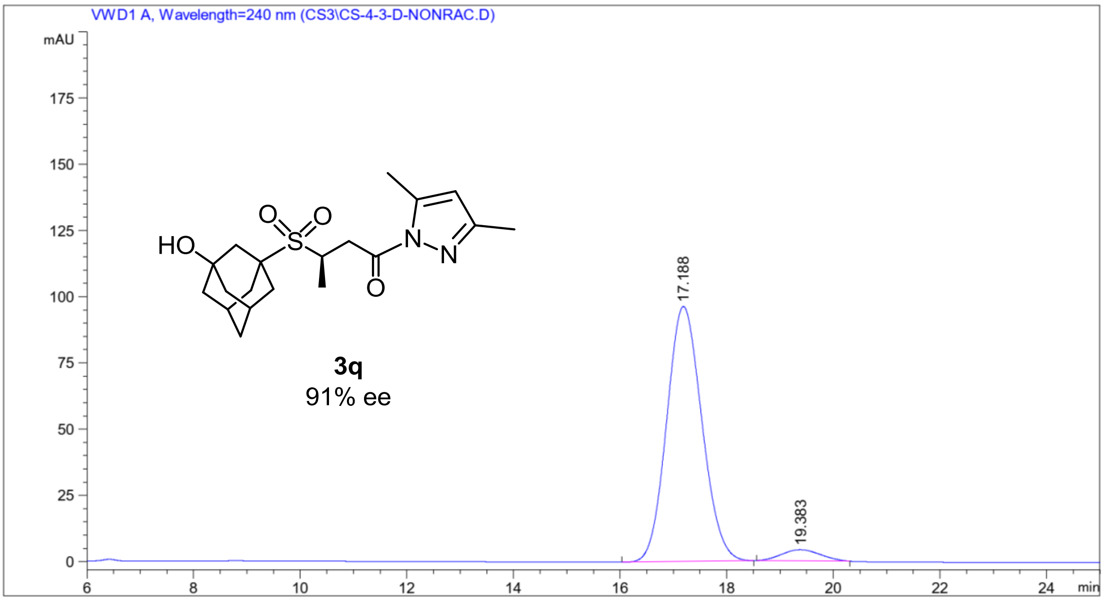

#	[min]		[min]	[mAU*s]	[mAU]	%
1	17.188	BB	0.7149	4400.11035	96.15068	95.3723
2	19.383	MM R	0.8421	213.50354	4.22569	4.6277

Supplementary Fig. 29 HPLC trace for the racemic reference *rac*-**3q**, and non-racemic product **3q** generated from the photocatalytic asymmetric reaction.

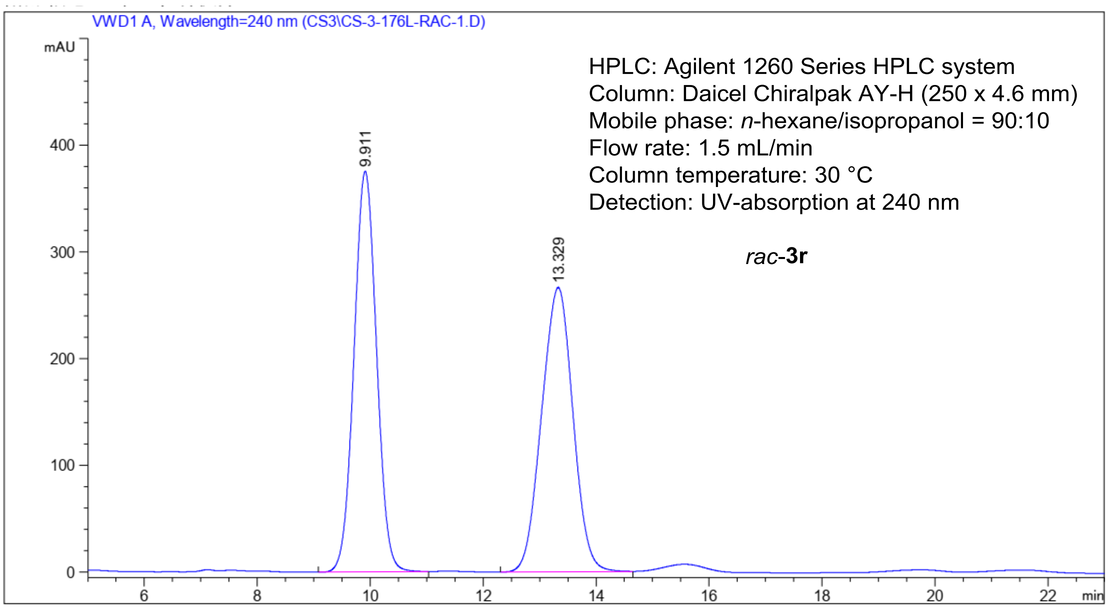

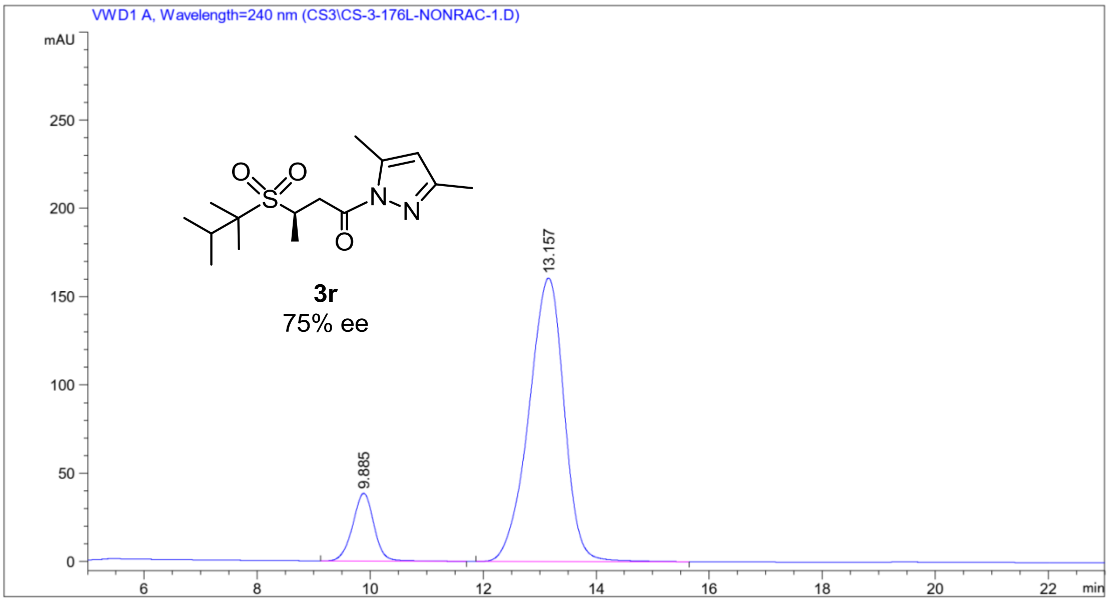

#	[min]		[min]	[mAU*s]	[mAU]	%
1	9.885	BB	0.4010	1003.97028	38.27881	12.6255
2	13.157	BB	0.6742	6947.93799	160.45760	87.3745

Supplementary Fig. 30 HPLC trace for the racemic reference *rac*-**3r**, and non-racemic product **3r** generated from the photocatalytic asymmetric reaction.

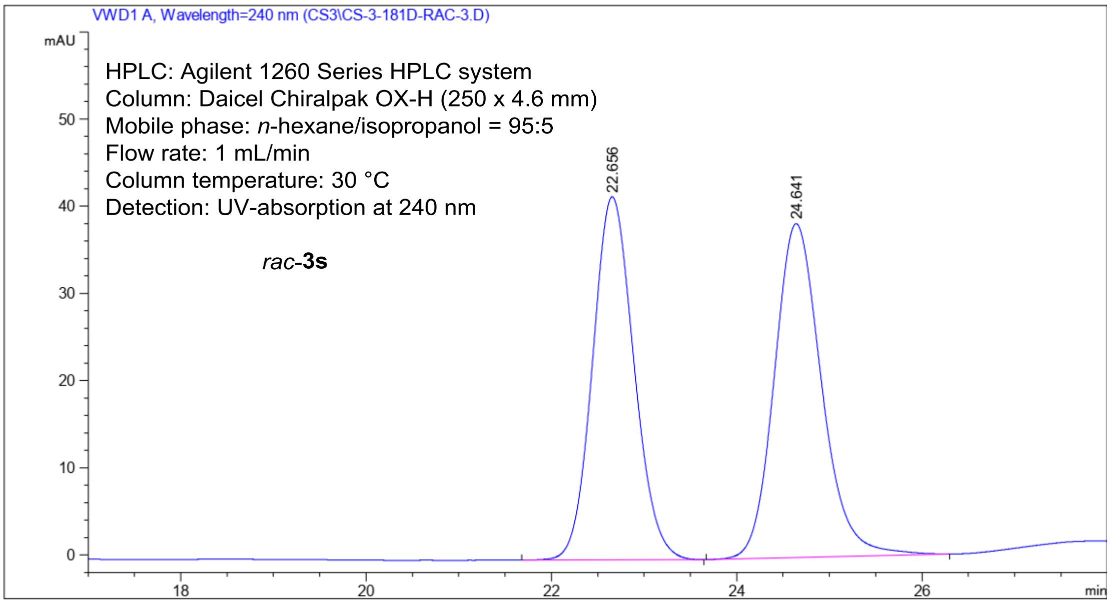

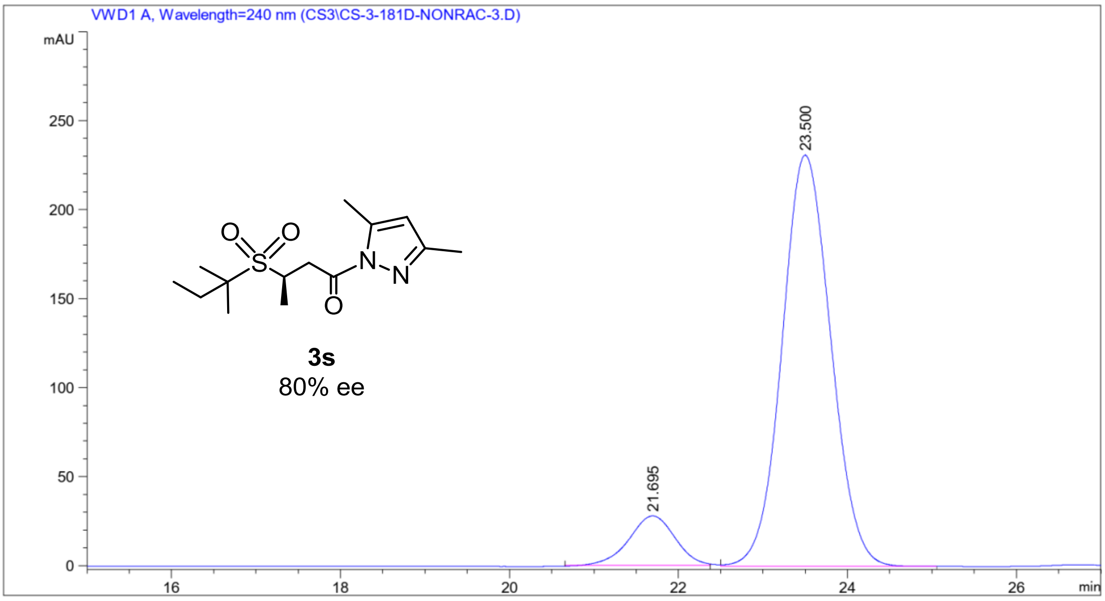

#	[min]		[min]	[mAU*s]	[mAU]	%
1	21.695	MM R	0.6261	1038.96924	27.65536	10.0875
2	23.500	VB	0.6257	9260.60840	231.00209	89.9125

Supplementary Fig. 31 HPLC trace for the racemic reference *rac*-**3s**, and non-racemic product **3s** generated from the photocatalytic asymmetric reaction.

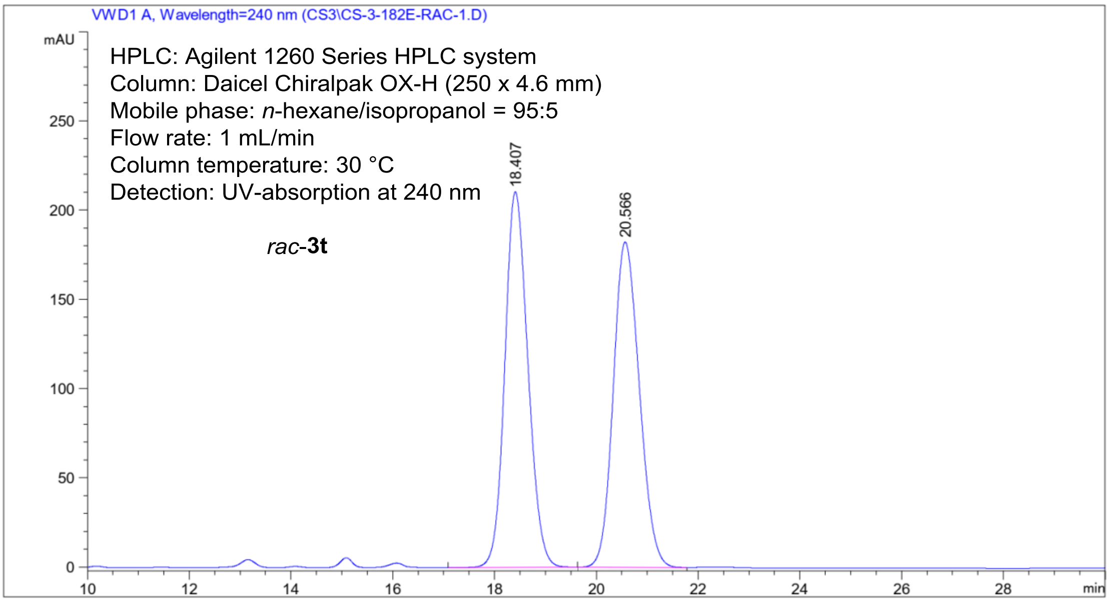

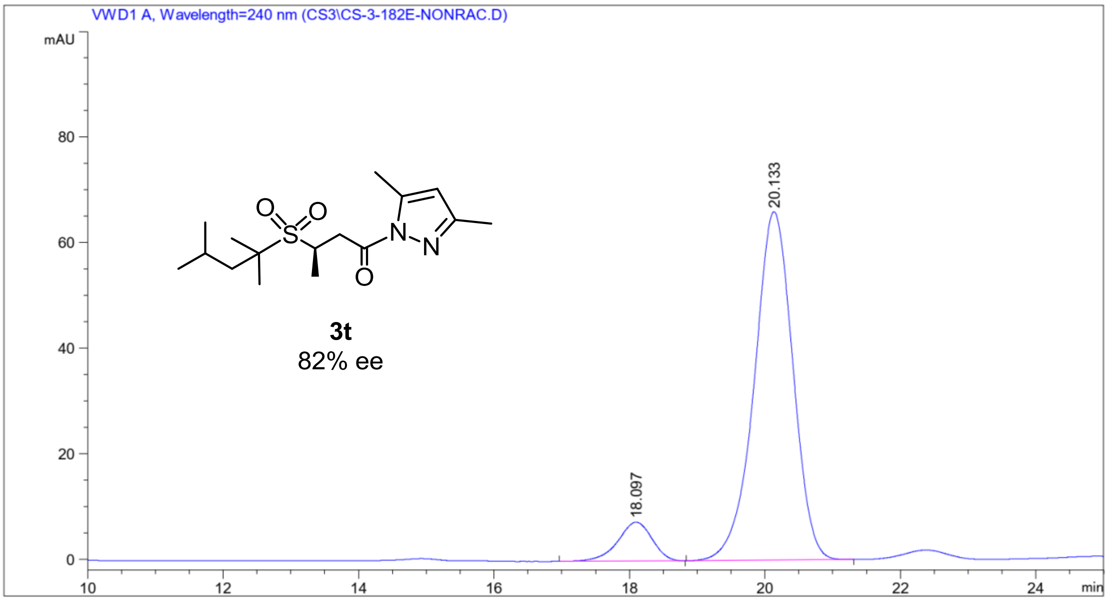

#	[min]		[min]	[mAU*s]	[mAU]	%
1	18.097	BB	0.5368	259.71500	7.35776	9.0360
2	20.133	BB	0.6121	2614.52319	66.00646	90.9640

Supplementary Fig. 32 HPLC trace for the racemic reference *rac*-**3t**, and non-racemic product **3t** generated from the photocatalytic asymmetric reaction.

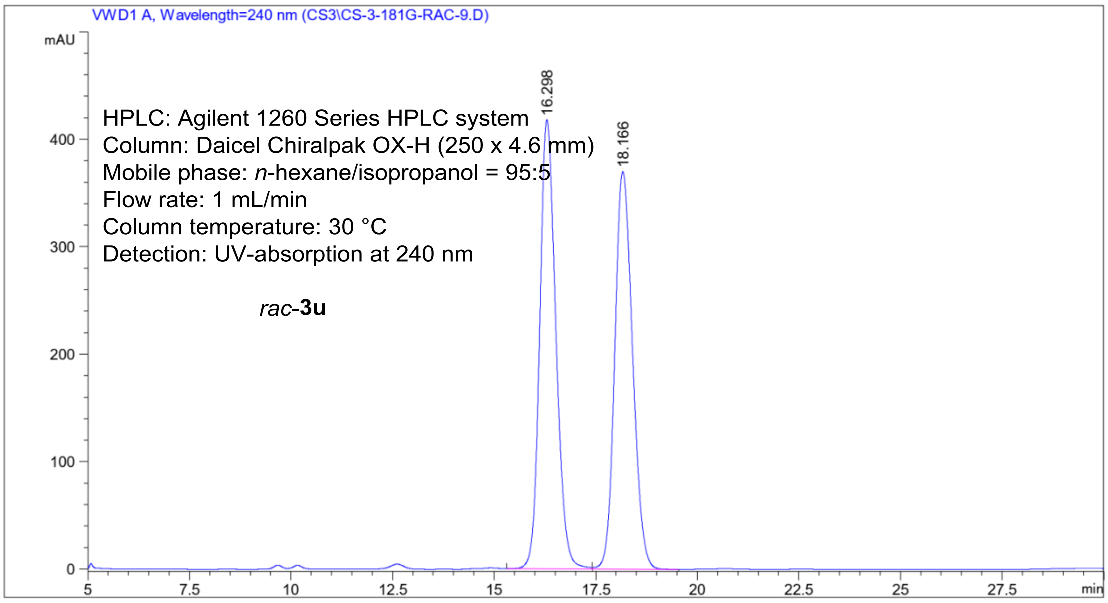

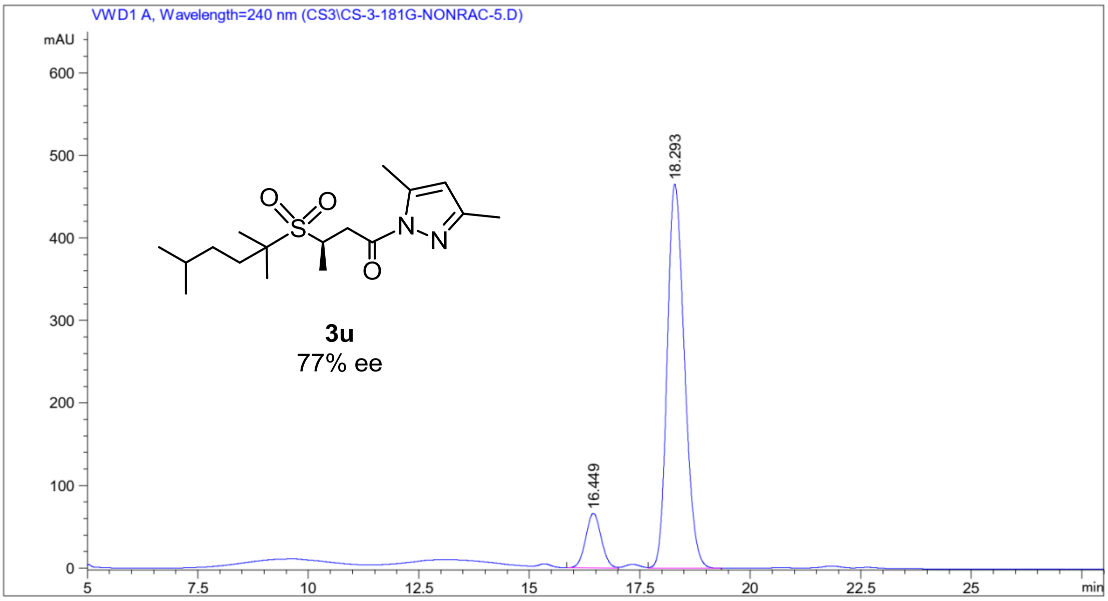

#	[min]		[min]	[mAU*s]	[mAU]	%
1	16.449	BV	0.3776	1608.11755	66.15073	11.3287
2	18.293	VB	0.4217	1.25870e4	465.15942	88.6713

Supplementary Fig. 33 HPLC trace for the racemic reference *rac*-**3u**, and non-racemic product **3u** generated from the photocatalytic asymmetric reaction.

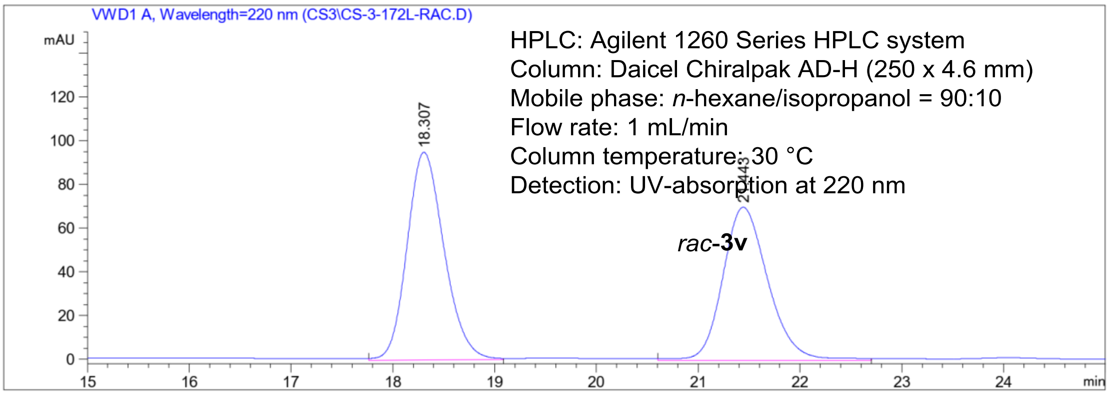

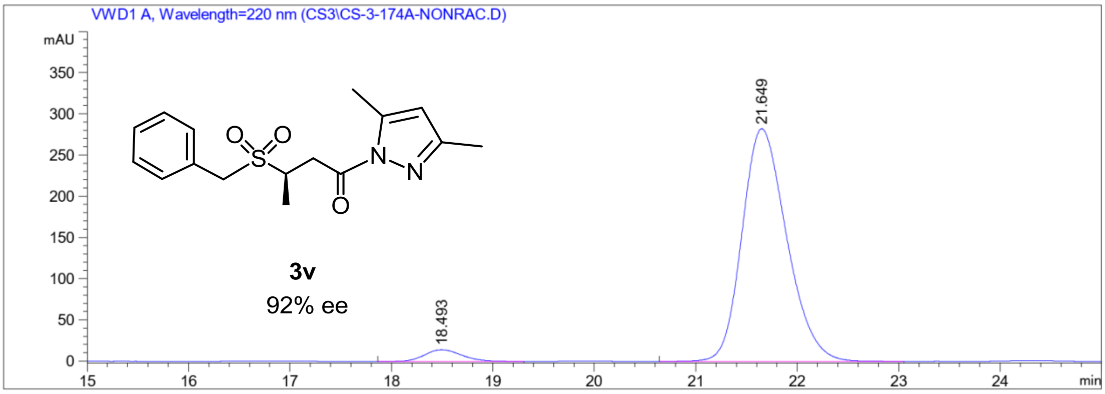

#	[min]		[min]	[mAU*s]	[mAU]	%
1	11.311	BB	0.3386	4113.02832	186.24892	96.5877
2	17.775	BB	0.5027	145.30856	4.41825	3.4123

Supplementary Fig. 34 HPLC trace for the racemic reference *rac*-**3v**, and non-racemic product **3v** generated from the photocatalytic asymmetric reaction.

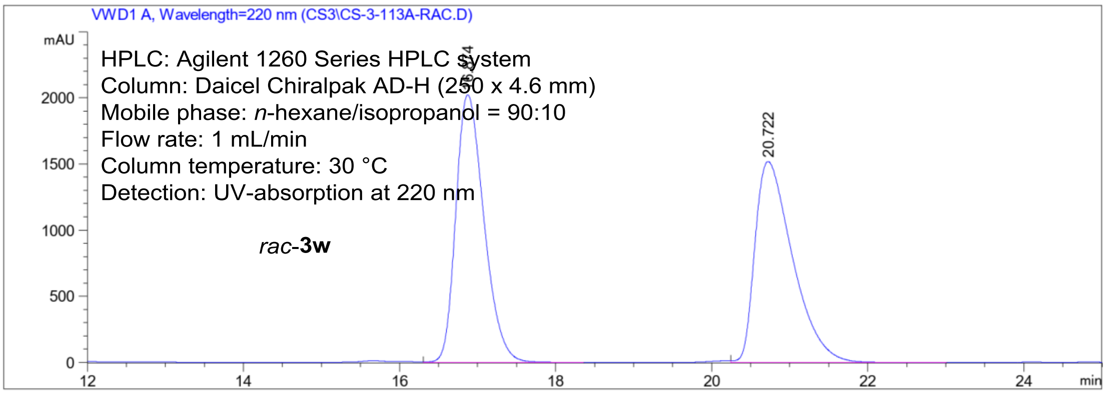

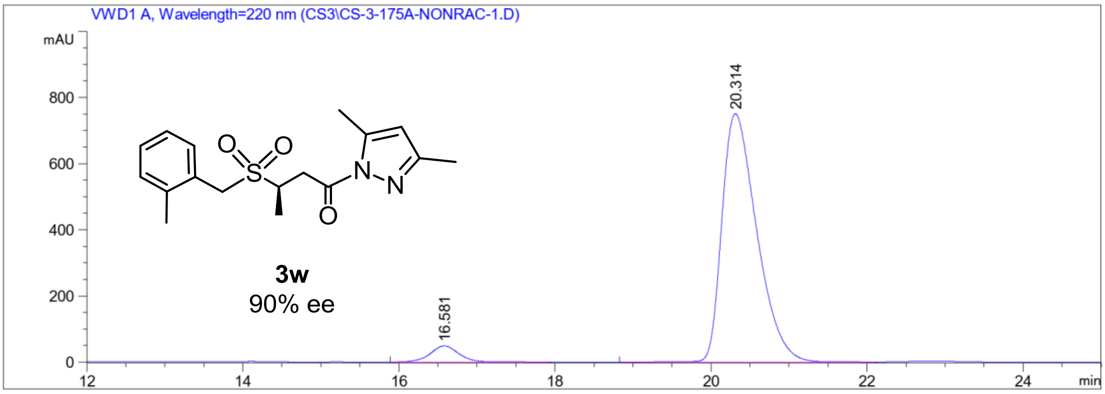

#	[min]		[min]	[mAU*s]	[mAU]	%
1	16.581	BB	0.3830	1228.69946	48.42366	5.0242
2	20.314	BB	0.4761	2.32267e4	750.35443	94.9758

Supplementary Fig. 35 HPLC trace for the racemic reference *rac*-**3w**, and non-racemic product **3w** generated from the photocatalytic asymmetric reaction.

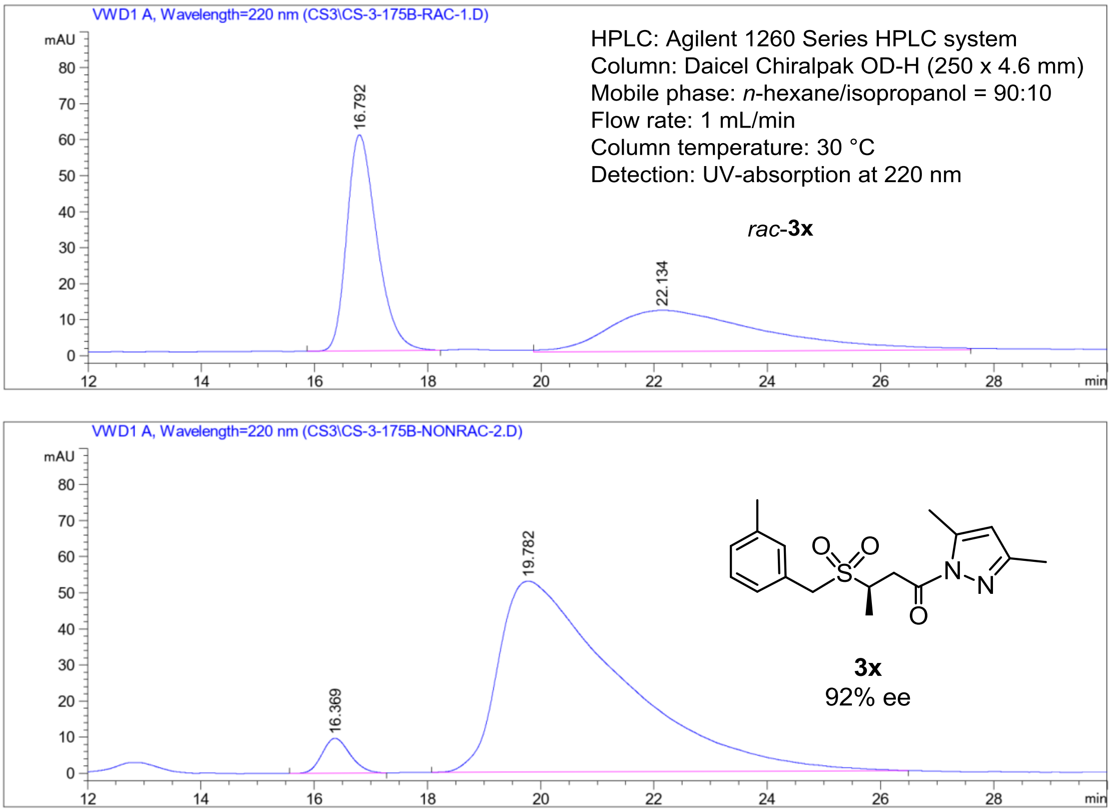

#	[min]		[min]	[mAU*s]	[mAU]	%
1	16.369	BB	0.5338	331.53140	9.65048	4.0089
2	19.782	BB	1.9873	7938.29590	52.88121	95.9911

Supplementary Fig. 36 HPLC trace for the racemic reference *rac*-**3x**, and non-racemic product **3x** generated from the photocatalytic asymmetric reaction.

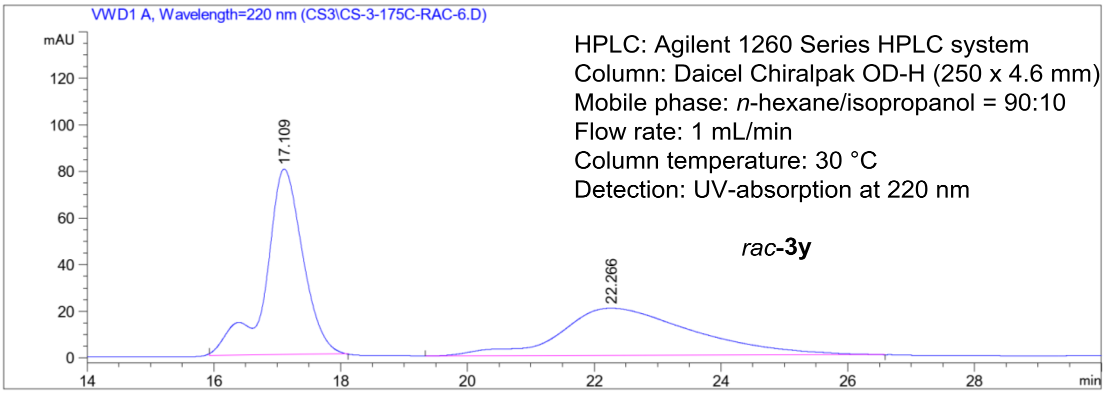

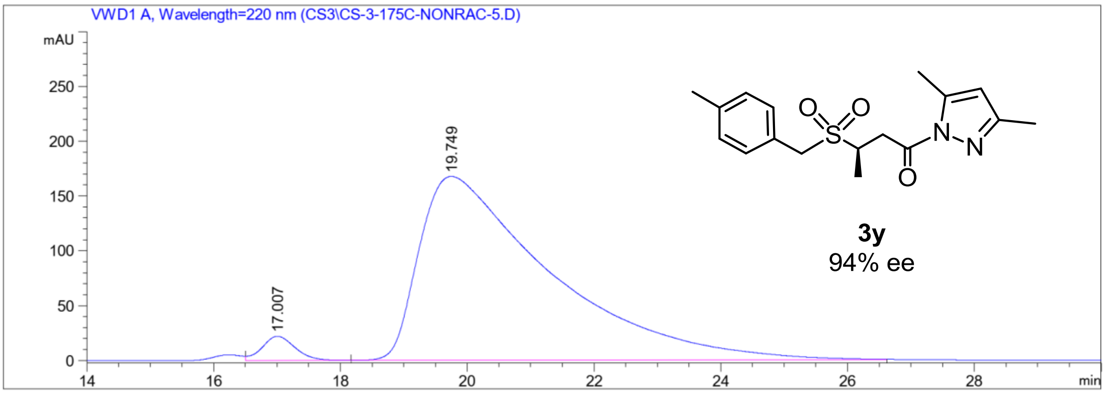

#	[min]		[min]	[mAU*s]	[mAU]	%
1	17.007	VB	0.5543	797.00940	22.06990	3.2058
2	19.749	BV	1.9672	2.40643e4	167.59036	96.7942

Supplementary Fig. 37 HPLC trace for the racemic reference *rac*-**3y**, and non-racemic product **3y** generated from the photocatalytic asymmetric reaction.

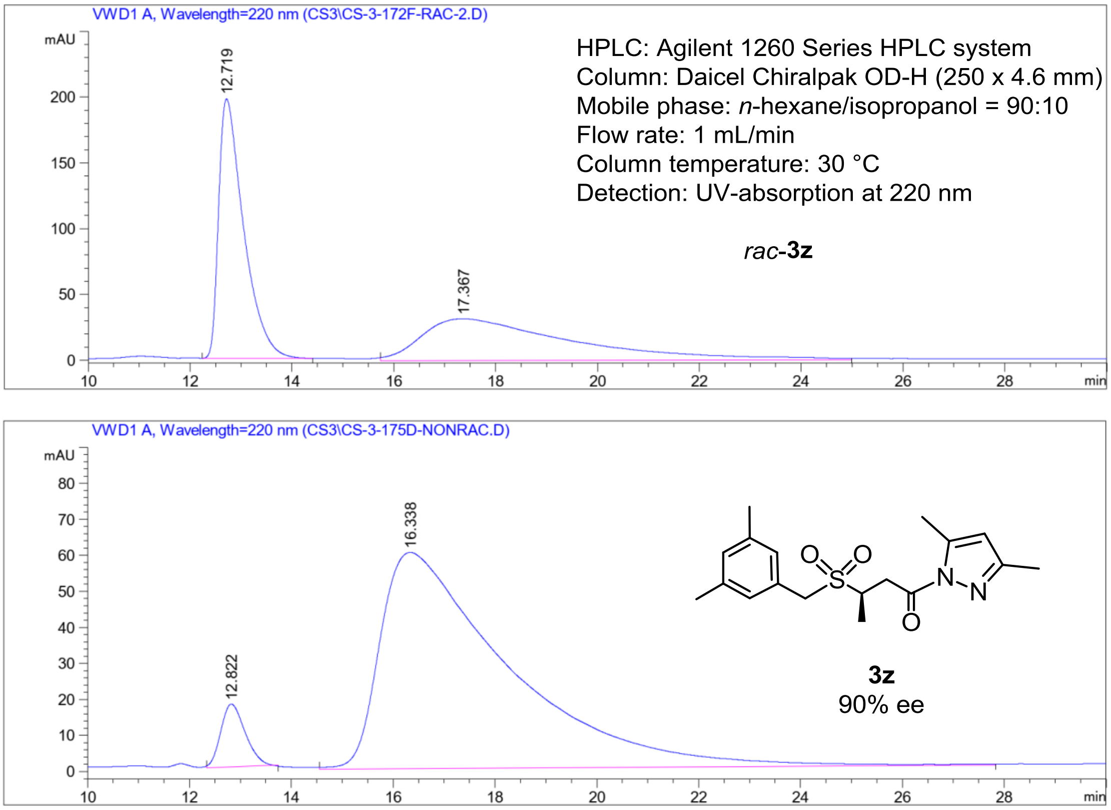

#	[min]		[min]	[mAU*s]	[mAU]	%
1	12.822	MM R	0.5544	580.72504	17.45671	5.2413
2	16.338	MM R	2.9168	1.04991e4	59.99216	94.7587

Supplementary Fig. 38 HPLC trace for the racemic reference *rac*-**3z**, and non-racemic product **3z** generated from the photocatalytic asymmetric reaction.

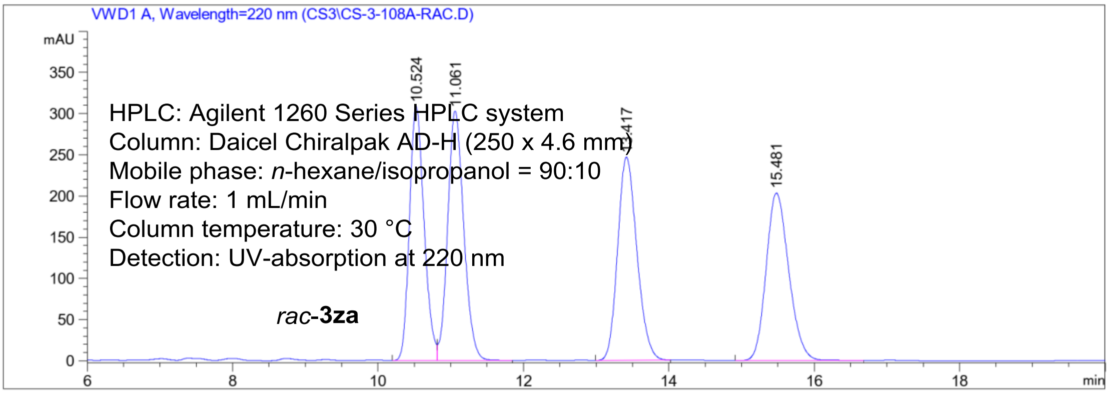

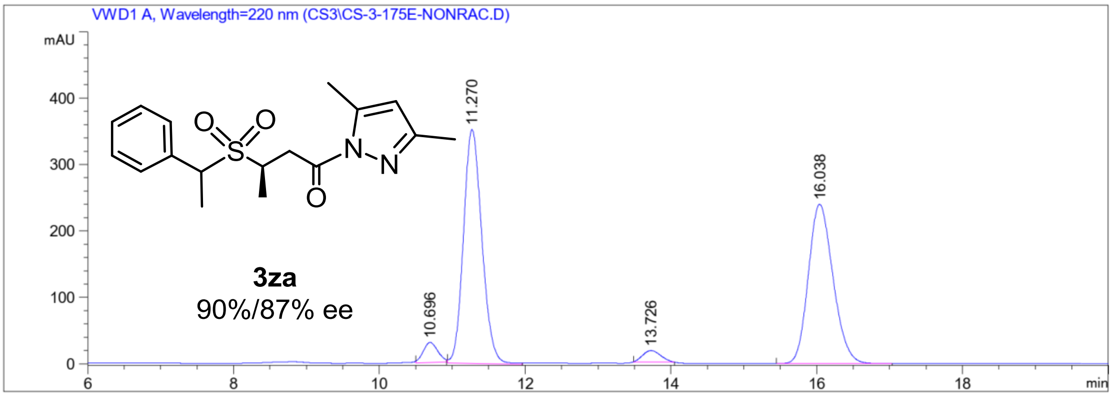

#	[min]		[min]	[mAU*s]	[mAU]	%
1	10.696	MM R	0.2318	422.46368	30.37390	3.3688
2	11.270	MM R	0.2927	6184.50488	352.09500	49.3165
3	13.726	MM R	0.2828	292.99478	17.26727	2.3364
4	16.038	BB	0.3645	5640.46191	239.69688	44.9782

Supplementary Fig. 39 HPLC trace for the racemic reference *rac*-**3za**, and non-racemic product **3za** generated from the photocatalytic asymmetric reaction.

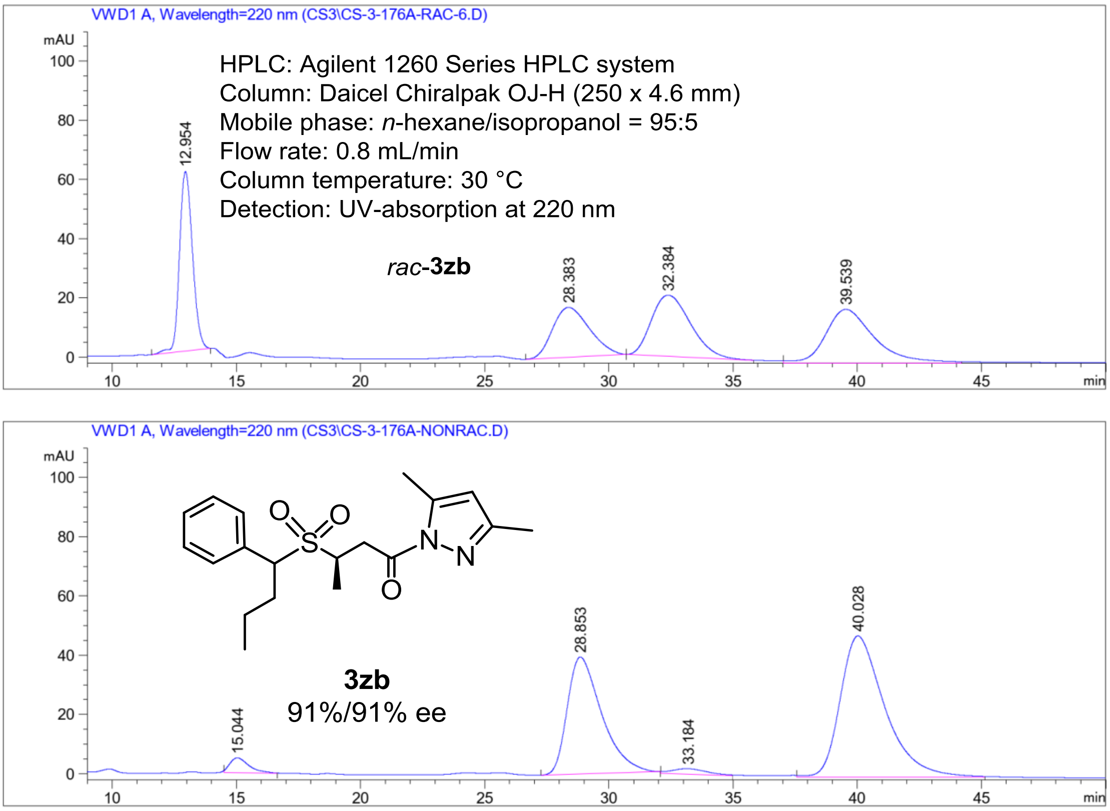

#	[min]		[min]	[mAU*s]	[mAU]	%
1	15.044	MM R	0.9051	269.38443	4.96059	2.6469
2	28.853	BB	1.4152	3802.69653	39.49795	37.3637
3	33.184	MM R	1.6511	188.58760	1.90360	1.8530
4	40.028	MM R	2.0709	5916.83496	47.61801	58.1364

Supplementary Fig. 40 HPLC trace for the racemic reference *rac*-**3zb**, and non-racemic product **3zb** generated from the photocatalytic asymmetric reaction.

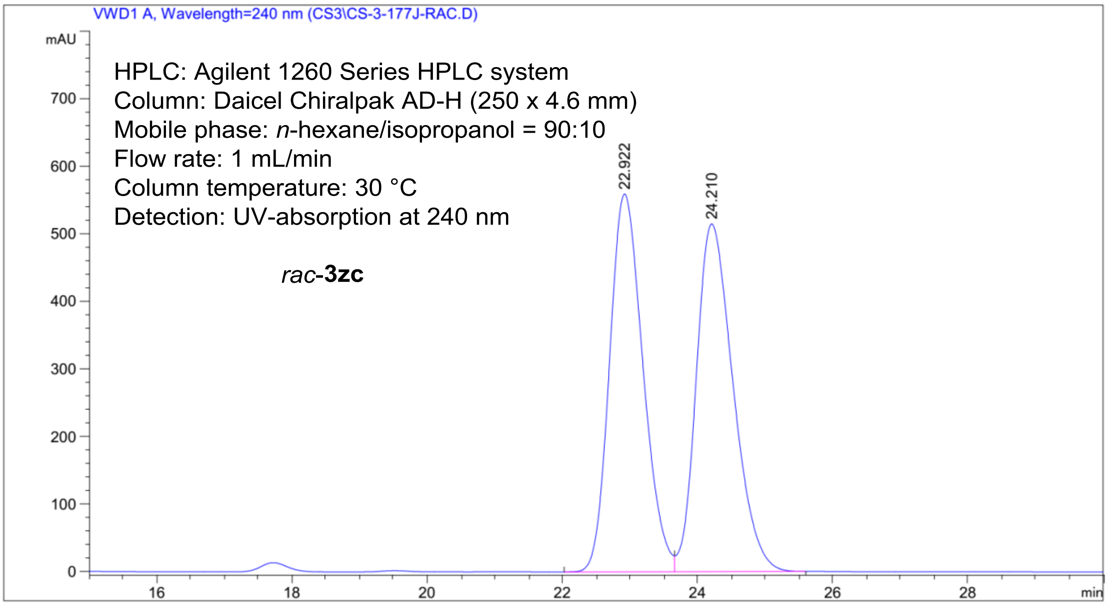

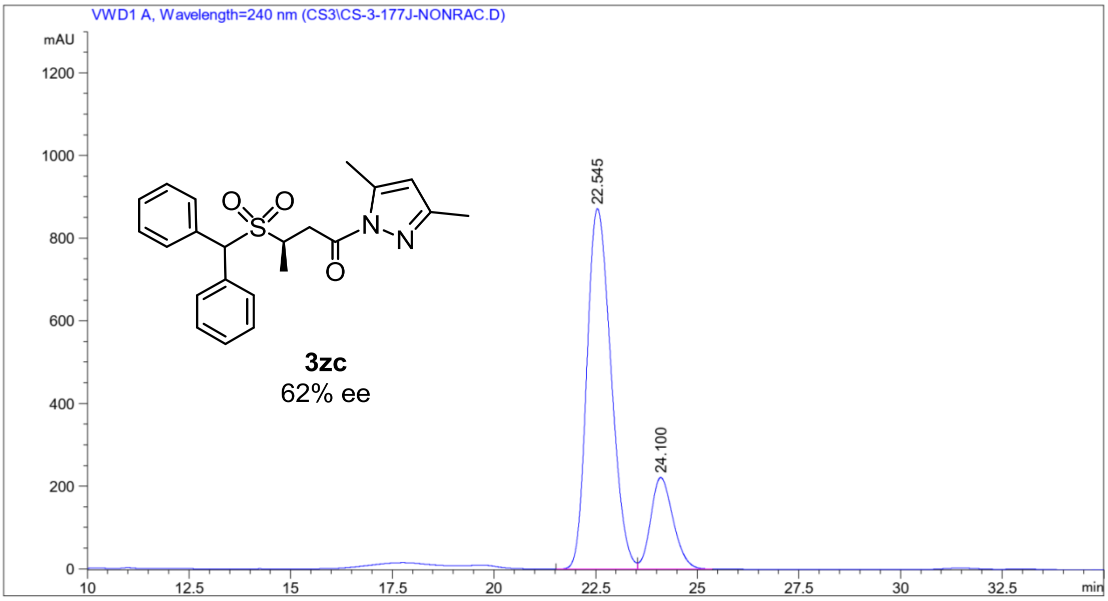

#	[min]		[min]	[mAU*s]	[mAU]	%
1	22.545	BV	0.6470	3.58829e4	872.21118	80.9112
2	24.100	VB	0.5912	8465.59277	221.36983	19.0888

Supplementary Fig. 41 HPLC trace for the racemic reference *rac*-**3zc**, and non-racemic product **3zc** generated from the photocatalytic asymmetric reaction.

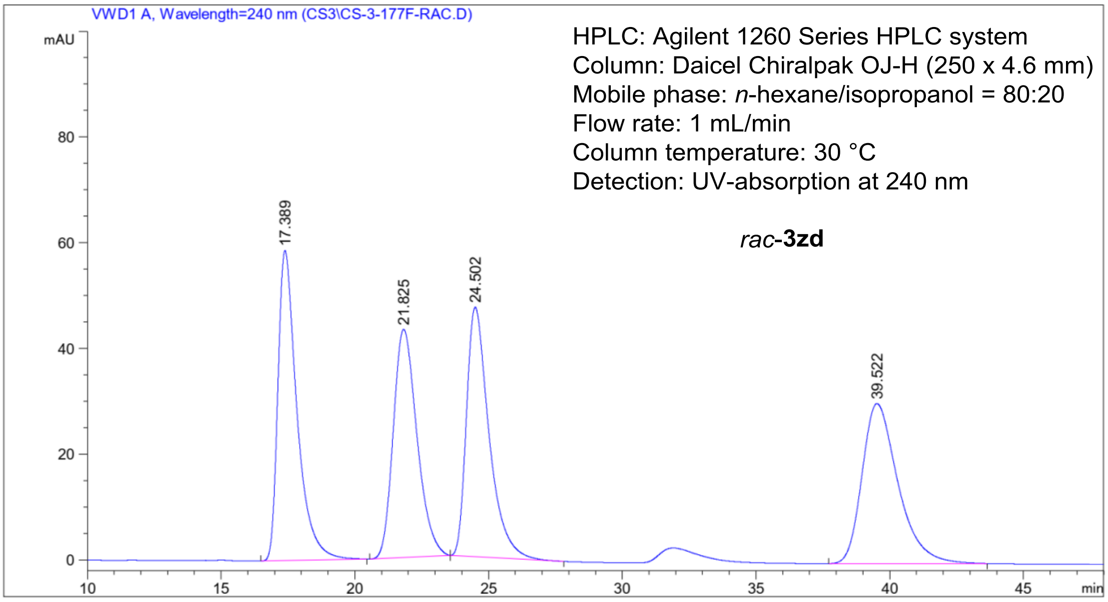

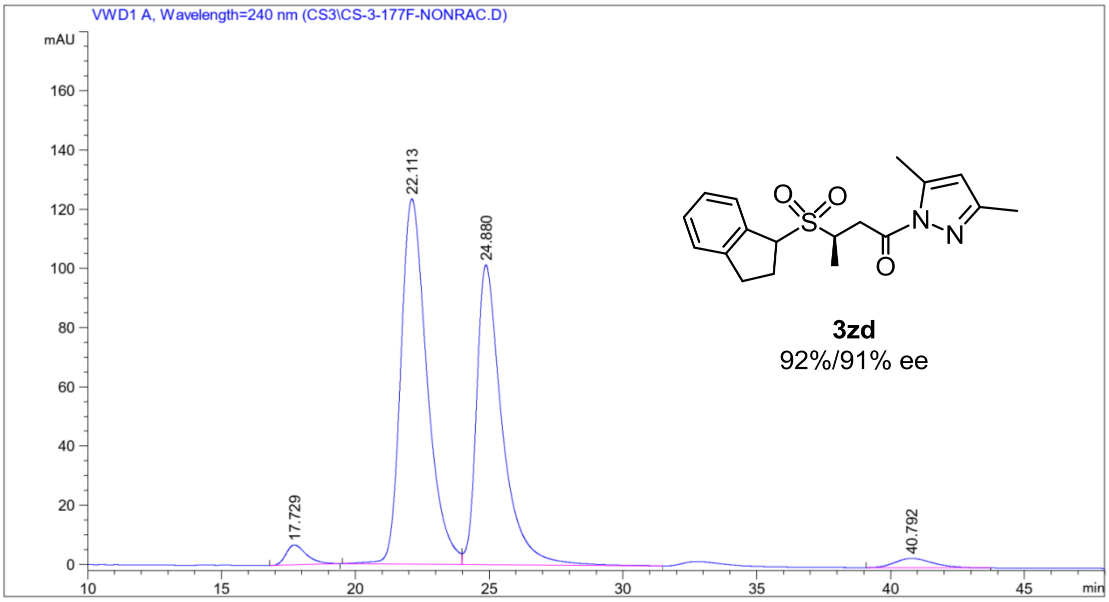

#	[min]		[min]	[mAU*s]	[mAU]	%
1	17.729	BB	0.7819	356.93219	6.74971	2.2595
2	22.113	BV	1.0381	8407.92480	123.36489	53.2247
3	24.880	VB	0.9803	6720.79102	101.26258	42.5447
4	40.792	BB	1.1591	311.37842	3.16136	1.9711

Supplementary Fig. 42 HPLC trace for the racemic reference *rac*-**3zd**, and non-racemic product **3zd** generated from the photocatalytic asymmetric reaction.

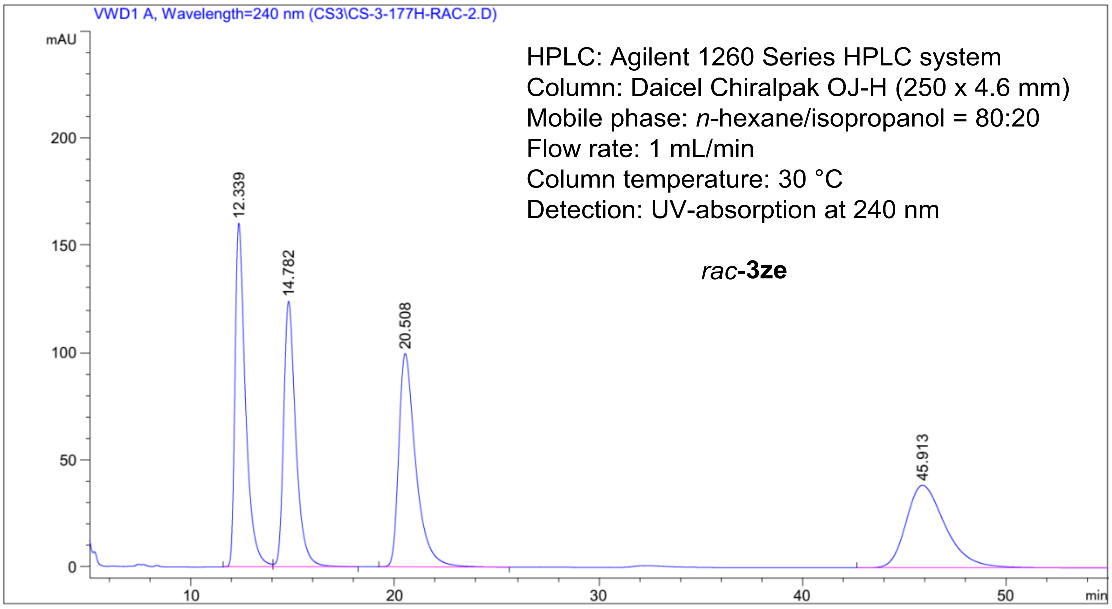

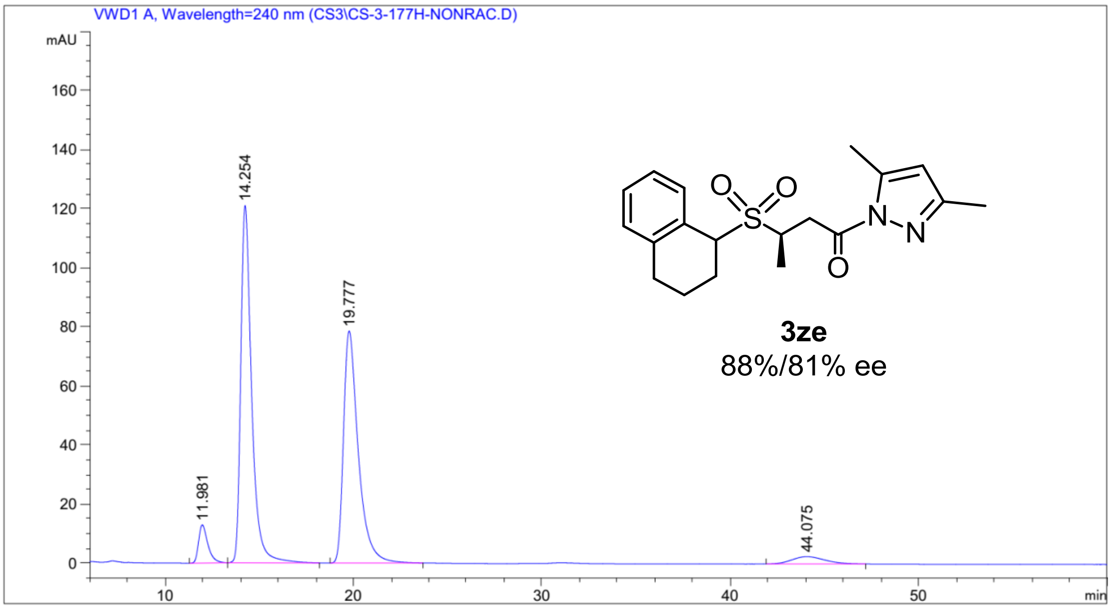

#	[min]		[min]	[mAU*s]	[mAU]	%
1	11.981	BB	0.5179	441.27225	12.90730	4.4775
2	14.254	BB	0.6006	4836.59424	120.92686	49.0760
3	19.777	BB	0.8105	4276.86328	78.51878	43.3965
4	44.075	BB	1.4320	300.58850	2.46782	3.0500

Supplementary Fig. 43 HPLC trace for the racemic reference *rac*-**3ze**, and non-racemic product **3ze** generated from the photocatalytic asymmetric reaction.

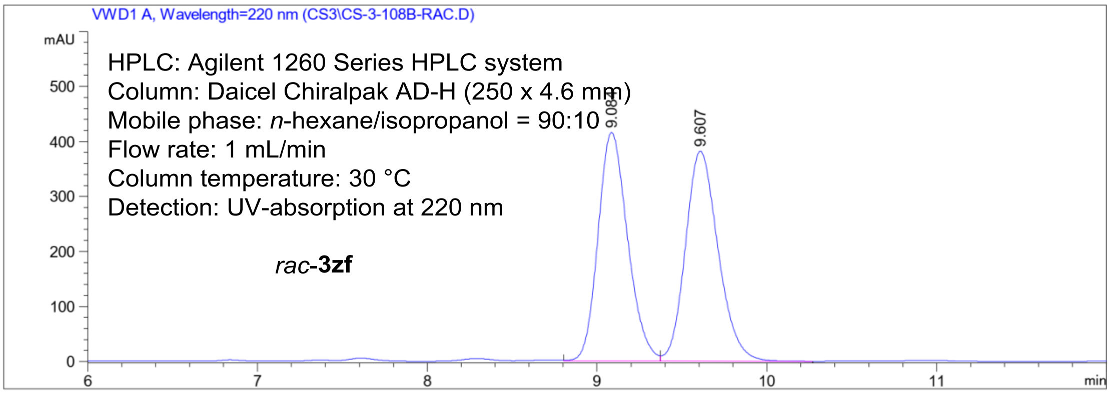

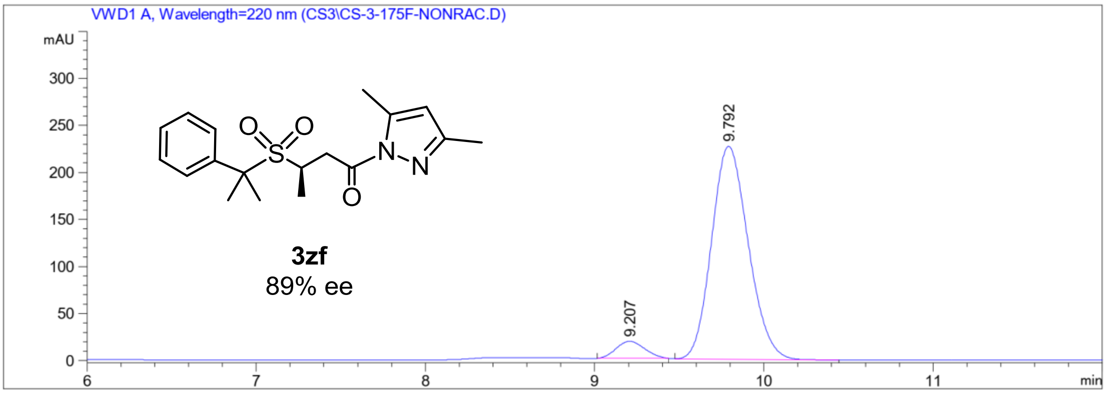

#	[min]		[min]	[mAU*s]	[mAU]	%
1	9.207	MM R	0.1928	207.37646	17.92649	5.7268
2	9.792	VB	0.2339	3413.80908	226.45361	94.2732

Supplementary Fig. 44 HPLC trace for the racemic reference *rac*-**3zf**, and non-racemic product **3zf** generated from the photocatalytic asymmetric reaction.

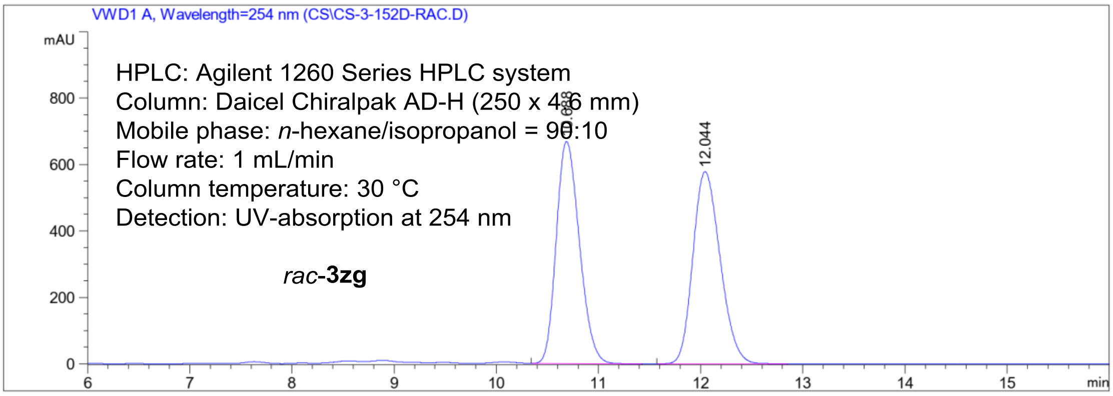

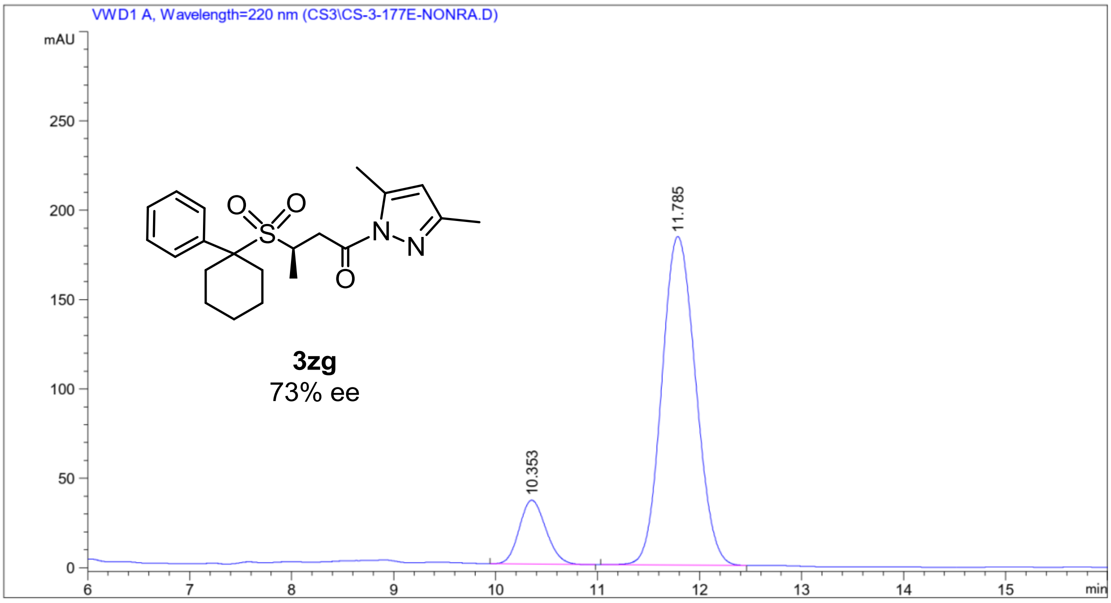

#	[min]		[min]	[mAU*s]	[mAU]	%
1	10.353	BB	0.2834	647.37146	35.72091	13.2620
2	11.785	BB	0.3637	4234.01172	183.83789	86.7380

Supplementary Fig. 45 HPLC trace for the racemic reference *rac*-**3zg**, and non-racemic product **3zg** generated from the photocatalytic asymmetric reaction.

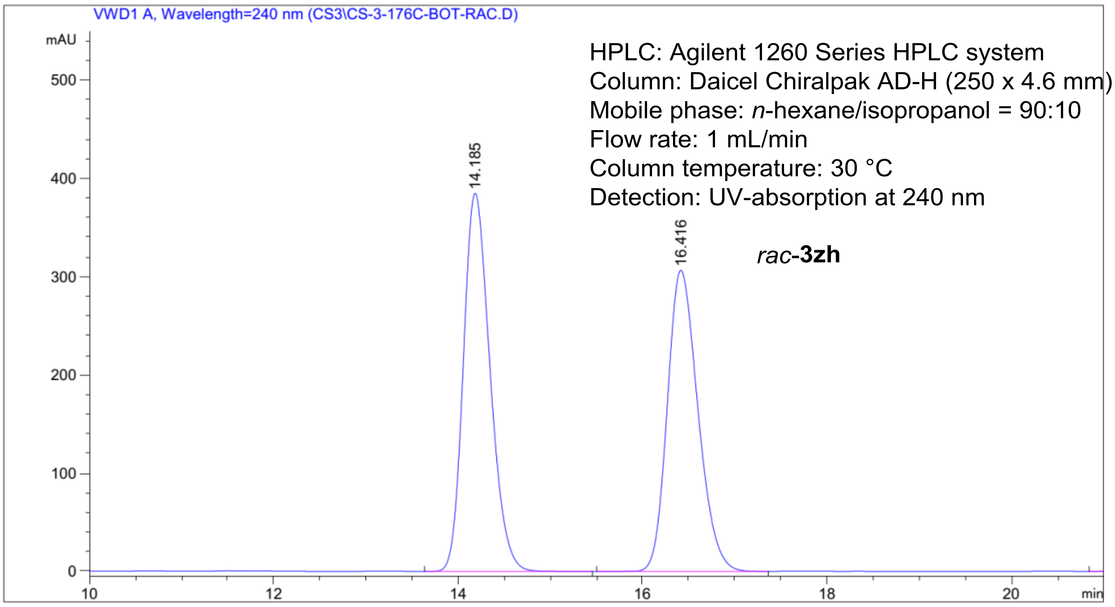

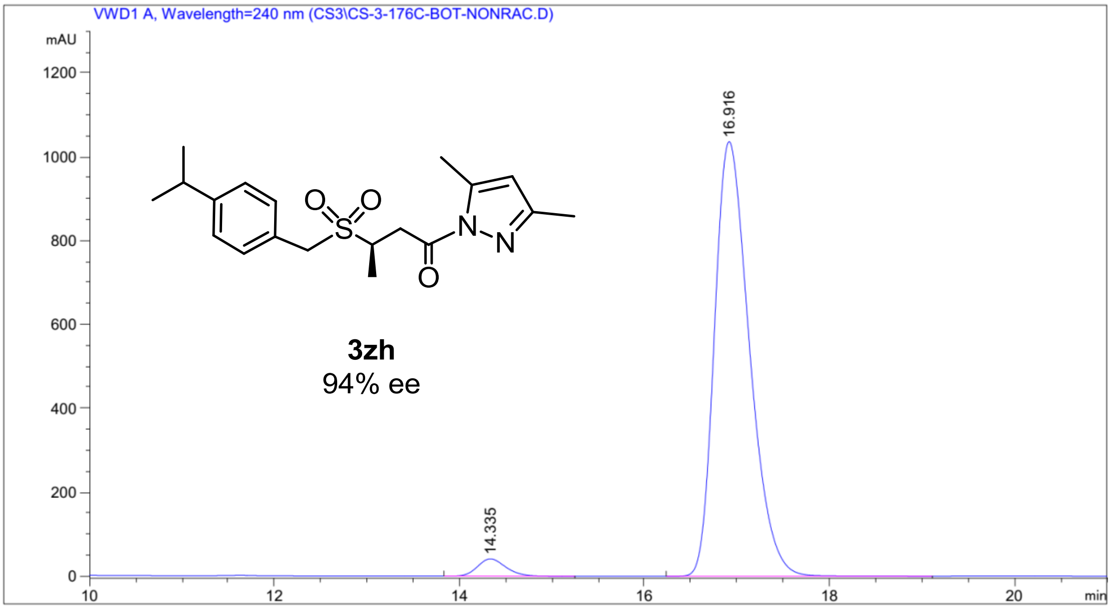

#	[min]		[min]	[mAU*s]	[mAU]	%
1	14.335	BB	0.3271	870.73352	41.11052	3.1108
2	16.916	BB	0.4054	2.71196e4	1036.09644	96.8892

Supplementary Fig. 46 HPLC trace for the racemic reference *rac*-**3zh**, and non-racemic product **3zh** generated from the photocatalytic asymmetric reaction.

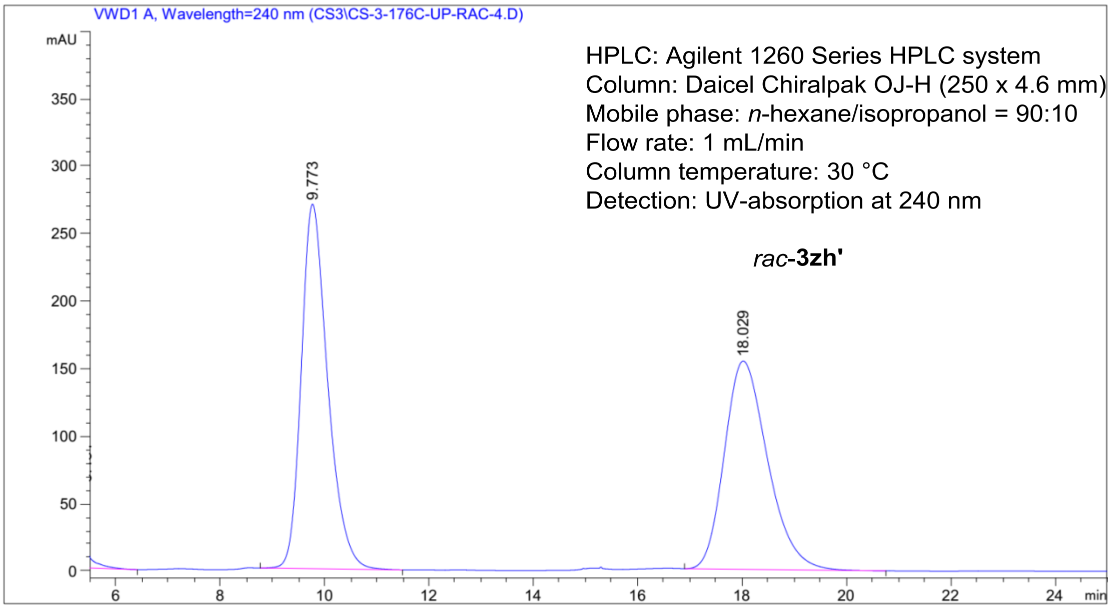

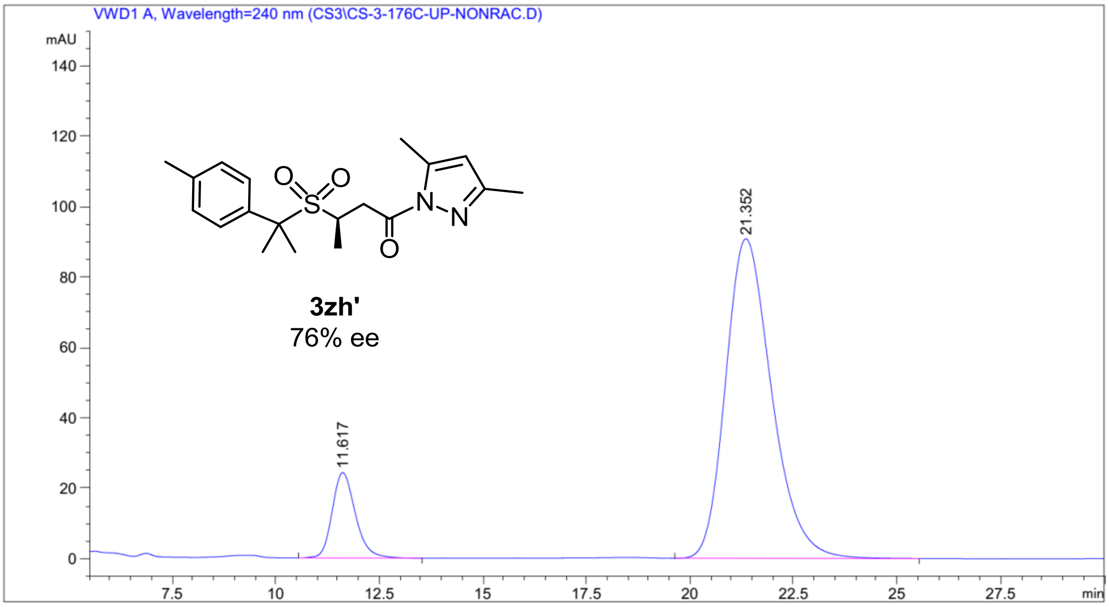

#	[min]		[min]	[mAU*s]	[mAU]	%
1	11.617	BB	0.5977	946.05316	24.27400	11.8849
2	21.352	BB	1.1870	7014.05615	90.81917	88.1151

Supplementary Fig. 47 HPLC trace for the racemic reference *rac*-**3zh'**, and non-racemic product **3zh'** generated from the photocatalytic asymmetric reaction.

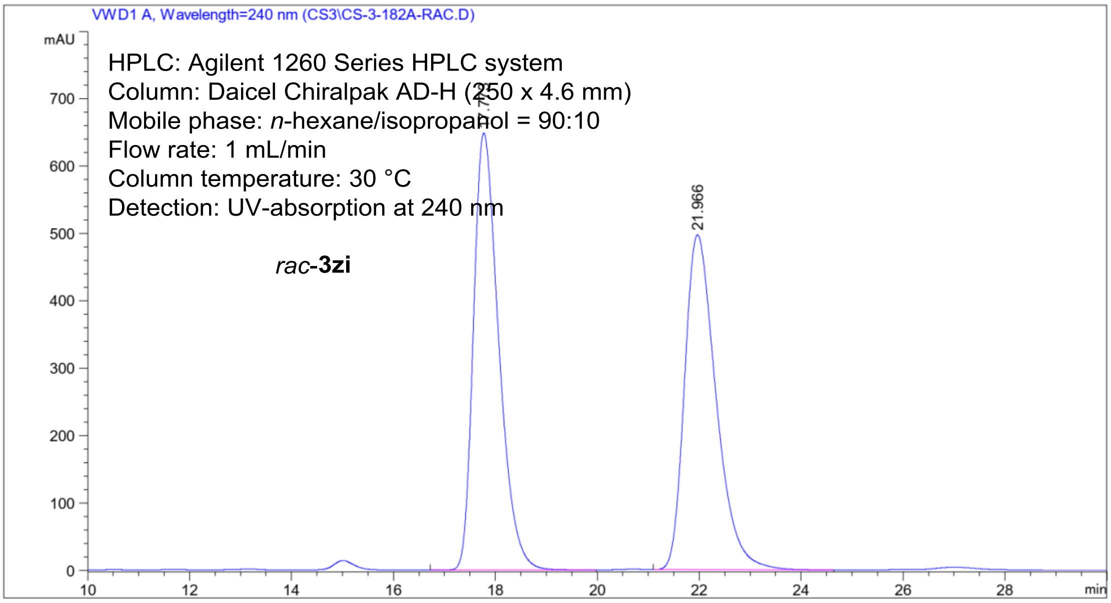

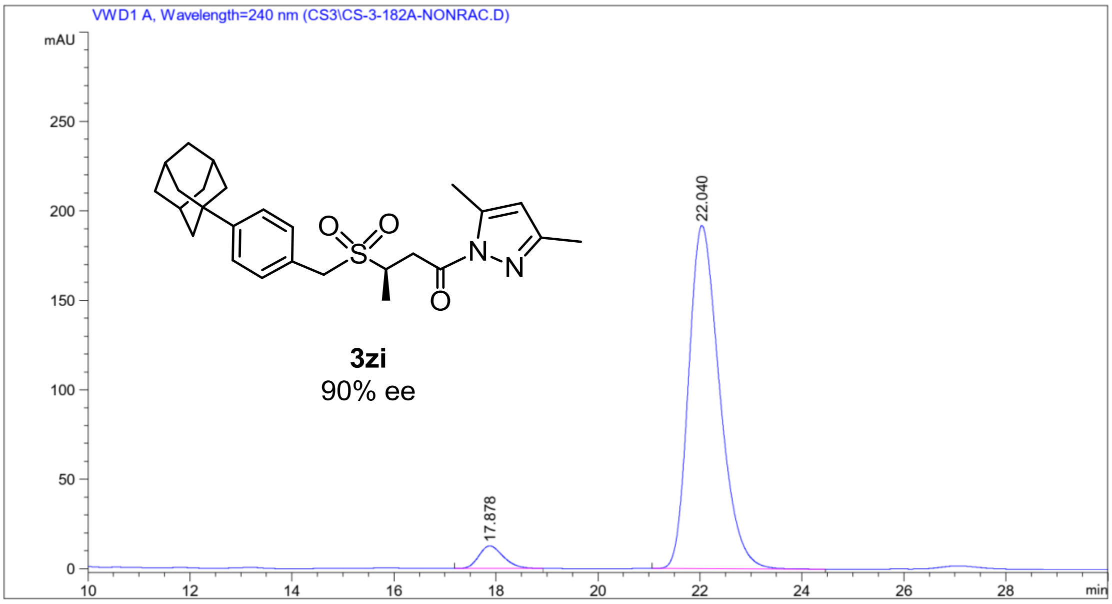

#	[min]		[min]	[mAU*s]	[mAU]	%
1	17.878	BB	0.5114	419.51840	12.60136	4.9402
2	22.040	BB	0.6512	8072.47314	191.74106	95.0598

Supplementary Fig. 48 HPLC trace for the racemic reference *rac*-**3zi**, and non-racemic product **3zi** generated from the photocatalytic asymmetric reaction.

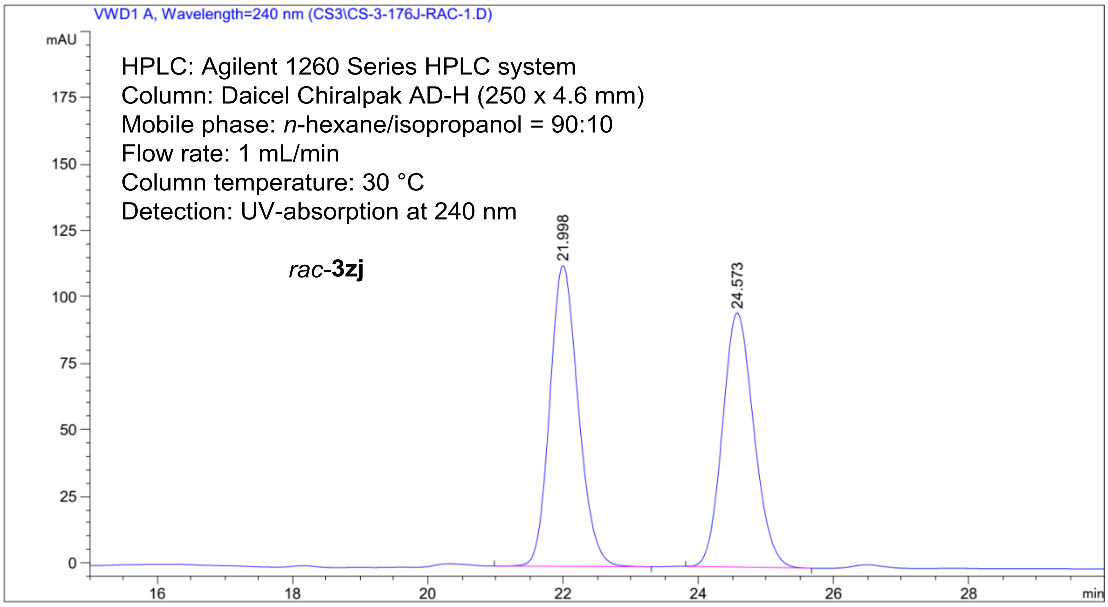

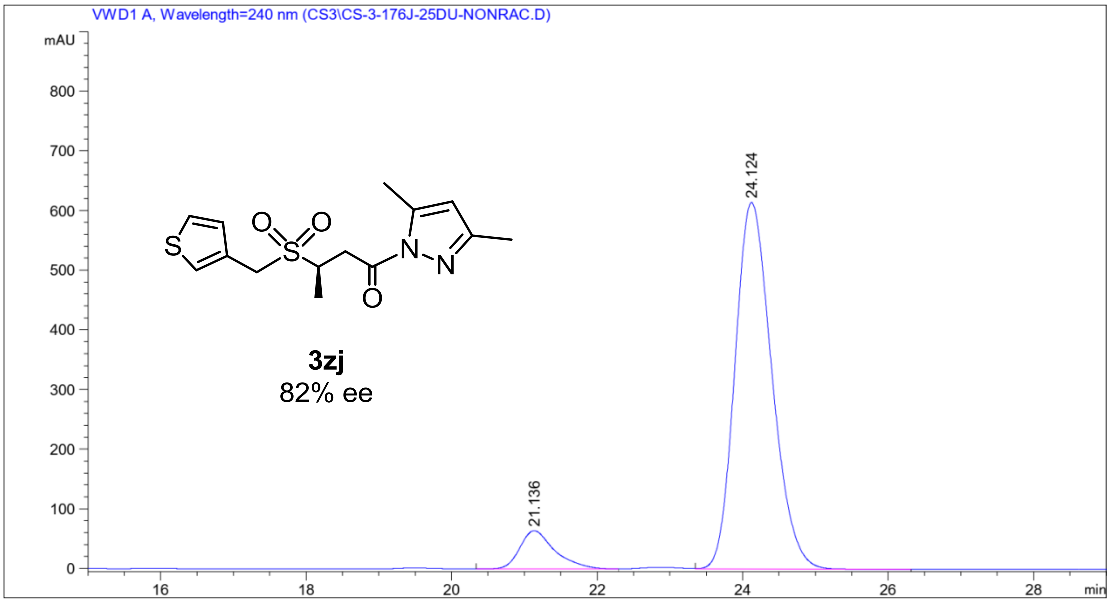

#	[min]		[min]	[mAU*s]	[mAU]	%
1	21.136	BB	0.4962	2139.10645	64.12009	8.9892
2	24.124	VB	0.5461	2.16573e4	614.65405	91.0108

Supplementary Fig. 49 HPLC trace for the racemic reference *rac*-**3zj**, and non-racemic product **3zj** generated from the photocatalytic asymmetric reaction.

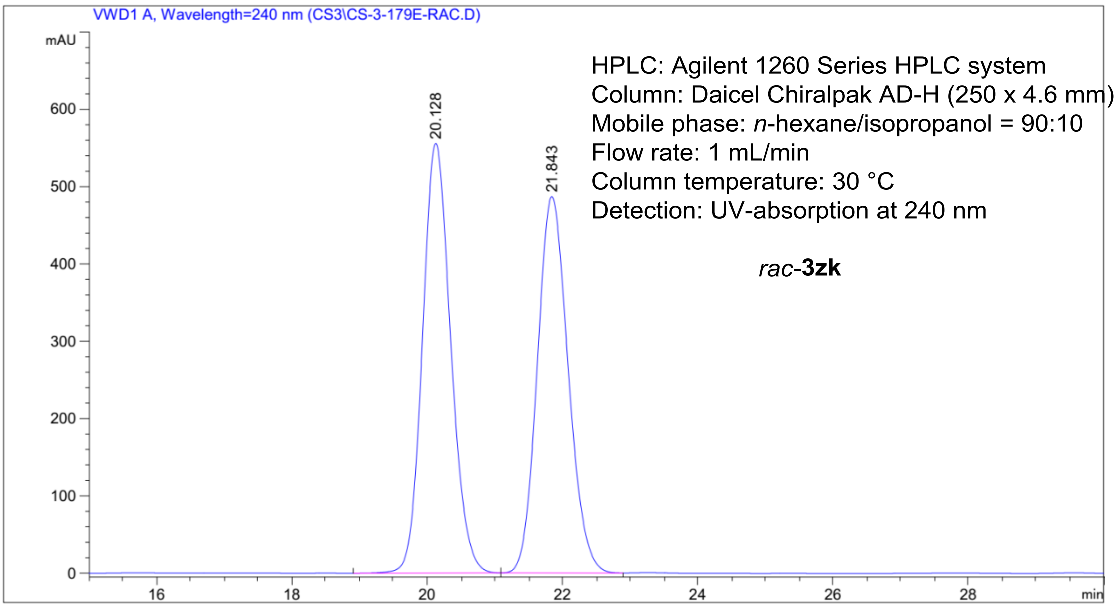

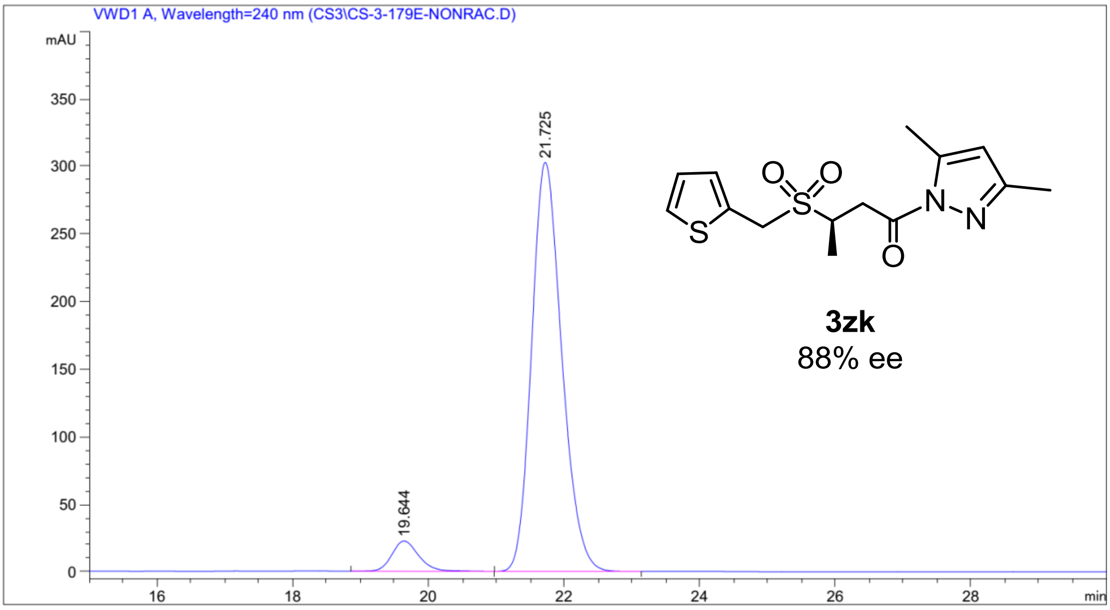

#	[min]		[min]	[mAU*s]	[mAU]	%
1	19.644	BB	0.4204	616.15204	22.36259	6.1943
2	21.725	BB	0.4749	9330.99609	302.51266	93.8057

Supplementary Fig. 50 HPLC trace for the racemic reference *rac*-**3zk**, and non-racemic product **3zk** generated from the photocatalytic asymmetric reaction.

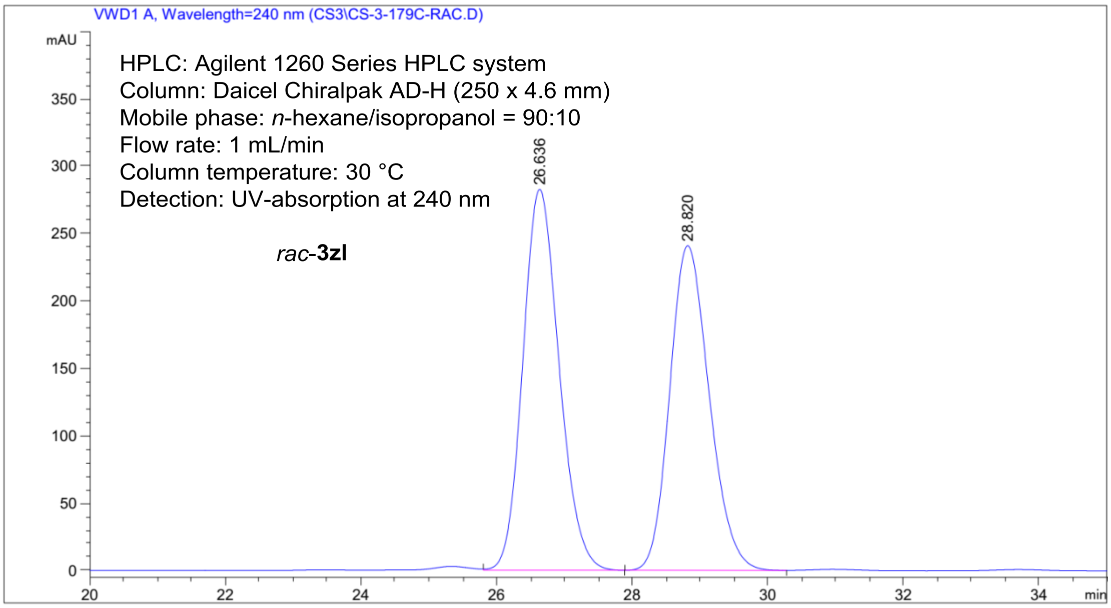

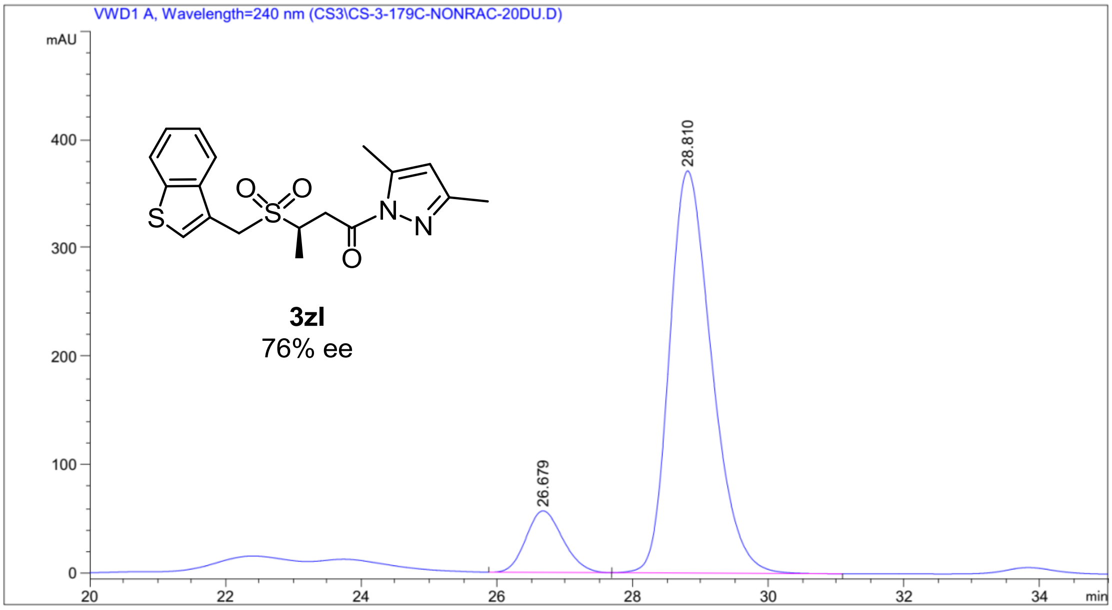

#	[min]		[min]	[mAU*s]	[mAU]	%
1	26.679	BB	0.5832	2142.97339	56.79232	11.7891
2	28.810	BB	0.6677	1.60347e4	370.68622	88.2109

Supplementary Fig. 51 HPLC trace for the racemic reference *rac*-**3zl**, and non-racemic product **3zl** generated from the photocatalytic asymmetric reaction.

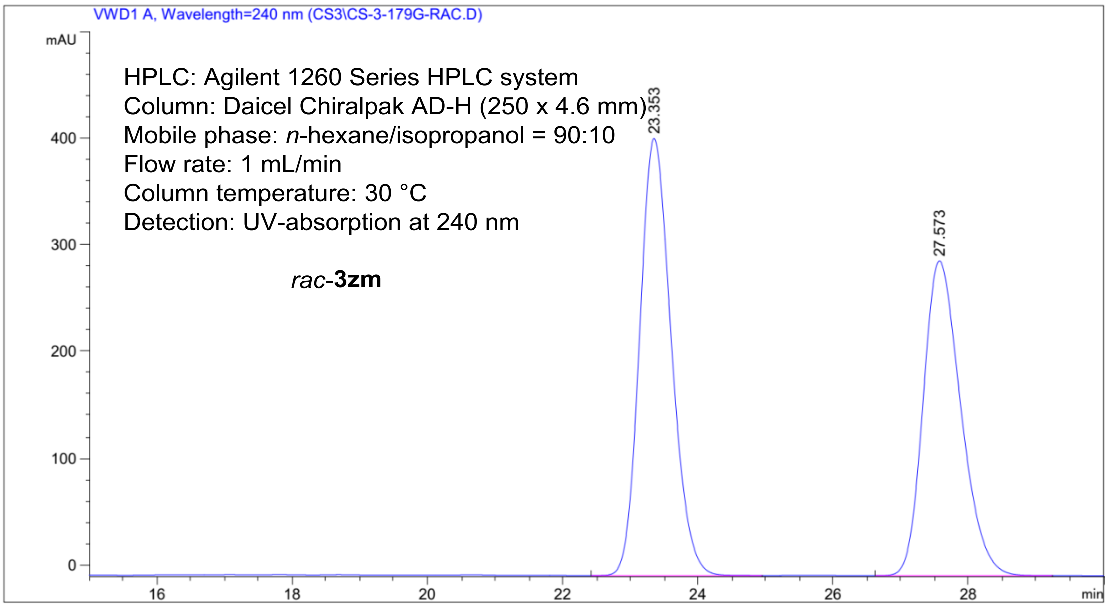

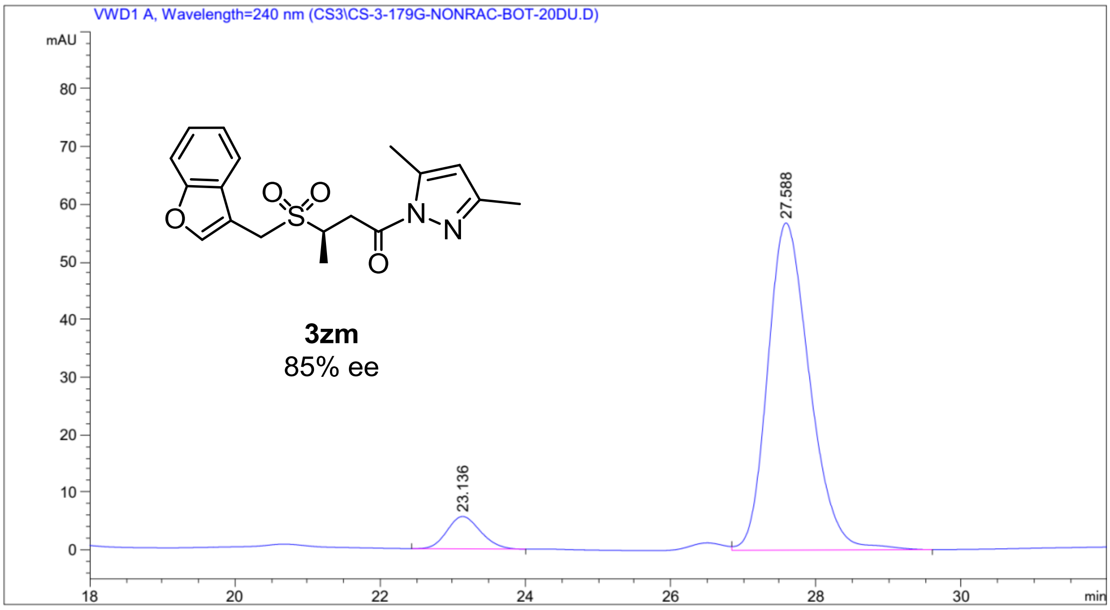

#	[min]		[min]	[mAU*s]	[mAU]	%
1	23.136	VB	0.4975	179.80463	5.61627	7.3137
2	27.588	VB	0.6206	2278.65186	56.73196	92.6863

Supplementary Fig. 52 HPLC trace for the racemic reference *rac*-**3zm**, and non-racemic product **3zm** generated from the photocatalytic asymmetric reaction.

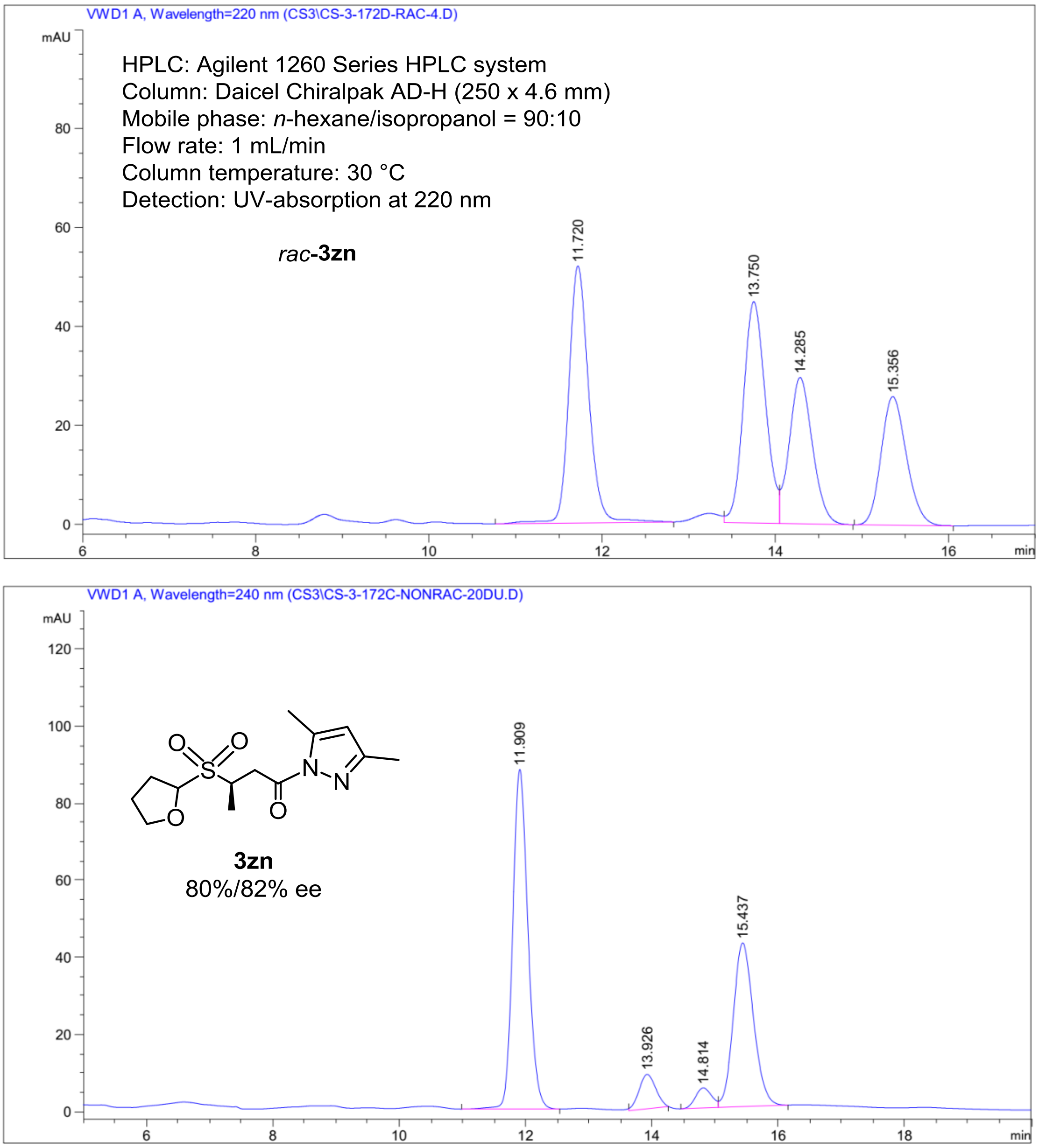

#	[min]		[min]	[mAU*s]	[mAU]	%
1	11.909	BB	0.2564	1468.26904	88.09670	54.9642
2	13.926	MM R	0.3063	163.48448	8.89659	6.1200
3	14.814	BV	0.2758	92.79695	5.20926	3.4738
4	15.437	VB	0.3454	946.76886	42.41631	35.4420

Supplementary Fig. 53 HPLC trace for the racemic reference *rac*-**3zn**, and non-racemic product **3zn** generated from the photocatalytic asymmetric reaction.

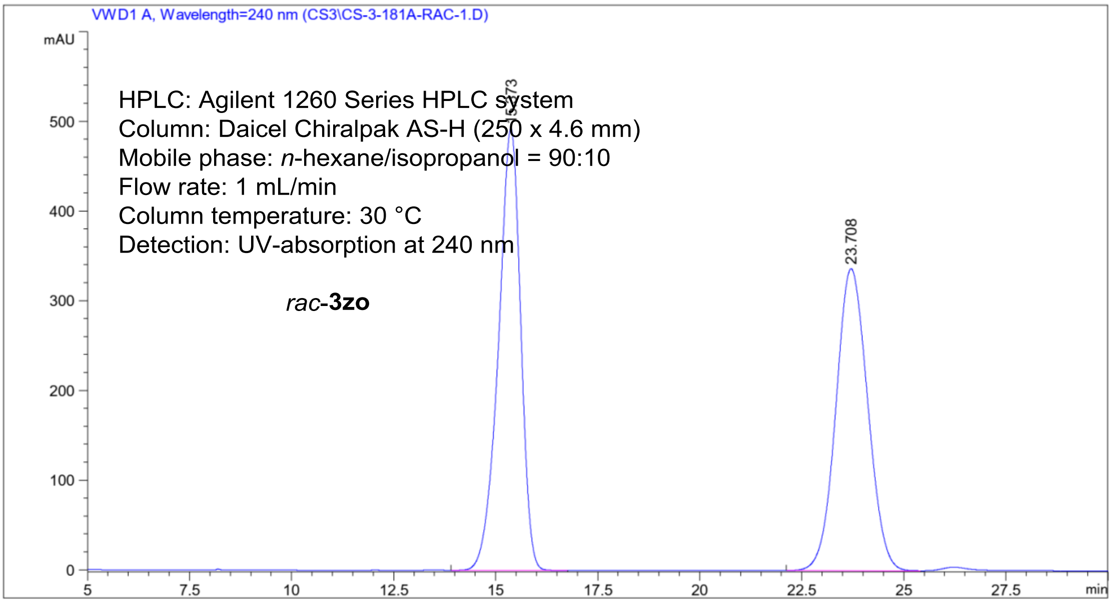

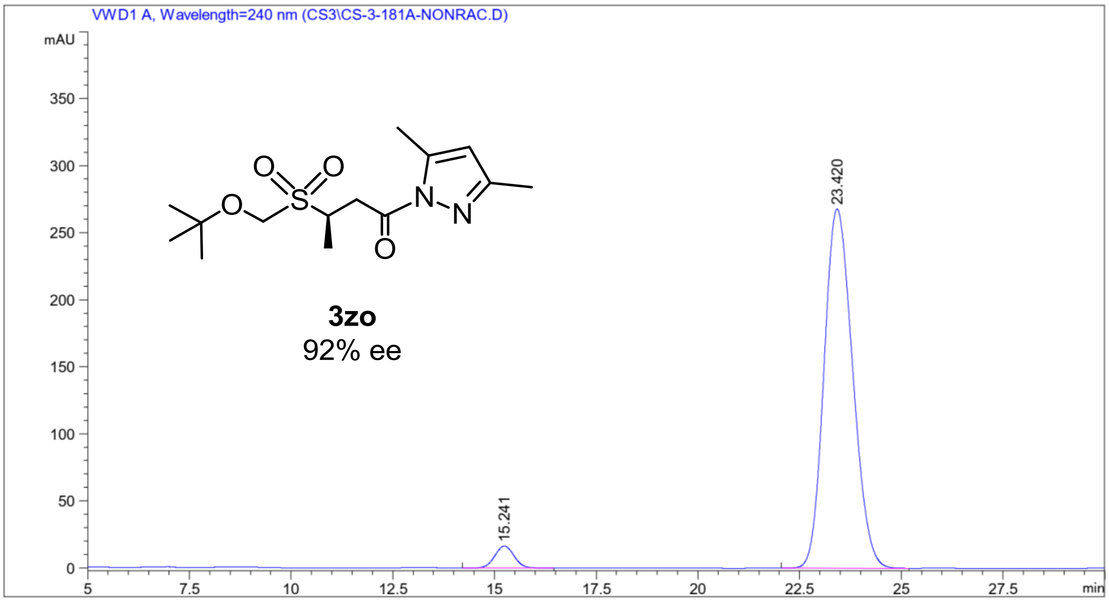

#	[min]		[min]	[mAU*s]	[mAU]	%
1	15.241	BB	0.5202	548.76935	16.44848	4.0305
2	23.420	BB	0.7681	1.30666e4	267.86639	95.9695

Supplementary Fig. 54 HPLC trace for the racemic reference *rac*-**3zo**, and non-racemic product **3zo** generated from the photocatalytic asymmetric reaction.

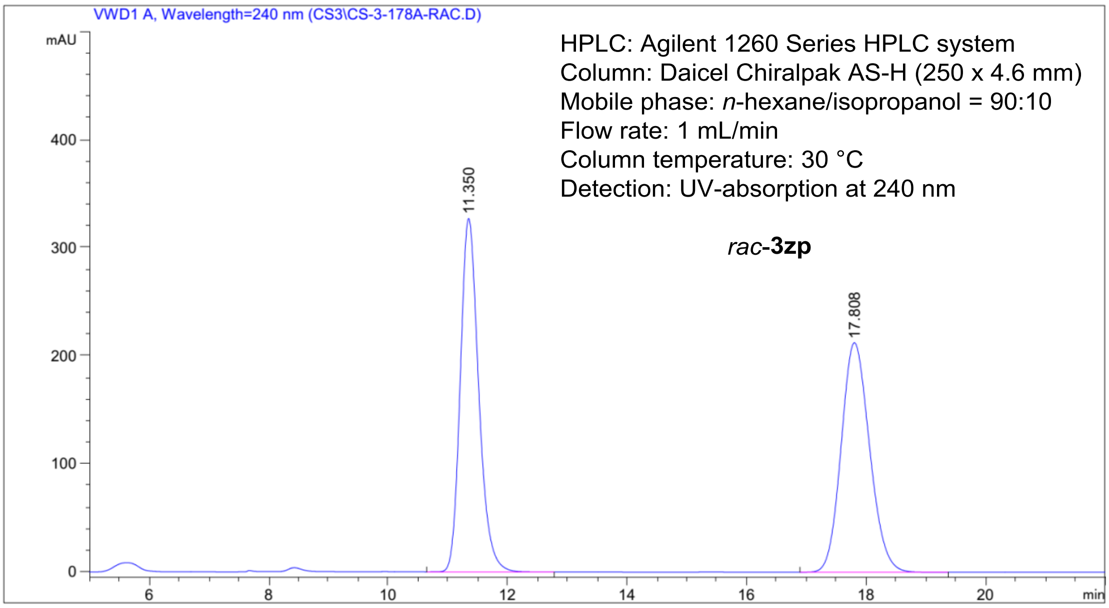

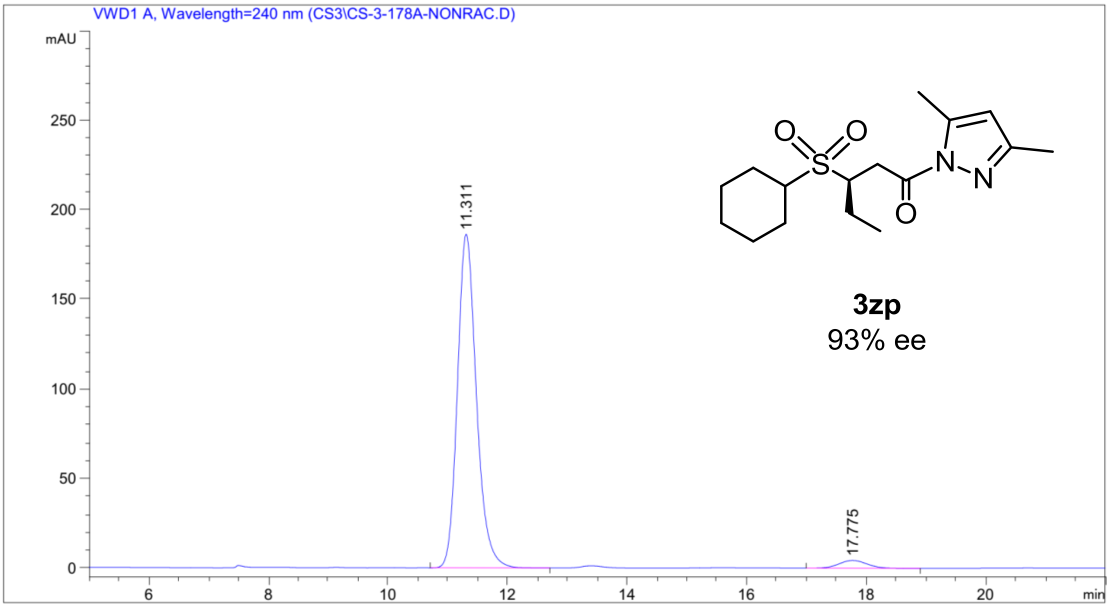

#	[min]		[min]	[mAU*s]	[mAU]	%
1	11.311	BB	0.3386	4113.02832	186.24892	96.5877
2	17.775	BB	0.5027	145.30856	4.41825	3.4123

Supplementary Fig. 55 HPLC trace for the racemic reference *rac*-**3zp**, and non-racemic product **3zp** generated from the photocatalytic asymmetric reaction.

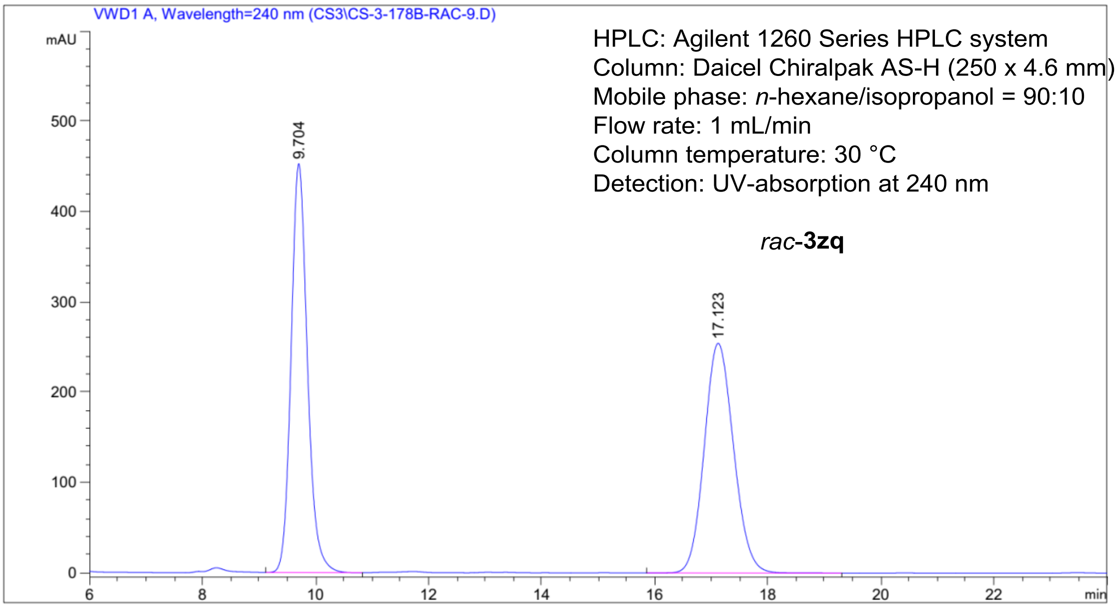

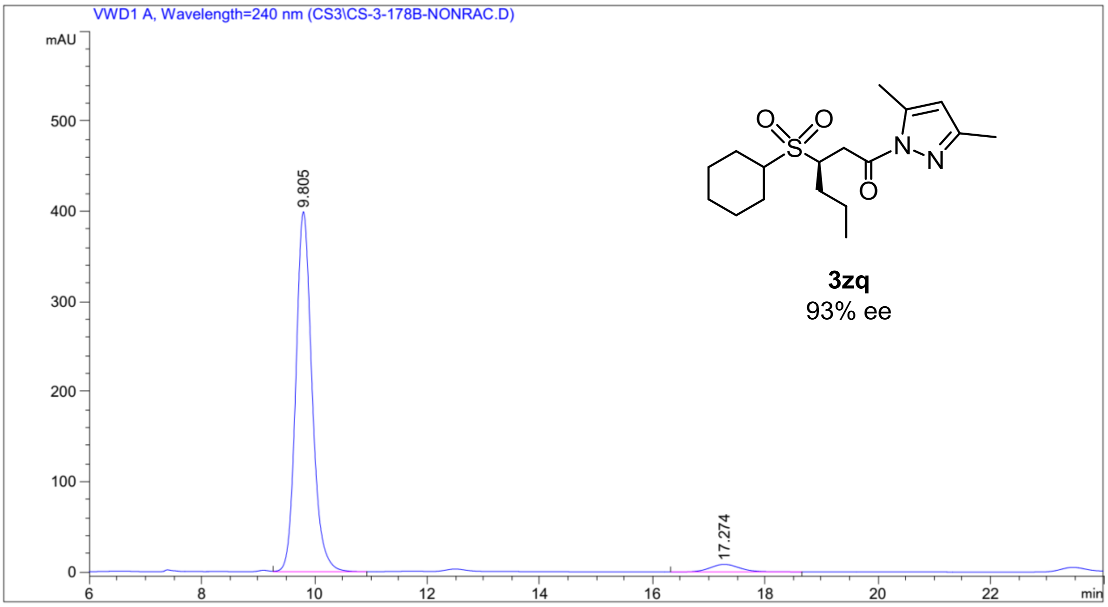

#	[min]		[min]	[mAU*s]	[mAU]	%
1	9.805	VB	0.3060	7978.52393	399.42349	96.3545
2	17.274	BB	0.5563	301.86172	8.30064	3.6455

Supplementary Fig. 56 HPLC trace for the racemic reference *rac*-**3zq**, and non-racemic product **3zq** generated from the photocatalytic asymmetric reaction.

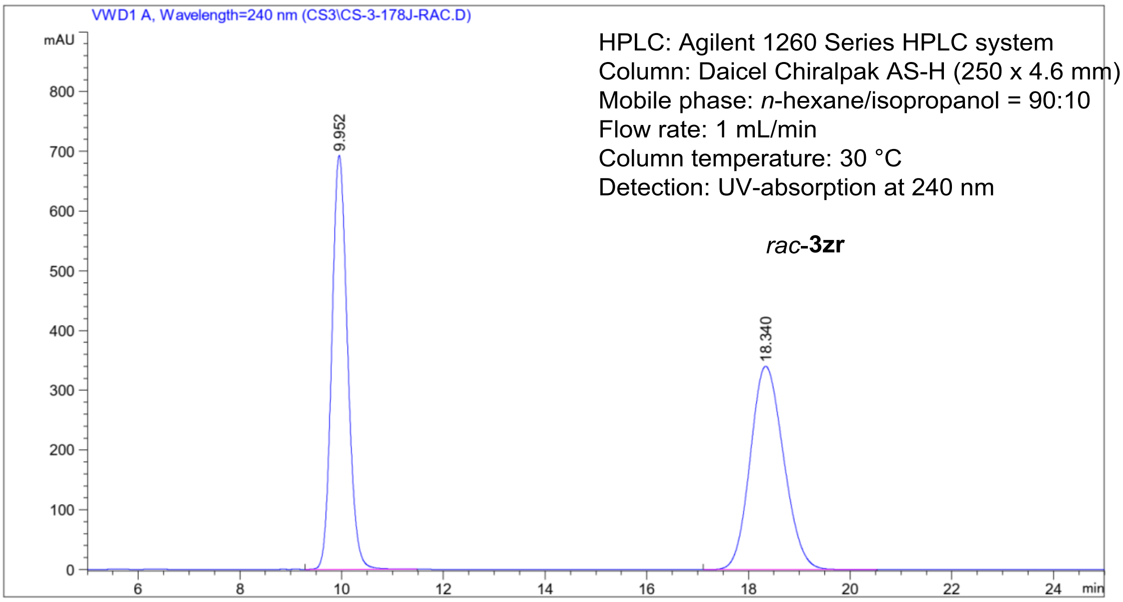

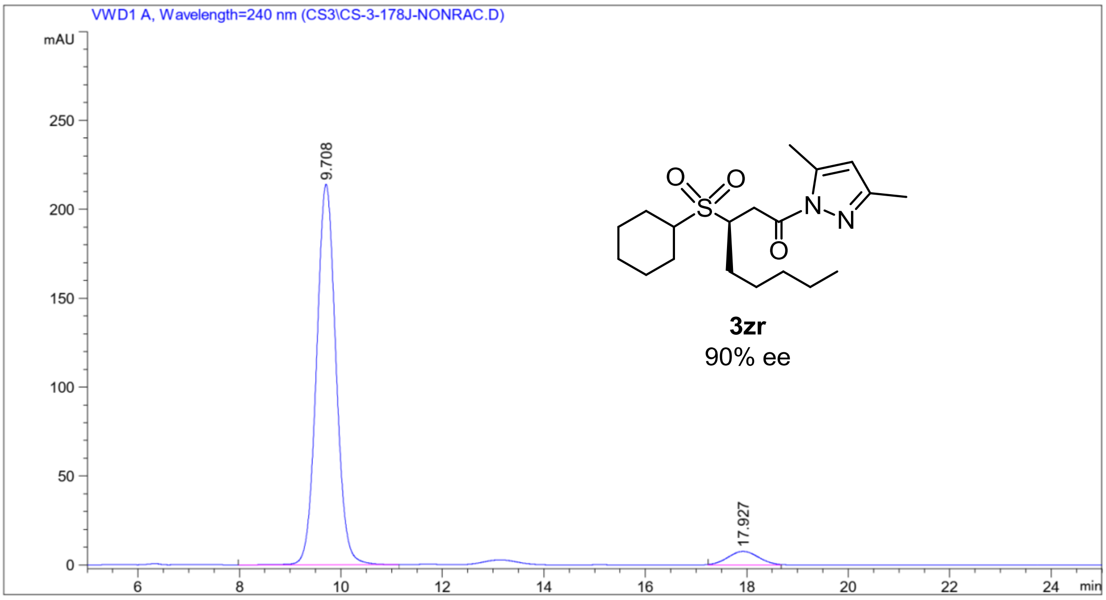

#	[min]		[min]	[mAU*s]	[mAU]	%
1	9.708	BB	0.4114	5673.95508	213.93188	94.7615
2	17.927	MM R	0.6901	313.66321	7.57573	5.2385

Supplementary Fig. 57 HPLC trace for the racemic reference *rac*-**3zr**, and non-racemic product **3zr** generated from the photocatalytic asymmetric reaction.

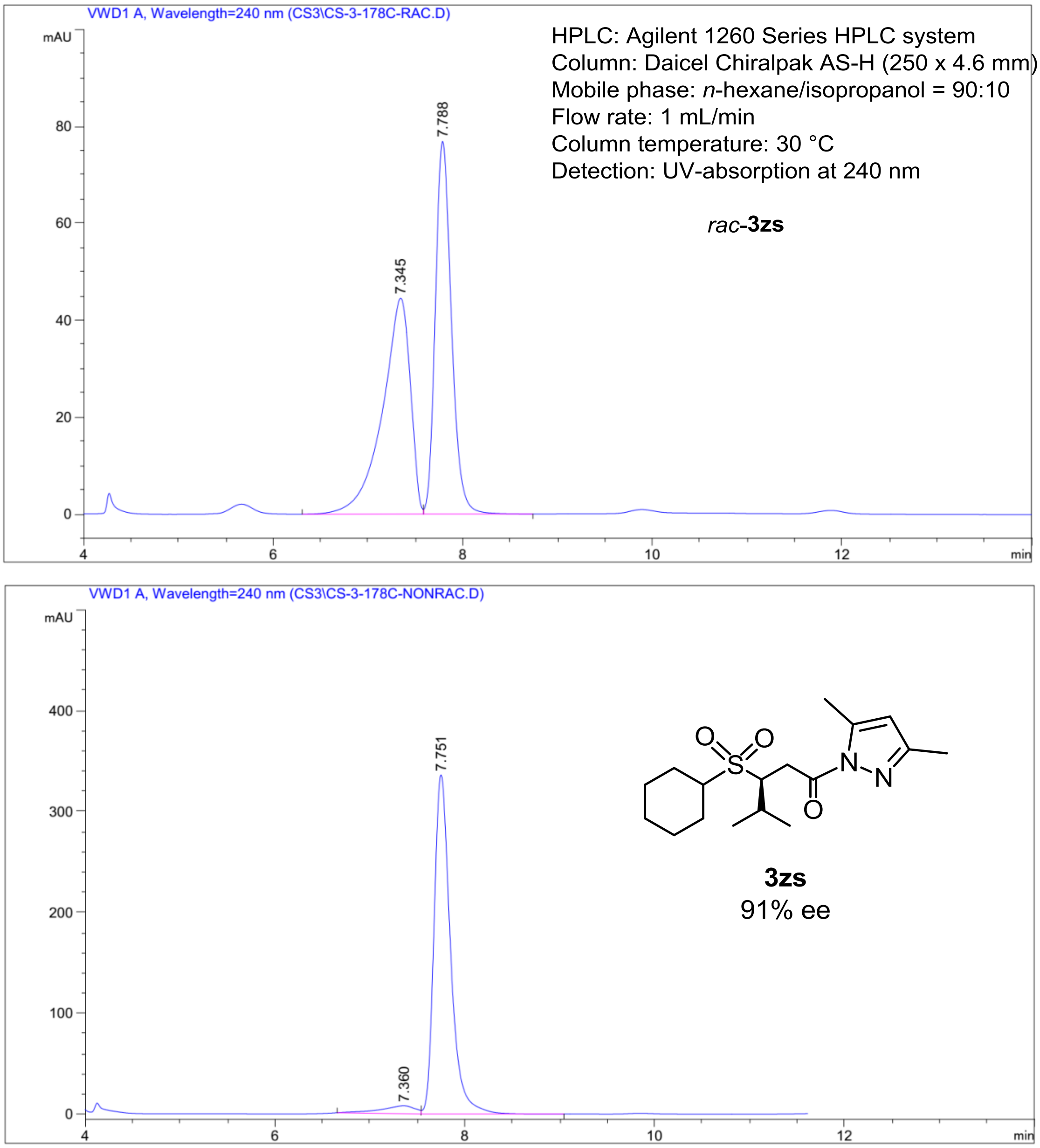

#	[min]		[min]	[mAU*s]	[mAU]	%
1	7.360	MM R	0.4203	200.39612	7.94694	4.7125
2	7.751	VB	0.1838	4052.04565	335.86633	95.2875

Supplementary Fig. 58 HPLC trace for the racemic reference *rac*-**3zs**, and non-racemic product **3zs** generated from the photocatalytic asymmetric reaction.

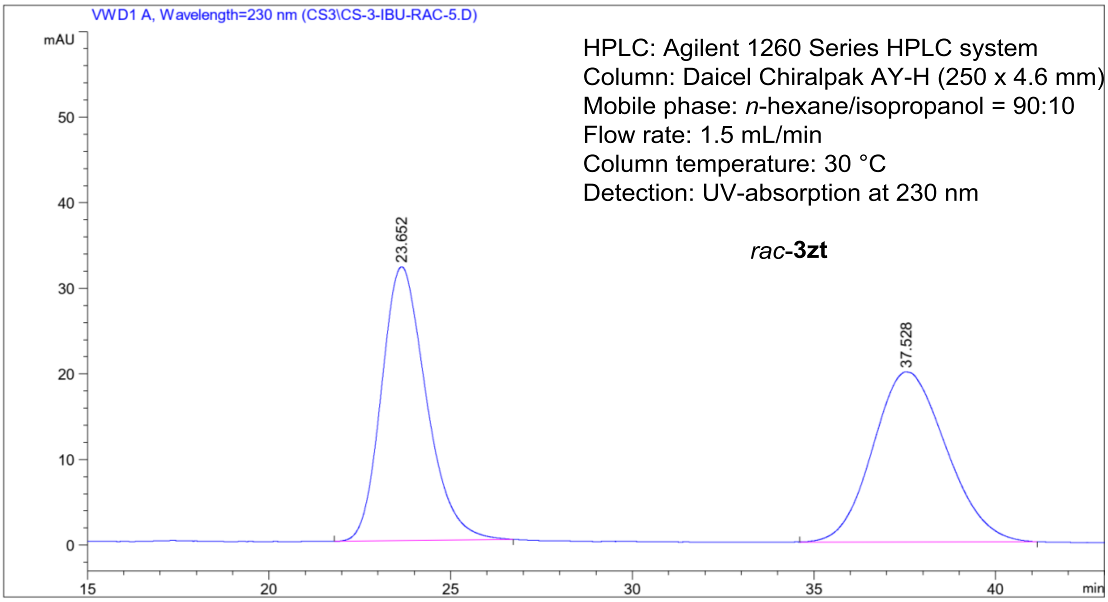

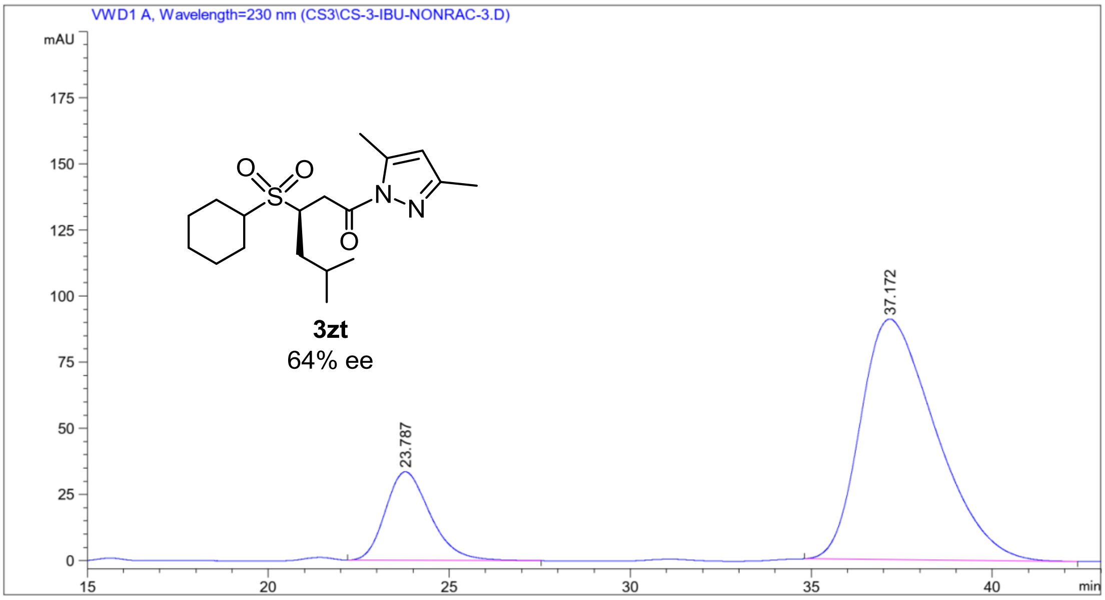

#	[min]		[min]	[mAU*s]	[mAU]	%
1	23.787	BB	1.2732	2869.37500	33.43701	17.8770
2	37.172	BB	2.0004	1.31813e4	90.91800	82.1230

Supplementary Fig. 59 HPLC trace for the racemic reference *rac*-**3zt**, and non-racemic product **3zt** generated from the photocatalytic asymmetric reaction.

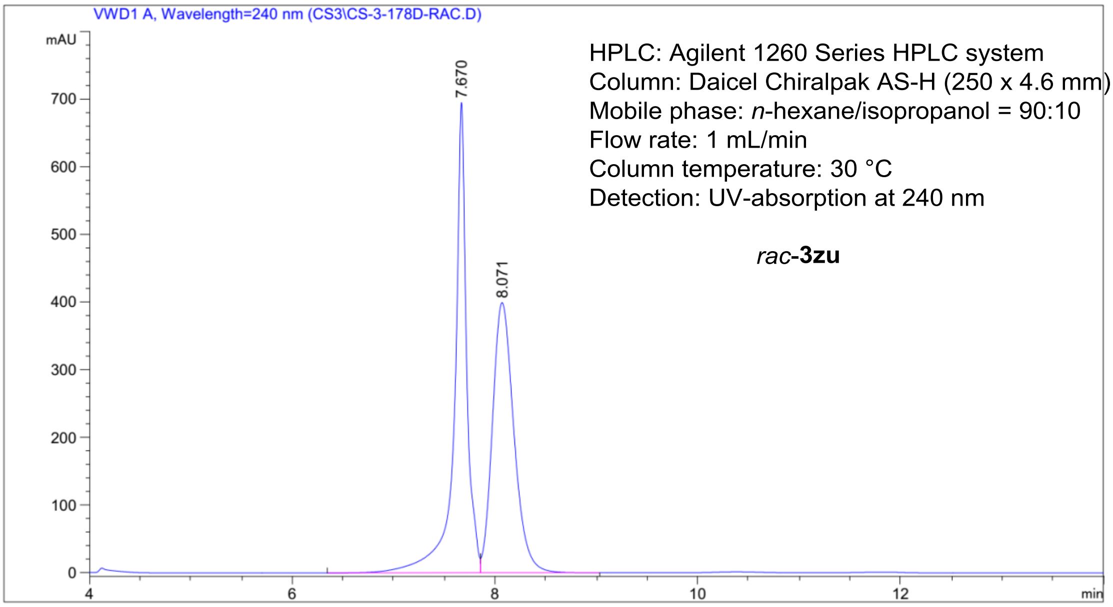

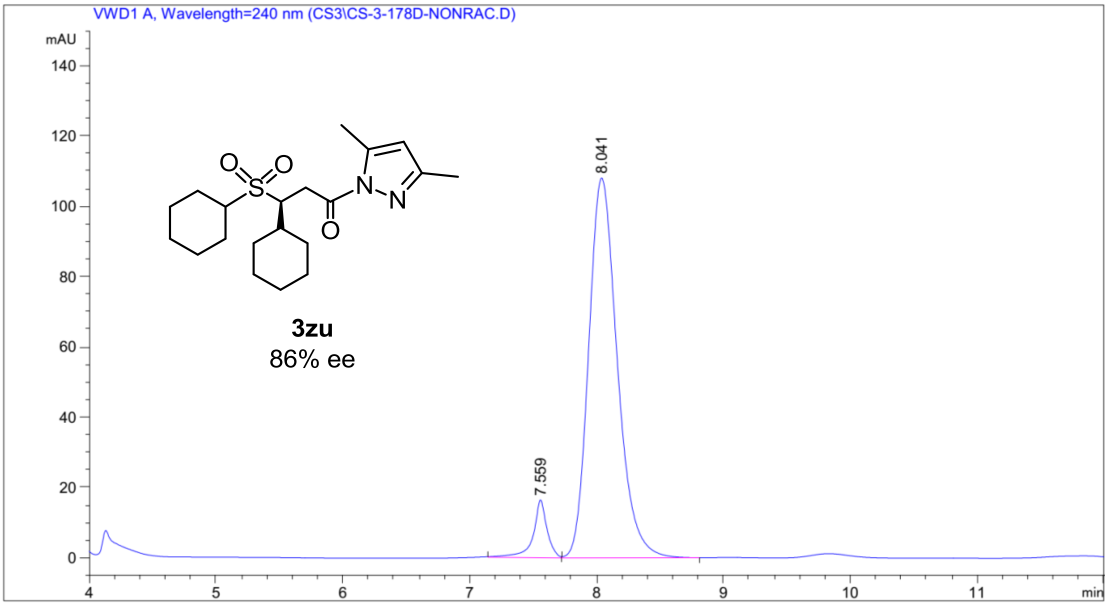

#	[min]		[min]	[mAU*s]	[mAU]	%
1	7.559	MM R	0.1277	125.97083	16.43508	6.9319
2	8.041	VB	0.2405	1691.30151	108.11472	93.0681

Supplementary Fig. 60 HPLC trace for the racemic reference *rac*-**3zu**, and non-racemic product **3zu** generated from the photocatalytic asymmetric reaction.

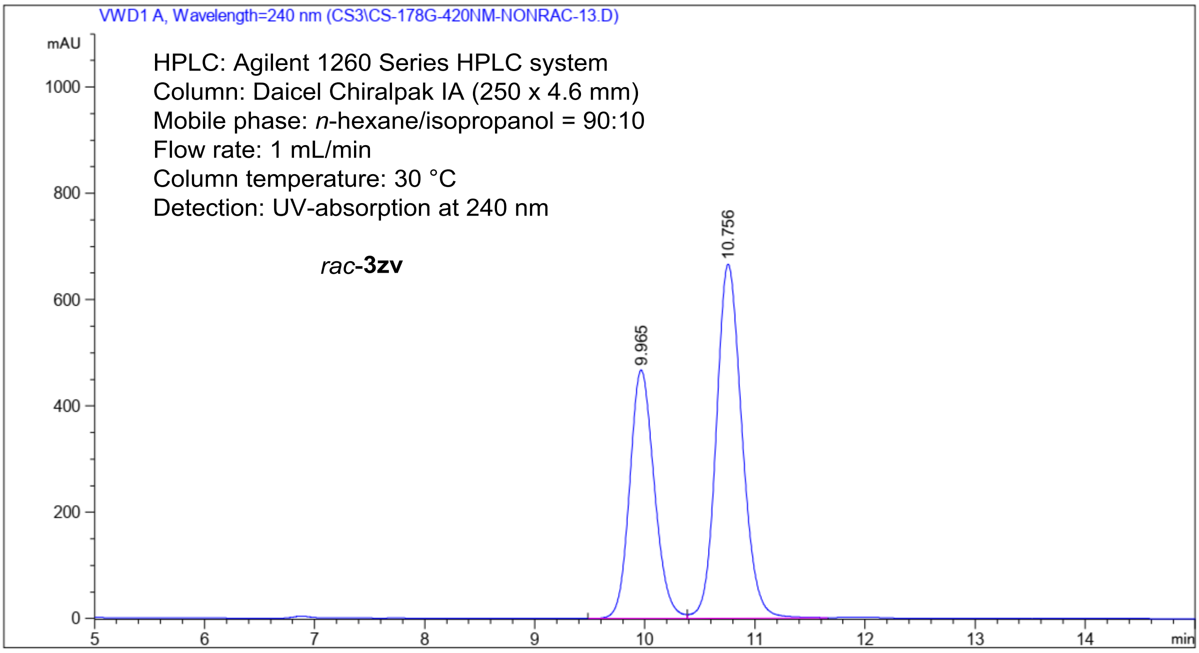

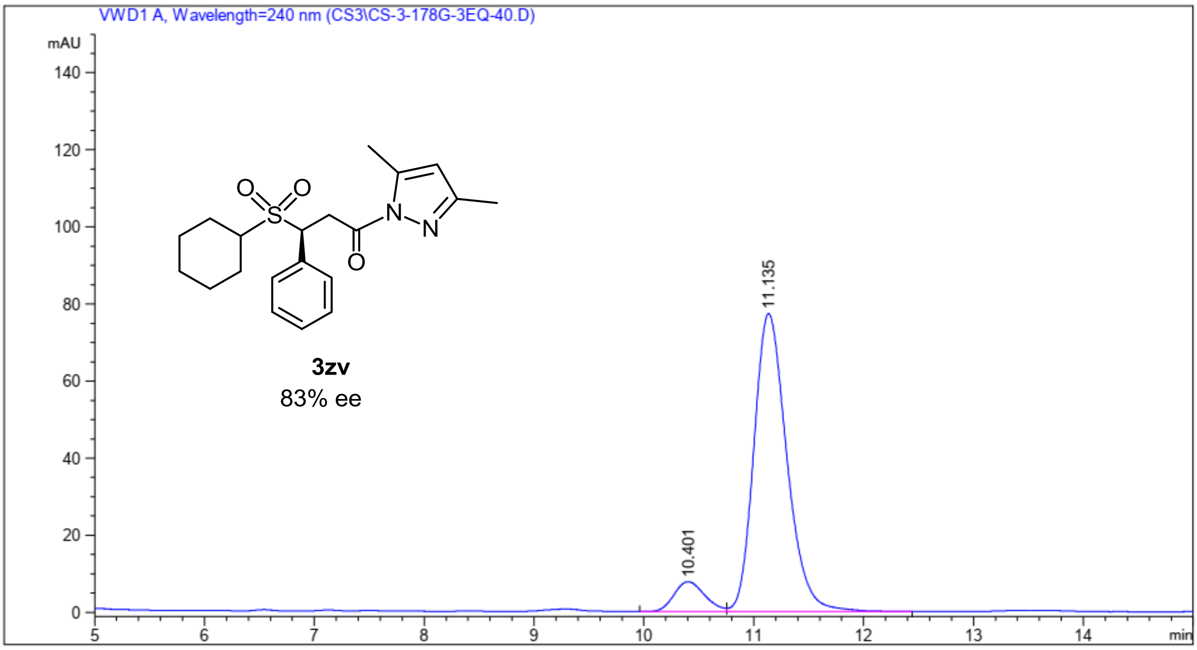

#	[min]		[min]	[mAU*s]	[mAU]	%
1	10.401	BV	0.3006	150.61443	7.75447	8.5644
2	11.135	VB	0.3207	1608.00366	77.29150	91.4356

Supplementary Fig. 61 HPLC trace for the racemic reference *rac*-**3zv**, and non-racemic product **3zv** generated from the photocatalytic asymmetric reaction.

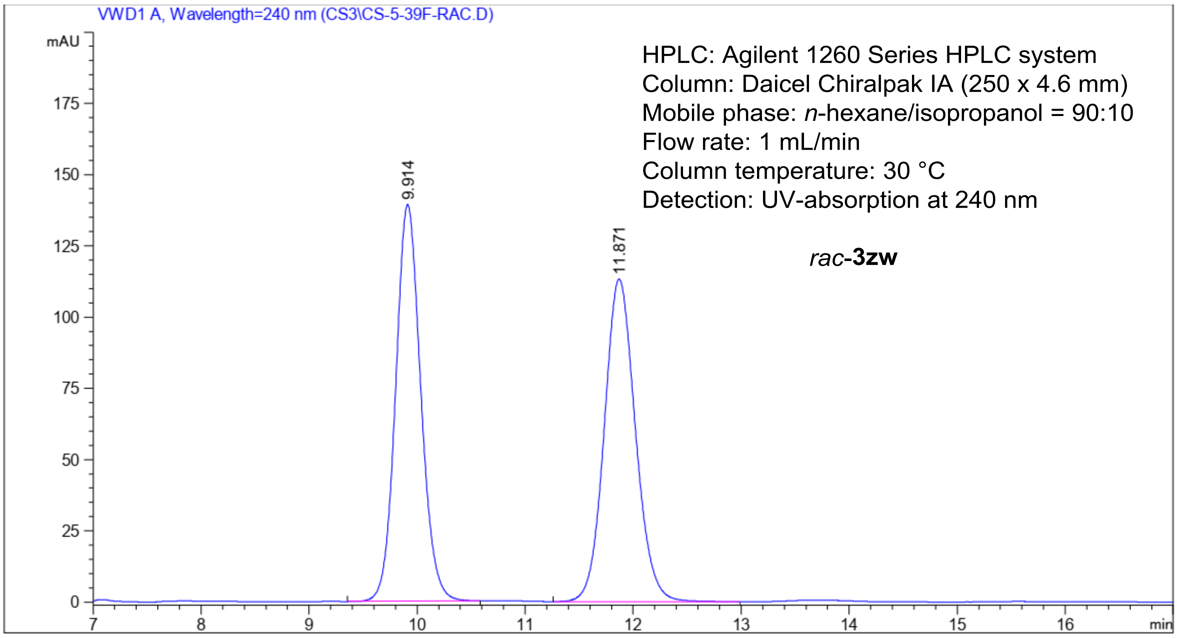

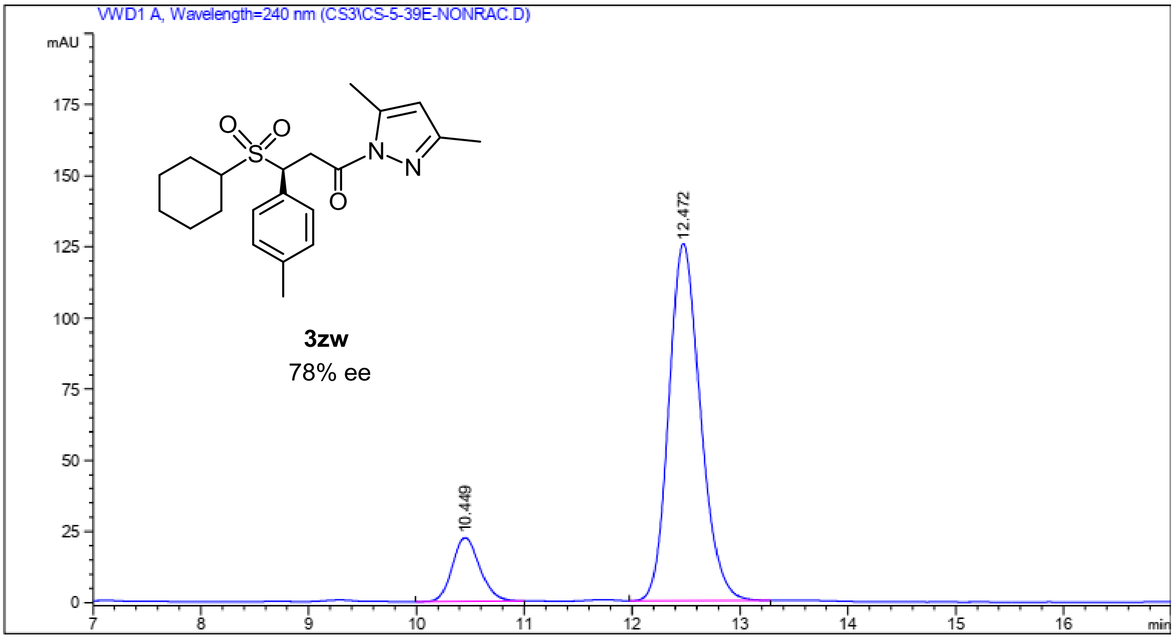

#	[min]		[min]	[mAU*s]	[mAU]	%
1	10.449	MM	0.2721	344.90289	21.12687	10.9695
2	12.472	MM	0.3681	2799.28809	126.73707	89.0305

Supplementary Fig. 62 HPLC trace for the racemic reference *rac*-**3zw**, and non-racemic product **3zw** generated from the photocatalytic asymmetric reaction.

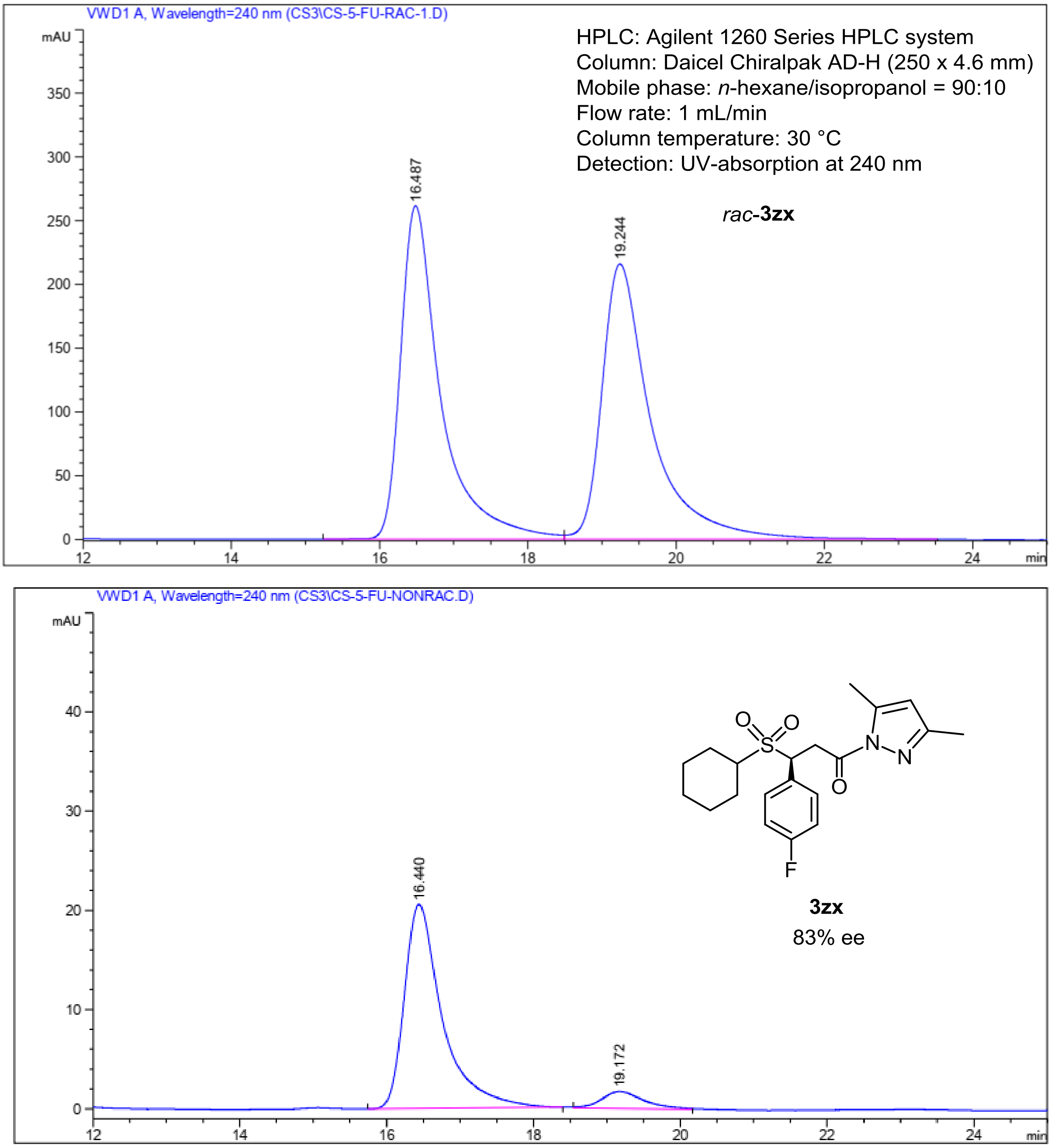

#	[min]		[min]	[mAU*s]	[mAU]	%
1	16.440	BB	0.5302	740.87164	20.56790	91.5328
2	19.172	MM R	0.6674	68.53375	1.71137	8.4672

Supplementary Fig. 63 HPLC trace for the racemic reference *rac*-**3zx**, and non-racemic product **3zx** generated from the photocatalytic asymmetric reaction.

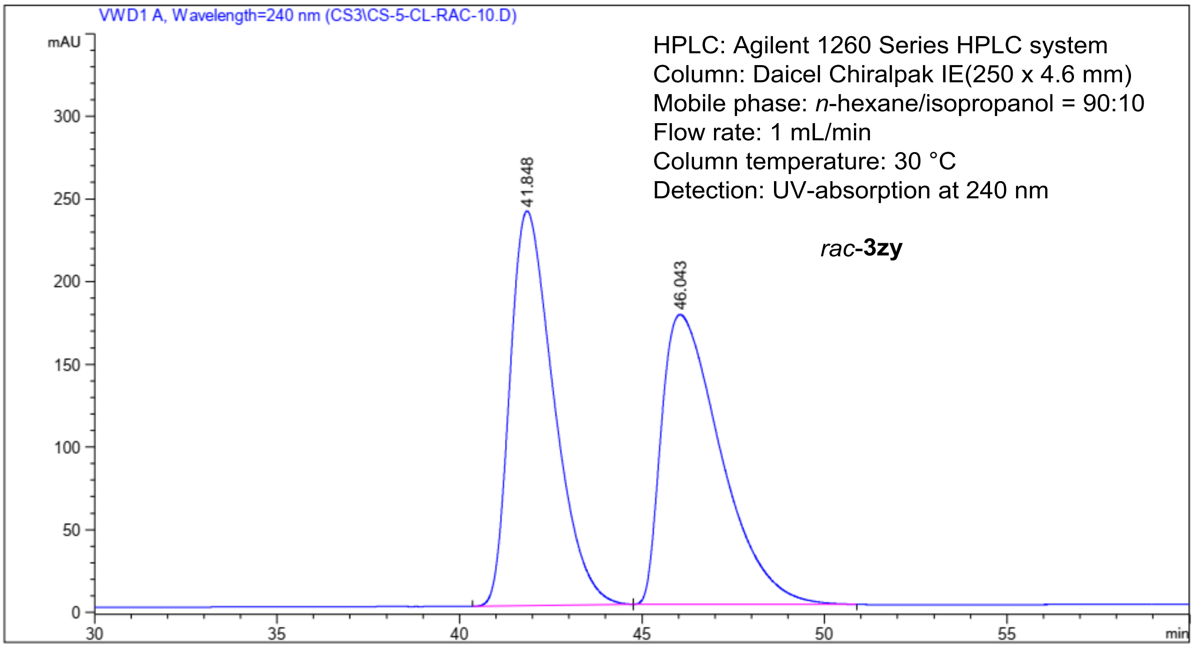

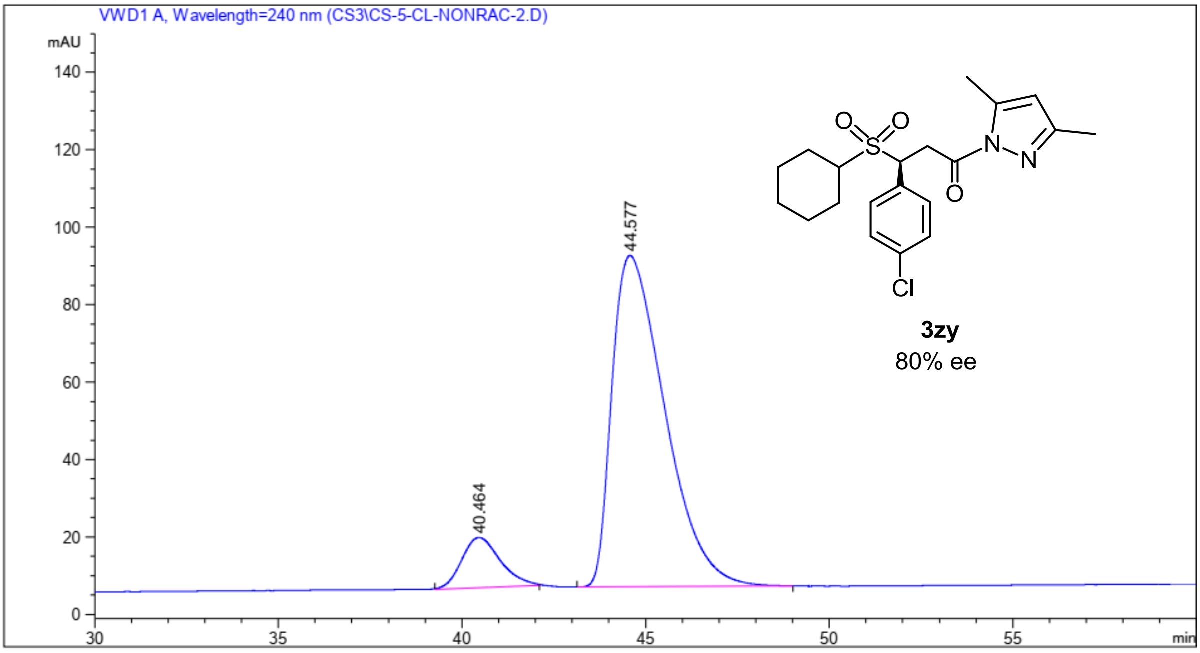

#	[min]		[min]	[mAU*s]	[mAU]	%
1	40.464	MM R	1.2175	946.58685	12.95835	9.9422
2	44.577	BB	1.5241	8574.26855	85.56657	90.0578

Supplementary Fig. 64 HPLC trace for the racemic reference *rac*-**3zy**, and non-racemic product **3zy** generated from the photocatalytic asymmetric reaction.

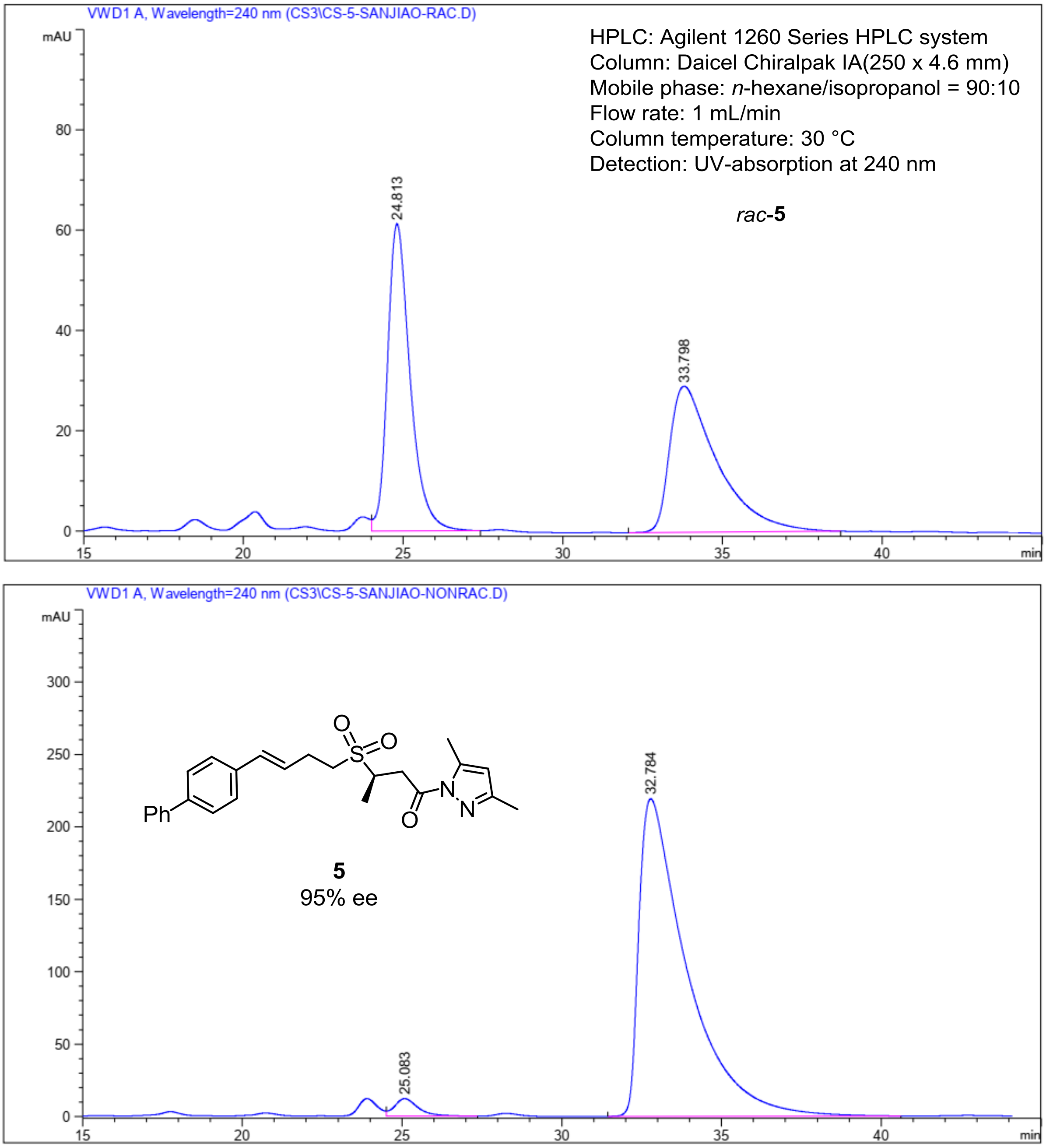

#	[min]		[min]	[mAU*s]	[mAU]	%
1	25.083	VB	0.7682	629.14948	12.06918	2.6778
2	32.784	BB	1.4645	2.28658e4	219.11383	97.3222

Supplementary Fig. 65 HPLC trace for the racemic reference *rac*-**5**, and non-racemic product **5** generated from the photocatalytic asymmetric reaction.

6.2 Determination of Enantiopurities of the Transformation Products

Optical purities of the compounds **7**−**12** were determined with a Daicel Chiralpak AD-H, AY-H or AZ-H column on an Agilent 1260 Series HPLC System. The column temperature was 30 ℃ and UV-absorption was measured at 220 nm.

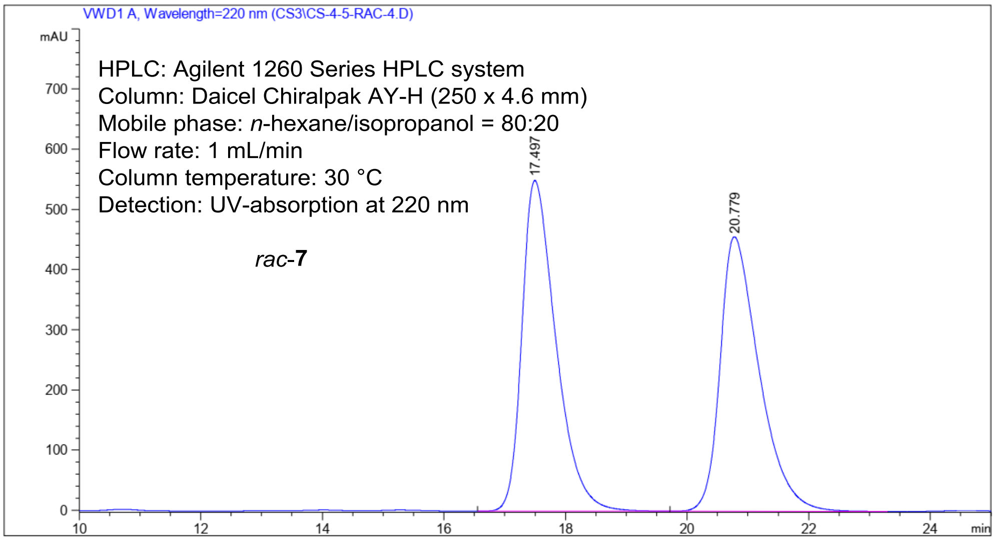

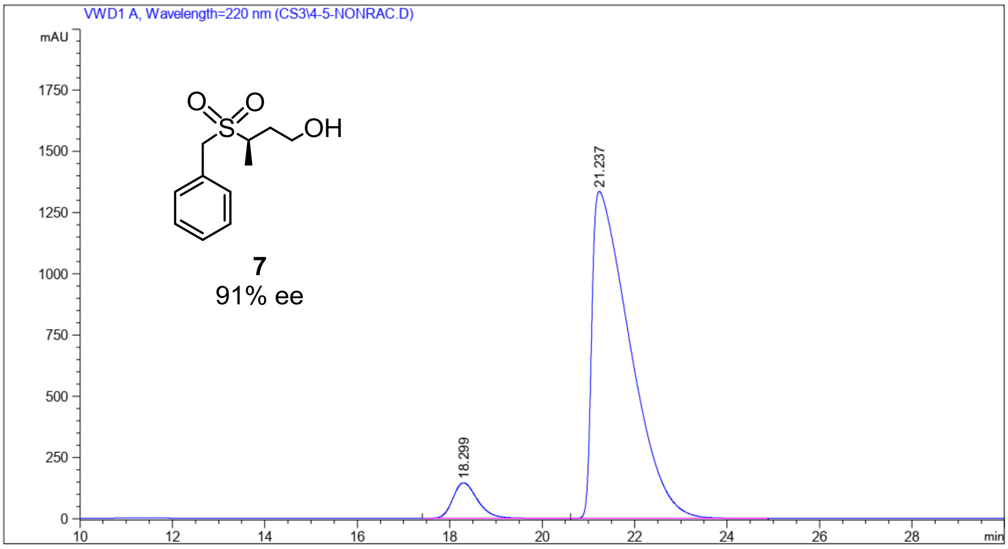

#	[min]		[min]	[mAU*s]	[mAU]	%
1	18.299	MM R	0.5575	4537.04590	135.62859	4.6995
2	21.237	MM R	1.1077	9.20052e4	1384.32349	95.3005

Supplementary Fig. 66 HPLC trace for the racemic reference *rac*-**7**, and non-racemic product **7** generated from the transformation of **3v**.

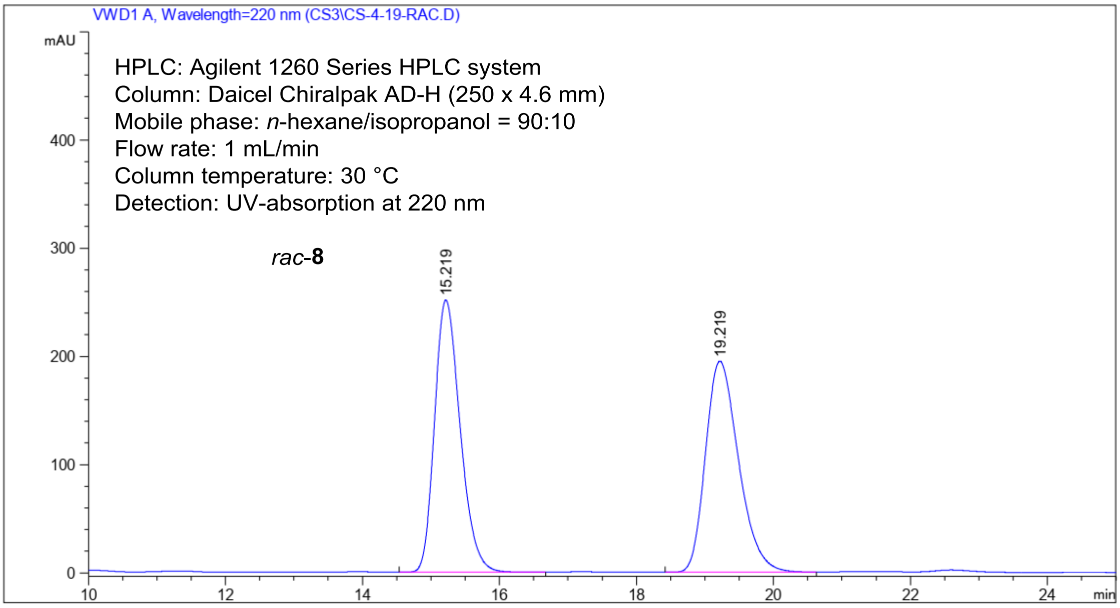

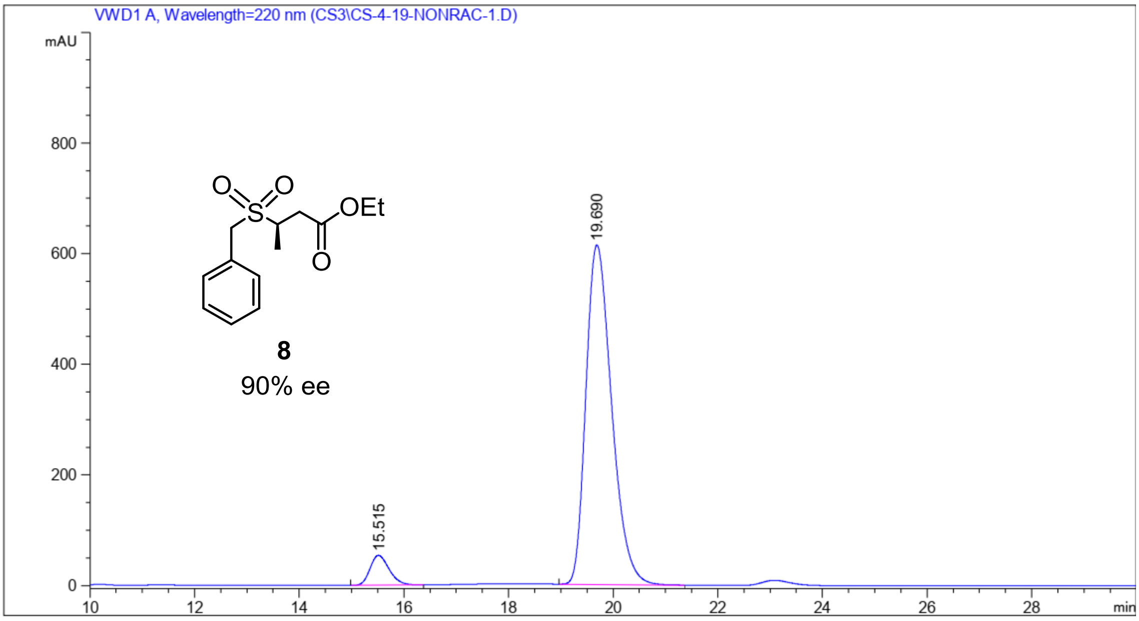

#	[min]		[min]	[mAU*s]	[mAU]	%
1	15.515	MM R	0.3960	1223.28503	51.49138	5.1357
2	19.690	MM R	0.6084	2.25957e4	619.03986	94.8643

Supplementary Fig. 67 HPLC trace for the racemic reference *rac*-**8** and non-racemic product **8** generated from the transformation of **3v**.

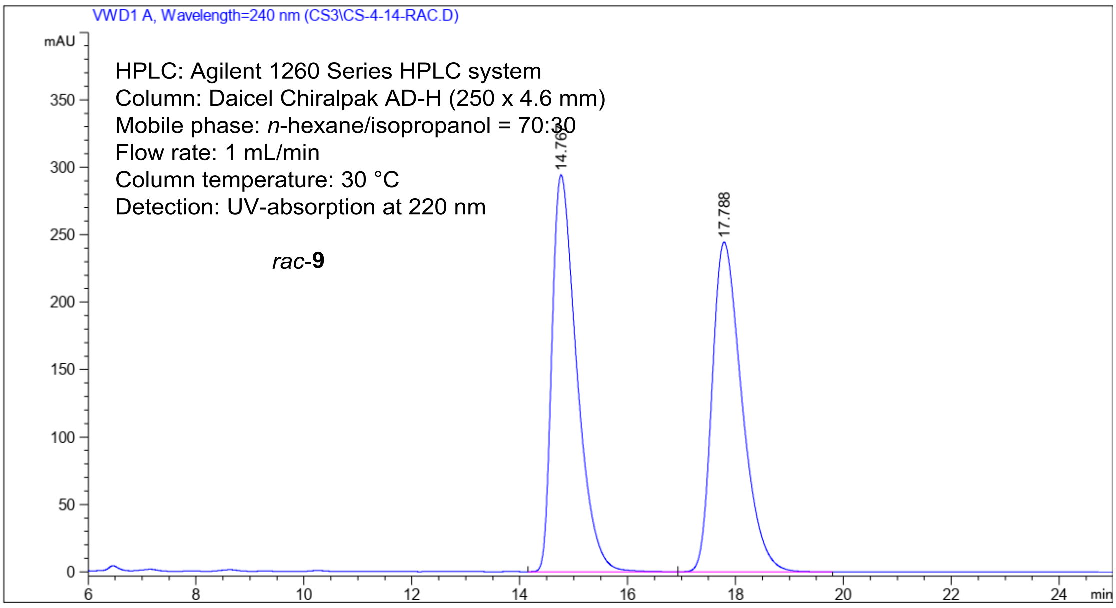

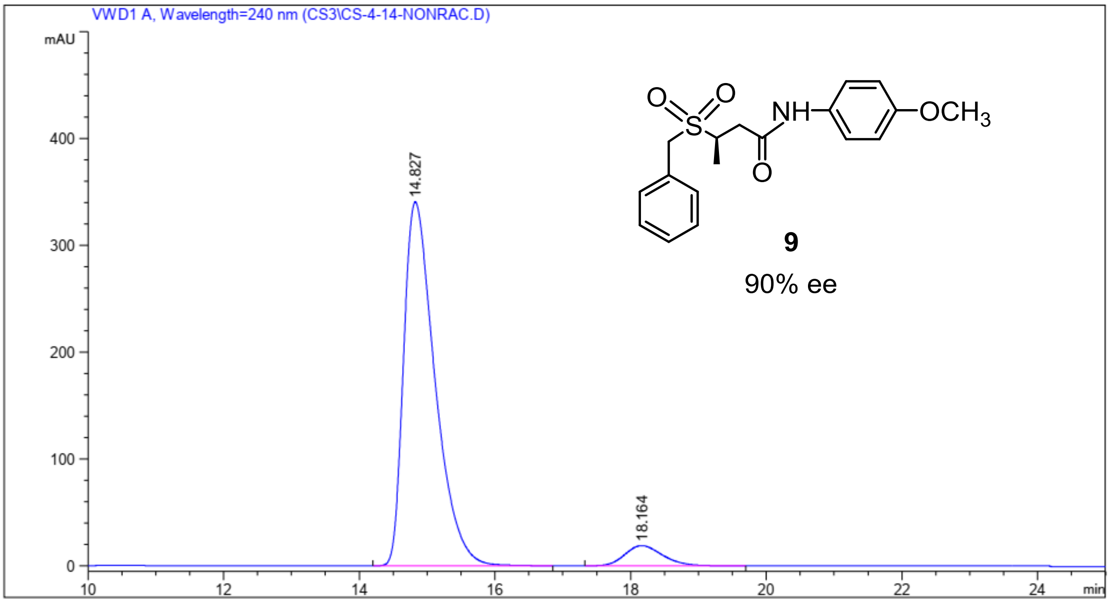

#	[min]		[min]	[mAU*s]	[mAU]	%
1	14.827	MM R	0.5606	1.15758e4	344.14117	94.7932
2	18.164	MM R	0.5918	635.83649	17.90621	5.2068

Supplementary Fig. 68 HPLC trace for the racemic reference *rac*-**9**, and non-racemic product **9** generated from the transformation of **3v**.

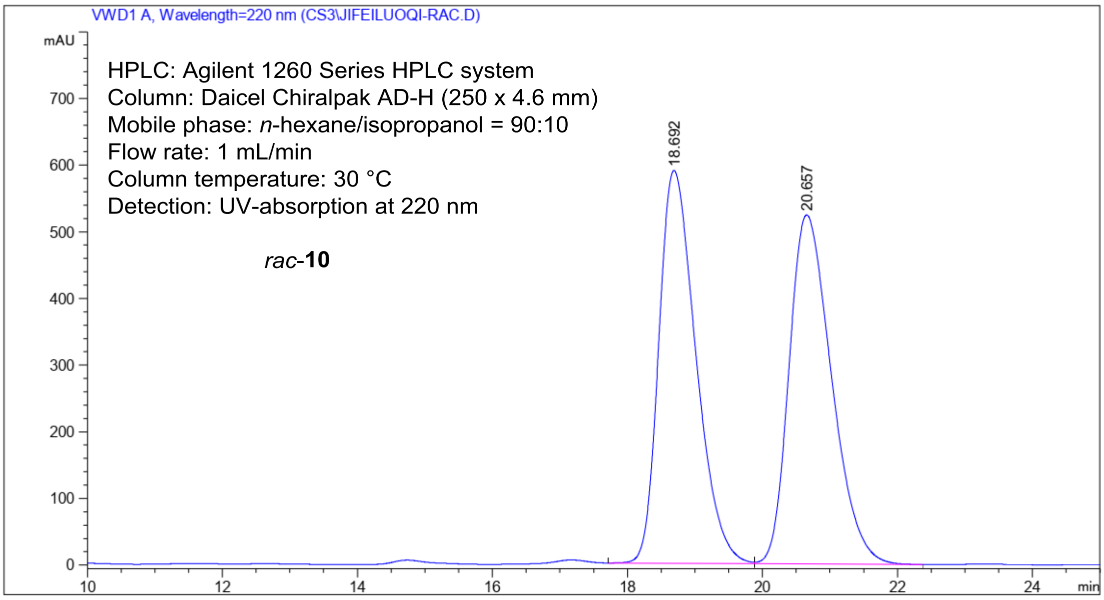

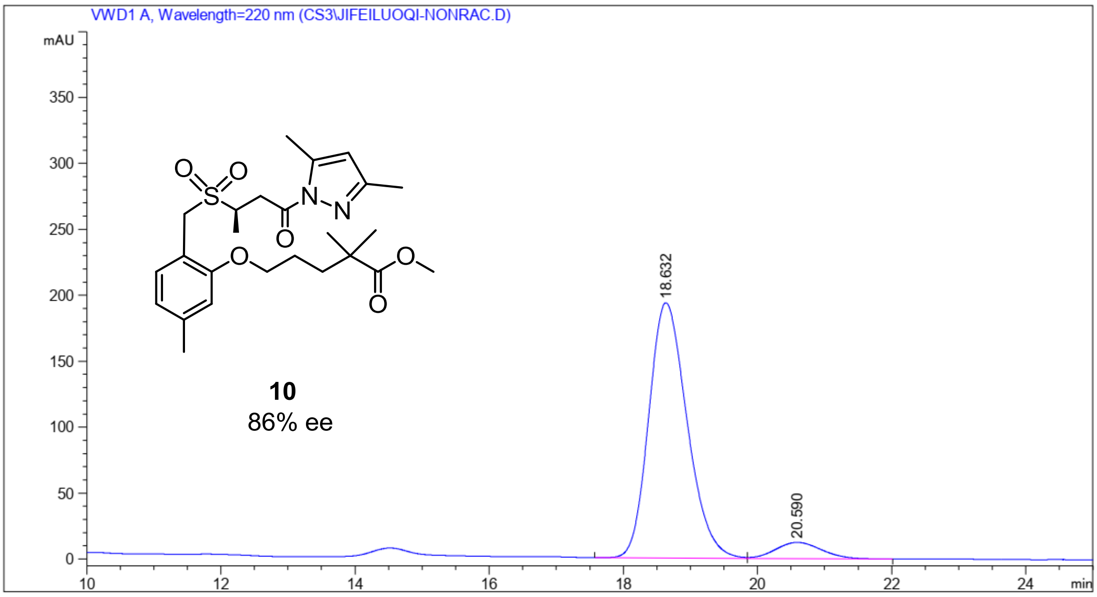

#	[min]		[min]	[mAU*s]	[mAU]	%
1	18.632	BV	0.6131	7664.78467	193.50002	92.9724
2	20.590	VB	0.7151	579.36731	12.26546	7.0276

Supplementary Fig. 69 HPLC trace for the racemic reference *rac*-**10**, and non-racemic product **10**.

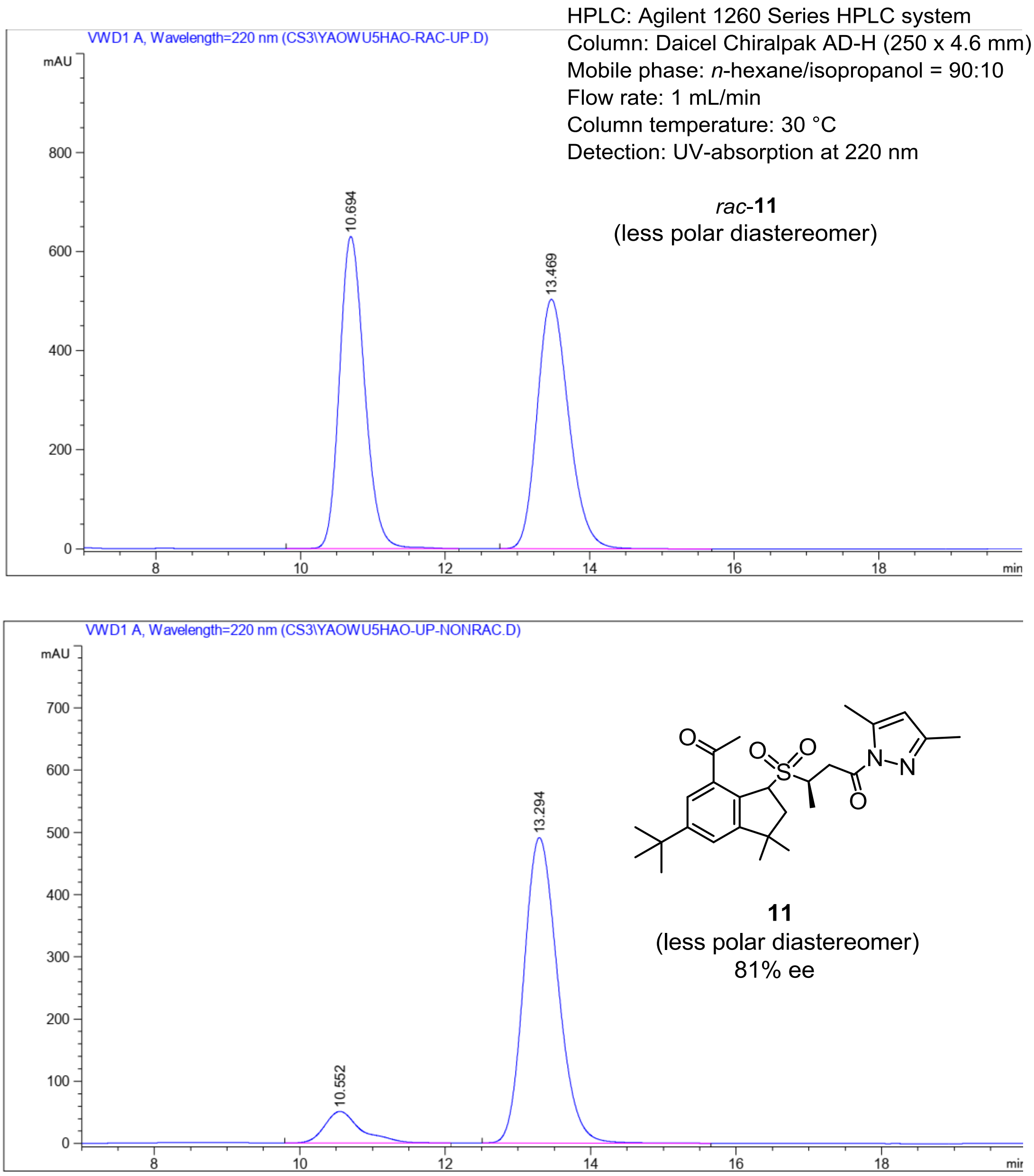

#	[min]		[min]	[mAU*s]	[mAU]	%
1	10.552	MM	0.5424	1674.40869	51.45013	9.5546
2	13.294	BB	0.5049	1.58502e4	490.64377	90.4454

Supplementary Fig. 70 HPLC trace for the racemic reference *rac*-**11** (less polar diastereomer), and non-racemic product **11** (less polar diastereomer).

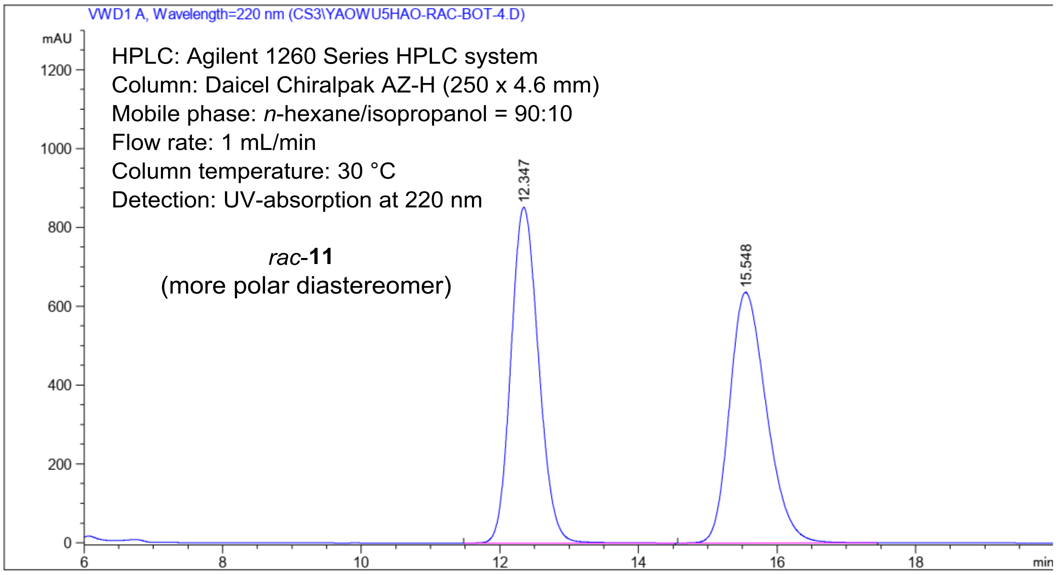

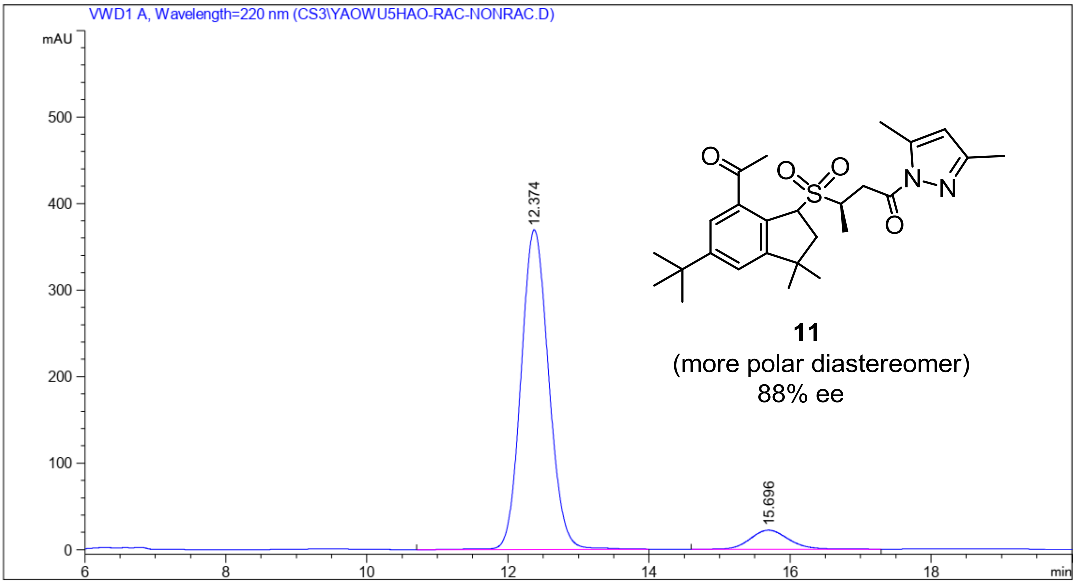

#	[min]		[min]	[mAU*s]	[mAU]	%
1	12.374	BB	0.4228	1.00295e4	369.40253	93.9998
2	15.696	MM R	0.5401	640.20941	19.75668	6.0002

Supplementary Fig. 71 HPLC trace for the racemic reference *rac*-**11** (more polar diastereomer), and non-racemic product **11** (more polar diastereomer).

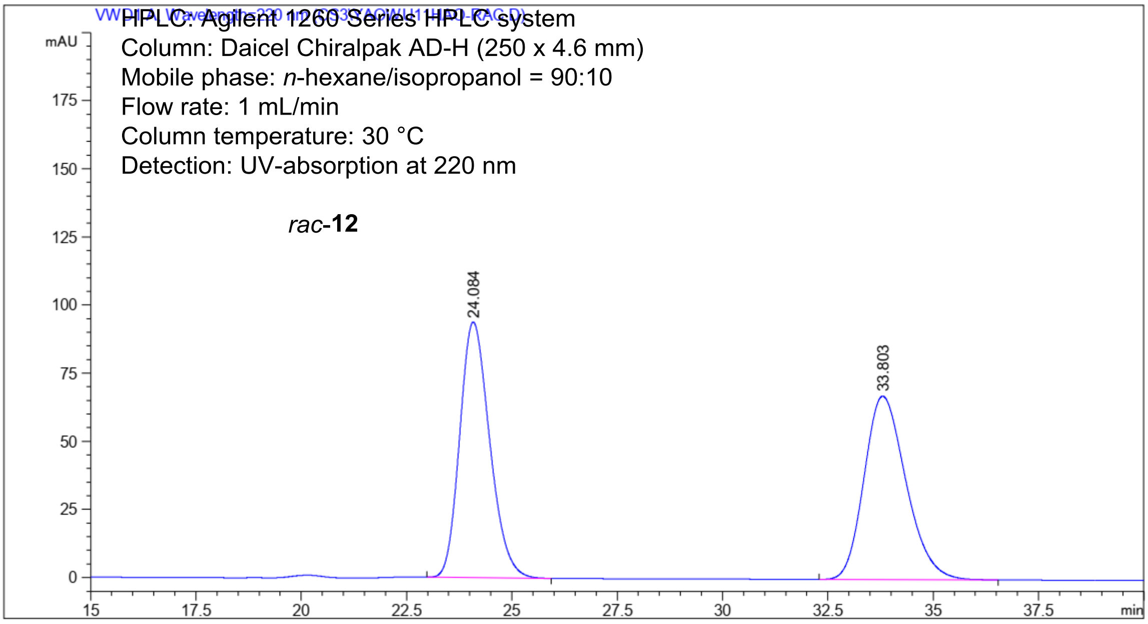

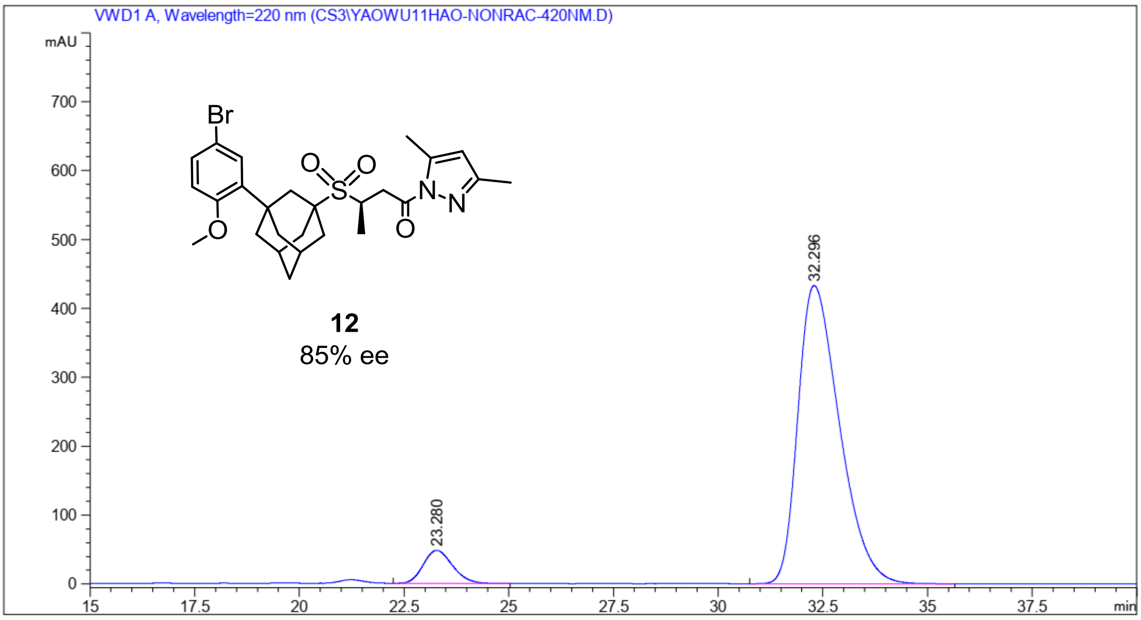

#	[min]		[min]	[mAU*s]	[mAU]	%
1	23.280	BB	0.7568	2337.21680	47.94188	7.2602
2	32.296	BB	1.0712	2.98550e4	432.58679	92.7398

Supplementary Fig. 72 HPLC trace for the racemic reference *rac*-**12**, and non-racemic product **12**.

7. X-Ray Diffraction

7.1 Crystal Structure of Chiral Nickel Catalyst [L6-Ni]

Crystallization procedure. A solution of $Ni(ClO_4)_2 \cdot 6H_2O$ (73.0 mg, 0.20 mmol) and chiral ligand **L6** (71.6 mg, 0.20 mmol) in CH_3CN (6.0 mL) and water (75 μL) was stirred at 80 °C for 5 h, the reaction was concentrated under reduced pressure. The residue was redissolved in a mixture of Et_2O:CH_2Cl_2=2:1. Single crystals were obtained after evaporation under ambient conditions for 5 days.

Data collection and solution. Data was collected on a XtaLAB Synergy four-circle diffractometer with monochromatic Cu Kα radiation (λ = 1.54184 Å) at 100 K. Data reduction and absorption correction were applied by using the multi-scan program. The structures were determined and refined using full-matrix least-squares based on F^2 with SHELXT and SHELXL within Olex2. The structure is shown on Supplementary Fig. 73. Crystallographic data for chiral nickel complex **[L6-Ni]** has been deposited with the Cambridge Crystallographic Data Centre as supplementary publication number CCDC 2054187.

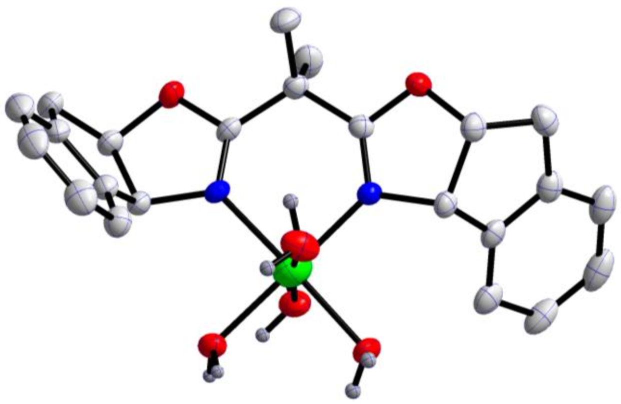

Supplementary Fig. 73 Ortep drawing of compound **[L6-Ni]** with thermal ellipsoids.

7.2 Crystal Structure of Chiral Product 3b

Crystallization procedure. A solution of compound **3b** (29.8 mg, 0.10 mmol) was dissolved in a mixture of MeOH (6 mL) and CH_2Cl_2 (2 mL). Single crystals were obtained after evaporation under ambient conditions for 7 days.

Data collection and solution. Data was collected on a XtaLAB Synergy four-circle diffractometer with monochromatic Cu Kα radiation (λ = 1.54184 Å) at 100 K. Data reduction and absorption correction were applied by using the multi-scan program. The structures were determined and refined using full-matrix least-squares based on F^2 with SHELXT and SHELXL within Olex2. The structure is shown on Supplementary Fig. 74. Crystallographic data for **3b** has been deposited with the Cambridge Crystallographic Data Centre as supplementary publication number CCDC 2028396.

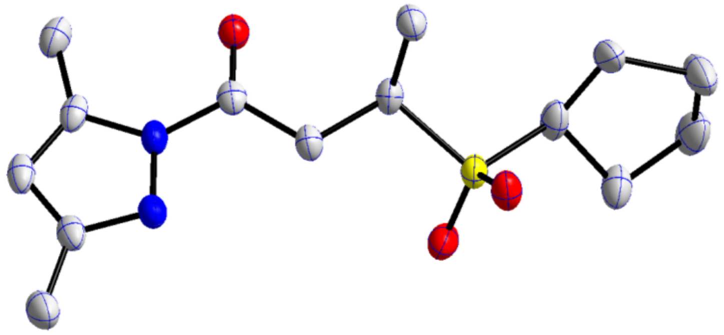

Supplementary Fig. 74 Ortep drawing of compound **3b** with thermal ellipsoids.

Table 2. Data collection and refinement statistics for the compounds **[L6-Ni]** and **3b.**

	**[L6-Ni]**	**3b**
Empirical formula	$C_{46}H_{60}Cl_3N_5Ni_2O_{27}$	$C_{14}H_{22}N_2O_3S$
Formula weight	669.38	298.39
Temperature (K)	99.9(8)	99.9(5)
Wavelength (Å)	1.54814	1.54814
Crystal system	Orthorhombic	Orthorhombic

Space group	$P2_12_12_1$	$P2_12_12_1$
Cell dimensions		
a, b, c (Å)	9.3755, 20.3342, 29.8106	6.7381, 10.3679, 21.8408
α, β, γ (°)	90, 90, 90	90, 90, 90
Volume (Å^3)	5683.19(1)	1525.80(8)
Z	4	4
Density (calculated, mg/m^3)	1.565	1.299
Absorption coefficient (mm^{-1})	2.920	1.966
F(000)	2776.0	640.0
Crystal size (mm^3)	0.2 x 0.05 x 0.03	0.1 x 0.05 x 0.03
Theta range for data collection/ °	5.26 to 150.536	9.442 to 133.838
Index ranges	-11<=h<=11, -21<=k<=25, -37<=l<=33	-7<=h<=8, -12<=k<=11, -25<=l<=26
Reflections collected	32814	9452
Independent reflections	11409 [R_{int} = 0.0417, R_{sigma} = 0.0441]	2699 [R_{int} = 0.0473, R_{sigma} = 0.0439]
Completeness	99.9 %	100 %
Absorption correction	multi-scan	multi-scan
Refinement method	Full-matrix least-squares on F^2	Full-matrix least-squares on F^2
Data / restraints / parameters	11409 / 1031 / 869	2699 / 0 / 184
Goodness-of-fit on F^2	1.029	1.051
Final *R* indices [I>=2σ (I)]	R_1 = 0.0470, wR_2 = 0.1236	R_1 = 0.0365, wR_2 = 0.0915
R indices (all data)	R_1 = 0.0501, wR_2 = 0.1263	R_1 = 0.0422, wR_2 = 0.0990
Flack parameter	0.004(6)	0.018(13)
Largest diff. peak and hole (e.Å^{-3})	1.31 and -0.63	0.47 and -0.25

8. ^{1}H and ^{13}C NMR Spectrum

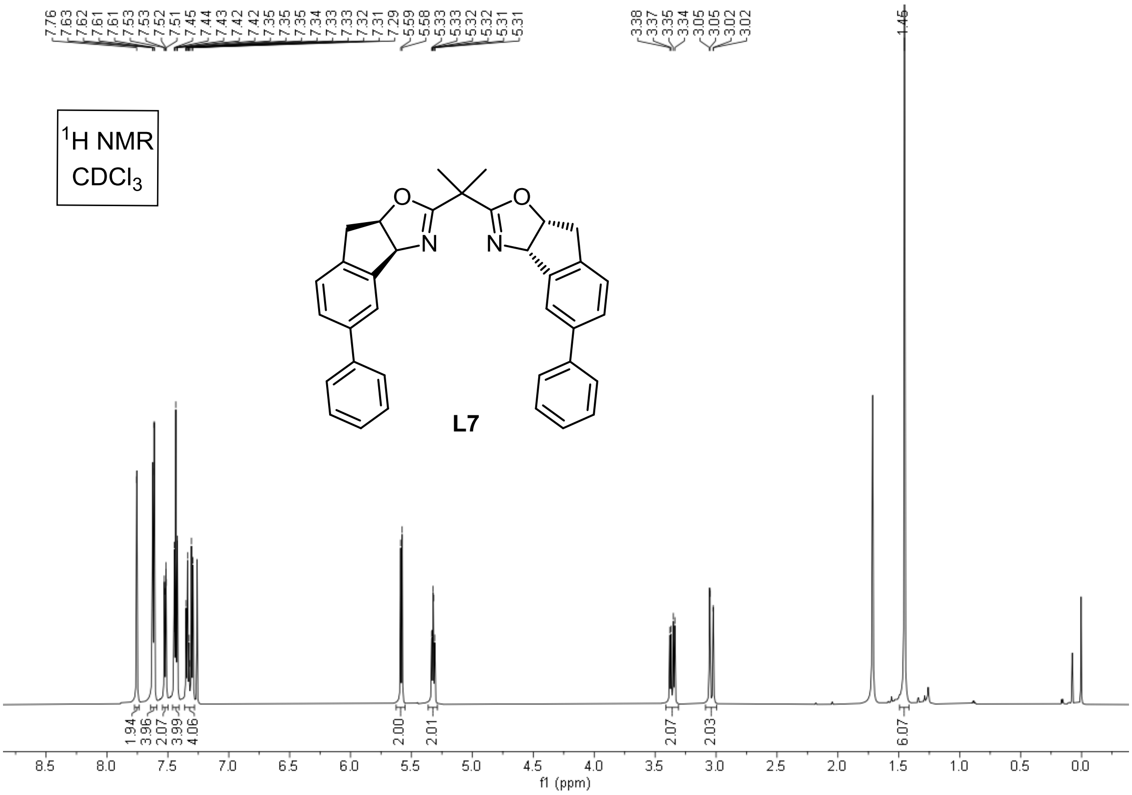

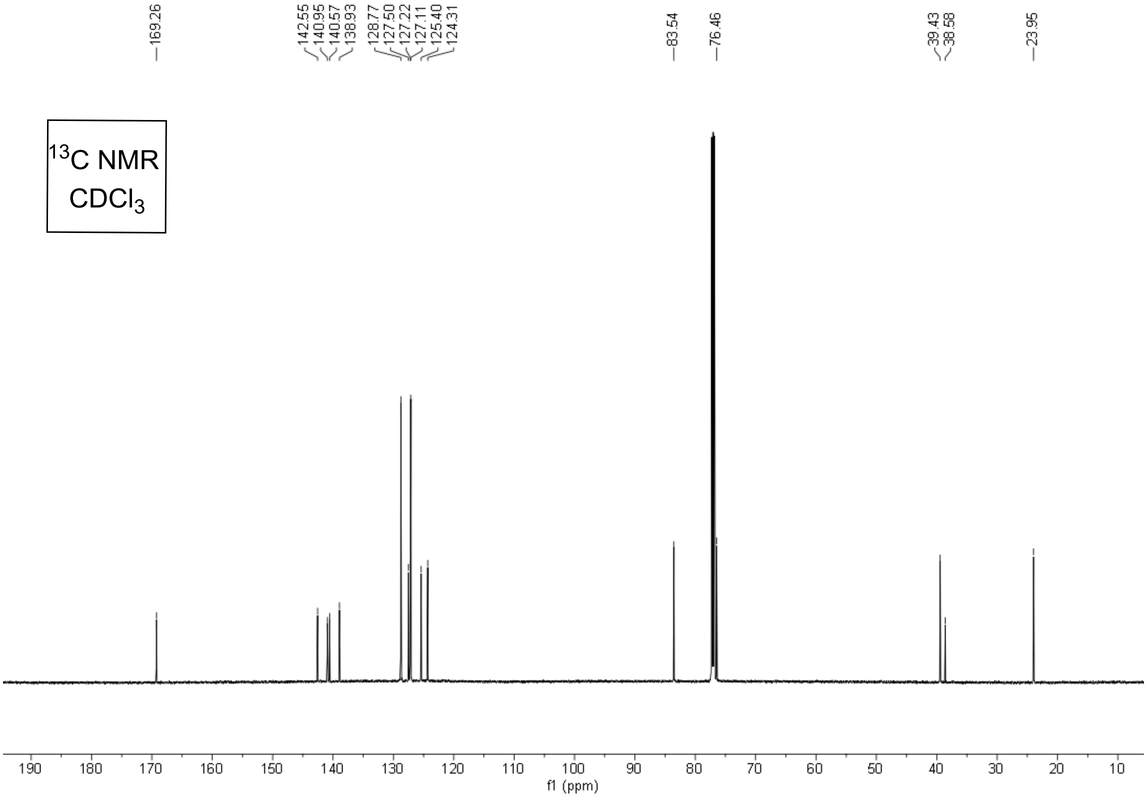

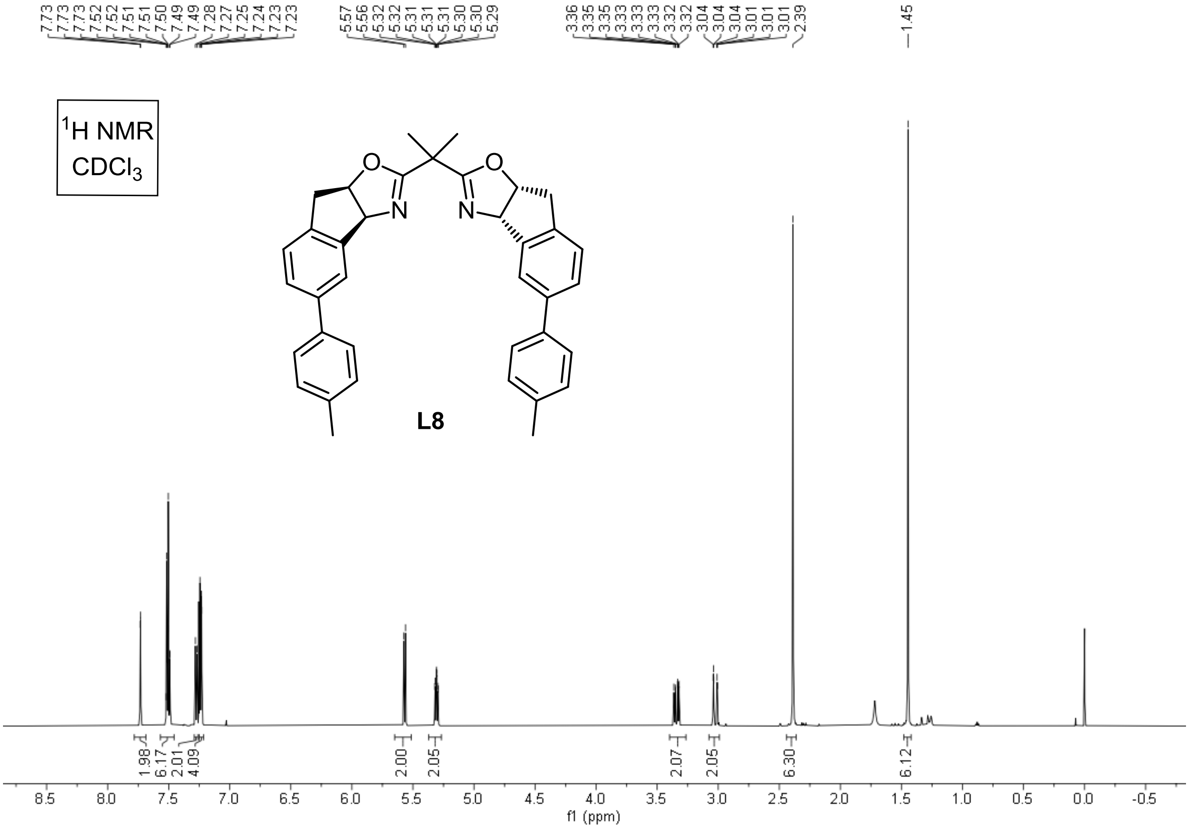
7.73 7.73 7.73 7.52 7.52 7.51 7.51 7.50 7.49 7.49 7.28 7.27 7.25 7.24 7.23 7.23
5.57 5.56 5.32 5.32 5.31 5.31 5.31 5.30 5.30 5.29
3.36 3.35 3.35 3.33 3.33 3.33 3.32 3.32 3.04 3.04 3.04 3.01 3.01 3.01 2.39
1.45
^{1}H NMR
CDCl$_3$
O
N
L8
1.98 6.17 2.01 4.09 2.00 2.05 2.07 2.05 6.30 6.12
f1 (ppm)

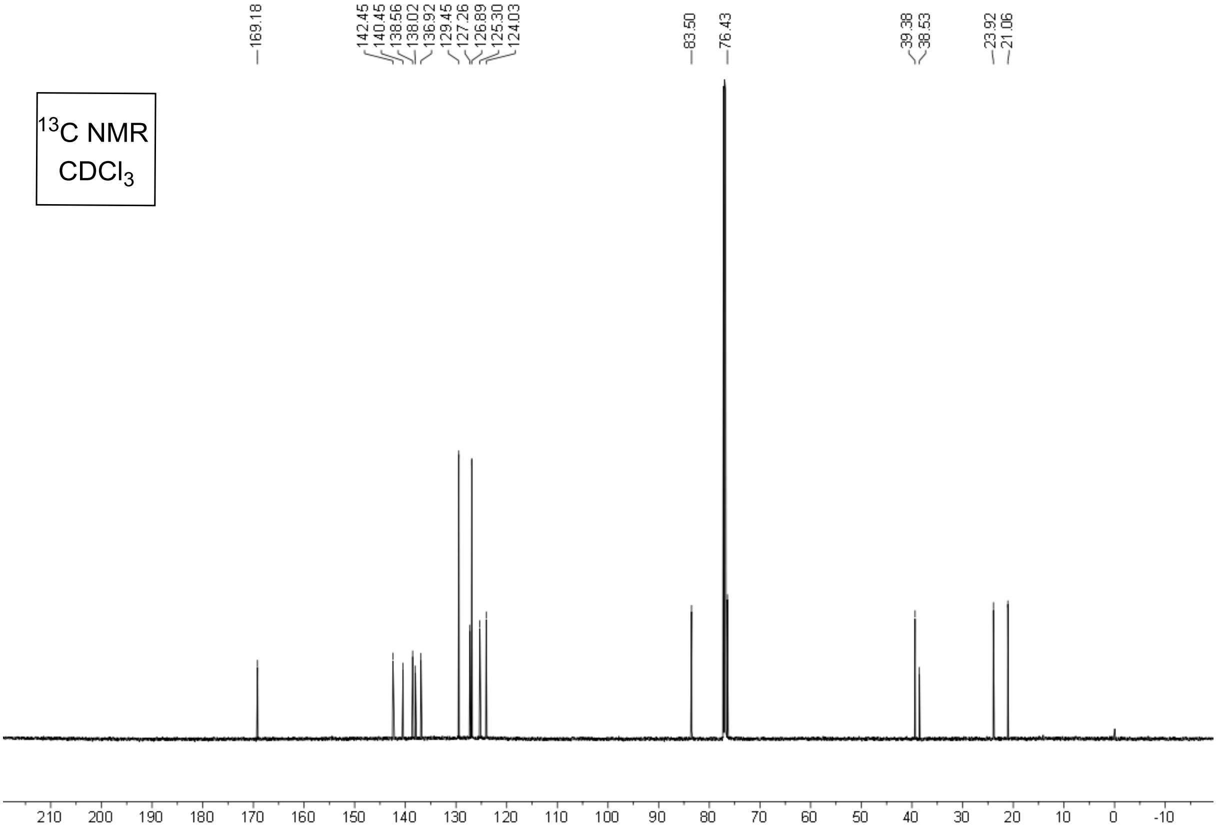
169.18
142.45 140.45 138.56 138.02 136.92 129.45 127.26 126.89 125.30 124.03
83.50
76.43
39.38 38.53
23.92 21.06
^{13}C NMR
CDCl$_3$
f1 (ppm)

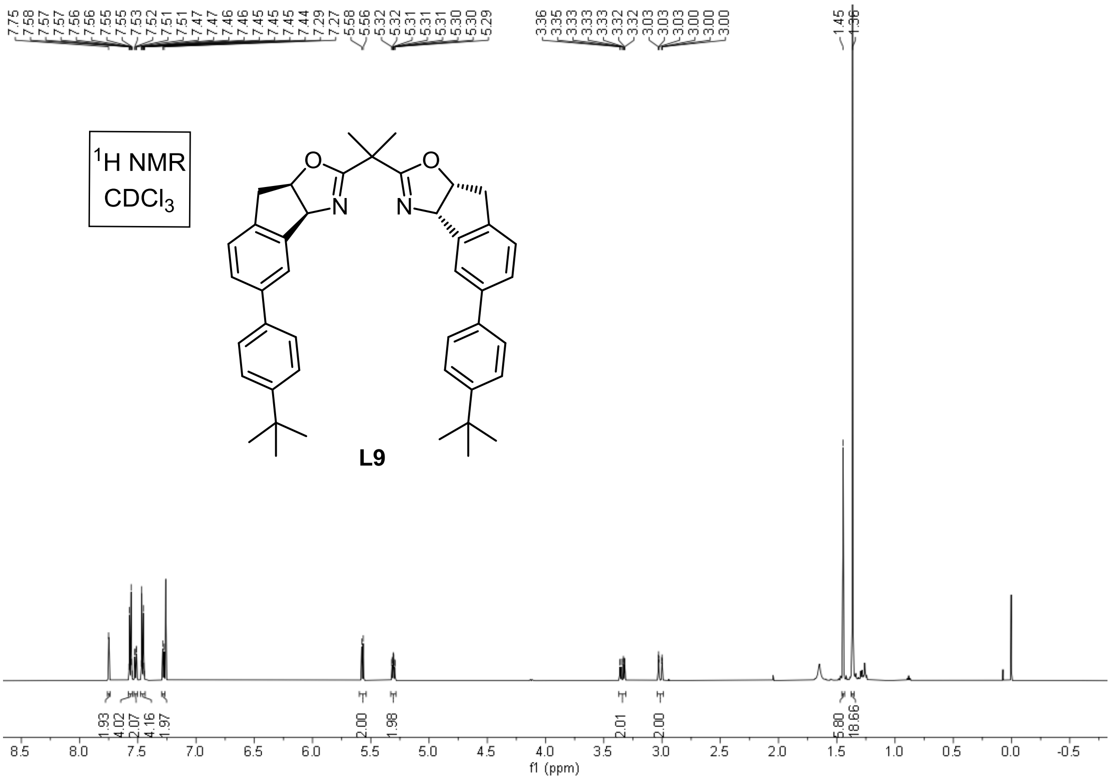
^{1}H NMR
CDCl$_3$
L9
f1 (ppm)

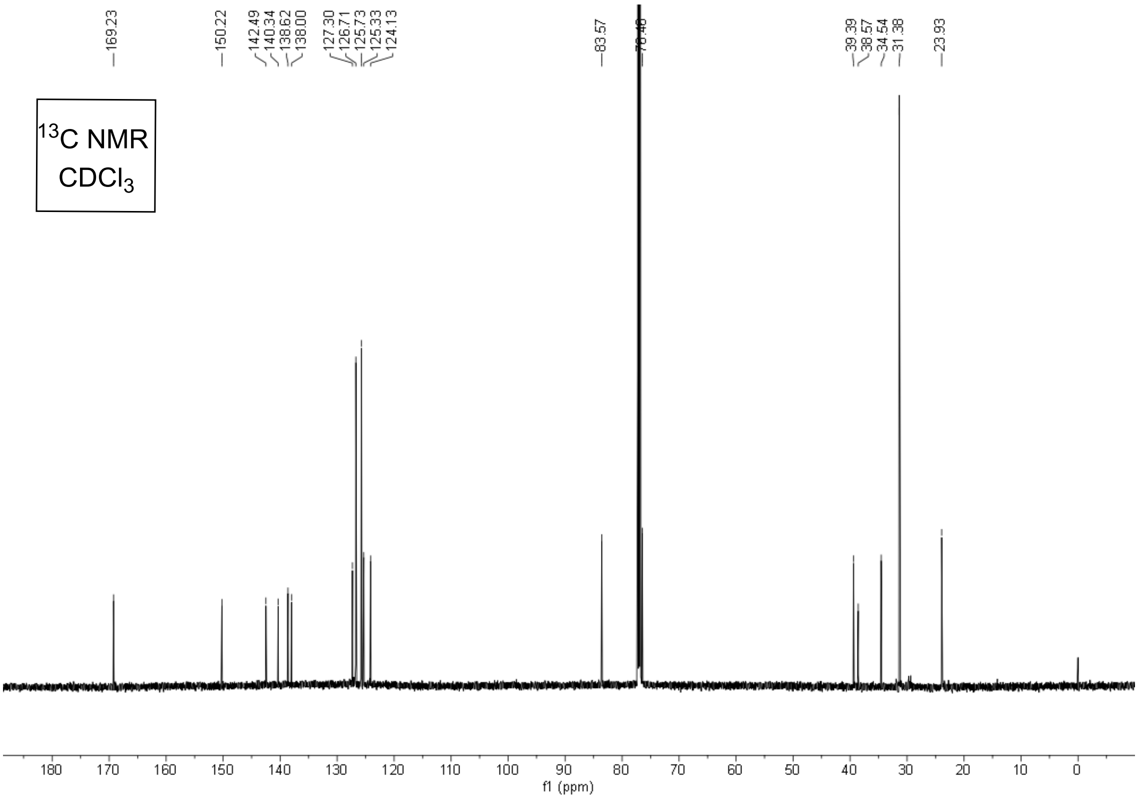
^{13}C NMR
CDCl$_3$
f1 (ppm)

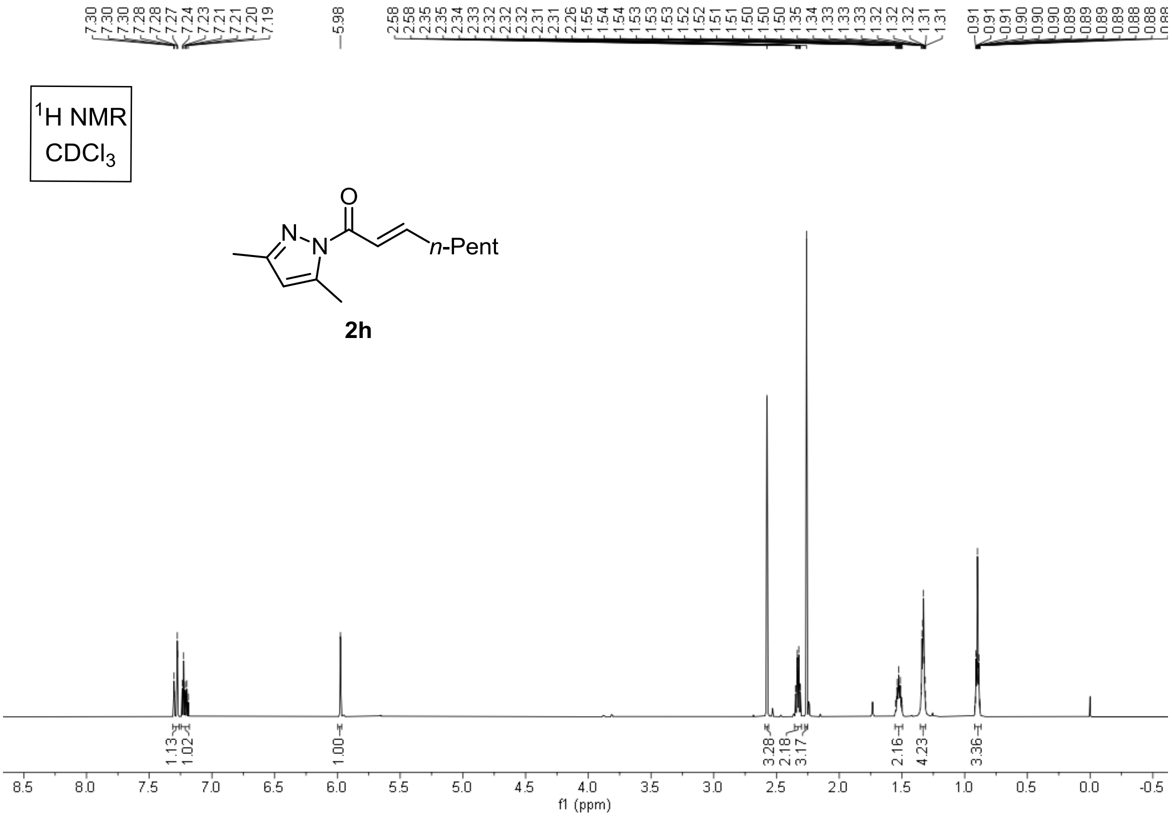

7.30 7.30 7.30 7.28 7.27 7.24 7.23 7.21 7.21 7.20 7.19
5.98
2.58 2.58 2.35 2.35 2.34 2.33 2.32 2.32 2.32 2.31 2.31 2.26 1.55 1.54 1.54 1.53 1.53 1.53 1.52 1.52 1.51 1.51 1.50 1.50 1.50 1.35 1.34 1.33 1.33 1.33 1.32 1.32 1.32 1.32 1.31 1.31
0.91 0.91 0.91 0.90 0.90 0.90 0.89 0.89 0.89 0.88 0.88
^{1}H NMR
$CDCl_3$
O
N
N
n-Pent
2h
1.13
1.02
1.00
3.28
2.18
3.17
2.16
4.23
3.36
f1 (ppm)

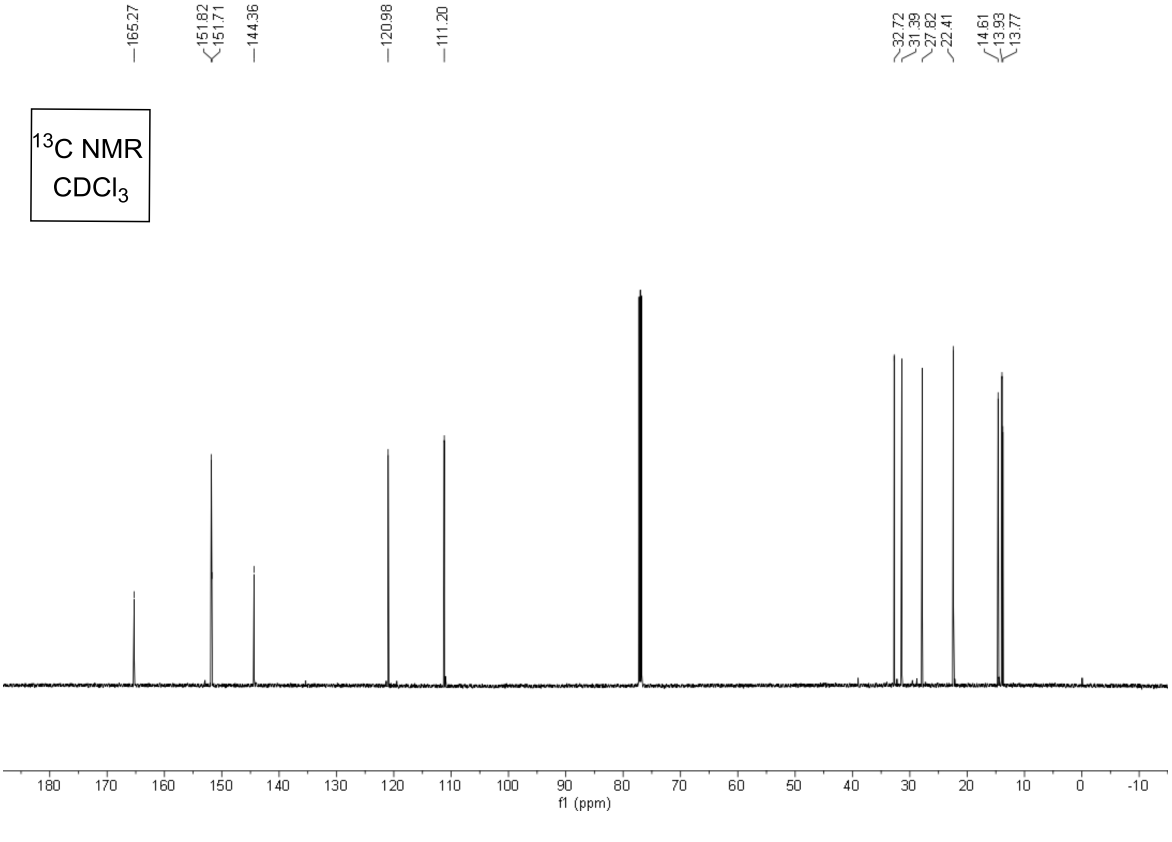

165.27
151.82
151.71
144.36
120.98
111.20
32.72
31.39
27.82
22.41
14.61
13.93
13.77
^{13}C NMR
$CDCl_3$
f1 (ppm)

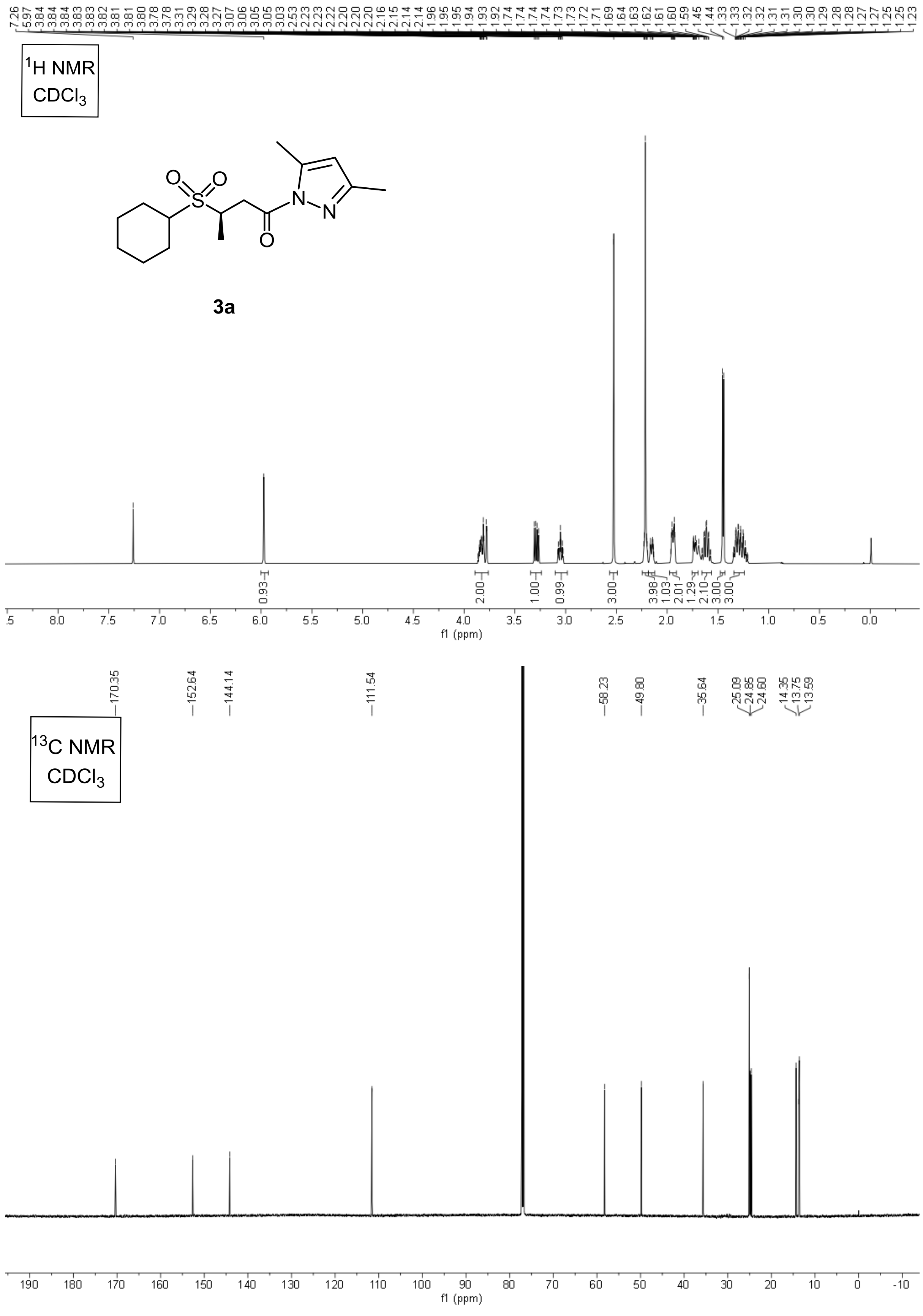

^{1}H NMR
CDCl$_3$
3a
^{13}C NMR
CDCl$_3$
f1 (ppm)

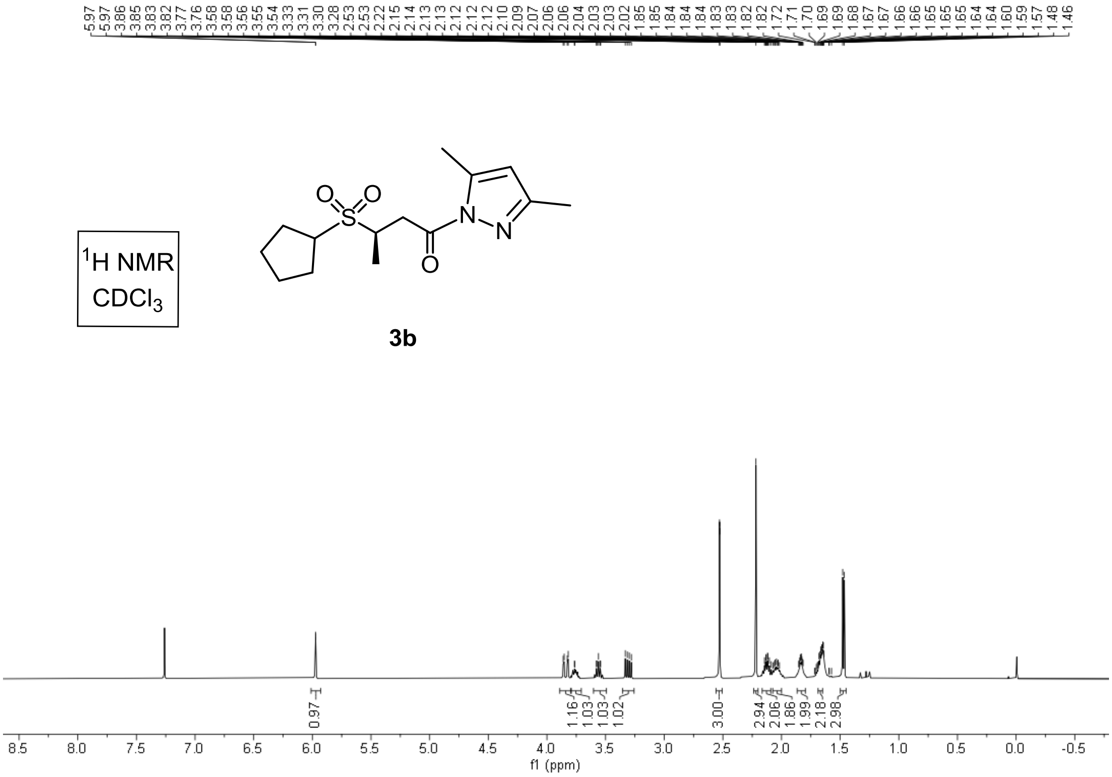
1H NMR
CDCl3
3b
f1 (ppm)

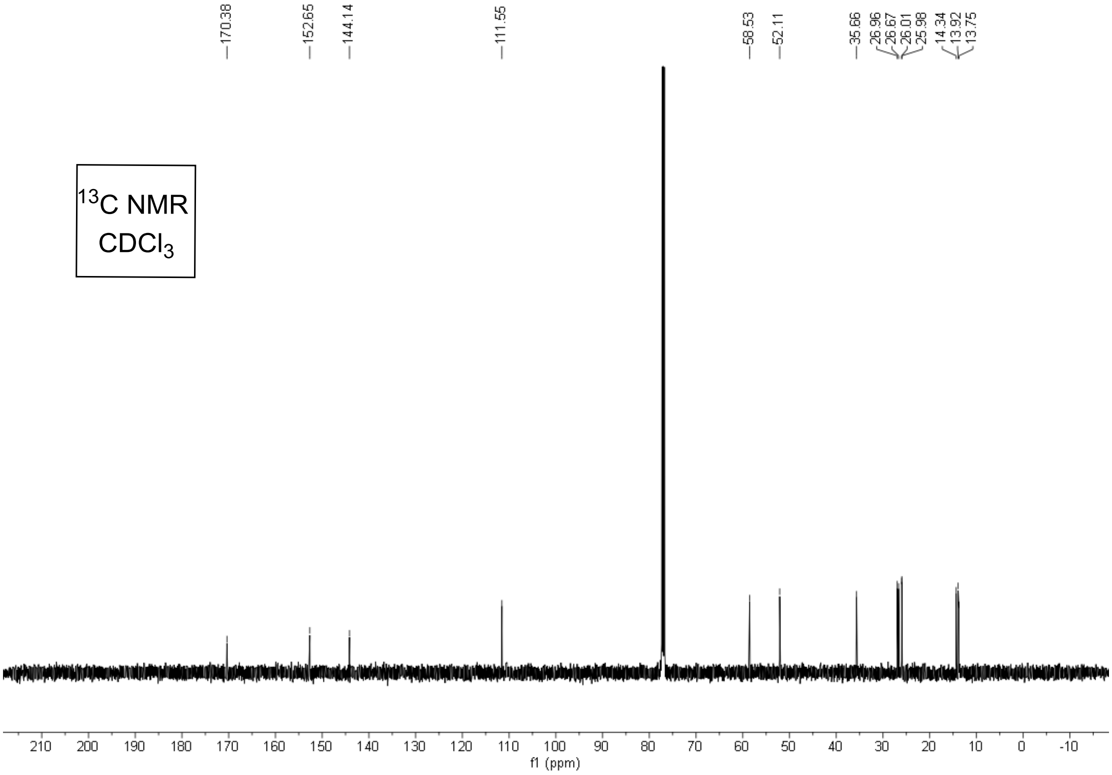
170.38
152.65
144.14
111.55
58.53
52.11
35.66
26.96
26.67
26.01
25.98
14.34
13.92
13.75
13C NMR
CDCl3
f1 (ppm)

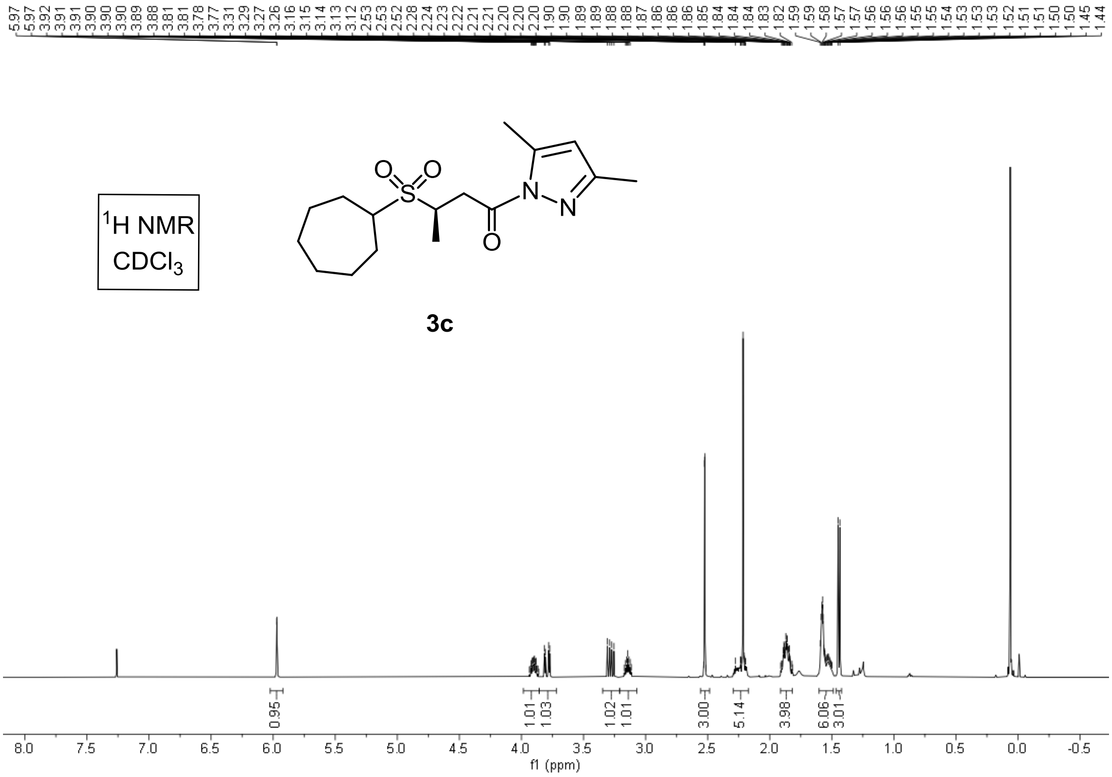

^{1}H NMR
$CDCl_3$

3c

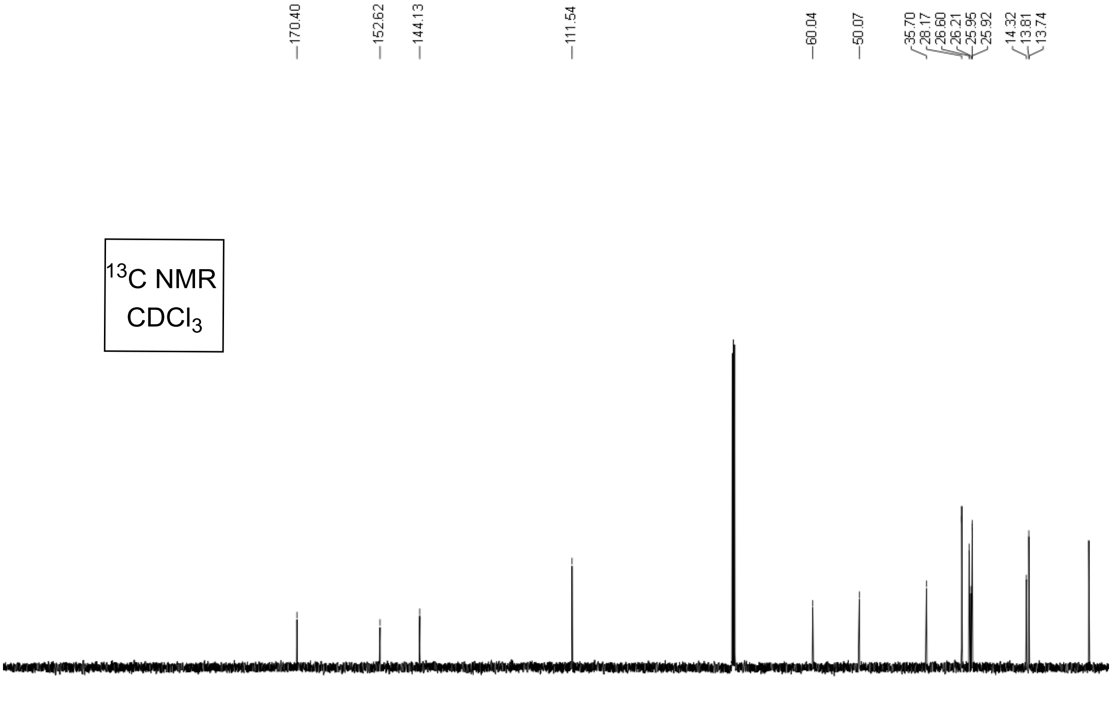

^{13}C NMR
$CDCl_3$

^{1}H NMR
$CDCl_3$

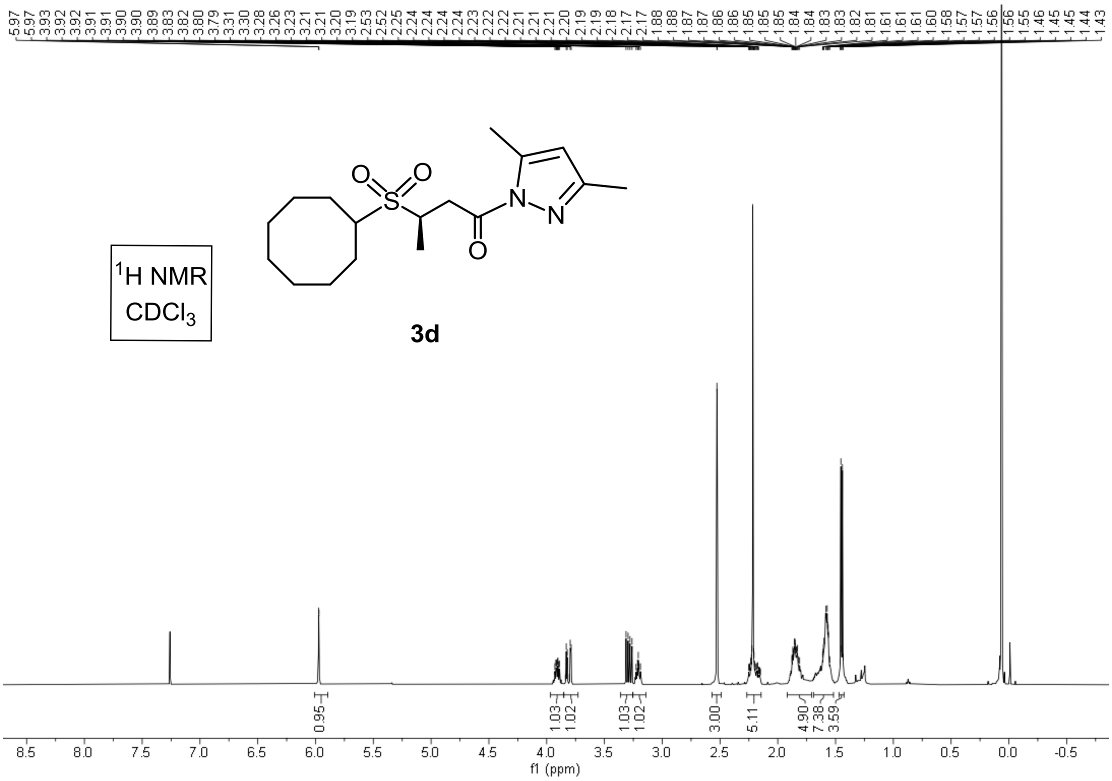

3d

^{13}C NMR
$CDCl_3$

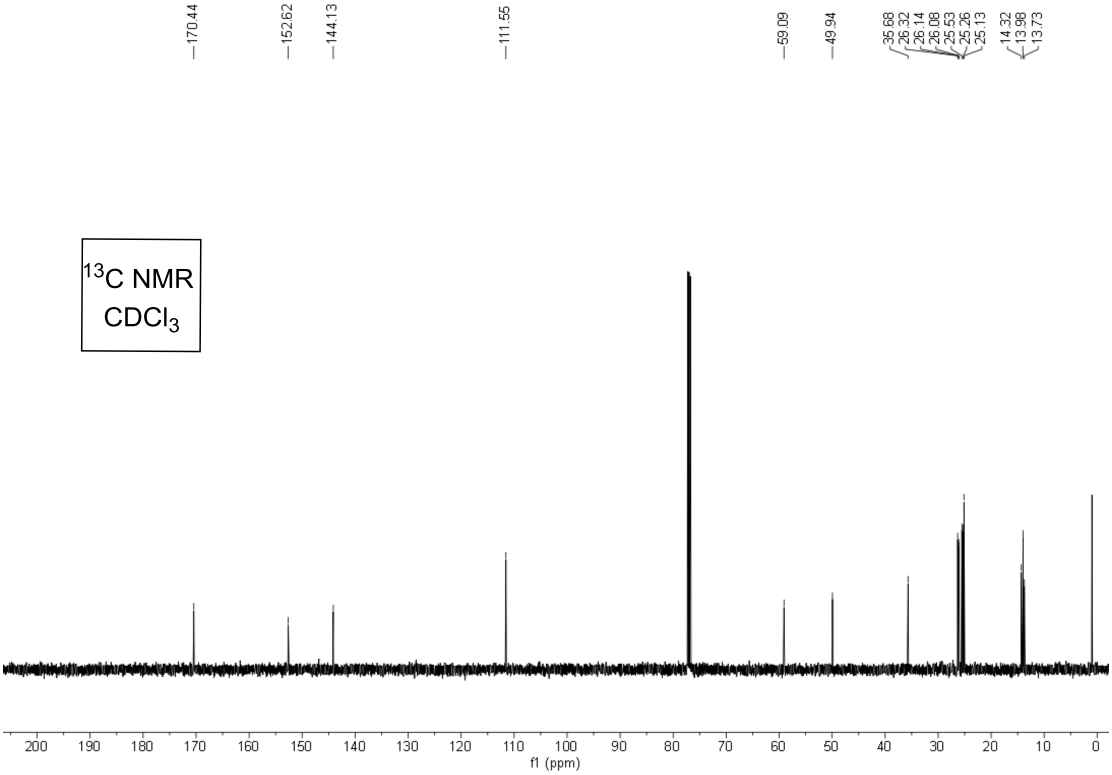

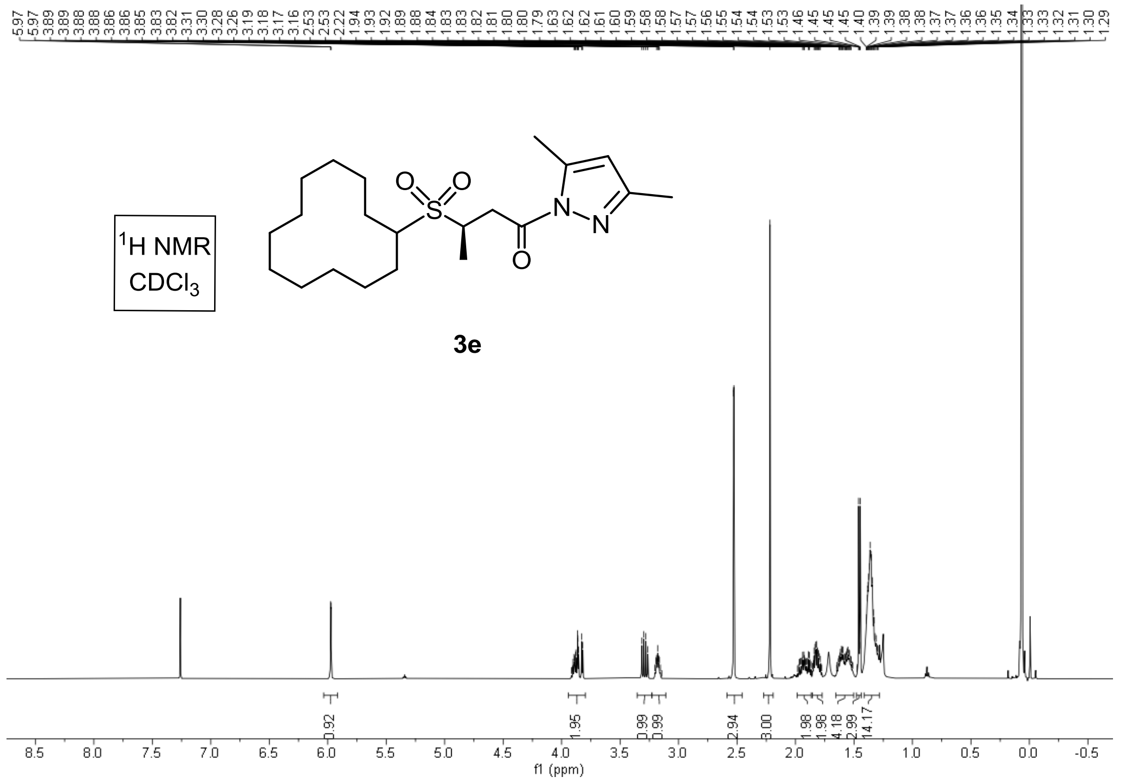
1H NMR
CDCl3
3e
f1 (ppm)

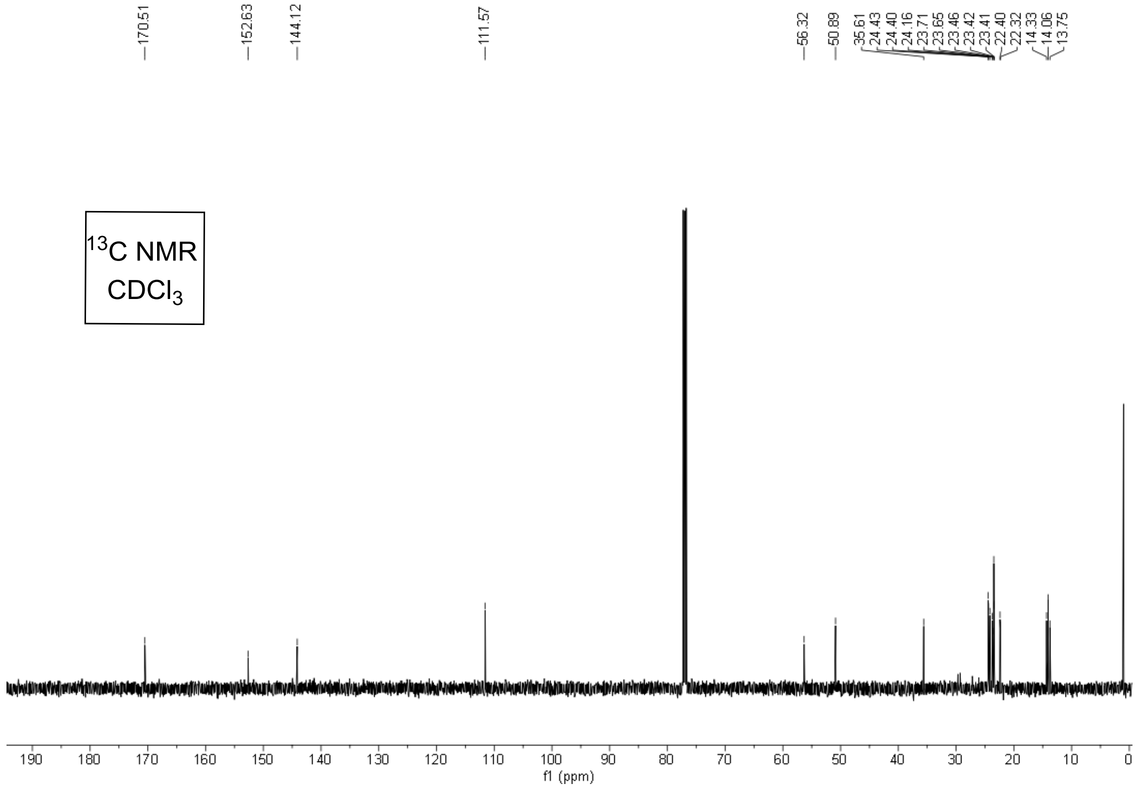
13C NMR
CDCl3
f1 (ppm)

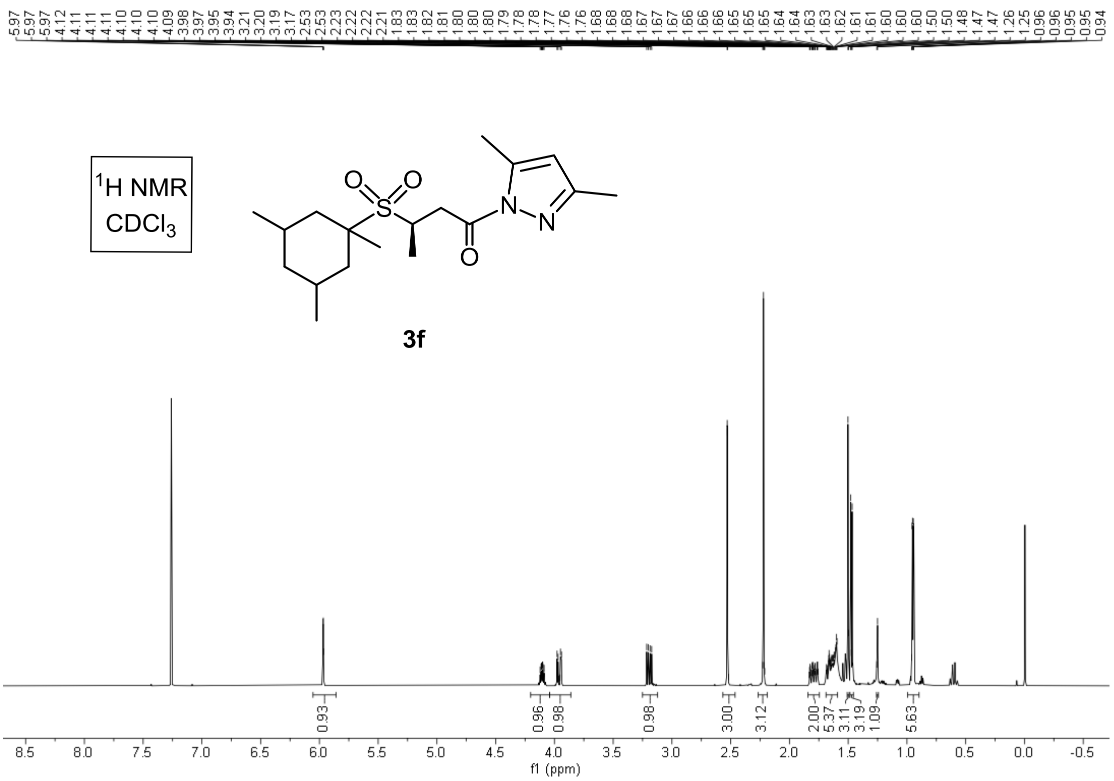
5.97 5.97 5.97 4.12 4.11 4.11 4.11 4.10 4.10 4.09 3.98 3.97 3.95 3.94 3.21 3.20 3.19 3.17 2.53 2.23 2.22 2.22 2.21 1.83 1.82 1.81 1.80 1.80 1.79 1.78 1.78 1.77 1.76 1.76 1.68 1.68 1.67 1.67 1.67 1.66 1.66 1.65 1.65 1.65 1.64 1.64 1.63 1.63 1.62 1.61 1.61 1.60 1.60 1.60 1.50 1.48 1.47 1.47 1.26 1.25 0.96 0.96 0.95 0.94
1H NMR
CDCl3
3f
0.93
0.96
0.98
0.98
3.00
3.12
2.00
5.37
3.11
3.19
1.09
5.63
f1 (ppm)

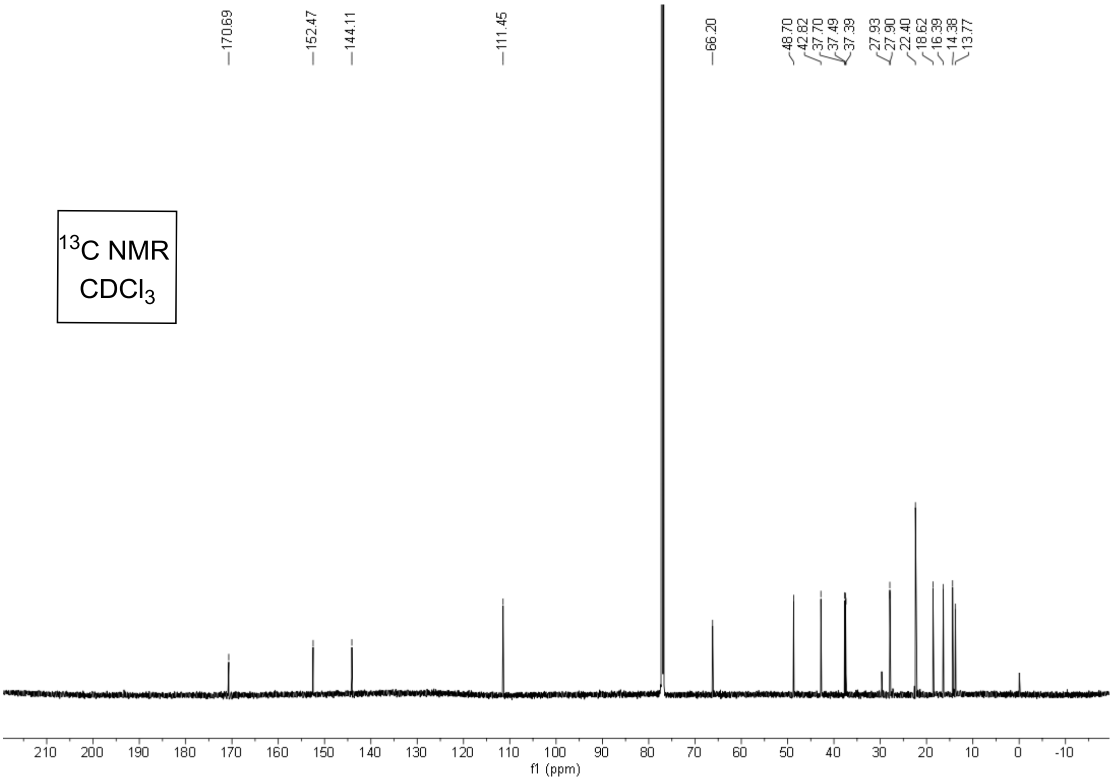
170.69
152.47
144.11
111.45
66.20
48.70 42.82 37.70 37.49 37.39 27.93 27.90 22.40 18.62 16.39 14.38 13.77
13C NMR
CDCl3
f1 (ppm)

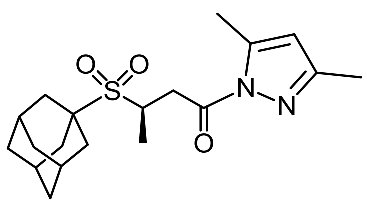

3g

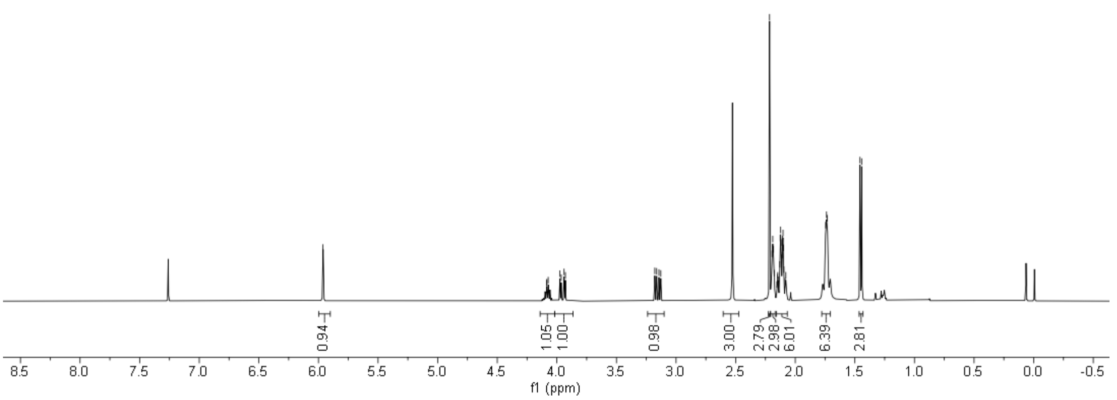

¹H NMR
CDCl3
5.96
4.09
4.08
4.07
3.97
3.96
3.94
3.93
3.18
3.16
3.14
3.13
2.53
2.22
2.20
2.19
2.18
2.18
2.15
2.15
2.14
2.13
2.13
2.12
2.12
2.12
2.11
2.11
2.10
2.10
2.10
2.08
1.75
1.74
1.73
1.46
0.94
1.05
1.00
0.98
3.00
2.79
2.98
6.01
6.39
2.81
f1 (ppm)

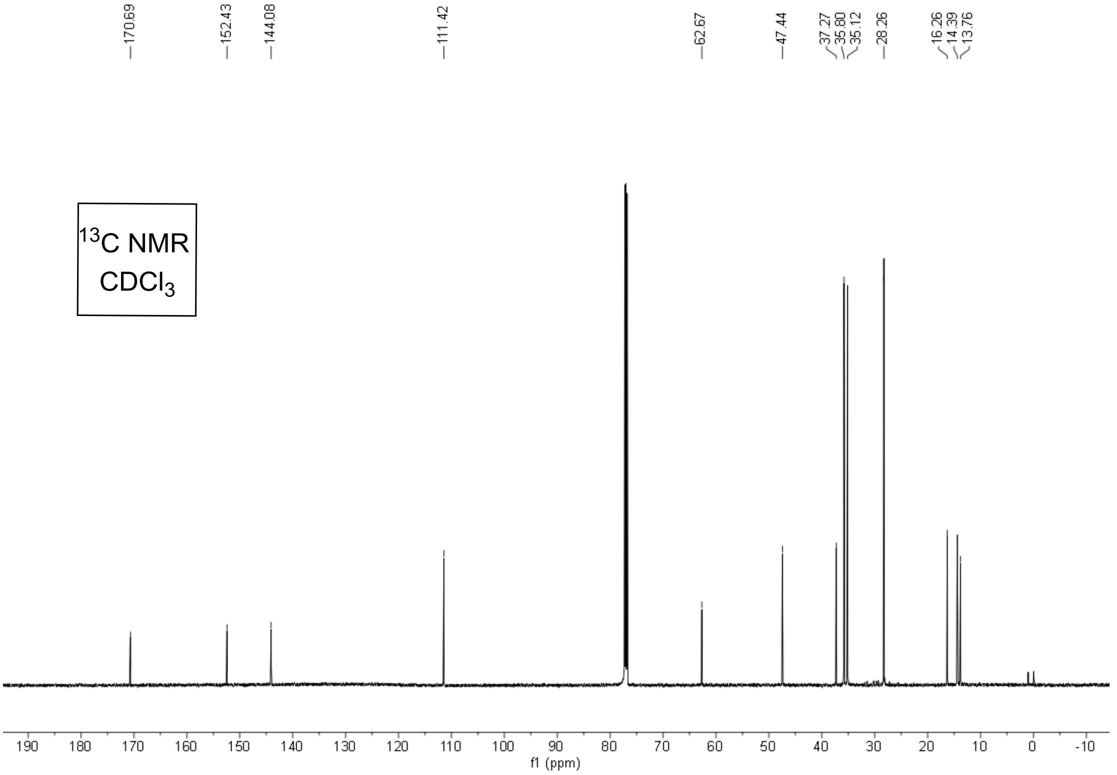

¹³C NMR
CDCl3
170.69
152.43
144.08
111.42
62.67
47.44
37.27
35.80
35.12
28.26
16.26
14.39
13.76
f1 (ppm)

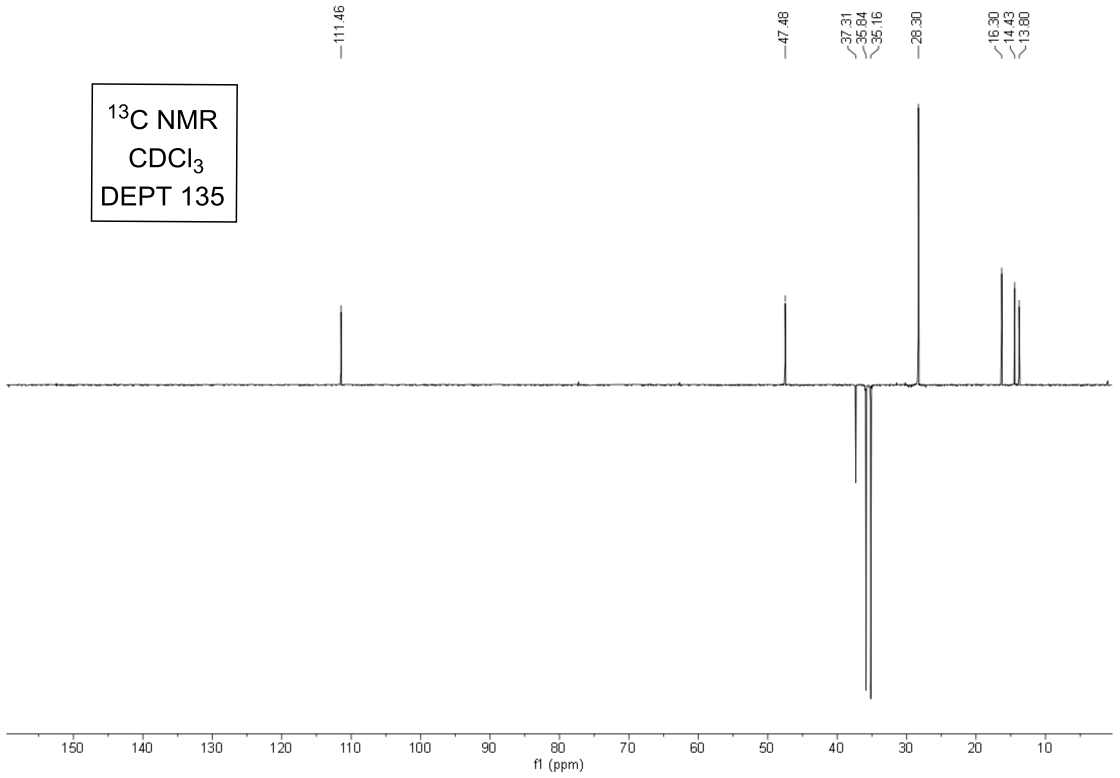
111.46
47.48
37.31
35.84
35.16
28.30
16.30
14.43
13.80
^{13}C NMR
$CDCl_3$
DEPT 135
150
140
130
120
110
100
90
80
70
60
50
40
30
20
10
f1 (ppm)

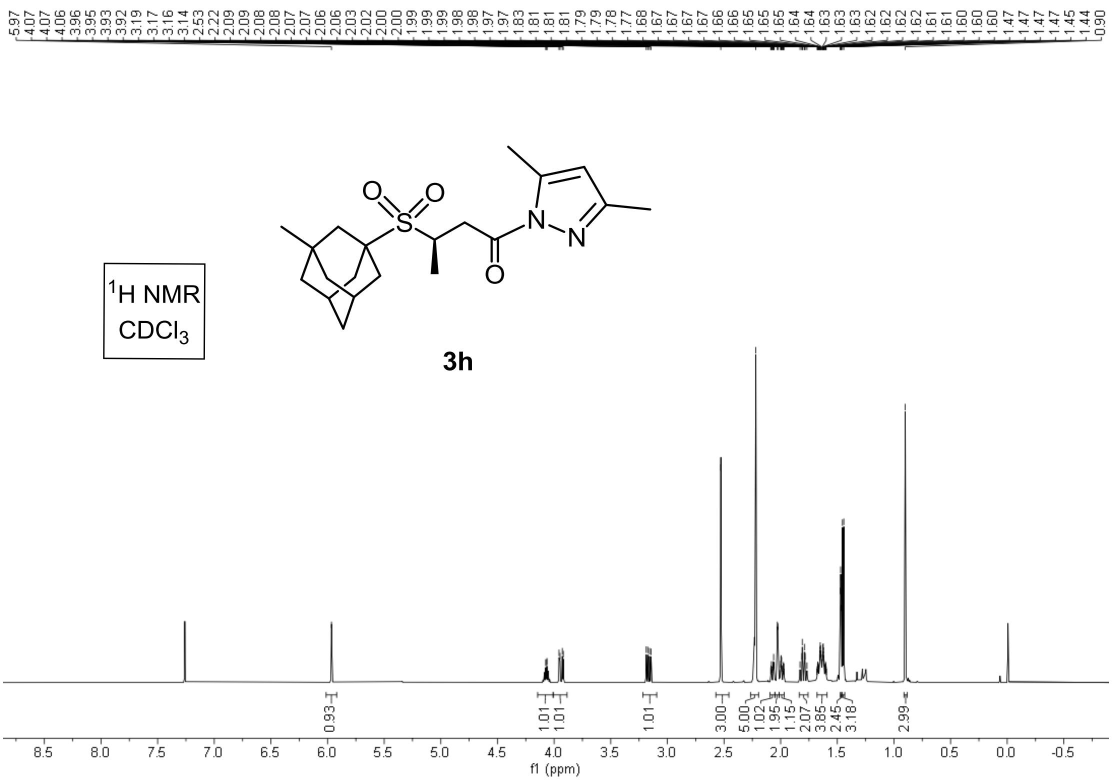
5.97 4.07 4.07 4.06 3.96 3.95 3.93 3.92 3.19 3.17 3.16 3.14 2.53 2.22 2.09 2.09 2.08 2.08 2.07 2.07 2.06 2.06 2.03 2.02 2.00 2.00 1.99 1.99 1.98 1.98 1.97 1.97 1.83 1.81 1.81 1.81 1.79 1.79 1.78 1.77 1.68 1.67 1.67 1.67 1.66 1.66 1.65 1.65 1.64 1.64 1.63 1.63 1.63 1.62 1.62 1.62 1.61 1.61 1.60 1.60 1.60 1.47 1.47 1.47 1.47 1.45 1.44 0.90
1H NMR
CDCl3
3h
0.93 1.01 1.01 1.01 3.00 5.00 1.02 1.95 1.15 2.07 3.85 2.45 3.18 2.99
f1 (ppm)

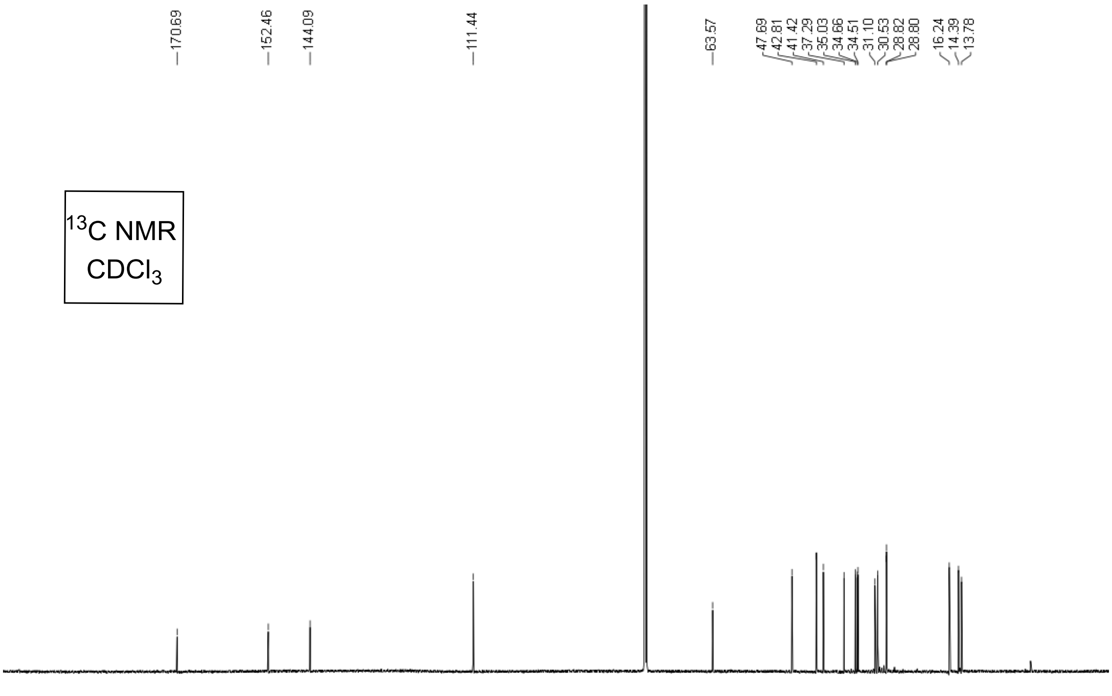
170.69 152.46 144.09 111.44 63.57 47.69 42.81 41.42 37.29 35.03 34.66 34.51 31.10 30.53 28.82 28.80 16.24 14.39 13.78
13C NMR
CDCl3
f1 (ppm)

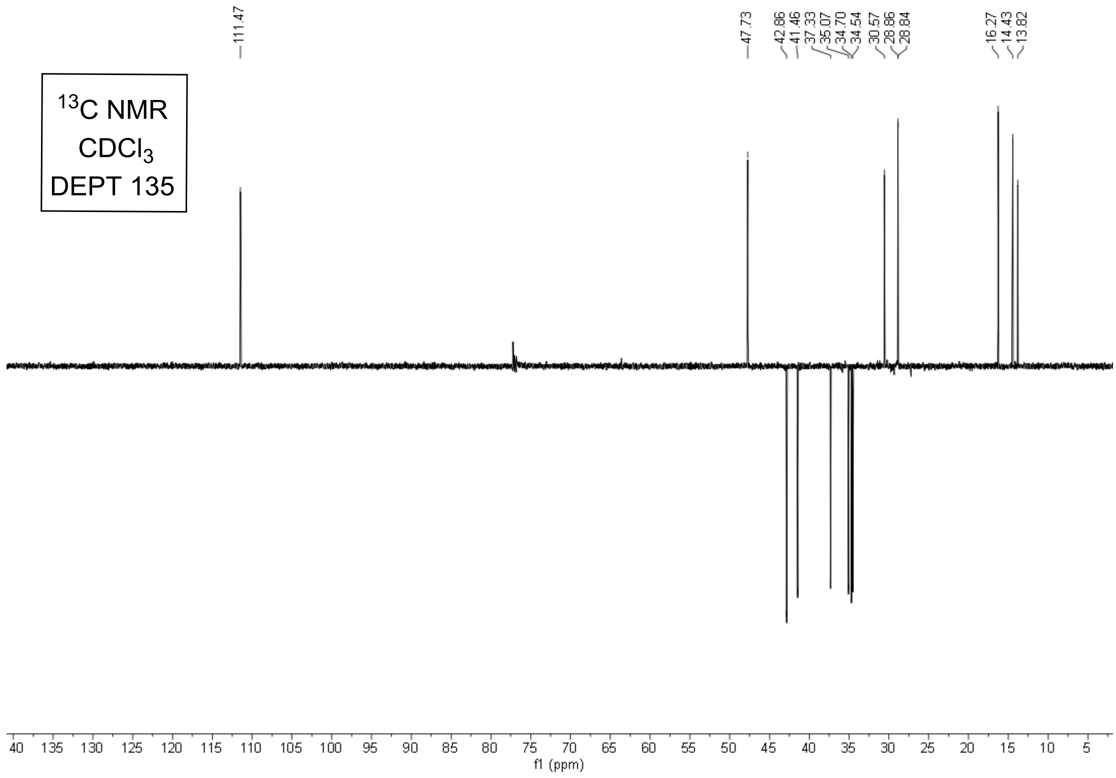

^{13}C NMR
$CDCl_3$
DEPT 135
111.47
47.73
42.86
41.46
37.33
35.07
34.70
34.54
30.57
28.86
28.84
16.27
14.43
13.82
f1 (ppm)

¹H NMR
$CDCl_3$

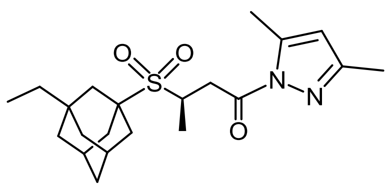

3i

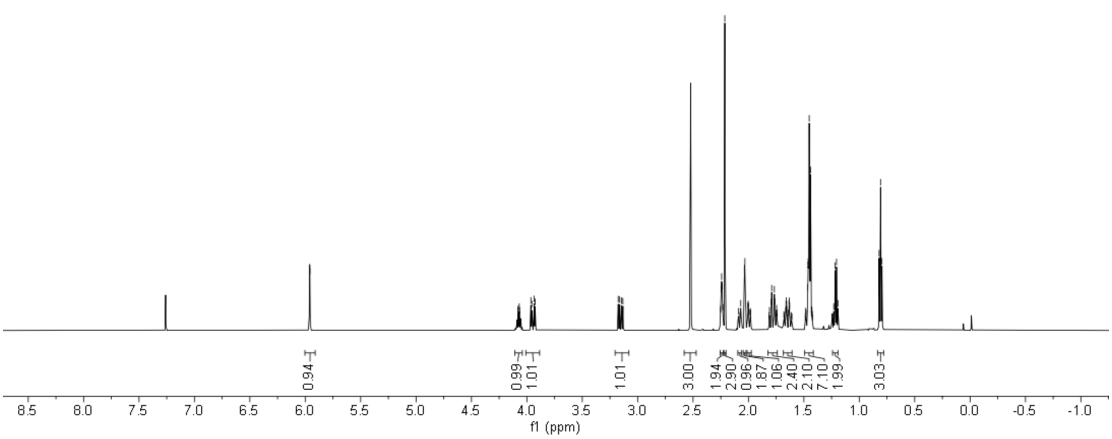

¹³C NMR
$CDCl_3$

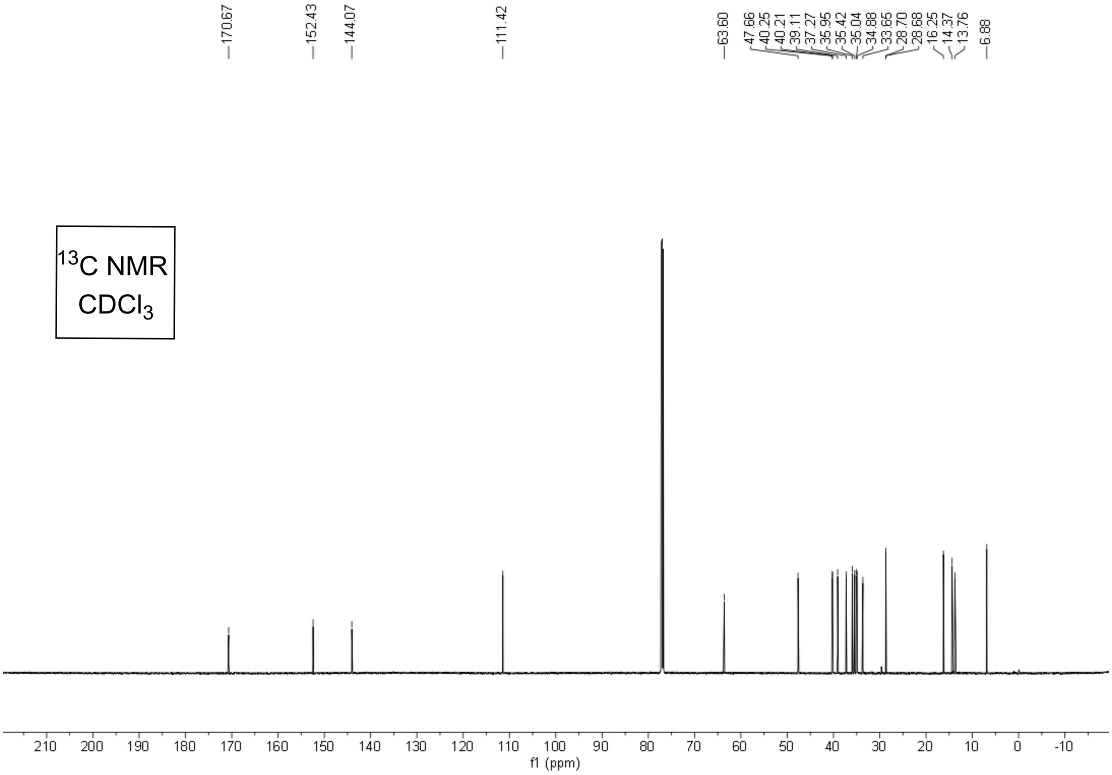

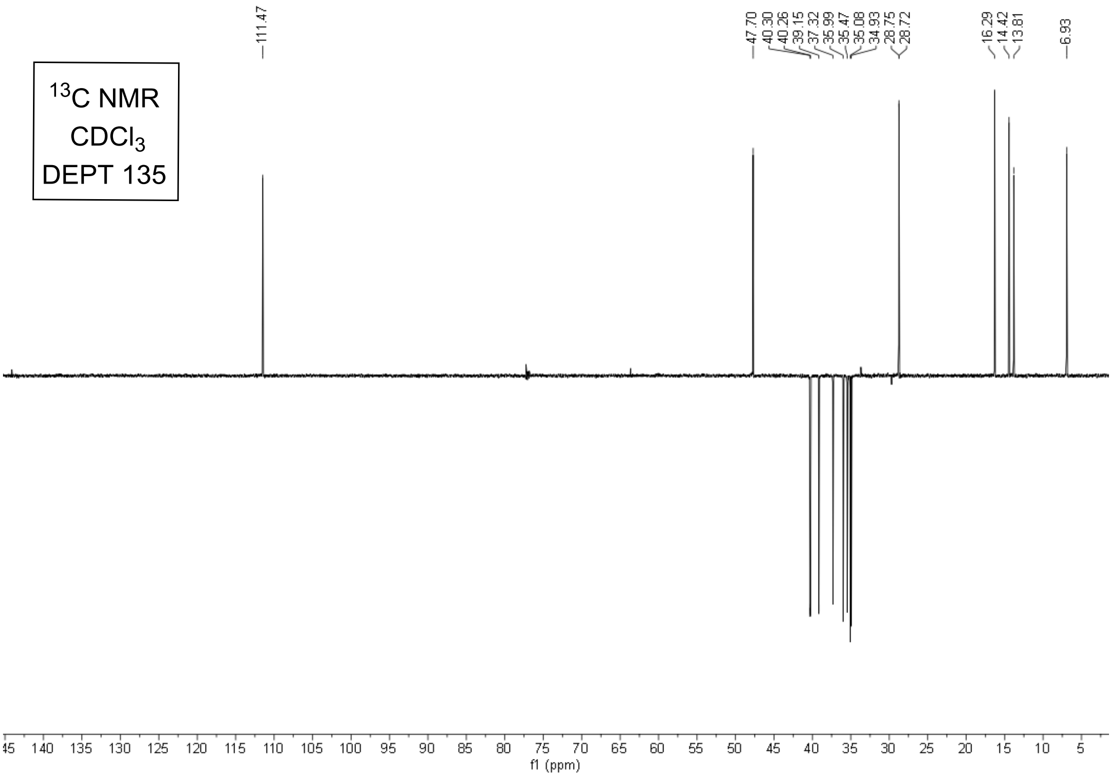

^{13}C NMR
CDCl$_3$
DEPT 135
111.47
47.70
40.30
40.26
39.15
37.32
35.99
35.47
35.08
34.93
28.75
28.72
16.29
14.42
13.81
6.93
f1 (ppm)

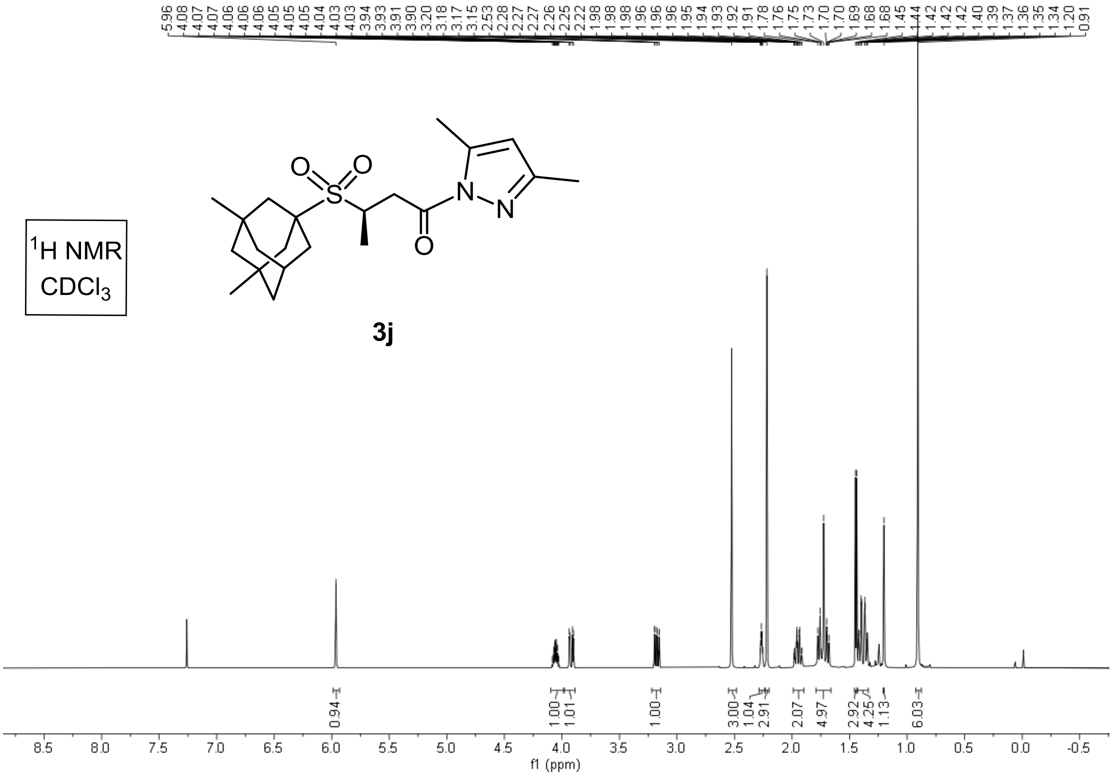
5.96 4.08 4.07 4.07 4.06 4.06 4.06 4.05 4.05 4.05 4.04 4.03 4.03 3.94 3.93 3.91 3.90 3.20 3.18 3.17 3.15 2.53 2.28 2.27 2.27 2.26 2.25 2.25 2.22 1.98 1.98 1.98 1.96 1.96 1.96 1.95 1.94 1.93 1.92 1.91 1.78 1.76 1.75 1.73 1.70 1.70 1.69 1.68 1.68 1.45 1.44 1.42 1.42 1.42 1.40 1.39 1.37 1.36 1.35 1.34 1.20 0.91
^{1}H NMR
$CDCl_3$
3j
0.94 1.00 1.01 1.00 3.00 1.04 2.91 2.07 4.97 2.92 4.25 1.13 6.03
8.5 8.0 7.5 7.0 6.5 6.0 5.5 5.0 4.5 4.0 3.5 3.0 2.5 2.0 1.5 1.0 0.5 0.0 -0.5
f1 (ppm)

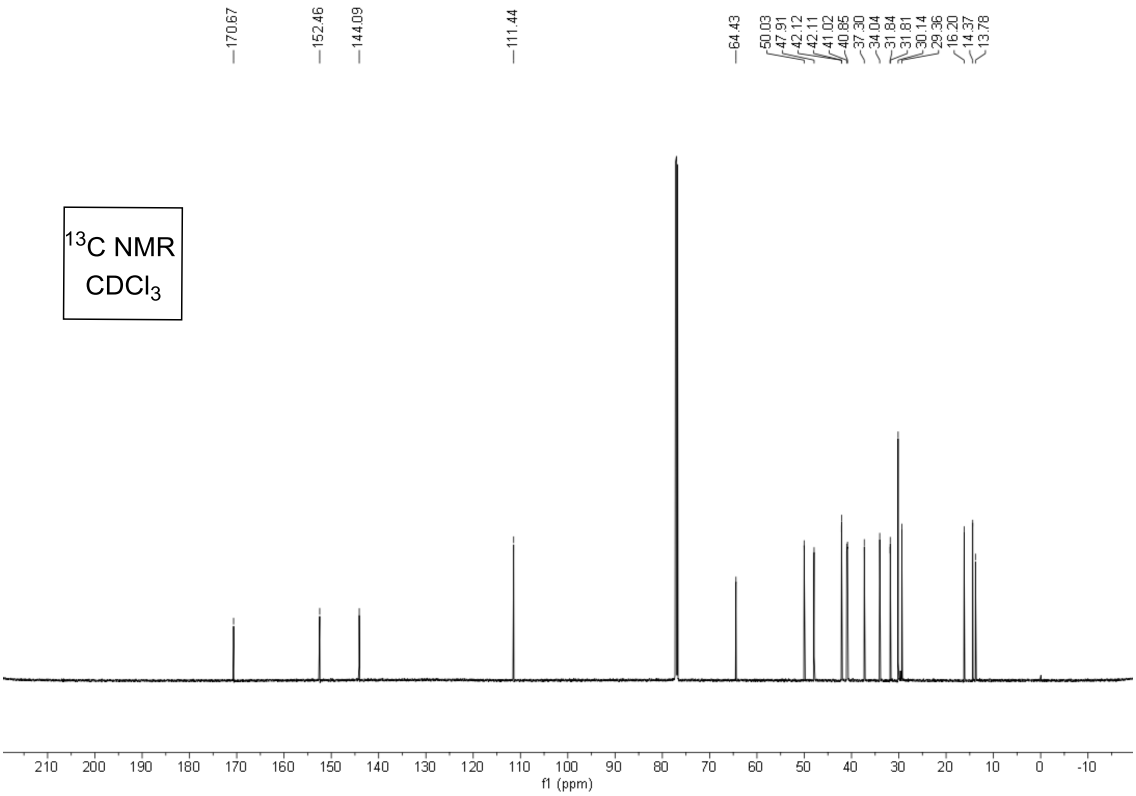
170.67 152.46 144.09 111.44 64.43 50.03 47.91 42.12 42.11 41.02 40.85 37.30 34.04 31.84 31.81 30.14 29.36 16.20 14.37 13.78
^{13}C NMR
$CDCl_3$
210 200 190 180 170 160 150 140 130 120 110 100 90 80 70 60 50 40 30 20 10 0 -10
f1 (ppm)

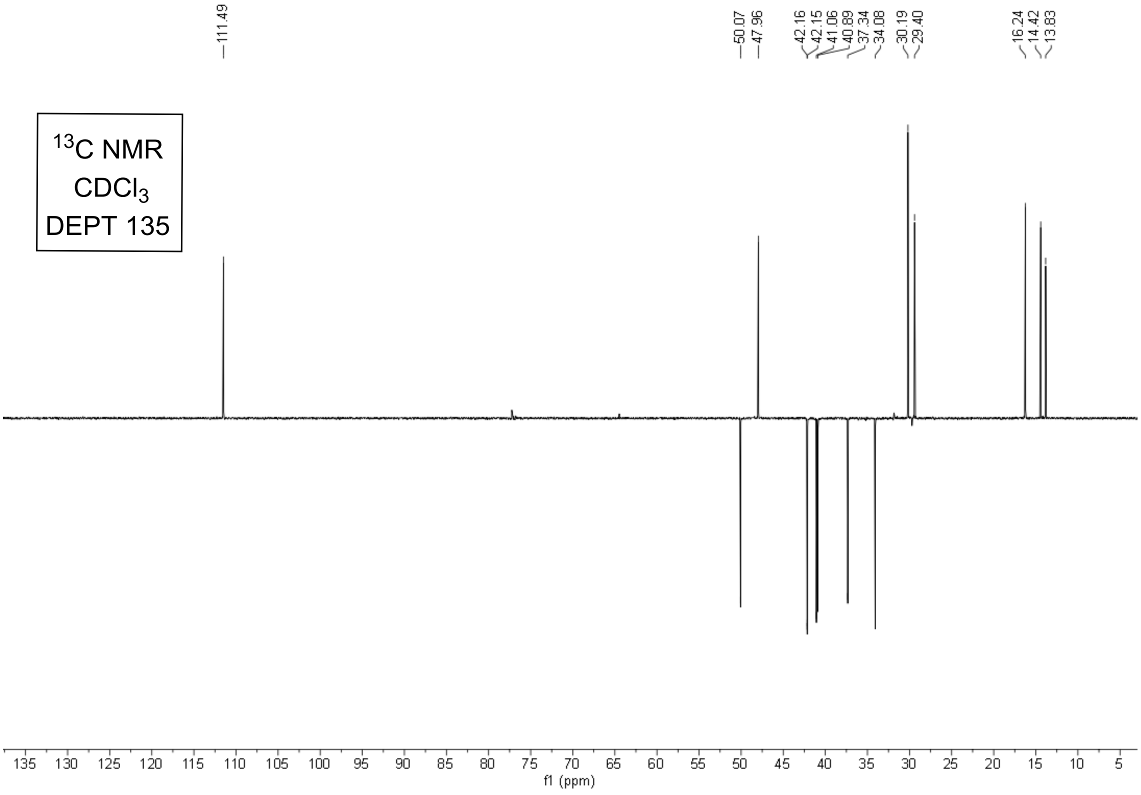
111.49
50.07
47.96
42.16
42.15
41.06
40.89
37.34
34.08
30.19
29.40
16.24
14.42
13.83
13C NMR
CDCl3
DEPT 135
135 130 125 120 115 110 105 100 95 90 85 80 75 70 65 60 55 50 45 40 35 30 25 20 15 10 5
f1 (ppm)

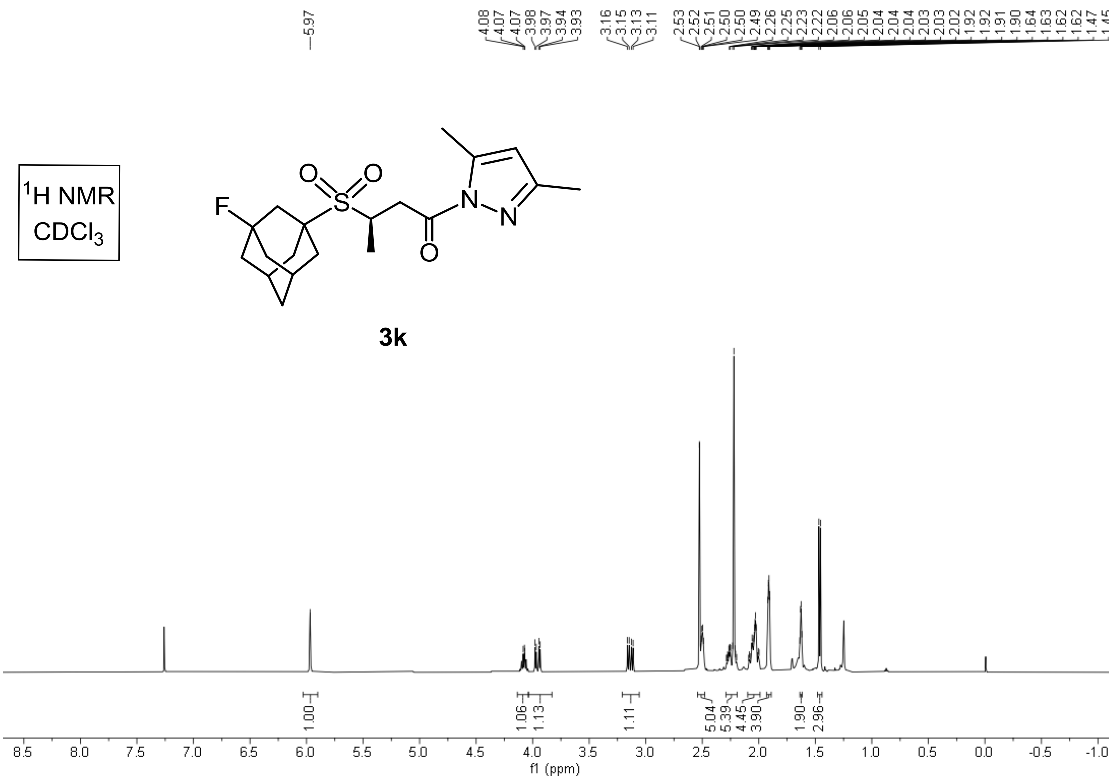

1H NMR
CDCl3
3k

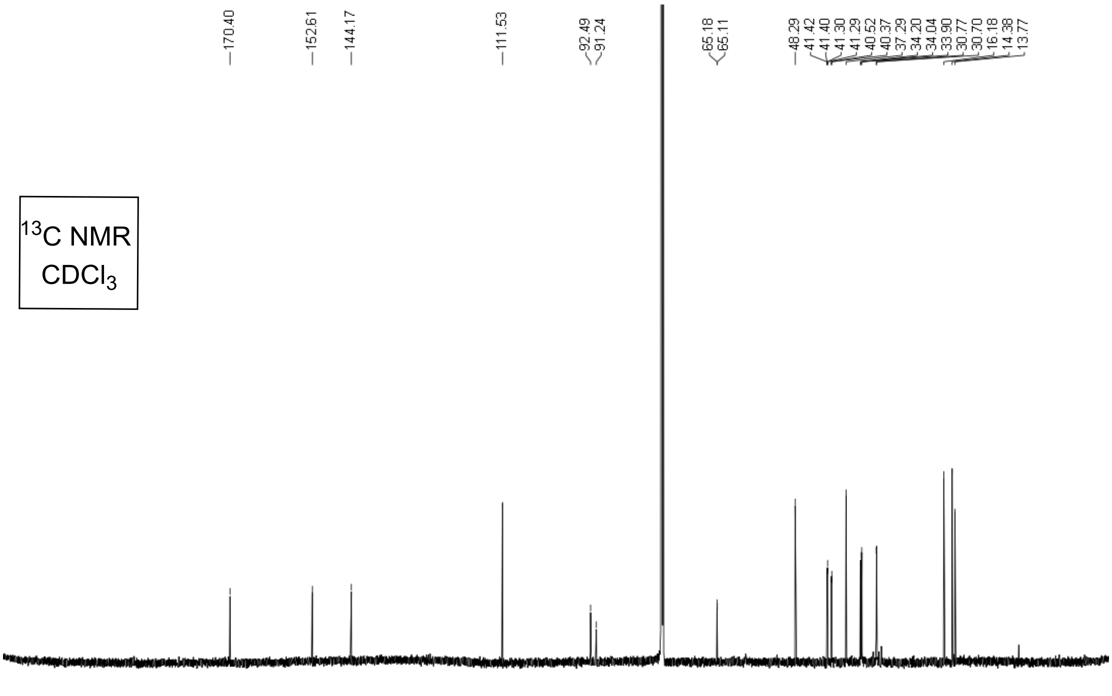

13C NMR
CDCl3

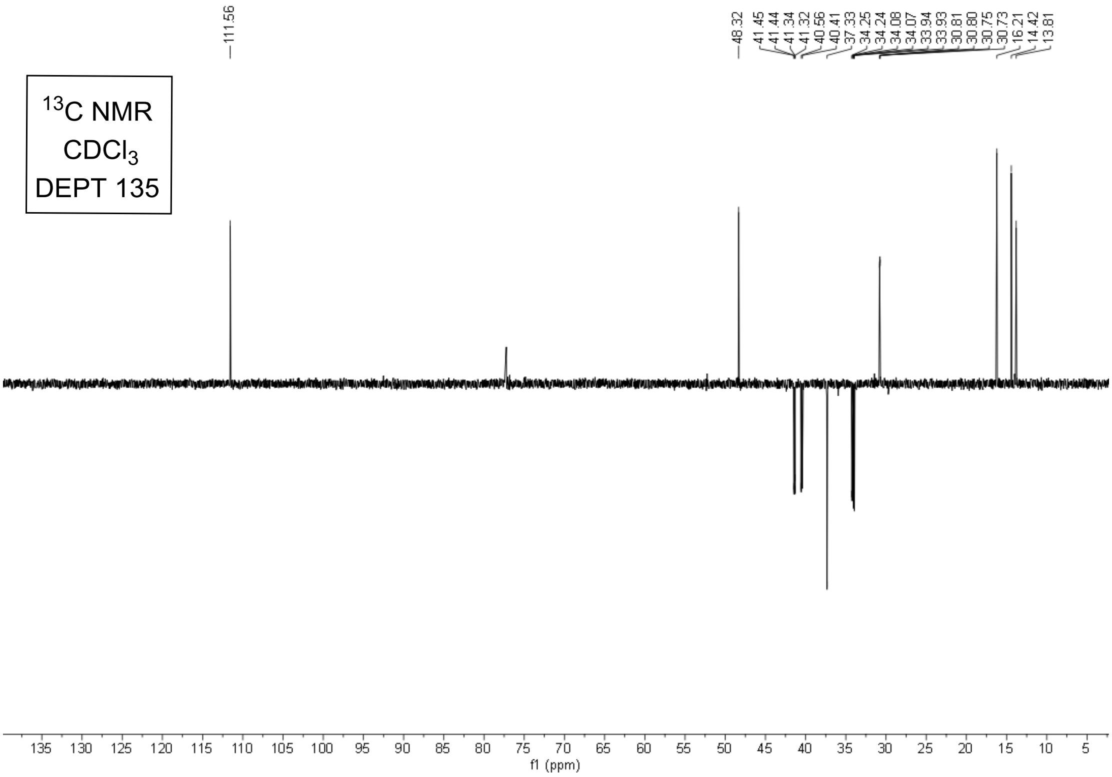

^{13}C NMR
CDCl$_3$
DEPT 135
111.56
48.32
41.45
41.44
41.34
41.32
40.56
40.41
37.33
34.25
34.24
34.08
34.07
33.94
33.93
30.81
30.80
30.75
30.73
16.21
14.42
13.81
135 130 125 120 115 110 105 100 95 90 85 80 75 70 65 60 55 50 45 40 35 30 25 20 15 10 5
f1 (ppm)

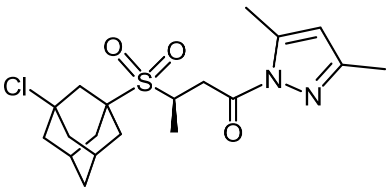

3l

^{1}H NMR
$CDCl_3$

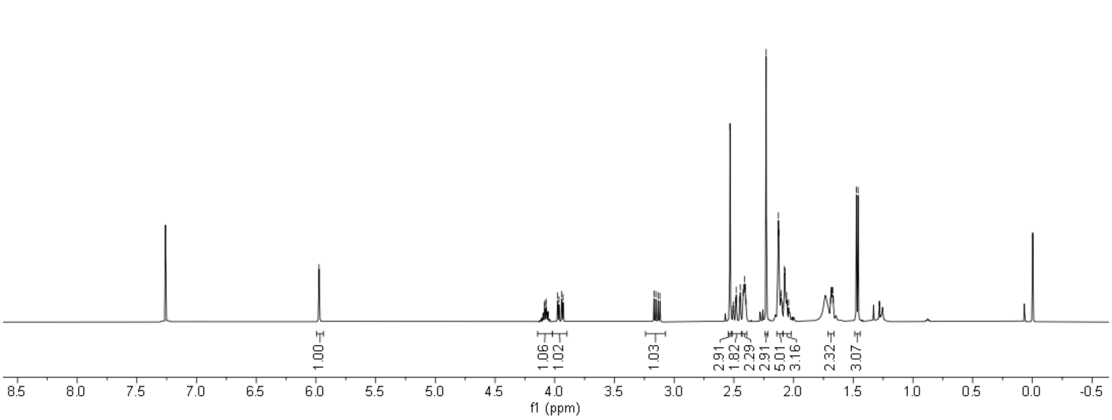

^{13}C NMR
$CDCl_3$

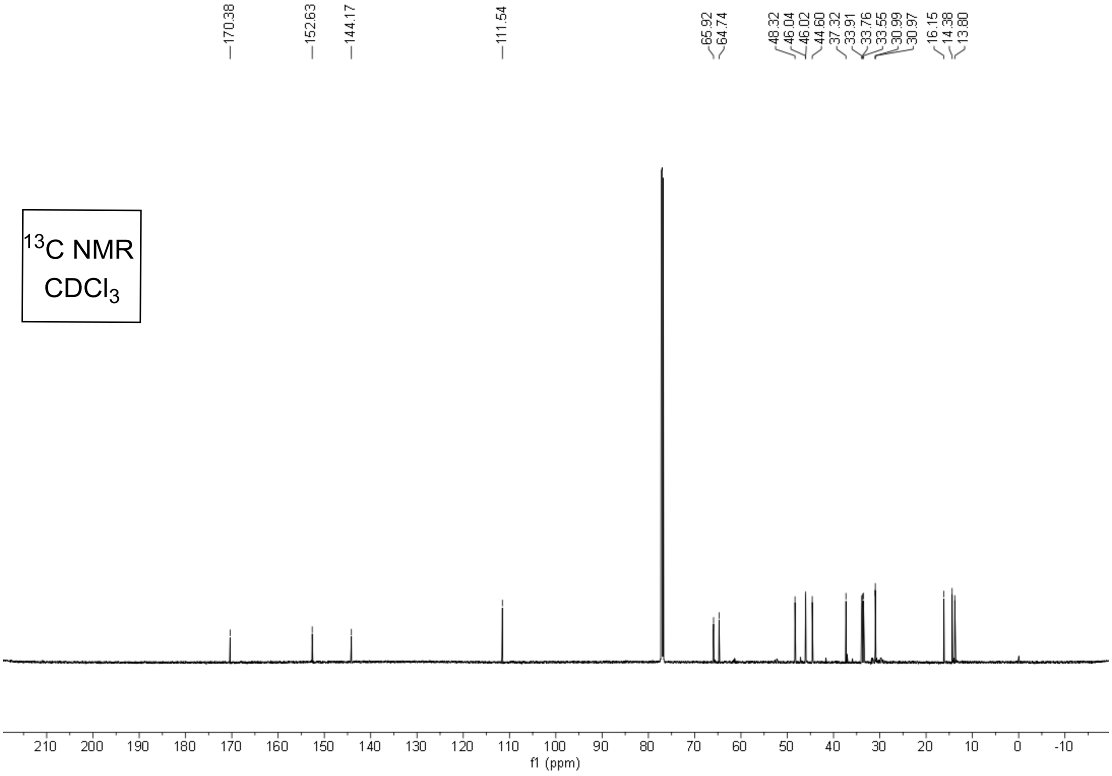

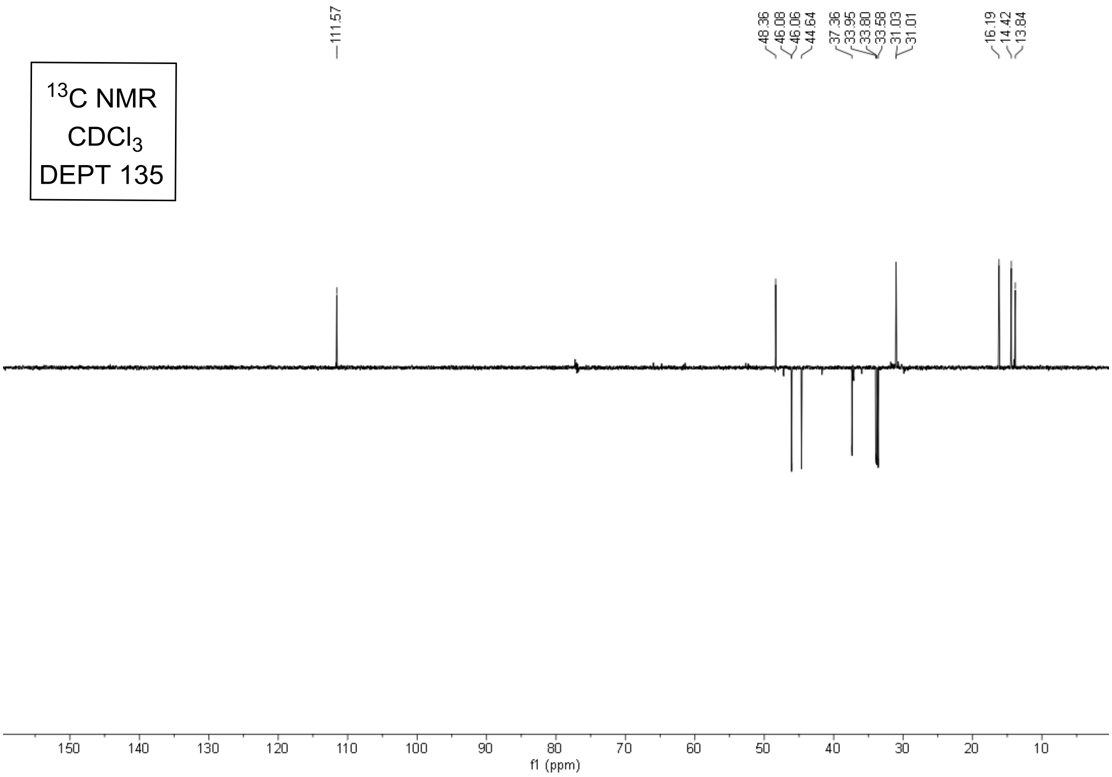

^{13}C NMR
CDCl$_3$
DEPT 135
111.57
48.36
46.08
46.06
44.64
37.36
33.95
33.80
33.58
31.03
31.01
16.19
14.42
13.84
150 140 130 120 110 100 90 80 70 60 50 40 30 20 10
f1 (ppm)

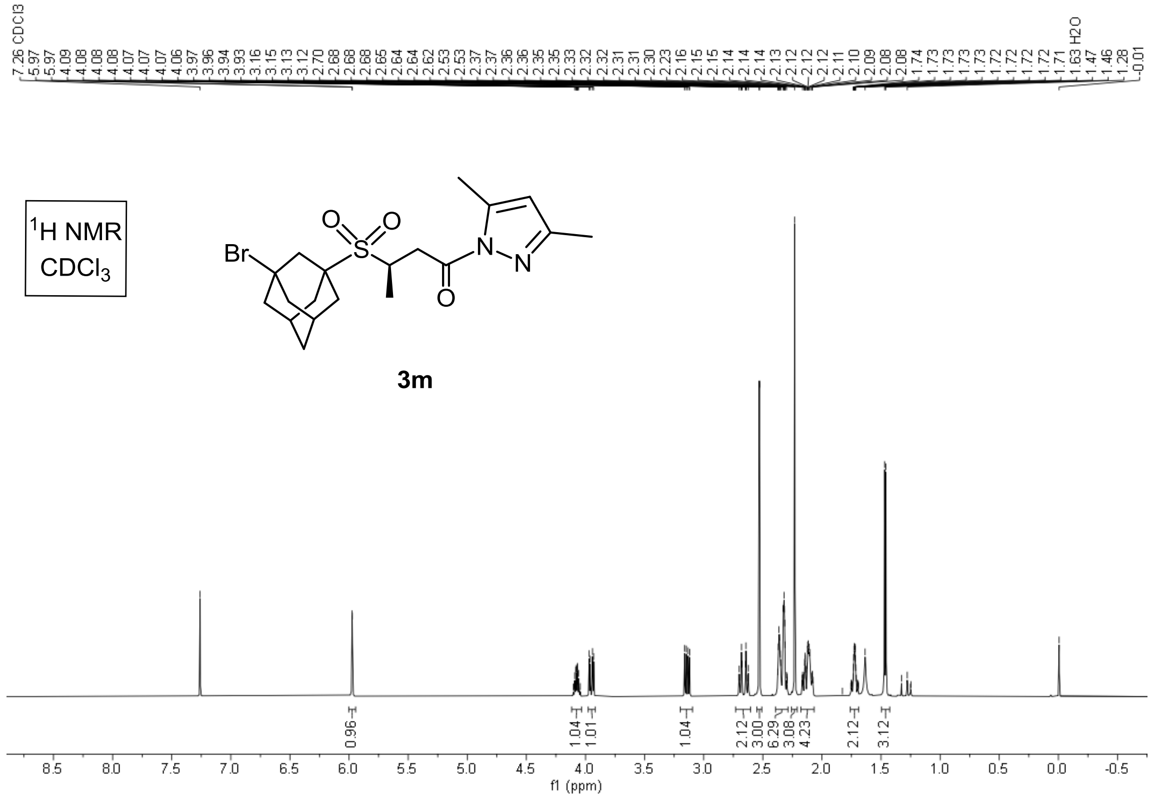
7.26 CDCl3
5.97
5.97
4.09
4.08
4.08
4.08
4.07
4.07
4.07
4.06
3.97
3.96
3.94
3.93
3.16
3.15
3.13
3.12
2.70
2.68
2.68
2.65
2.64
2.62
2.53
2.37
2.37
2.36
2.35
2.35
2.33
2.32
2.32
2.31
2.31
2.30
2.23
2.16
2.15
2.15
2.14
2.14
2.13
2.12
2.12
2.12
2.11
2.10
2.09
2.08
2.08
1.74
1.73
1.73
1.73
1.73
1.72
1.72
1.72
1.71
1.63 H2O
1.47
1.46
1.28
-0.01
^{1}H NMR
$CDCl_3$
Br
O
O
S
N
N
O
3m
0.96
1.04
1.01
1.04
2.12
3.00
6.29
3.08
4.23
2.12
3.12
8.5
8.0
7.5
7.0
6.5
6.0
5.5
5.0
4.5
4.0
3.5
3.0
2.5
2.0
1.5
1.0
0.5
0.0
-0.5
f1 (ppm)

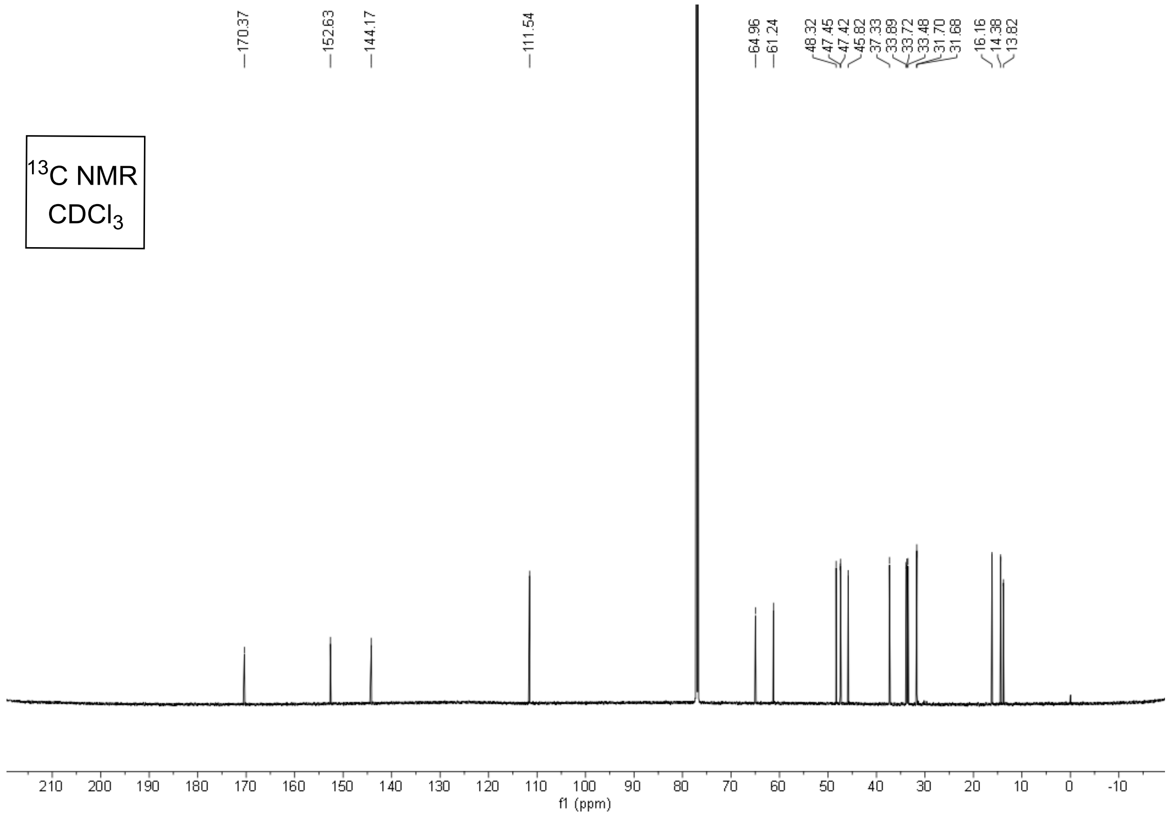
170.37
152.63
144.17
111.54
64.96
61.24
48.32
47.45
47.42
45.82
37.33
33.89
33.72
33.48
31.70
31.68
16.16
14.38
13.82
^{13}C NMR
$CDCl_3$
210
200
190
180
170
160
150
140
130
120
110
100
90
80
70
60
50
40
30
20
10
0
-10
f1 (ppm)

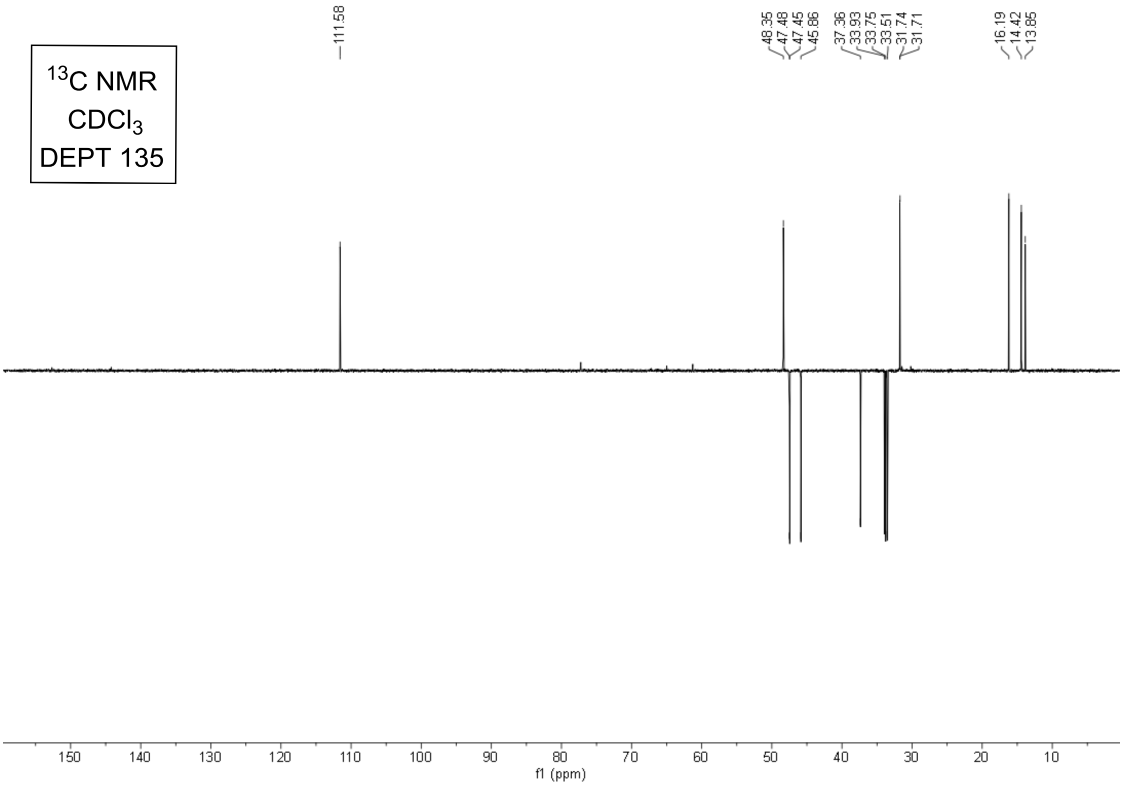

^{13}C NMR
$CDCl_3$
DEPT 135
111.58
48.35
47.48
47.45
45.86
37.36
33.93
33.75
33.51
31.74
31.71
16.19
14.42
13.85
150
140
130
120
110
100
90
80
70
60
50
40
30
20
10
f1 (ppm)

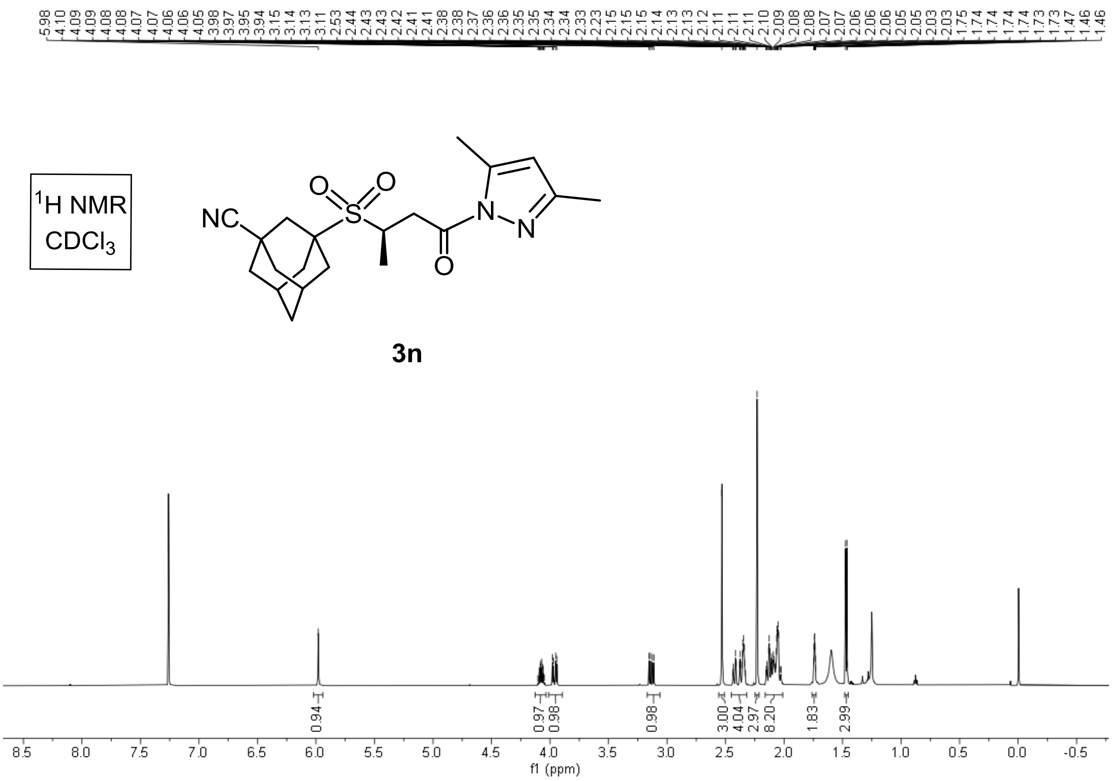
1H NMR
CDCl3
NC
O
S
O
N
N
O
3n
f1 (ppm)

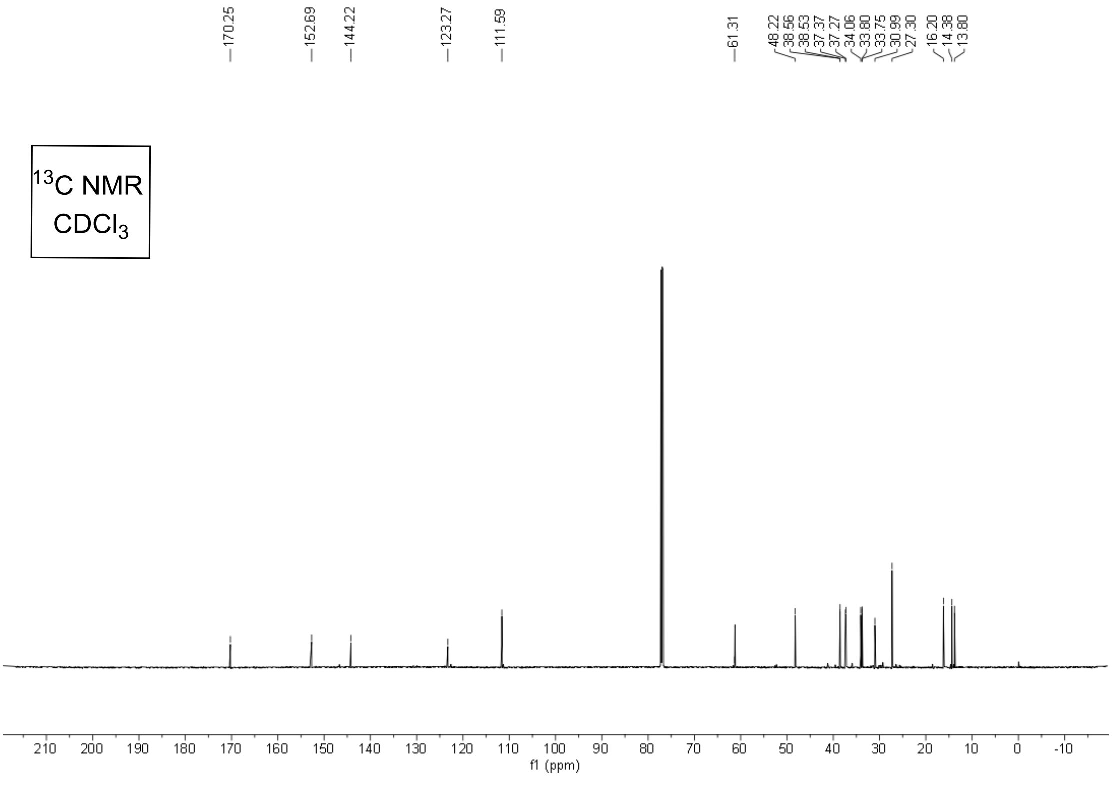
13C NMR
CDCl3
170.25
152.69
144.22
123.27
111.59
61.31
48.22
38.56
38.53
37.37
37.27
34.06
33.80
33.75
30.99
27.30
16.20
14.38
13.80
f1 (ppm)

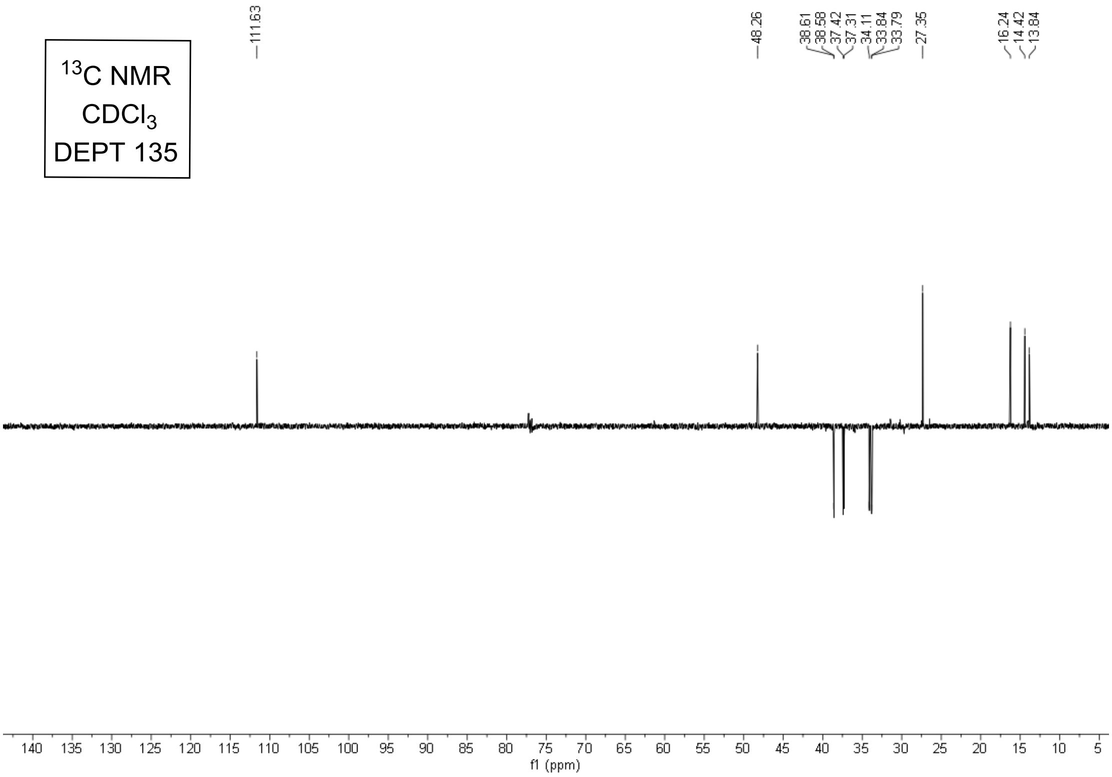
13C NMR
CDCl3
DEPT 135
111.63
48.26
38.61
38.58
37.42
37.31
34.11
33.84
33.79
27.35
16.24
14.42
13.84
f1 (ppm)

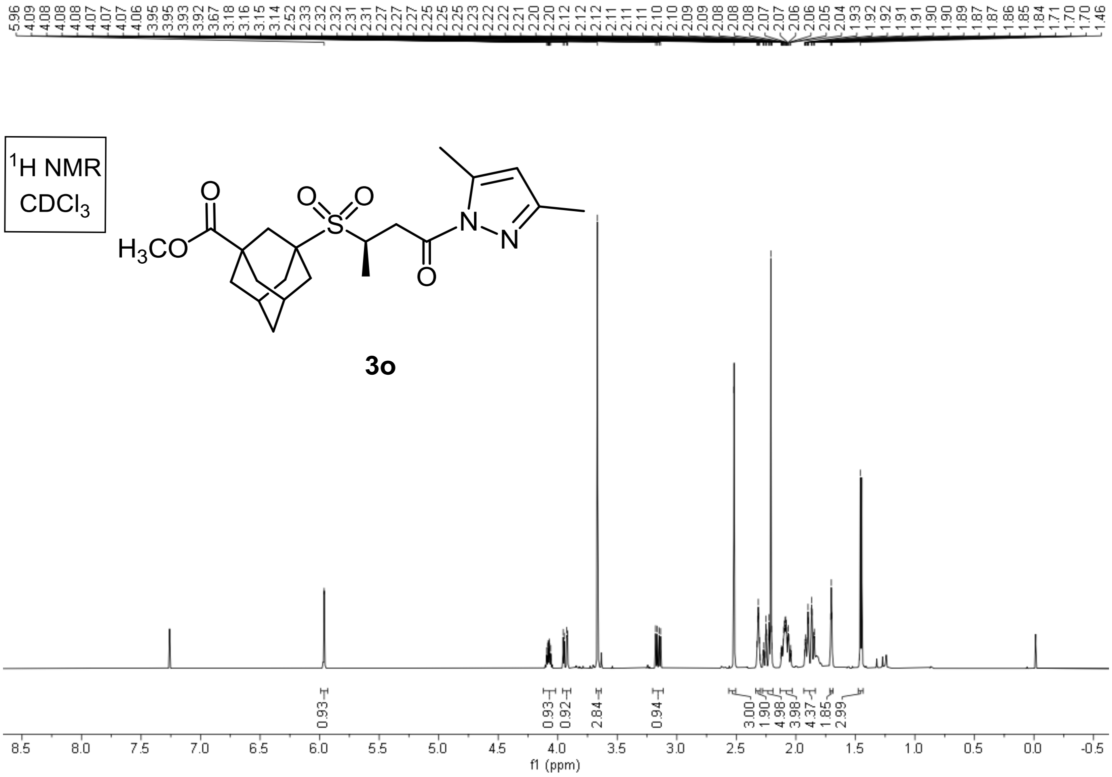
5.96
4.09
4.08
4.08
4.08
4.07
4.07
4.07
4.06
3.95
3.95
3.93
3.92
3.67
3.18
3.16
3.15
3.14
2.52
2.33
2.32
2.32
2.31
2.31
2.27
2.27
2.25
2.25
2.25
2.23
2.22
2.22
2.21
2.20
2.20
2.12
2.12
2.12
2.11
2.11
2.11
2.10
2.10
2.09
2.09
2.08
2.08
2.08
2.07
2.07
2.06
2.06
2.05
2.04
1.93
1.92
1.92
1.91
1.91
1.90
1.90
1.89
1.87
1.87
1.86
1.85
1.84
1.71
1.70
1.46
1H NMR
CDCl3
H3CO
O
O
S
O
O
N
N
3o
0.93
0.93
0.92
2.84
0.94
3.00
1.90
4.98
3.98
4.37
1.85
2.99
8.5
8.0
7.5
7.0
6.5
6.0
5.5
5.0
4.5
4.0
3.5
3.0
2.5
2.0
1.5
1.0
0.5
0.0
-0.5
f1 (ppm)

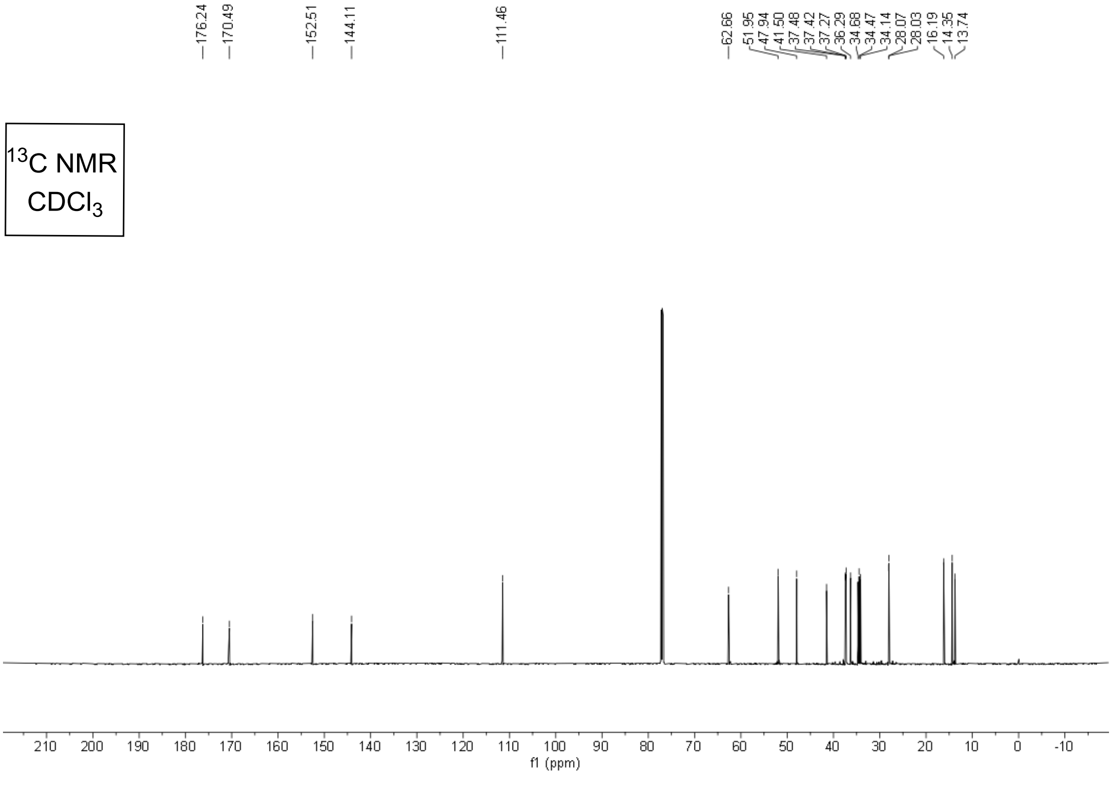
176.24
170.49
152.51
144.11
111.46
62.66
51.95
47.94
41.50
37.48
37.42
37.27
36.29
34.68
34.47
34.14
28.07
28.03
16.19
14.35
13.74
13C NMR
CDCl3
210
200
190
180
170
160
150
140
130
120
110
100
90
80
70
60
50
40
30
20
10
0
-10
f1 (ppm)

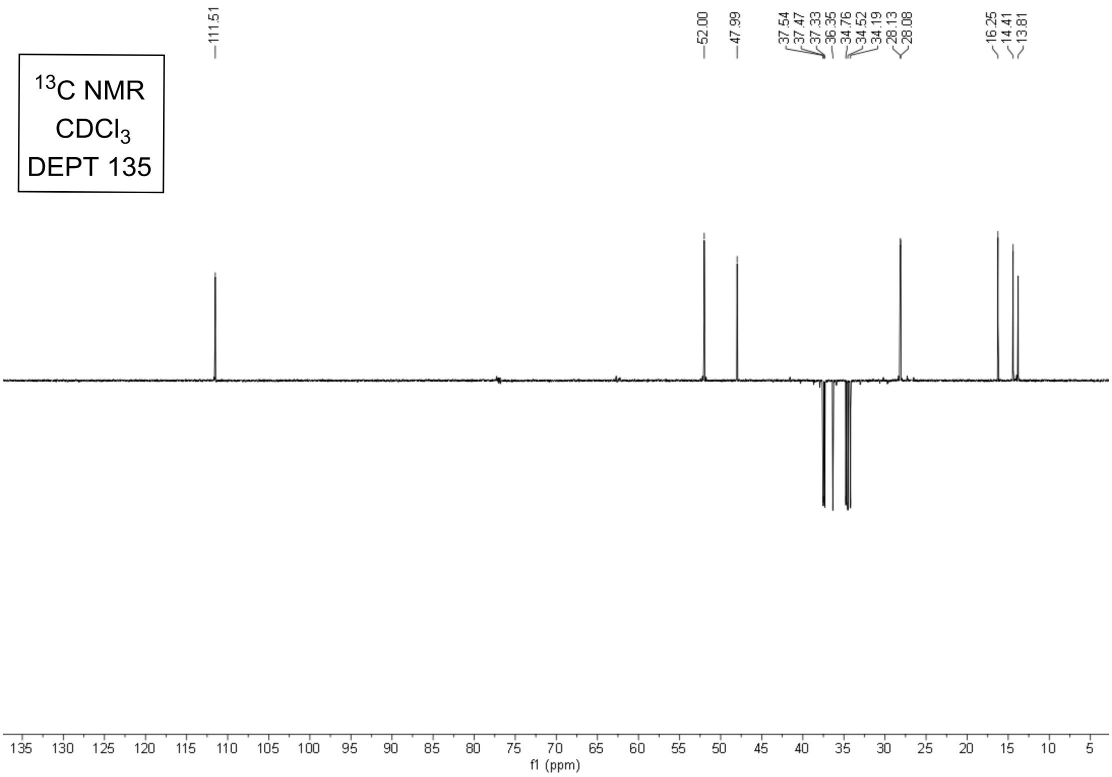
^{13}C NMR
$CDCl_3$
DEPT 135
111.51
52.00
47.99
37.54
37.47
37.33
36.35
34.76
34.52
34.19
28.13
28.08
16.25
14.41
13.81
f1 (ppm)

^{1}H NMR
$CDCl_3$

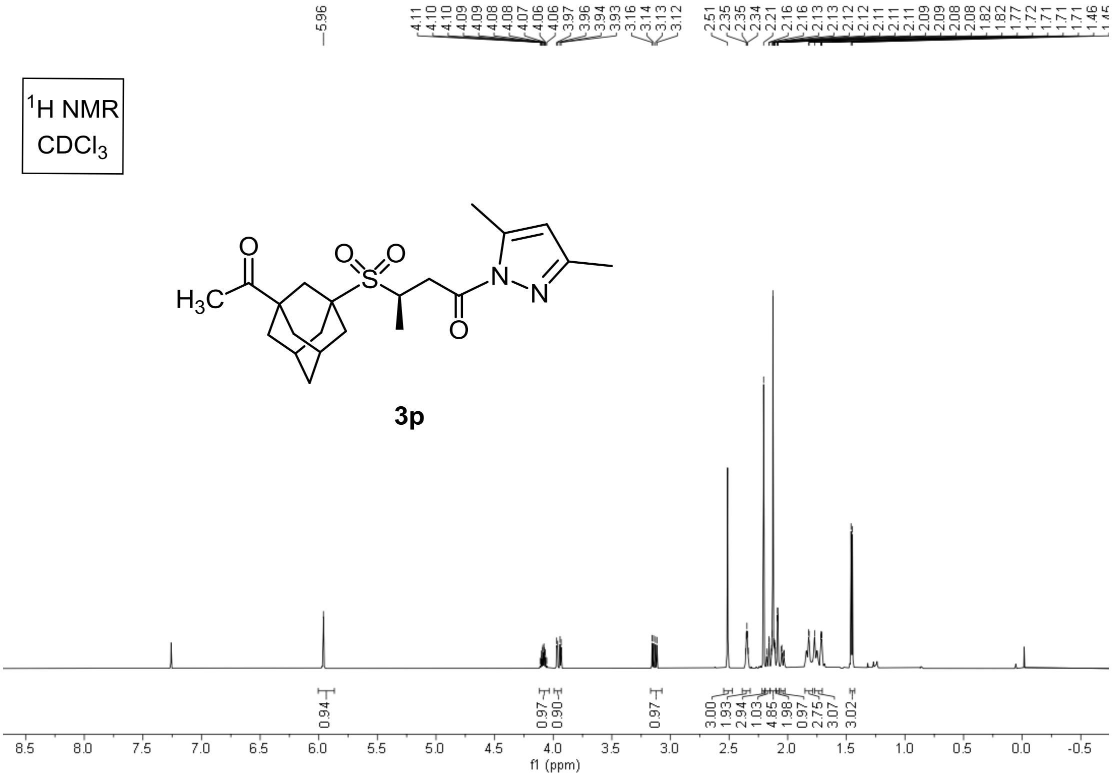

3p

^{13}C NMR
$CDCl_3$

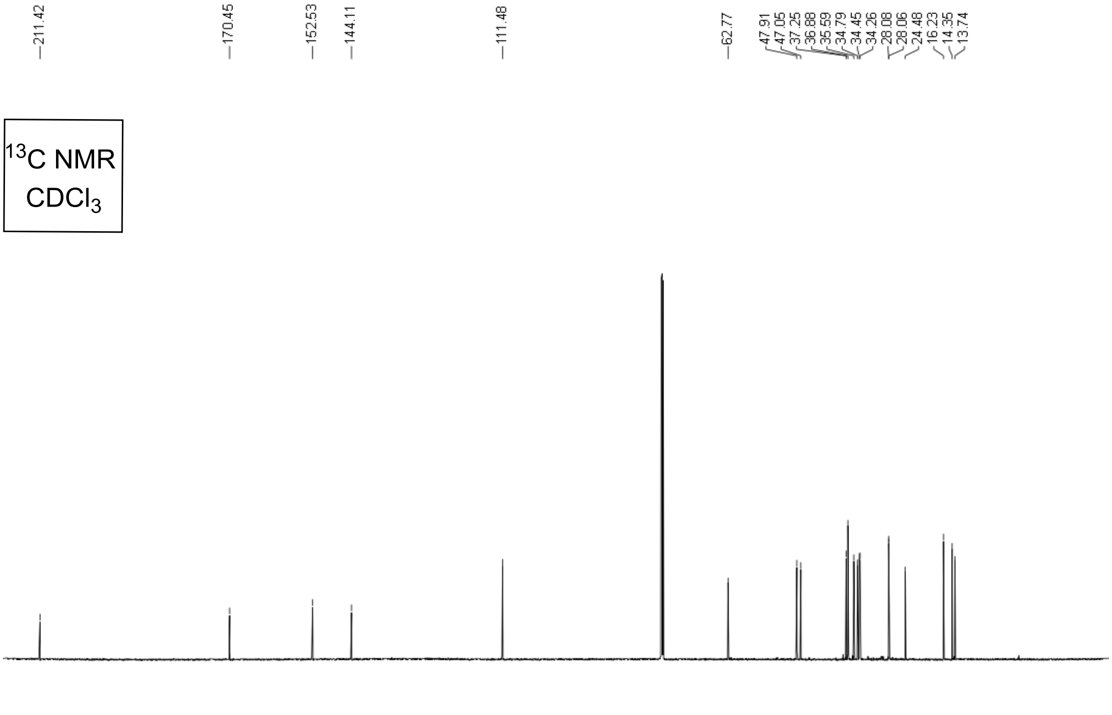

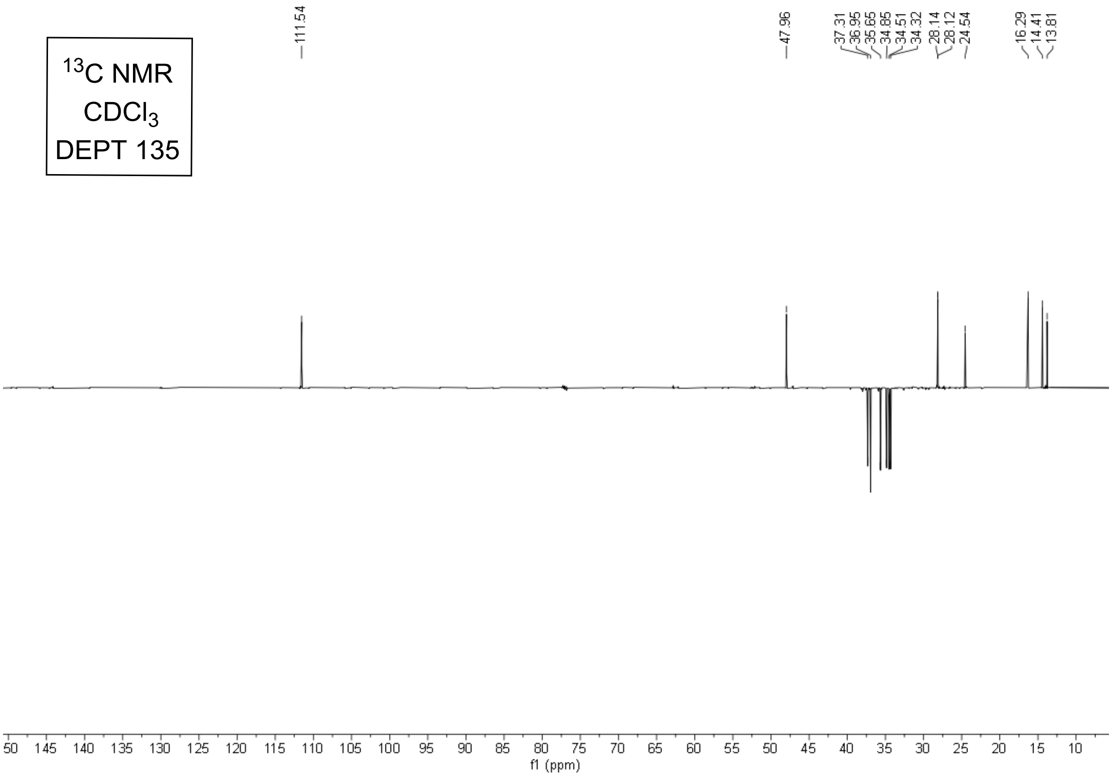

^{13}C NMR
$CDCl_3$
DEPT 135
111.54
47.96
37.31
36.95
35.65
34.85
34.51
34.32
28.14
28.12
24.54
16.29
14.41
13.81
150 145 140 135 130 125 120 115 110 105 100 95 90 85 80 75 70 65 60 55 50 45 40 35 30 25 20 15 10
f1 (ppm)

^{1}H NMR
$CDCl_3$

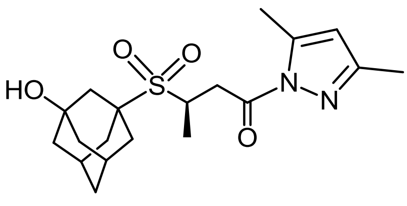

3q

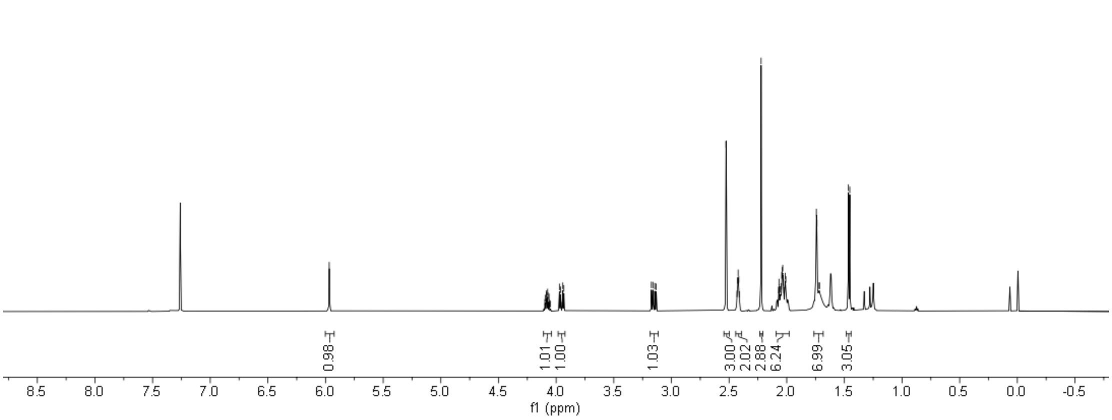

^{13}C NMR
$CDCl_3$

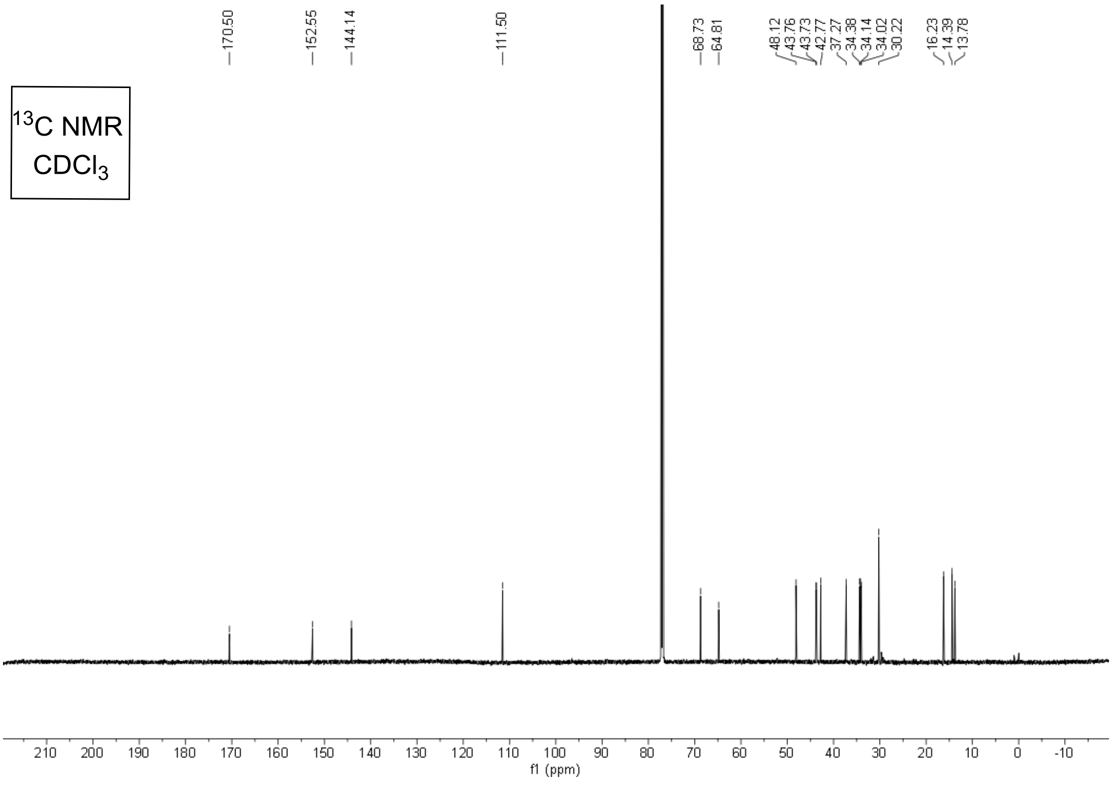

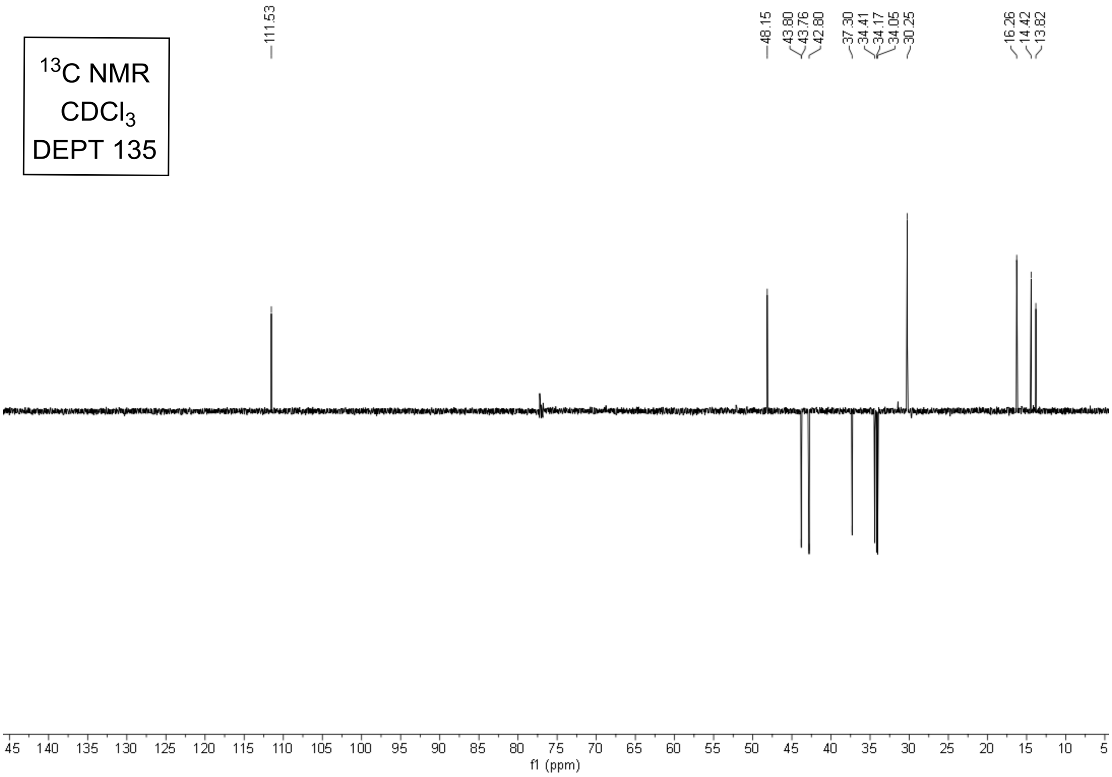
^{13}C NMR
$CDCl_3$
DEPT 135
111.53
48.15
43.80
43.76
42.80
37.30
34.41
34.17
34.05
30.25
16.26
14.42
13.82
f1 (ppm)

^{1}H NMR
$CDCl_3$

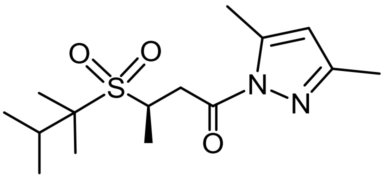

3r

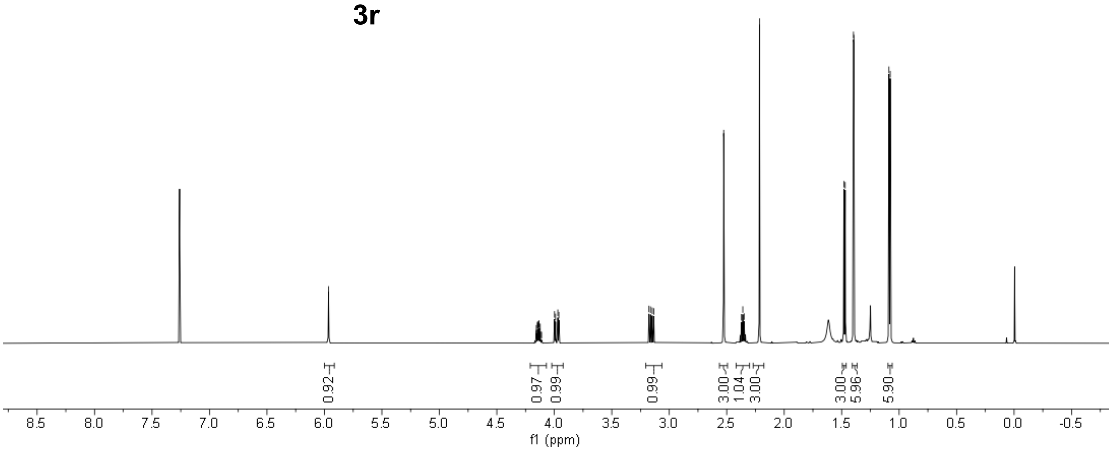

^{13}C NMR
$CDCl_3$

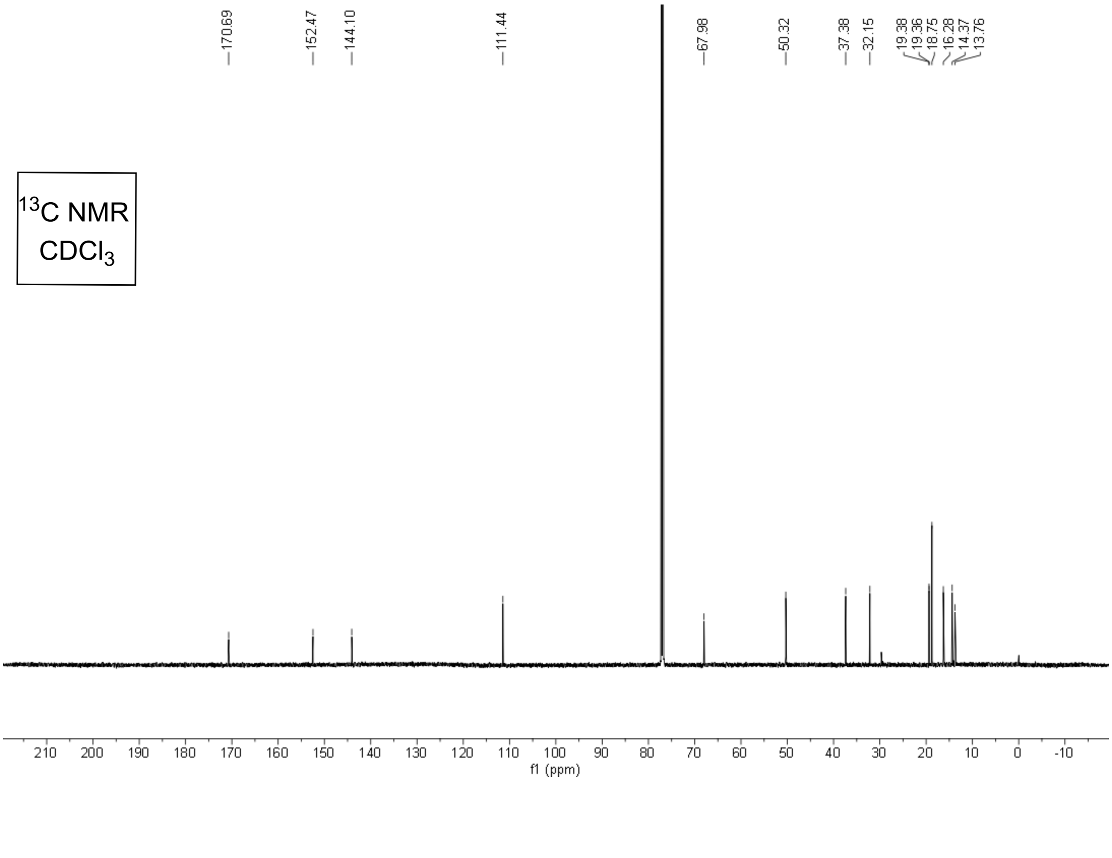

^{1}H NMR
$CDCl_3$

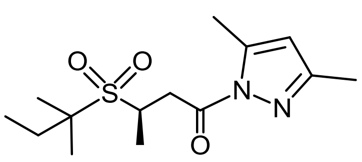

3s

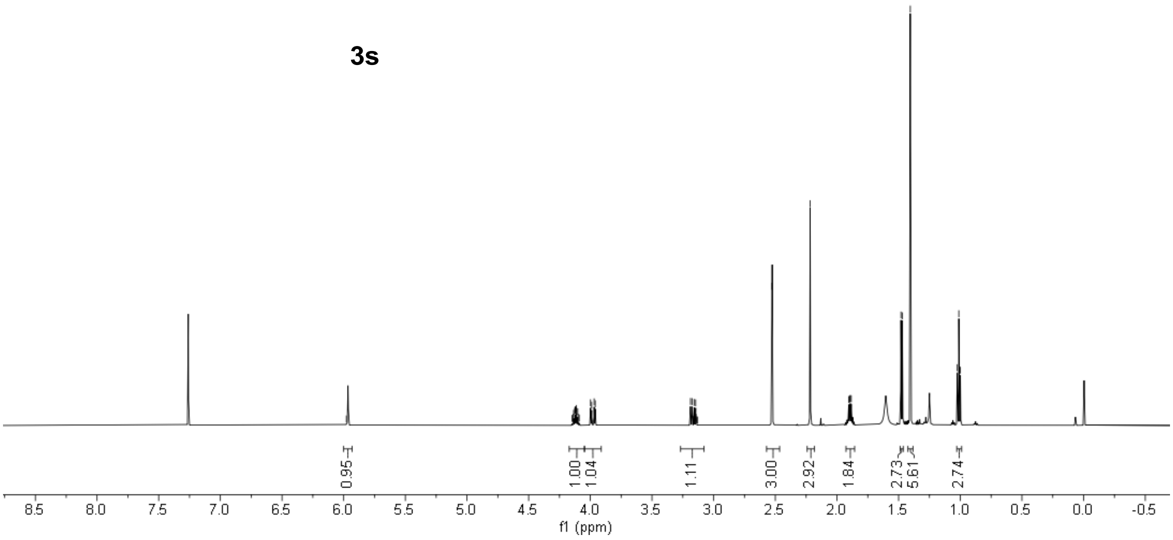

^{13}C NMR
$CDCl_3$

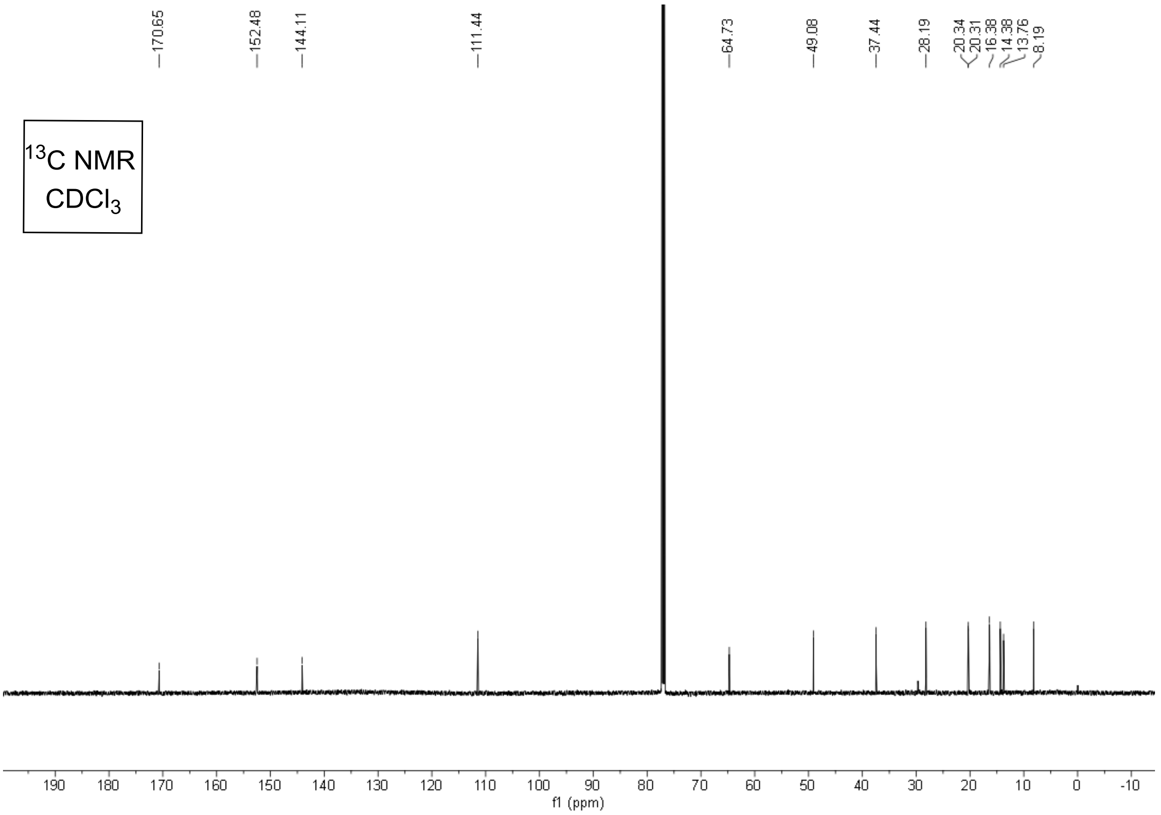

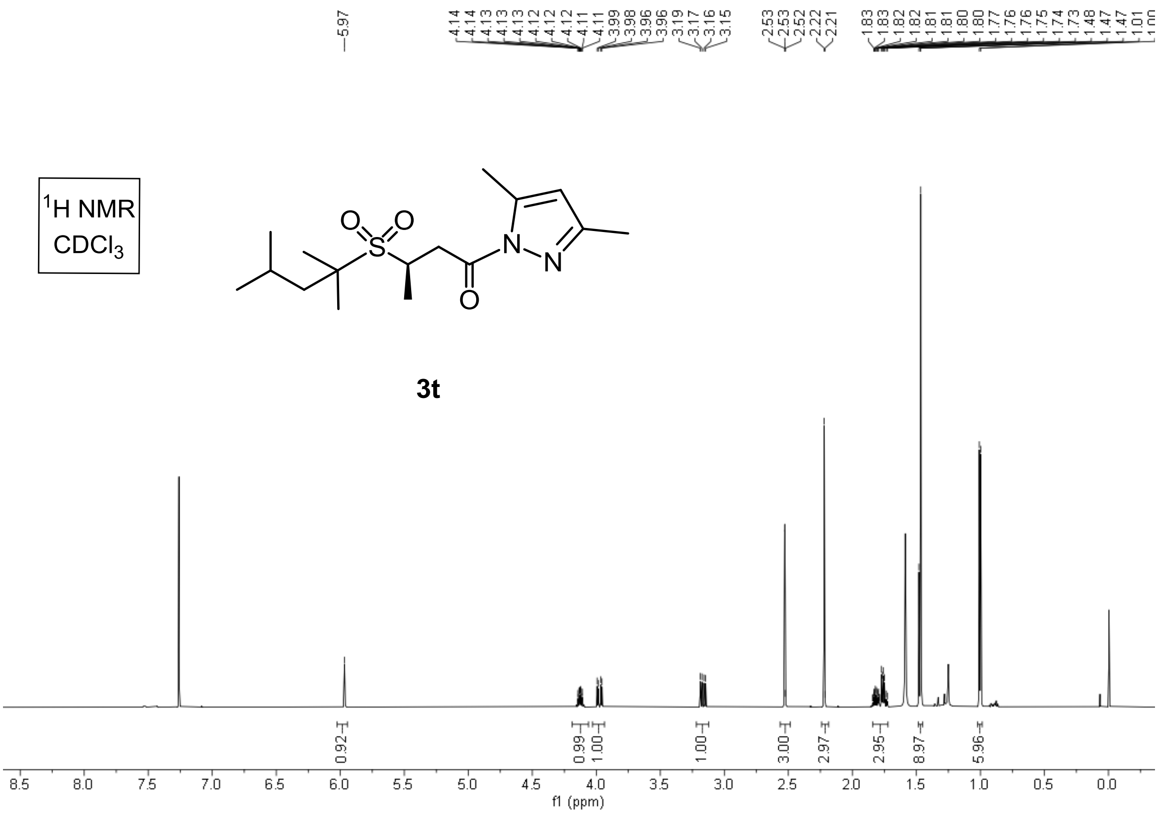
1H NMR
CDCl3
3t
5.97
4.14
4.14
4.13
4.13
4.12
4.12
4.11
4.11
3.99
3.98
3.96
3.96
3.19
3.17
3.16
3.15
2.53
2.53
2.52
2.22
2.21
1.83
1.83
1.82
1.82
1.81
1.81
1.80
1.80
1.77
1.76
1.75
1.74
1.73
1.48
1.47
1.01
1.00
0.92
0.99
1.00
1.00
3.00
2.97
2.95
8.97
5.96
f1 (ppm)

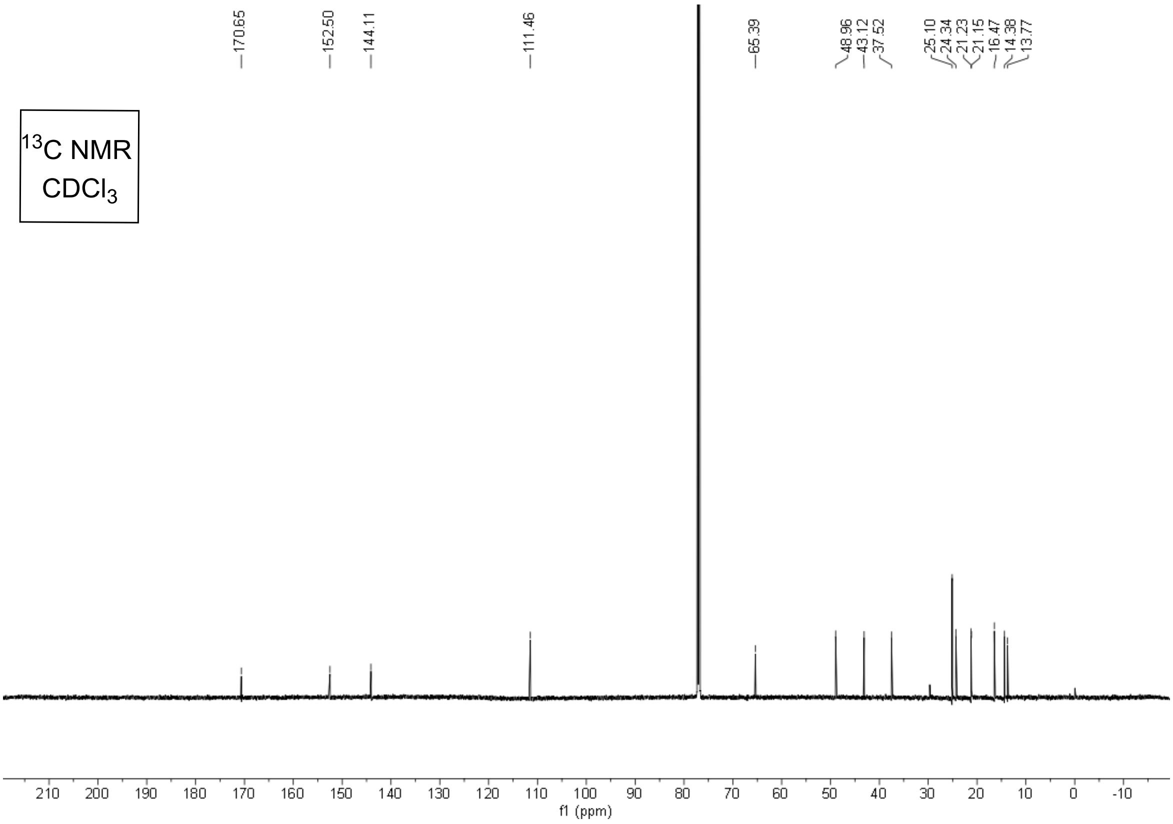
13C NMR
CDCl3
170.65
152.50
144.11
111.46
65.39
48.96
43.12
37.52
25.10
24.34
21.23
21.15
16.47
14.38
13.77
f1 (ppm)

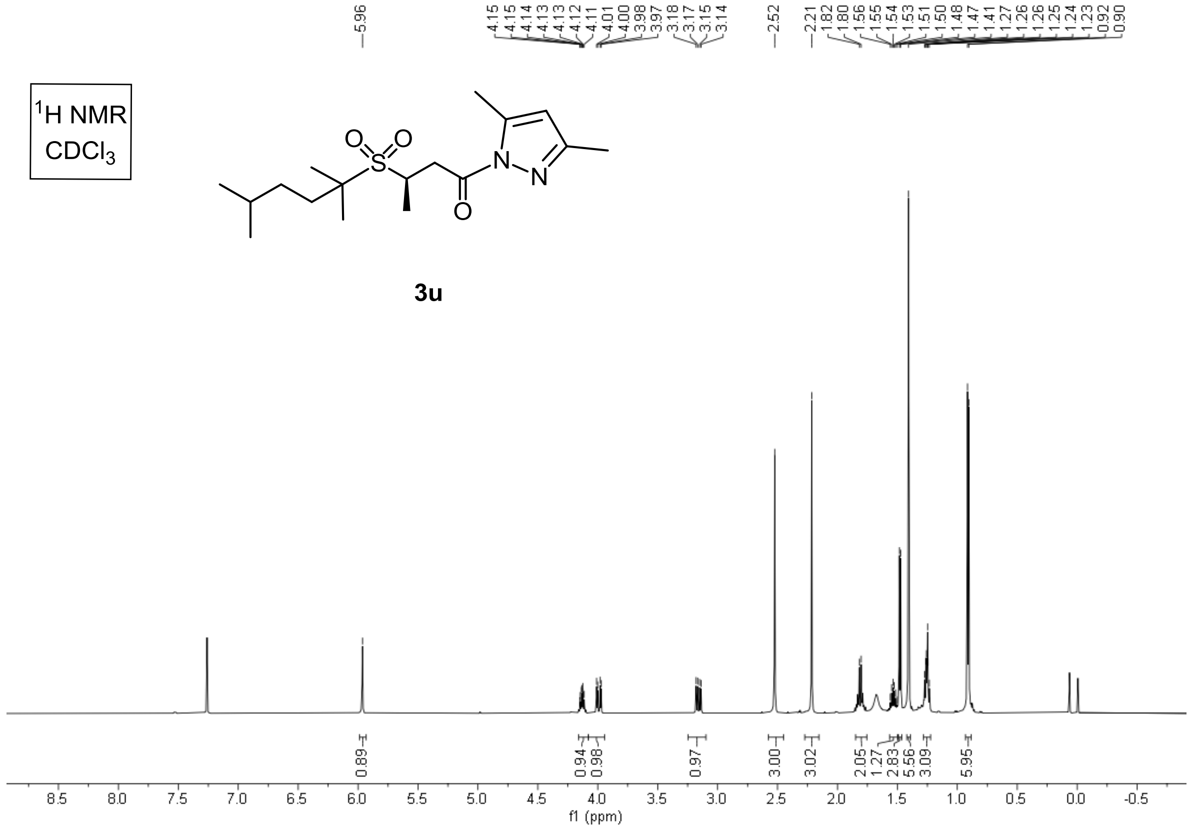

5.96
4.15 4.15 4.14 4.13 4.13 4.12 4.11 4.01 4.00 3.98 3.97 3.18 3.17 3.15 3.14
2.52
2.21 1.82 1.80 1.56 1.55 1.54 1.53 1.51 1.50 1.48 1.47 1.41 1.27 1.26 1.26 1.25 1.24 1.23 0.92 0.90
1H NMR
CDCl3
0.89 0.94 0.98 0.97 3.00 3.02 2.05 1.27 2.83 5.56 3.09 5.95
f1 (ppm)

3u

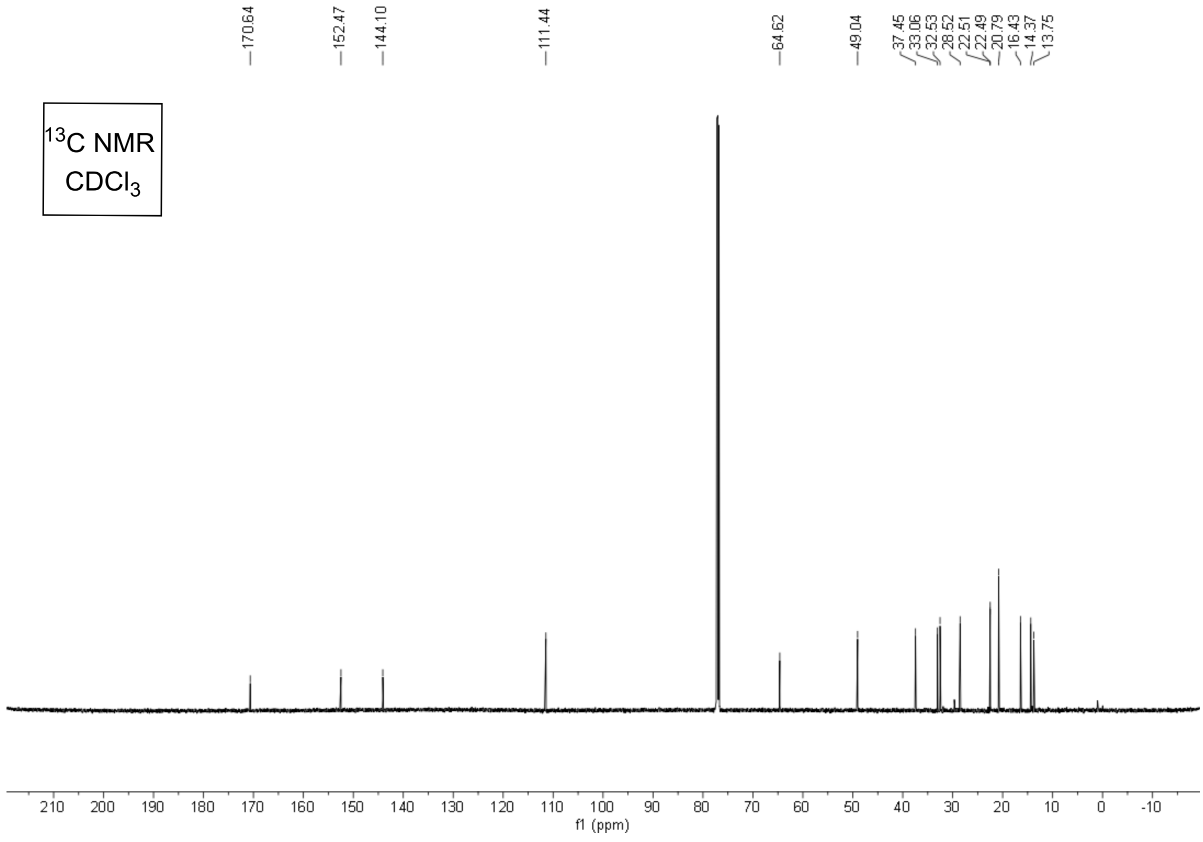

170.64
152.47
144.10
111.44
64.62
49.04
37.45 33.06 32.53 28.52 22.51 22.49 20.79 16.43 14.37 13.75
13C NMR
CDCl3
f1 (ppm)

^{1}H NMR
$CDCl_3$

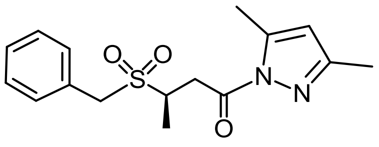

3v

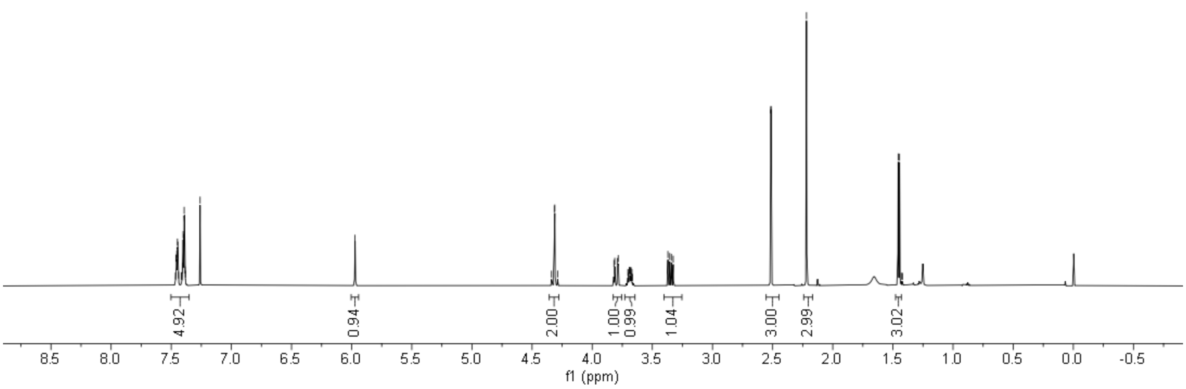

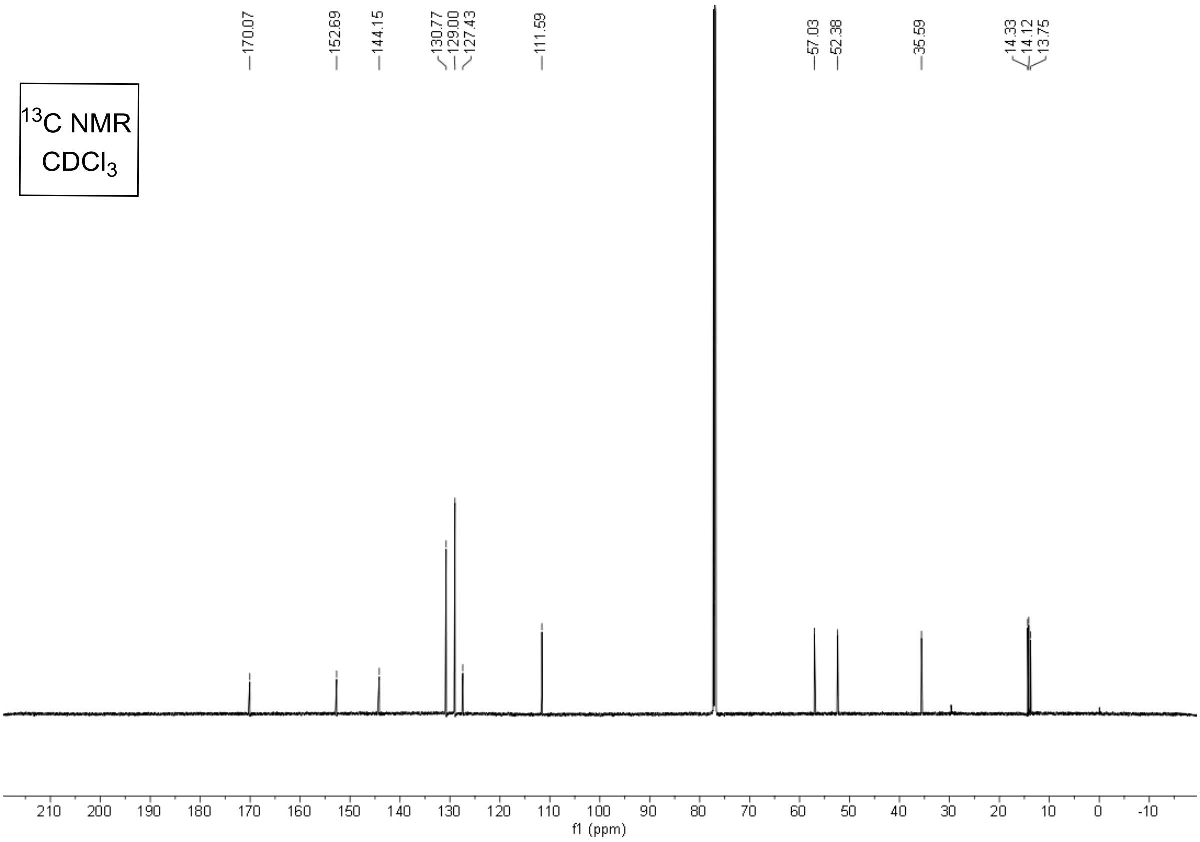

^1H NMR
$CDCl_3$

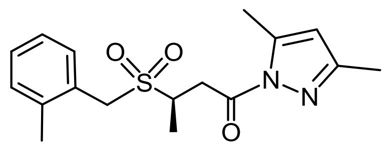

3w

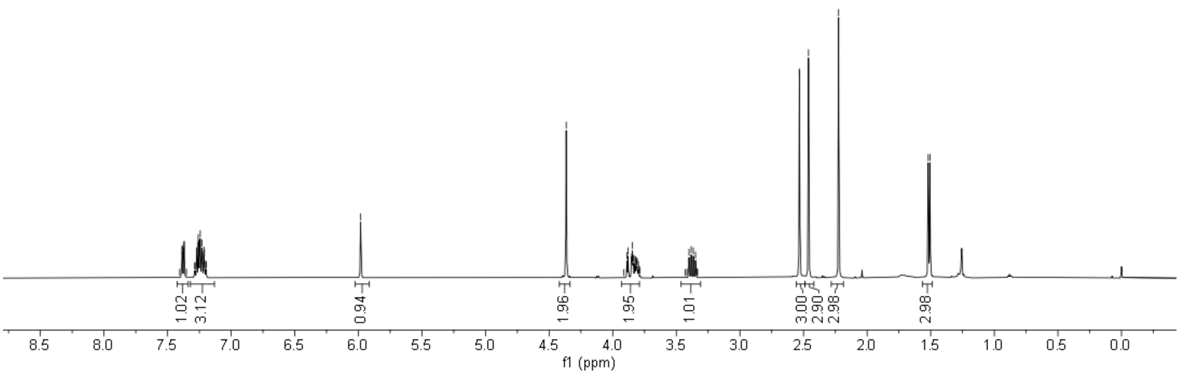

^{13}C NMR
$CDCl_3$

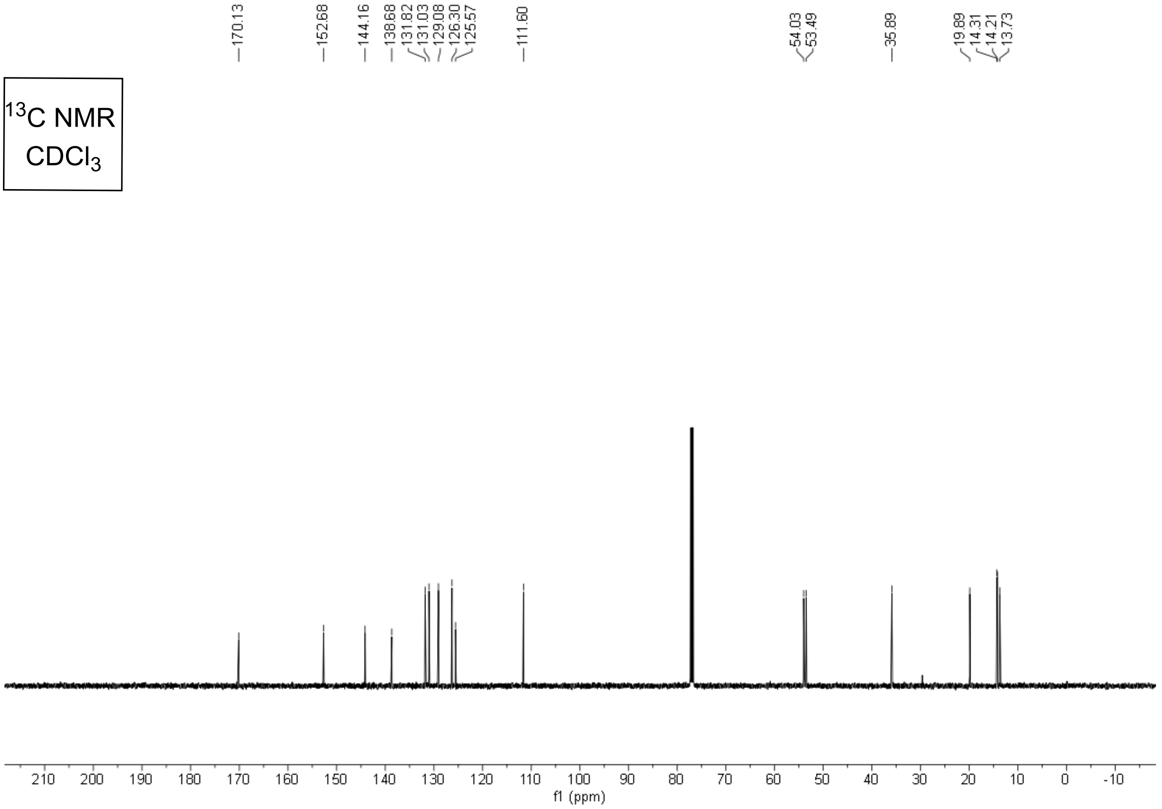

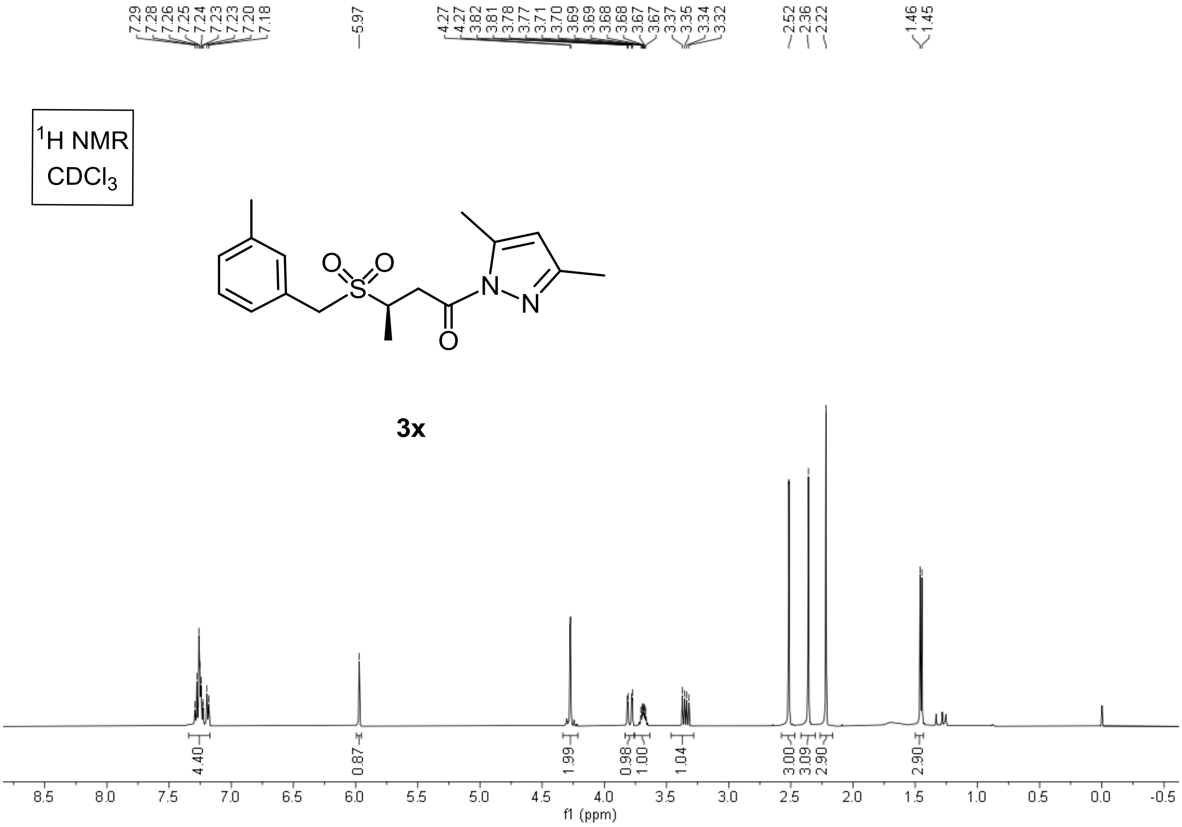
7.29 7.28 7.26 7.25 7.24 7.23 7.23 7.20 7.18
5.97
4.27 4.27 3.82 3.81 3.78 3.77 3.71 3.70 3.69 3.69 3.68 3.68 3.67 3.67 3.37 3.35 3.34 3.32
2.52 2.36 2.22
1.46 1.45
1H NMR
CDCl3
O
S
O
O
N
N
3x
4.40
0.87
1.99
0.98
1.00
1.04
3.00
3.09
2.90
2.90
8.5 8.0 7.5 7.0 6.5 6.0 5.5 5.0 4.5 4.0 3.5 3.0 2.5 2.0 1.5 1.0 0.5 0.0 -0.5
f1 (ppm)

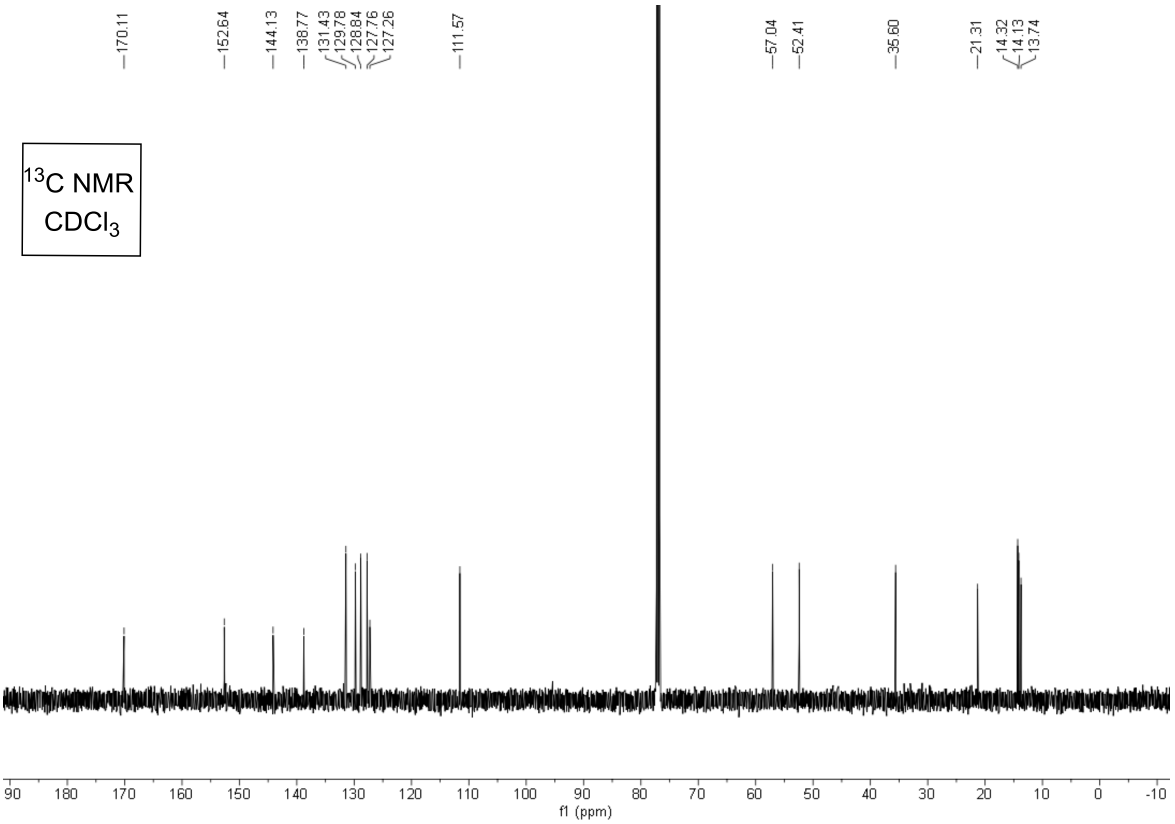
170.11
152.64
144.13
138.77
131.43 129.78 128.84 127.76 127.26
111.57
57.04
52.41
35.60
21.31
14.32 14.13 13.74
13C NMR
CDCl3
90 180 170 160 150 140 130 120 110 100 90 80 70 60 50 40 30 20 10 0 -10
f1 (ppm)

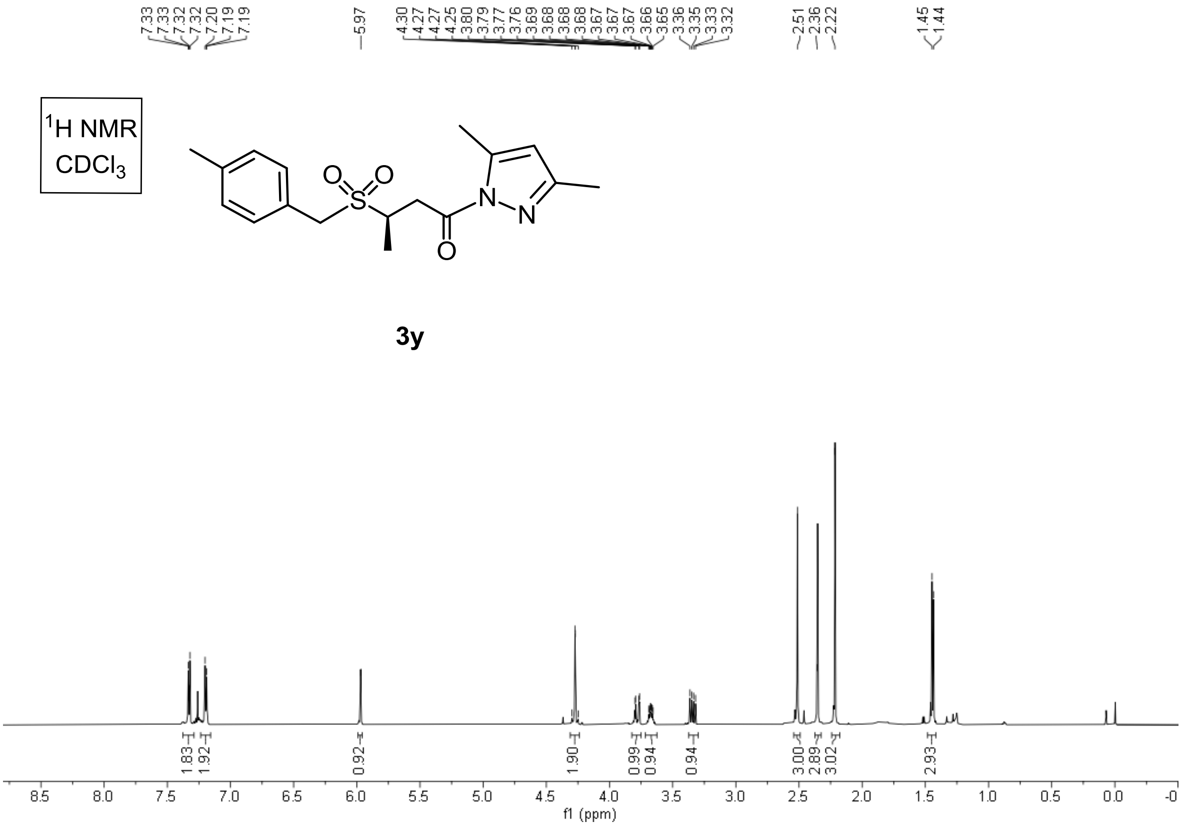
7.33 7.33 7.32 7.32 7.20 7.19 7.19
5.97
4.30 4.27 4.27 4.25 3.80 3.79 3.77 3.76 3.69 3.68 3.68 3.68 3.67 3.67 3.67 3.66 3.65 3.36 3.35 3.33 3.32
2.51 2.36 2.22
1.45 1.44
1H NMR
CDCl3
O
S
O
O
N
N
3y
1.83 1.92
0.92
1.90
0.99 0.94
0.94
3.00 2.89 3.02
2.93
8.5 8.0 7.5 7.0 6.5 6.0 5.5 5.0 4.5 4.0 3.5 3.0 2.5 2.0 1.5 1.0 0.5 0.0 -0
f1 (ppm)

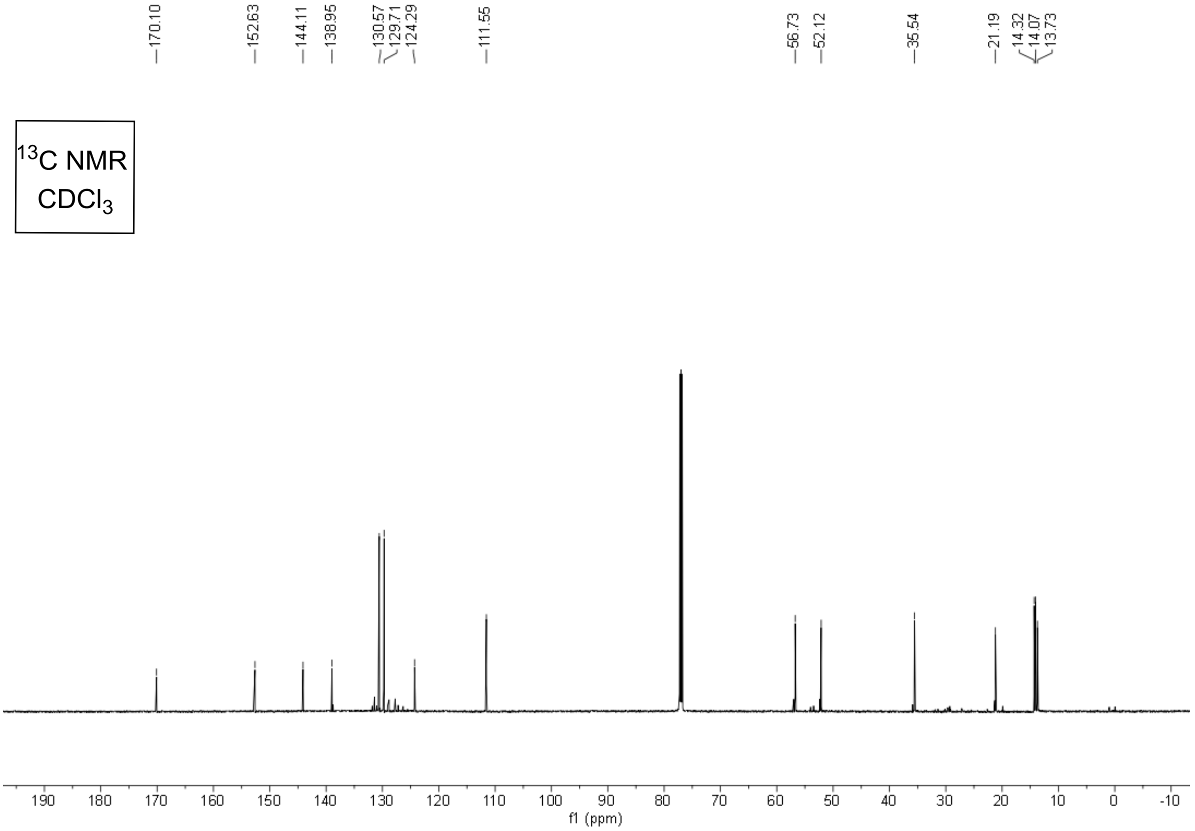
170.10
152.63
144.11
138.95
130.57 129.71 124.29
111.55
56.73
52.12
35.54
21.19
14.32 14.07 13.73
13C NMR
CDCl3
190 180 170 160 150 140 130 120 110 100 90 80 70 60 50 40 30 20 10 0 -10
f1 (ppm)

^{1}H NMR
$CDCl_3$

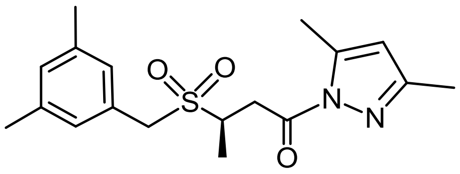

3z

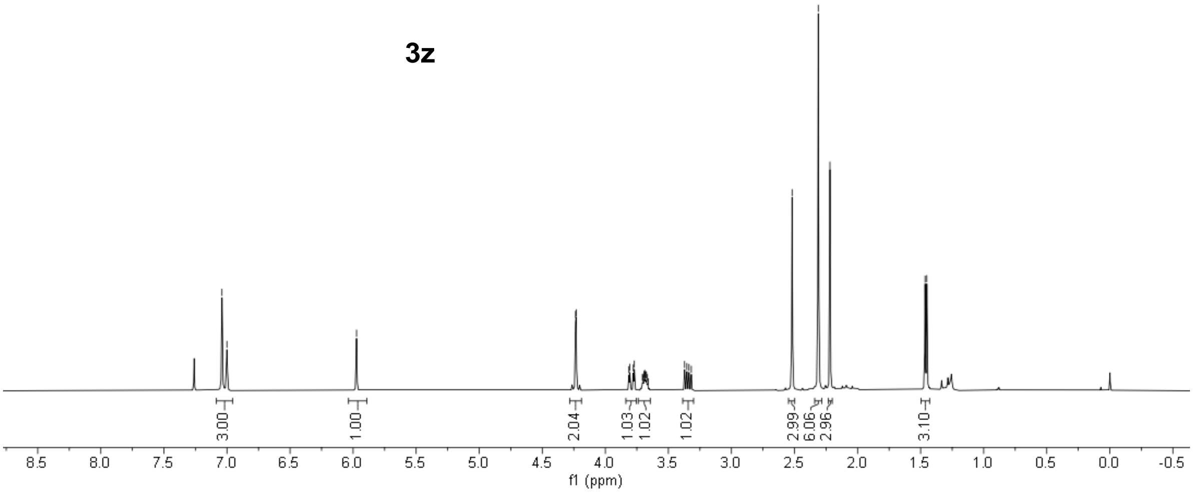

^{13}C NMR
$CDCl_3$

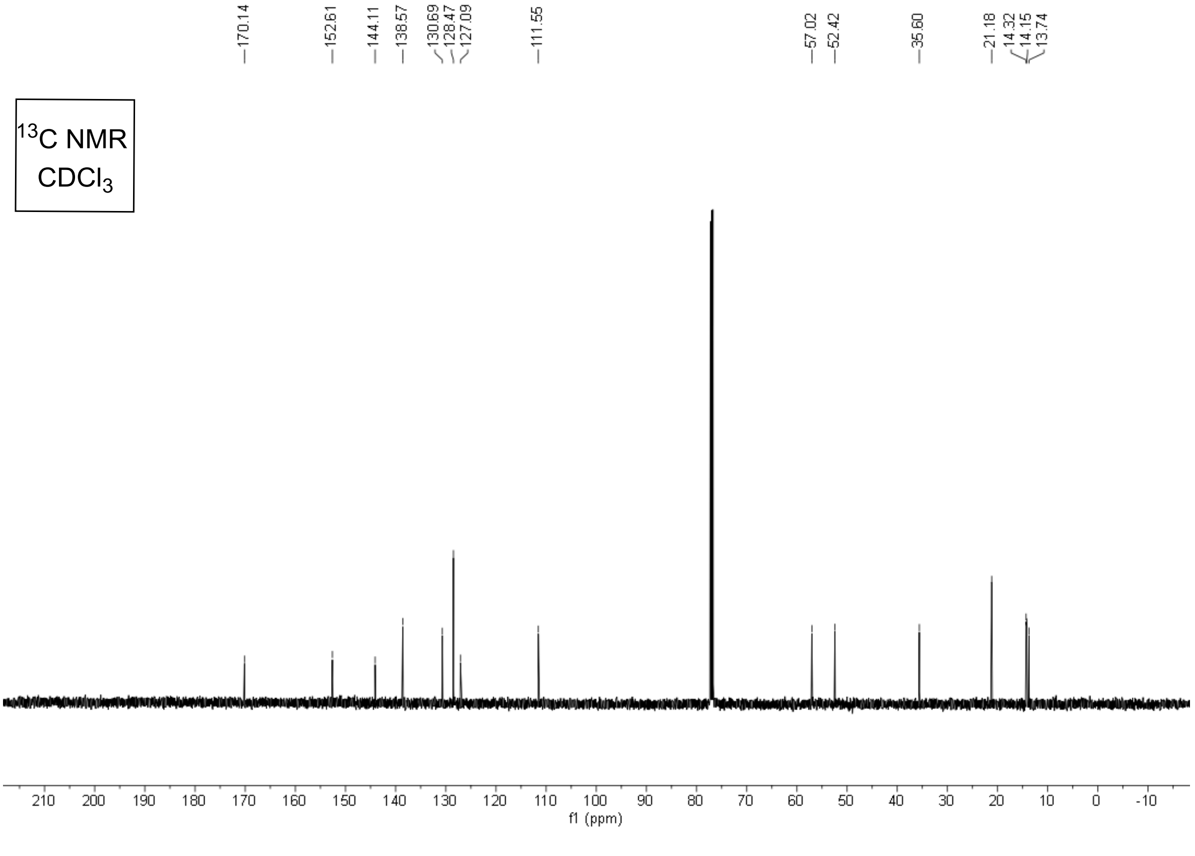

^1H NMR
$CDCl_3$

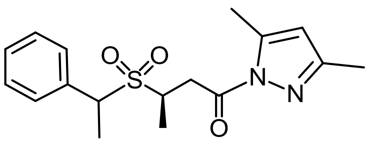

3za

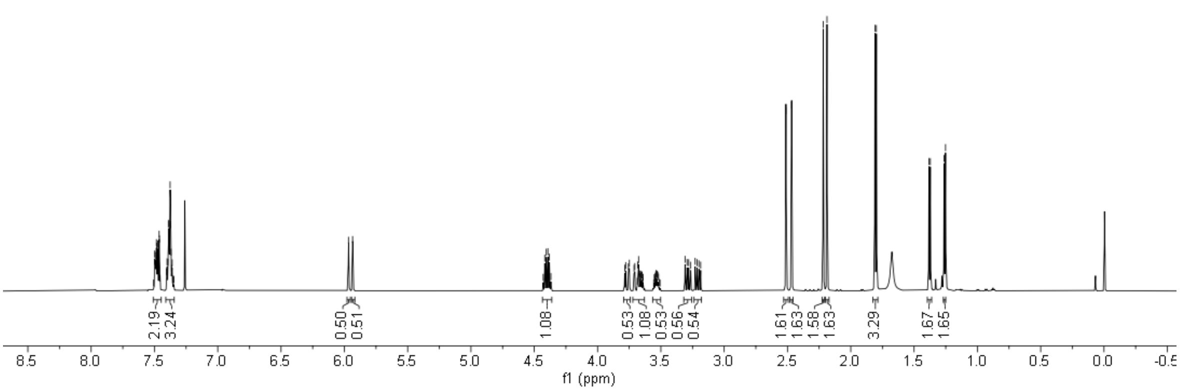

^{13}C NMR
$CDCl_3$

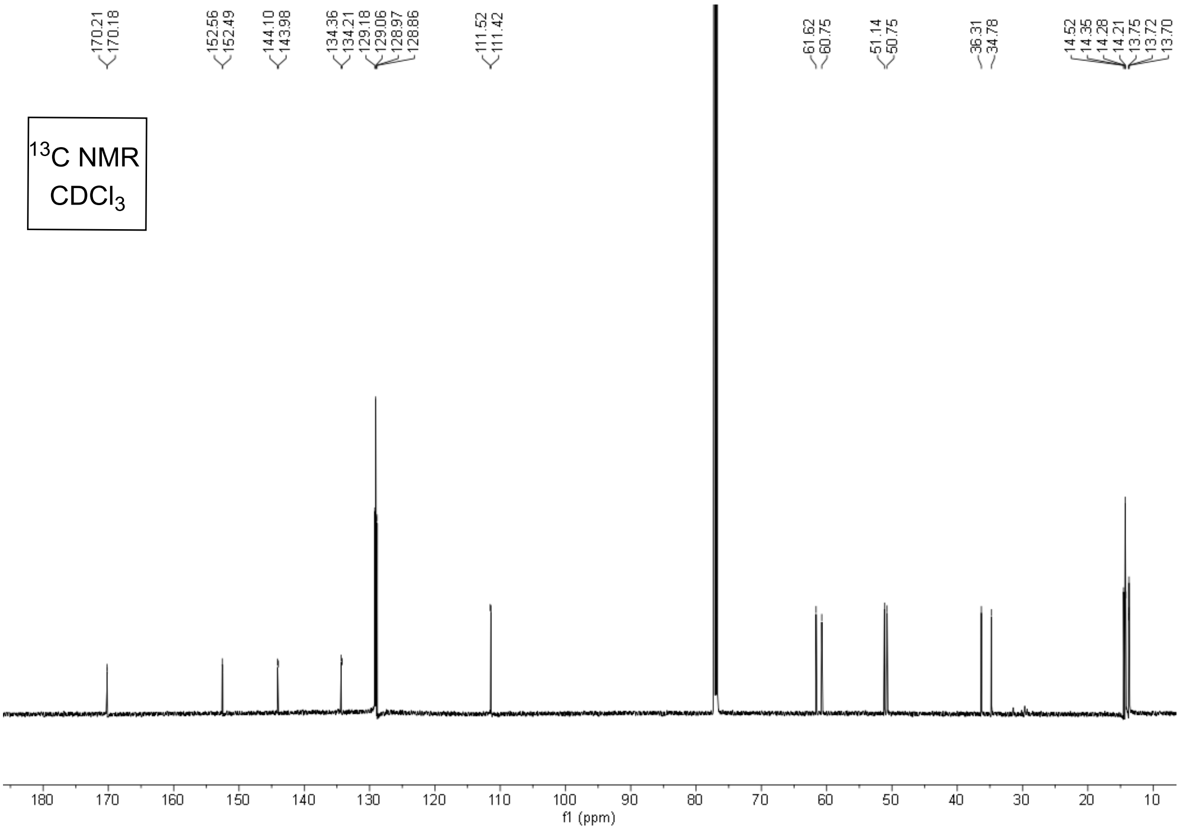

^{1}H NMR
CDCl3

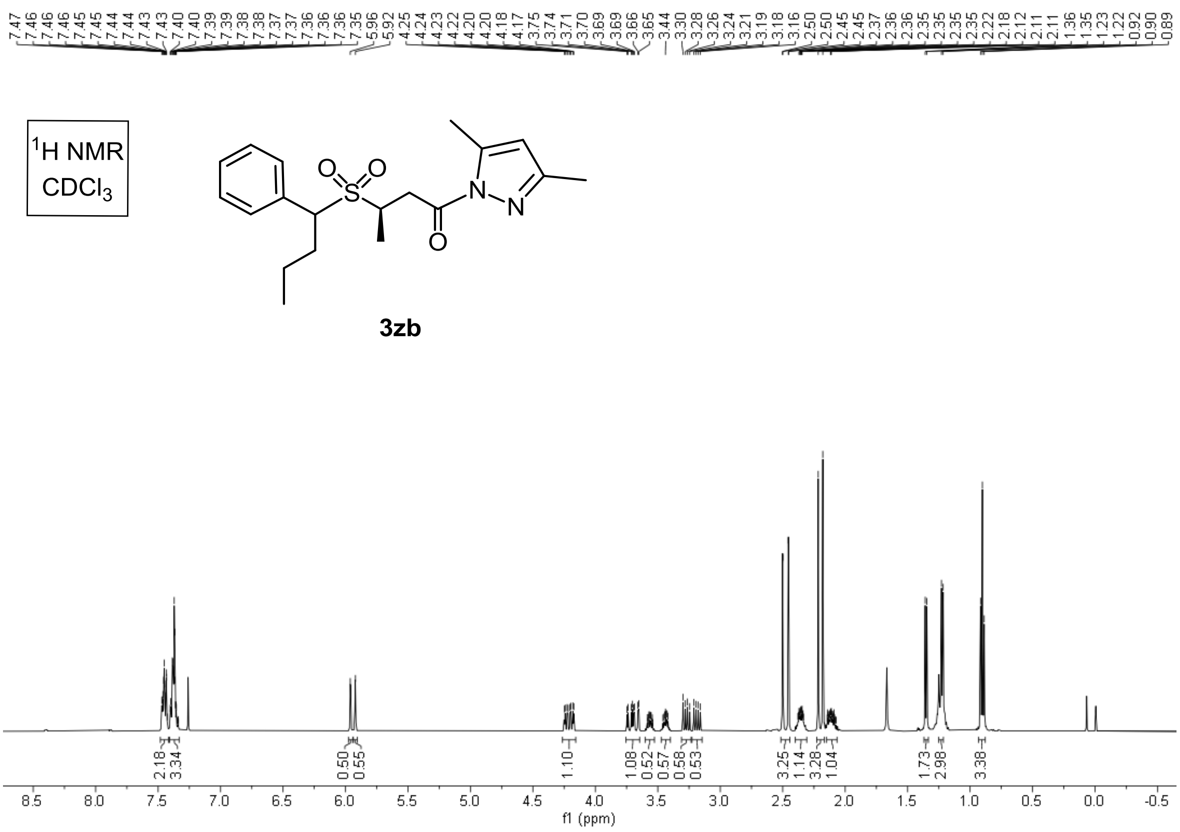

3zb

^{13}C NMR
$CDCl_3$

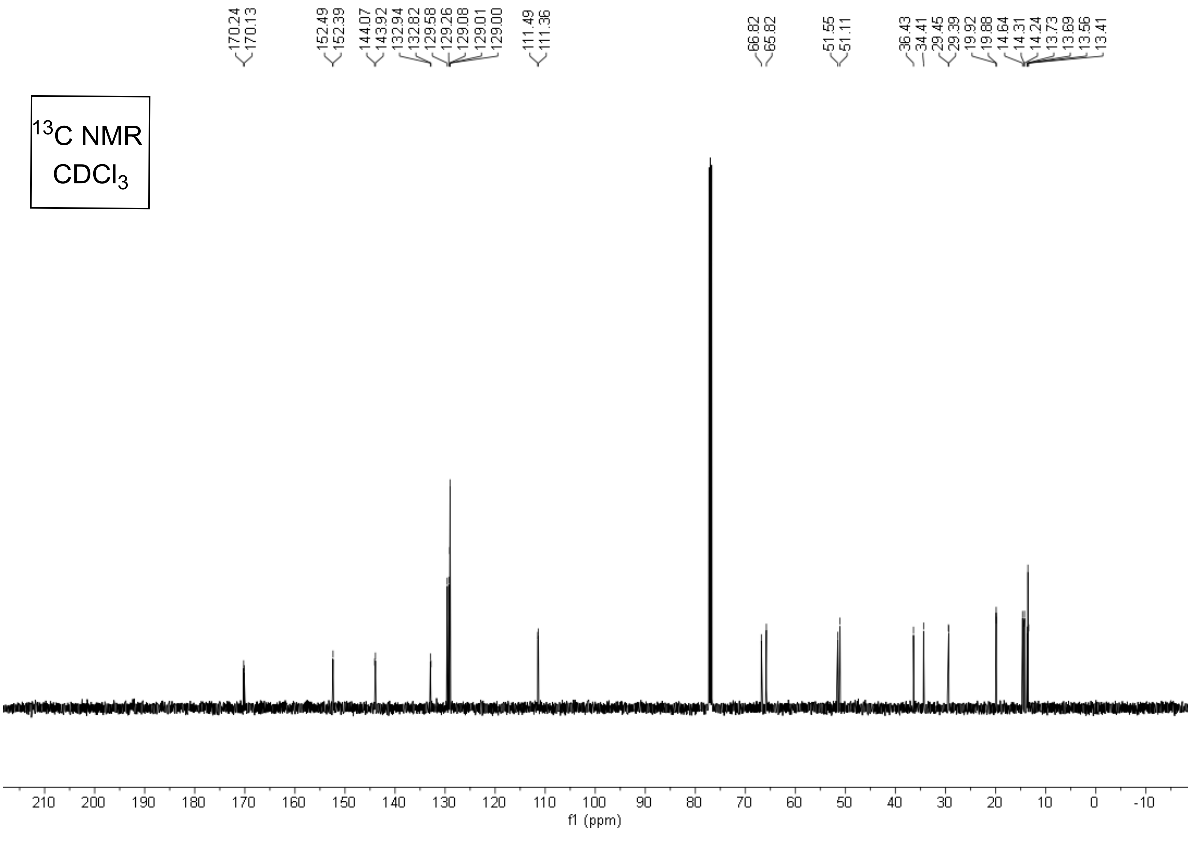

^{1}H NMR
$CDCl_3$

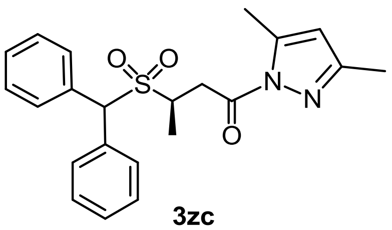

3zc

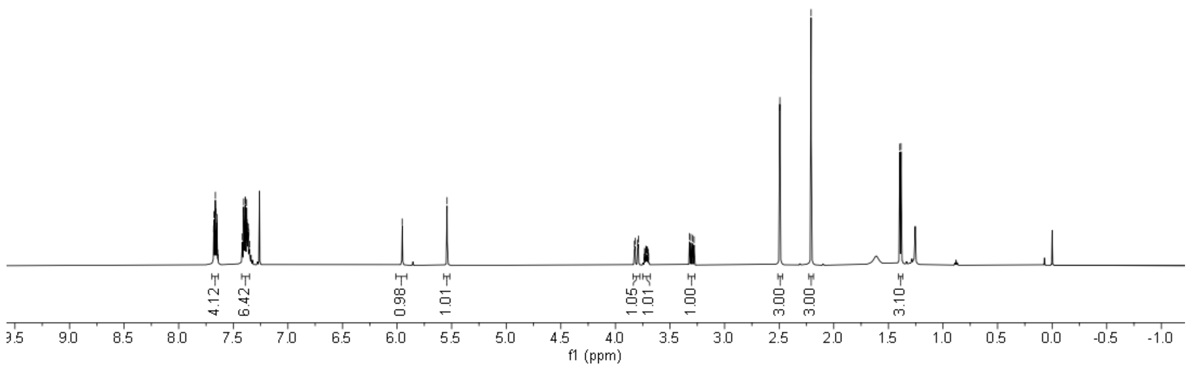

^{13}C NMR
$CDCl_3$

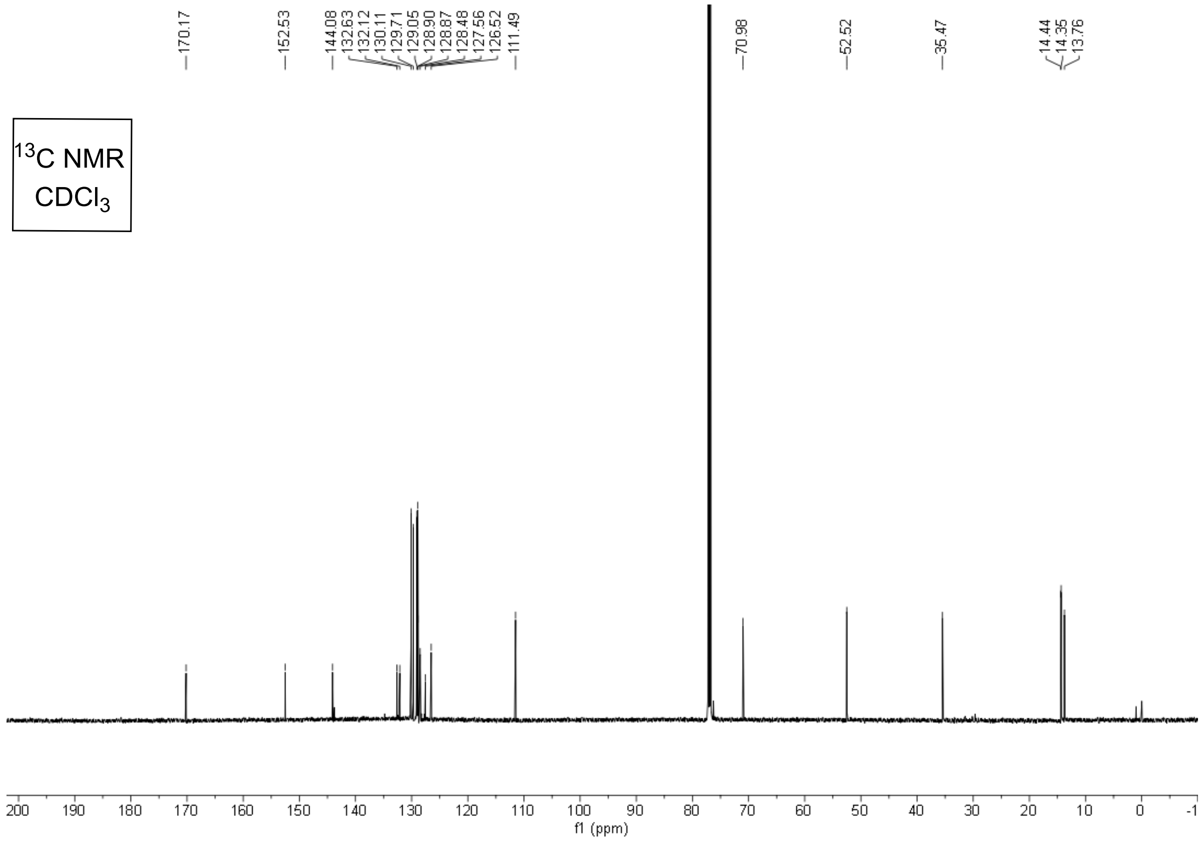

^{1}H NMR
$CDCl_3$

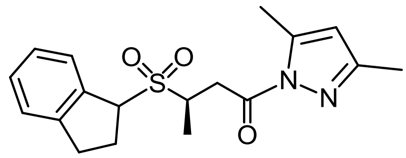

3zd

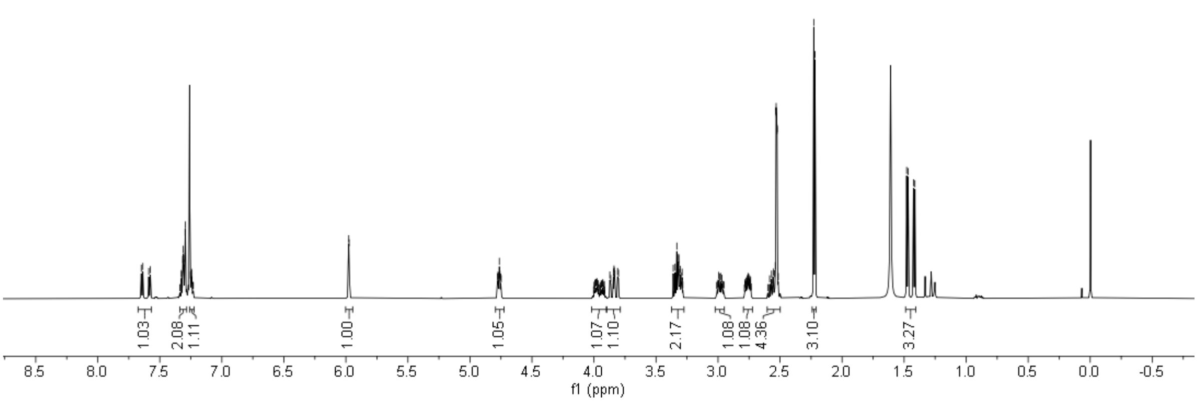

^{13}C NMR
$CDCl_3$

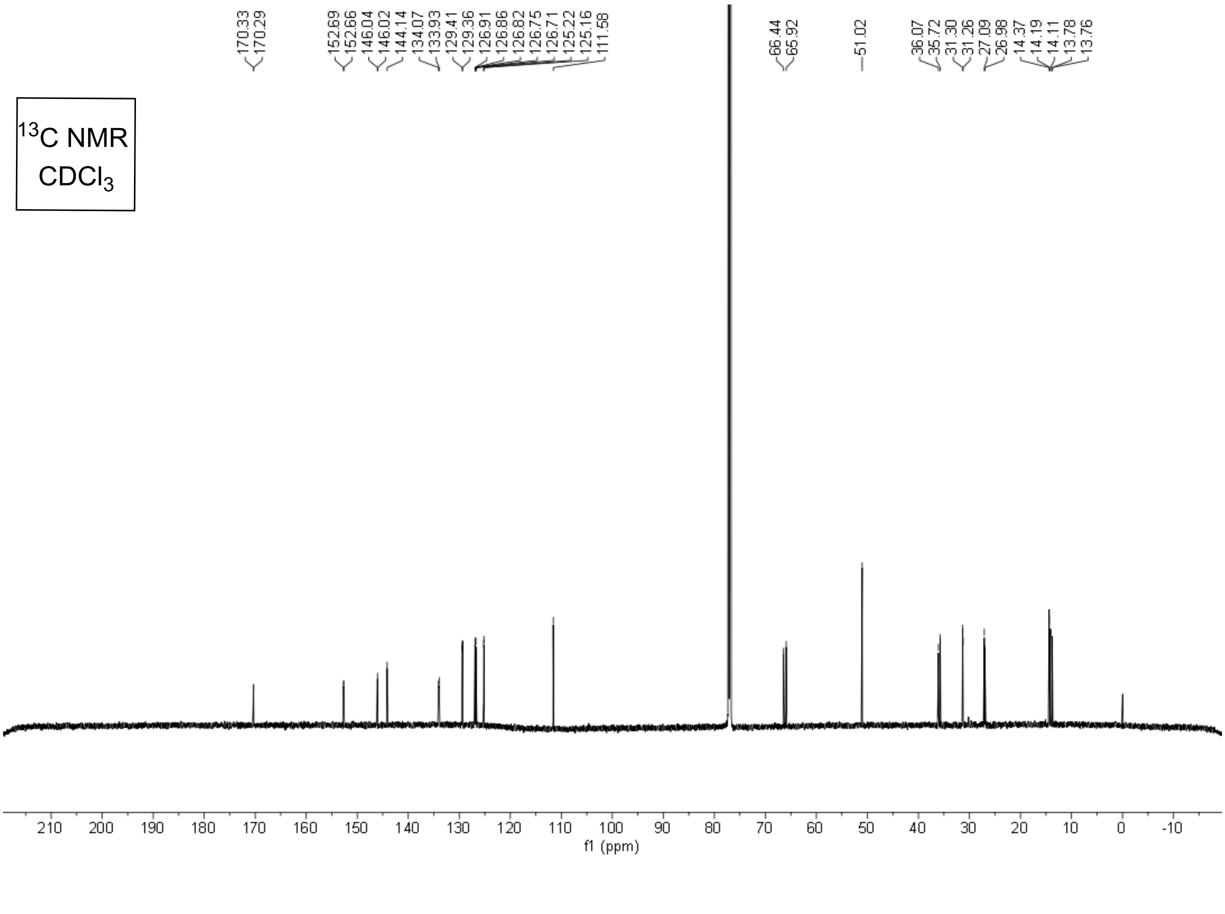

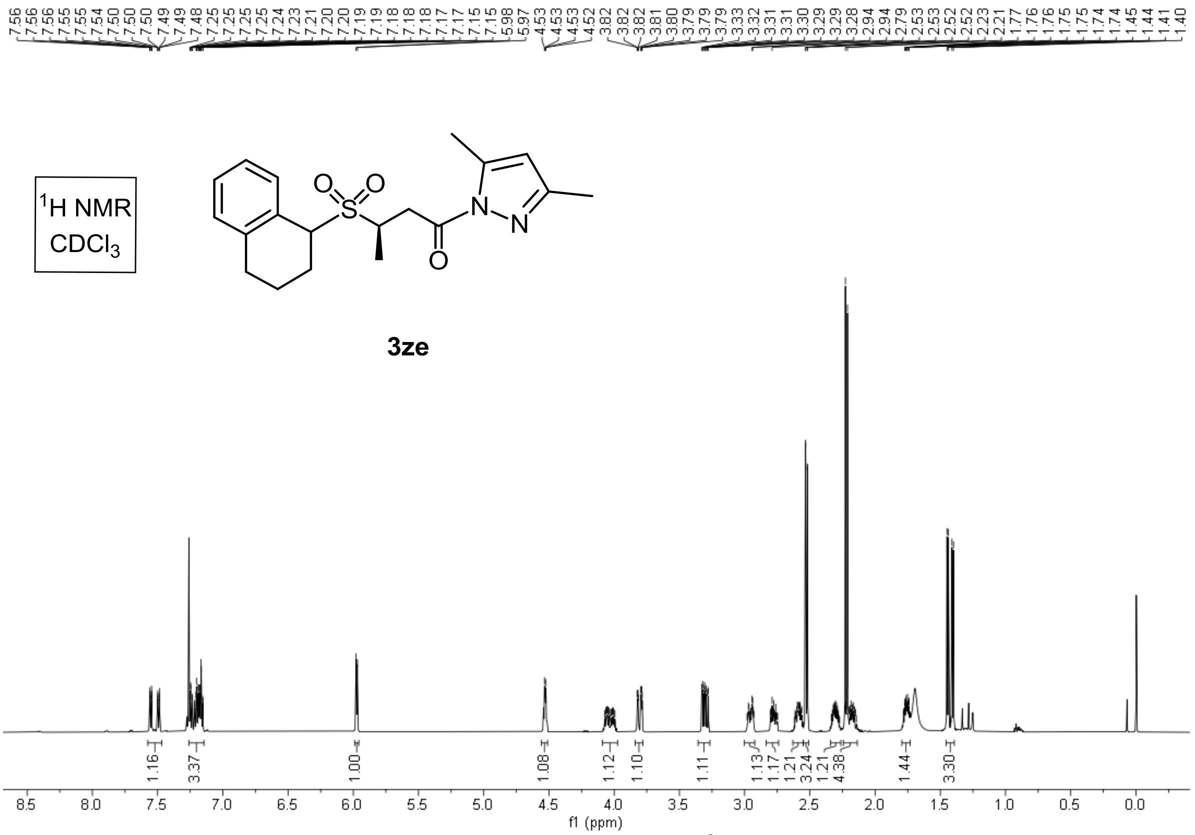

7.56 7.56 7.55 7.55 7.54 7.50 7.50 7.49 7.49 7.48 7.25 7.25 7.25 7.24 7.23 7.21 7.21 7.20 7.20 7.19 7.19 7.18 7.18 7.17 7.17 7.15 5.98 5.97 4.53 4.53 4.53 4.52 3.82 3.82 3.81 3.80 3.79 3.79 3.79 3.33 3.32 3.31 3.31 3.30 3.29 3.29 3.28 2.94 2.94 2.94 2.79 2.53 2.53 2.52 2.52 2.23 2.21 1.77 1.76 1.76 1.75 1.74 1.74 1.45 1.41 1.41 1.40
¹H NMR
CDCl₃
O
S
O
N
N
O
3ze
1.16
3.37
1.00
1.08
1.12
1.10
1.11
1.13
1.17
1.21
3.24
1.21
4.38
1.44
3.30
8.5 8.0 7.5 7.0 6.5 6.0 5.5 5.0 4.5 4.0 3.5 3.0 2.5 2.0 1.5 1.0 0.5 0.0
f1 (ppm)

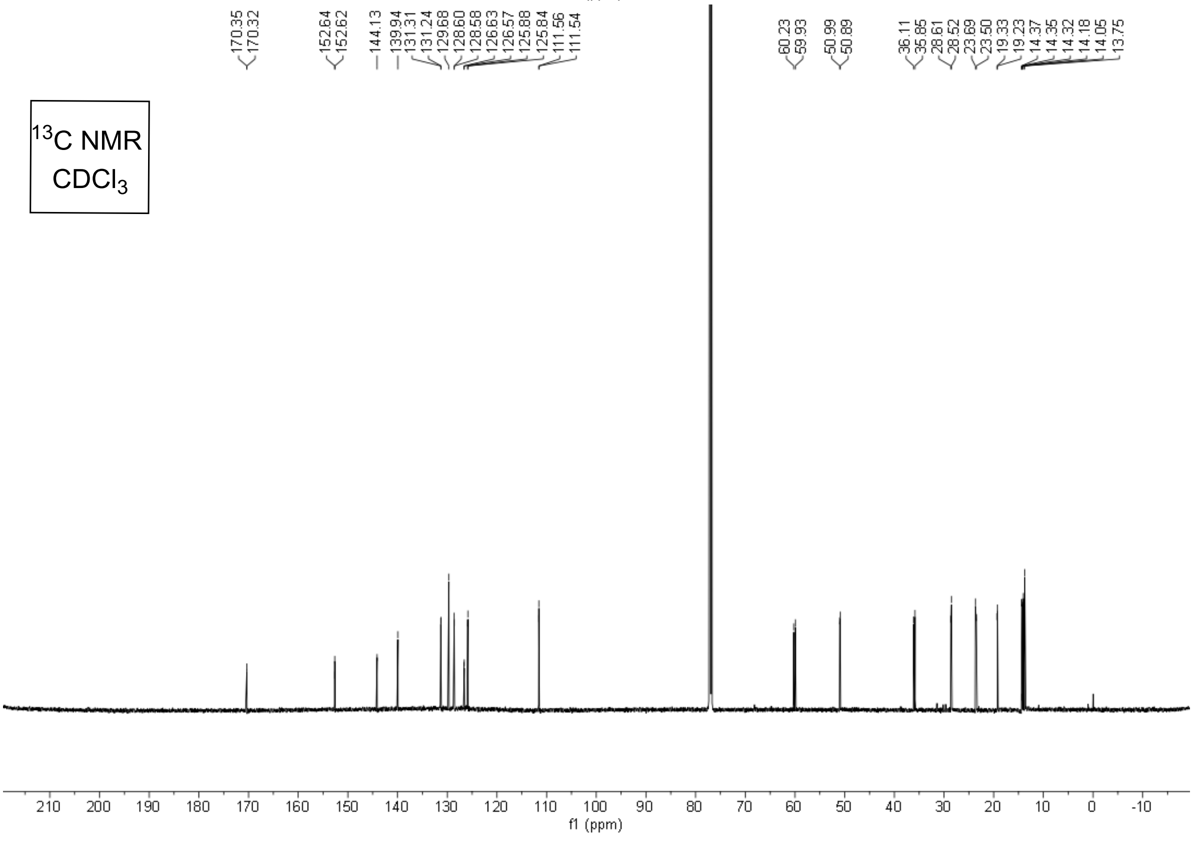

170.35 170.32 152.64 152.62 144.13 139.94 131.31 131.24 129.68 128.60 128.58 126.63 126.57 125.88 125.84 111.56 111.54 60.23 59.93 50.99 50.89 36.11 35.85 28.61 28.52 23.69 23.50 19.33 19.23 14.37 14.35 14.32 14.18 14.05 13.75
¹³C NMR
CDCl₃
210 200 190 180 170 160 150 140 130 120 110 100 90 80 70 60 50 40 30 20 10 0 -10
f1 (ppm)

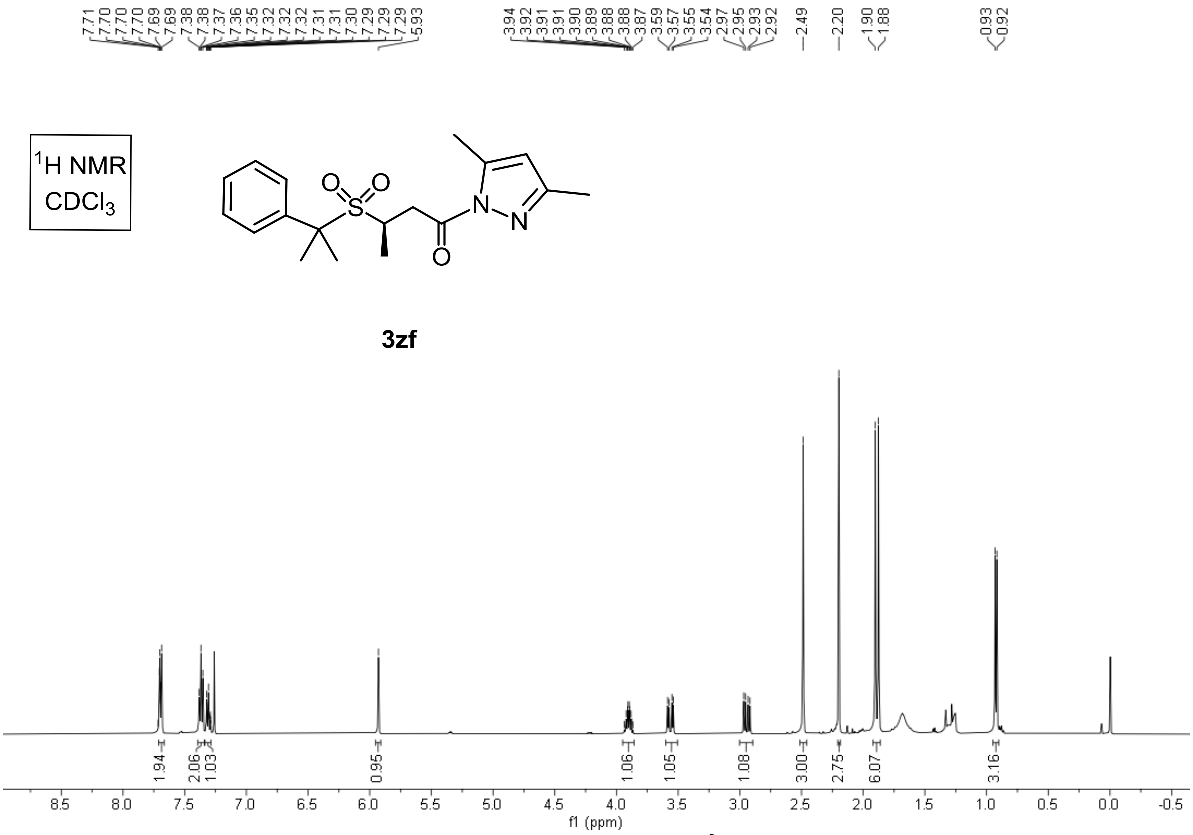

7.71
7.70
7.70
7.69
7.69
7.38
7.38
7.37
7.36
7.35
7.32
7.32
7.32
7.31
7.30
7.29
7.29
7.29
5.93
3.94
3.92
3.91
3.91
3.90
3.89
3.88
3.88
3.87
3.59
3.57
3.55
3.54
2.97
2.95
2.93
2.92
2.49
2.20
1.90
1.88
0.93
0.92
1H NMR
CDCl3
O
O
S
N
N
O
1.94
2.06
1.03
0.95
1.06
1.05
1.08
3.00
2.75
6.07
3.16
8.5 8.0 7.5 7.0 6.5 6.0 5.5 5.0 4.5 4.0 3.5 3.0 2.5 2.0 1.5 1.0 0.5 0.0 -0.5
f1 (ppm)

3zf

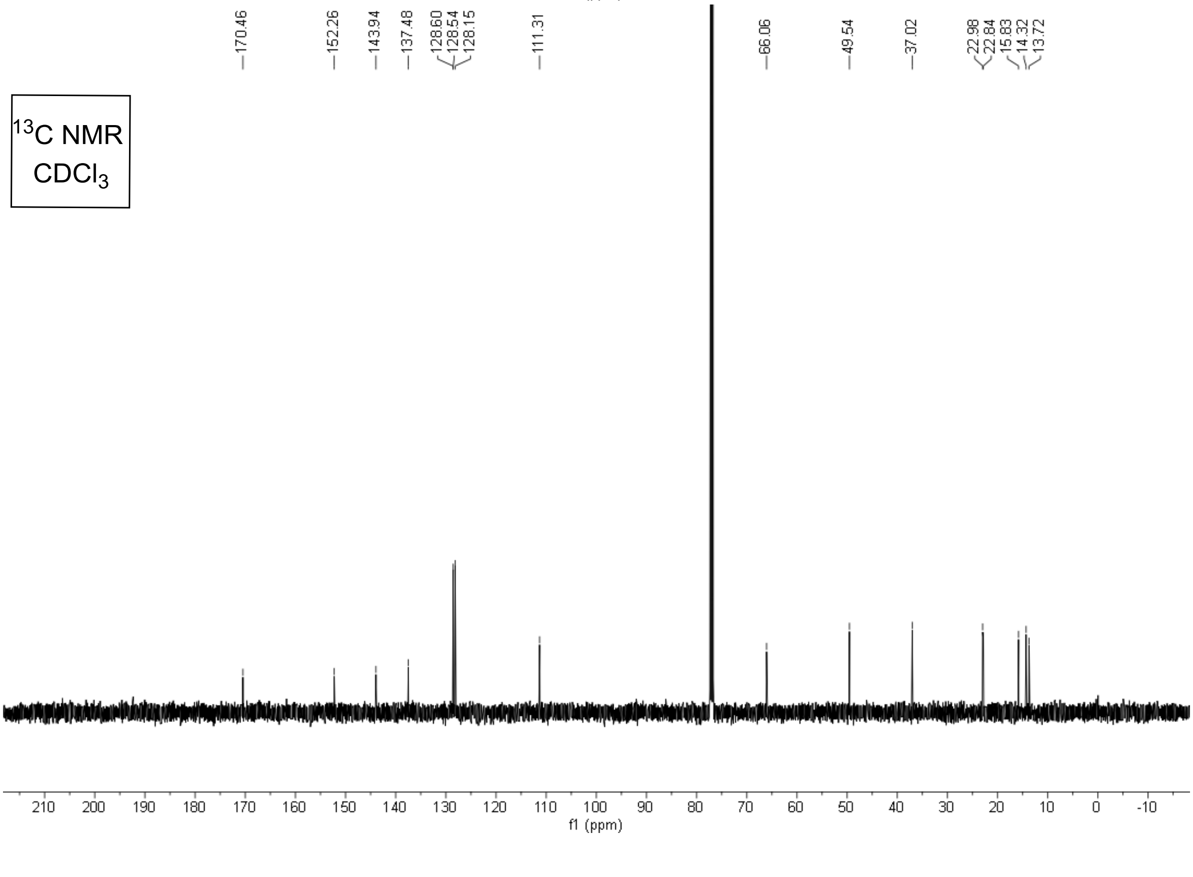

170.46
152.26
143.94
137.48
128.60
128.54
128.15
111.31
66.06
49.54
37.02
22.98
22.84
15.83
14.32
13.72
13C NMR
CDCl3
210 200 190 180 170 160 150 140 130 120 110 100 90 80 70 60 50 40 30 20 10 0 -10
f1 (ppm)

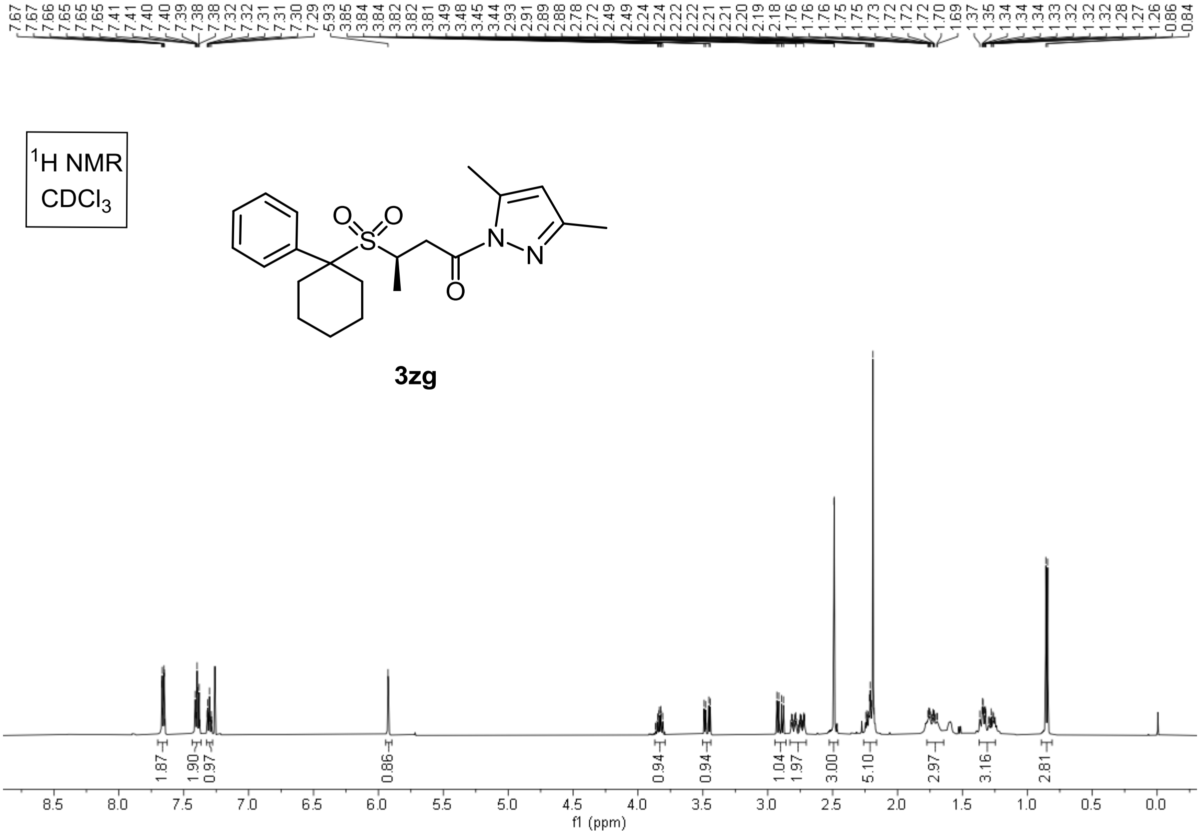
7.67 7.67 7.66 7.65 7.65 7.41 7.41 7.40 7.40 7.39 7.38 7.38 7.32 7.32 7.31 7.31 7.30 7.29 5.93 3.85 3.84 3.84 3.82 3.82 3.81 3.49 3.48 3.45 3.44 2.93 2.91 2.89 2.88 2.78 2.72 2.49 2.49 2.24 2.24 2.22 2.22 2.21 2.21 2.20 2.19 2.18 1.76 1.76 1.75 1.75 1.73 1.72 1.72 1.72 1.70 1.69 1.37 1.35 1.34 1.34 1.34 1.33 1.32 1.32 1.32 1.28 1.27 1.26 0.86 0.84
1H NMR
CDCl3
O
S
O
N
N
O
3zg
1.87
1.90
0.97
0.86
0.94
0.94
1.04
1.97
3.00
5.10
2.97
3.16
2.81
8.5 8.0 7.5 7.0 6.5 6.0 5.5 5.0 4.5 4.0 3.5 3.0 2.5 2.0 1.5 1.0 0.5 0.0
f1 (ppm)

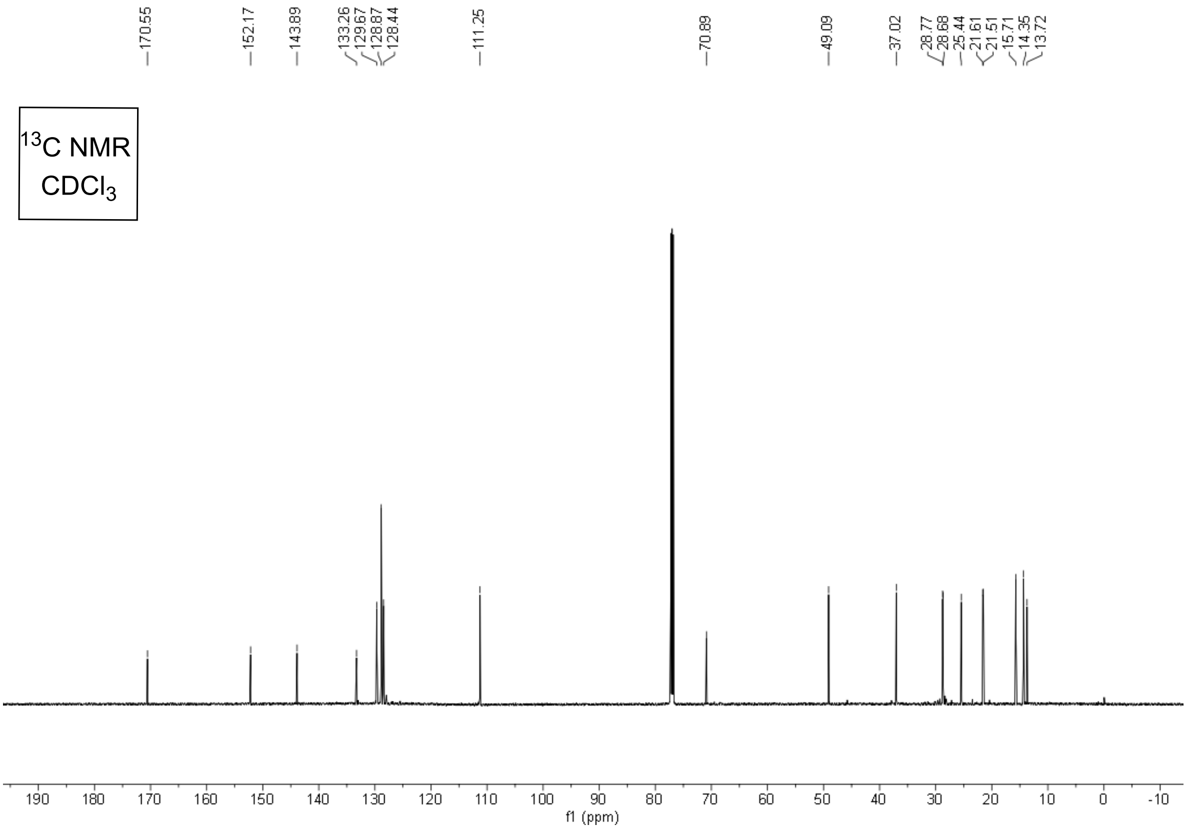
170.55 152.17 143.89 133.26 129.67 128.87 128.44 111.25 70.89 49.09 37.02 28.77 28.68 25.44 21.61 21.51 15.71 14.35 13.72
13C NMR
CDCl3
190 180 170 160 150 140 130 120 110 100 90 80 70 60 50 40 30 20 10 0 -10
f1 (ppm)

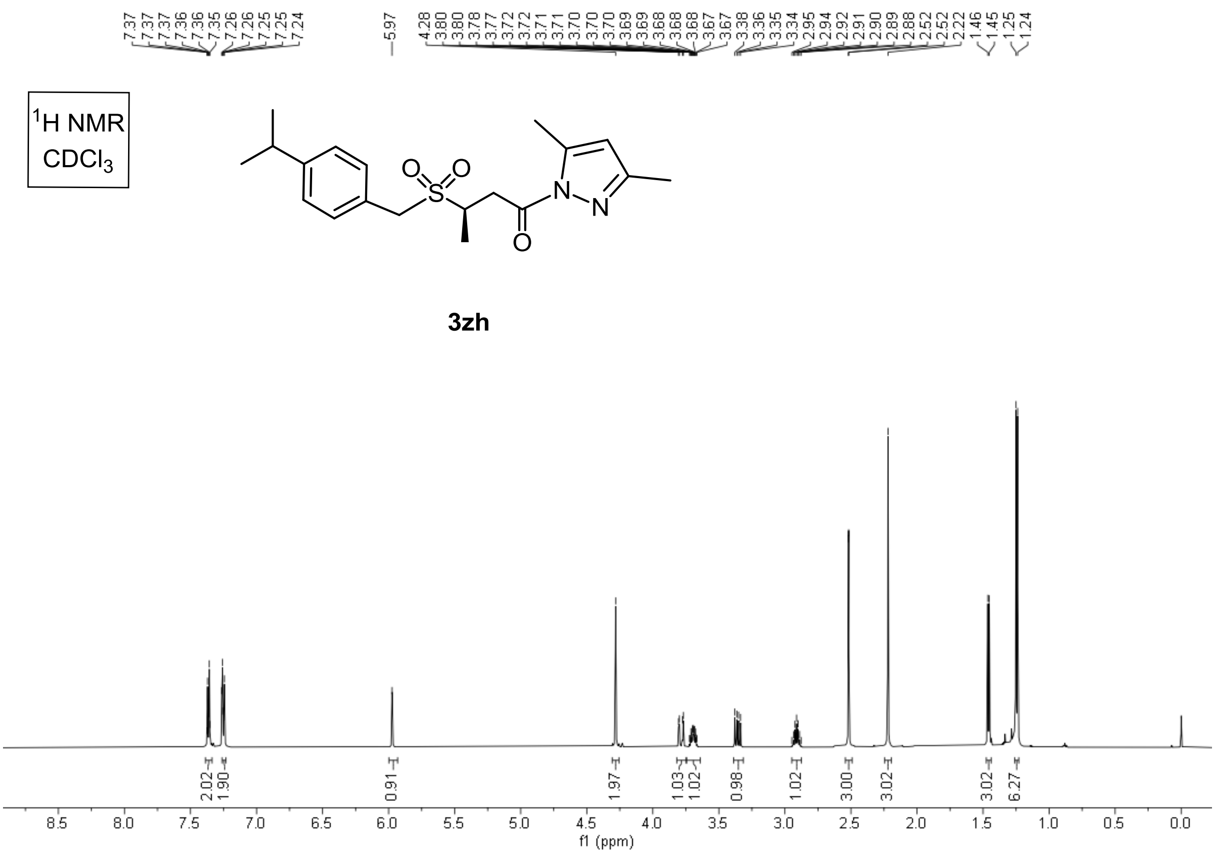

1H NMR
CDCl3
3zh

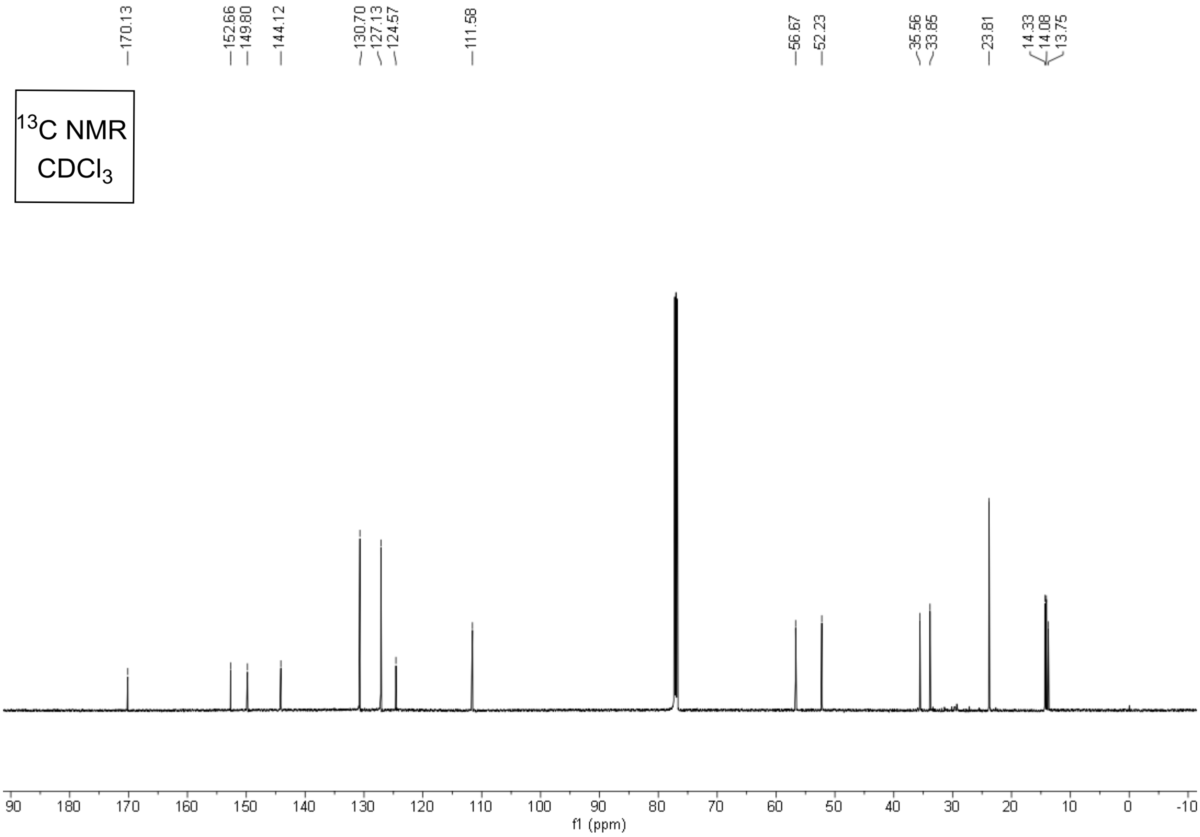

13C NMR
CDCl3

^{1}H NMR
$CDCl_3$

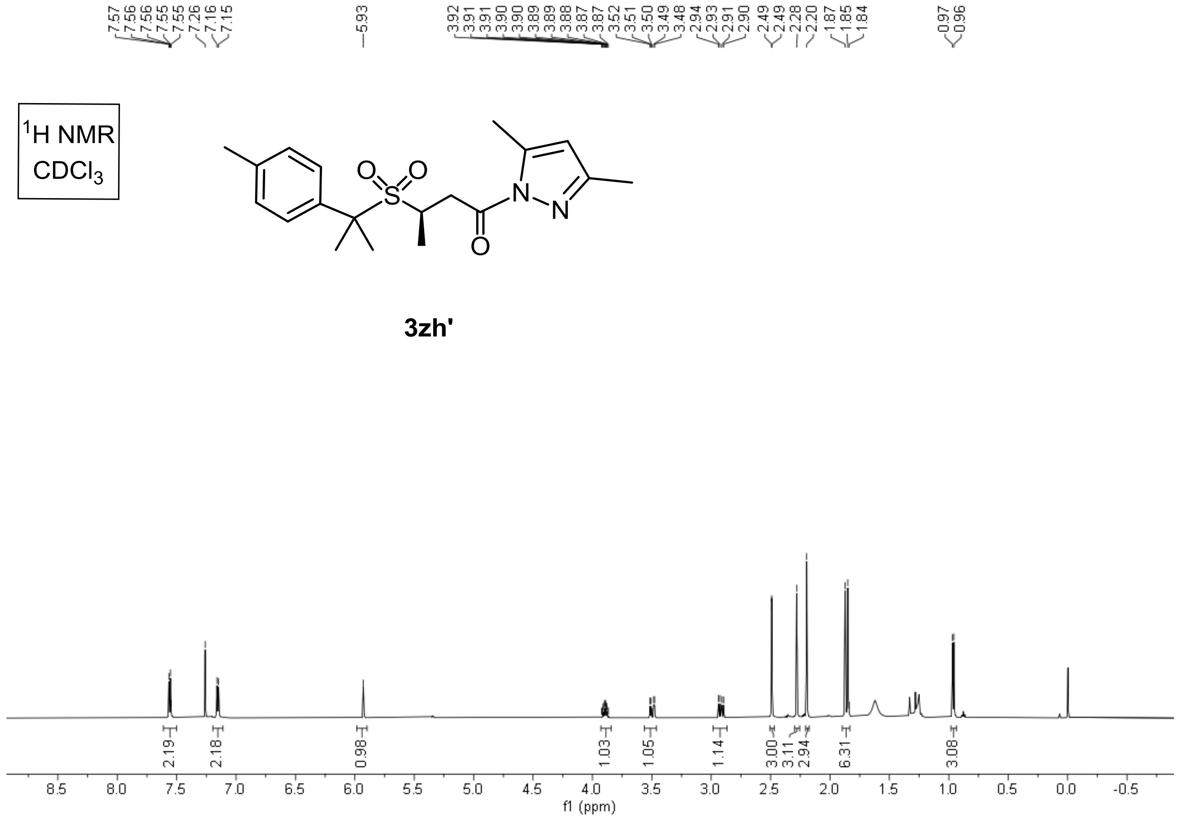

3zh'

^{13}C NMR
$CDCl_3$

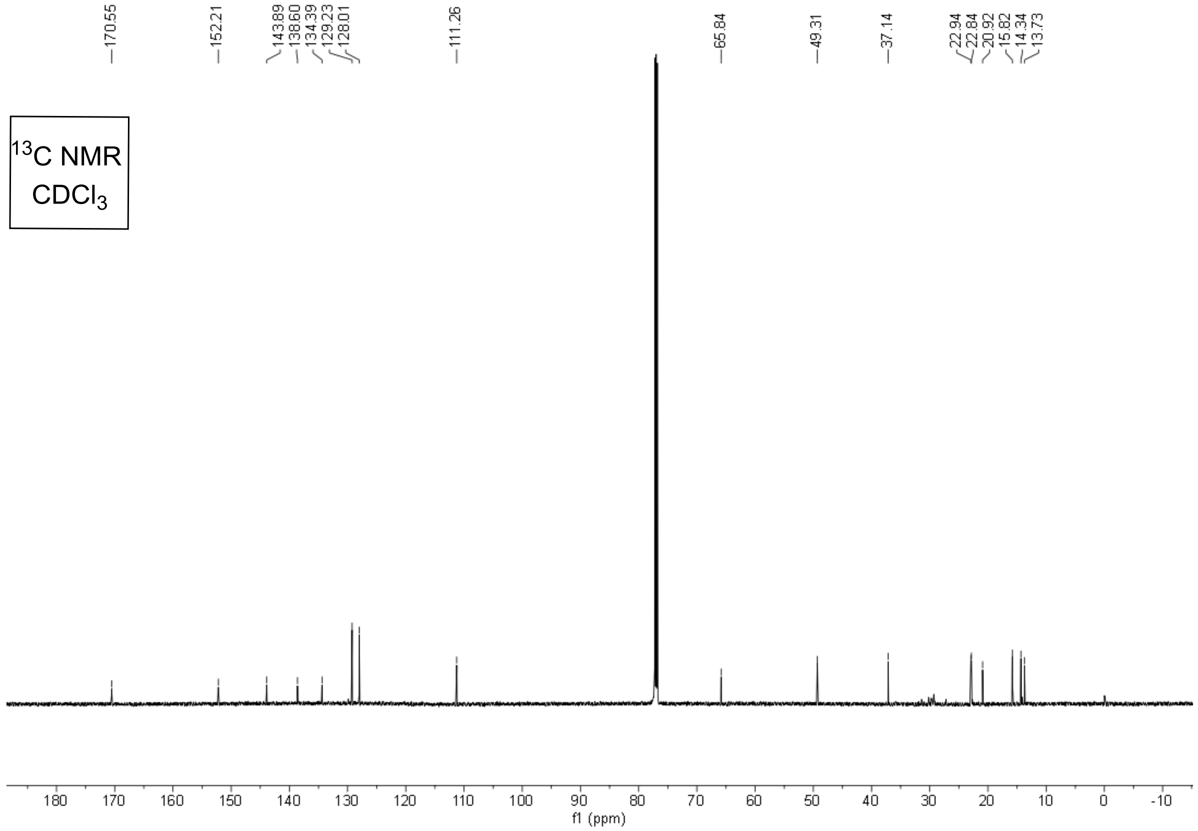

^{1}H NMR
$CDCl_3$

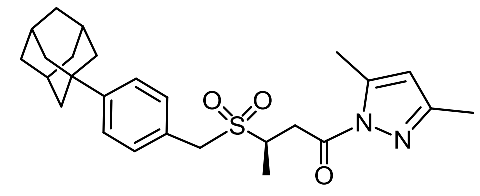

3zi

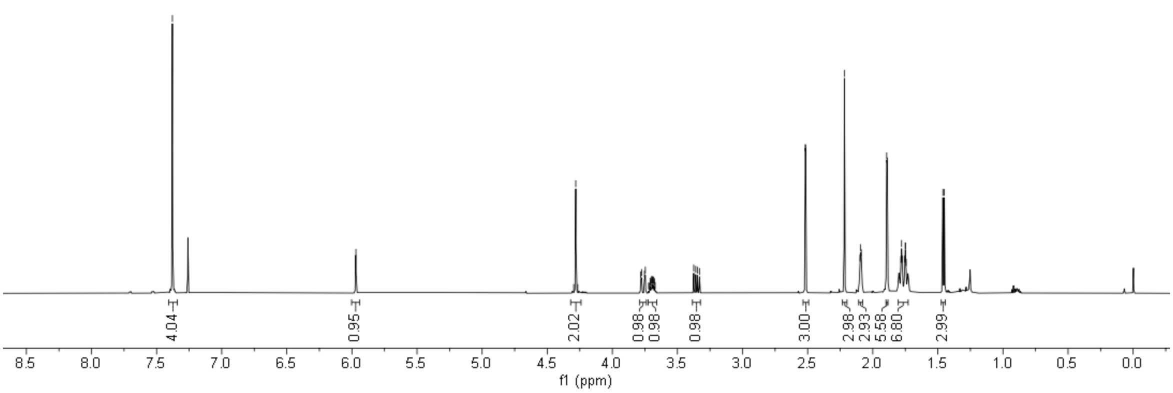

^{13}C NMR
$CDCl_3$

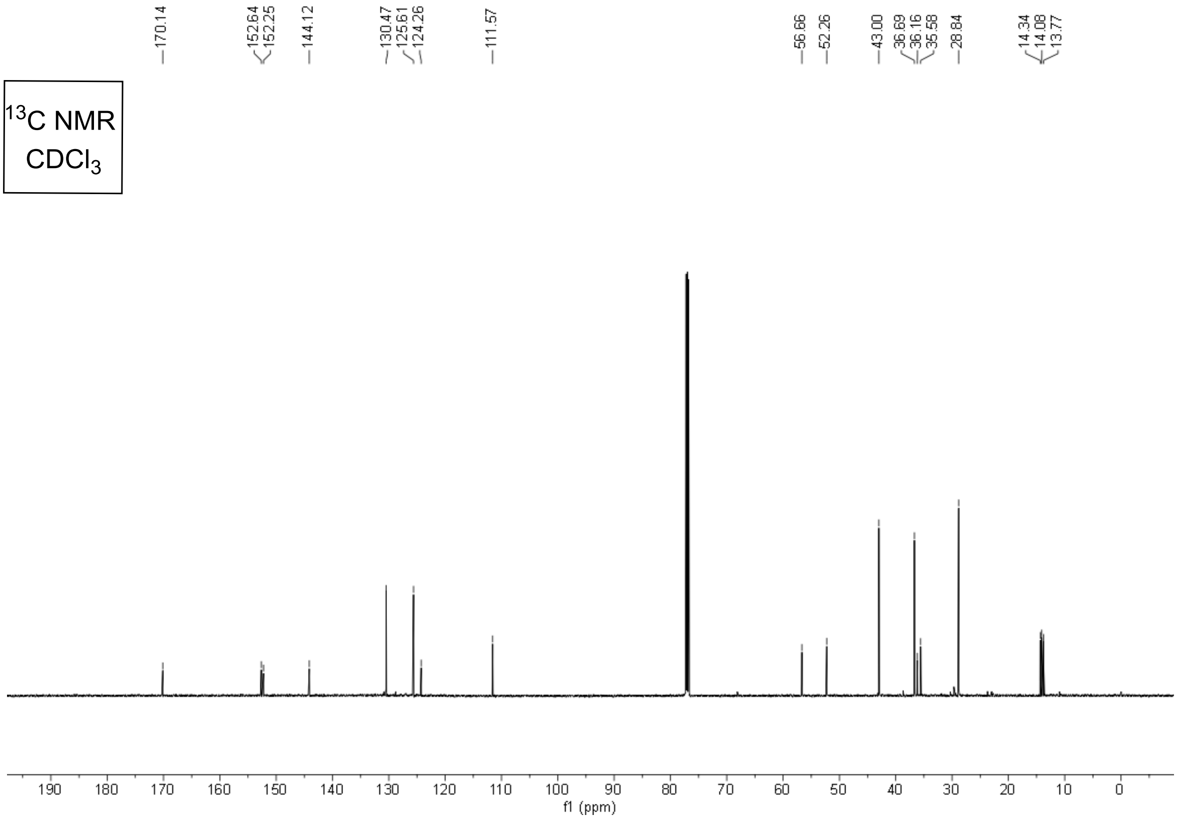

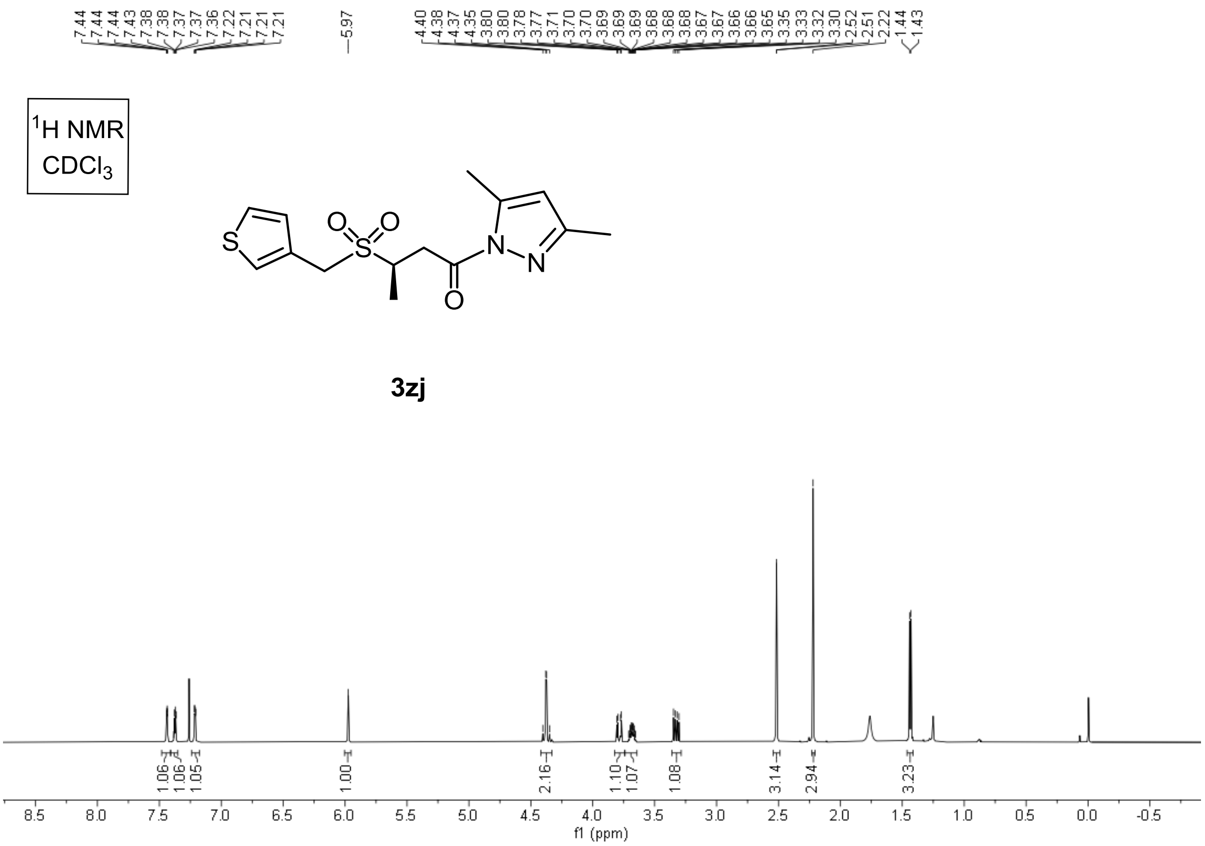
7.44 7.44 7.44 7.43 7.38 7.38 7.37 7.37 7.36 7.22 7.21 7.21 7.21 7.21
5.97
4.40 4.38 4.37 4.35 3.80 3.80 3.78 3.77 3.71 3.70 3.70 3.69 3.69 3.69 3.68 3.68 3.68 3.67 3.67 3.66 3.66 3.65 3.35 3.33 3.32 3.30 2.52 2.51 2.22 1.44 1.43
^{1}H NMR
$CDCl_3$
S
O
O
S
N
N
O
1.06 1.06 1.05 1.00 2.16 1.10 1.07 1.08 3.14 2.94 3.23
8.5 8.0 7.5 7.0 6.5 6.0 5.5 5.0 4.5 4.0 3.5 3.0 2.5 2.0 1.5 1.0 0.5 0.0 -0.5
f1 (ppm)

3zj

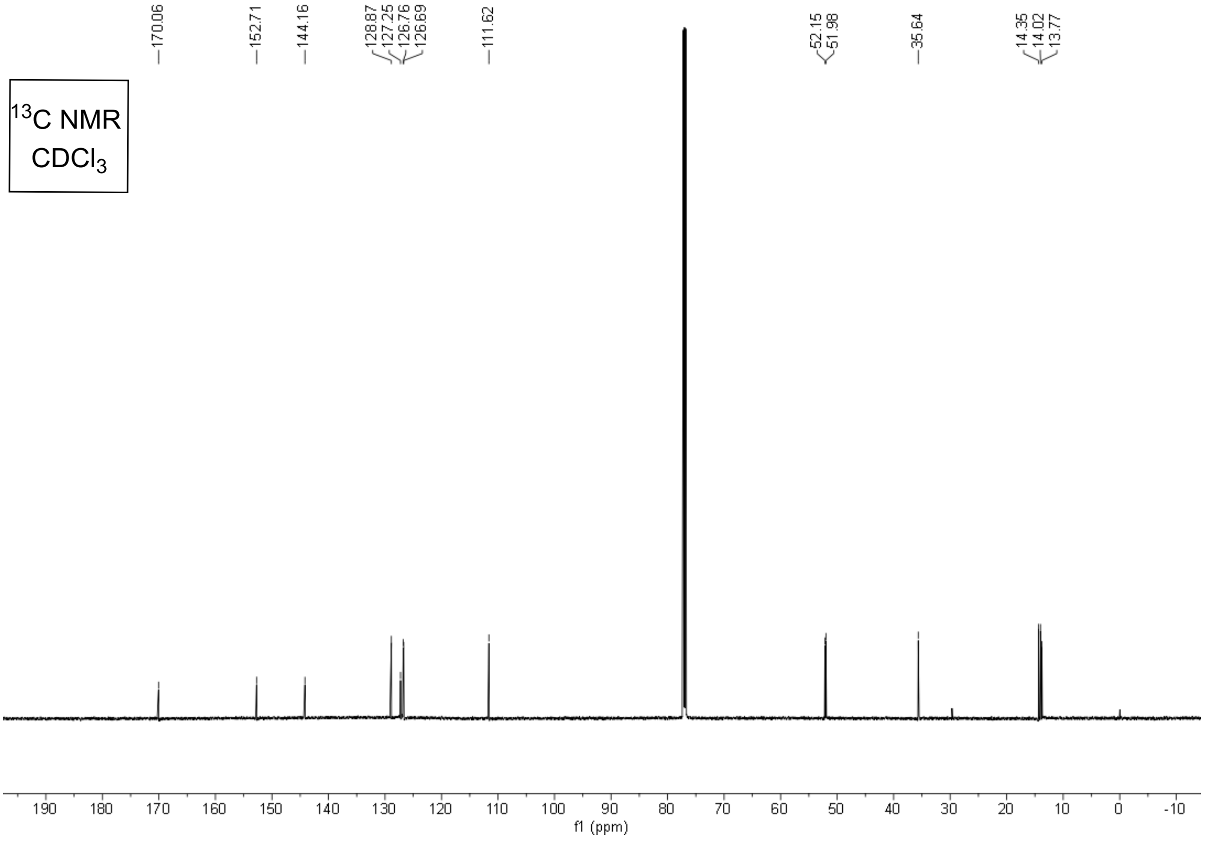
170.06
152.71
144.16
128.87 127.25 126.76 126.69
111.62
52.15 51.98
35.64
14.35 14.02 13.77
^{13}C NMR
$CDCl_3$
190 180 170 160 150 140 130 120 110 100 90 80 70 60 50 40 30 20 10 0 -10
f1 (ppm)

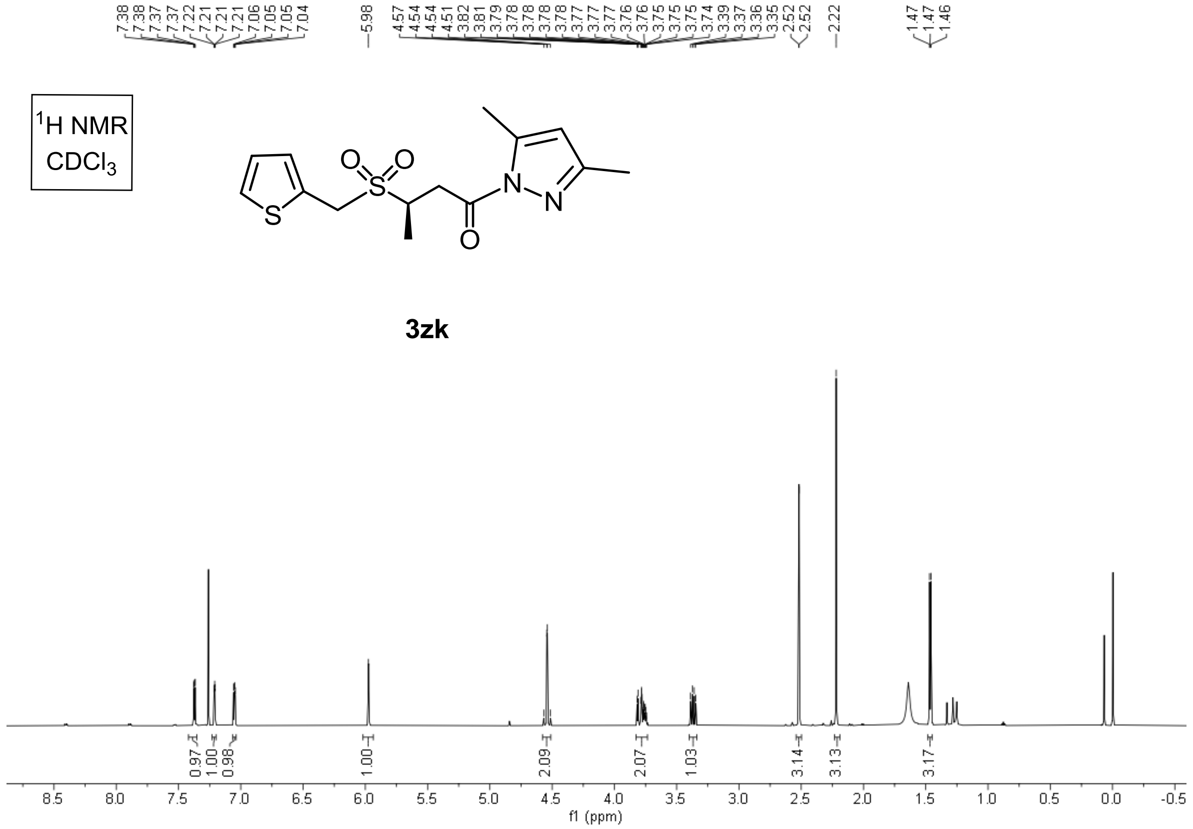
7.38 7.38 7.37 7.37 7.22 7.21 7.21 7.21 7.06 7.05 7.05 7.04 5.98 4.57 4.54 4.54 4.51 3.82 3.81 3.79 3.78 3.78 3.78 3.77 3.77 3.76 3.76 3.75 3.75 3.75 3.74 3.39 3.37 3.36 3.35 2.52 2.52 2.22 1.47 1.47 1.46
^{1}H NMR
$CDCl_3$
3zk
0.97 1.00 0.98 1.00 2.09 2.07 1.03 3.14 3.13 3.17
f1 (ppm)

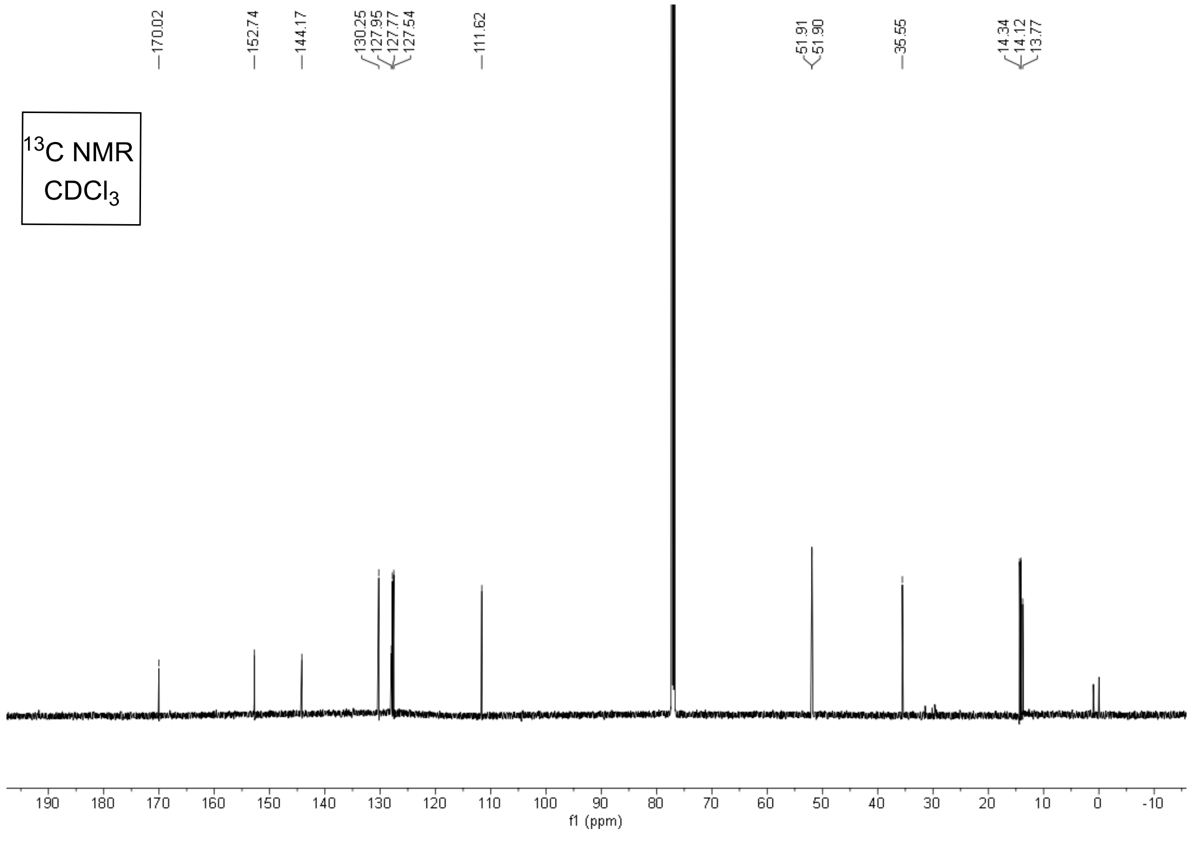
170.02 152.74 144.17 130.25 127.95 127.77 127.54 111.62 51.91 51.90 35.55 14.34 14.12 13.77
^{13}C NMR
$CDCl_3$
f1 (ppm)

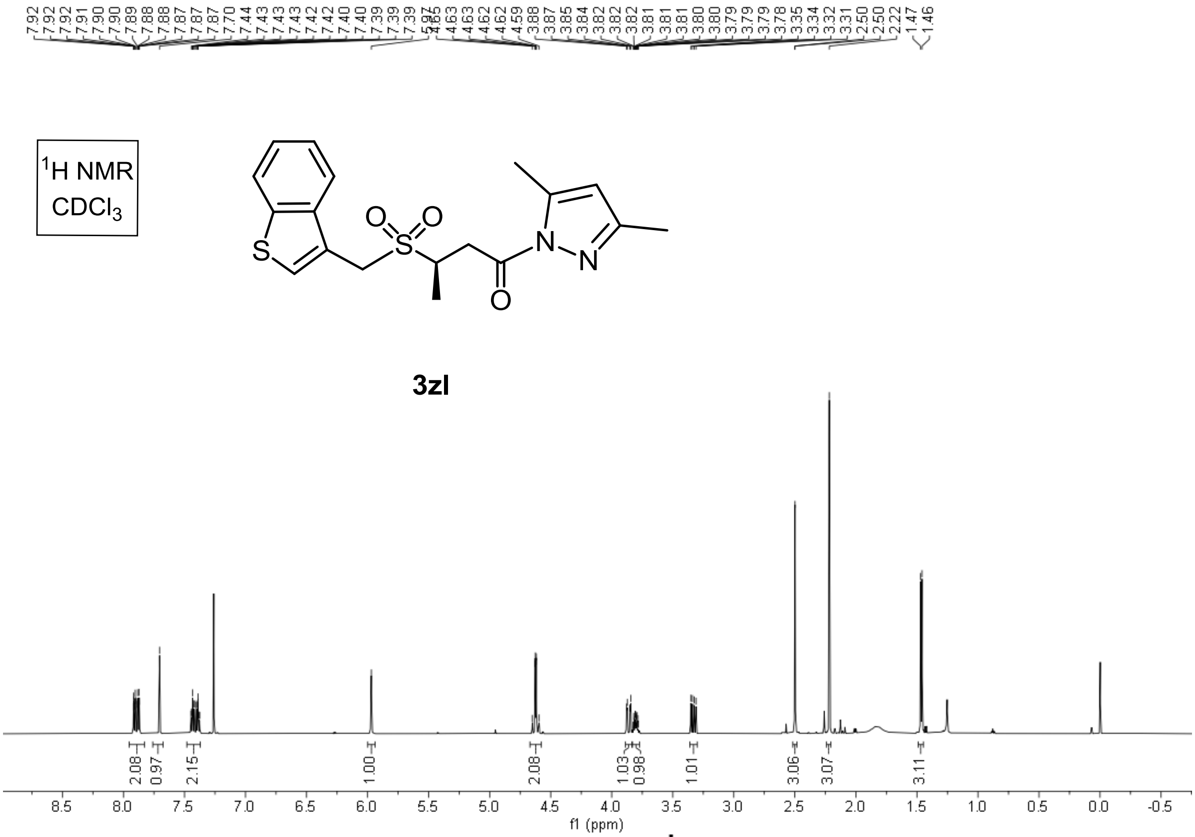
7.92 7.92 7.91 7.90 7.89 7.88 7.87 7.87 7.70 7.44 7.43 7.43 7.42 7.42 7.40 7.39 7.39 7.39 5.97 4.65 4.63 4.63 4.62 4.62 4.59 3.88 3.87 3.85 3.84 3.82 3.82 3.82 3.81 3.81 3.81 3.80 3.80 3.79 3.79 3.79 3.78 3.35 3.34 3.32 3.31 2.50 2.50 2.22 1.47 1.46
^{1}H NMR
CDCl$_3$
S
O
N
3zl
2.08 0.97 2.15 1.00 2.08 1.03 0.98 1.01 3.06 3.07 3.11
8.5 8.0 7.5 7.0 6.5 6.0 5.5 5.0 4.5 4.0 3.5 3.0 2.5 2.0 1.5 1.0 0.5 0.0 -0.5
f1 (ppm)

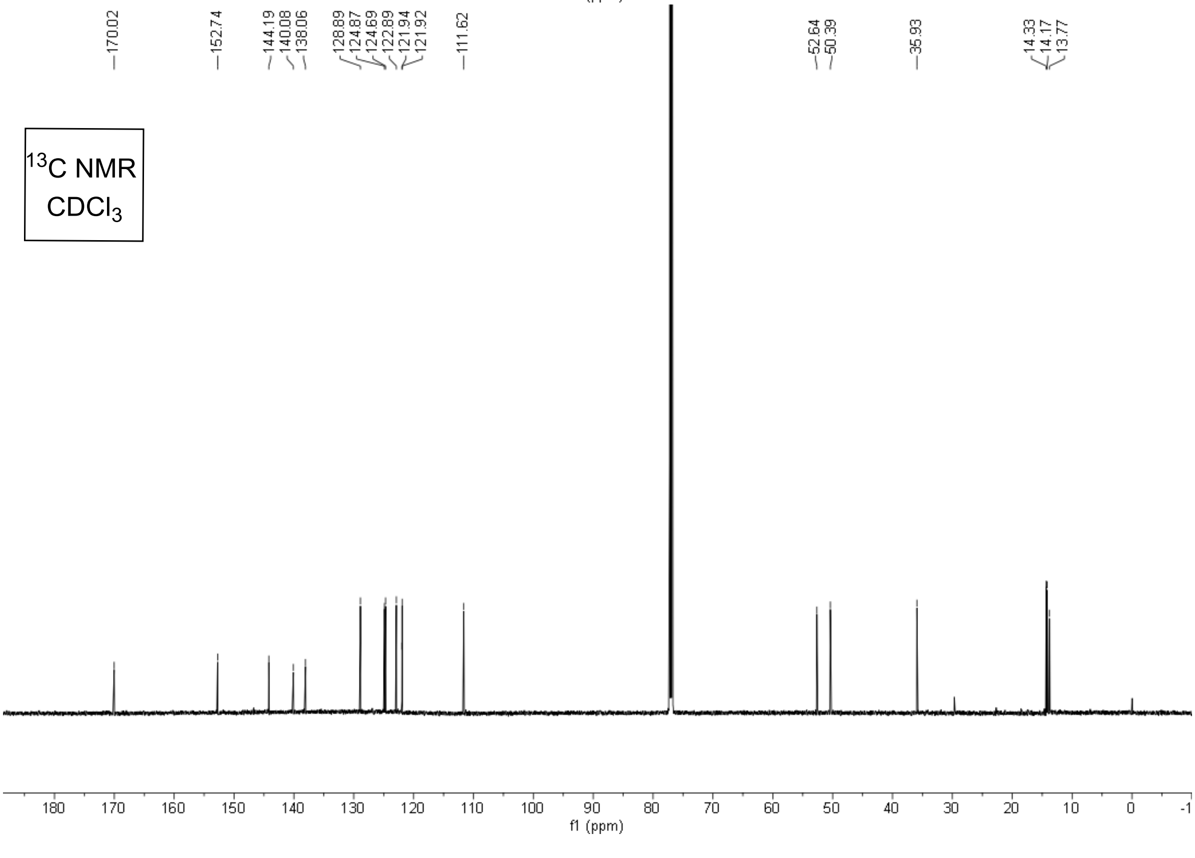
170.02 152.74 144.19 140.08 138.06 128.89 124.87 124.69 122.89 121.94 121.92 111.62 52.64 50.39 35.93 14.33 14.17 13.77
^{13}C NMR
CDCl$_3$
180 170 160 150 140 130 120 110 100 90 80 70 60 50 40 30 20 10 0 -1
f1 (ppm)

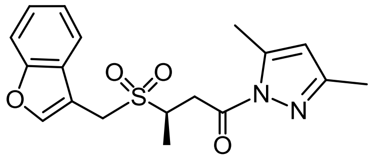

3zm

^{1}H NMR
$CDCl_3$

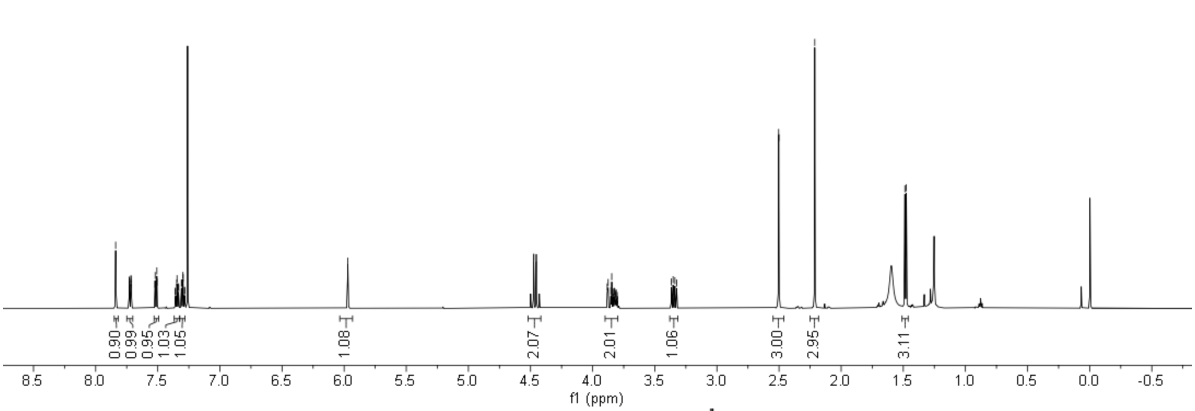

^{13}C NMR
$CDCl_3$

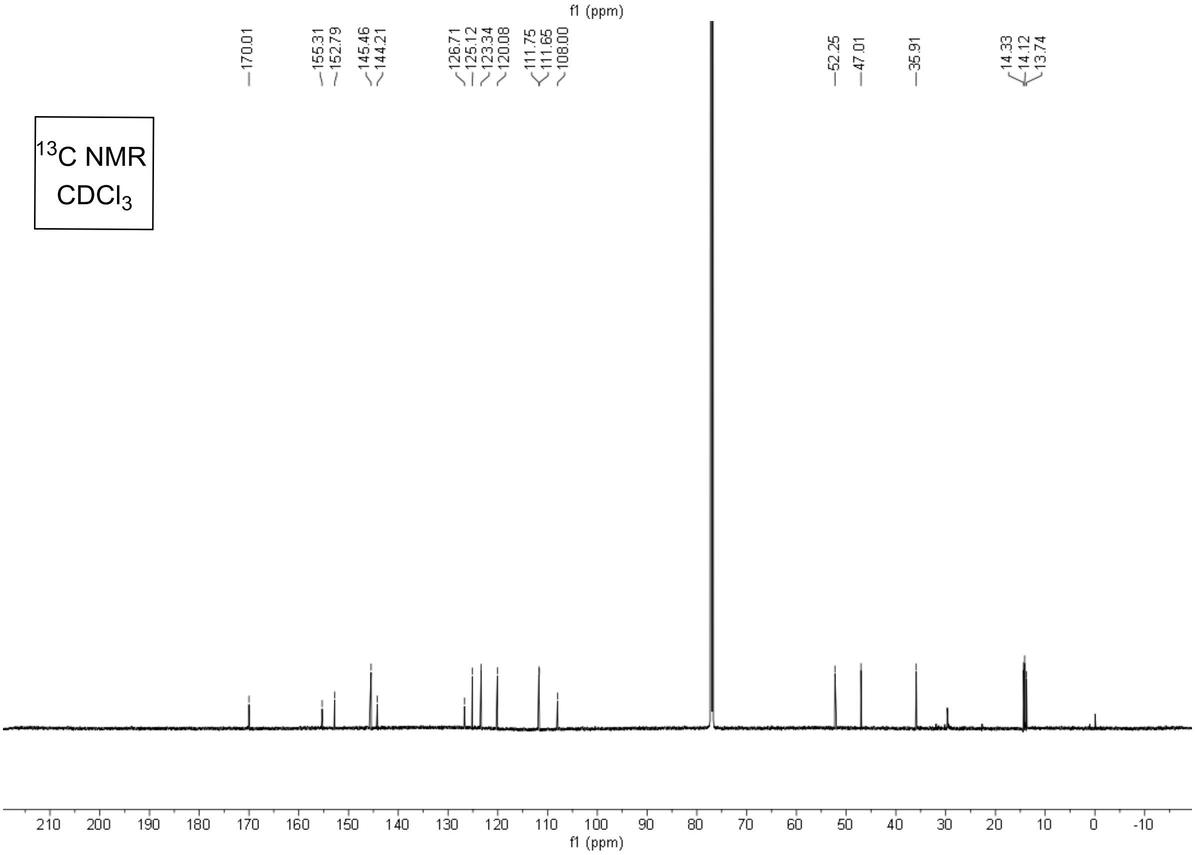

^{1}H NMR
$CDCl_3$

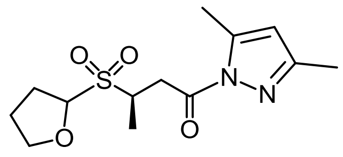

3zn

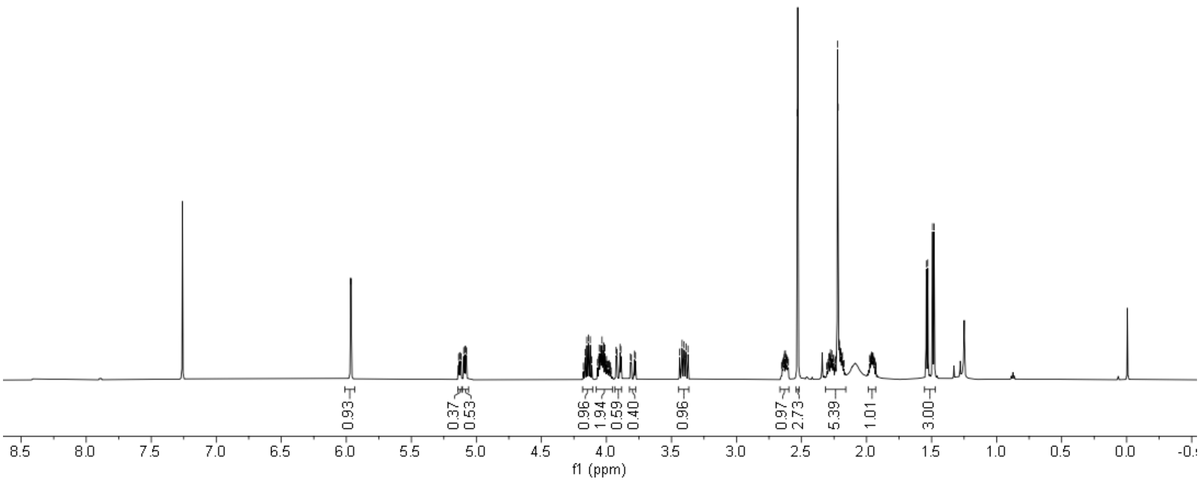

^{13}C NMR
$CDCl_3$

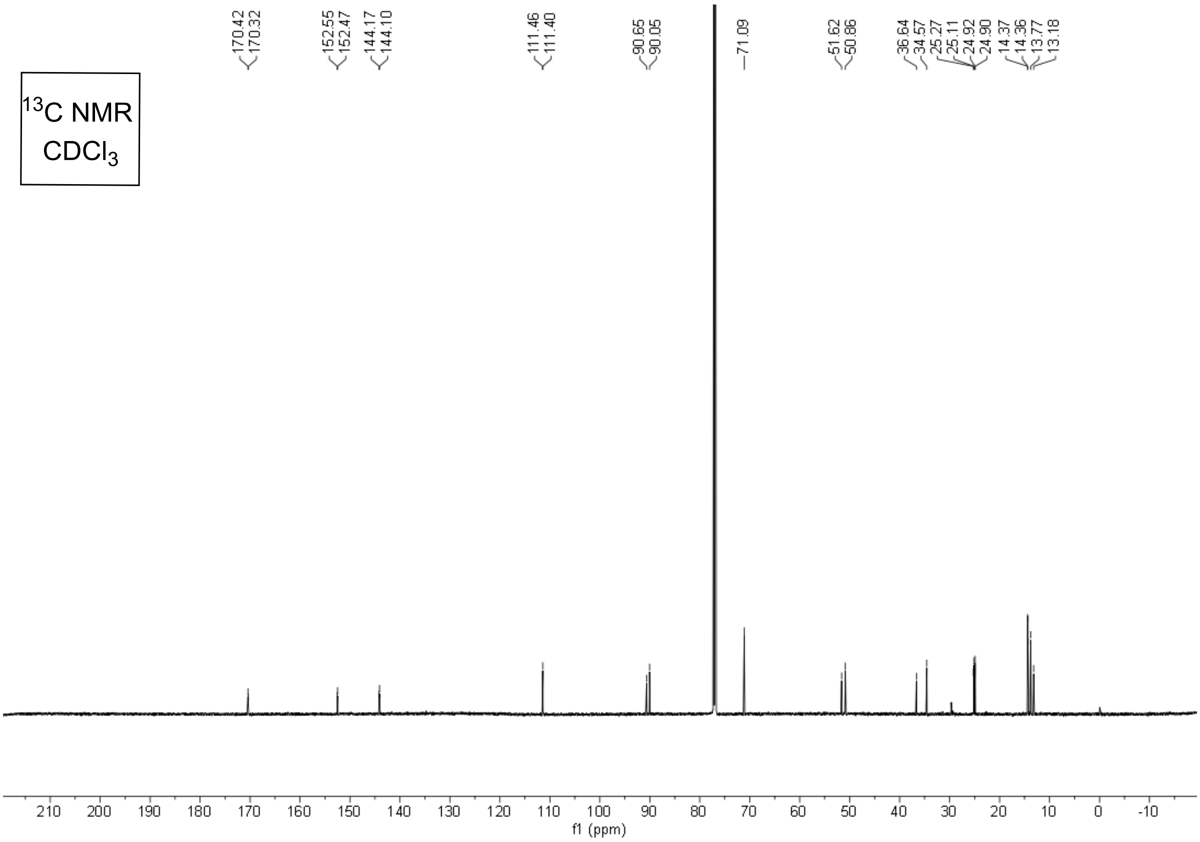

^{1}H NMR
$CDCl_3$

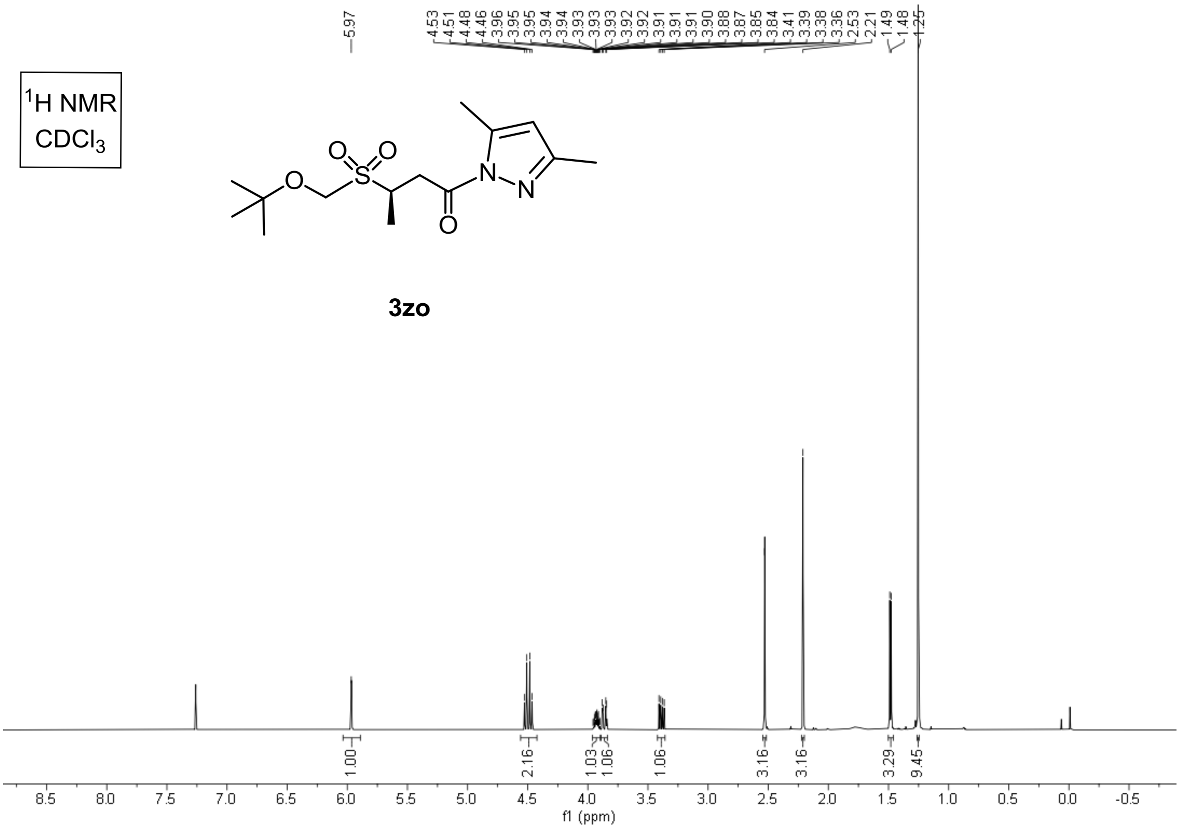

3zo

^{13}C NMR
$CDCl_3$

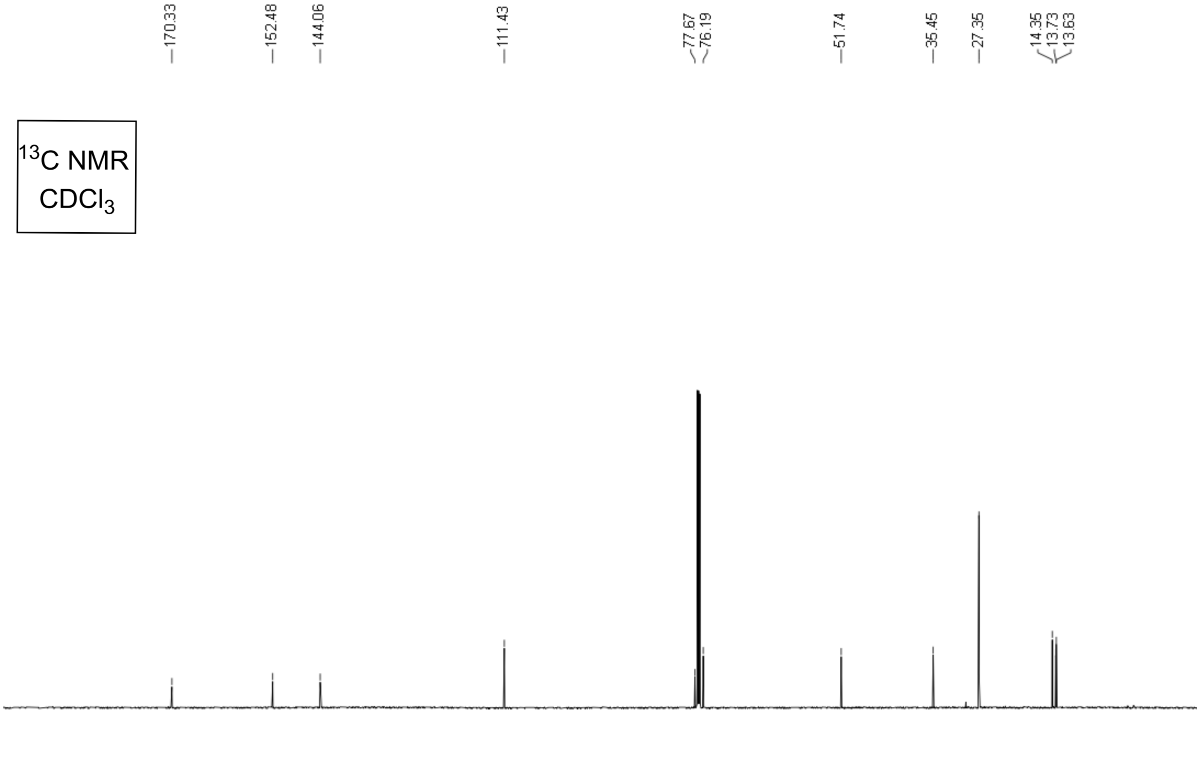

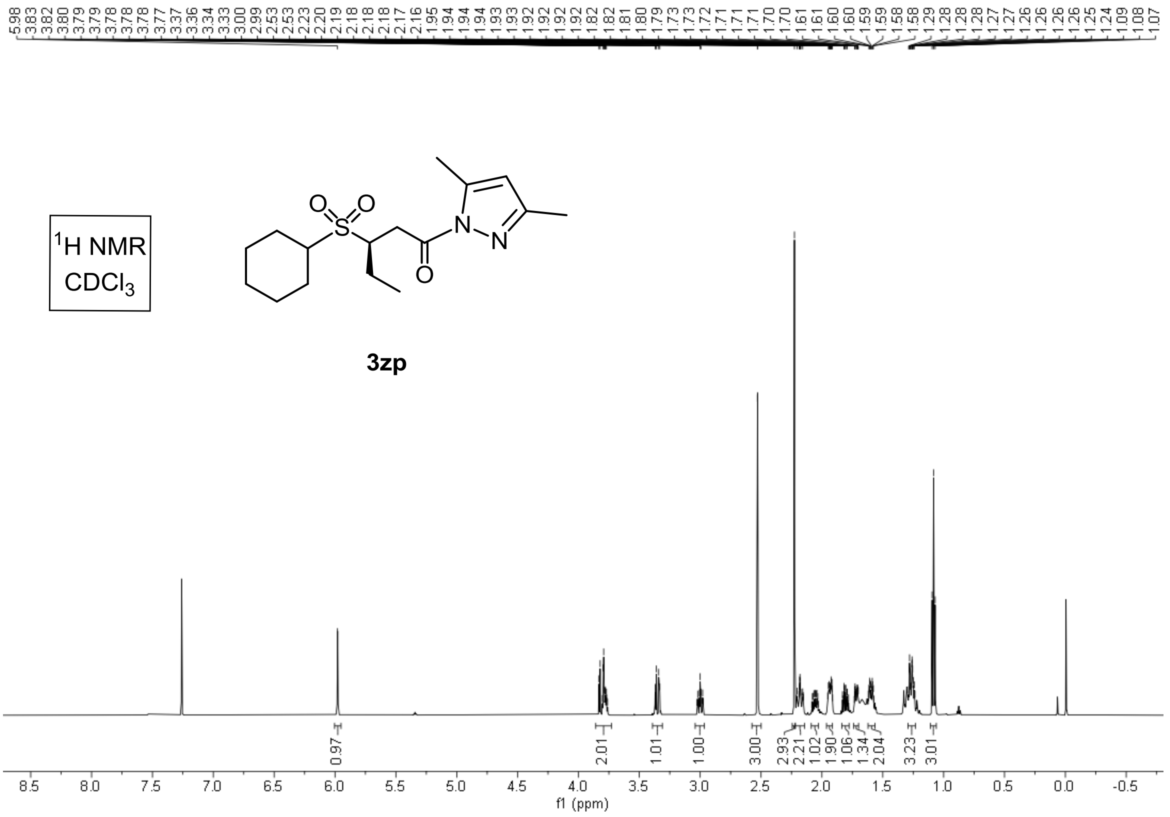

^{1}H NMR
$CDCl_3$
3zp
5.98 3.83 3.82 3.80 3.79 3.79 3.78 3.78 3.78 3.77 3.37 3.36 3.34 3.33 3.00 2.99 2.53 2.53 2.23 2.20 2.19 2.18 2.18 2.17 2.16 1.95 1.94 1.94 1.94 1.93 1.93 1.92 1.92 1.92 1.92 1.82 1.82 1.81 1.80 1.79 1.73 1.73 1.72 1.71 1.71 1.71 1.70 1.70 1.61 1.61 1.60 1.60 1.59 1.59 1.58 1.58 1.29 1.28 1.28 1.28 1.27 1.26 1.26 1.26 1.25 1.24 1.09 1.08 1.07
0.97
2.01
1.01
1.00
3.00
2.93
2.21
1.02
1.90
1.06
1.34
2.04
3.23
3.01
8.5 8.0 7.5 7.0 6.5 6.0 5.5 5.0 4.5 4.0 3.5 3.0 2.5 2.0 1.5 1.0 0.5 0.0 -0.5
f1 (ppm)

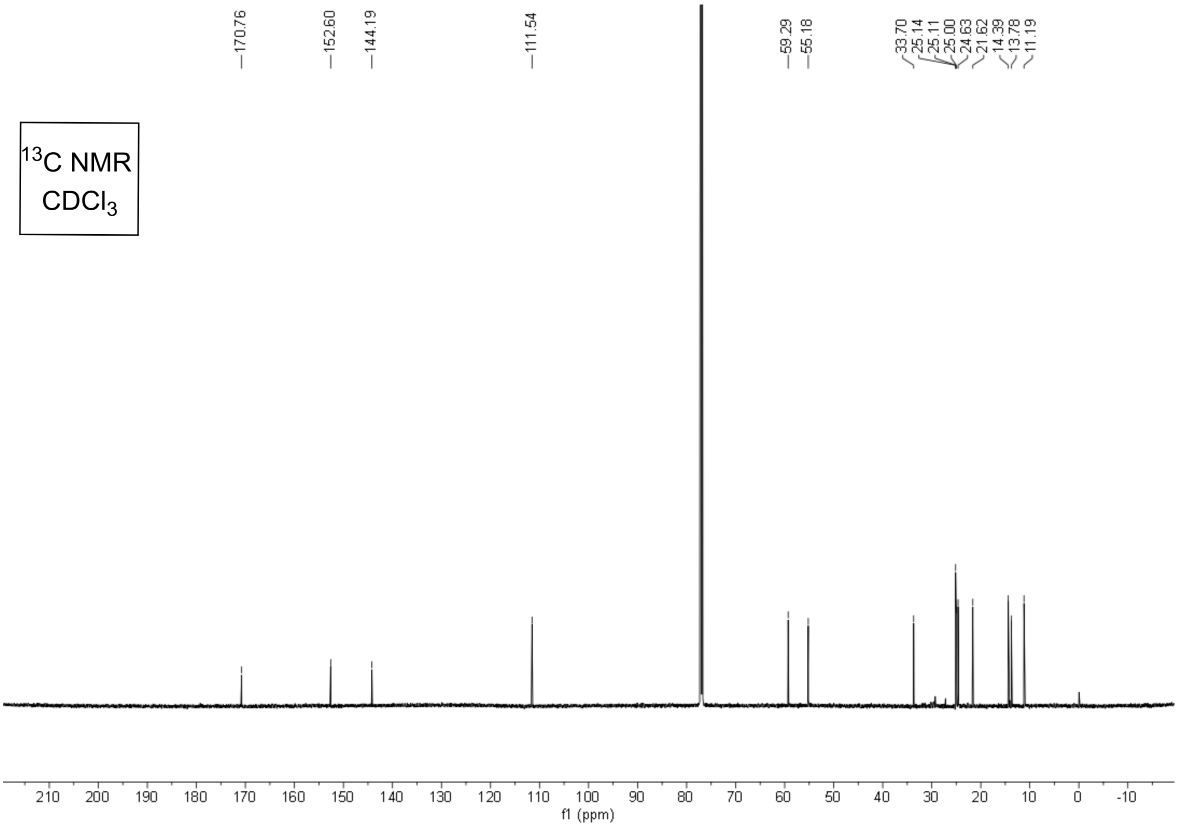

^{13}C NMR
$CDCl_3$
170.76
152.60
144.19
111.54
59.29
55.18
33.70
25.14
25.11
25.00
24.63
21.62
14.39
13.78
11.19
210 200 190 180 170 160 150 140 130 120 110 100 90 80 70 60 50 40 30 20 10 0 -10
f1 (ppm)

^{1}H NMR
CDCl$_3$

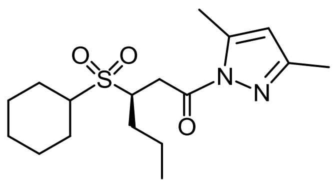

3zq

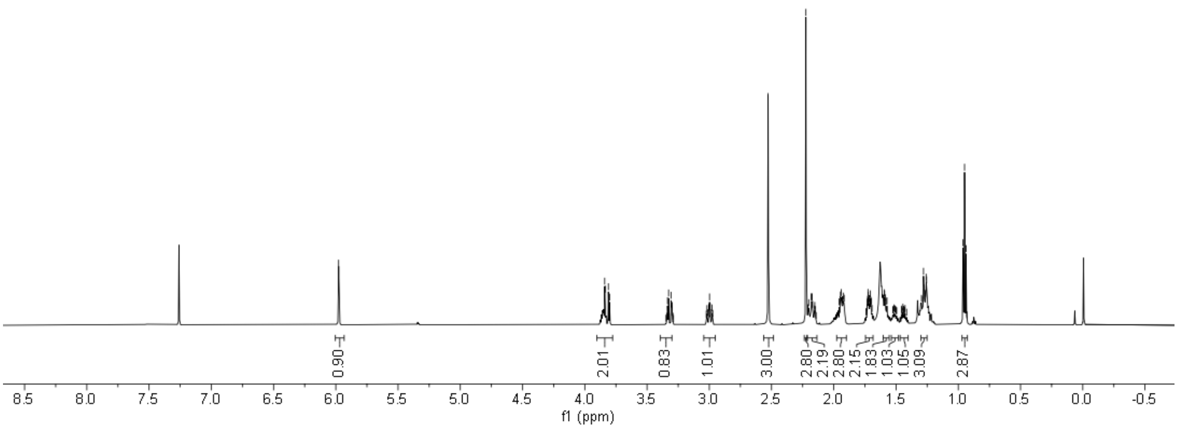

^{13}C NMR
CDCl$_3$

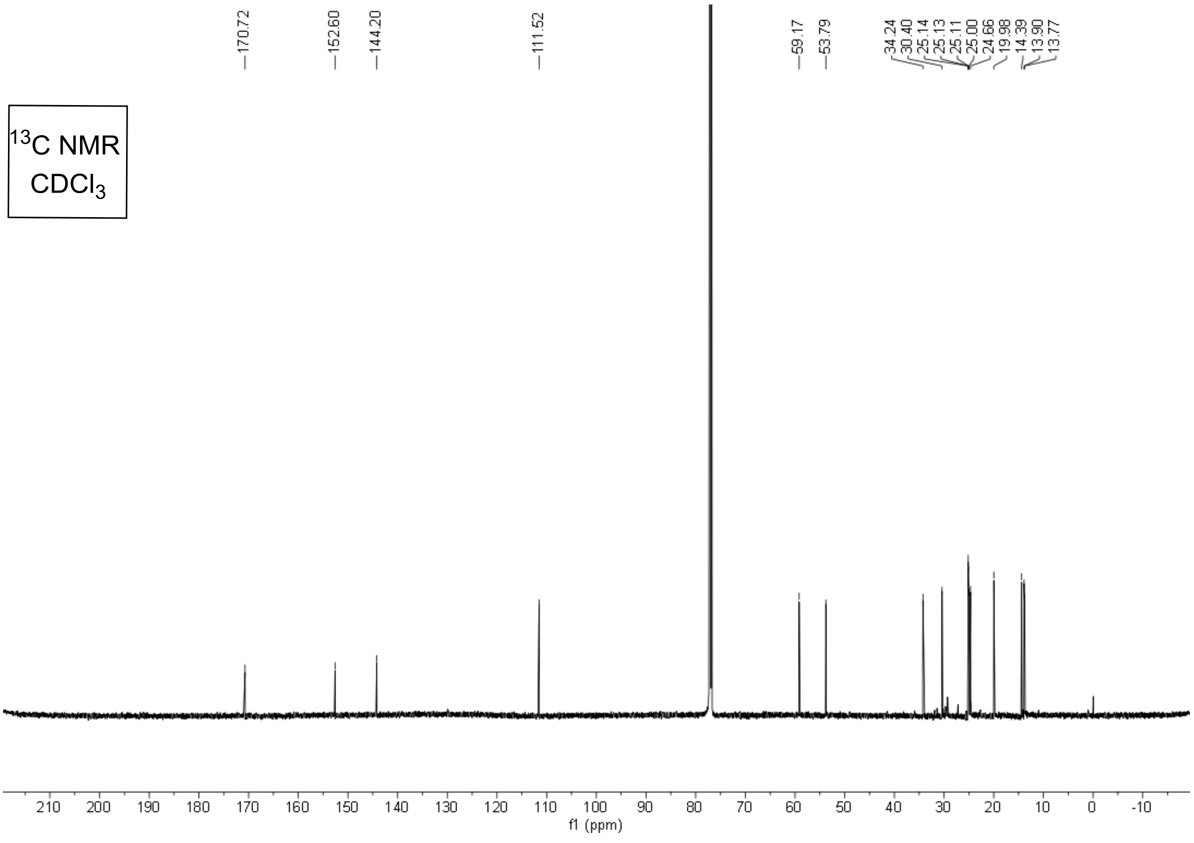

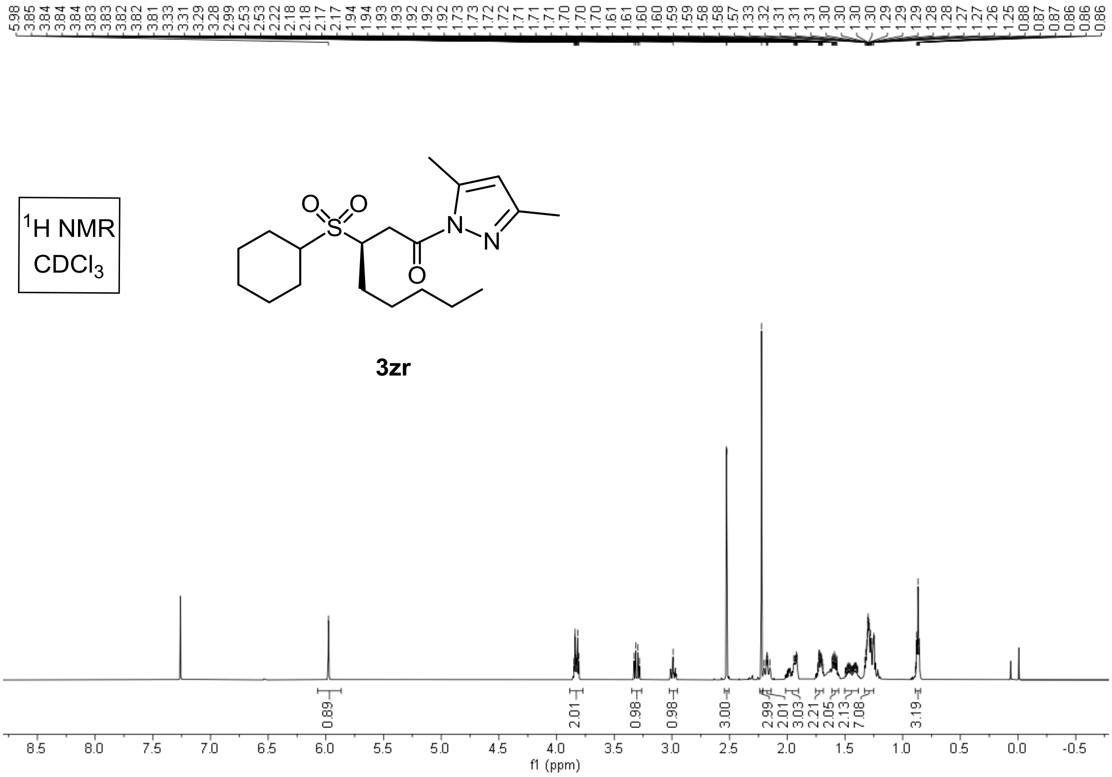
5.98
3.85
3.84
3.84
3.84
3.83
3.83
3.82
3.82
3.81
3.33
3.31
3.29
3.28
2.99
2.53
2.53
2.22
2.18
2.18
2.17
2.17
1.94
1.94
1.93
1.93
1.92
1.92
1.92
1.73
1.73
1.72
1.72
1.71
1.71
1.71
1.70
1.70
1.70
1.61
1.61
1.60
1.60
1.59
1.59
1.58
1.57
1.33
1.32
1.31
1.31
1.30
1.30
1.30
1.30
1.29
1.29
1.28
1.28
1.27
1.27
1.26
1.25
0.88
0.87
0.87
0.86
0.86
1H NMR
CDCl3
O
S
O
N
N
O
3zr
0.89
2.01
0.98
0.98
3.00
2.99
2.01
3.03
2.21
2.05
2.13
7.08
3.19
8.5 8.0 7.5 7.0 6.5 6.0 5.5 5.0 4.5 4.0 3.5 3.0 2.5 2.0 1.5 1.0 0.5 0.0 -0.5
f1 (ppm)

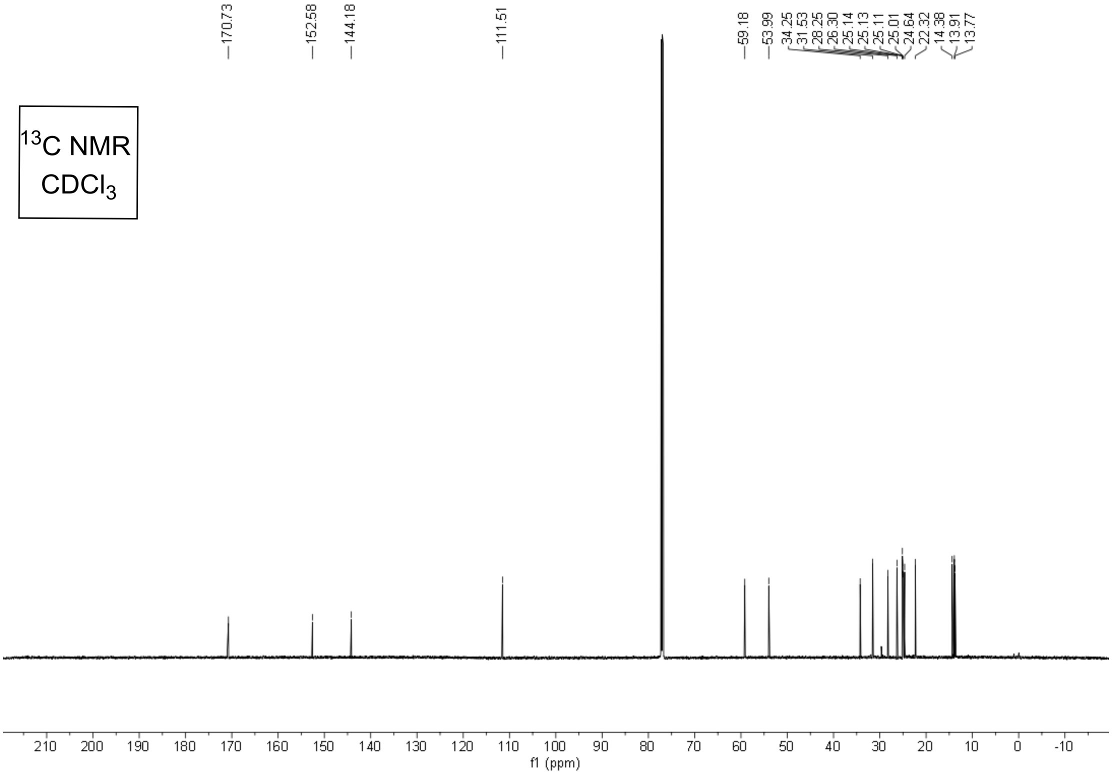
170.73
152.58
144.18
111.51
59.18
53.99
34.25
31.53
28.25
26.30
25.14
25.13
25.11
25.01
24.64
22.32
14.38
13.91
13.77
13C NMR
CDCl3
210 200 190 180 170 160 150 140 130 120 110 100 90 80 70 60 50 40 30 20 10 0 -10
f1 (ppm)

1H NMR
CDCl3

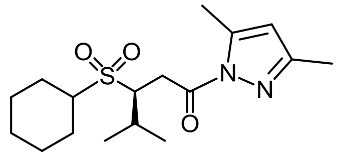

3zs

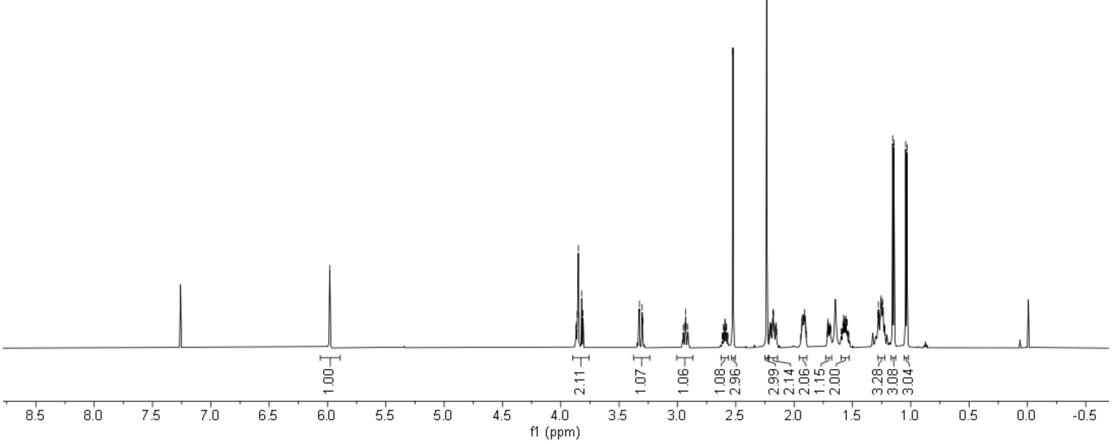

13C NMR
CDCl3

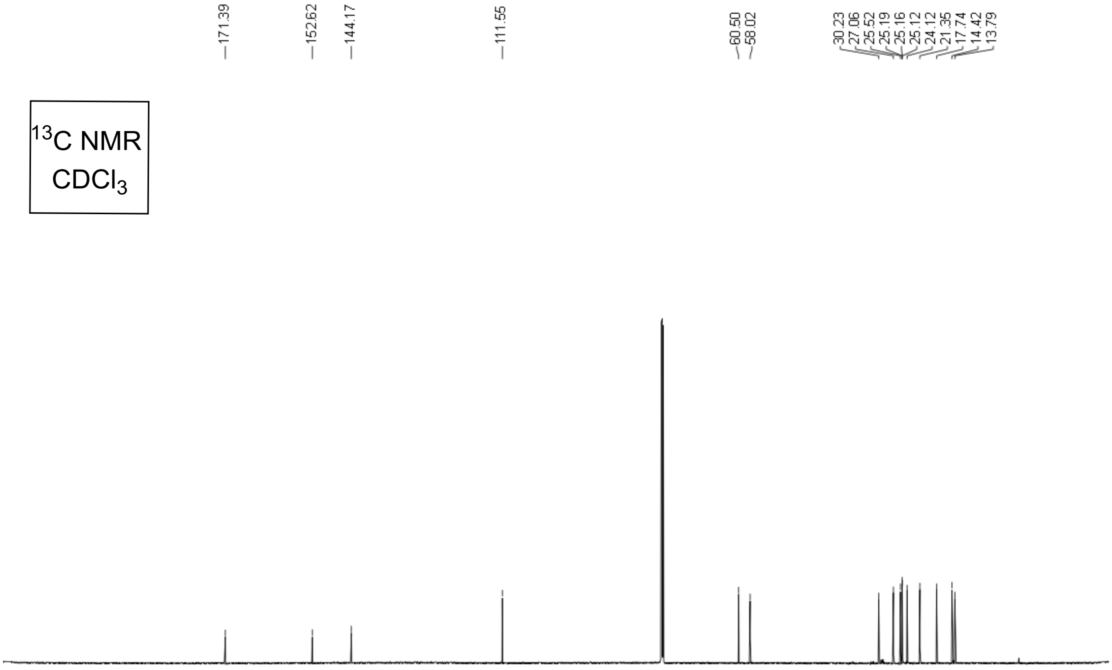

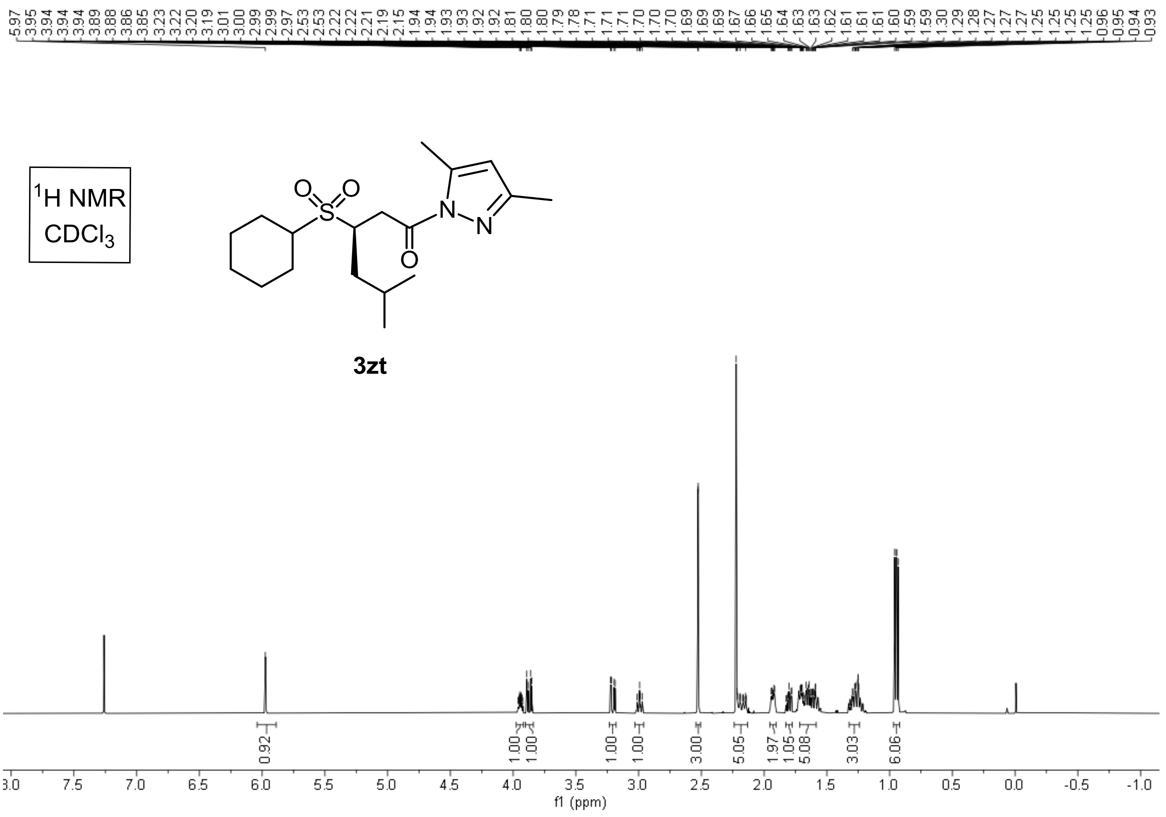
1H NMR
CDCl3
3zt

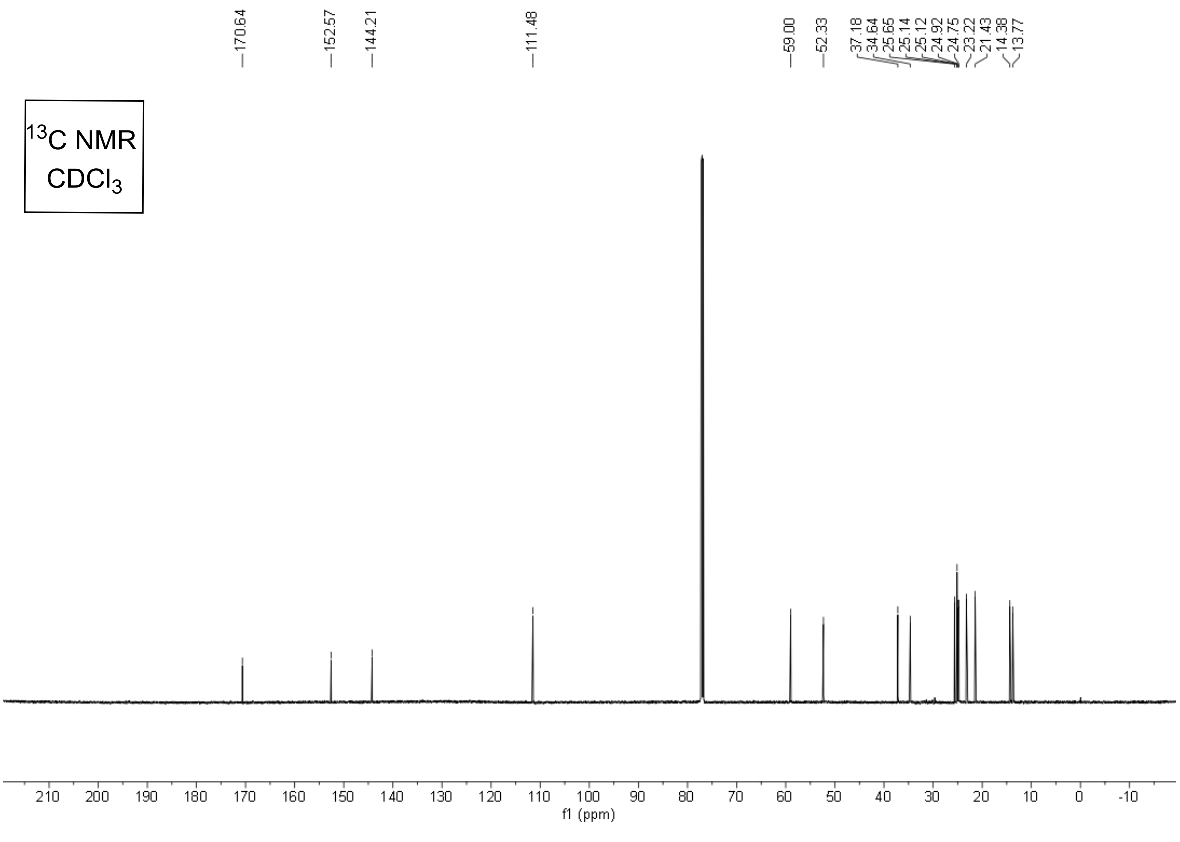
13C NMR
CDCl3

^{1}H NMR
CDCl$_3$

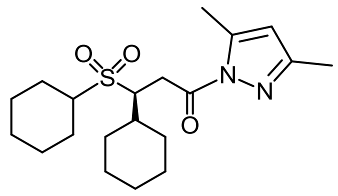

3zu

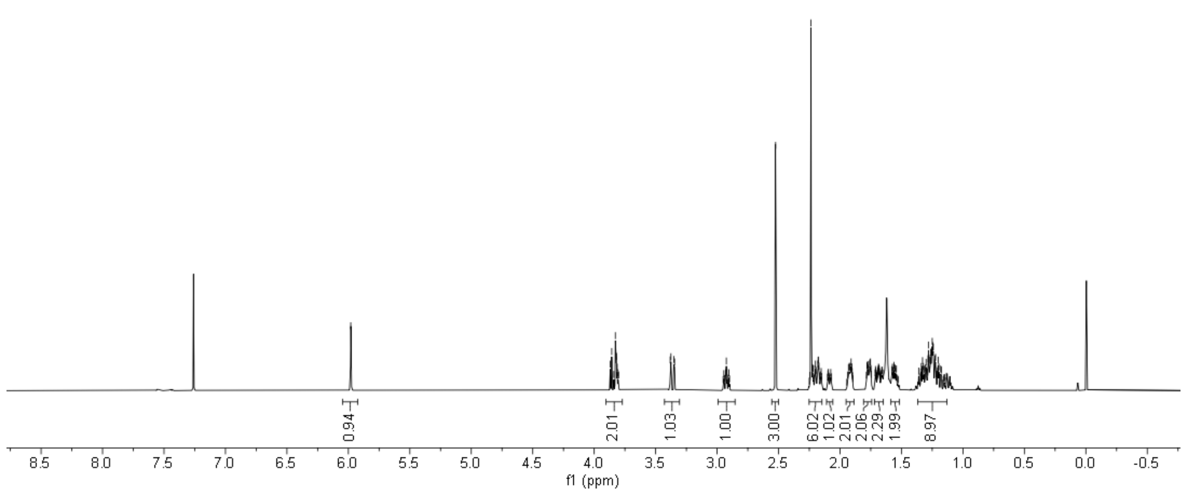

^{13}C NMR
CDCl$_3$

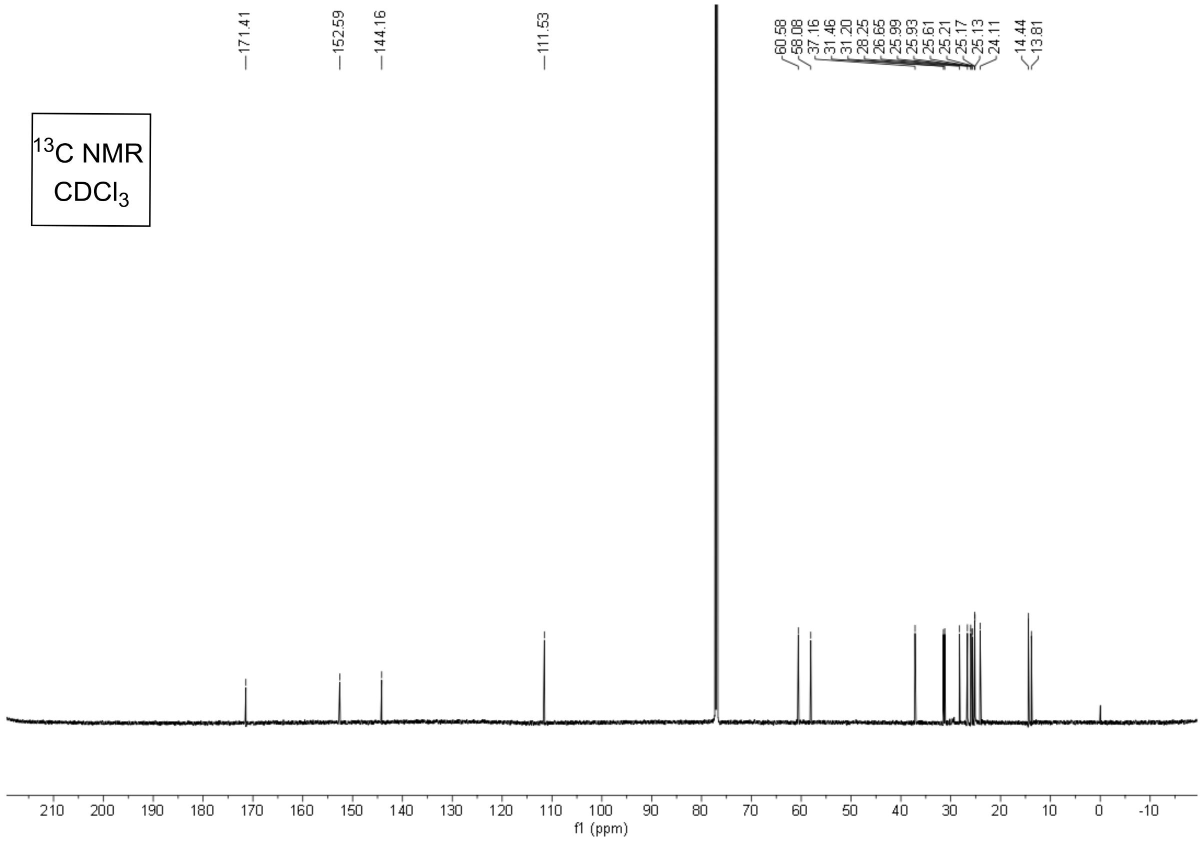

^{1}H NMR
$CDCl_3$

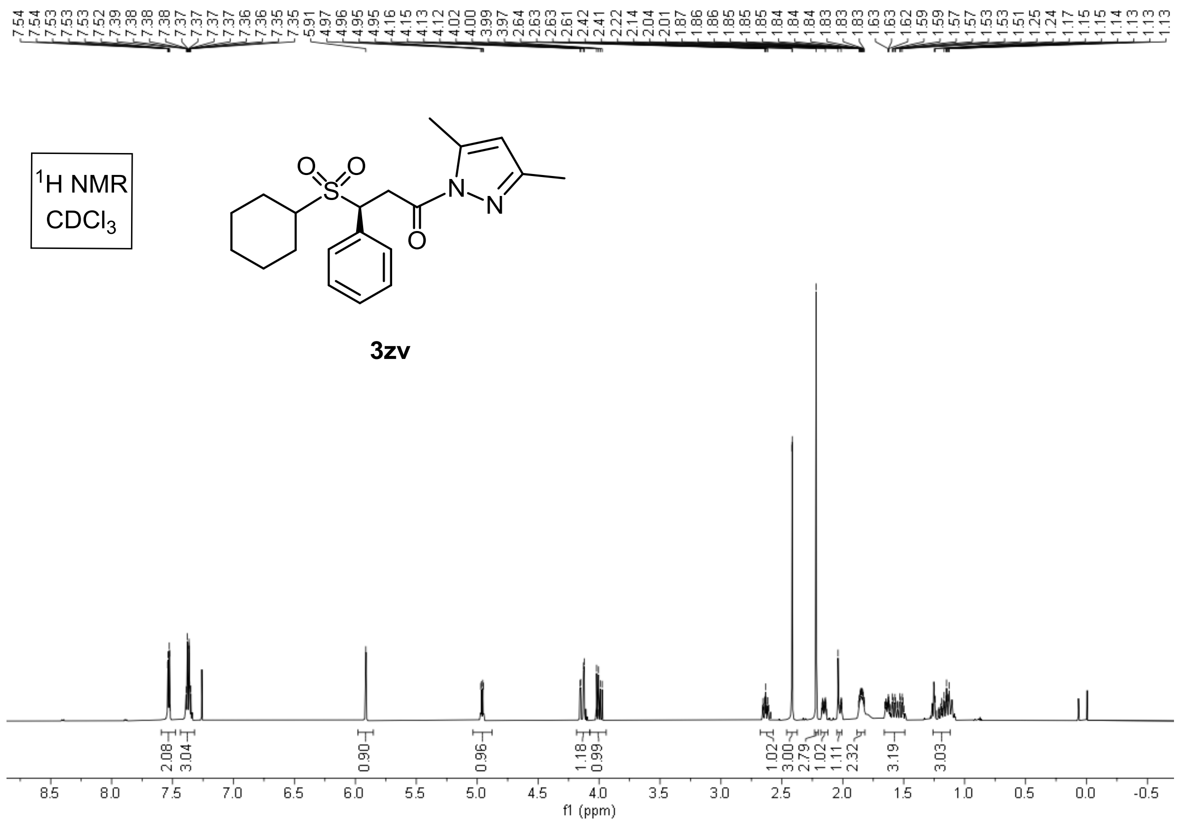

3zv

^{13}C NMR
$CDCl_3$

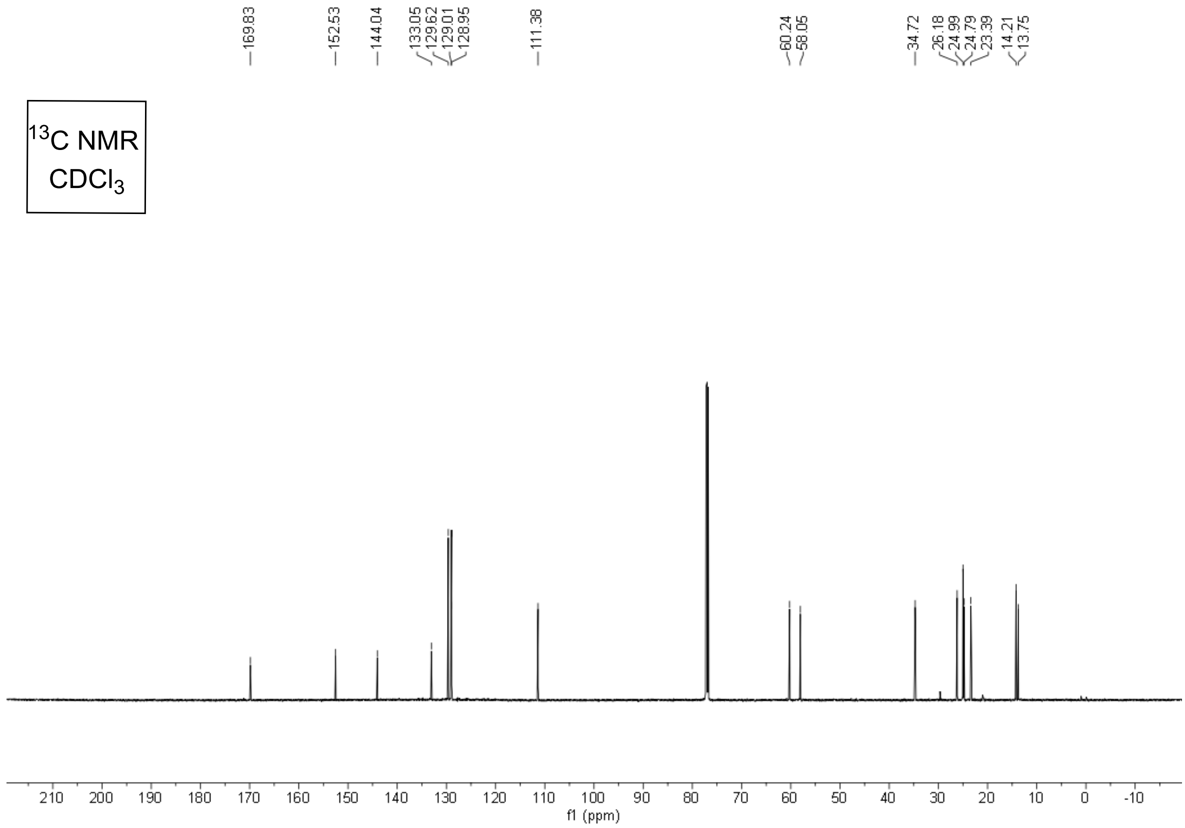

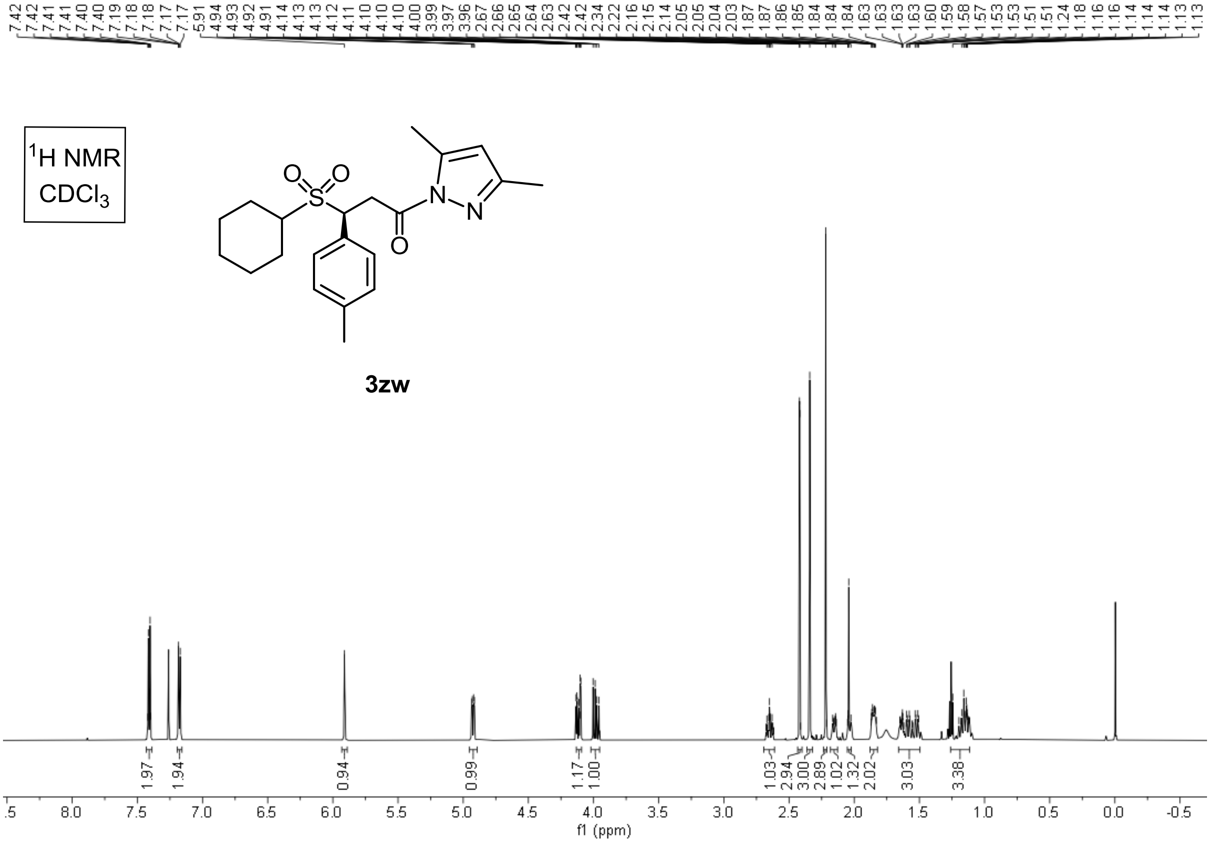
7.42 7.42 7.41 7.40 7.40 7.19 7.18 7.18 7.17 5.91 4.94 4.93 4.92 4.91 4.14 4.13 4.13 4.12 4.11 4.10 4.10 4.10 4.00 3.99 3.97 3.96 2.67 2.66 2.65 2.64 2.63 2.42 2.42 2.34 2.22 2.16 2.15 2.14 2.05 2.05 2.04 2.03 1.87 1.87 1.86 1.85 1.84 1.84 1.84 1.84 1.63 1.63 1.63 1.63 1.60 1.59 1.58 1.57 1.53 1.53 1.51 1.51 1.24 1.18 1.16 1.16 1.14 1.14 1.14 1.13 1.13
1H NMR
CDCl3
3zw
1.97 1.94 0.94 0.99 1.17 1.00 1.03 2.94 3.00 2.89 1.02 1.32 2.02 3.03 3.38
f1 (ppm)
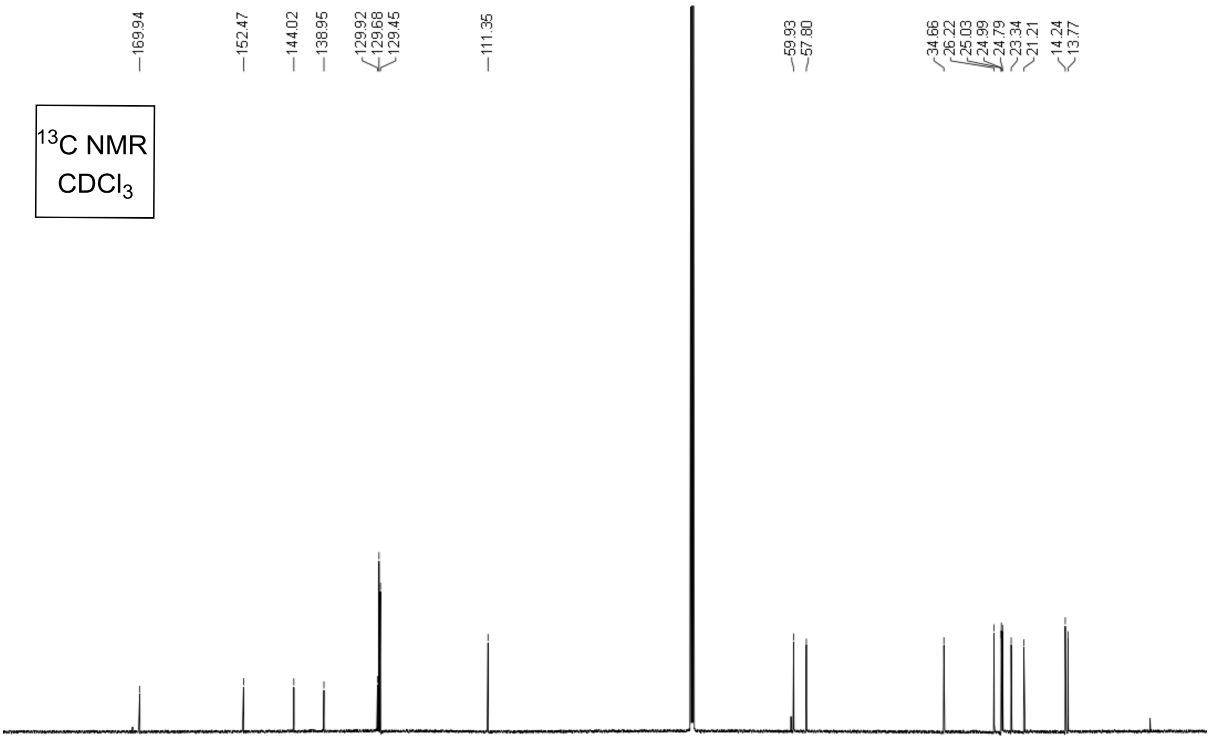
169.94 152.47 144.02 138.95 129.92 129.68 129.45 111.35 59.93 57.80 34.66 26.22 25.03 24.99 24.79 23.34 21.21 14.24 13.77
13C NMR
CDCl3
f1 (ppm)

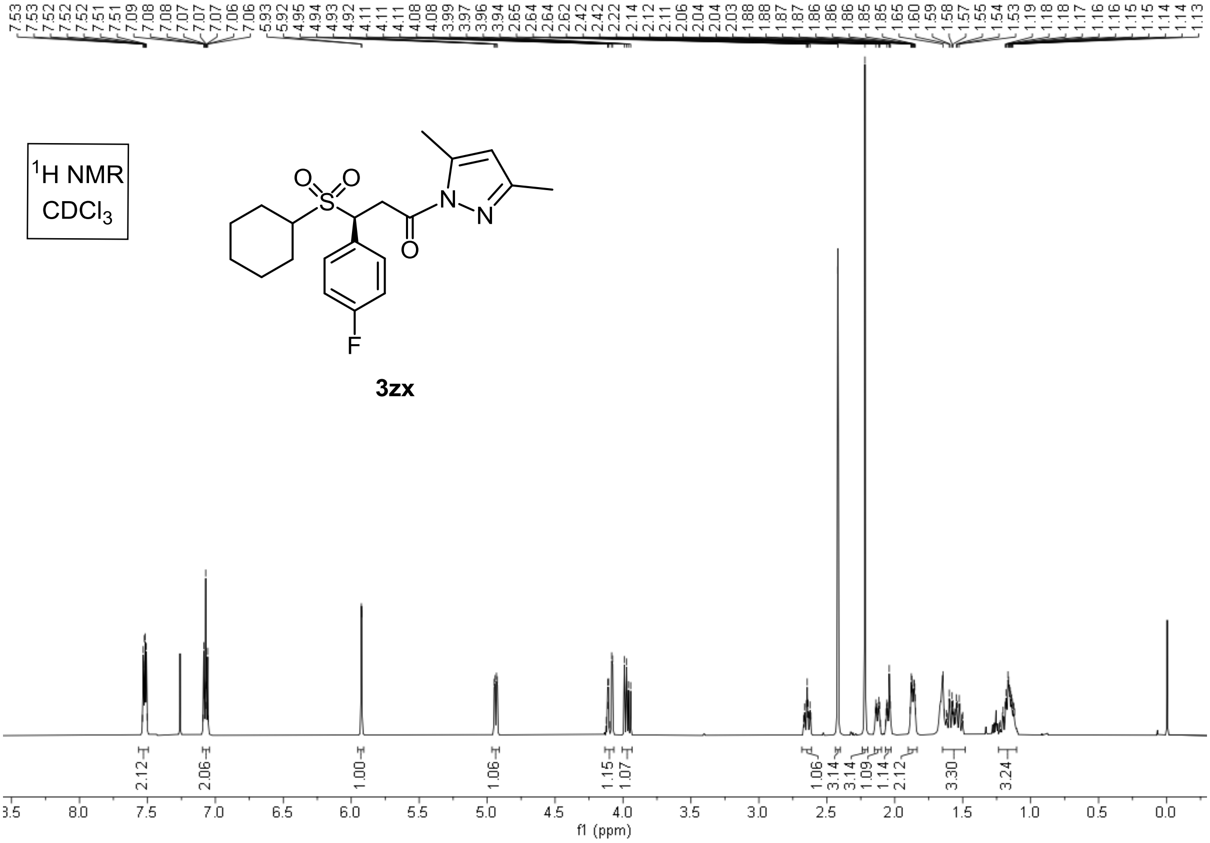
7.53 7.53 7.52 7.52 7.52 7.51 7.51 7.09 7.08 7.08 7.07 7.07 7.07 7.06 7.06 5.93 5.92 4.95 4.94 4.93 4.93 4.92 4.11 4.11 4.11 4.08 4.08 3.99 3.97 3.96 3.94 2.65 2.64 2.64 2.62 2.42 2.42 2.22 2.14 2.12 2.11 2.06 2.04 2.04 2.03 1.88 1.88 1.87 1.87 1.86 1.86 1.86 1.85 1.85 1.65 1.60 1.59 1.58 1.57 1.55 1.54 1.53 1.19 1.18 1.18 1.17 1.16 1.16 1.15 1.15 1.14 1.14 1.13
^{1}H NMR
$CDCl_3$
O O
S
O
N
N
F
3zx
2.12 2.06 1.00 1.06 1.15 1.07 1.06 3.14 3.14 1.09 1.14 2.12 3.30 3.24
8.5 8.0 7.5 7.0 6.5 6.0 5.5 5.0 4.5 4.0 3.5 3.0 2.5 2.0 1.5 1.0 0.5 0.0
f1 (ppm)

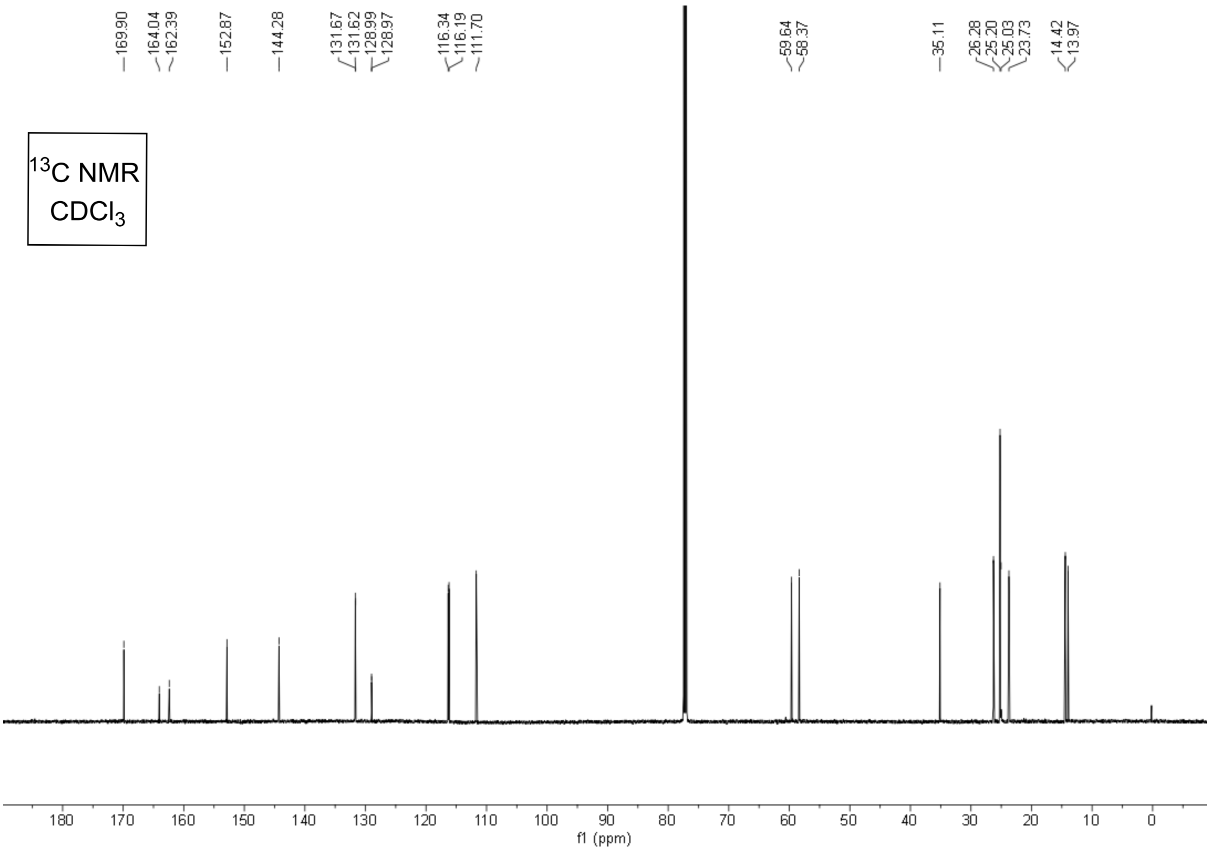
169.90 164.04 162.39 152.87 144.28 131.67 131.62 128.99 128.97 116.34 116.19 111.70 59.64 58.37 35.11 26.28 25.20 25.03 23.73 14.42 13.97
^{13}C NMR
$CDCl_3$
180 170 160 150 140 130 120 110 100 90 80 70 60 50 40 30 20 10 0
f1 (ppm)

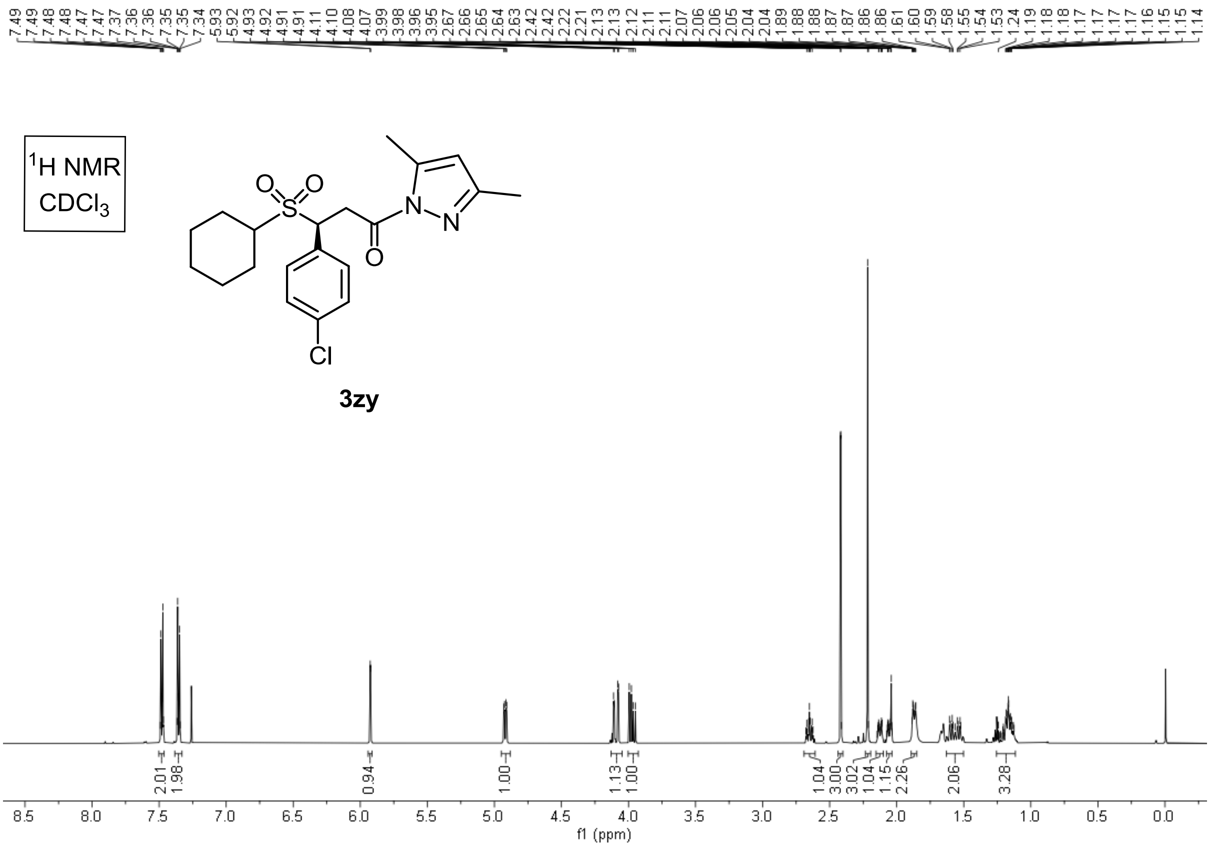
7.49 7.49 7.48 7.48 7.47 7.47 7.37 7.36 7.36 7.35 7.35 7.34 5.93 5.92 4.93 4.92 4.91 4.91 4.11 4.10 4.08 4.07 3.99 3.98 3.96 3.95 2.67 2.66 2.65 2.64 2.63 2.42 2.42 2.22 2.21 2.13 2.13 2.12 2.11 2.11 2.07 2.06 2.06 2.05 2.04 2.04 1.89 1.88 1.88 1.87 1.87 1.86 1.86 1.61 1.60 1.59 1.58 1.55 1.54 1.53 1.24 1.19 1.18 1.18 1.17 1.17 1.17 1.17 1.16 1.15 1.15 1.14
1H NMR
CDCl3
O
O
S
O
N
N
O
Cl
3zy
2.01
1.98
0.94
1.00
1.13
1.00
1.04
3.00
3.02
1.04
1.15
2.26
2.06
3.28
8.5 8.0 7.5 7.0 6.5 6.0 5.5 5.0 4.5 4.0 3.5 3.0 2.5 2.0 1.5 1.0 0.5 0.0
f1 (ppm)

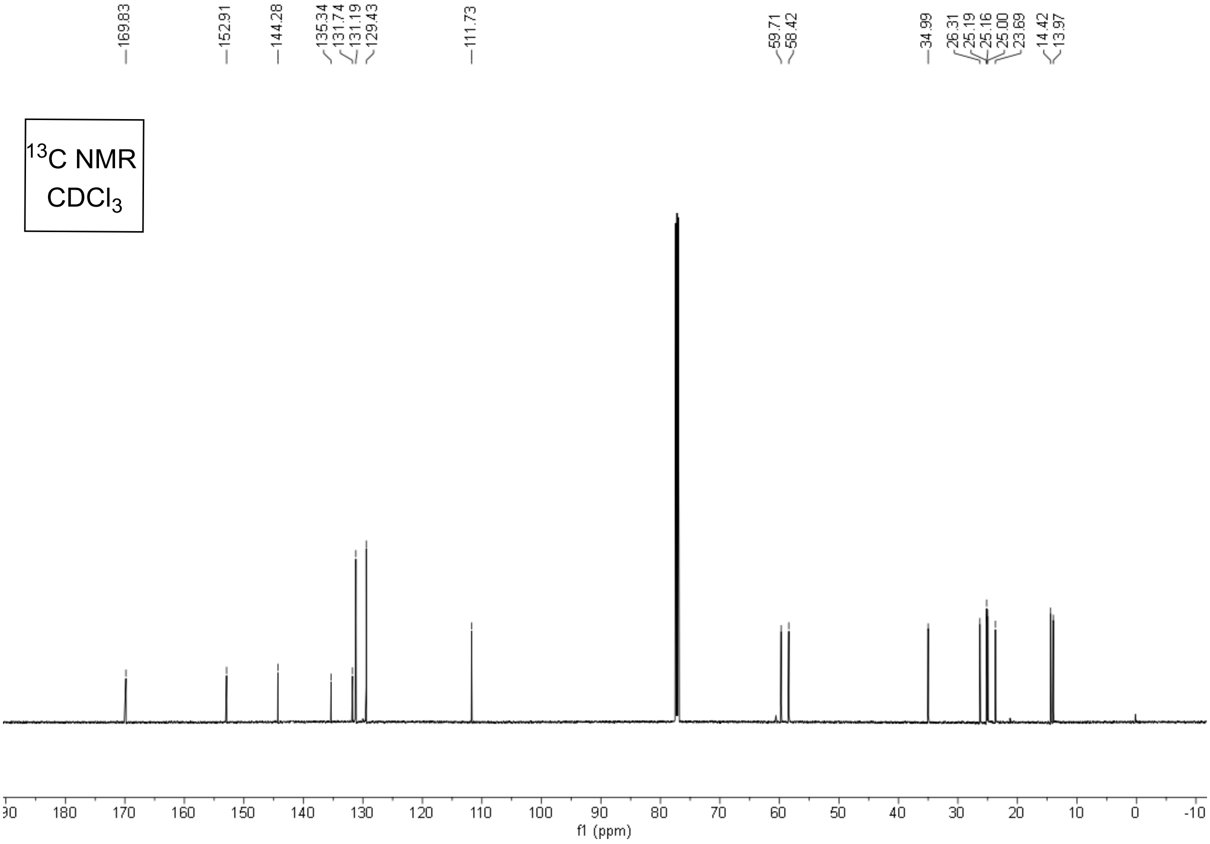
169.83 152.91 144.28 135.34 131.74 131.19 129.43 111.73 59.71 58.42 34.99 26.31 25.19 25.16 25.00 23.69 14.42 13.97
13C NMR
CDCl3
180 170 160 150 140 130 120 110 100 90 80 70 60 50 40 30 20 10 0 -10
f1 (ppm)

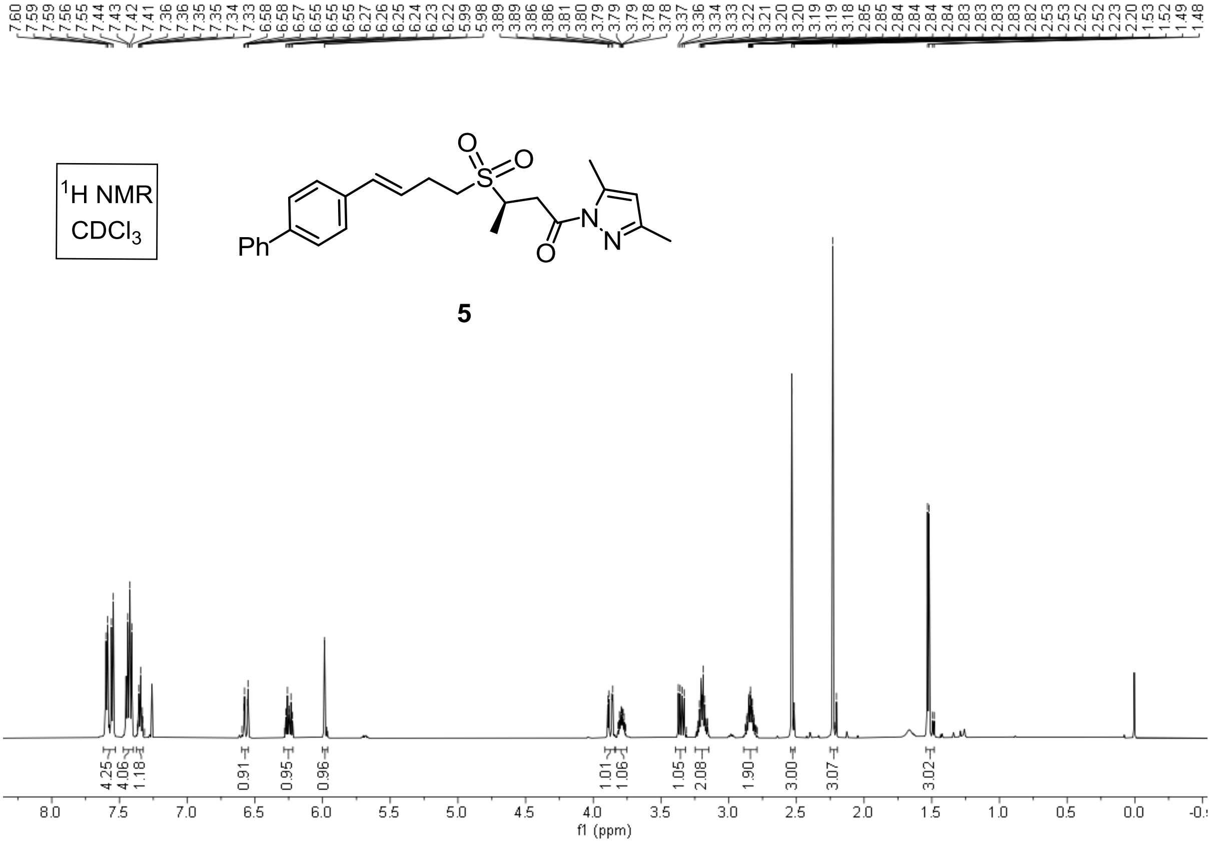
7.60 7.59 7.59 7.56 7.55 7.44 7.43 7.42 7.41 7.36 7.36 7.35 7.35 7.34 7.33 6.58 6.58 6.57 6.55 6.55 6.55 6.27 6.26 6.25 6.24 6.23 6.22 5.99 5.98 3.89 3.89 3.86 3.86 3.81 3.80 3.79 3.79 3.79 3.78 3.37 3.36 3.34 3.33 3.22 3.21 3.20 3.20 3.19 3.19 3.18 2.85 2.85 2.84 2.84 2.84 2.84 2.83 2.83 2.83 2.82 2.53 2.53 2.52 2.52 2.23 2.20 1.53 1.52 1.49 1.48
^{1}H NMR
CDCl$_3$
Ph
5
4.25 4.06 1.18 0.91 0.95 0.96 1.01 1.06 1.05 2.08 1.90 3.00 3.07 3.02
f1 (ppm)

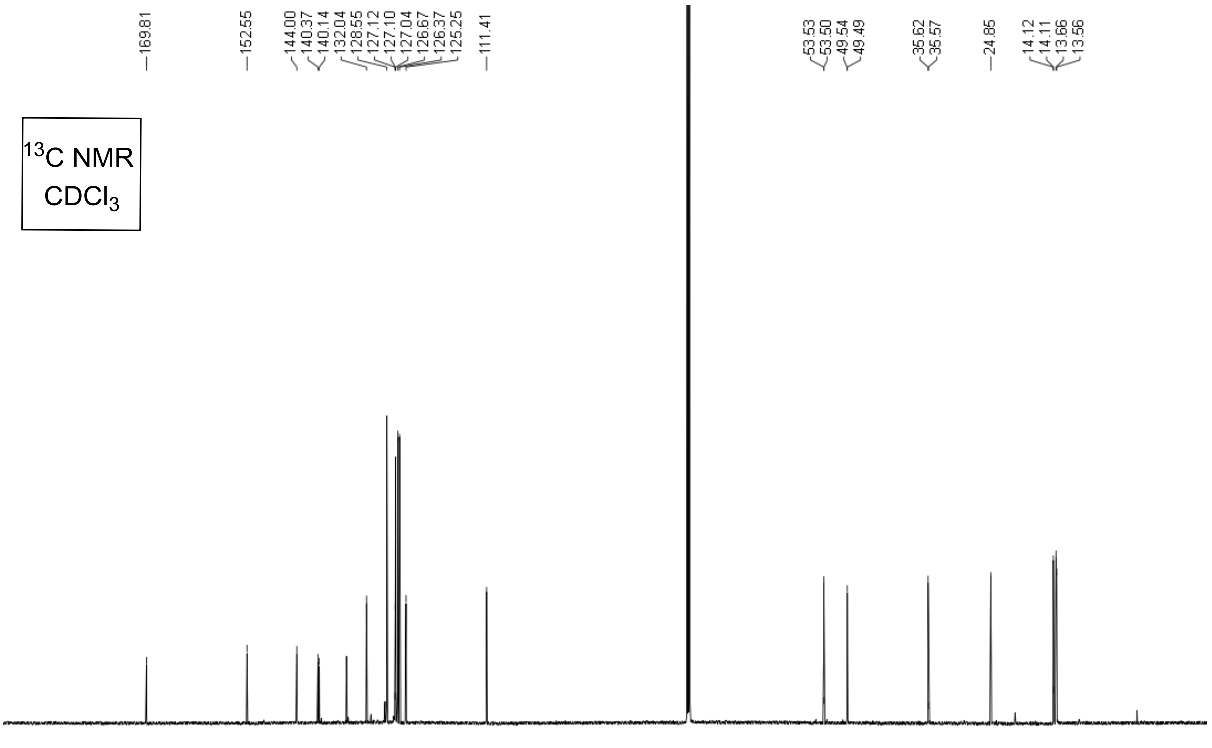
169.81
152.55
144.00 140.37 140.14 132.04 128.55 127.12 127.10 127.04 126.67 126.37 125.25
111.41
53.53 53.50 49.54 49.49
35.62 35.57
24.85
14.12 14.11 13.66 13.56
^{13}C NMR
CDCl$_3$
f1 (ppm)

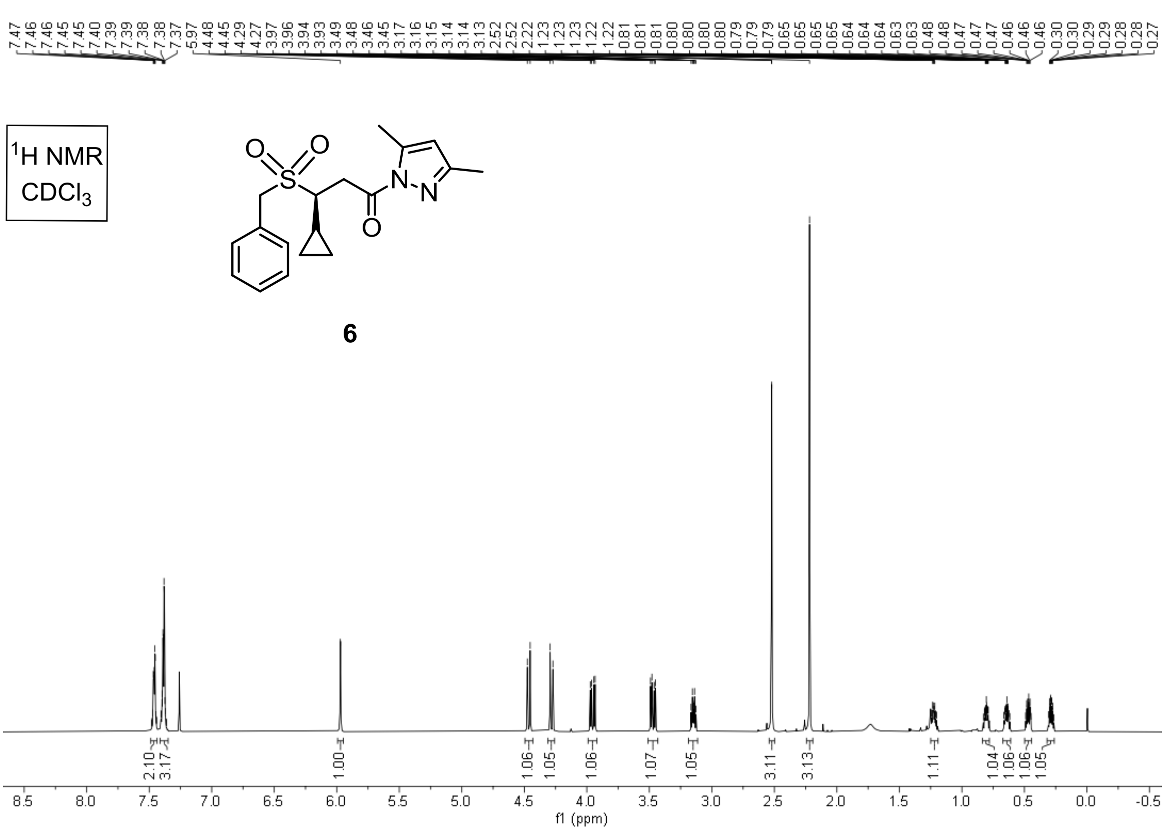

^{1}H NMR
$CDCl_3$
6

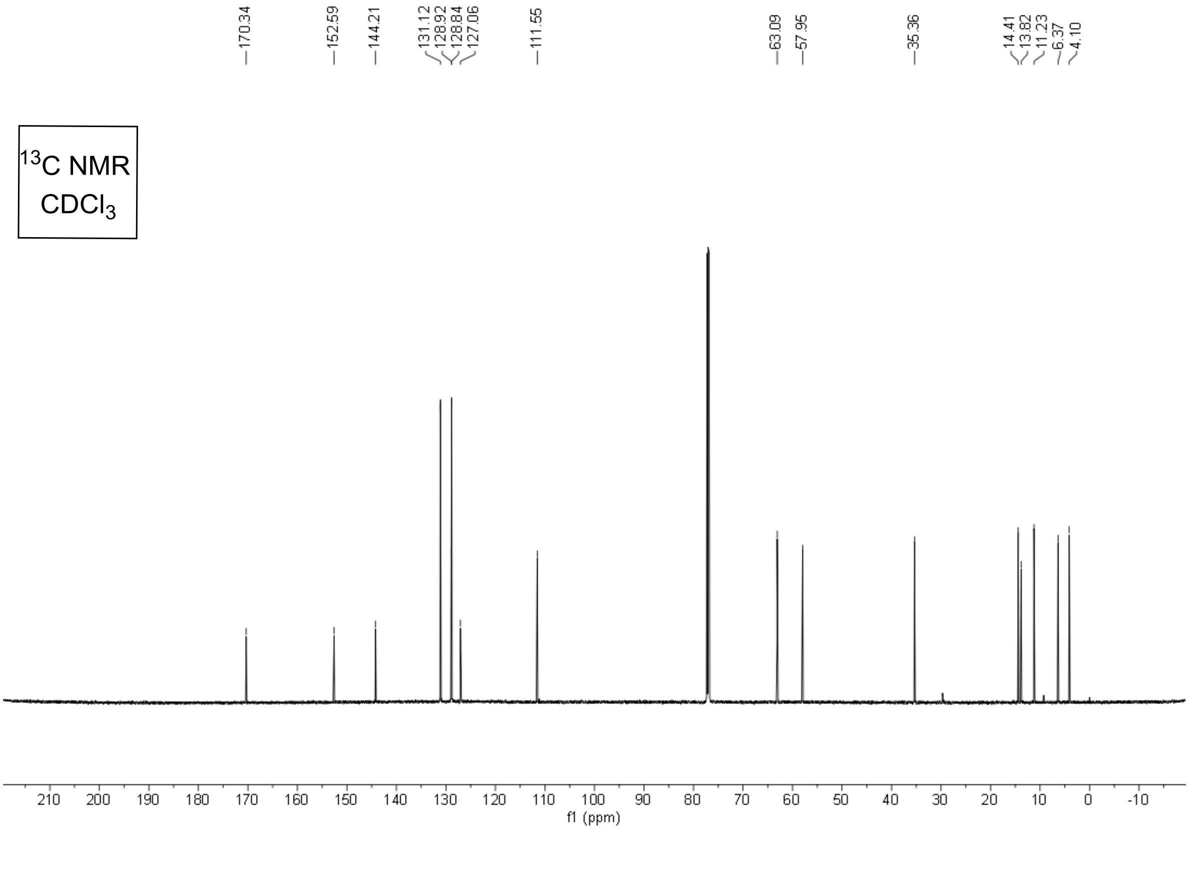

^{13}C NMR
$CDCl_3$

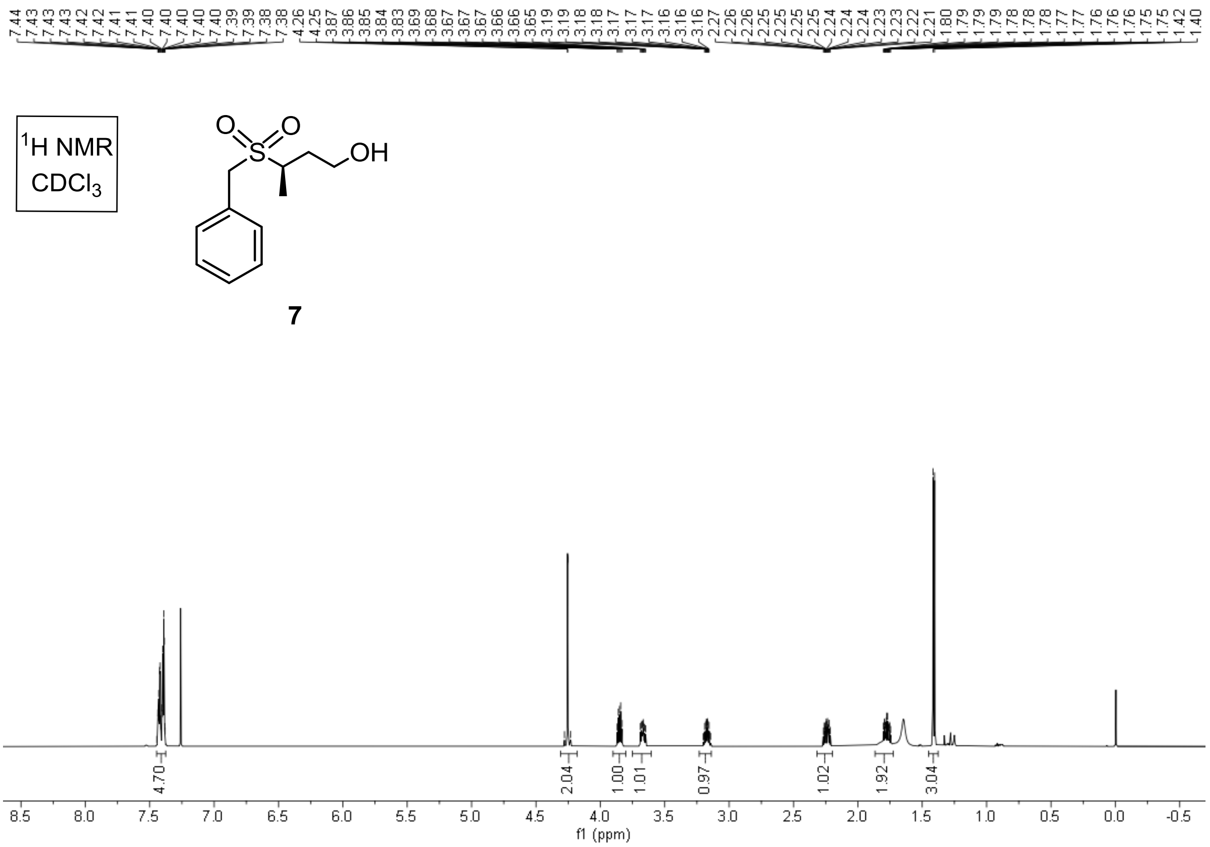

1H NMR
CDCl3
OH
7
4.70
2.04
1.00
1.01
0.97
1.02
1.92
3.04
f1 (ppm)

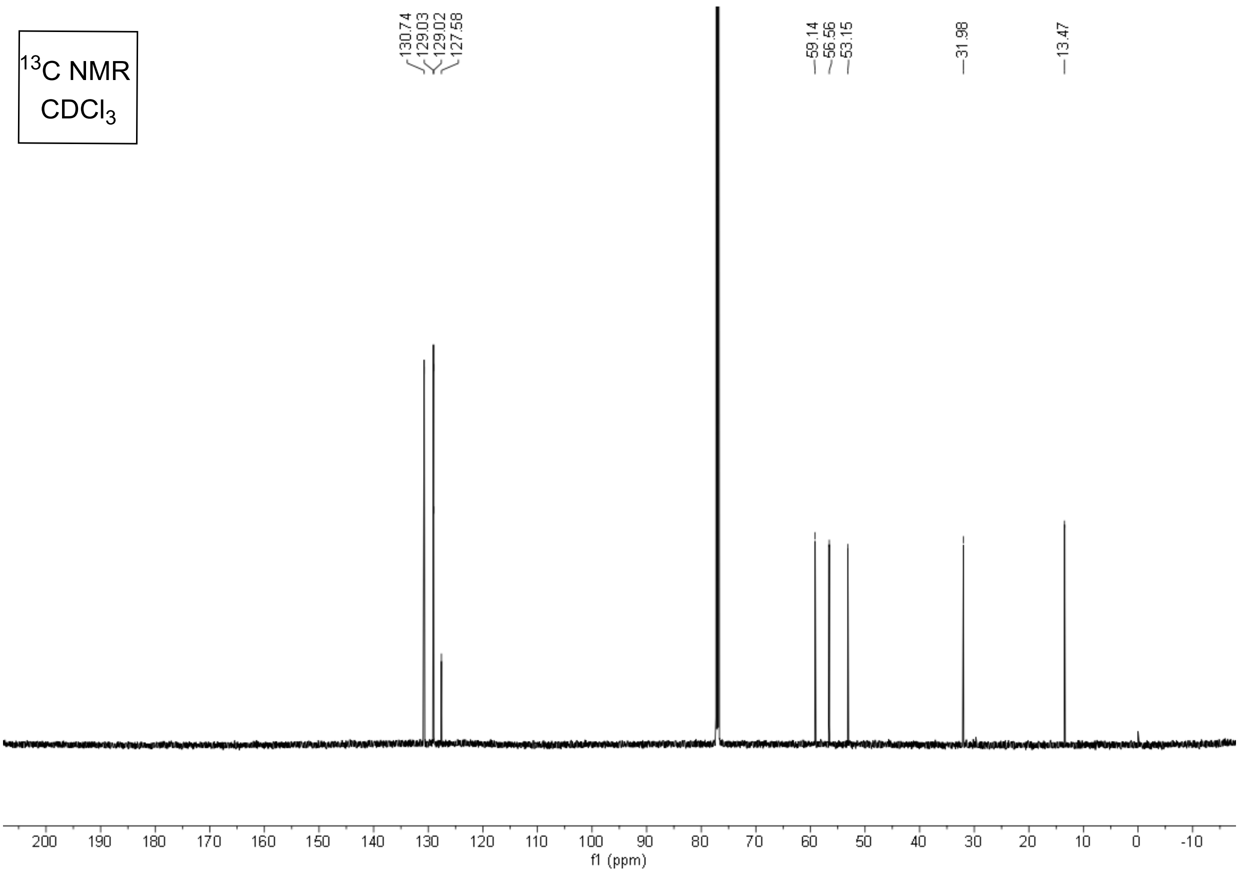

13C NMR
CDCl3
130.74
129.03
129.02
127.58
59.14
56.56
53.15
31.98
13.47
f1 (ppm)

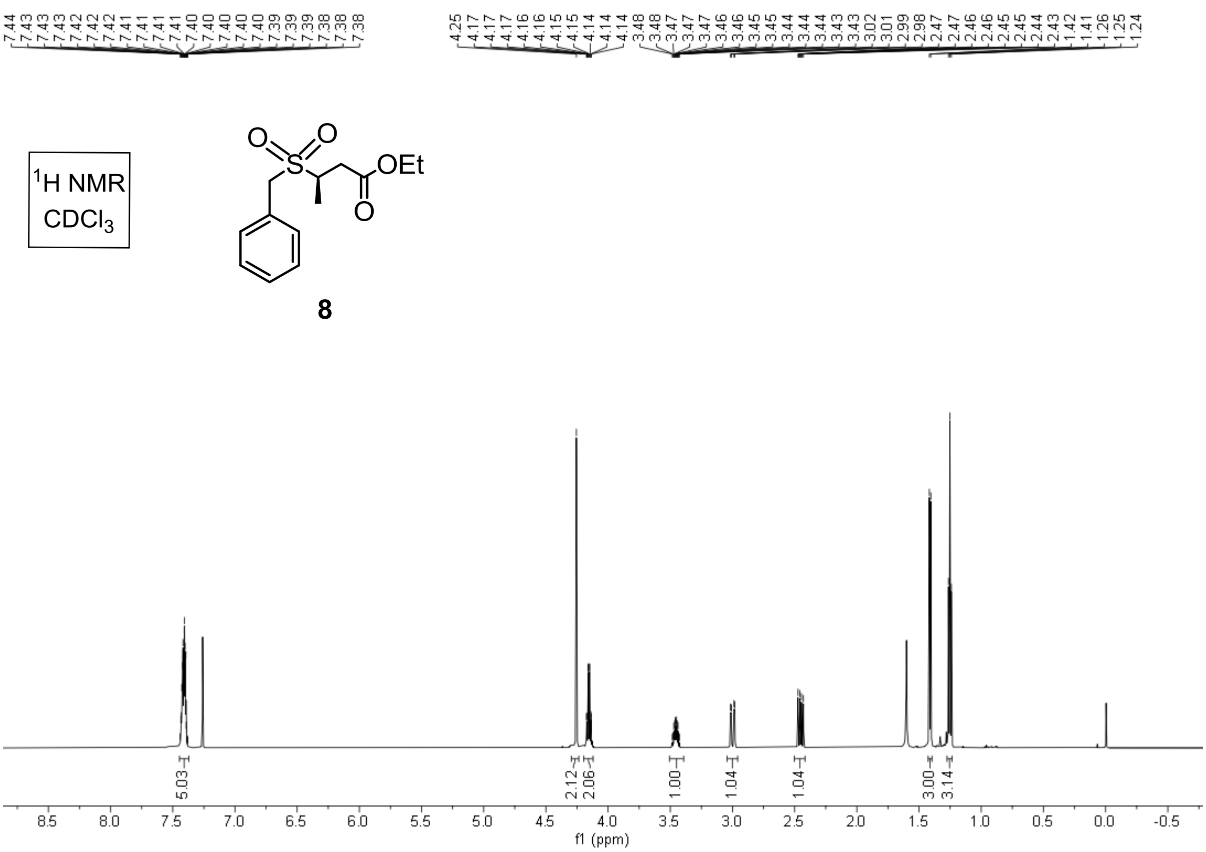
7.44 7.43 7.43 7.42 7.42 7.42 7.41 7.41 7.41 7.40 7.40 7.40 7.40 7.39 7.39 7.39 7.38 7.38 7.38
4.25 4.17 4.17 4.16 4.16 4.15 4.15 4.14 4.14 4.14 3.48 3.48 3.47 3.47 3.47 3.46 3.46 3.45 3.45 3.44 3.44 3.44 3.43 3.43 3.02 3.01 2.99 2.98 2.47 2.47 2.46 2.46 2.45 2.45 2.44 2.43 1.42 1.41 1.26 1.25 1.24
1H NMR
CDCl3
OEt
8
5.03
2.12
2.06
1.00
1.04
1.04
3.00
3.14
f1 (ppm)

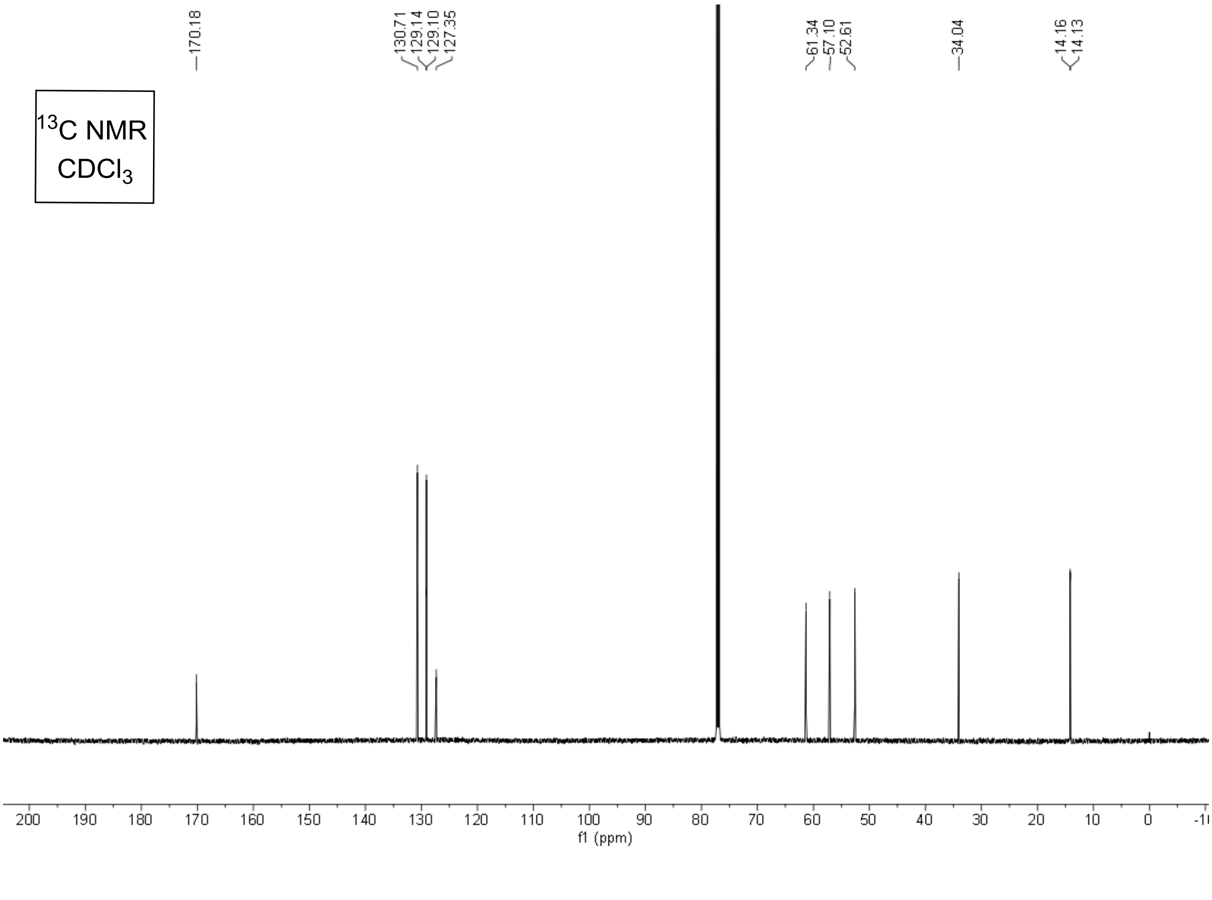
170.18
130.71 129.14 129.10 127.35
61.34 57.10 52.61
34.04
14.16 14.13
13C NMR
CDCl3
f1 (ppm)

^{1}H NMR
$CDCl_3$

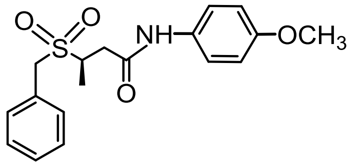

9

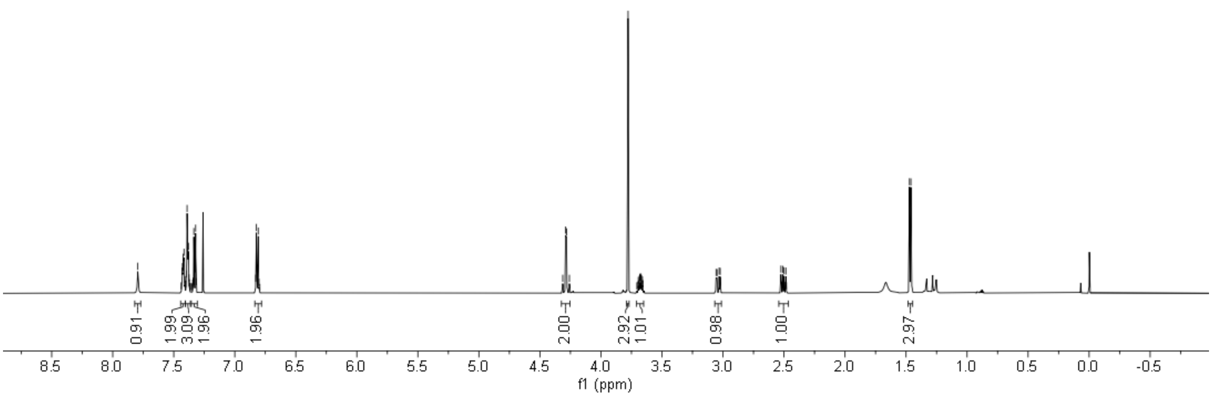

^{13}C NMR
$CDCl_3$

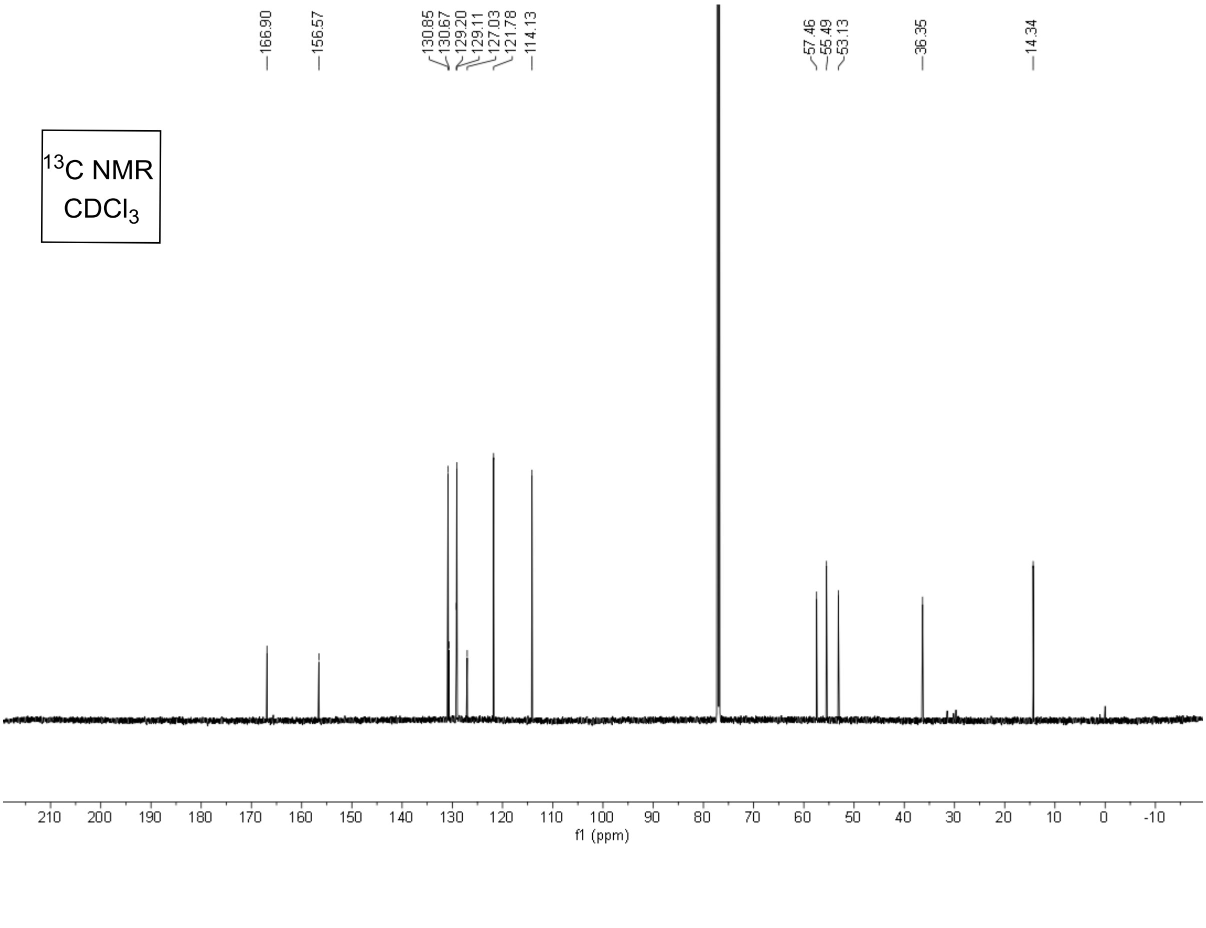

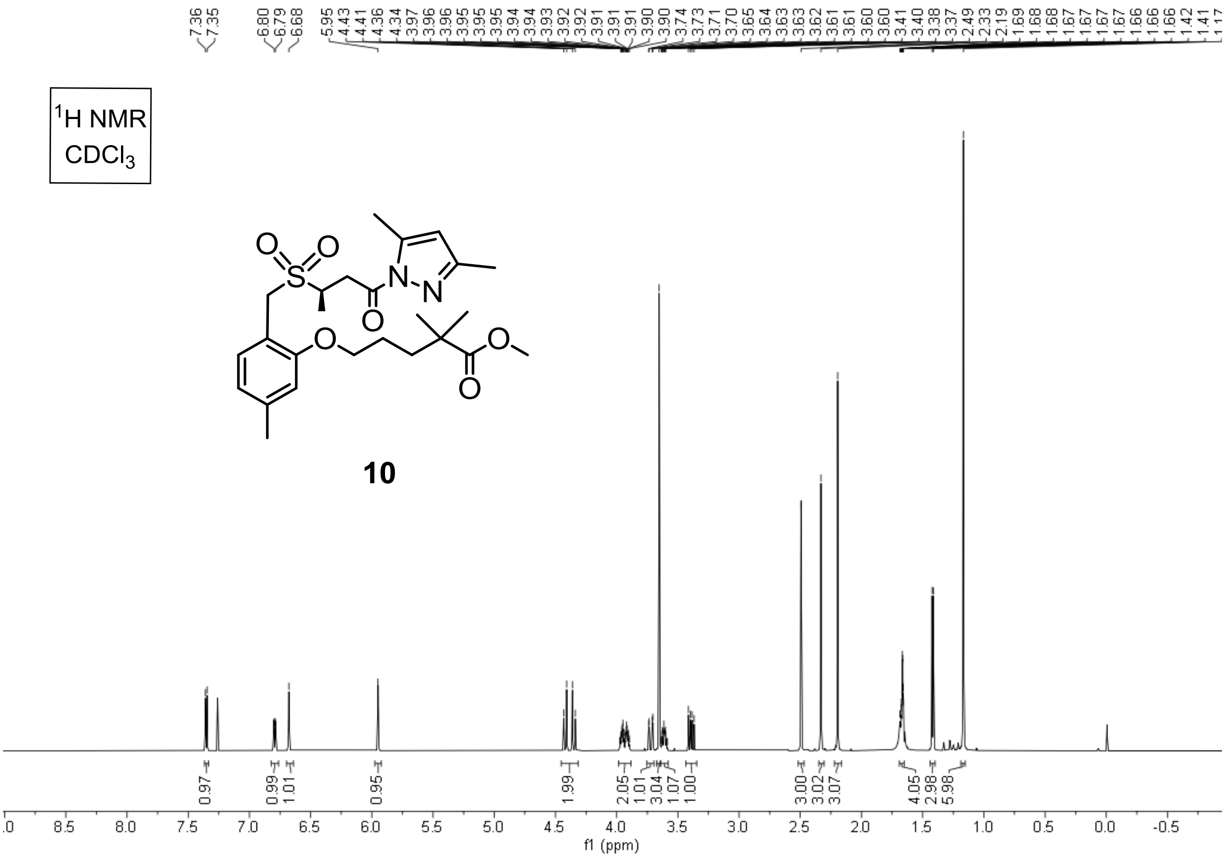

7.36 7.35 6.80 6.79 6.68 5.95 4.43 4.41 4.36 4.34 3.97 3.96 3.96 3.95 3.95 3.95 3.94 3.94 3.93 3.92 3.92 3.91 3.91 3.91 3.91 3.90 3.90 3.74 3.73 3.71 3.70 3.65 3.64 3.63 3.63 3.62 3.61 3.61 3.60 3.60 3.41 3.40 3.38 3.37 2.49 2.33 2.19 1.69 1.68 1.67 1.67 1.67 1.66 1.66 1.42 1.41 1.17
^{1}H NMR
$CDCl_3$
10
0.97 0.99 1.01 0.95 1.99 2.05 1.01 3.04 1.07 1.00 3.00 3.02 3.07 4.05 2.98 5.98
f1 (ppm)

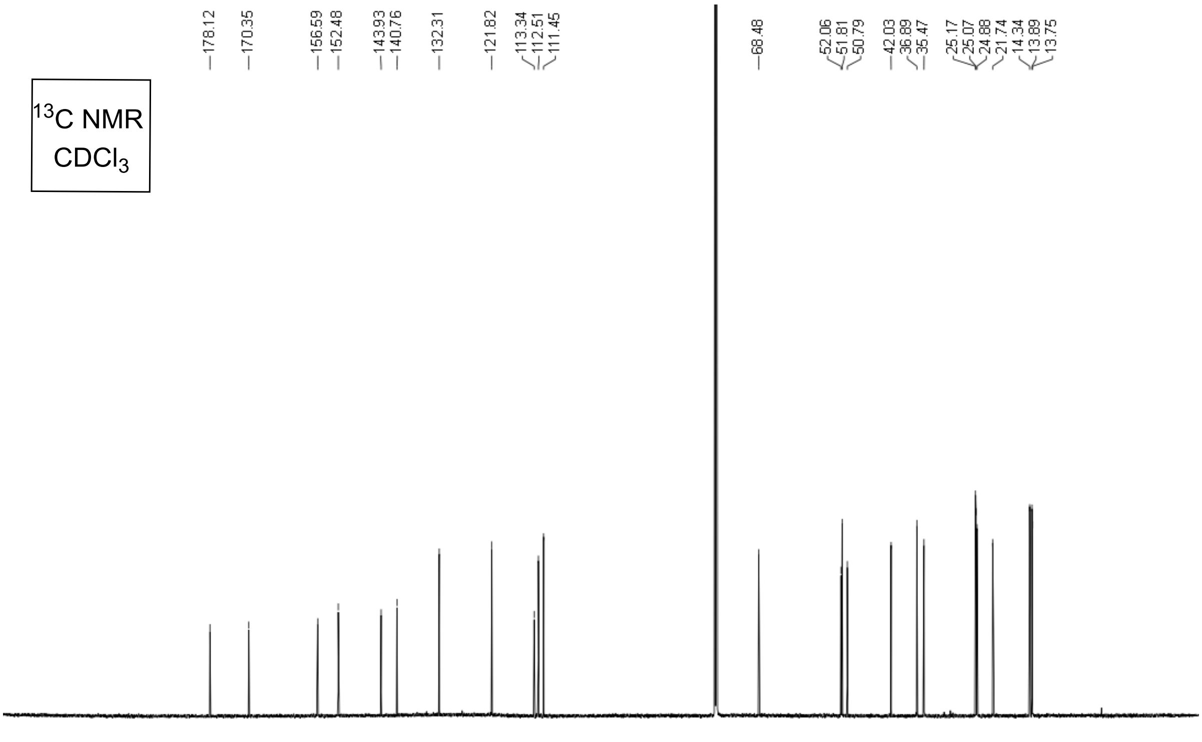

178.12 170.35 156.59 152.48 143.93 140.76 132.31 121.82 113.34 112.51 111.45 68.48 52.06 51.81 50.79 42.03 36.89 35.47 25.17 25.07 24.88 21.74 14.34 13.89 13.75
^{13}C NMR
$CDCl_3$
f1 (ppm)

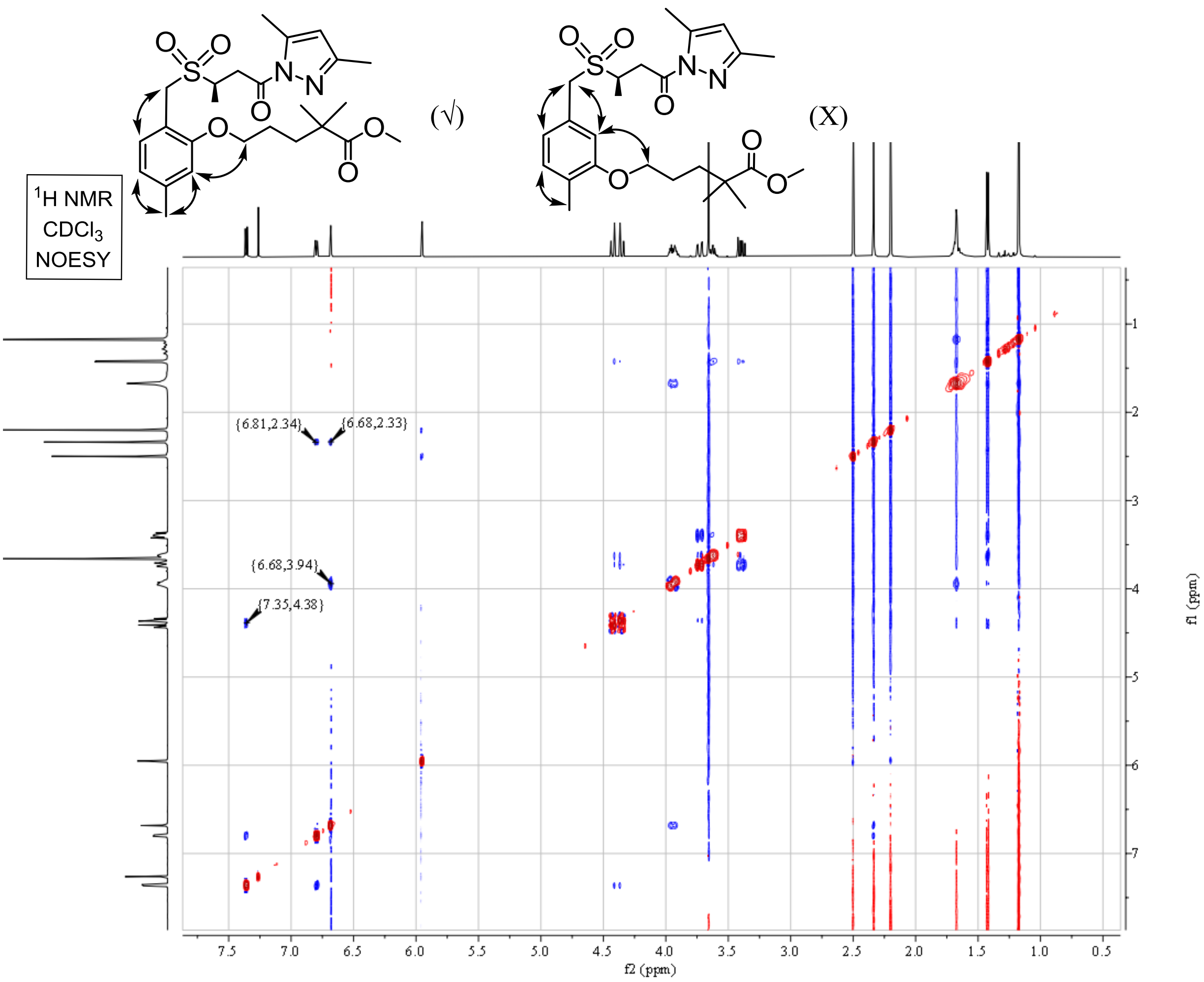
(√)
(X)
¹H NMR
CDCl3
NOESY
{6.81,2.34}
{6.68,2.33}
{6.68,3.94}
{7.35,4.38}
f2 (ppm)
f1 (ppm)

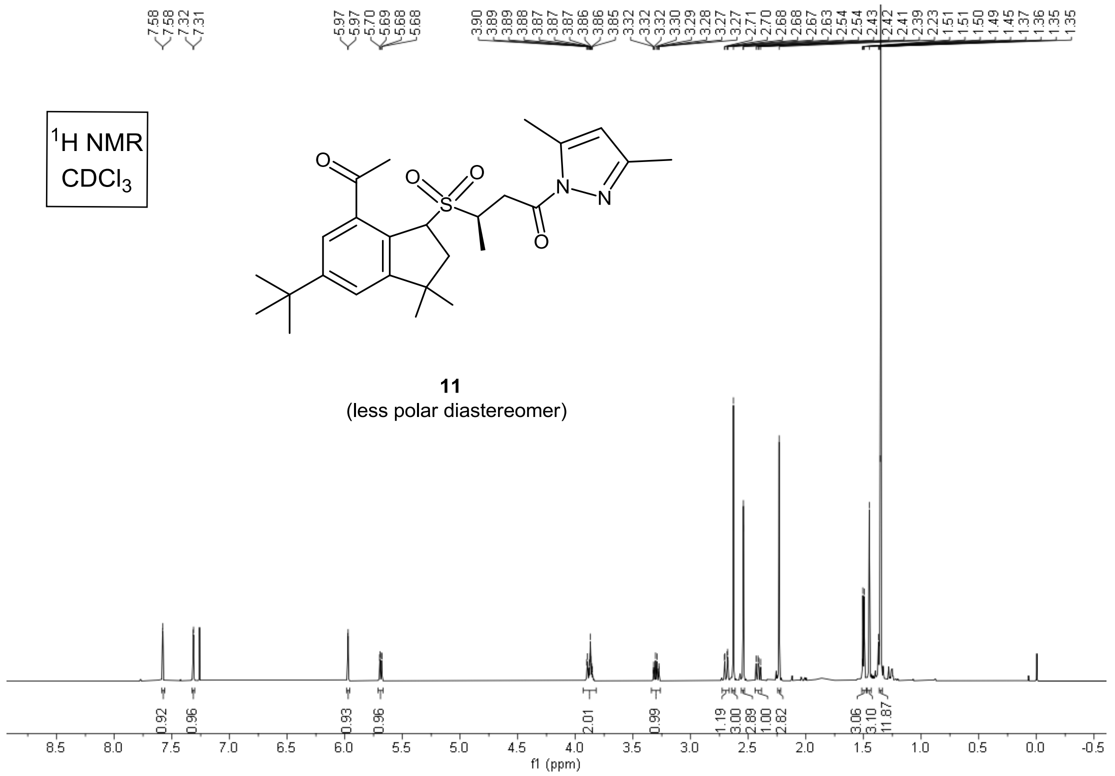

11
(less polar diastereomer)

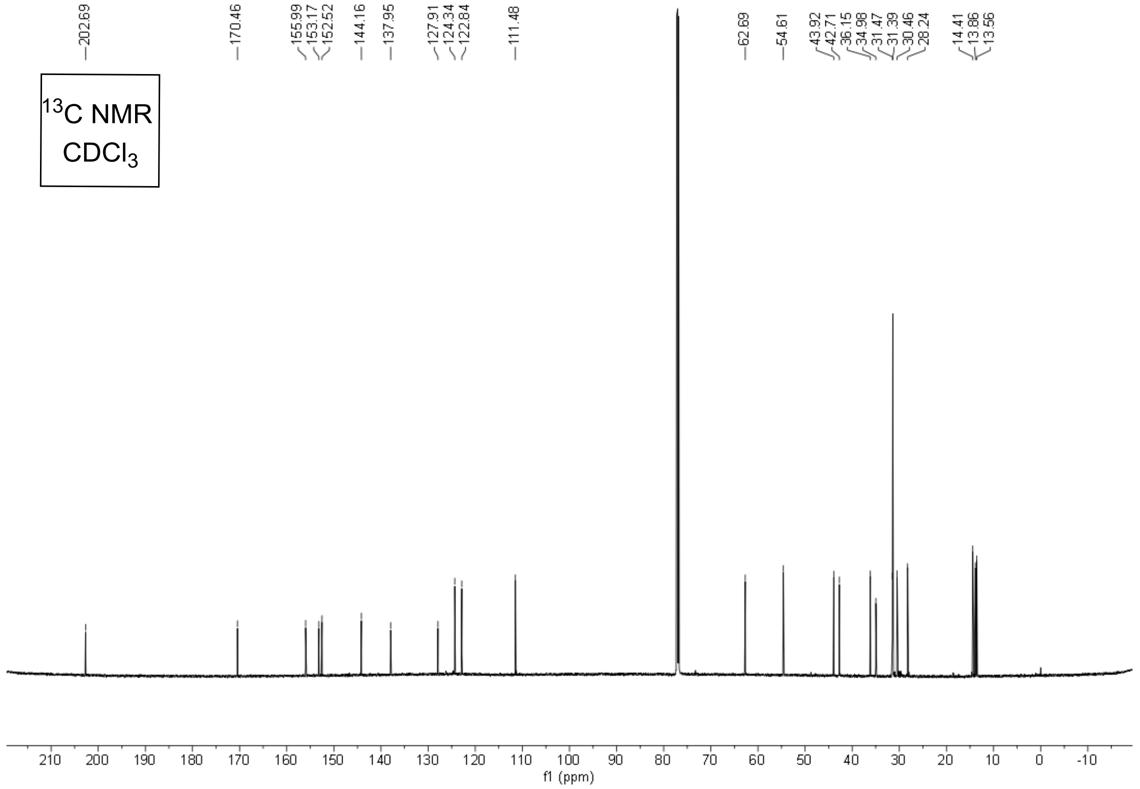

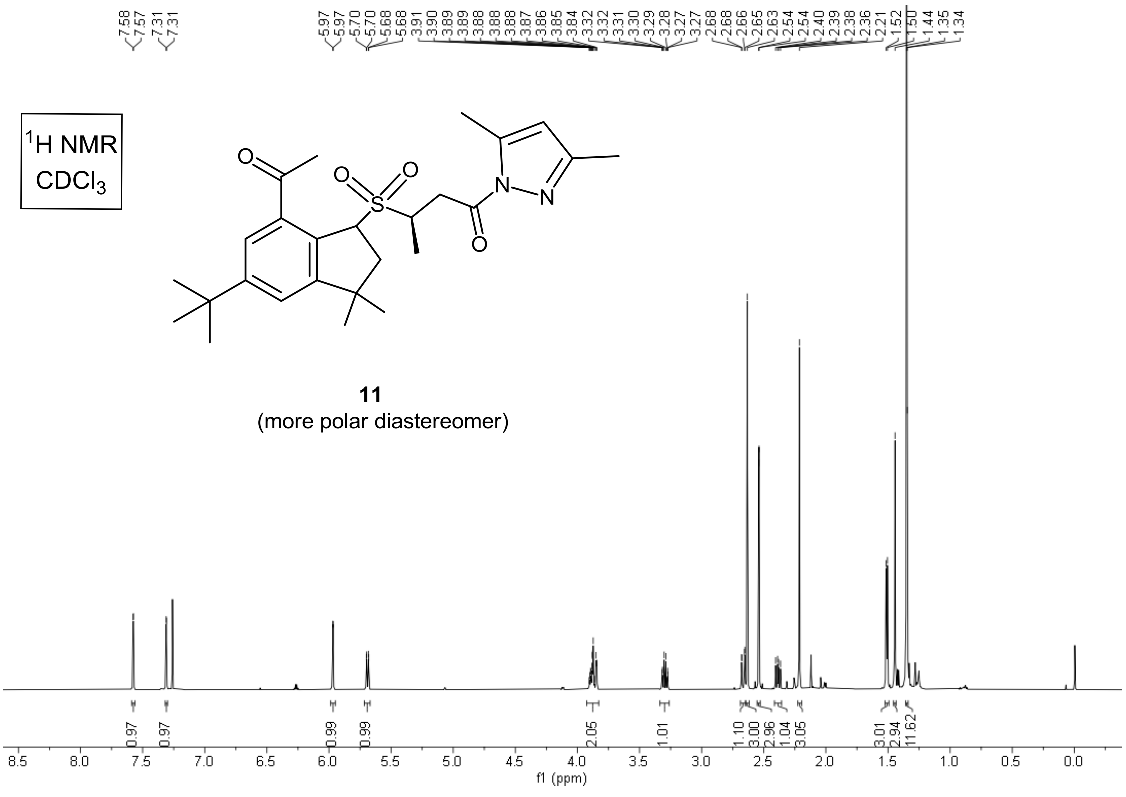

11
(more polar diastereomer)

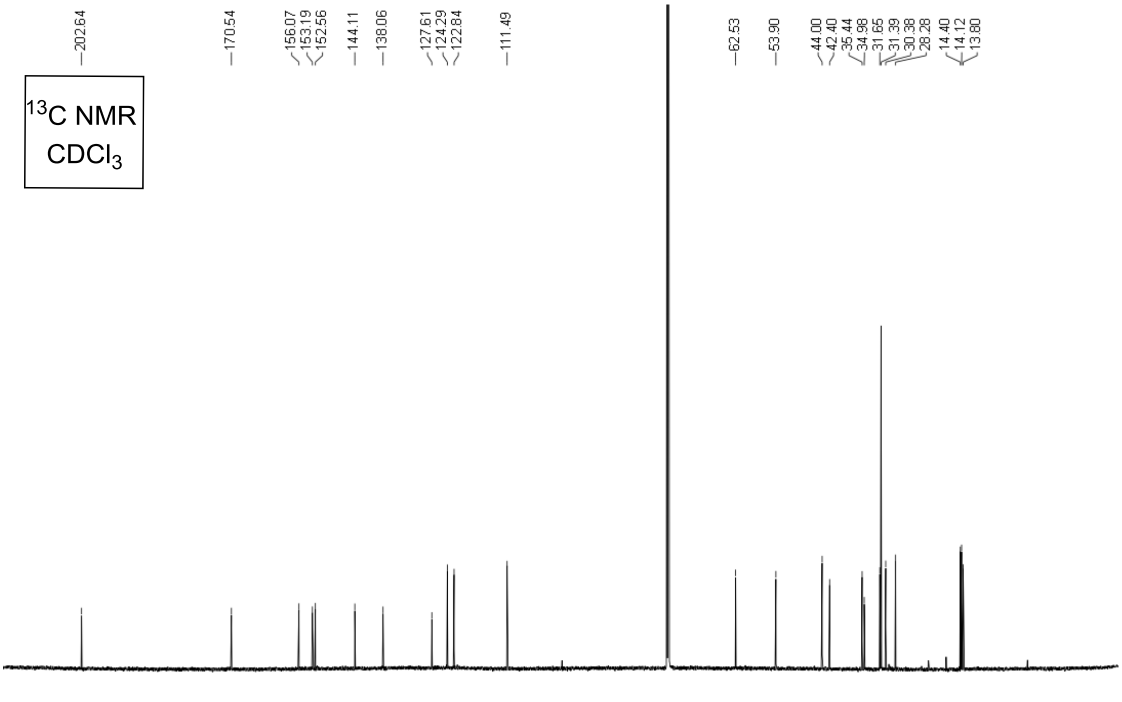

^{1}H NMR
$CDCl_3$

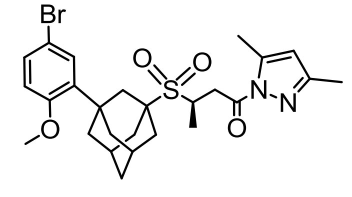

12

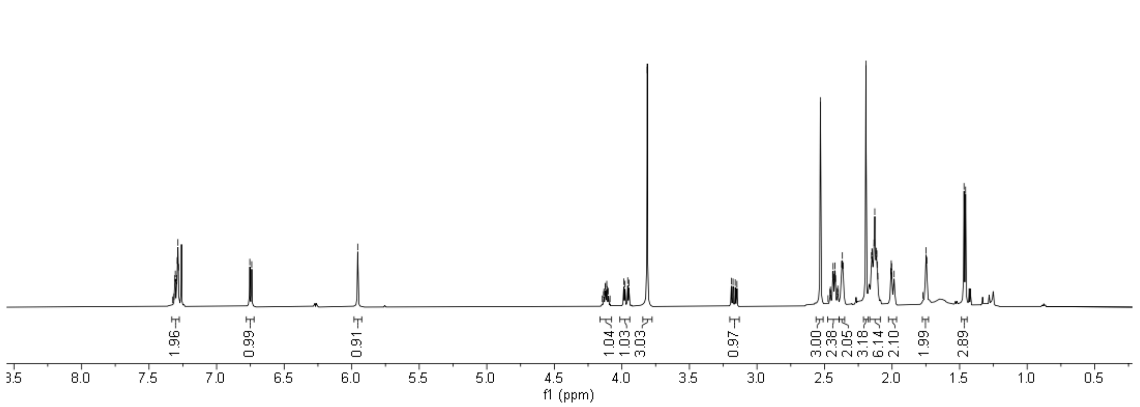

^{13}C NMR
$CDCl_3$

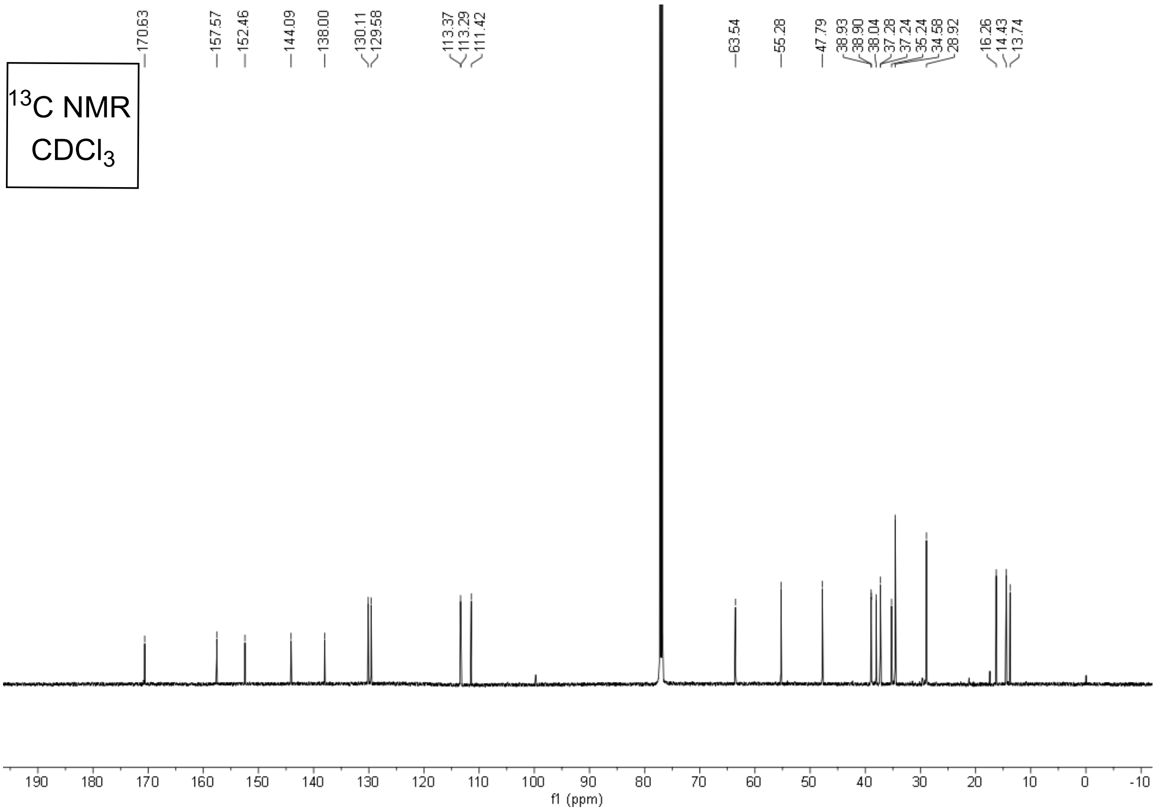

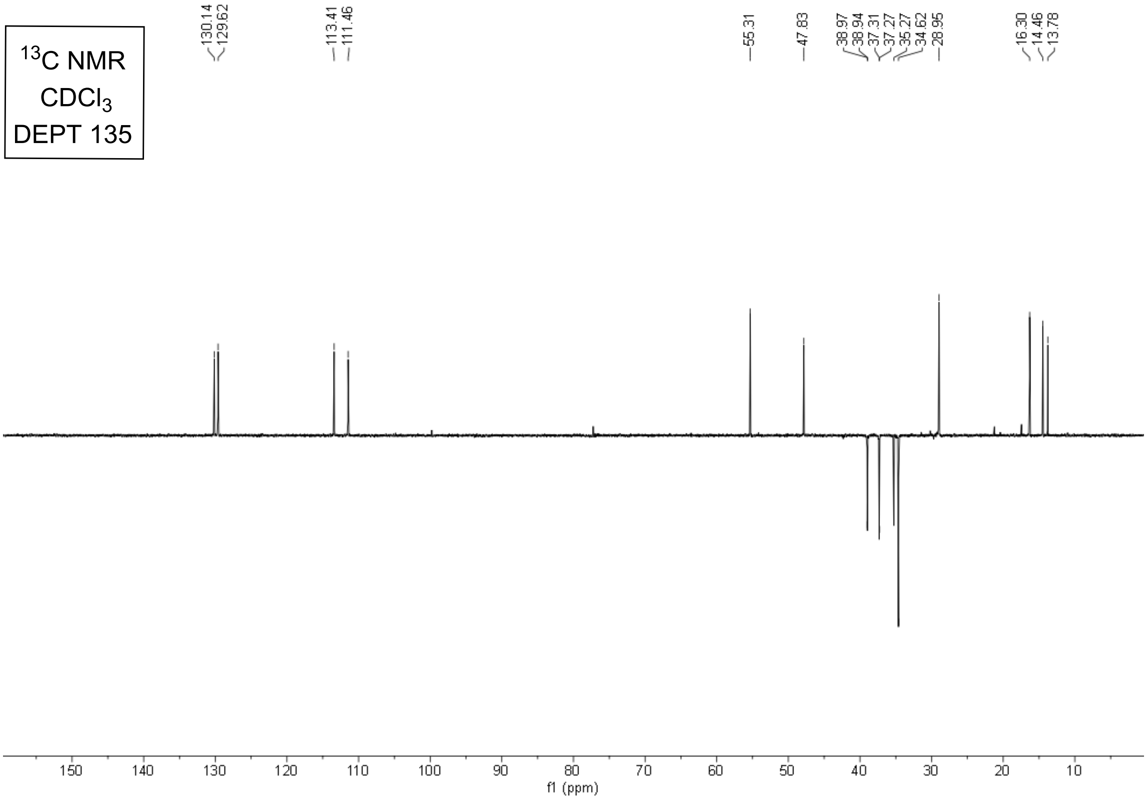

^{13}C NMR
CDCl$_3$
DEPT 135
130.14
129.62
113.41
111.46
55.31
47.83
38.97
38.94
37.31
37.27
35.27
34.62
28.95
16.30
14.46
13.78
150 140 130 120 110 100 90 80 70 60 50 40 30 20 10
f1 (ppm)

^{1}H NMR
$CDCl_3$

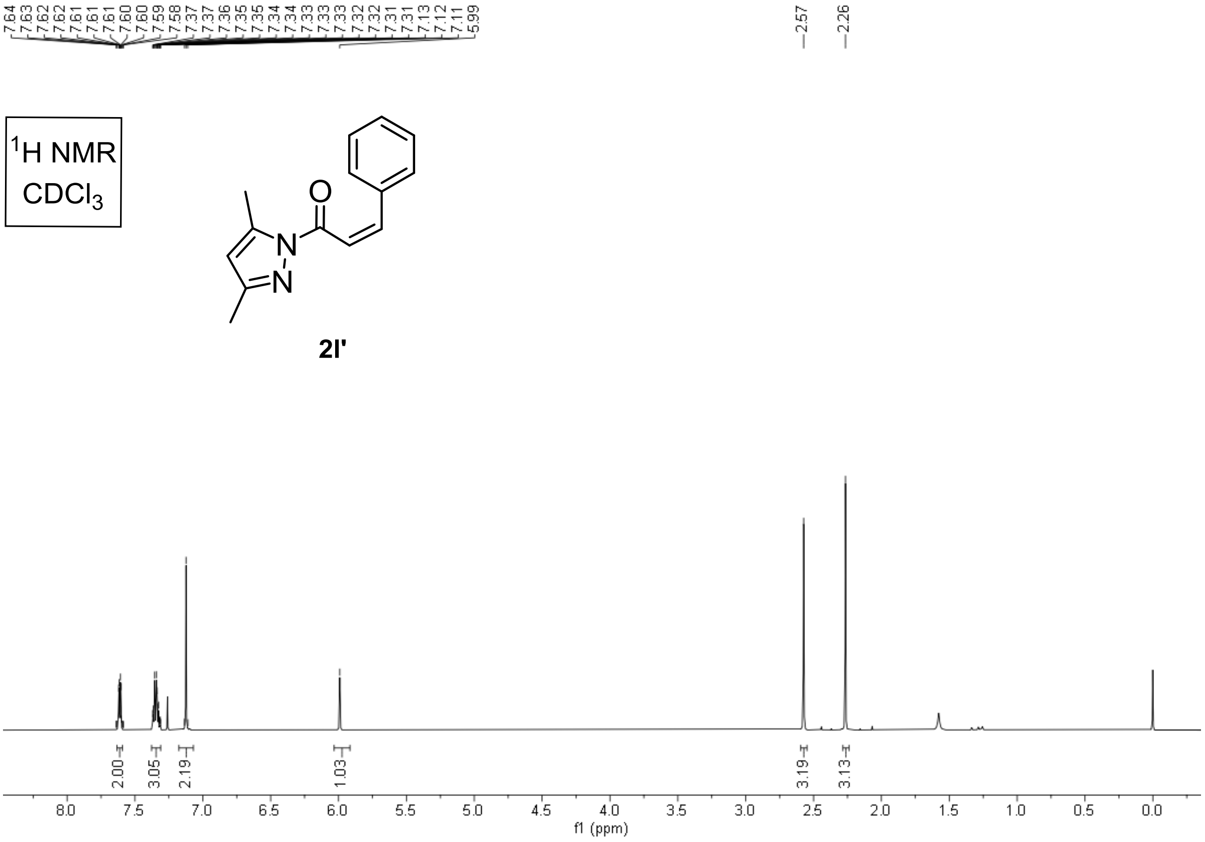

2l'

^{13}C NMR
$CDCl_3$

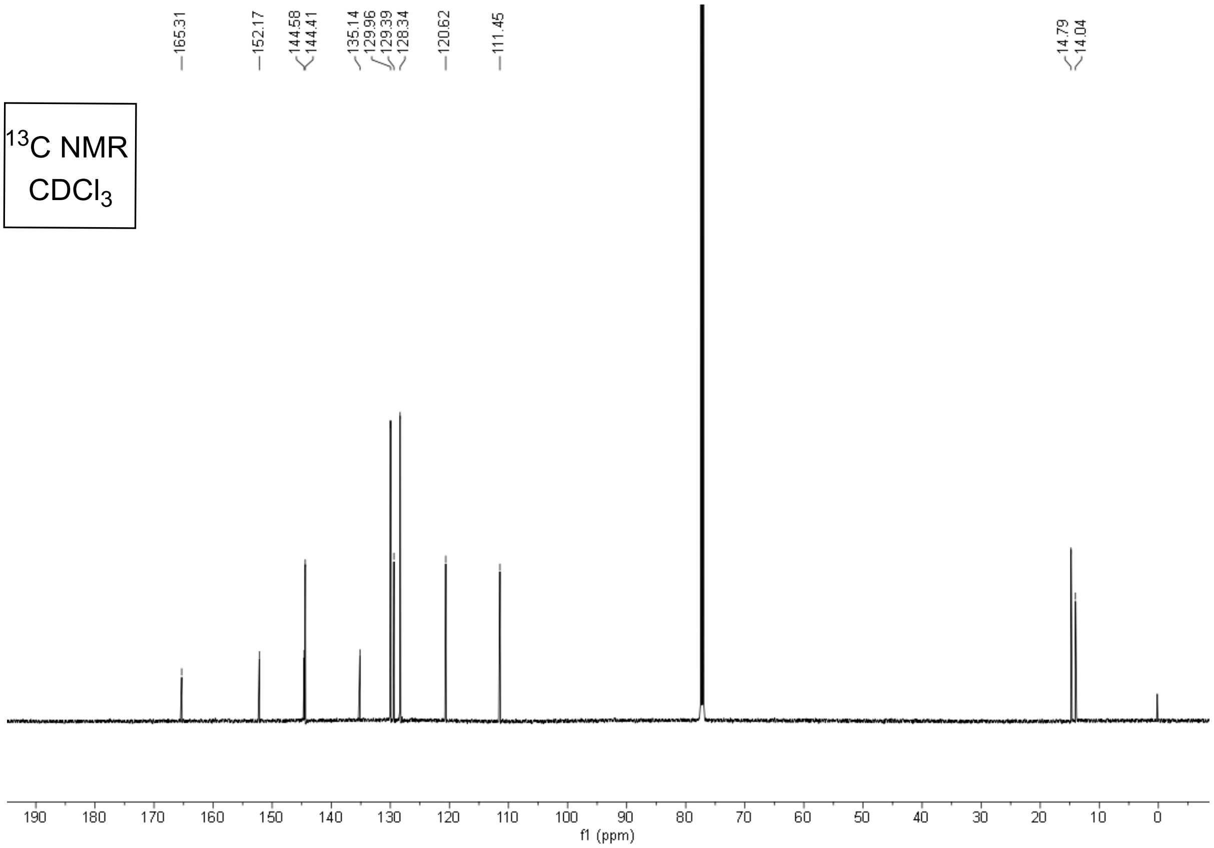

Supplementary References

1. Sibi, M. P. & Itoh, K. Organocatalysis in conjugate amine additions. Synthesis of β-amino acid derivatives. *J. Am. Chem. Soc.* **129,** 8064−8065 (2007).

2. Ma, J., Lin, J., Zhao, L., Harms, K., Marsch, M., Xie, X. & Meggers, E. Synthesis of β-substituted γ-aminobutyric acid derivatives through enantioselective photoredox catalysis. *Angew. Chem. Int. Ed.* **57**, 11193−11197 (2018).

3. Deng, C., Wang, L.-J., Zhu, J. & Tang, Y. A chiral cage-like copper(I) catalyst for the highly enantioselective synthesis of 1,1-cyclopropane diesters. *Angew. Chem. Int. Ed.* **51**, 11620−11623 (2012).

4. Liu, C., Yi, J. C., Zheng, Z. B., Tang, Y., Dai, L. X. & You, S. L. Enantioselective synthesis of 3a-amino-pyrroloindolines by copper-catalyzed direct asymmetric dearomative amination of tryptamines. *Angew. Chem. Int. Ed.* **55**, 751−754 (2016).

5. Chen, S., Wu, L., Shao, Q., Yang, G. & Zhang, W. Pd(II)-catalyzed asymmetric 1,6-conjugate addition of arylboronic acids to Meldrum's acid-derived dienes. *Chem. Commun.* **54**, 2522−2525 (2018).

6. Barnes, D. M., Ji, J., Fickes, M. G., Fitzgerald, M. A.;, King, S. A., Morton, H. E., Plagge, F. A., Preskill, M., Wagaw, S. H., Wittenberger, S. J. & Zhang, J. Development of a catalytic enantioselective conjugate addition of 1,3-dicarbonyl compounds to nitroalkenes for the synthesis of endothelin-A antagonist ABT-546. Scope, mechanism, and further application to the synthesis of the antidepressant rolipram. *J. Am. Chem. Soc.* **124**, 13097−13105 (2002).

7. Zhou, Y. Y., Wang, L. J., Li, J., Sun, X. L. & Tang, Y. Side-arm-promoted highly enantioselective ring-opening reactions and kinetic resolution of donor-acceptor cyclopropanes with amines. *J. Am. Chem. Soc.* **134**, 9066−9069 (2012).

8. Hofstra, J. L., Cherney, A. H., Ordner, C. M. & Reisman, S. E. Synthesis of

enantioenriched allylic silanes via nickel-catalyzed reductive cross-coupling. *J. Am. Chem. Soc*. **140**, 139−142 (2018).

9. Frisch, M. J., Trucks, G. W., Schlegel, H. B., Scuseria, G. E., Robb, M. A., Cheeseman, J. R., Scalmani, G., Barone, V., Mennucci, B., Petersson, G. A., Nakatsuji, H., Caricato, M., Li, X., Hratchian, H. P., Izmaylov, A. F., Bloino, J., Zheng, G., Sonnenberg, J. L., Hada, M., Ehara, M., Toyota, K., Fukuda, R., Hasegawa, J., Ishida, M., Nakajima, T., Honda, Y., Kitao, O., Nakai, H., Vreven, T., Montgomery, J. A., Jr., Peralta, J. E., Ogliaro, F., Bearpark, M., Heyd, J. J., Brothers, E., Kudin, K. N., Staroverov, V. N., Keith, T., Kobayashi, R., Normand, J., Raghavachari, K., Rendell, A., Burant, J. C., Iyengar, S. S., Tomasi, J., Cossi, M., Rega, N., Millam, J. M., Klene, M., Knox, J. E., Cross, J. B., Bakken, V., Adamo, C., Jaramillo, J., Gomperts, R., Stratmann, R. E., Yazyev, O., Austin, A. J., Cammi, R., Pomelli, C., Ochterski, J. W., Martin, R. L., Morokuma, K., Zakrzewski, V. G., Voth, G. A., Salvador, P., Dannenberg, J. J., Dapprich, S., Daniels, A. D., Farkas, O., Foresman, J. B., Ortiz, J. V., Cioslowski, J., & Fox, D. J. Gaussian 09, Revision E.01 (Gaussian, Inc., Wallingford CT). (2013).

10. Weigend, F., Furche, F. & Ahlrichs, R. Gaussian basis sets of quadruple zeta valence quality for atoms H–Kr. *J. Chem. Phys.* **119**, 12753–12762 (2003).
